# Systemic delivery of AAV-GFM1 corrects COXPD1 molecular alterations in *Gfm1*^*R671C/−*^ mice

**DOI:** 10.1038/s44321-026-00426-4

**Published:** 2026-04-17

**Authors:** Miguel Molina-Berenguer, Diego Herrero-Martínez, Antoni Vallbona-Garcia, Ferran Vila-Julià, Yolanda Cámara, África Vales, Gloria González-Aseguinolaza, Javier Torres-Torronteras, Ramon Martí

**Affiliations:** 1 Research Group on Neuromuscular and Mitochondrial Diseases, Vall d’Hebron Research Institute, Universitat Autònoma de Barcelona, and Biomedical Network Research Centre on Rare Diseases (CIBERER), Instituto de Salud Carlos III, Barcelona, Spain; 2 Institute for Bioengineering of Catalonia (IBEC), Barcelona, Spain; 3 Programa de Investigación de Terapia Génica de Enfermedades Raras, División de Medicina de ADN y ARN, Centro de Investigación Médica Aplicada (CIMA), Pamplona, Spain; 4 Barcelonaβeta Brain Research Center (BBRC), Pasqual Maragall Foundation, Barcelona, Spain; 5 Hospital del Mar Research Institute, Barcelona, Spain

**Keywords:** Genetics, Gene Therapy & Genetic Disease

## Abstract

Hepatoencephalopathy due to mutations in the nuclear gene *GFM1*, known as combined oxidative phosphorylation (OXPHOS) deficiency type I (COXPD1), is an autosomal recessive mitochondrial disease caused by defects or deficiency of the mitochondrial translation elongation factor G1 (EFG1), with no currently available cure. Patients with COXPD1 develop a severe encephalopathy, sometimes combined with liver failure, with neonatal onset and rapid progression that normally causes premature death. The *Gfm1*^*R671C/−*^ mouse recapitulates the COXPD1 molecular phenotype in liver and brain, with drastic reduction of EFG1 levels, impaired mitochondrial translation, and OXPHOS deficiency. We conducted a gene therapy study using two different recombinant adeno-associated virus (rAAV) vectors targeting the liver or the brain to introduce the human *GFM1* gene into 6-week-old *Gfm1*^*R671C/−*^ mice. Successful transduction of the liver and the brain was observed after four weeks, entailing substantial recovery from mitochondrial EFG1 depletion and OXPHOS correction in both tissues, which demonstrates that transgenic human EFG1 is functional in mouse mitochondrial translation. Our study constitutes the first evidence supporting AAV-mediated gene therapy as a potential treatment for COXPD1.

## Synopsis

COXPD1 is a fatal disorder caused by mutations in GFM1, encoding the mitochondrial translation factor EFG1. The systemic administration of two liver- and brain-targeted rAAV vectors carrying the human GFM1 gene to a murine model of the disease corrected its molecular phenotype.

- Successful transduction was observed in the liver (using the AAV9 serotype) and in the brain (using the AAV9P31 serotype).
- The transgene product, the human EFG1 protein, was imported into mouse mitochondria, where it proved functional.
- As a result, the COXPD molecular phenotype (OXPHOS dysfunction) was almost fully corrected in the liver and the brain.
- AAV-mediated gene therapy constitutes a potential curative treatment for COXPD1 that deserves further investigation.

## Abstract (web summary)

COXPD1 is a fatal disorder caused by mutations in GFM1, encoding the mitochondrial translation factor EFG1. The systemic administration of two liver- and brain-targeted rAAV vectors carrying the human GFM1 gene to a murine model of the disease corrected its molecular phenotype.

## Graphical abstract

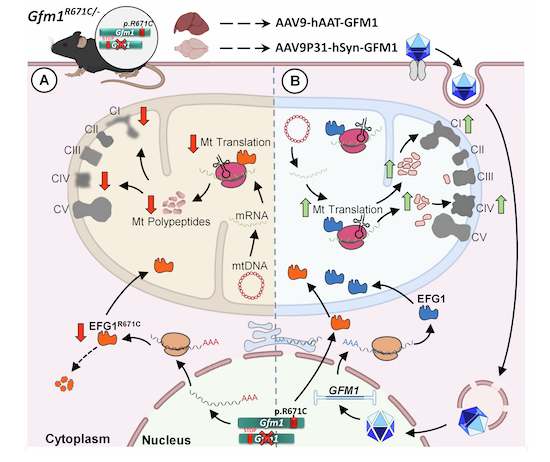

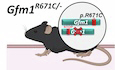

The paper explained **Problem**

Combined Oxidative Phosphorylation Deficiency type 1 (COXPD1) is a mitochondrial disorder caused by mutations in *GFM1*, the nuclear gene encoding the mitochondrial translation elongation factor G1 (EFG1). COXPD1 is fatal during infancy in most patients, with no currently available cure.

**Results**

Using a murine model of the disease, the administration of an AAV9 vector (targeted to the liver) and an AAV9P31 vector (targeted to the CNS) carrying the human *GFM1* gene efficiently transduced the target cells, leading to expression of EFG1. This factor was transferred to mitochondria, and the OXPHOS complex I and IV deficiency was almost fully corrected in the target tissues.

**Impact**

Patients with COXPD1 are currently treated with palliative and symptomatic treatments, but a curative treatment does not exist, and patients die during the first few months or years of life. This in vivo preclinical study supports the plausibility of gene therapy for COXPD1, opening a new therapy approach for this devastating disorder.

## Introduction

The nuclear *GFM1* gene encodes the mitochondrial translation elongation factor G1 (EFG1). Since its characterization in 2001 (Gao et al, 2001), several pathogenic mutations in *GFM1* have been found to cause a hepatoencephalopathy known as Combined Oxidative Phosphorylation Deficiency type 1 (COXPD1, [MIM:609060]), a serious autosomal recessive disease with at least 41 reported patients (Aleksic et al, 2023; Antonicka et al, 2006; Balasubramaniam et al, 2012; Barcia et al, 2020; Bravo-Alonso et al, 2019; Brito et al, 2015; Calvo et al, 2012; Coenen et al, 2004; Galmiche et al, 2012; Khan et al, 2022; Kohda et al, 2016; Nayan et al, 2024; Ravn et al, 2015; Simon et al, 2017; Smits et al, 2011; Su and Wang, 2020; Valente et al, 2007; You et al, 2020).

In the elongation phase of mitochondrial translation, EFG1 catalyzes the translocation of the peptidyl-tRNA from the acceptor (A) to the peptidyl (P) site in the mitoribosome. Simultaneously, the tRNA in the P site that has transferred its amino acid to the mitochondrial polypeptide being elongated is moved to the exit (E) site, making possible the peptide bond formation and the progression to the next mRNA codon (Nierhaus, 1996). *GFM1* mutations cause EFG1 loss of function and/or protein destabilization and degradation, resulting in impaired mitochondrial translation and combined OXPHOS system defect (COXPD), as observed in muscle, liver, and cultured fibroblasts from patients (Antonicka et al, 2006; Smits et al, 2011; Valente et al, 2007).

COXPD1 is clinically characterized by a neonatal severe encephalopathy, usually accompanied by lactic acidosis and sometimes presenting with feeding difficulties, developmental delay, dystonia, West’s syndrome, and brain abnormalities on nuclear magnetic resonance (MRI). Liver failure occurs in about a quarter of patients, leading to premature death in almost all cases. The disease progression is fast and patients usually die in the first days or months of life, although some long-term survival cases have been reported (Aleksic et al, 2023; Barcia et al, 2020; Bravo-Alonso et al, 2019; Brito et al, 2015; Galmiche et al, 2012; Khan et al, 2022; Nayan et al, 2024; Simon et al, 2017). Nowadays, there are no therapies for COXPD1 beyond palliative treatments common to other mitochondrial disorders (Pompei and Pompei, 2019; Smeitink et al, 2006; Uittenbogaard and Chiaramello, 2014).

The human EFG1 protein is highly homologous to ortholog proteins from prokaryotes and other eukaryotic organisms (Gao et al, 2001). We previously generated a genetically modified mouse, *Gfm1*^*R671C/−*^ (KI/KO), carrying a knockout allele (*Gfm1*^*−*^, KO) and the p.R671C mutation, found in at least ten patients who survived more than one year, in the other allele (*Gfm1*^*R671C*^, KI). *Gfm1*^*R671C/−*^ mice showed a drastic reduction of EFG1 protein levels in liver and brain mitochondria, which significantly reduced the mitochondrial translation rates in both tissues. In addition, these mice developed COXPD, i.e., low levels of assembled complexes I and IV, causing a reduction in their enzyme activities (Molina-Berenguer et al, 2022).

Monogenic recessive diseases are ideal candidates for their treatment with gene therapy. The COXPD1 molecular phenotype has been corrected in patients’ fibroblasts transfected with the gene encoding the recombinant wild-type EFG1 protein (Smits et al, 2011; Valente et al, 2007). This was important not only for diagnostic purposes but also as an experimental clue supporting the plausibility of a gene therapy approach for COXPD1.

Recombinant adeno-associated virus (rAAV) vectors are currently the preferred delivery system for in vivo gene therapy. Several preclinical studies have been reported using rAAV for the treatment of mitochondrial diseases in mouse models (Hanaford et al, 2022), and the 5-year follow-up study of the 2 phase 3 clinical studies for LHON (Leber Hereditary Optic Neuropathy), caused by mutations on mitochondrial DNA (mtDNA) genes, has been recently published (Yu-Wai-Man et al, 2025).

In this study, we developed two rAAV vectors carrying the human *GFM1* coding sequence transcriptionally targeted to two different tissues: an AAV9 vector carrying the human hepatocyte promoter α-1-antitrypsin (hAAT) fused to the apolipoprotein E enhancer (AAV9-hAAT-GFM1), and an AAV9P31 vector carrying the human neuronal promoter α-synapsin (hSyn) (AAV9P31-hSyn-GFM1). In two independent approaches, each vector was intravenously administered to six-week-old *Gfm1*^*R671C/−*^ mice, and 4 weeks later their efficacies were analyzed in their respective target tissues. Each vector provided substantial rAAV transduction of the liver and brain, respectively, which correlated with *GFM1* expression. EFG1 levels were partially restored in *Gfm1*^*R671C/−*^ liver and brain, leading to almost complete correction of the COXPD molecular phenotype.

This study demonstrated successful AAV-mediated transduction of the target tissues and showed the therapeutic protein to be functional and capable of restoring the downstream activities that are impaired in patients’ mitochondria, constituting the first in vivo preclinical evidence supporting the feasibility of gene addition as a potential treatment for COXPD1.

## Results

### The COXPD molecular phenotype is maintained in 30-week-old *Gfm1*^*R671C/−*^ mice

**Figure EV1:**
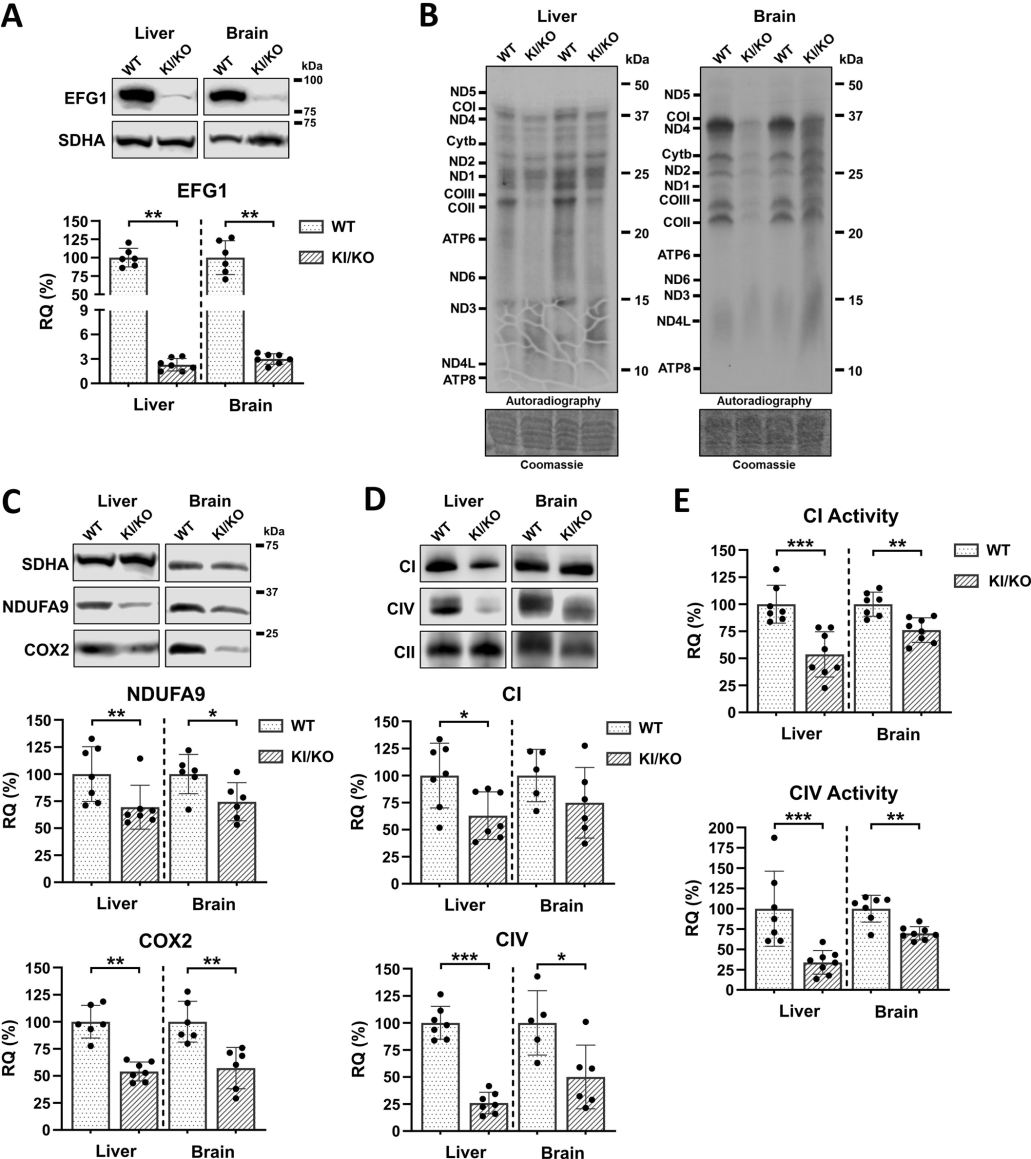
Liver and brain COXPD molecular phenotype in 30-week-old *Gfm1*^*R671C/−*^ mice. (**A**) Mitochondrial EFG1 immunodetection in liver and brain by western blot SDS-PAGE. EFG1 protein levels were normalized to SDHA levels (used as mitochondrial loading control). (**B**) In organello mitochondrial translation performed using fresh liver and brain mitochondria. De novo synthesized proteins were radiolabelled with ^35^S and detected through autoradiography. A Coomassie-stained electrophoresis gel was used as a protein loading control. (**C**) Western blot SDS-PAGE of NDUFA9 (CI subunit, nDNA encoded) and COX2 (CIV subunit, mtDNA encoded) in liver and brain mitochondria. Protein levels were normalized to SDHA levels. (**D**) Assembled complex I and IV levels analyzed by western blot BN-PAGE on liver and brain mitochondria. CII levels were used as mitochondrial protein loading control. (**E**) Spectrophotometric determination of complex I and IV enzyme activities, normalized to citrate synthase activity. Dots represent results for each mouse, and relative quantifications (RQ) are expressed as a percentage of the wild-type mean; bars represent the mean RQ ( ± SD). The exact *n* for each experiment is indicated in Appendix Table S1. Asterisks indicate statistical differences between the WT and KI/KO groups (*P < 0.05, **P < 0.01, ***P < 0.001, Mann–Whitney *U* test). RQ relative quantification. Source data are available online for this figure.

Our first characterization of the *Gfm1*^*R671C/−*^ mouse model performed at 8 weeks of age showed EFG1 depletion, mitochondrial translation impairment, and COXPD (reduced assembled complex I and IV levels and enzyme activities) in the liver and brain (Molina-Berenguer et al, 2022). In this study, we show that this molecular phenotype is maintained at 30 weeks of age, as all these alterations remain present in *Gfm1*^*R671C/−*^ mice at this age in similar degrees as those observed in younger mice: EFG1 depletion (Fig. EV1A), impaired mitochondrial translation (Fig. EV1B), reduced OXPHOS subunits (Fig. EV1C), reduced assembled OXPHOX complexes (Fig. EV1D), and dysfunctional respiratory complex I and IV enzyme activities (Fig. EV1E) (Molina-Berenguer et al, 2022). This observation provides a wide temporal window for testing potential therapies, as mouse aging does not reverse the COXPD phenotype of the model, at least until 30 weeks of age.

### Lentiviral transduction reverses EFG1 depletion and COXPD phenotype in patients’ fibroblasts

**Figure EV2:**
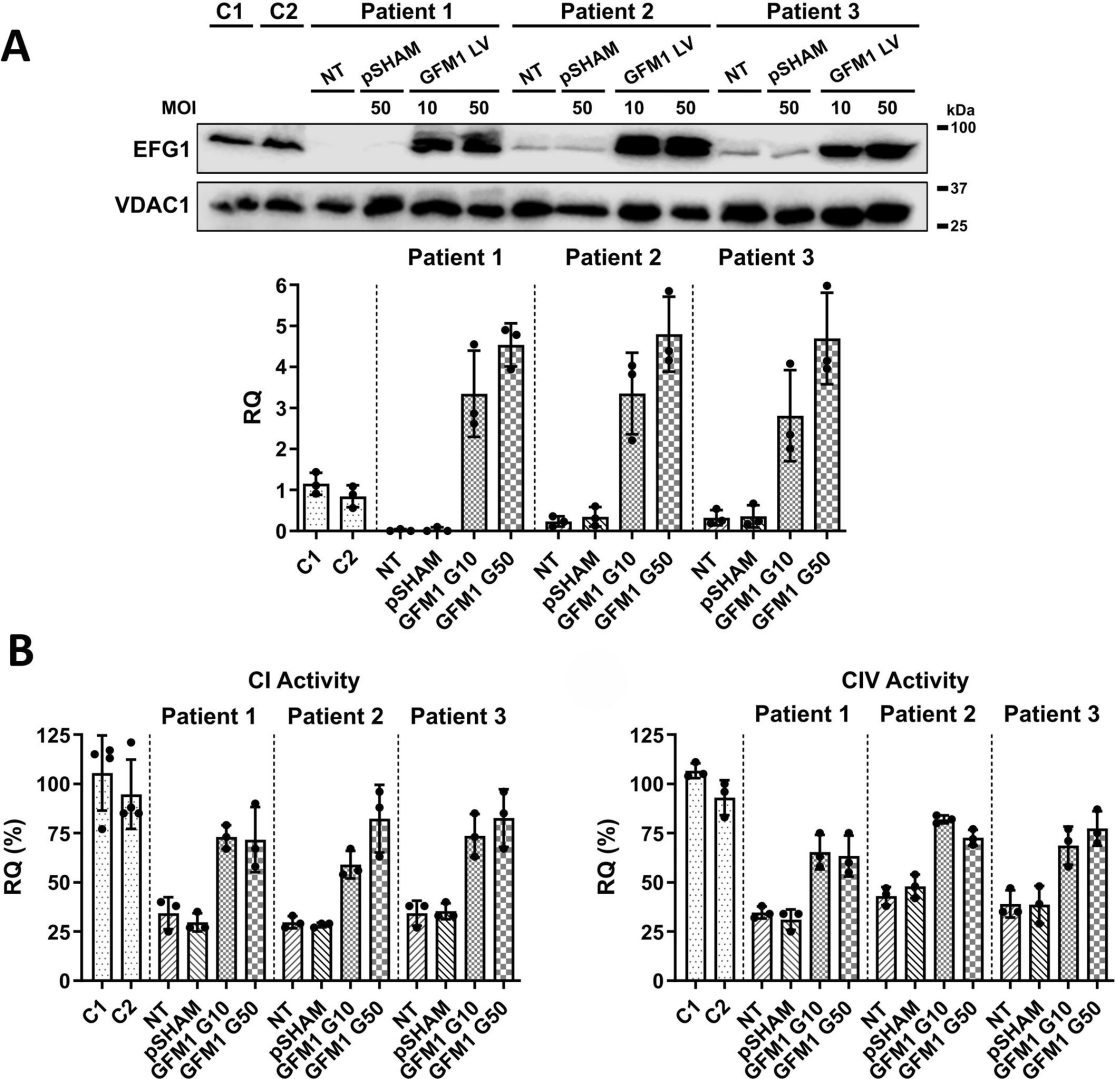
Lentiviral gene therapy rescues EFG1 depletion and corrects OXPHOS deficiency in patients’ fibroblasts. (**A**) Western blot analysis of EFG1 protein levels after lentiviral transduction. EFG1 protein relative amount in the three patients’ fibroblasts after transduction with the pSham LV at a MOI 50 or with the p305-GFM1LV at a MOI 10 (G10) or MOI 50 (G50), in comparison with their corresponding non-transduced cell lines (NT) or two healthy controls (C1 and C2). Results are expressed as the mean ± SD of three experiments carried with the same homogenate. All the values are normalized to VDAC1 and referred to the control protein levels mean (C1 and C2). (**B**) Enzyme activity determination of RC complexes CI and CIV. Relative activities in fibroblasts from C1 and C2 and in fibroblasts from P1, P2, and P3 non-treated (NT), transduced with pSham LV MOI 50 (pSham), or transduced with p305-GFM1LV at a MOI 10 (G10) or 50 (G50). Relative activity is defined as CI activity normalized to CS activity and referred to controls CI/CS activity mean. Bars represent the mean ± SD. RQ relative quantification. Three experimental replicates are depicted for each sample/condition, except for CI enzyme activity determination in C1 and C2, in which four experimental replicates are depicted. Source data are available online for this figure.

We first conducted in vitro experiments by transducing cultured skin fibroblasts from three COXPD1 patients with a lentiviral vector carrying the *GFM1* gene under the control of the human phosphoglycerate kinase (hPGK) promoter (p305-GFM1LV) or a sham vector (pSham LV) without the mentioned therapeutic gene (Appendix Fig. S1). One month after this treatment, both non-transduced and pSham LV-transduced (MOI 50) fibroblasts from patients showed negligible EFG1 protein levels, as compared with those observed in cells from healthy controls. Transduction with p305-GFM1LV resulted in EFG1 expression, reaching levels threefold to fivefold above the levels observed in cells from healthy controls. EFG1 levels were not proportional to the MOI, although a trend to higher values was observed in cells treated with the high MOI (Fig. EV2A). In addition, the combined respiratory chain (RC) deficiency observed in non-transduced and pSham LV-transduced patients’ fibroblasts (∼70% reduction of complex I (CI) activity, and between 50 and 70% reduction of CIV activity) was substantially corrected after p305-GFM1LV treatment (Fig. EV2B). These in vitro results replicated and expanded the previous observed complementation results reported by others (Coenen et al, 2004; Smits et al, 2011) and further supported the notion that *GFM1* gene transfer is a plausible strategy to correct the molecular defects of COXPD1.

### The treatment with the rAAV vectors does not alter the mouse body weight and welfare

The experimental design of the in vivo AAV-based gene therapy study is summarized in Appendix Fig. S2. For both the liver-targeted (AAV9-hAAT-GFM1) and the brain-targeted (AAV9P31-hSyn-GFM1) vectors, a single 5 × 10^12^ vg/kg intravenous dose was administered to 6-week-old *Gfm1*^*R671C/−*^ mice (KI/KO AAV group). The body weight and general behavior of the animals were monitored over the following 4 weeks; at this point (10 weeks of age), the animals were sacrificed for tissue collection to conduct molecular and biochemical analyses.

**Figure EV3:**
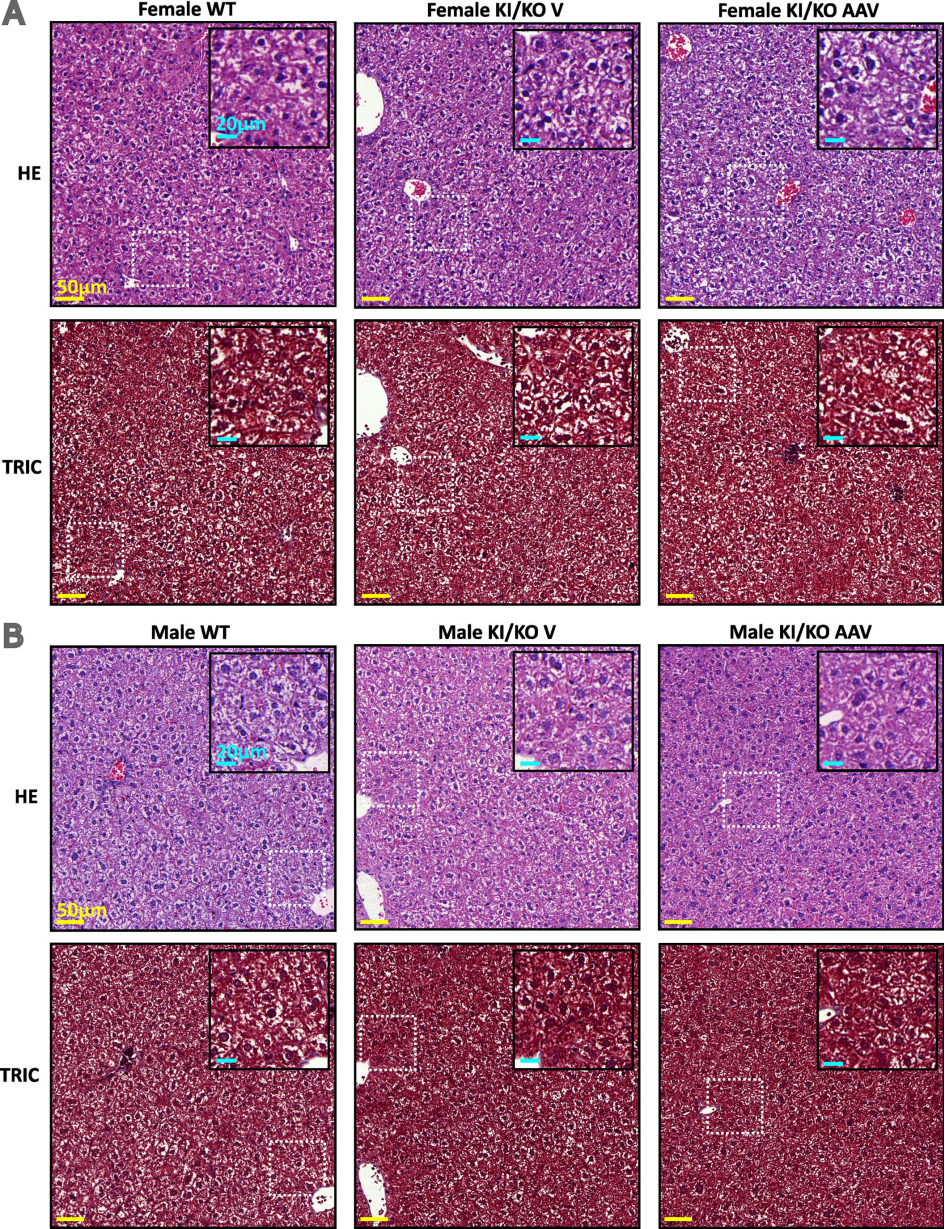
Mouse liver histology of the AAV9-hAAT-GFM1 study. Representative images from formalin-fixed and paraffin-embedded livers of 10-week-old female (**A**) and male (**B**) mice subjected to hematoxylin–eosin staining (HE) and Masson trichrome staining (TRIC). Zoom-out bar: 50 µm. Zoom-in bar: 20 µm. WT wild-type mice, KI/KO V *Gfm1*^*R671C/−*^ mice treated with vehicle, KI/KO AAV *Gfm1*^*R671C/−*^ mice treated with the therapeutic vector. Some selected areas (dotted white boxes) are magnified at the upper right corners of every panel. Source data are available online for this figure.

The treatment with neither vector affected the body weight progression or behavior of the *Gfm1*^*R671C/−*^ mice, compared with age and sex-matched wild-type mice (WT) and the group of *Gfm1*^*R671C/−*^ mice treated with vehicle (KI/KO V) (Appendix Fig. S3). At the end of the monitoring period, the target tissues (liver and brain) had similar weights and appearance among the compared groups. The necropsy examination did not reveal any anatomic alteration associated with rAAV treatments. In addition, hematoxylin–eosin (HE) and Masson trichrome staining in livers from mice treated with AAV9-hAAT-GFM1 showed similar morphological features compared to those observed in livers from control groups (WT and KI/KO V), with no signs of fibrosis or carcinogenic processes (Figs. EV3A, females and EV3B, males).

### AAV9-hAAT-GFM1 transduces *Gfm1*^*R671C/−*^ liver and partially rescues EFG1 depletion

**Figure 1:**
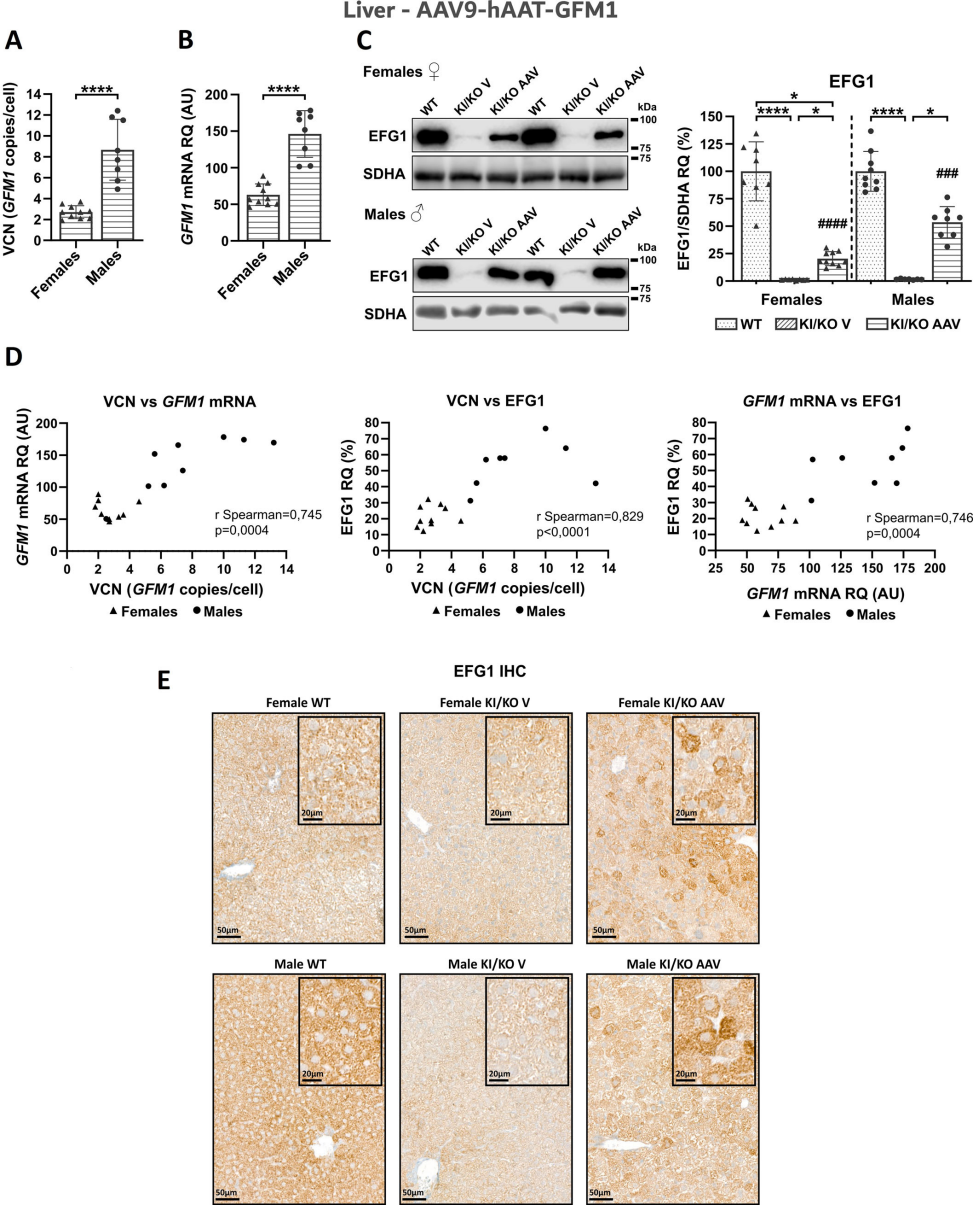
AAV9-hAAT-GFM1 transduction and hGFM1 expression in *Gfm1*^*R671C/−*^ liver. (**A**) Liver transduction of rAAV-treated *Gfm1*^*R671C/−*^ mice analyzed by RT-qPCR using a specific human *GFM1* cDNA probe (VCN: vector copy number). Asterisks indicate statistical differences between females (*n* = 10) and males (*n* = 8) (****P < 0.0001, Mann–Whitney *U* test). (**B**) *hGFM1* mRNA levels determined by RT-qPCR using a specific human *GFM1* cDNA probe in livers from *Gfm1*^*R671C/−*^ rAAV-treated mice. Values were normalized to *Ppia* mRNA levels. Null amplification was observed in samples obtained from vehicle-treated mice. Asterisks indicate statistical differences between females (*N* = 10) and males (*N* = 8) (****P < 0.0001, Mann–Whitney *U* test). (**C**) EFG1 levels in liver mitochondria. SDHA was used as a mitochondrial loading control. The SDHA blots of this panel were reused in Fig. 3A (bottom SDHA strips). Single mouse values (triangles and dots) are expressed as a percentage of the wild-type mean, and bars represent the mean relative quantity ( ± SD). Statistical analyses were independently performed for females (WT *n* = 8; KI/KO V *n* = 8; KI/KO AAV *n* = 10) and males (WT *n* = 9; KI/KO V *n* = 7; KI/KO AAV *n* = 8) (*P < 0.05, ****P < 0.0001, Kruskal–Wallis and Dunn’s multiple comparisons test). Hash marks indicate statistical differences between KI/KO V and KI/KO AAV mice (^###^*P* < 0.001, ^####^*P* < 0.0001, Mann–Whitney *U* test). (**D**) Correlations between VCN, *GFM1* mRNA, and EFG1 levels in the liver of rAAV-treated *Gfm1*^*R671C/−*^ mice (females *n* = 10; males *n* = 8 for all correlations). (**E**) EFG1 IHC stains of formalin-fixed and paraffin-embedded murine livers. Representative images from male and female mice are shown. EFG1-positive cells stain brown. (Zoom-out bar: 50 µm. Zoom-in bar: 20 µm). WT wild-type mice, KI/KO V *Gfm1*^*R671C/−*^ mice treated with vehicle, KI/KO AAV *Gfm1*^*R671C/−*^ mice treated with the therapeutic vector, RQ relative quantification. Source data are available online for this figure.

Since sex influences rAAV liver transduction in mice, with greater transduction efficiency in males than females (Davidoff et al, 2003), we conducted parallel experiments in male and female mice to test the efficacy of the liver-targeted vector. At the tested dose of 5 ×10^12^ vg/kg, AAV9-hAAT-GFM1 successfully transduced *Gfm1*^*R671C/−*^ mouse liver. Vector genomes (vg) were determined by qPCR targeting the human *GFM1* cDNA. Vector copy numbers (VCN) were notably higher in males (8.7 ± 2.9 vg/cell, mean ± SD), representing an approximately threefold increase compared to females (2.7 ± 0.6 vg/cell) (Fig. 1A). Null amplification was always observed for DNA samples from WT mice and KI/KO mice treated with vehicle.

**Figure EV4:**
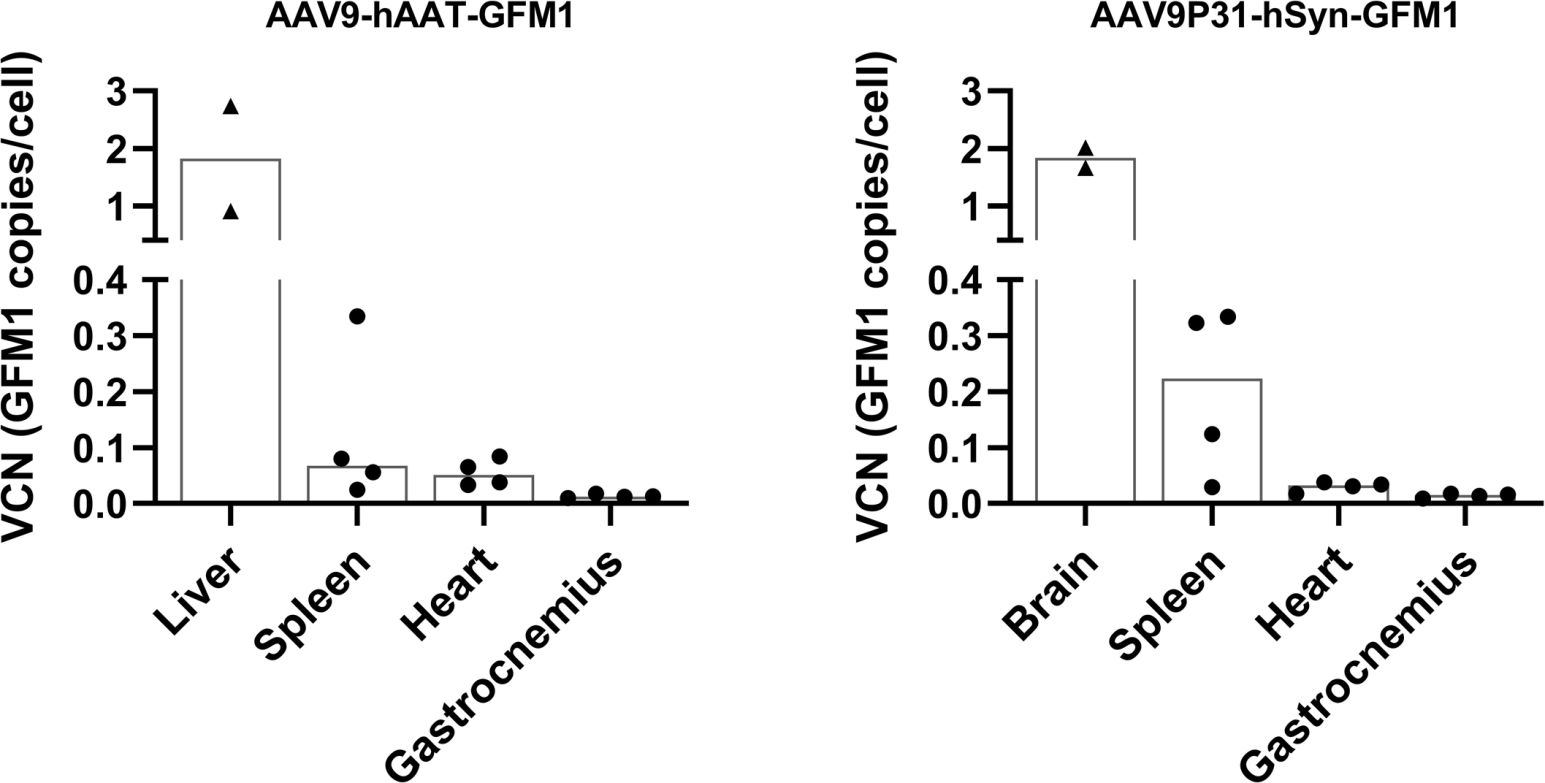
Biodistribution of AAV9-hAAT-GFM1 and AAV9P31-hSyn-GFM1 in non-target tissues. Transduction levels observed in *Gfm1*^*R671C/−*^ mice treated with AAV9-hAAT-GFM1 (left) and AAV9P31-hSyn-GFM1 (right) in three non-target tissues (*n* = 4 animals for each treatment), as compared with the levels observed in the corresponding target tissues for each vector (liver *n* = 2, and brain *n* = 2, respectively) in two animals. Vector copy number (VCN) was analyzed by RT-qPCR using a specific human *GFM1* cDNA probe. Source data are available online for this figure.

In order to evaluate the transduction levels achieved in the brain when using the liver-targeted vector, the VCN was also analyzed in this tissue, although therapeutic gene expression was not expected in this case. Brain transduction was barely detectable (∼0.003 vg/cell) in both males and females one month after the administration of the vector at the aforementioned dose. VCN was also analyzed in three non-target tissues: skeletal muscle (gastrocnemius), heart, and spleen. In all cases, the transduction was detectable but low (below 0.1 vg/cell, except for one spleen sample with 0.3 vg/cell), as compared with values observed in target tissues (Fig. EV4, left).

The human *GFM1* transgene was efficiently expressed in livers from *Gfm1*^*R671C/−*^-treated mice, and *GFM1* mRNA levels were approximately threefold higher in males than in females, as assessed by RT-qPCR (Fig. 1B), correlating with the difference observed in transduction levels. No amplification was detected in samples from WT mice and vehicle-treated KI/KO mice. Western blot immunodetection was performed on liver mitochondrial extracts in order to quantify the EFG1 levels. Since the anti-EFG1 primary antibody used (ab173529, Abcam) detects both the murine and human EFG1 forms, and they have a similar molecular weight, we assume that the increased EFG1 signal observed in mitochondrial extracts of KI/KO AAV mice compared with the levels observed in the KI/KO V was attributable to the expression of the therapeutic transgene. Treatment of *Gfm1*^*R671C/−*^ with AAV9-hAAT-GFM1 resulted in significant increases of mitochondrial EFG1 levels in females (20.4 ± 6.4% vs 1.2 ± 0.4% in the vehicle cohort, mean ± SD expressed as percentages of the mean level of the WT mice) and in males (53.6 ± 14.3% vs 1.6 ± 0.5% in the vehicle cohort) (Fig. 1C). This result not only demonstrated that the therapeutic gene was being expressed, but also that the human EFG1 protein is imported into murine liver mitochondria. Furthermore, *GFM1* mRNA and mitochondrial EFG1 levels positively correlated with AAV9-hAAT-GFM1VCN per liver cell, and mitochondrial EFG1 with *GFM1* mRNA levels (Fig. 1D).

EFG1 immunohistochemical analyses performed on paraffin-embedded livers revealed a differential staining between WT, KI/KO V, and KI/KO AAV mice. A clear positive staining of a considerable number of hepatocytes was observed throughout all the tissue slides in liver samples from AAV9-hAAT-GFM1-treated mice, showing a mosaic-like pattern with higher intensities in centrilobular regions (Fig. 1E).

### AAV9P31-hSyn-GFM1 transduces *Gfm1*^*R671C/−*^ brain and partially rescues EFG1 depletion

**Figure 2:**
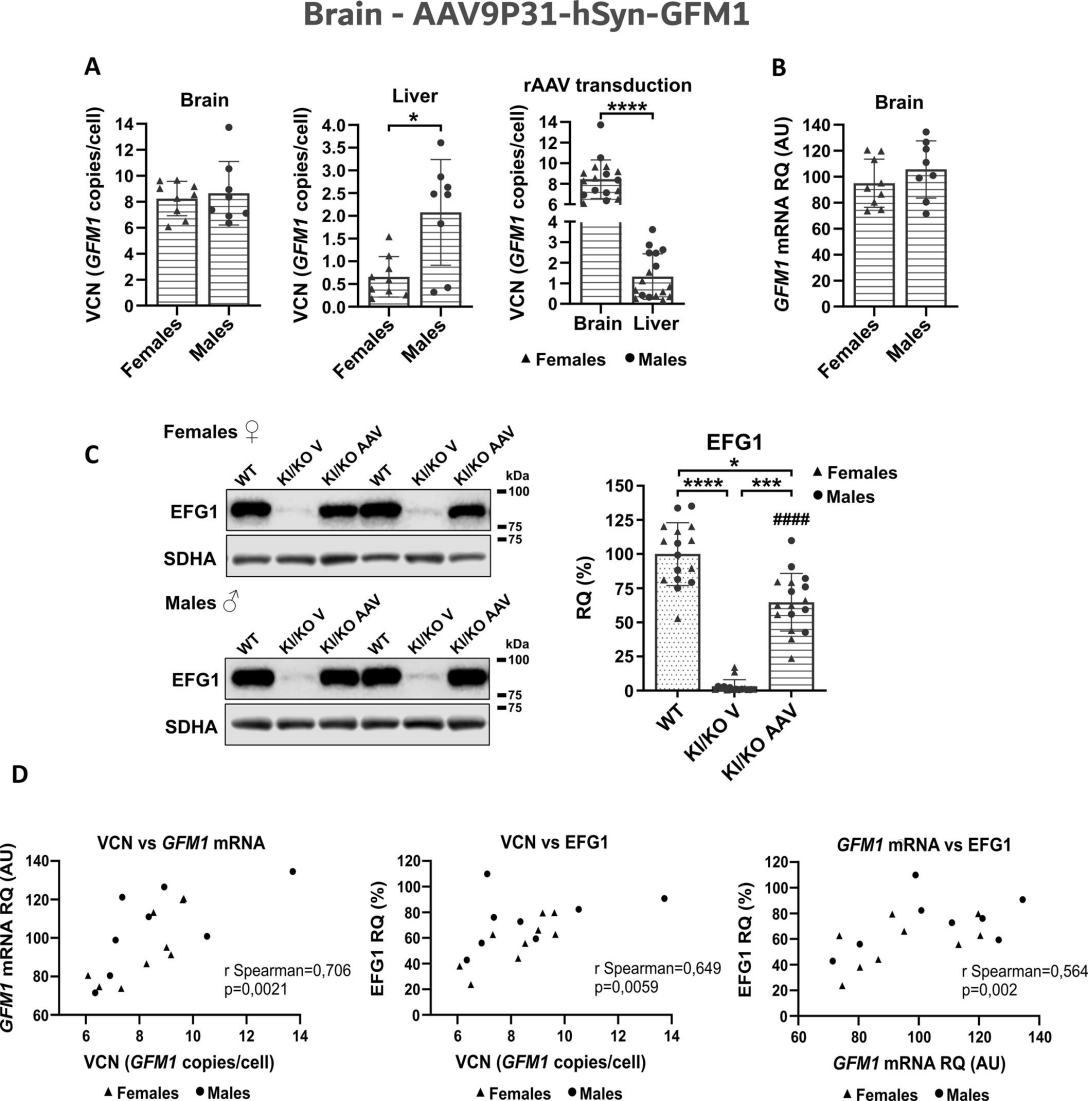
AAV9P31-hSyn-GFM1 transduction and *hGFM1* expression in *Gfm1*^*R671C/−*^ brain. (**A**) AAV9P31-hSyn-GFM1-mediated brain and liver transduction was analyzed in *Gfm1*^*R671C/−*^-treated mice by RT-qPCR using a specific human *GFM1* cDNA probe (VCN: Vector copy number). Brain and liver VCN, females *n* = 9, males *n* = 8; rAAV transduction, brain *n* = 17 and liver *n* = 17. Asterisks indicate statistical differences between groups (*P < 0.05, ****P < 0.0001, Mann–Whitney *U* test). (**B**) *hGFM1* mRNA levels determined by RT-qPCR using a specific *hGFM1* cDNA probe and normalized with *Ppia* mRNA levels in brains from *Gfm1*^*R671C/−*^ rAAV-treated mice. Females *n* = 9, males *n* = 8. Null amplification was observed in samples obtained from vehicle-treated mice. (**C**) EFG1 levels in brain mitochondria. SDHA was used as a mitochondrial loading control. The SDHA blots of this panel are reused in Fig. 3B (top SDHA strips). Single mouse values (dots and triangles) are expressed as a percentage of the wild-type mean, and bars represent the mean ± SD relative quantity. WT and KI/KO V, females *n* = 8, males *n* = 8; KI/KO AAV, females *n* = 9, males *n* = 8. Asterisks indicate statistical differences between groups (*P < 0.05, ****P < 0.0001, Kruskal–Wallis and Dunn’s multiple comparisons test). Hash marks indicate statistical differences between KI/KO V and KI/KO AAV mice (^####^*P* < 0.001, Mann–Whitney *U* test comparing KI/KO V and KI/KO AAV mice). (**D**) Correlations between brain VCN, *GFM1* mRNA, and EFG1 levels in brains of *Gfm1*^*R671C/−*^ rAAV-treated mice. WT wild-type mice, KI/KO V *Gfm1*^*R671C/−*^ mice treated with vehicle, KI/KO AAV *Gfm1*^*R671C/−*^ mice treated with the therapeutic vector, RQ relative quantification (females *n* = 9; males *n* = 8 for all correlations). Source data are available online for this figure.

After intravenous administration at a dose of 5 × 10^12^ vg/kg, the rAAV vector targeted to the central nervous system (CNS) effectively transduced the brain in *Gfm1*^*R671C/−*^ mice, with similar efficiencies in females (8.2 ± 1.3 vg/cell, mean ± SD) and males (8.7 ± 2.4 vg/cell). The tropism of this new AAV9-engineered capsid (AAV9P31) was lower for the liver in comparison to the parental serotype AAV9. Again, the transduction efficiency was higher in males (2.1 ± 1.2 vg/cell) than in females (0.7 ± 0.4 vg/cell) (Fig. 2A). Non-target tissues were also investigated: skeletal muscle (gastrocnemius), heart, and spleen, with detectable but low VCN values (Fig. EV4, right).

The *GFM1* transgene transcripts were detected in brains from rAAV-treated *Gfm1*^*R671C/−*^ mice, reaching similar levels in females and males (Fig. 2B). Again, RT-qPCR always showed null amplification for DNA and cDNA samples from untreated or vehicle-treated mice. The treatment with the CNS-targeted rAAV vector rescued the EFG1 protein levels in *Gfm1*^*R671C/−*^ mice (64.8 ± 21.2%, referred to the mean of the WT group) as compared with the levels of murine EFG1 observed in the group treated with vehicle (3.1 ± 4.9%) (Fig. 2C). As observed in livers from mice treated with the liver-targeted vector, the levels of mRNA and mitochondrial EFG1 correlated with the CNS-targeted vector genomes per cell in the brain, and mitochondrial EFG1 with *GFM1* mRNA levels (Fig. 2D).

### Liver-targeted gene therapy corrects the hepatic COXPD molecular phenotype

**Figure 3:**
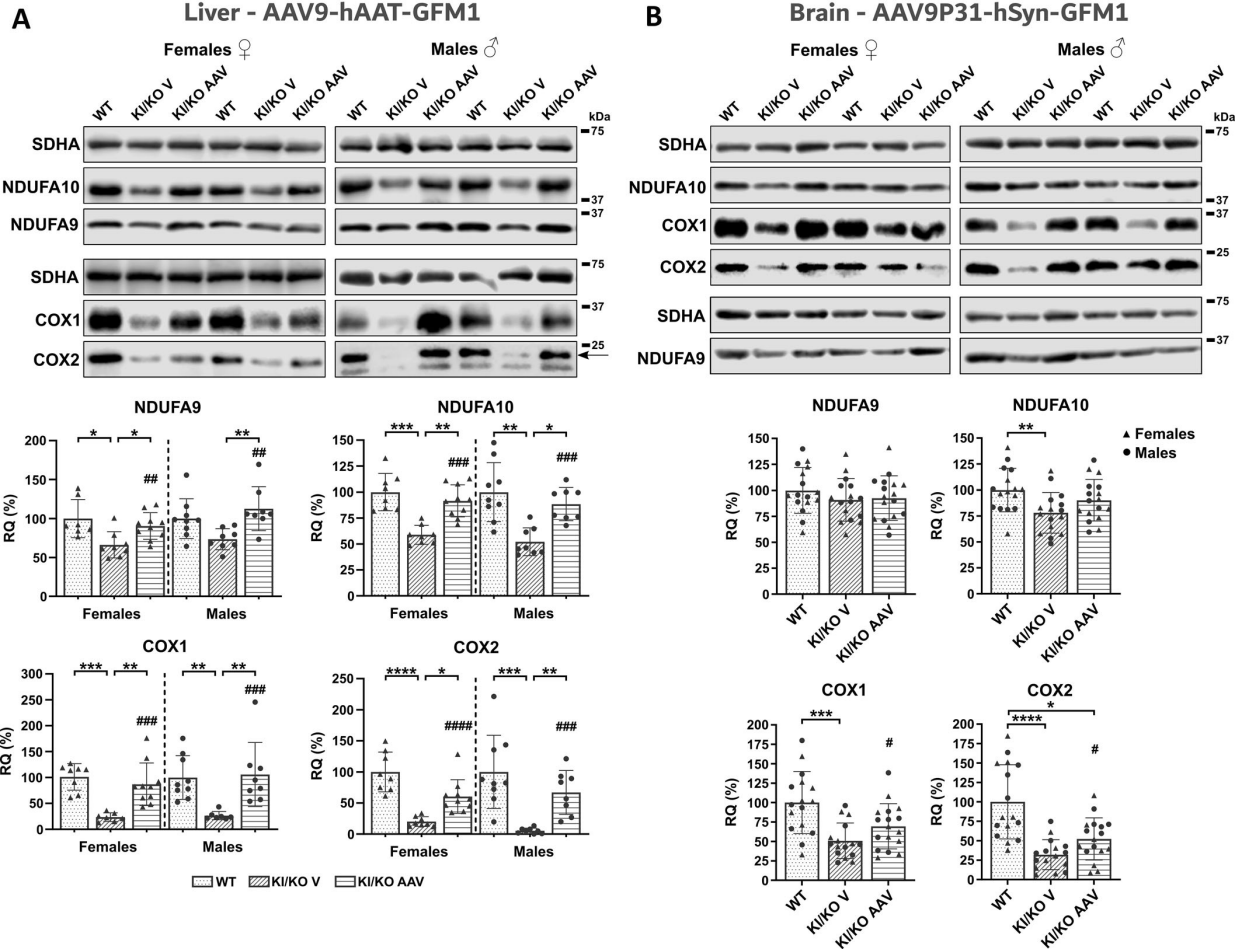
Levels of complex I and IV subunits in liver and brain mitochondria from rAAV-treated *Gfm1*^*R671C/−*^ mice. SDS-PAGE and western blot immunodetection of CI subunits NDUFA9 and NDUFA10, and CIV subunits COX1 and COX2 in (**A**) liver mitochondria from mice treated with AAV9-hAAT-GFM1 and (**B**) brain mitochondria from mice treated with AAV9P31-hSyn-GFM1. SDHA was used as a mitochondrial loading control. The bottom SDHA blot strips of panel A were reused from those shown in Fig. 1C, and the top SDHA blot strips of panel B were reused from those shown in Fig. 2C. For COX2, the arrow points to the specific band. Separate statistical analyses were performed for males and females for the liver-targeted approach. Single mouse values, represented as dots and triangles, are expressed as a percentage of the wild-type mean, and bars represent the mean relative quantity ( ± SD). The exact *n* for each experiment is indicated in Appendix Table S1. Asterisks indicate statistical differences between groups (*P < 0.05, **P < 0.01, ***P < 0.001, ****P < 0.0001, Kruskal–Wallis and Dunn’s multiple comparisons test). Hash marks indicate statistical differences between KI/KO V and KI/KO AAV mice (^#^*P* < 0.05, ^##^*P* < 0.01, ^###^*P* < 0.001, ^####^*P* < 0.001, Mann–Whitney *U* test). RQ relative quantification. Source data are available online for this figure.

**Figure EV5:**
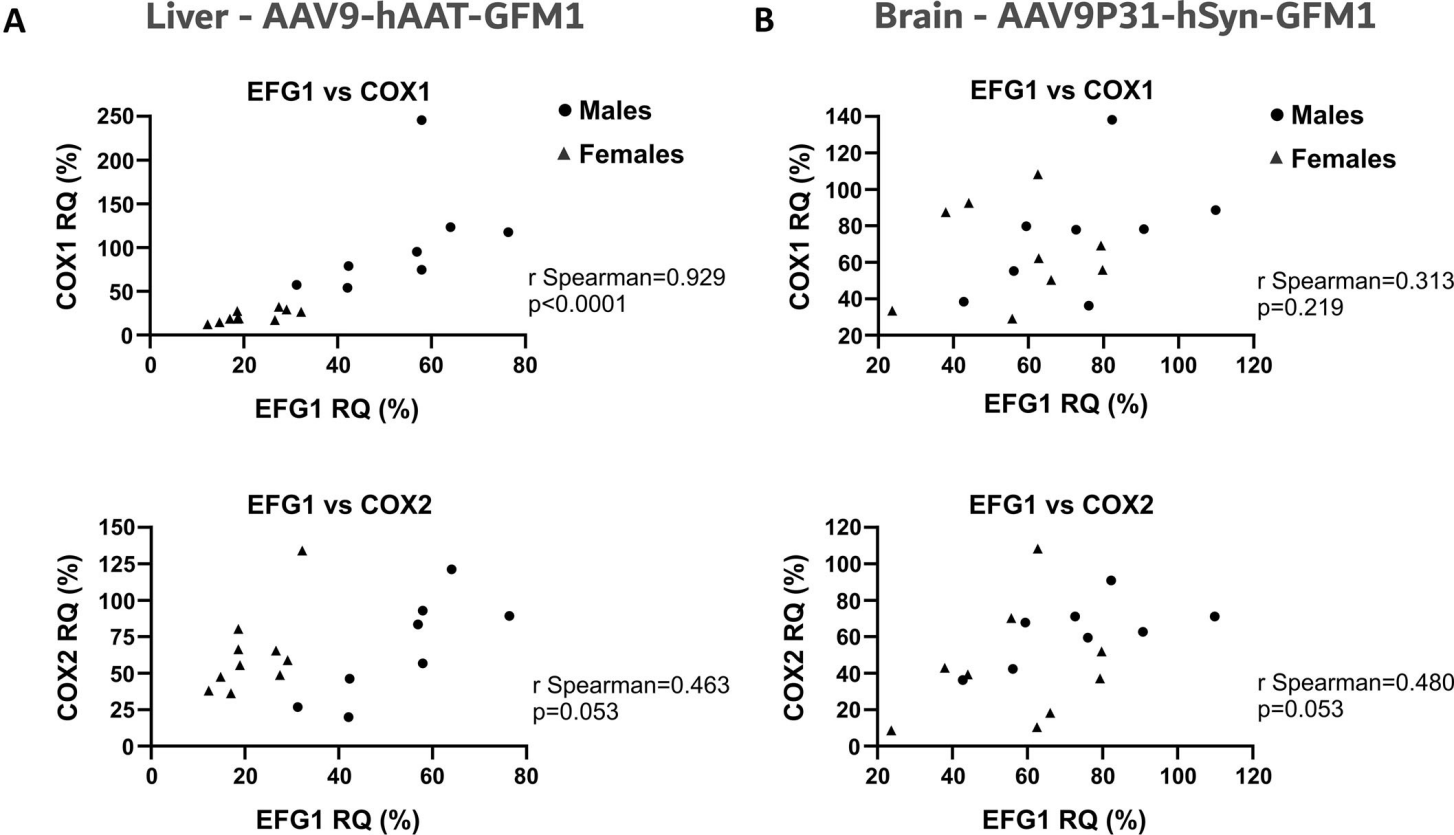
Correlations between mitochondrial levels of EFG1 and complex CIV subunits. Correlation comparing mitochondrial levels of EFG1 and mtDNA encoded CIV subunits (COX1 and COX2) in *Gfm1*^*R671C/−*^ mice samples from (**A**) livers treated with AAV9-hAAT-GFM1 (females *n* = 10, males *n* = 8) and (**B**) brains treated with AAV9P31-hSyn-GFM1 (females *n* = 9 and males *n *= 8). Analysis was performed considering EFG1, COX1, and COX2 protein levels (RQ, % of WT mean values) in mitochondria from target tissues of KI/KO AAV females (triangles) and males (circles). The Spearman statistical test was applied to each comparison. RQ relative quantification. Source data are available online for this figure.

We analyzed through western blot the levels of two mtDNA encoded CIV subunits (COX1 and COX2) in liver mitochondria (Fig. 3A). The results revealed that AAV9-hAAT-GFM1 treatment restored the levels of both subunits in *Gfm1*^*R671C/−*^ mice, as compared with those found in the vehicle-treated group (results expressed as percentages of levels found in WT mice, mean ± SD): for COX1, 86.9 ± 41.1% vs 23.5 ± 8.4% in females and 105.9 ± 61.9% vs 26.3 ± 7.9% in males; and for COX2, 60.4 ± 27.1% vs 20.0 ± 7.9% in females and 67.0 ± 35.4% vs 5.2 ± 4.4% in males. Furthermore, a significant positive correlation was found between EFG1 and COX1 levels (and nearly significant with COX2 levels) in liver mitochondria in the KI/KO mice treated with the therapeutic vector (Fig. EV5A). All these results indicate that the human EFG1 protein is functional in *Gfm1*^*R671C/−*^ mice, since it restores mtDNA-encoded polypeptide synthesis.

In addition, the analysis of two nuclear DNA (nDNA) encoded CI subunits (NDUFA9 and NDUFA10) also showed partial restoration (Fig. 3A): for NDUFA9, 90.5 ± 17.1% in rAAV-treated vs 66.3 ± 16.9% in vehicle-treated KI/KO females, and 112.7 ± 28.2% in rAAV-treated vs 73.5 ± 13.6% in vehicle-treated KI/KO males; for NDUFA10, 91.6 ± 15.3% in rAAV-treated vs 58.9 ± 9.1% in vehicle-treated KI/KO females and 88.4 ± 15.8% in rAAV-treated vs 52.2 ± 13.4% in vehicle-treated KI/KO males. These results reflect that nDNA-encoded OXPHOS subunits are stabilized and not degraded when they are assembled in the complexes with the newly synthesized mtDNA-encoded subunits.

**Figure 4:**
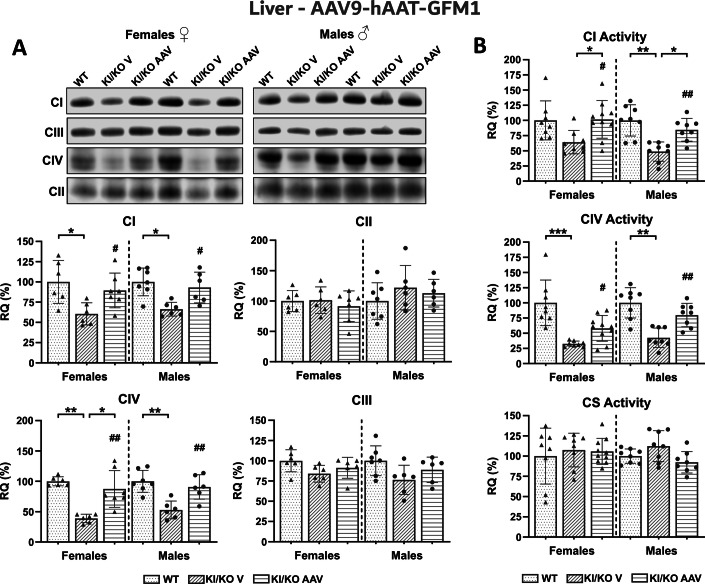
AAV9-hAAT-GFM1 effect on *Gfm1*^*R671C/−*^ liver combined OXPHOS defect. (**A**) BN-PAGE and western blot immunodetection of respiratory chain complexes (CI, CII, CIII, CIV) from liver mitochondria using specific antibodies. Relative quantities were determined by densitometry of each band, normalized by the complex CII amount, and referred to a calibrator sample loaded in all westerns. (**B**) Relative enzyme activities of complexes I and IV, and citrate synthase (CS) in liver homogenates. CI and CIV enzyme activities were normalized by CS activity that was used as a mitochondrial content control. Single mouse values, represented as dots and triangles, are expressed as a percentage of the wild-type mean, and bars represent the mean relative quantity ( ± SD). The exact n for each experiment is indicated in Appendix Table S1. Asterisks indicate statistical differences between groups (*P < 0.05, **P < 0.01, ***P < 0.001, Kruskal–Wallis and Dunn’s multiple comparisons test). Hash marks indicate statistical differences between KI/KO V and KI/KO AAV mice (^#^*P* < 0.05, ^##^*P* < 0.01, Mann–Whitney *U* test). RQ relative quantification. Source data are available online for this figure.

BN-PAGE (Blue Native PolyAcrylamide Gel Electrophoresis) western blot for the analysis of fully assembled OXPHOS complexes showed that the levels of CI and CIV in the liver were increased in KI/KO mice treated with AAV9-hAAT-GFM1 as compared with those found in the vehicle-treated group, reaching values close to those of WT mice. For CI, 89.5 ± 21.4% in AAV-treated vs 60.5 ± 13.7% in vehicle-treated females and 93.1 ± 19.1% in AAV-treated vs 66.3 ± 8.5% in vehicle-treated males; for CIV, 87.4 ± 30.2% in AAV-treated vs 38.9 ± 6.9% in vehicle-treated females and 90.8 ± 20.0% in AAV-treated vs 52.8 ± 14.7% in vehicle-treated males (Fig. 4A). Taken together, these results show that AAV9-hAAT-GFM1 treatment increases the levels of mitochondrial OXPHOS subunits, enabling the restoration of assembled complexes I and IV levels.

Finally, the enzyme activities of the RC complexes I and IV (and the Krebs cycle enzyme citrate synthase (CS), as a marker of mitochondrial mass) were quantified in liver homogenates. The treatment with AAV9-hAAT-GFM1 restored almost completely the enzymatic activity of these complexes in KI/KO mice, as compared with the low activities observed in the vehicle-treated group. For CI, the activity reaches 101.7 ± 30.9% in rAAV-treated vs 64.1 ± 19.2% in vehicle-treated female mice and 84.5 ± 18.7% in rAAV-treated vs 48.7 ± 15.9% in vehicle-treated male mice. For CIV (which is more affected than CI in the animal model), the activity reaches 58.0 ± 21.2% in rAAV-treated vs 32.5 ± 4.6% in vehicle-treated female mice and 79.8 ± 19.3% in rAAV-treated vs 42.5 ± 16.0% in vehicle-treated male mice (Fig. 4B). No significant differences among groups were detected for CS activity. Overall, these results demonstrate that liver-targeted gene therapy expressing human *GFM1* restores the mtDNA-encoded polypeptide synthesis and corrects the hepatic OXPHOS dysfunction in COXPD1 mice.

### CNS-targeted gene therapy partially corrects the brain COXPD molecular phenotype

In order to test the effect of the CNS-targeted vector AAV9P31-hSyn-GFM1 on the COXPD molecular phenotype in the brain of *Gfm1*^*R671C/−*^ mice, we replicated in this tissue the analyses of endpoints previously studied in the liver-targeted approach. No differences in brain transduction efficiency or in the downstream related effects were observed between male and female mice treated with the CNS-targeted rAAV vector. Therefore, data from both sexes were pooled for the analyses of the CNS-targeted study.

The levels of the nDNA-encoded CI subunit NDUFA9 were not affected in the KI/KO mice, while those of NDUFA10 were slightly but significantly reduced (to 78.2 ± 19.6%, referred to the mean of WT levels). A trend to recover WT levels was observed in mice treated with the CNS-targeted vector, but the difference did not reach statistical significance. The levels of the CIV subunits COX1 and COX2 were partially restored four weeks after AAV9P31-hSyn-GFM1 treatment, in comparison with the clear reduction detected in mitochondrial pellets from KI/KO mice treated with the vehicle: for COX1, 69.5 ± 29.1% vs 50.8 ± 22.8%; for COX2, 52.3 ± 27.1% vs 32.0 ± 19.3% (Fig. 3B). No correlation was observed between EFG1 and COX1 (Fig. EV5B) in contrast to what was found in the liver-targeted mice.

**Figure 5:**
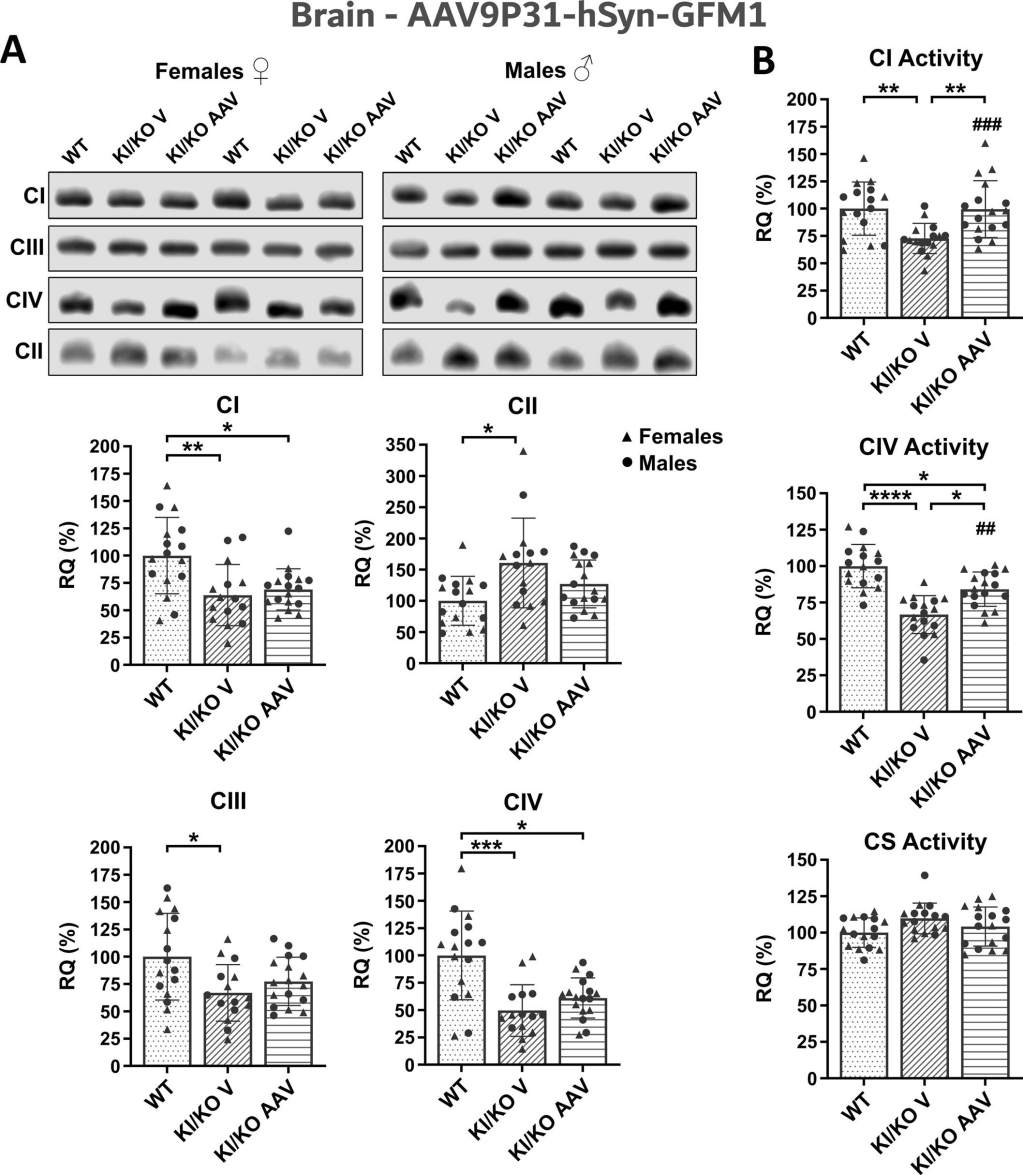
AAV9P31-hSyn-GFM1 effect on *Gfm1*^*R671C/−*^ brain combined OXPHOS defect. (**A**) BN-PAGE and western blot immunodetection of respiratory chain complexes (CI, CII, CIII, CIV) from brain mitochondria using specific antibodies. Relative quantities were determined by densitometry of each band, normalized by the complex CII amount, and referred to a calibrator sample loaded in all westerns. (**B**) Relative enzyme activities of complexes I and IV, and citrate synthase (CS) in brain homogenates. CI and CIV enzyme activities were normalized by CS activity that was used as a mitochondrial content control. Single mouse values, represented as dots and triangles, are expressed as a percentage of the wild-type mean, and bars represent the mean relative quantity ( ± SD). The exact *n* for each experiment is indicated in Appendix Table S1. Asterisks indicate statistical differences between groups (*P < 0.05, **P < 0.01, ***P < 0.001, ****P < 0.0001, Kruskal–Wallis and Dunn’s multiple comparisons test). Hash marks indicate statistical differences between KI/KO V and KI/KO AAV mice (^##^*P* < 0.01, ^###^*P* < 0.001, Mann–Whitney *U* test). RQ relative quantification. Source data are available online for this figure.

BN-PAGE western blot analysis showed a slight increase in complex I, III, and IV levels in brain mitochondria from rAAV-treated mice, in comparison with those observed in the vehicle-treated group: for CI, 69.1 ± 18.9% vs 63.8 ± 27.9%; for CIII, 77.4 ± 22.1% vs 66.9 ± 25.9%; for CIV, 60.9 ± 18.4% vs 49.5 ± 23.7%. However, the therapy did not induce statistically significant increases in assembled complexes when comparing their levels to those from the vehicle-treated group. It is worth noting that CII levels (used as mitochondrial loading control) were significantly increased in brain mitochondria from vehicle-treated KI/KO mice (160.6 ± 71.7%), and this increase was attenuated in rAAV-treated mice (127.1 ± 38.3%) (Fig. 5A).

As observed with the liver-targeted vector, the treatment with AAV9P31-hSyn-GFM1 not only rescued the levels of complex subunits that are depleted in the brain of the animal model, but also their enzymatic function. The treatment resulted in a significant recovery of CI and CIV enzyme activities, as compared with the vehicle-treated animals: for CI, 99.5 ± 25.9% vs 72.7 ± 13.8%; for CIV, 84.2 ± 11.8% vs 66.7 ± 13.1%. A slight trend towards a higher CS activity was observed in vehicle-treated KI/KO mice, although not reaching statistical significance (Fig. 5B).

### The molecular responses to EFG1 depletion are partially prevented by gene therapy

**Figure 6:**
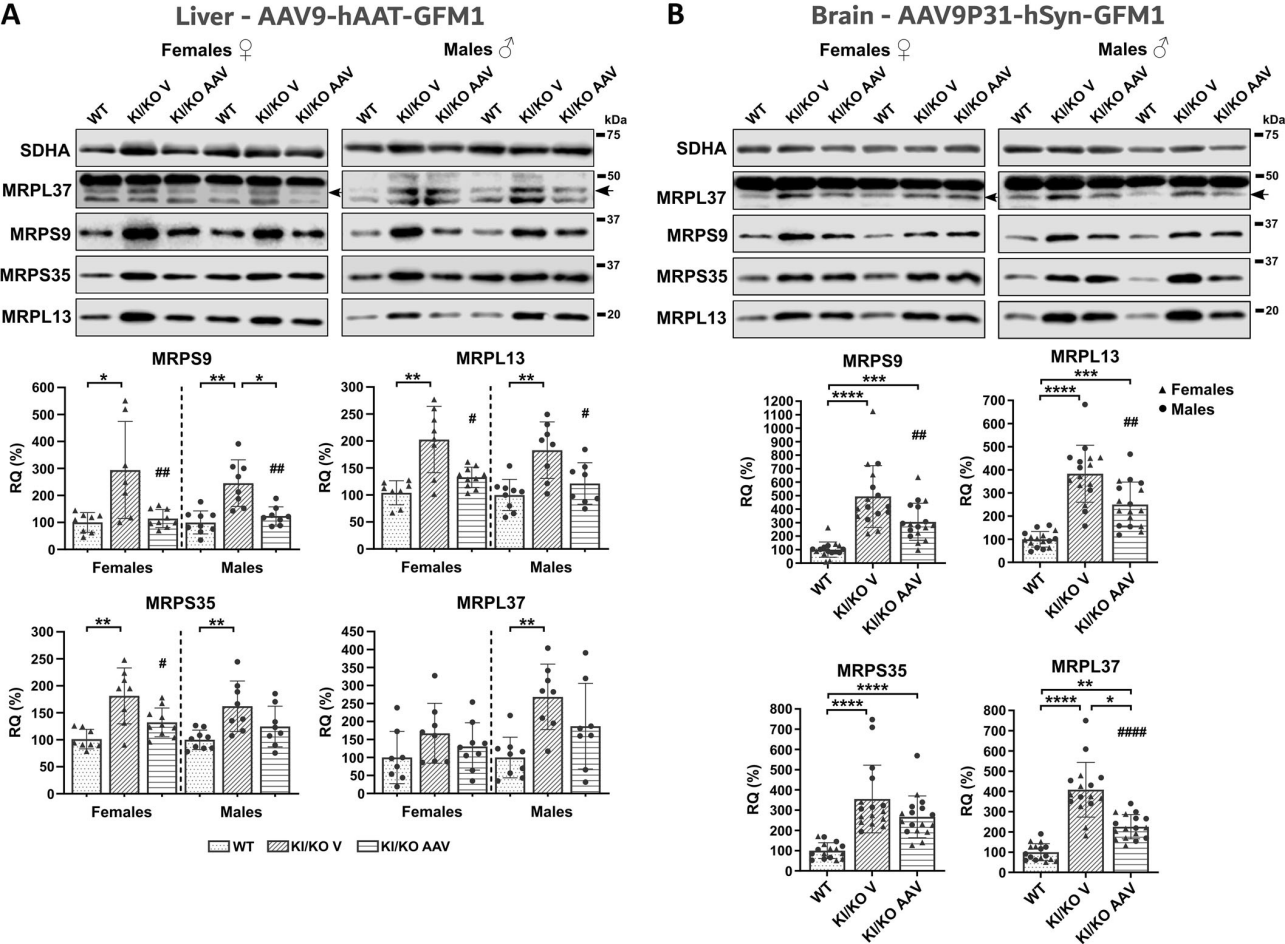
Mitoriboprotein levels in liver and brain mitochondria from rAAV-treated *Gfm1*^*R671C/−*^ mice. SDS-PAGE and western blot immunodetection of MRPS9 and MRPS35 (28S – SSU subunits), MRPL13 and MRPL37 (39S – LSU subunits) in (**A**) liver mitochondria from mice treated with the liver-targeted vector and (**B**) brain mitochondria from mice treated with the CNS-targeted vector. SDHA was used as a loading control. Separated statistical analyses were performed in males and females in the liver-targeted approach, while analysis in the CNS-targeted study was not split by sex. Single mouse values, represented as dots and triangles, are expressed as a percentage of the wild-type mean, and bars represent the mean relative quantity ( ± SD). For MRPL13, the arrow points to the specific band. The exact n for each experiment is indicated in Appendix Table S1. Asterisks indicate statistical differences between groups (*P < 0.05, **P < 0.01, ***P < 0.001, ****P < 0.0001, Kruskal–Wallis and Dunn’s multiple comparisons test). Hash marks indicate statistical differences between KI/KO V and KI/KO AAV mice (^#^*P* < 0.05, ^##^*P* < 0.01, ^####^*P* < 0.001, Mann–Whitney *U* test). RQ relative quantification. Source data are available online for this figure.

We had previously found increased expression of some mitoribosomal proteins (MRPs) in the *Gfm1*^*R671C/R671C*^ mouse model, which could represent a molecular compensatory response to the impaired mitochondrial translation caused by EFG1 depletion (Molina-Berenguer et al, 2022). Here, we have explored if a similar finding is observed in *Gfm1*^*R671C/−*^ mice, and what is the effect of AAV-gene therapy on these and other molecular responses in the liver and brain. We found that *Gfm1*^*R671C/−*^ mice present a substantial increase of MRPs of the 28S subunit (SSU) (MRPS9 and MRPS35) and of the 39S subunit (LSU) (MRPL13 and MRPL37) in the liver and brain mitochondria. In the liver, the AAV9-hAAT-GFM1 treatment resulted in a reduction of MRPs in KI/KO mice in both sexes in comparison to vehicle-treated mice (Fig. 6A; Table 1). Similarly, the substantial increase of MRPs in brain mitochondria from KI/KO mice was significantly reduced in mice treated with the AAV9P31-hSyn-GFM1 vector (Fig. 6B; Table 1).

**Table 1:** Mitoribosomal proteins and mtRNA levels from *Gfm1*^*R671C/−*^ mice included in the gene therapy studies.

AAV9-AAT-GFM1—liver (mean (SD))				
	Female KI/KO V	Female KI/KO AAV	Male KI/KO V	Male KI/KO AAV
MRPS9	294.3% (180.2)	114.6% (33.3)	245.5% (86.3)	124.0% (33.9)
MRPS35	181.4% (51.9)	132.2% (26.6)	162.2 % (46.8)	124.6% (37.7)
MRPL13	202.8% (61.4)	132.8% (18.9)	183.1% (52.3)	121.3% (38.4)
MRPL37	167.3% (83.3)	130.6% (65.8)	268.6% (90.9)	187.2% (119.2)
*Nd4 mRNA*	148.9% (22.6)	141.7% (29.9)	138.6% (26.3)	140.5% (33.9)
*Cox1 mRNA*	134.7% (18.5)	129.2% (20.3)	126.0% (17.9)	120.7% (20.9)
*12S rRNA*	161.6% (12.4)	131.0% (9.4)	164.5% (23.7)	148.7% (65.8)
*16S rRNA*	159.6% (17.5)	137.1% (20.1)	162.3% (27.6)	140.8% (42.9)
**AAV9P31-hSyn-GFM1—brain (mean (SD))**				
	**KI/KO V**		**KI/KO AAV**	
MRPS9	493.9% (229.2)		305.8% (137.3)	
MRPS35	355.4% (167.1)		267.1% (103.1)	
MRPL13	383.2% (123.2)		250.2% (96.9)	
MRPL37	408.3% (135.2)		225.9% (59.6)	
*Nd4 mRNA*	164.7% (16.7)		148.2% (14.3)	
*Cox1 mRNA*	148.9% (14.9)		137.7% (13.4)	
*12S rRNA*	184.1% (24.0)		157.9% (16.8)	
*16S rRNA*	184.6% (16.2)		155.9% (12.5)	

**Figure 7:**
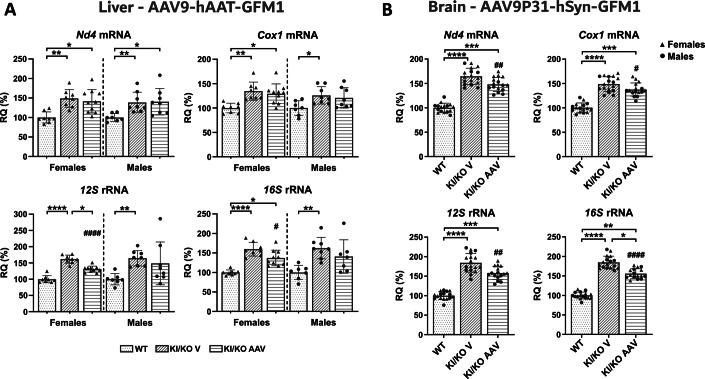
Mitochondrial RNA levels in liver and brain from rAAV-treated *Gfm1*^*R671C/−*^ mice. RNA steady state levels from mtDNA encoded genes *mt-Nd4*, *mt-Cox1*, *mt-Rnr1* (12S), and *mt-Rnr2* (16S) were quantified by RT-qPCR and normalized by *Ppia* mRNA levels. Determinations were performed on total RNA from (**A**) livers of mice treated with the liver-targeted vector and (**B**) brains of mice treated with the CNS-targeted vector. The values are expressed as a relative quantity (percentage of wild-type mean), and bars represent the mean relative quantity ( ± SD). Separated statistical analyses were performed in males and females in the liver-targeted approach, while analysis in the CNS-targeted study was not split by sex. The exact *n* for each experiment is indicated in Appendix Table S1. Asterisks indicate statistical differences between groups (*P < 0.05, **P < 0.01, ***P < 0.001, ****P < 0.0001, Kruskal–Wallis and Dunn’s multiple comparisons test). Hash marks indicate statistical differences between KI/KO V and KI/KO AAV mice (^#^*P* < 0.05, ^##^*P* < 0.01, ^####^*P* < 0.001, Mann–Whitney *U* test). RQ relative quantification. Source data are available online for this figure.

Analyses performed using livers from mice subjected to AAV9-hAAT-GFM1 vector treatment and brains from mice included in the AAV9P31-hSyn-GFM1 study. Protein levels were analyzed using mitochondrial protein pellets, and mtRNAs were quantified in total RNA from the target tissues. The values are represented as the mean percentage ±SD, being referred to against 100% of the WT mean. The corresponding graphs are represented in Figs. 6 and  7.

On the other hand, RT-qPCR analysis revealed a significant increase of some mitochondrial mRNA (mt-mRNA) species (*Nd4* and *Cox1* mRNAs) and mt-rRNA species (*12S* and *16S* rRNAs) in the liver and brain of KI/KO mice, as compared to WT animals. While the liver-targeted therapy did not have any detectable effect on the mt-mRNA levels, it partially reversed the expansion of both mt-rRNAs observed in the KI/KO mice (Fig. 7A; Table 1). The CNS-targeted therapy partially reversed the increased mt-mRNA and mt-rRNA levels observed in vehicle-treated KI/KO mice (Fig. 7B; Table 1). The fact that the effect of mt-RNA expansion was more evident in the CNS than in the liver could be in part due to the larger number of animals in the CNS group.

Finally, because our results had shown a trend towards increased CS activity and assembled complex II levels in the vehicle-treated mice, we investigated mtDNA replication and mitochondrial proliferation as an additional potential compensatory mechanism in response to EFG1 depletion. Protein levels of CS, subunit A of succinate dehydrogenase (SDHA), and voltage dependent anion channel 1 (VDAC1) are commonly used as markers of mitochondrial content. We did not observe a significant increase in any of these proteins in the brain of untreated KI/KO animals, although some of them showed a tendency to be higher in the liver of these animals (Appendix Fig. S4A). Similarly, the mtDNA copy number was unaltered in the liver and brain of the *Gfm1*^*R671C/−*^ animals, suggesting that processes related to mtDNA replication and/or maintenance are not stimulated in the target tissues of these animals (Appendix Fig. S4B).

## Discussion

Currently, there is no curative treatment for COXPD1, making it impossible to control disease progression or prevent the premature death of patients (Antonicka et al, 2006; Coenen et al, 2004; Ravn et al, 2015). Moreover, it is challenging to attenuate the severe clinical traits of most patients or to improve their quality of life (Aleksic et al, 2023; Barcia et al, 2020; Bravo-Alonso et al, 2019; Brito et al, 2015; Galmiche et al, 2012; Nayan et al, 2024).

As a monogenic recessive disease, providing a functional copy of the *GFM1* gene to cells of the most affected tissues in patients, the brain and liver, is a plausible approach to stop the disease progression and to prevent some of its clinical manifestations. In the present study, we first showed that treating COXPD1 patients’ fibroblasts with a lentiviral vector carrying the *GFM1* coding sequence, under the control of the hPGK promoter, leads to efficient EFG1 expression, correcting the molecular phenotype, similarly to what had been reported in complementation analyses using patients’ fibroblasts (Coenen et al, 2004; Smits et al, 2011). The OXPHOS dysfunction (CI and CIV deficiency) was substantially reverted, but the correction was incomplete. The reasons for this insufficiency are unknown, considering that EFG1 levels in LV-treated patient cells were 3 to 5-fold above those observed in control cells. A possible explanation for this partial restoration of the downstream effect of EFG1 is that not all cells were effectively transduced by the LV, which would partially dilute the full restoration in transduced cells. However, we cannot rule out that the LV-mediated over-restoration of EFG1 levels could result in a molecular imbalance that makes mitochondrial translation less efficient than that observed with normal EFG1 levels.

Next, leveraging the availability of the compound heterozygous knock-in knock-out (KI/KO) mouse *Gfm1*^*R671C/−*^, which recapitulates the COXPD1 molecular phenotype in liver and brain (Molina-Berenguer et al, 2022), we conducted a gene therapy study using two rAAV vectors carrying the human *GFM1* gene and designed to drive the transgene expression specifically in the liver or the brain, with the aim to correct the COXPD molecular phenotype in these tissues. We prioritized targeting these organs because they are the most affected in COXPD1 patients, and selected the human *GFM1* coding sequence to facilitate further translation to the clinic.

We first focused on the liver as a target tissue because most rAAV vectors have a strong liver tropism (Zincarelli et al, 2008). Among those serotypes with hepatic tropism, we selected the AAV9 capsid. AAV9 has been described to have a certain capacity to cross the blood-brain barrier (BBB) after systemic injection (Manfredsson et al, 2009). However, the transduction levels of the AAV9-hAAT-GFM1 in the brain were negligible, in contrast to what has been reported in other studies (Foust et al, 2009; Gray et al, 2011). Thus, we designed a brain-targeted rAAV using the engineered AAV9P31 capsid, which has been reported to result in 385-fold higher EGFP expression levels in the mouse CNS and 1000-fold higher in the mouse spinal cord, as compared to the wild-type AAV9 (Nonnenmacher et al, 2021).

We have not yet identified an appropriate blood biomarker associated with the COXPD profile in *Gfm1*^*R671C/−*^ mice, and thus, monitoring the effect of the gene therapy in live animals over time was not possible. Therefore, we decided to analyze the effect of the therapy in liver and brain four weeks after vector administration, based on what was observed in previous studies reporting successful AAV-mediated transduction (Bottani et al, 2014) and transgene expression (Cabrera-Perez et al, 2019). Our results confirmed that the efficacy of the therapy was clearly detectable after this short time. Analyzing the COXPD phenotype beyond this time (e.g., at 30 weeks of age, since *Gfm1*^*R671C/−*^ mice maintain the COXPD phenotype at this age) would add valuable information, such as which is the long-term effect of the therapy and whether there is dilution of the transgene over time.

Although the aim of our study is focused on evaluating the efficacy of our gene therapy approach, the observation that administration with AAV9-hAAT-GFM1 and AAV9P31-hSyn-GFM1 vectors was not associated to body or tissue alterations in KI/KO mice supports the safety of the treatment, which is relevant as some deleterious effects associated with AAV-mediated therapies have been observed in other studies (Chandler et al, 2015; Donsante et al, 2007). However, four weeks is certainly too short to detect potential toxic effects of an AAV-mediated gene transfer therapy, and further studies aimed at exploring the biosafety of our treatment will be necessary.

One factor that will certainly influence the effect of the therapy is the vector dose. We did not conduct a dose–response study because our aim was to generate a first body of results showing that an AAV-mediated gene therapy ameliorates the molecular/biochemical phenotype observed in the COXPD1 animal model, thus identifying a potential therapy to be further investigated in additional studies. Our previous preclinical studies using AAV8 vectors for mitochondrial neurogastrointestinal encephalomyopathy showed that doses around 5 × 10^12^ vg/kg were sufficient to restore the molecular function of the transgene (Cabrera-Perez et al, 2019; Torres-Torronteras et al, 2018; Torres-Torronteras et al, 2014; Vila-Julià et al, 2020), and this dose falls within the range of doses used in approved treatments and clinical trials using AAV vectors with different serotypes (Byrne et al, 2025). On the other hand, COXPD1 is a recessive disease, and our previous characterization of the animal model showed that heterozygous KI animals had reduced levels of EFG1 but preserved OXPHOS function (Molina-Berenguer et al, 2022). Accordingly, the partial restoration of EFG1 levels observed with this dose led to a nearly total recovery of OXPHOS function, as discussed below. Therefore, the results obtained with this dose are informative about the potential of an AAV-based therapy for this disease, although further investigations should be conducted exploring the influence of the dose on both efficacy and safety endpoints.

Liver transduction in mice has been reported to be up to seven times more efficient in males than in females due to the influence of androgens (Davidoff et al, 2003). In our studies, the VCN was around threefold higher in livers from males than females for both vectors, consistent with the fact that 6-week-old mice already present hormonal differences between sexes. However, it has been reported that there are no sex-dependent liver transduction differences in non-human primates and patients (Pañeda et al, 2013), which would facilitate the design of a potential clinical trial for this therapy.

In contrast, no differences in the transduction efficiency of the AAV9P31-hSyn-GFM1 vector were observed between males and females in the brain. The AAV9P31 capsid proved to be a better option for targeting the mouse brain, as it facilitated the delivery of a greater number of copies of the *GFM1* transgene to this tissue than the AAV9. In mice, the AAV9P31 capsid interacts with the carbonic anhydrase IV (Car4), the receptor enabling the vector to cross the blood-brain barrier (BBB) (Shay et al, 2023). However, although a recent study speculated that carbon anhydrases are conserved in vertebrates and that AAV9P31 could interact with the human receptors, the experimental results revealed undetectable binding of AAV9P31 to human Car4 (Zhang et al, 2024). Furthermore, experiments in non-human primates with AAV9P31 revealed that this serotype was not able to cross the BBB after systemic injection (Bunuales et al, 2024). This information indicates that for a potential translation of the AAV9P31-hSyn-GFM1, the vector capsid should be replaced with one that efficiently crosses the BBB in humans. Therefore, until the efficiency to cross the BBB in humans is improved, the dose tested in this study (5 × 10^12^ vg/kg) is too low for targeting the CNS in patients by intravenous AAV infusion, and future dose escalating studies will have to be conducted to find the maximum tolerated dose leading to full rescue of the CNS phenotype in the animal model.

Biodistribution data for AAV9 and AAV9P31 in mice and other species have been reported elsewhere (Bunuales et al, 2024; Walkey et al, 2025), and a formal biodistribution study was beyond the scope of this work. Nonetheless, we analyzed the transduction levels in some non-target tissues (skeletal muscle, heart, and spleen), and the results revealed that the VCN was low but not negligible (between 0.01 and 0.1 vg/cell in most cases, although it reached higher values, up to 0.3 vg/cell, in the spleen of some animals, especially for the AAV9P31 vector). These results agree with what has been reported for AAV9 (Walkey et al, 2025) and AAV9P31(Bunuales et al, 2024) in mice, and suggest that non-target tissues should not be neglected if accurate safety studies are conducted in the future for this therapy approach.

The expression of the therapeutic gene was controlled by tissue-specific promoters. The hepato-specific enhancer-promoter ApoE-hAAT cassette has been demonstrated to effectively promote hepatic expression of the transgene using AAV vectors in mice in our laboratory (Cabrera-Perez et al, 2019; Vila-Julià et al, 2020), and has been successfully used to control the expression of UGT1A1 in an AAV clinical trial for the treatment of the Crigler-Najjar syndrome (D’Antiga et al, 2023). The CNS-targeted AAV contained the hSyn1 promoter, with neuron-specific expression (McLean et al, 2014). Both promoters resulted in substantial *GFM1* expression in their respective target tissues.

Although we used rAAV vectors with liver- and brain-targeted promoters (the most severely affected tissues in COXPD1), the expression of *GFM1* is ubiquitous and thus patients with COXPD1 (as well as the *Gfm1*^*R671C/−*^ mice) present EFG1 depletion in all studied tissues (Antonicka et al, 2006). In this regard, we chose the neuron-specific hSyn promoter for our CNS-targeted vector because it allowed us to study the effect in neurons, which constitute most cellular mass in the CNS. As we just mentioned, the molecular restoration observed in the total brain was relevant and nearly total when assessed as CI and CIV enzyme activities, showing the potential of this therapy when targeted to the CNS. However, the hSyn promoter does not promote the transgene expression in other brain cell types, such as glial cells, which, although being less contributors to the total CNS mass, have an important physiological function. Therefore, the restoration of the mitochondrial function of this subpopulation may also be required to ensure clinical efficacy. The construction of a vector carrying a ubiquitous promoter would be an alternative for spreading the therapy effects to more cell types and tissues (Grieger and Samulski, 2012; Slone and Huang, 2020). However, this strategy poses a risk of adverse effects, highlighting the challenge of finding a dosage that is sufficient to correct OXPHOS dysfunction while minimizing the potential for unwanted outcomes (Slone and Huang, 2020). Furthermore, using ubiquitous promoters favors the expression of the transgene product in the antigen-presenting cells, thus increasing the risk of immune response induction against the therapeutic gene (Sun et al, 2005).

Other studies where the AAV vector carries a human gene whose product localization is mitochondrial show that the transgenic human protein is translated in the cytosol and imported into mitochondria (Di Meo et al, 2017; Lopez-Gomez et al, 2021), and this critical step has been purposely analyzed (Guy et al, 2009). Consistently, here we provide evidence that the transgenic human EFG1 is correctly transported from the cytosol into mouse mitochondria in the liver and brain. Furthermore, our results show that the human protein is functional in the murine mitochondrial translation machinery.

Liver mitochondria showed nearly full recovery of the analyzed mtDNA-encoded CI and CIV subunits in KI/KO mice after AAV9-hAAT-GFM1 treatment, indicating an increase in mitochondrial translation rate derived from human EFG1 activity. In addition to the restoration of the mtDNA-encoded subunits of the OXPHOS system (such as COX1 and COX2), nuclear-encoded subunits, which are also depleted in untreated KI/KO mice (such as NDUFA9 and NDUFA10), showed increased steady-state levels after the treatment. These observations strongly suggest that the improved availability of complex subunits translated in mitochondria after rAAV treatment of KI/KO animals resulted in the correct assembly with nDNA-encoded subunits, preventing them from being degraded in a free state.

In accordance with the restoration of the steady state levels of mtDNA and nDNA encoded OXPHOS subunits, the amount of fully assembled RC complexes is also stabilized, and the CI and CIV enzyme activities are restored, reaching an almost full correction of the COXPD molecular phenotype in treated mice. Actually, the small sample size and the need to use nonparametric statistical methods may compromise the detection of some additional differences. For instance, the trend to reduced levels of assembled CIII in the liver of vehicle-treated KIKO mice does not reach statistical significance, probably due to the reduced number of mice, which were separated by sex. By contrast, this reduction reaches statistical significance in the brain, where the sample is larger because males and females were pooled. Previous reports had shown that AAV-based gene therapy aimed to restore nDNA encoded subunits (NDUFS4) (Di Meo et al, 2017; Reynaud-Dulaurier et al, 2020; Silva-Pinheiro et al, 2020), mtDNA encoded subunits (ND4) (Pereira et al, 2020) or assembly and stabilization factors (SURF1) (Ling et al, 2021), were able to restore the balanced amounts of complex subunits encoded in both genomes and to correct the specific OXPHOS complex defect of the model. Moreover, our study has made a step forward by increasing the pool of polypeptides synthesized through mitochondrial translation and balancing them with nDNA encoded subunits, thus restoring CI and CIV to nearly normal levels.

In *Gfm1*^*R671C/−*^ mice, the COXPD molecular phenotype is more evident in the liver than in the brain (Molina-Berenguer et al, 2022), which may favor the detection of the hepatic therapeutic effect. Furthermore, the CIV dysfunction is more pronounced than that of CI in both tissues, which also facilitates noticing the correction of that RC complex (Molina-Berenguer et al, 2022). This differential molecular picture may have contributed to the specificities of our results in the brain. In addition, the brain’s complexity in terms of cellular diversity and the associated vector biodistribution complicates the analysis of the therapeutic efficacy throughout the tissue, which we believe further contributes to the observation that the efficacy of CNS-targeted gene therapy was not as clearly detected as it was in the liver. Treatment with the AAV9P31-hSyn-GFM1 vector led to a significant recovery of CIV subunits in brain mitochondria of KI/KO mice, but only a subtle recovery of assembled CI and CIV levels was detected. However, spectrophotometric analyses of CI and CIV enzyme activities in brain homogenates (which are quantitatively more accurate than the densitometry-based quantification of bands in western blots) clearly showed a significant increase in AAV9P31-hSyn-GFM1-treated mice, highlighting the positive effect of the therapy.

The results obtained with the liver-targeted vector support the notion that EFG1 is in excess in WT mice, and a partial restoration of its levels above a certain threshold could be enough to correct the combined OXPHOS defect in the KI/KO mice. In fact, livers from KI/KO females treated with the liver-targeted vector presented lower levels of the *GFM1* transgene than those observed in treated males, but the assembled CI and CIV amounts and their enzyme activities reached similar values in both sexes. Therefore, it can be expected that targeting other murine tissues or even translating the therapy to humans may provide sufficient correction of the COXPD1 molecular phenotype even if EFG1 levels do not fully normalize. On the other hand, we are aware that the more widespread the transduction throughout the target tissue is, the more effective the therapy will be, as the COXPD may just be corrected in transduced cells. Immunohistochemical (IHC) analyses of EFG1 in the liver showed that the AAV9-hAAT-GFM1 vector reached the whole organ, transducing a vast proportion of the target cells, and the gene was expressed throughout the tissue. However, we did not conduct similar histological analyses in brains from *Gfm1*^*R671C/−*^ mice treated with the AAV9P31-hSyn-GFM1 vector, although other studies have already reported that a similar i.v. dose of a vector with the AAV9P31 capsid results in high EGFP reporter expression in all brain regions (Bunuales et al, 2024; Nonnenmacher et al, 2021). Nevertheless, it would be informative to conduct further studies in order to optimize the distribution of transgenic EFG1 in both target tissues.

Despite the profound EFG1 depletion observed in *Gfm1*^*R671C/−*^ mice, the COXPD molecular phenotype in liver and brain was moderate and did not lead to relevant further pathogenic effects on the function of these organs. We aimed to explore whether certain compensatory mechanisms could counteract EFG1 depletion. The study of other mouse models with mitochondrial translation dysfunction and OXPHOS defects has revealed an increase of mtRNA basal levels (Ferreira et al, 2019; Lagouge et al, 2015; Metodiev et al, 2009) and induction of mitoribosome biogenesis (Camara et al, 2011; Lagouge et al, 2015; Metodiev et al, 2014; Rudler et al, 2019). We detected a significant increase in some mtDNA encoded mRNA (*Nd4* and *Cox1*) and rRNA (*12S* and *16S*) levels in liver and brain in KI/KO mice, suggesting that the mitochondrial translation dysfunction could trigger mitochondrial transcription stimulation and/or stabilization of the resulting transcripts. We also observed increased levels of some mitoribosome subunits in liver and brain mitochondria of KI/KO mice, in accordance with the rRNA increase, suggesting mitoribosome biogenesis induction. Importantly, the treatments with rAAV partially normalized those molecular responses in the liver and brain, reinforcing the notion that they could be to some extent compensating for the EFG1 depletion and preventing severe effects in the KI/KO mice, and further supporting the efficacy of our gene therapy approach.

Overall, we have shown that an AAV-based approach has a translational potential for this fatal disorder, but we must acknowledge that some limitations of this study will have to be further addressed before the clinical phases. Some of them are inherent to the animal model, which fails to recapitulate the clinical phenotype of the patients, at least to the degree that we have studied the mice so far (Molina-Berenguer et al, 2022). We cannot rule out that a more in-depth neurobehavioral assessment could reveal so far hidden subtle phenotypical traits, and if this were the case, the effect of this therapy approach on them would have to be explored. Other limitations derive from the therapeutic tool in itself. The use of AAV vectors often triggers immunological responses (*vs*. the vector and/or the transgene) that need to be minimized and controlled in the context of clinical use (Keeler et al, 2025). As previously mentioned, our study did not conduct a proper toxicity evaluation and therefore could not rule out the possibility of immunological events or other potential adverse effects. In addition, since the transduced gene remains episomal after AAV transduction, some dilution effect in proliferating tissues, such as liver, could be a potential issue compromising the long-term effect of the therapy, which has not been studied here and will need to be explored in future studies. In this regard, our observation that the molecular phenotype remains altered in untreated mice at least until 30 weeks of age will facilitate the exploration of these long-term effects, as previously noted.

Although COXPD1 is certainly a rare disease, 20 new cases of patients with COXPD1 have been reported since 2019 (Aleksic et al, 2023; Barcia et al, 2020; Bravo-Alonso et al, 2019; Khan et al, 2022; Nayan et al, 2024; Su and Wang, 2020; You et al, 2020), which constitutes a 48.8% of the total cases so far reported, and these figures could be notably increased during the next years. Our work provides evidence that *GFM1* gene therapy using rAAV vectors constitutes a potentially effective approach to treat COXPD1 that deserves to be studied more in-depth in order to further confirm its efficacy and to advance toward its use in patients.

## Methods

**Table:** Reagents and tools table

Reagent/resource	Reference or source	Identifier or catalog number
**Experimental models**		
Primary skin fibroblasts P1 (*H. Sapiens*)	Brito et al, 2015	N/A
Primary skin fibroblasts P2 (*H. Sapiens*)	University Pablo de Olavide (Seville, Spain)	N/A
Primary skin fibroblasts P3 (*H. Sapiens*)	University Pablo de Olavide (Seville, Spain)	N/A
Primary skin fibroblasts C1 (*H. Sapiens*)	Vall d’Hebron Research Institute, our own sample collection	Sample collection # 0003899, Instituto de Salud Carlos IIII, Spain
Primary skin fibroblasts C2 (*H. Sapiens*)	Vall d’Hebron Research Institute, our own sample collection	Sample collection # 0003899, Instituto de Salud Carlos IIII, Spain
Strain G *Gfm1*^*R671C/−*^ (*M. Musculus*)	Molina-Berenguer et al, 2022	N/A
HEK293T cell line (*H. Sapiens*)	ATCC	CRL-11268
**Recombinant DNA**		
pMK-RQ GFM1-Insert plasmid	ThermoFisher	NCBI accession NM_024996.5 (for the cGFM1 insert)
pCCL.sin.PPT.hPGK.IRESem.vcv.wt.eGFP.Wpre	Torres-Torronteras et al, 2011	N/A
p305-GFM1LV	This study	N/A
pCCL.sin.PPT.hPGK.wt.eGFP.Wpre	San Raffaele Telethon Institute for Gene Therapy	N/A
AAV9-AAT-GFM1	This study	N/A
AAV9P31-hSyn-GFM1	This study	N/A
**Antibodies**		
Anti-GFM1 (for western blot)	Abcam	ab173529
Anti-GFM1 (for immunohistochemistry)	Proteintech	14274-1-AP
Anti-SDHA	Abcam	ab14715
Anti-NDUFA9	ThermoFisher	20C11B11B11
Anti-NDUFA10	GeneTex	GTX114572
Anti-COX1	ThermoFisher	459600
Anti-COX2	GeneTex	GTX33329
Anti-MRPS9	Boster Biological Technologies	A14072-1
Anti-MRPS35	Proteintech	16457-1-AP
Anti-MRPL13	Boster Biological Technologies	A13508-1
Anti-MRPL37	Proteintech	15190-1-AP
Anti-CS	Cell Signaling	14309
Anti-VDAC1	Abcam	ab15895
Anti-GAPDH	Cell Signaling	TA802519
Goat Anti-Rabbit Immunoglobulins/HRP (for western blot)	Dako	P0448
Rabbit Anti-Mouse Immunoglobulins/HRP (for western blot)	Dako	P0161
DISCOVERY UltraMap anti-Rb HRP (for immunohistochemistry)	Roche	760-4315
**Oligonucleotides and other sequence-based reagents**		
ITR primer forward 5’-GGAACCCCTAGTGATGGAGTT-3’	This study	N/A
ITR primer reverse 5’-CGGCCTCAGTGAGCGA-3’	This study	N/A
16S custom-designed TaqMan probe: FAM-5’ AAGTCCTACGTGATCTGAGGT 3’-MGB	ThermoFisher	N/A
16S custom-designed forward primer: 5’-AATGGTTCGTTTGTTCAACGATT-3’	ThermoFisher	N/A
16S custom-designed reverse primer: 5’-AGAAACCGACCTGGATTGCTC-3’	ThermoFisher	N/A
**Chemicals, enzymes, and other reagents**		
DMEM High glucose medium	ThermoFisher	11965092
Fetal bovine serum	ThermoFisher	A5256701
Penicillin/Streptomycin mix	ThermoFisher	15140122
L-glutamine	ThermoFisher	25030081
MEM non-essential amino acids	ThermoFisher	11140050
Sodium pyruvate	ThermoFisher	11360070
Nonidet	Sigma-Aldrich	56741
SDS	Sigma-Aldrich	11667289001
EDTA	Sigma-Aldrich	798681
Sodium deoxycholate	Sigma-Aldrich	264103
Tris	Sigma-Aldrich	93362
cOmplete™ Protease Inhibitor Cocktail	Sigma-Aldrich	11873580001
Polyethyleneimine polymer (PEI)	Sigma-Aldrich	40,872-7
Polyethylene glycol solution (PEG8000, 8% v/v)	Sigma-Aldrich	P5413
DNaseI	Roche	10104159001
RNase A	Roche	10109169001
Optiprep Density Gradient Medium-Iodixanol	Sigma-Aldrich	D1556-250ML
High Pure Viral DNA Kit	Roche	11858874001
GOTaq qPCR master mix 2X	Promega	A600A
Pluronic acid F68	PanReac-Applichem	A1288,0500
TES (2-[[1,3-dihydroxy-2-(hydroxymethyl)propan-2-yl]amino]ethanesulfonic acid	ThermoFisher	B21819.30
TaqMan MGB expression assay Hs00227997_m1	ThermoFisher	4331182 (Hs00227997_m1)
TaqMan MGB gene expression assay Mm00833184_s1	ThermoFisher	4331182 (Mm00833184_s1)
*TRIzol*^*TM*^ *Reagent*	ThermoFisher	15596026
DNA-freeTM Kit Procedure	ThermoFisher	AM1906
High-Capacity cDNA Reverse Transcription Kit	ThermoFisher	4374967
TaqMan Universal PCR Master Mix II, with UNG	ThermoFisher	4440046
[^35^S]-Methionine	Revvity	NEG709A500UC
[^35^S]-Cysteine	Revvity	NEG022T001MC
UltraView Universal DAB Detection Kit	Roche	760-500
Tissue-Tek® H&E Staining Kit	Sakura	6190
Ventana Trichrome Staining Kit	Roche	860-031
Thermo Scientific™ Pierce™ Bradford Protein Assay Kit	ThermoFisher	23200
**Software**		
GraphPad Prism 9	GraphPad Software	N/A
**Other**		
Amicon Ultra-15ml	Sigma-Aldrich	UFC901008D
RNA 6000 Nano Kit for 2100 Bioanalyzer Systems	Agilent Technologies	N/A

### Patients’ fibroblast cell lines and culture conditions

The three patients’ fibroblast lines are compound heterozygous for mutations in *GFM1* (NCBI accession NM_024996.5): Patient 1 (P1) (c.1404delA p.(Gly469Valfs∗84); c.2011C>T p.(Arg671Cys)) (Fibroblasts from P1 were kindly provided by Dr. Rafael Artuch Iriberri from Institut de Recerca St. Joan de Déu, Barcelona), Patient 2 (P2) (c.2011C>T p.(Arg671Cys); c.179 C > G p.(Thr60Ser)) and Patient 3 (P3) (c.2011C>T p.(Arg671Cys); c.179 C > G p.(Thr60Ser)) (P2 and P3 fibroblasts were kindly provided by Dr. José Antonio Sánchez Alcázar from the University Pablo de Olavide, Seville) and two healthy controls’ fibroblast lines of similar ages (C1 and C2) were cultured in tissue treated flasks using DMEM high glucose medium (ThermoFisher) supplemented (10% fetal bovine serum; 100 μl/ml penicillin/streptomycin; 4.5 g/L L-glutamine). Cells were always cultured in humidified incubators at 37 °C with a 5% of CO_2_.

HEK293T cells were cultured in DMEM High glucose medium supplemented (10% decomplemented fetal bovine serum; 100 μl/ml Penicillin/Streptomycin; 4.5 g/L L-glutamine; MEM non-essential amino acids (Biowest #X0557-100); 1 mM sodium pyruvate) for lentiviral vector production and titration.

All cell cultures were tested for mycoplasma contamination and resulted in negative.

### Lentiviral vector (LV) construction, production, titration, and transduction

The human *GFM1* cDNA (NCBI accession NM_024996.5) inserted into the plasmid pMK-RQ GFM1-Insert plasmid was purchased from Thermofisher (Waltham, MA). The *GFM1* cDNA was cloned into the lentiviral transfer vector backbone p305 (pCCL.sin.PPT.hPGK.IRESem.vcv.wt.eGFP.Wpre), obtaining the p305-GFM1LV plasmid (Appendix Fig. S1A). The LV called pSham (pCCL.sin.PPT.hPGK.wt.eGFP.Wpre) was kindly provided by Dr. Luigi Naldini (San Raffaele Telethon Institute for Gene Therapy) (Appendix Fig. S1B).

The LVs were produced by transient co-transfection of HEK293T cells and titrated using the same cell line as previously described (Torres-Torronteras et al, 2011). The patients’ fibroblast lines were transduced with p305-GFM1LV or pSham LV. The day before transduction, 10^5^ cells/well were seeded in 6-well plates. Fibroblasts from each patient were transduced with LVs at two different multiplicities of infection (MOI 10 and 50). We chose a moderate and a high MOI to maximize the chances of efficient transduction because the primary skin fibroblasts are slow-cycling cells, and LV transduction efficiency is lower in non-dividing cells (Naldini et al, 1996) than in the proliferating cell line used for the LV titration. The LV-containing medium was replaced 24 h after transduction with fresh LV-free medium, and cells were maintained in culture for an additional month with medium replacement whenever necessary to ensure stable cell transduction and no pseudo-transduction (Liu et al, 1996).

### Fibroblast extracts for western blot, SDS-PAGE, and RC activities determination

For western blot analyses, cell pellets were homogenized in 200 µL of RIPA buffer (1% Nonidet, 0.1% SDS, 1 mM EDTA, 150 mM NaCl, 0.5% sodium deoxycholate, and 50 mM Tris pH 7.4, cOmplete™ Protease Inhibitor Cocktail (Sigma-Aldrich#11873580001) by passing through a U-100 insulin syringe (BD Micro-Fine; 0.30 × 8 mm). The homogenates were centrifuged at 20,000×*g*, 4 °C for 10 min, the supernatants were recovered, protein concentration quantified using the *Bradford* method (Bradford, 1976), and 20 μg of denaturalized protein extracts were resolved through SDS-PAGE and analyzed by western blot.

For the RC complex activities determination, approximately, 10^6^ frozen cells were resuspended in 200 μl of mannitol buffer (225 mM mannitol, 75 mM sucrose, 10 mM Tris-HCl, 0.1 mM EDTA, pH 7.2) and homogenized by sonication for 5 s at 60% intensity with Microson Ultrasonic Cell Disruptor XL2000 (Misonix, Farmingdale, NY), centrifuged at 650×*g* during 20 min at 4 °C and the supernatant was recovered. The protein content was determined, and 1 mg/ml of proteins was aliquoted and stored at −80 °C to measure the enzyme activities of RC complexes I and IV, and CS (Bujan et al, 2022).

### Recombinant ssAAV vectors construction, production, and titration

The human coding sequence of *GFM1* (hcGFM1) (NCBI accession NM_024996.5) was cloned into two different single-stranded vectors: liver-targeted (AAV-AAT) and CNS-targeted (AAV-hSyn) constructions were packaged in AAV9 and AAV9P31 serotypes, respectively.

The recombinant single-stranded vectors AAV9-AAT-GFM1 and AAV9P31-hSyn-GFM1 (Appendix Fig. S2A) were produced in HEK-293T cells co-transfected (polyethyleneimine polymer (PEI), Sigma-Aldrich, Saint Louis, MO, #40,872-7) with two different plasmids: the first one included the expression cassettes flanked by ITRs, and the second one carried the adenoviral genes required for rAAV production, AAV2 *rep* genes and AAV9 or AAV9P31 *cap* genes.

Supernatants and cells were collected 72 h post-transfection. Supernatants were treated with a polyethylene glycol solution (PEG8000, 8% v/v Sigma-Aldrich #P5413) during 48–72 h at 4 °C, and centrifuged 15 min at 3000 rpm. Then, the pellets were resuspended in lysis buffer (50 mM Tris-HCl, 15 mM NaCl, 2 mM MgCl_2_, 0.1% Triton X-100) and preserved at −80 °C. Cells containing rAAV were collected and also treated with the lysis buffer. The cell lysis product was subjected to three freeze-thaw cycles. The lysate of cells and the PEG-treated supernatant were treated with DNaseI (Roche, Basel, Switzerland, #10104159001) and RNase A (Roche, #10109169001) 0.1 mg/10 µL, for 30–60 min at 37 °C, then kept at −80 °C until purification.

Viral particles were purified through ultracentrifugation in Optiprep Density Gradient Medium-Iodixanol (Sigma-Aldrich). Purified viral particles were concentrated in columns Amicon Ultra-15 ml (Amicon®; Millipore, Bedford, MA). To determine vector yield, viral DNA was extracted from 20 µL of the purified vector production using the High Pure Viral DNA Kit (Roche, #11858874001). Titers of the virus were calculated by qPCR using sequence-specific primers for ITR (Fw: 5’-GGAACCCCTAGTGATGGAGTT-3’ and Rv: 5’-CGGCCTCAGTGAGCGA-3’) and GOTaq qPCR master mix 2X (Promega, Madison, WI, #A600A) in a CFX96TM Real-Time PCR Detection system (Bio-Rad, Hercules, CA). Results were normalized with a standard curve generated using a serial dilution of the plasmid employed for the production. Titrated rAAV were reconstituted in the vehicle solution (5% w/v saccharose in PBS + 0.001% pluronic acid F68).

### Ethics statement for animal procedures

This study was conducted in accordance with the rules established by the Generalitat de Catalunya for the Care and Use of Laboratory Animals. The protocol was approved by the Ethics Committee for Animal Experimentation of the Vall d’Hebron Research Institute (Permit Number: 53/18).

### Mouse housing, tissue collection, and endpoints

The animal model used in this study was the compound heterozygous *Gfm1* knock-in (*Gfm1*^*R671C*^, KI) knock-out (*Gfm1*^*−*^, KO) mouse (*Gfm1*^*R671C/−*^, KI/KO) (Molina-Berenguer et al, 2022). Mice were housed and bred in a standard controlled environment under a 12-h light–dark cycle with ad libitum access to water and a regular rodent chow diet. Animal breeding and genotyping were performed as previously described (Molina-Berenguer et al, 2022).

The preclinical gene therapy study consisted of two approaches carried out independently in order to test the efficacy of the two generated rAAV vectors: a liver-targeted approach using the AAV9-AAT-GFM1 vector, and a CNS-targeted approach using the AAV9P31-hSyn-GFM1 vector. Both strategies followed an identical experimental design. The rAAV vectors were administered intravenously on 6-week-old *Gfm1*^*R671C/−*^ mice (KI/KO AAV) through tail vein injections at a 5 × 10^12^ vg/kg (vector genomes/mouse weight) dose (Appendix Fig. S2B). We chose this dose based on our previous experience with AAV in mice (Cabrera-Perez et al, 2019; Torres-Torronteras et al, 2018; Torres-Torronteras et al, 2014; Vila-Julià et al, 2020) and on ranges of doses used in approved AAV treatments and clinical trials. Treatment at six weeks of age was chosen to minimize the vector dilution effect, as mouse liver matures around weeks 6–8, reaching a weight plateau (Gong et al, 2020), and also considering the previous observation that *Gfm1*^*R671C/−*^ mice display molecular alterations already at this age (Molina-Berenguer et al, 2022), therefore allowing us to test whether the therapy can correct the phenotype. The control groups consisted of *Gfm1*^*R671C/−*^ animals treated with the equivalent volume vehicle solution (KI/KO V group) and untreated *Gfm1*^*+/+*^ (WT) mice. Each approach was performed in parallel on male and female mice (Appendix Fig. S2C). At least five different animals were allocated in each study group (WT, KIKO V, and KIKO AAV) and sex; in most cases, between 6 and 10 mice per group and sex. The exact *n* for each experiment is indicated in Appendix Table S1 and, in some cases, also in the corresponding figure legend. The result obtained for each individual animal is plotted in the figures with a single symbol (the number of represented values indicates the number of animals). The displayed values are the result of a single replicate per sample or, when indicated, the average of different replicates, depending on the variable (number of replicates used for each variable is indicated below for each method description involving replicates). The statistical measure represented by the bars and error bars is indicated in each figure legend.

Animals were allocated to different experimental groups without any specific randomization procedures. Animals with the appropriate genotype and sex were allocated to different groups based on availability from breeding. Blinding of the analyses was not implemented given the nature of the study.

Body weight and animal welfare were monitored during 4 weeks between the rAAV administration and the sacrifice. Ten-week-old mice were sacrificed by cervical dislocation and the target tissues were excised and preserved as follows: one liver lobe or brain hemisphere were immersed in cold homogenization buffer (0.25 M sucrose, 5 mM TES (2-[[1,3-dihydroxy-2-(hydroxymethyl)propan-2-yl]amino]ethanesulfonic acid), pH 7.2) for mitochondrial protein extractions, while the remaining tissue samples used for DNA, RNA or protein analyses were immediately preserved in liquid nitrogen. Tissue samples were long-term stored at −80 °C. In the case of livers from the AAV9-AAT-GFM1 study, one lobe was immersed in 1% paraformaldehyde and used for histological analyses.

The rAAV transduction efficiency was analyzed in the liver and brain by vector copy number (VCN) determination. Then, the efficacy was studied for each targeted approach (liver or CNS) by determining the therapeutic *GFM1* gene expression (mRNA and protein levels), analyzing CI and CIV subunit levels on mitochondrial extracts, assembled CI and CIV levels, and their enzyme activities. Additionally, we analyzed the effect of the therapy on the response mechanisms observed in *Gfm1*^*R671C/−*^ liver and brain: mtRNA (mRNA, rRNA) and mitoriboprotein (MRPs) steady-state levels.

### rAAV vector and mtDNA copy number determination

DNA was isolated from approximately 25 mg of frozen liver and brain samples using a phenol-chloroform extraction method, and resuspended in 10 mM Tris-HCl, pH 8.0. Vector genome copies per cell (vg/cell) were quantified by qPCR on the *QuantStudio 7 Pro Real-Time PCR System* (Applied Biosystems, Waltham, MA). Human *GFM1* copy number was quantified using the TaqMan MGB expression assay *Hs00227997_m1* and referred to the murine single-copy nuclear gene *Ang1* using the predesigned TaqMan MGB gene expression assay *Mm00833184_s1* as previously described (Torres-Torronteras et al, 2018). MtDNA copy number was quantified using the MGB 16S custom-designed TaqMan probe (FAM-5’-AAGTCCTACGTGATCTGAGGT-3’-MGB) and primers (16S Forward: AATGGTTCGTTTGTTCAACGATT and 16S Reverse: AGAAACCGACCTGGATTGCTC) and referred to the same murine single-copy nuclear gene *Ang1*. For each probe, two replicates of undiluted and 1/3 diluted DNA extract (a total of four replicates) were analyzed. Undiluted and diluted DNAs were analyzed to rule out potential PCR interferents in the extracts. The results represent averaged replicate values. In both cases, quantifications were determined by interpolation on standard curves of different dilutions of plasmids containing *hGFM1* coding sequence or a specific region of the *Ang1* or *16S* genes (Blazquez-Bermejo et al, 2019; Vila-Julià et al, 2020).

### RNA isolation and expression analysis

RNA was isolated from frozen and ground liver and brain samples using the TRIzol^TM^ Reagent protocol (Invitrogen, Thermo Scientific, Waltham, MA) and dissolved in RNAse-free water. RNA quality and quantity were assessed using RNA 6000 Nano Kit for 2100 Bioanalyzer Systems (Agilent Technologies, Santa Clara, CA). RNA samples were subjected to DNase treatment according to the DNA-free^TM^ Kit Procedure (Invitrogen, Thermo Scientific) and adjusted to 200 ng/μL with sterile double-distilled H_2_O. cDNA was synthesized from RNA using the High-Capacity cDNA Reverse Transcription Kit (Applied Biosystems) following the manufacturer’s instructions. RT-qPCR reactions were carried out in 384-well plates with TaqMan Universal PCR Master Mix II, with UNG (Applied Biosystems). Predesigned TaqMan Gene Expression Assays were used for quantification of *GFM1* mRNA *(Hs00227997_m1)*, *mt-Rnr1*/12S rRNA *(Mm04260177_s1*), and *mt-Co1* mRNA *(Mm04225243_g1)*. Mitochondrial 16S (*mt-Rnr2*) rRNA and *mt-Nd4* mRNA levels were analyzed using the specific *TaqMan™ MGB probes* as previously described (Cabrera-Perez et al, 2019). *Ppia* mRNA levels, using the *Mm02342430_g1 assay*, were used as the reference for normalization among samples. Each cDNA sample was analyzed in triplicate, using the average value as the mRNA measure.

### Mitochondria isolation

Mitochondrial extracts used for SDS-PAGE and BN-PAGE western blot analyses were obtained from liver and brain following a previously described protocol based on differential centrifugation isolation (Gonzalez-Vioque et al, 2011) with minor modifications (Molina-Berenguer et al, 2022). The mitochondrial protein concentration was quantified using the Bradford method (Bradford, 1976), and the obtained mitochondrial protein extracts were preserved at −80 °C until they were used.

### Western blot SDS-PAGE analysis

Protein pellets obtained from isolated mitochondria were diluted 1:2 in loading buffer (14% v/v glycerol, 2.8% w/w sodium dodecyl sulfate, 70 mM Tris-HCl, pH 6.8, 200 mM DTT, 0.1% w/v bromophenol blue). Protein extracts from whole tissue were obtained as follows: 20–50 mg of tissue powder were homogenized with 150 µL of Ripa buffer (50 mM Tris-HCl, pH 7.4, 50 mM NaCl, 1 mM EDTA, 0.1% w/v SDS, 0.5% w/v sodium deoxycholate, 1% v/v IGEPAL CA-630 (Sigma-Aldrich) and cOmplete^TM^, EDTA-free Protease Inhibitor Cocktail, Sigma-Aldrich, 11873580001). After centrifugation 10 min at 20,000×*g* and 4 °C, supernatants were kept, the protein concentration was quantified using the Bradford method, and the extracts were adjusted to 6 µg/µL with Ripa buffer. Then, the protein homogenates were diluted to 3 µg/µL using loading buffer 2×.

In both cases, the protein extracts (20–30 µg) were denatured at 95 °C for 5 min, resolved on 10–12% polyacrylamide gel at 120 V for 120 min and blotted to a polyvinylidene difluoride membrane (Immun-Blot® PVDF membrane, Bio-Rad) at 400 mA for 1 h at 4 °C. Protein membranes were blocked with 5% non-fat milk in TBST buffer (20 mM Tris-HCl, 150 mM NaCl, 0.1% w/v Tween 20, pH 7.6) at room temperature for 1 h before being incubated with primary antibodies overnight at 4 °C. Then, the membranes were incubated in TBST buffer for 30 min and in secondary antibodies for 1 h at room temperature. The membranes were washed 30 min in TBST and developed using the Millipore Immobilon Western Chemiluminiscence HRP Substrate (Fisher Scientific Co). Images were taken with Odyssey® - FC Imaging System (LI-COR Biosciences, Lincoln, NE) and analyzed with Image Studio Lite, version 5.2.

Antibodies were diluted in 5% non-fat milk TBST. Primary antibodies used at a 1:1000 dilution were: GFM1 *(*ab173529, Abcam, Cambridge, UK), SDHA (ab14715, Abcam), NDUFA9 (20C11B11B11, Thermo Fisher Scientific), NDUFA10 (GTX114572, GeneTex, San Antonio, TX), COX1 (459600, Thermo Fisher Scientific), COX2 (GTX33329, GeneTex), MRPS9 (A14072-1, Boster Biological Technologies, Pleasanton, CA), MRPS35 (16457-1-AP, Proteintech. Rosemont, IL), MRPL13 (A13508-1, Boster Biological Technologies), MRPL37 (15190-1-AP, Proteintech), CS (#14309, Cell Signaling, Danvers, MA), VDAC1 (ab15895, Abcam) and GAPDH (TA802519, OriGene, Rockville, MD). The secondary antibodies, used at a 1:5000 dilution, were Goat Anti-Rabbit Immunoglobulins/HRP (P0448, Dako, Glostrup, Denmark) and Rabbit Anti-Mouse Immunoglobulins/HRP (P0161, Dako).

### Western blot BN-PAGE analysis

BN-PAGE was carried out using the NativePAGE Novex Bis-Tris Mini gel system (Invitrogen, Waltham, MA) following the manufacturer’s specifications. Mitochondrial protein pellets from target tissues were solubilized using nondenaturing conditions (Wittig et al, 2006), and 10–20 µg of protein were loaded in the electrophoresis. Proteins were transferred onto a PVDF membrane, and assembled OXPHOS complexes were immunodetected using specific primary antibodies as described above (CI, NDUFA9; CII, SDHA; CIII, CoreII Subunit; CIV, COX1).

### Mitochondrial translation

In organello mitochondrial translation was performed as previously described (Wibom et al, 2002) with minor modifications (Molina-Berenguer et al, 2022). Briefly, the experimental protocol was based on radiolabelling with ^35^S of de novo synthesized mitochondrial proteins, using a translation buffer that contained a mix of [^35^S]-Methionine and [^35^S]-Cysteine (Perkin Elmer, Waltham, MA). After the separation of translation products through SDS-PAGE, the gel was fixed, stained with Coomassie, and revealed by autoradiography.

### Respiratory chain (RC) complex activities

Ground tissues were homogenized in a cold mannitol buffer. After centrifugation at 650×*g* at 4 °C, protein concentrations were determined in the supernatants using the *Bradford* method (Bradford, 1976). Tissue protein homogenates (0.67 mg/mL) in mannitol buffer were used for CI, CIV, and citrate synthase enzyme activities determination through a spectrophotometric method as previously described (Bujan et al, 2022). The specific absorbance of each reaction was measured using UV-2401PC UV-VIS recording spectrophotometer (Shimadzu, Kyoto, Japan).

### Liver histological evaluation

Liver samples from 10-week-old mice included in the AAV9-AAT-GFM1 study were fixed in paraformaldehyde and embedded in paraffin. Next, 4-μm slices were deparaffinized and hydrated before hematoxylin–eosin (HE) staining, Masson’s trichrome (TRIC) staining, and EFG1 immunohistochemistry (IHC) analysis. Finally, the sections were dehydrated, mounted on DPX-mounting medium, and scanned with Pannoramic 250 Flash III (3DHISTECH, Budapest, Hungary) for their evaluation using Case Viewer 2.4 software.

HE staining was performed using Tissue-Tek Plus (Sakura) system and Tissue-Tek® H&E Staining Kit (Sakura, Osaka, Japan). For TRIC staining, we used the BenchMark Special system (Roche) and reagents from Ventana Trichrome Staining Kit 860-031 (Roche), following the manufacturer’s recommendations.

EFG1 IHC was completed on BenchMark ULTRA IHC/ISH System (Roche) using ultraView Universal DAB Detection Kit - 760-500 (Roche), replacing the kit HRP antibody with the *DISCOVERY* UltraMap anti-Rb HRP, 760-4315 (Roche) secondary antibody. The epitope retrieval was done with Discovery Cell Conditioning CC1 pH 8.0 at 95 °C for 64 min. Blocking with Hydrogen Peroxide 3% was performed before the incubation with GFM1 antibody, 14274-1-AP (Proteintech) dil.1/50 for 1 h, and localization with the anti-rabbit secondary antibody. The process finished with DAB visualization and HE counterstain.

### Statistical analyses

Statistical analyses were performed using GraphPad Prism 9 (GraphPad Software, Inc., La Jolla, CA). Nonparametric tests were used in most comparisons due to the limited number of cases per group, which makes it difficult to robustly determine that the distributions meet the requirements of the parametric statistical tests. Mann–Whitney test was applied for two-group comparisons. Kruskal–Wallis test was performed for multiple comparisons, and when it reached statistical differences, Dunn’s multiple comparisons test was applied. Tests used on each particular case are detailed in the figure legends, and statistical significance was accepted when *P* < 0.05. Exact *P* values and exact numbers of animals or replicates for each group/condition can be found in the Appendix Table S1. In some cases, the *n* is also indicated in the figure legends.

## Supplementary information

Peer Review File

Source data Fig. 1

Source data Fig. 2

Source data Fig. 3

Source data Fig. 4

Source data Fig. 5

Source data Fig. 6

Source data Fig. 7

Figure EV1 Source Data

Figure EV2 Source Data

Figure EV3 Source Data

Figure EV4 Source Data

Figure EV5 Source Data

Appendix

Expanded View Figures

## Data Availability

This study includes no data deposited in external repositories. The source data of this paper are collected in the following database record: biostudies:S-SCDT-10_1038-S44321-026-00426-4.
