# Supplementary material for: Systemic delivery of AAV-GFM1 corrects COXPD1 molecular alterations in Gfm1R671C/− mice
Source: EMBO Mol Med. 2026 Apr 17;18(6):2152–79. doi: 10.1038/s44321-026-00426-4 (PMC13269562; doi:10.1038/s44321-026-00426-4)

Liver mitochondria
10 weeks old mice
ssAAV9-ApoE-hAAT-intron-GFM1

24/05/22

Western blot – SDS-PAGE

Females ♀

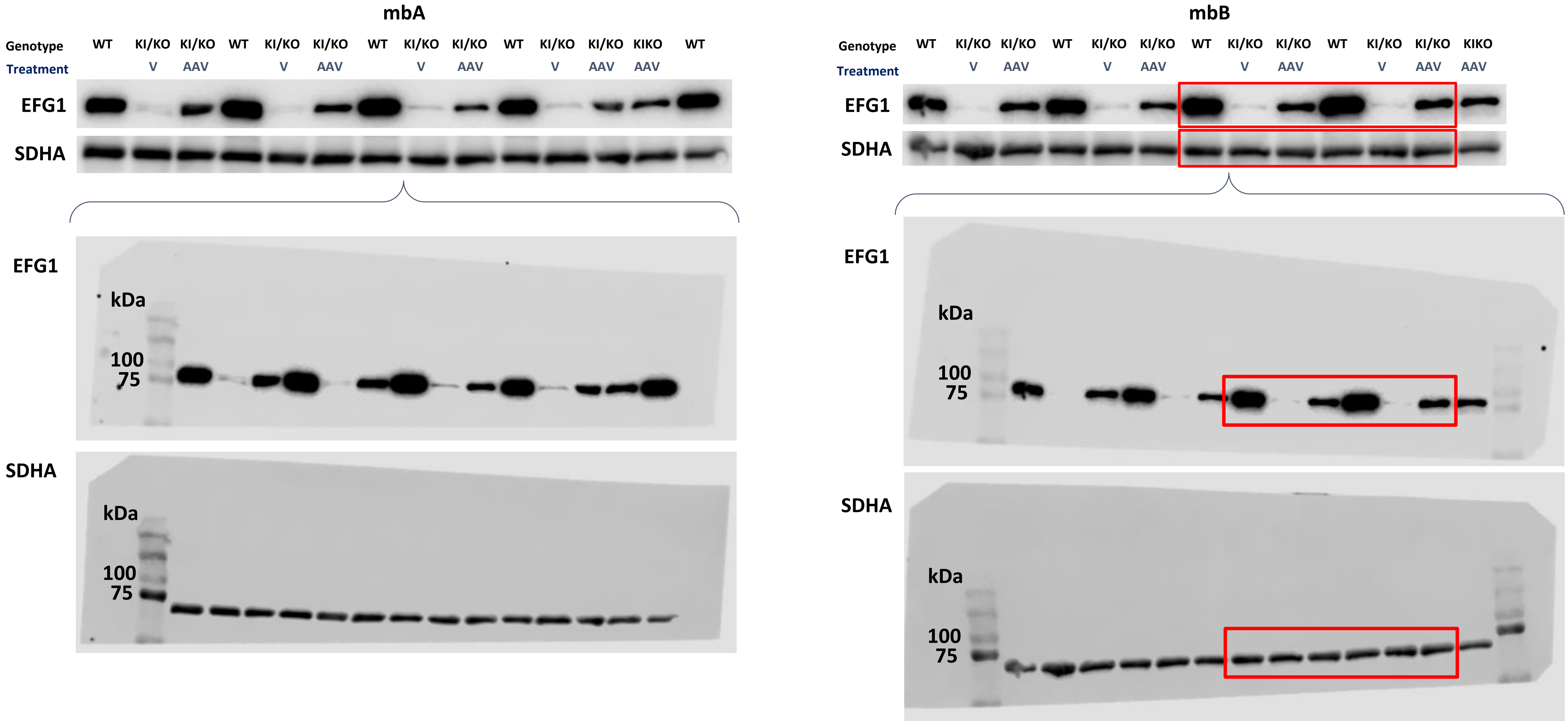

Selected area for publication

Liver mitochondria
10 weeks old mice
ssAAV9-ApoE-hAAT-intron-GFM1

15/06/22

Western blot – SDS-PAGE

Males ♂

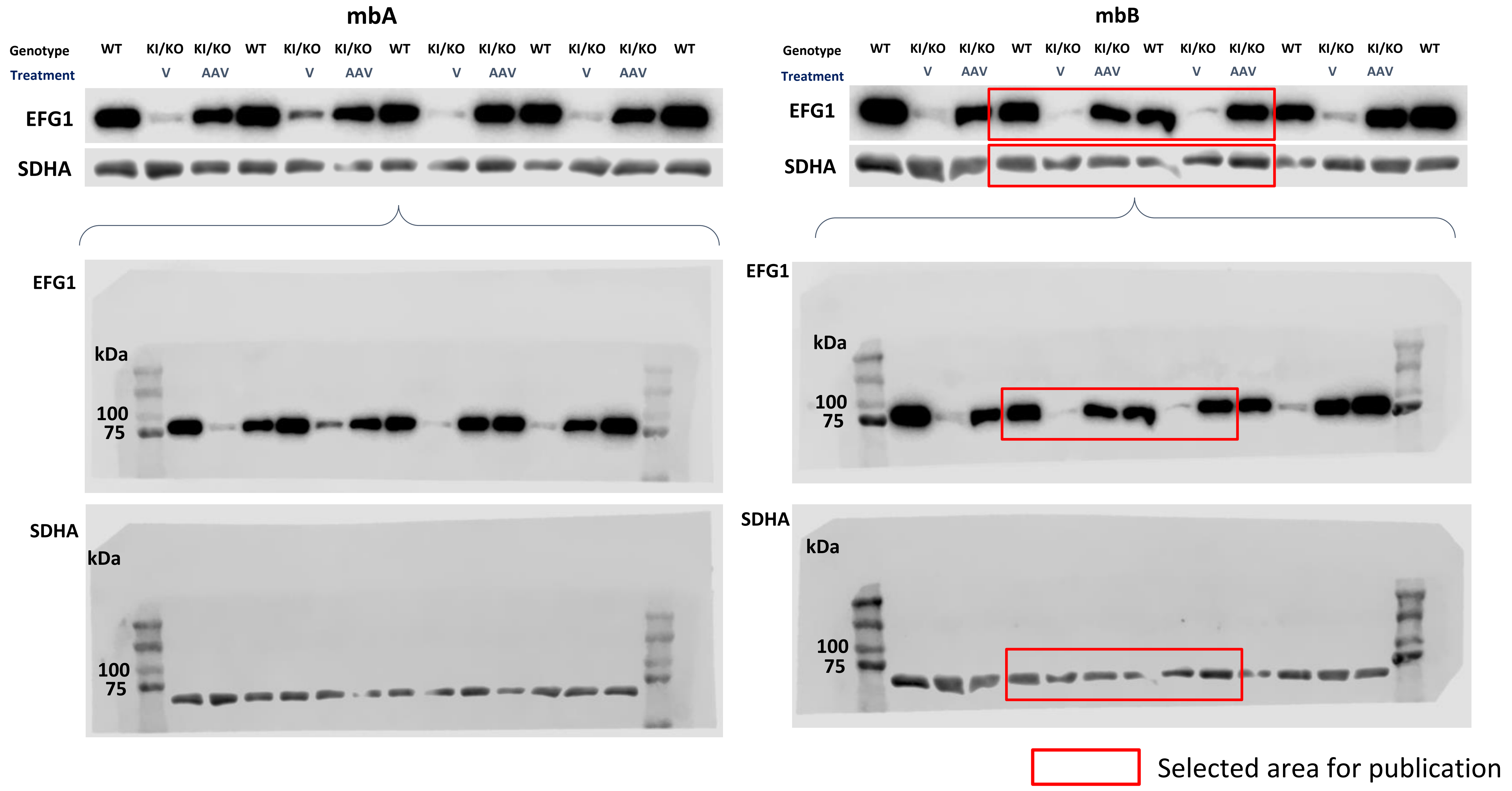

Supplement: Supplementary file 2 — Source data Fig. 1 [file 44321_2026_426_MOESM2_ESM.zip › Figure 1 updated/1C/Fig1C - WB mt hi Females and Males.pdf]
