# Supplementary figures and images for: Systemic delivery of AAV-GFM1 corrects COXPD1 molecular alterations in Gfm1R671C/− mice (part 1 of 2)
Source: EMBO Mol Med. 2026 Apr 17;18(6):2152–79. doi: 10.1038/s44321-026-00426-4 (PMC13269562; doi:10.1038/s44321-026-00426-4)

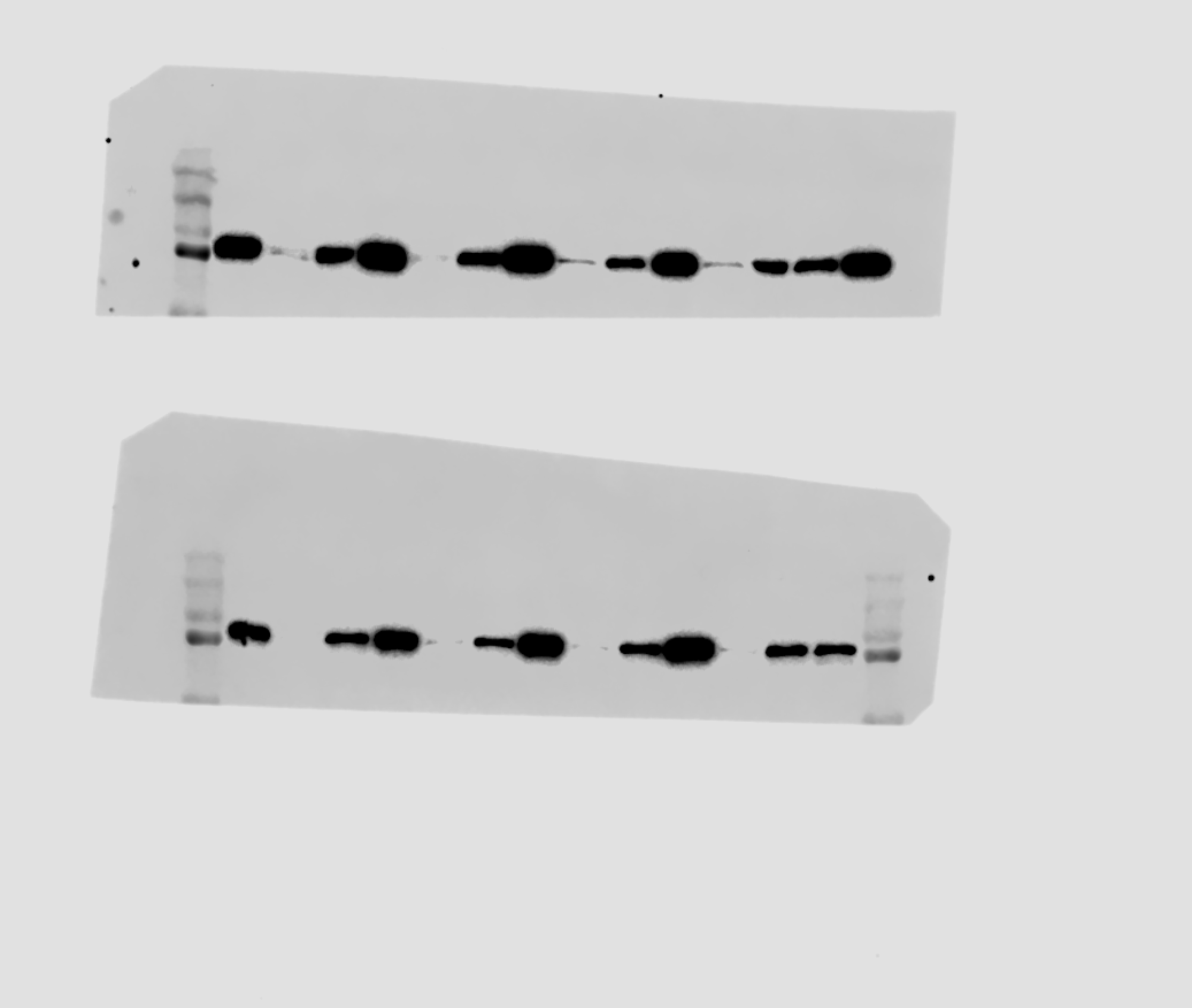

Supplement: Supplementary file 2 — Source data Fig. 1 [file 44321_2026_426_MOESM2_ESM.zip › Figure 1 updated/1C/F1C Females Liver EFG1 a b.tif]

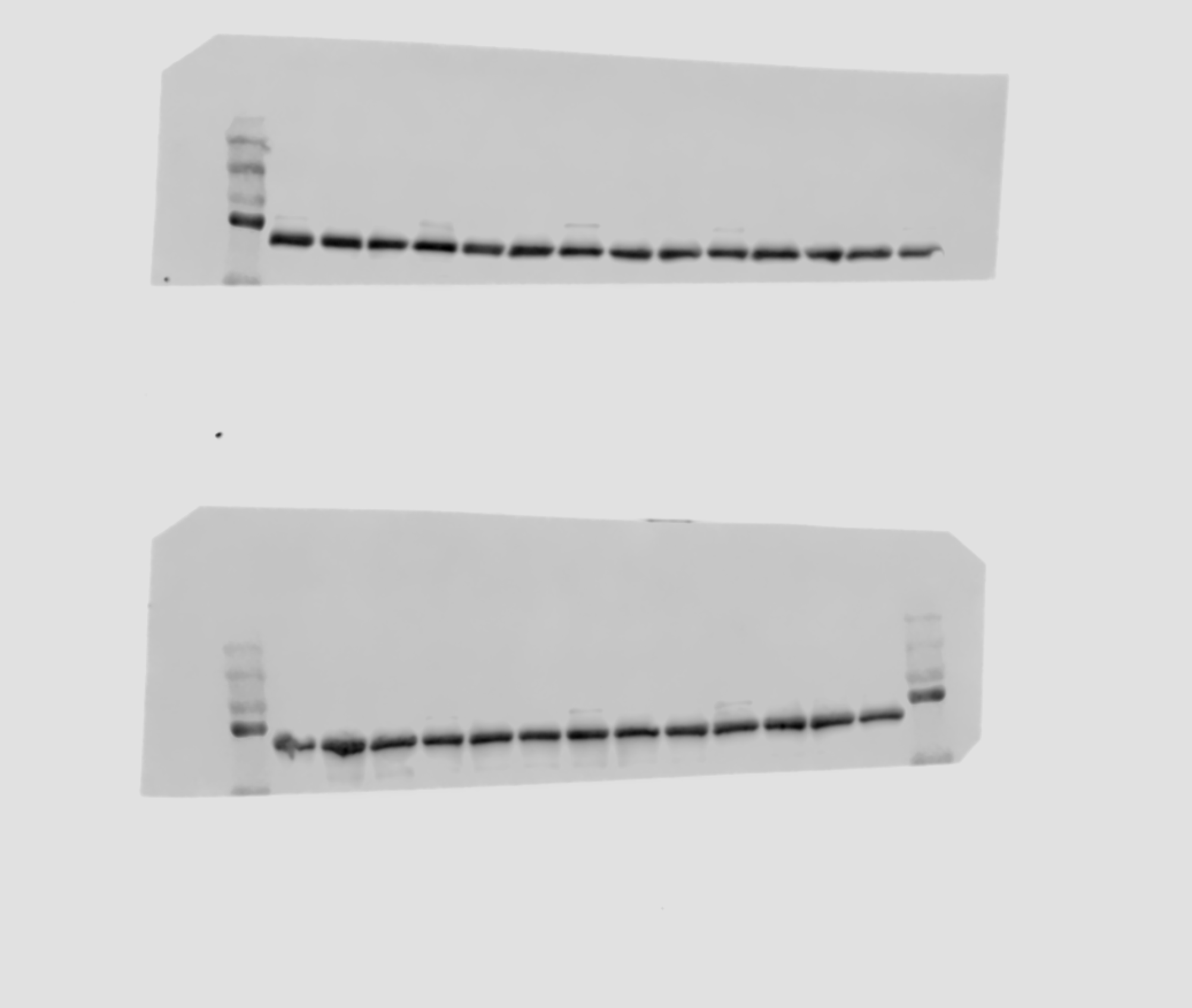

Supplement: Supplementary file 2 — Source data Fig. 1 [file 44321_2026_426_MOESM2_ESM.zip › Figure 1 updated/1C/F1C Females Liver SDHA a b.tif]

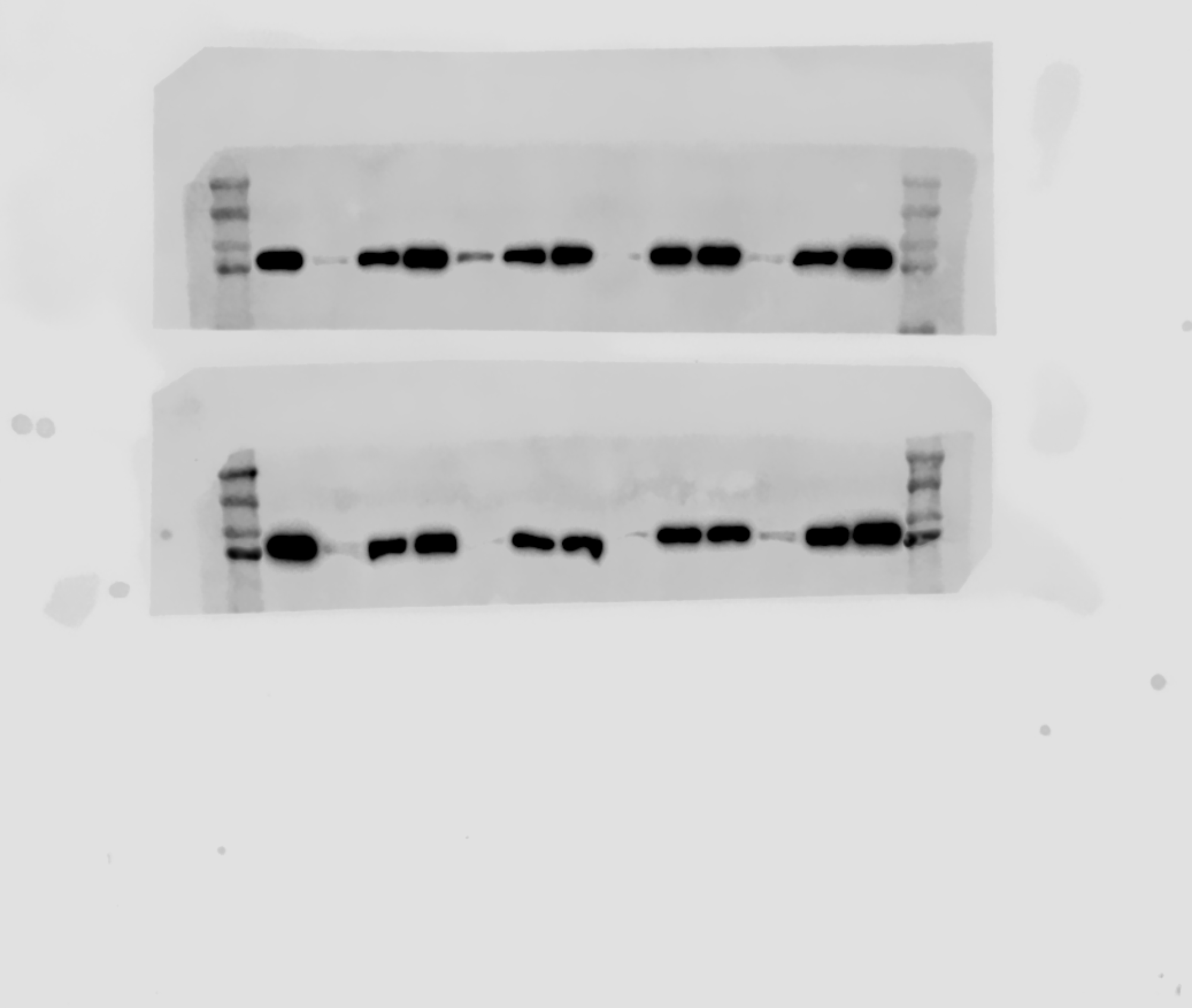

Supplement: Supplementary file 2 — Source data Fig. 1 [file 44321_2026_426_MOESM2_ESM.zip › Figure 1 updated/1C/F1C Males Liver EFG1 a b.tif]

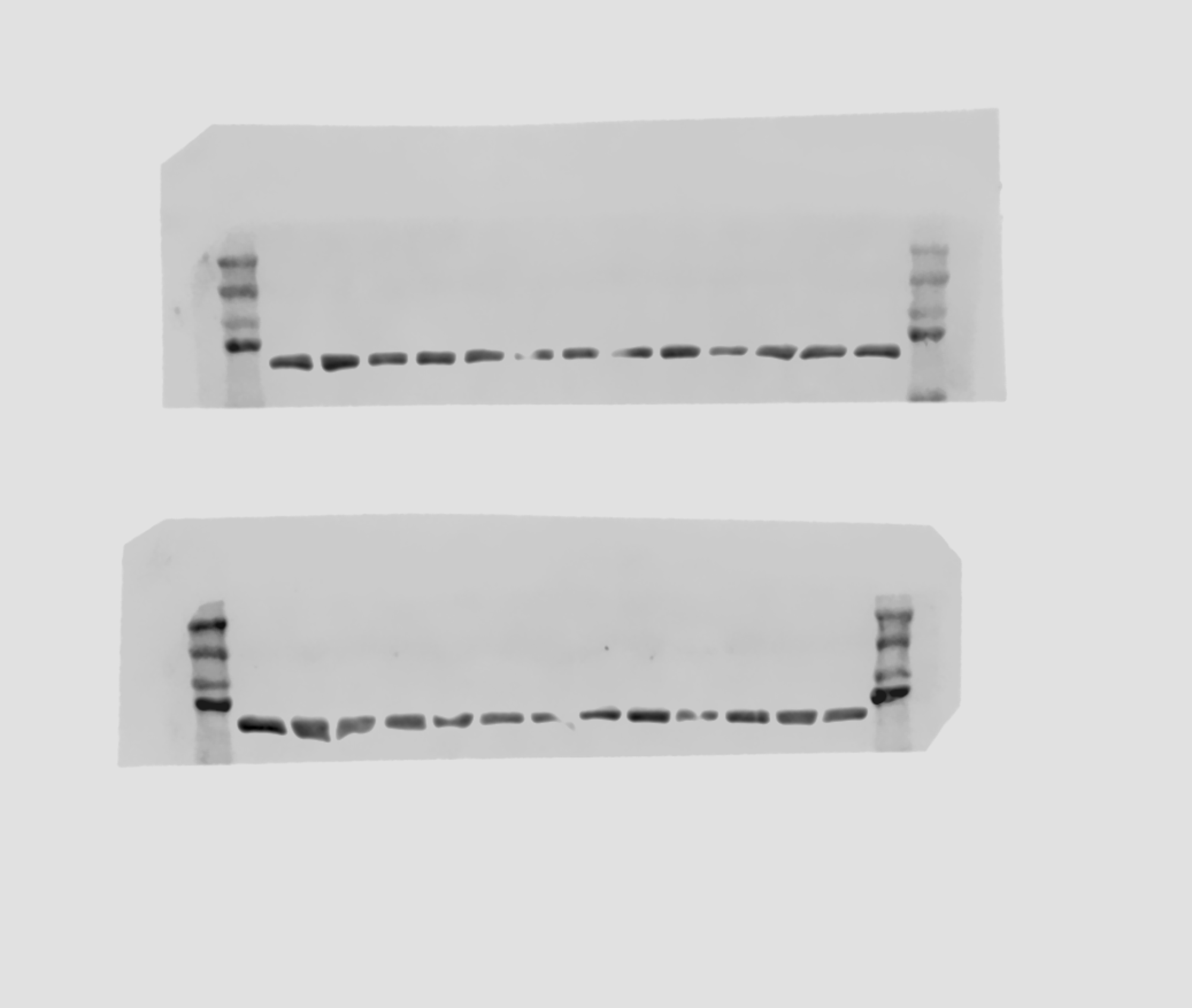

Supplement: Supplementary file 2 — Source data Fig. 1 [file 44321_2026_426_MOESM2_ESM.zip › Figure 1 updated/1C/F1C Males Liver SDHA a b.tif]

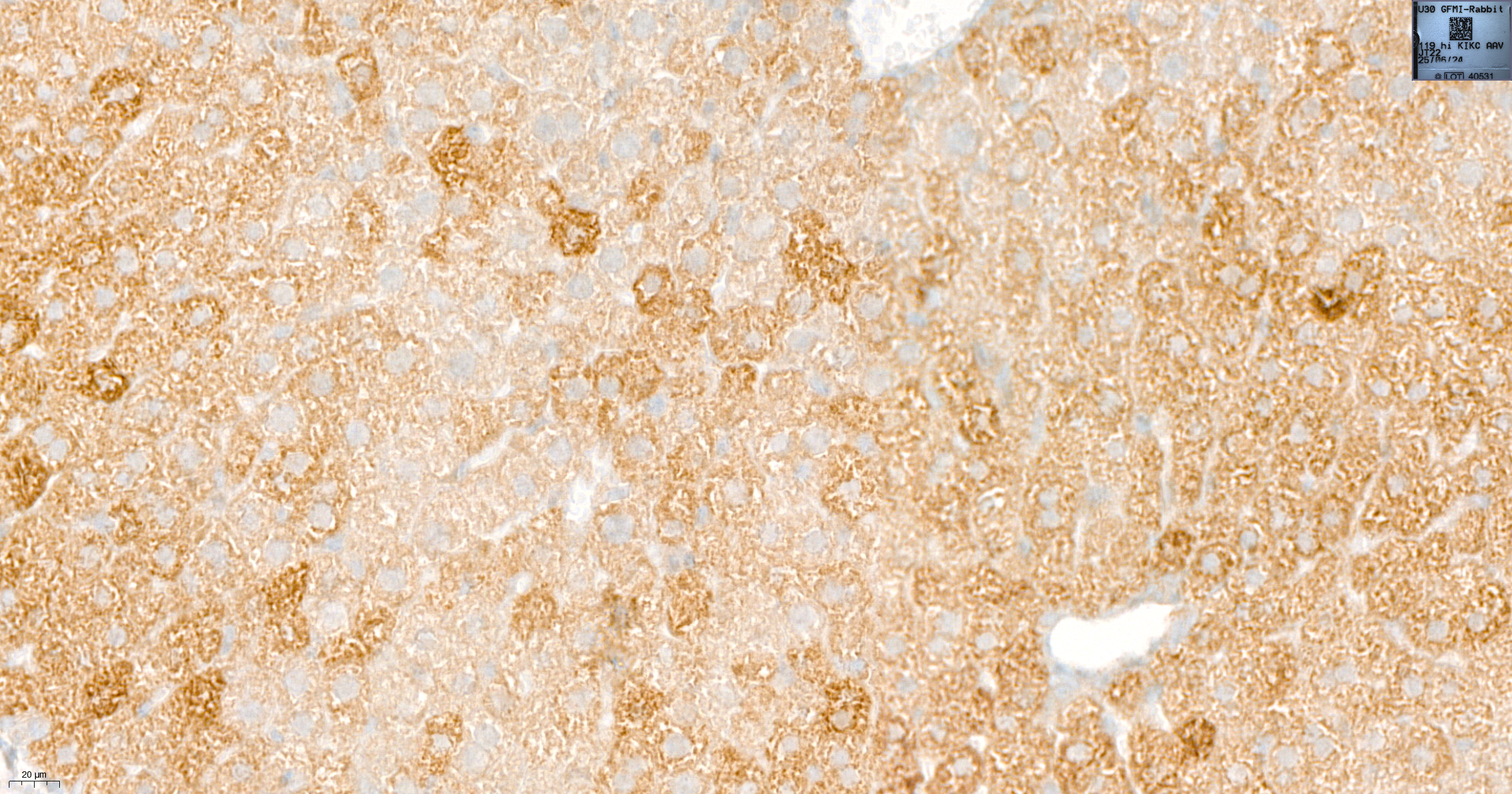

Supplement: Supplementary file 2 — Source data Fig. 1 [file 44321_2026_426_MOESM2_ESM.zip › Figure 1 updated/1E/Female KIKO AAV - 119 hi KIKO AAV_40.0x IHC EFG1 a.tif]

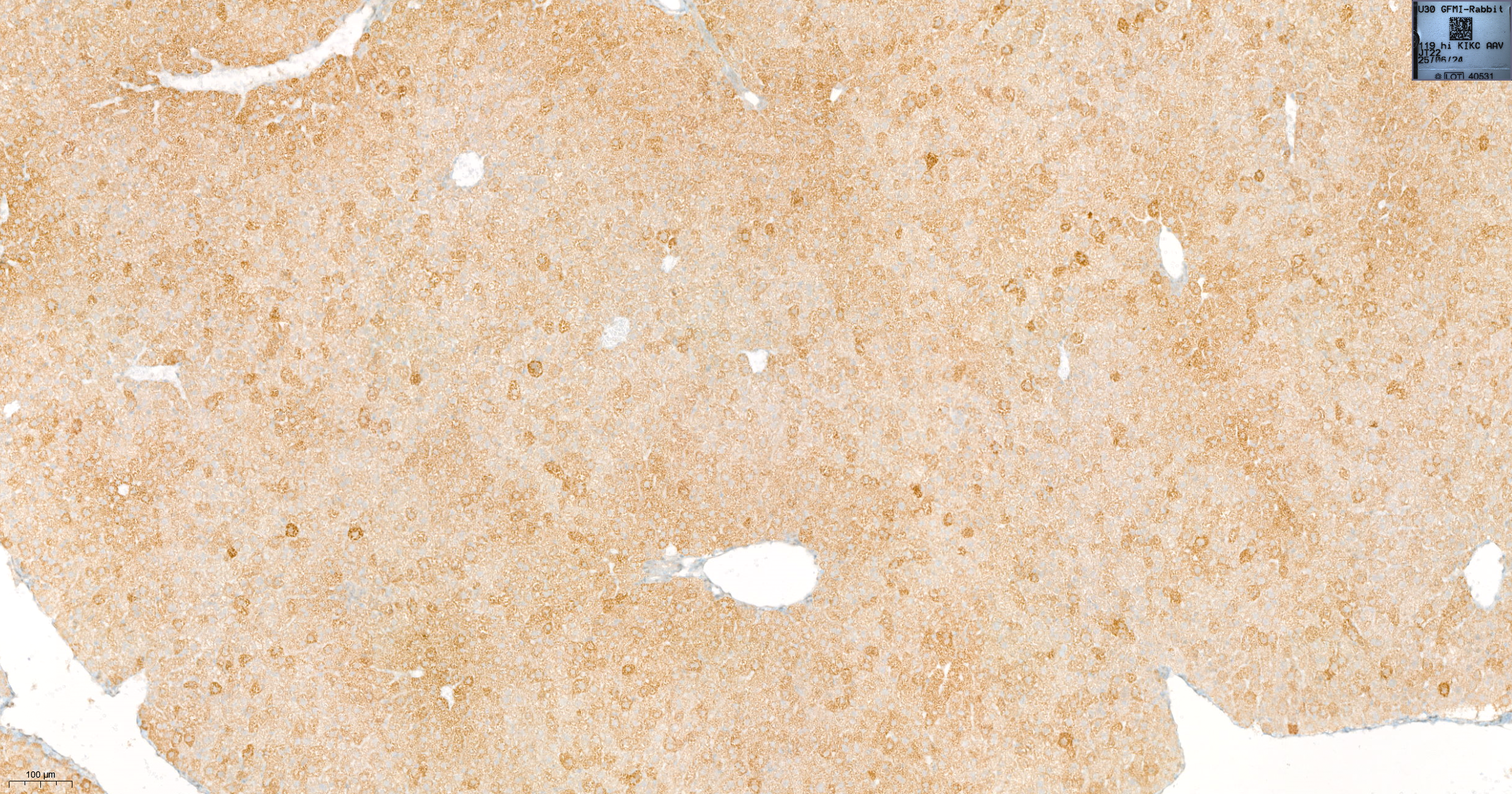

Supplement: Supplementary file 2 — Source data Fig. 1 [file 44321_2026_426_MOESM2_ESM.zip › Figure 1 updated/1E/Female KIKO AAV -119 hi KIKO AAV_10.0x IHC EFG1 a.tif]

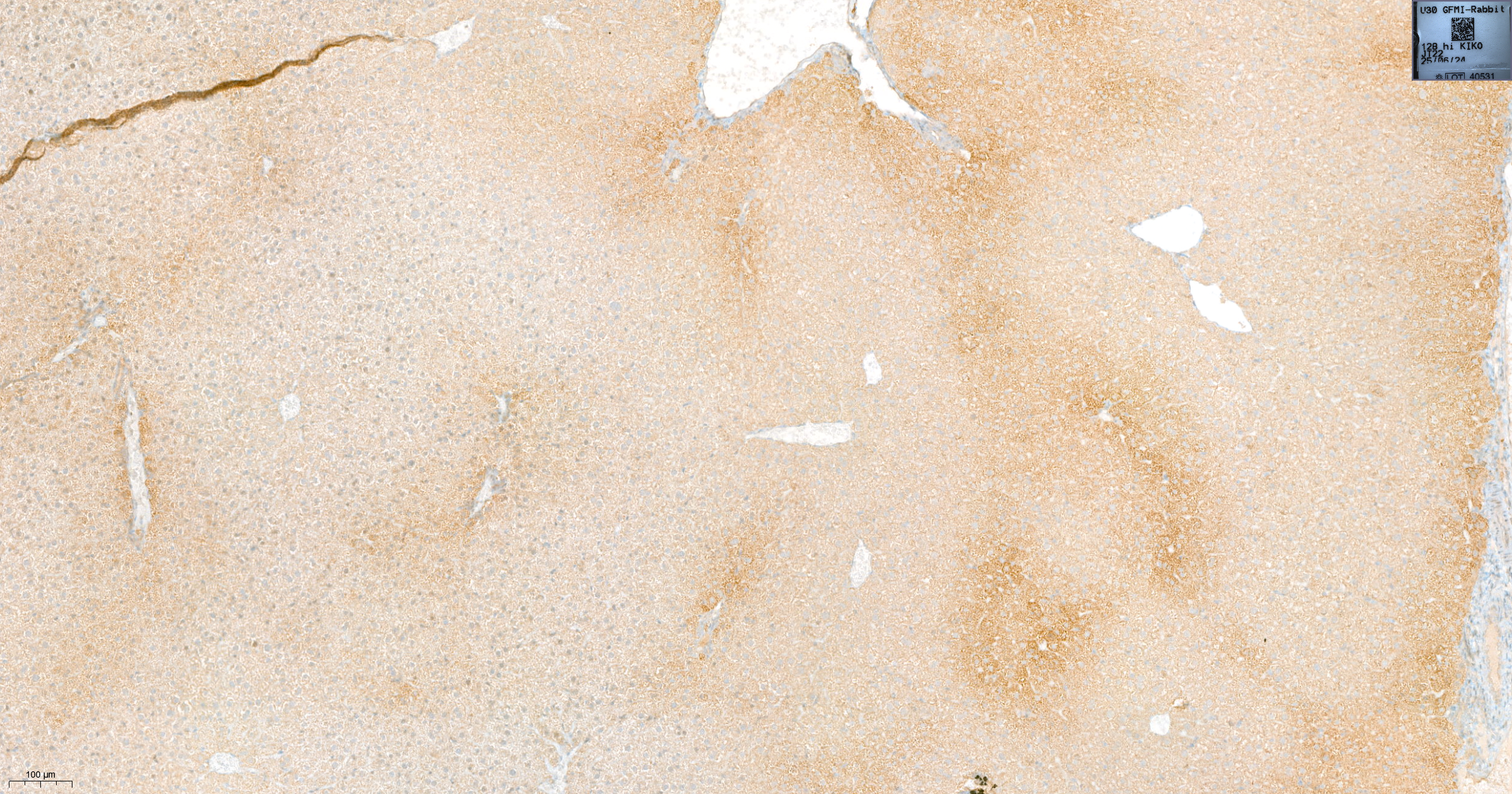

Supplement: Supplementary file 2 — Source data Fig. 1 [file 44321_2026_426_MOESM2_ESM.zip › Figure 1 updated/1E/Female KIKO V -128 hi KIKO_10.0x IHC EFG1 b.tif]

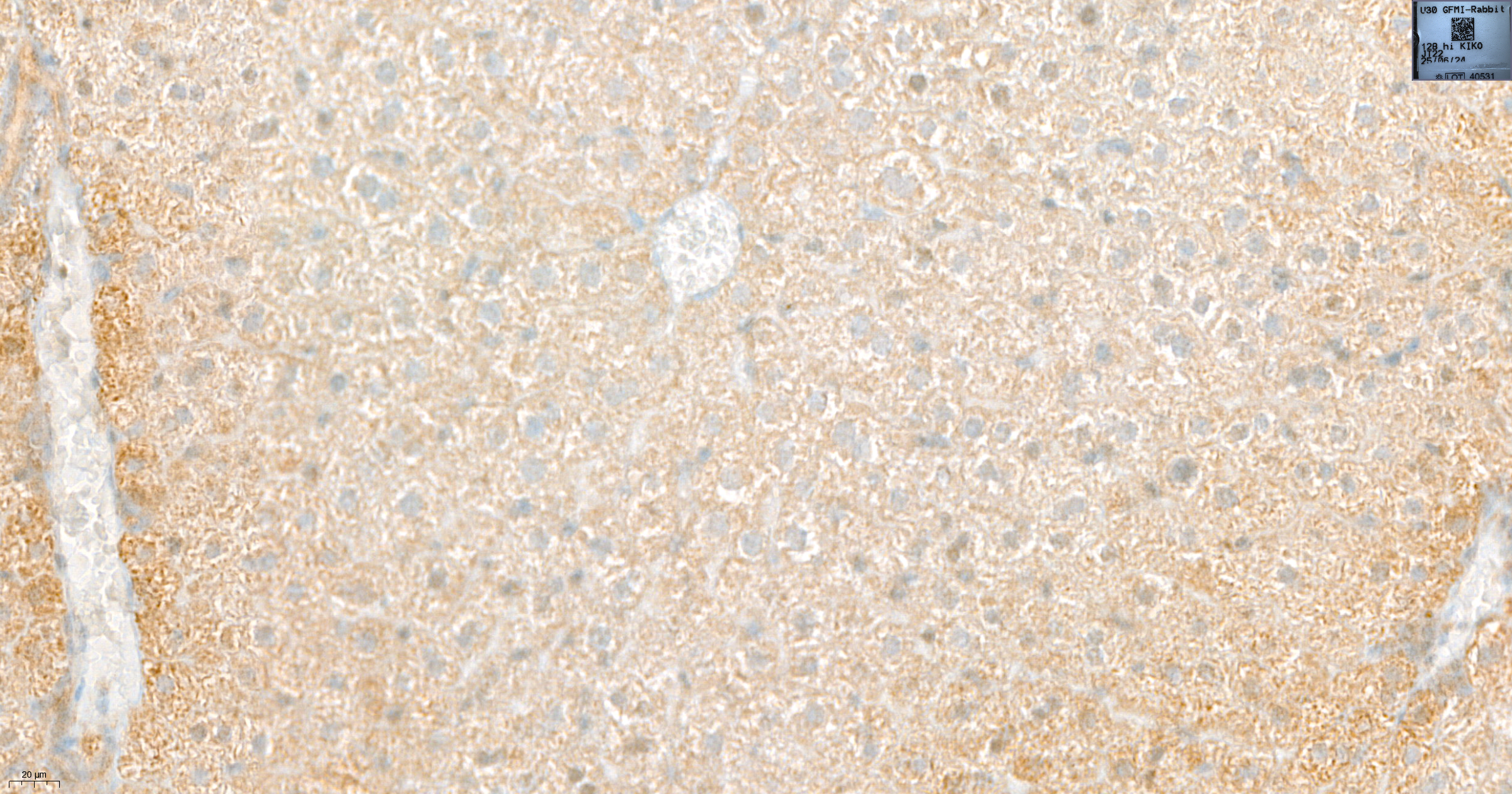

Supplement: Supplementary file 2 — Source data Fig. 1 [file 44321_2026_426_MOESM2_ESM.zip › Figure 1 updated/1E/Female KIKO V -128 hi KIKO_40.0x IHC EFG1 a.tif]

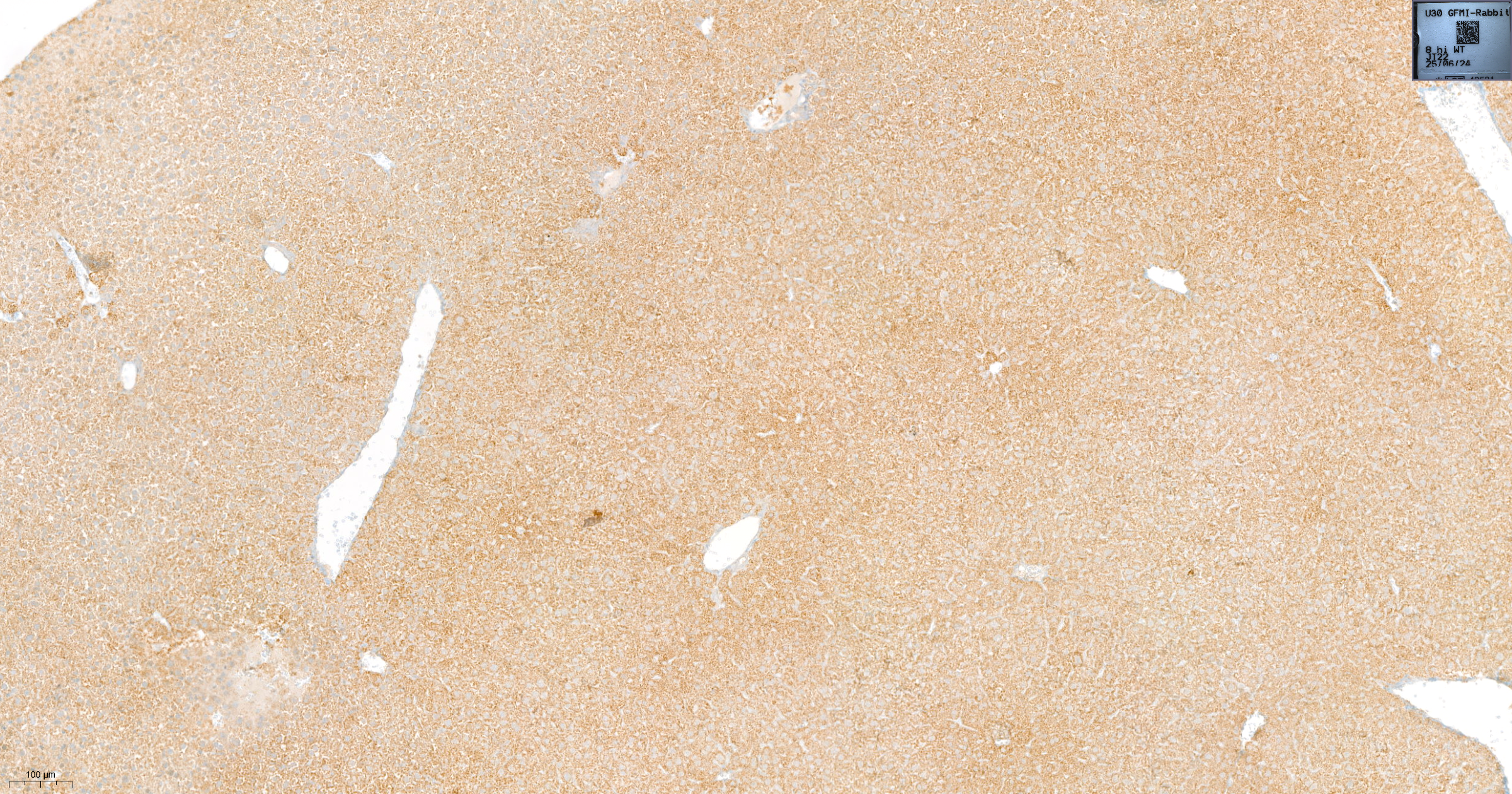

Supplement: Supplementary file 2 — Source data Fig. 1 [file 44321_2026_426_MOESM2_ESM.zip › Figure 1 updated/1E/Female WT - 8 hi WT_10.0x IHC EFG1 a.tif]

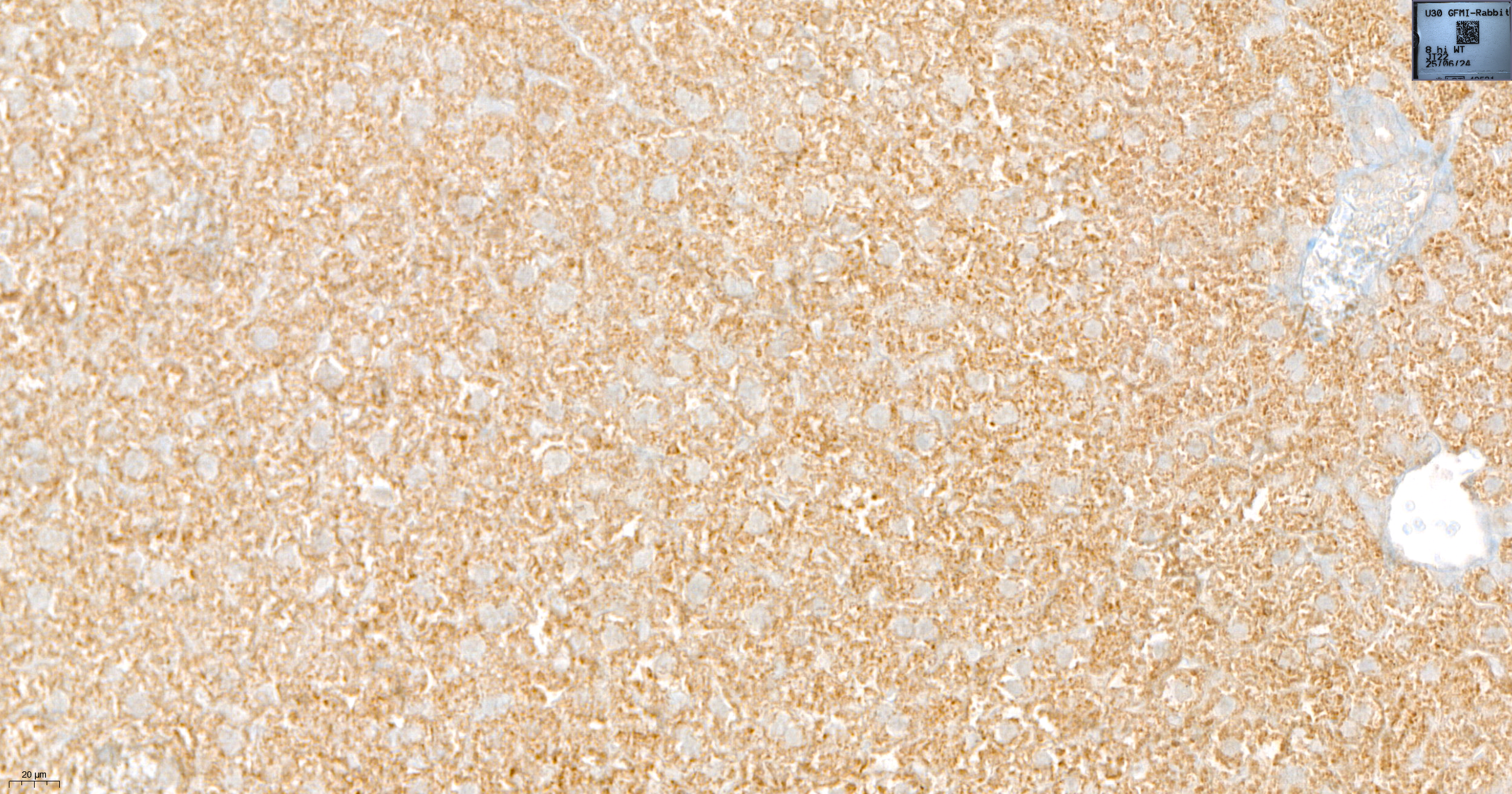

Supplement: Supplementary file 2 — Source data Fig. 1 [file 44321_2026_426_MOESM2_ESM.zip › Figure 1 updated/1E/Female WT - 8 hi WT_40.0x IHC EFG1 a.tif]

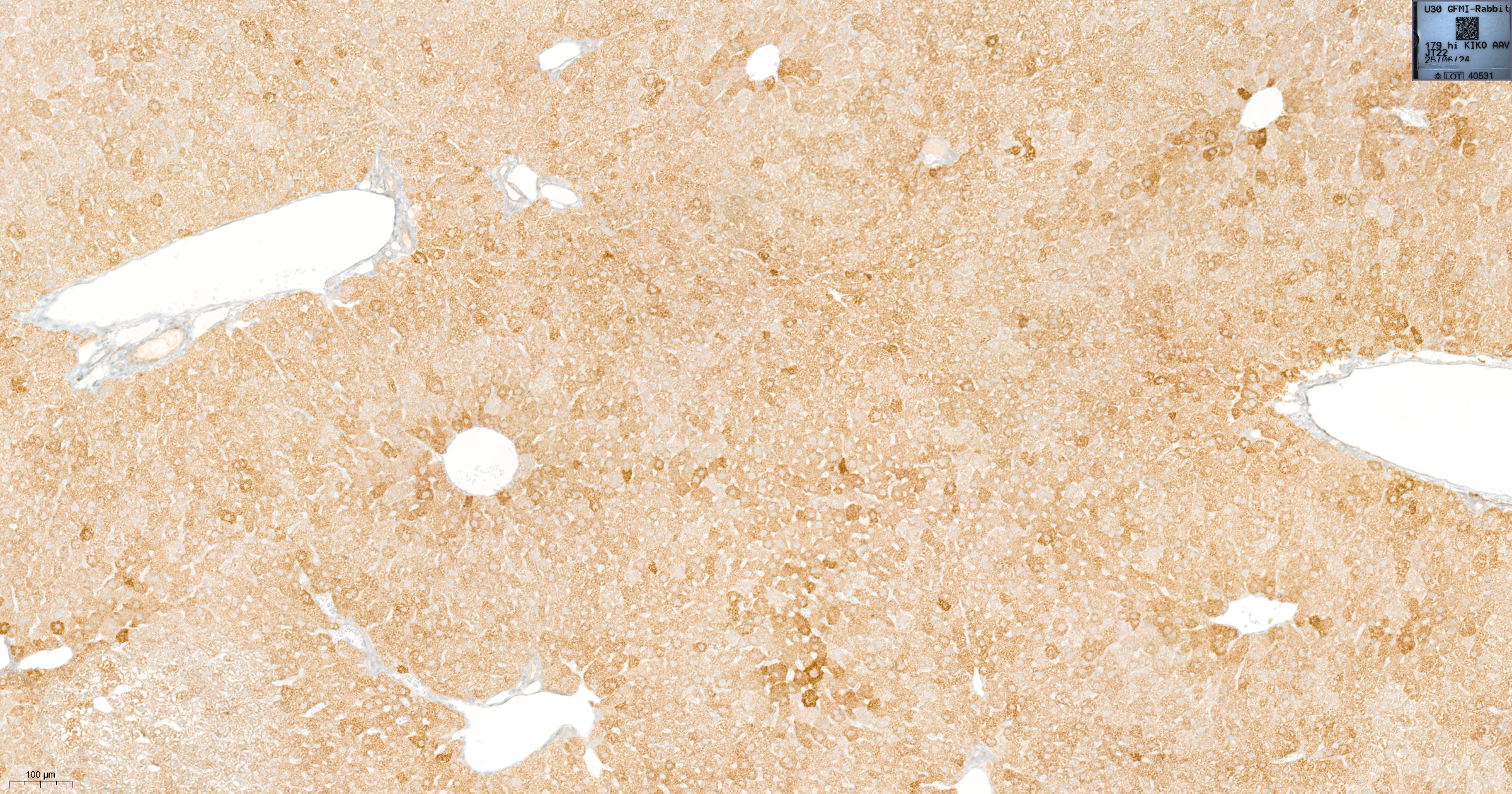

Supplement: Supplementary file 2 — Source data Fig. 1 [file 44321_2026_426_MOESM2_ESM.zip › Figure 1 updated/1E/Male KIKO AAV - 179 hi KIKO AAV_10.0x IHC EFG1 a.tif]

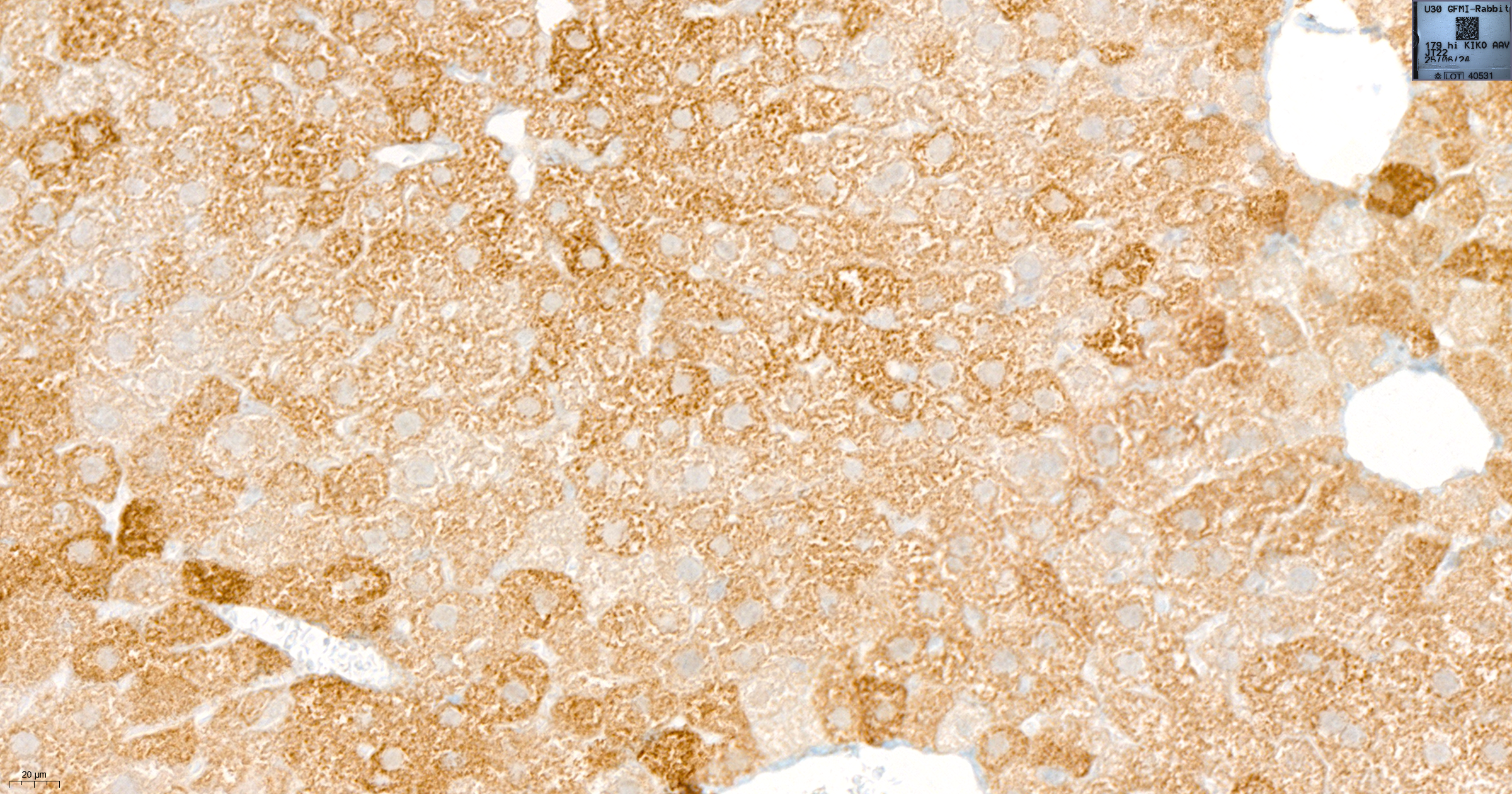

Supplement: Supplementary file 2 — Source data Fig. 1 [file 44321_2026_426_MOESM2_ESM.zip › Figure 1 updated/1E/Male KIKO AAV -179 hi KIKO AAV_40.0x IHC EFG1 b.tif]

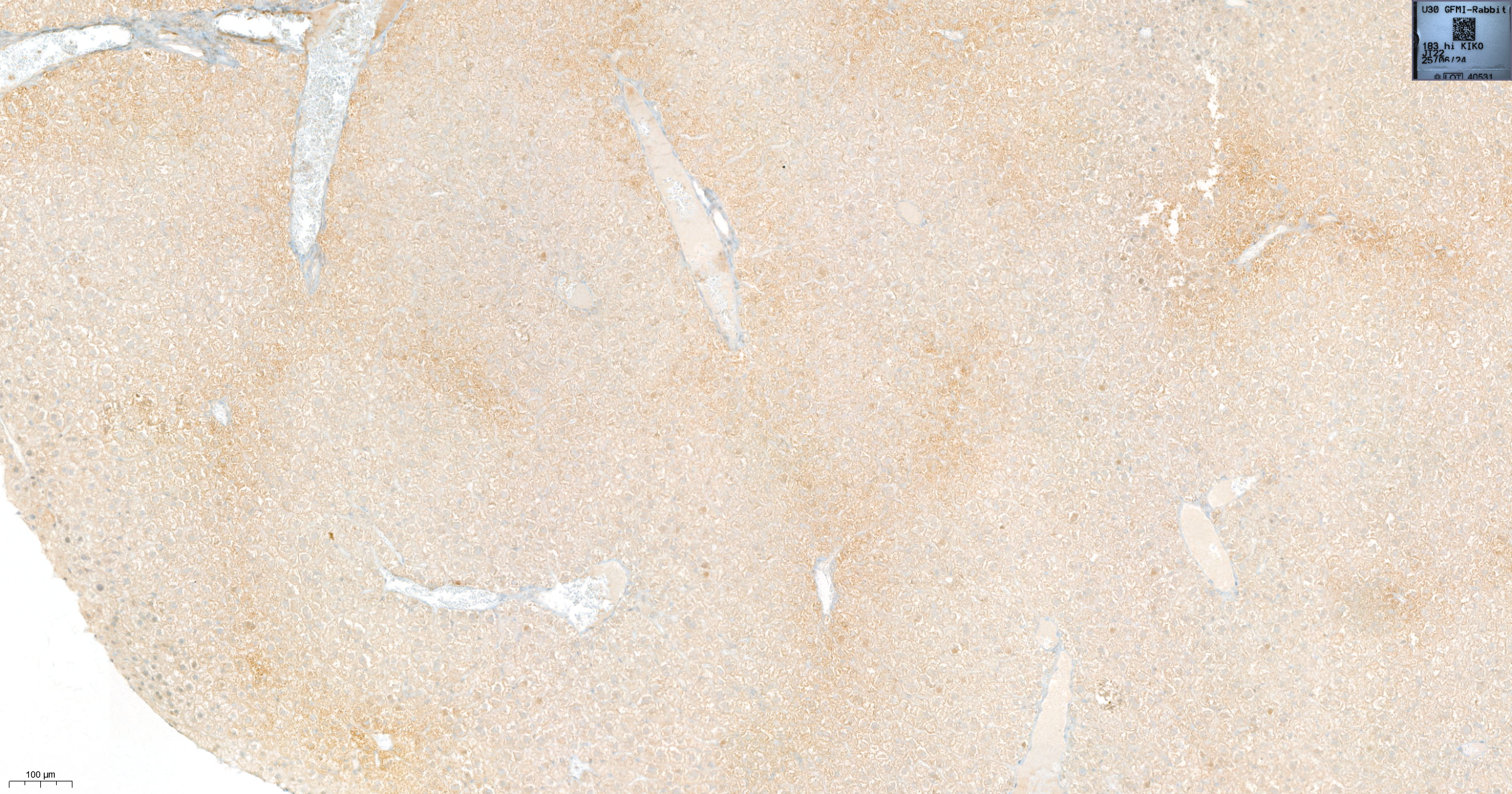

Supplement: Supplementary file 2 — Source data Fig. 1 [file 44321_2026_426_MOESM2_ESM.zip › Figure 1 updated/1E/Male KIKO V - 183 hi KIKO_10.0x IHC EFG1 a.tif]

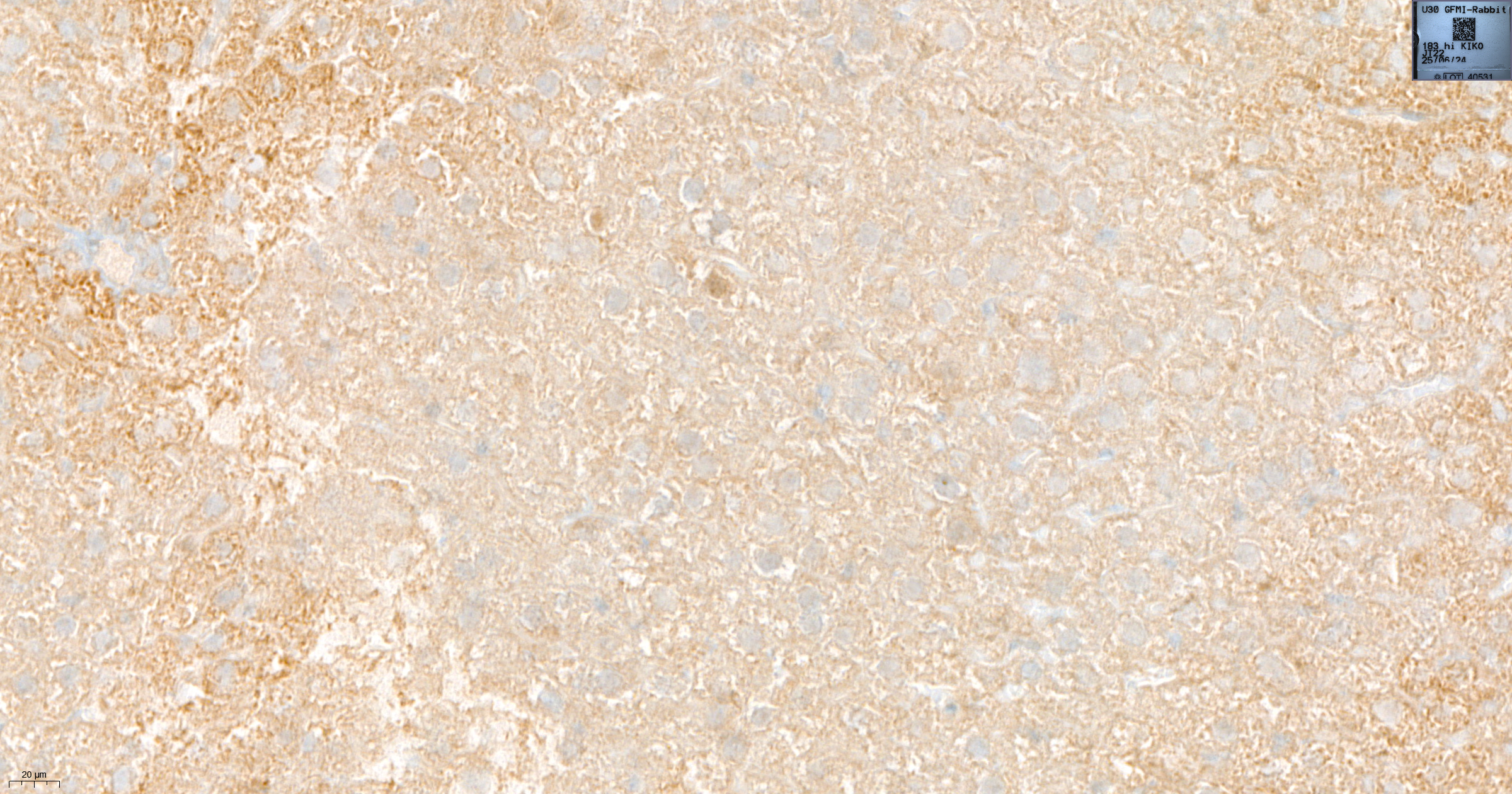

Supplement: Supplementary file 2 — Source data Fig. 1 [file 44321_2026_426_MOESM2_ESM.zip › Figure 1 updated/1E/Male KIKO V -183 hi KIKO_40.0x IHC EFG1 a.tif]

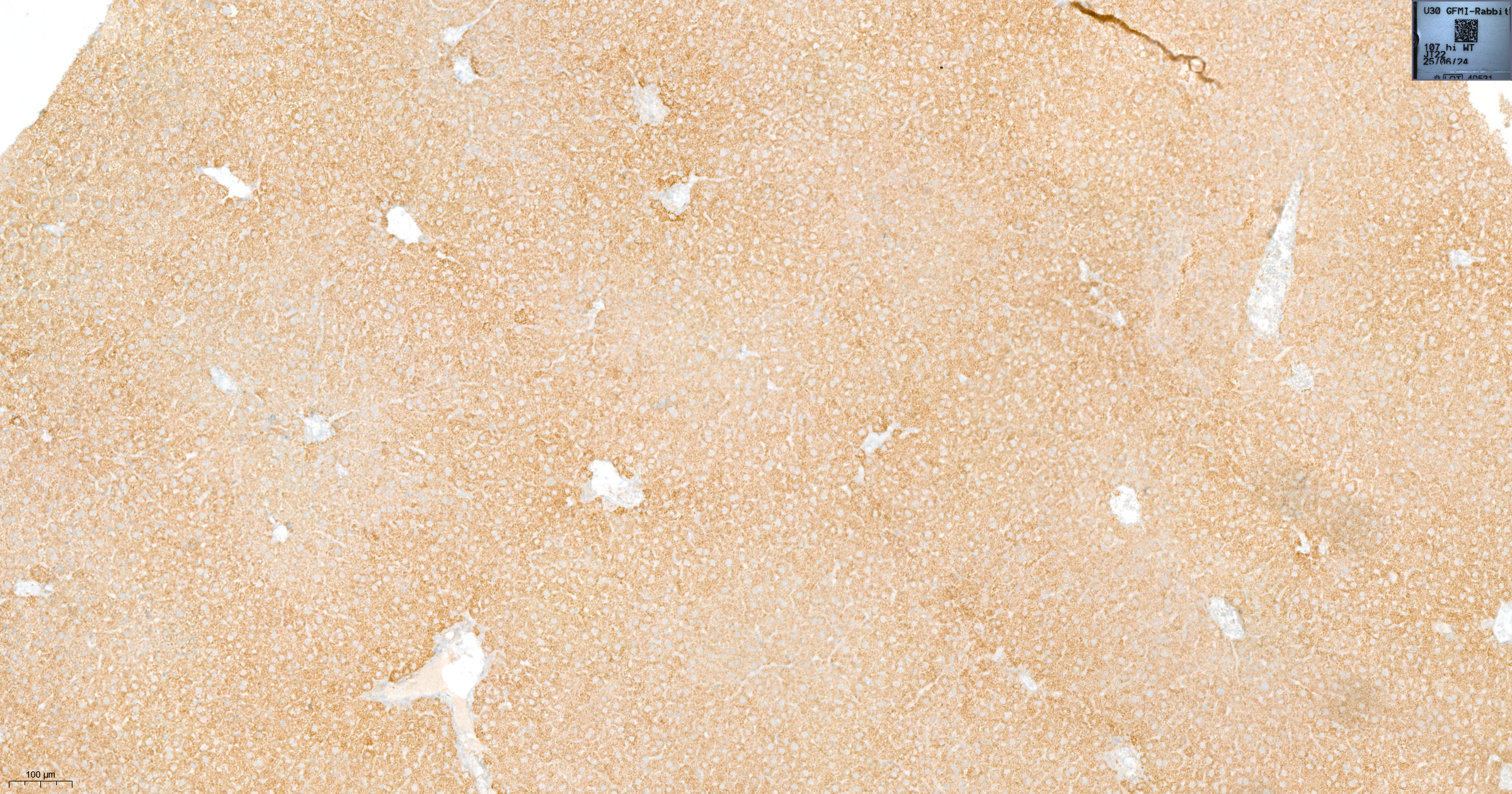

Supplement: Supplementary file 2 — Source data Fig. 1 [file 44321_2026_426_MOESM2_ESM.zip › Figure 1 updated/1E/Male WT - 107 hi WT_10.0x IHC EFG1 b.tif]

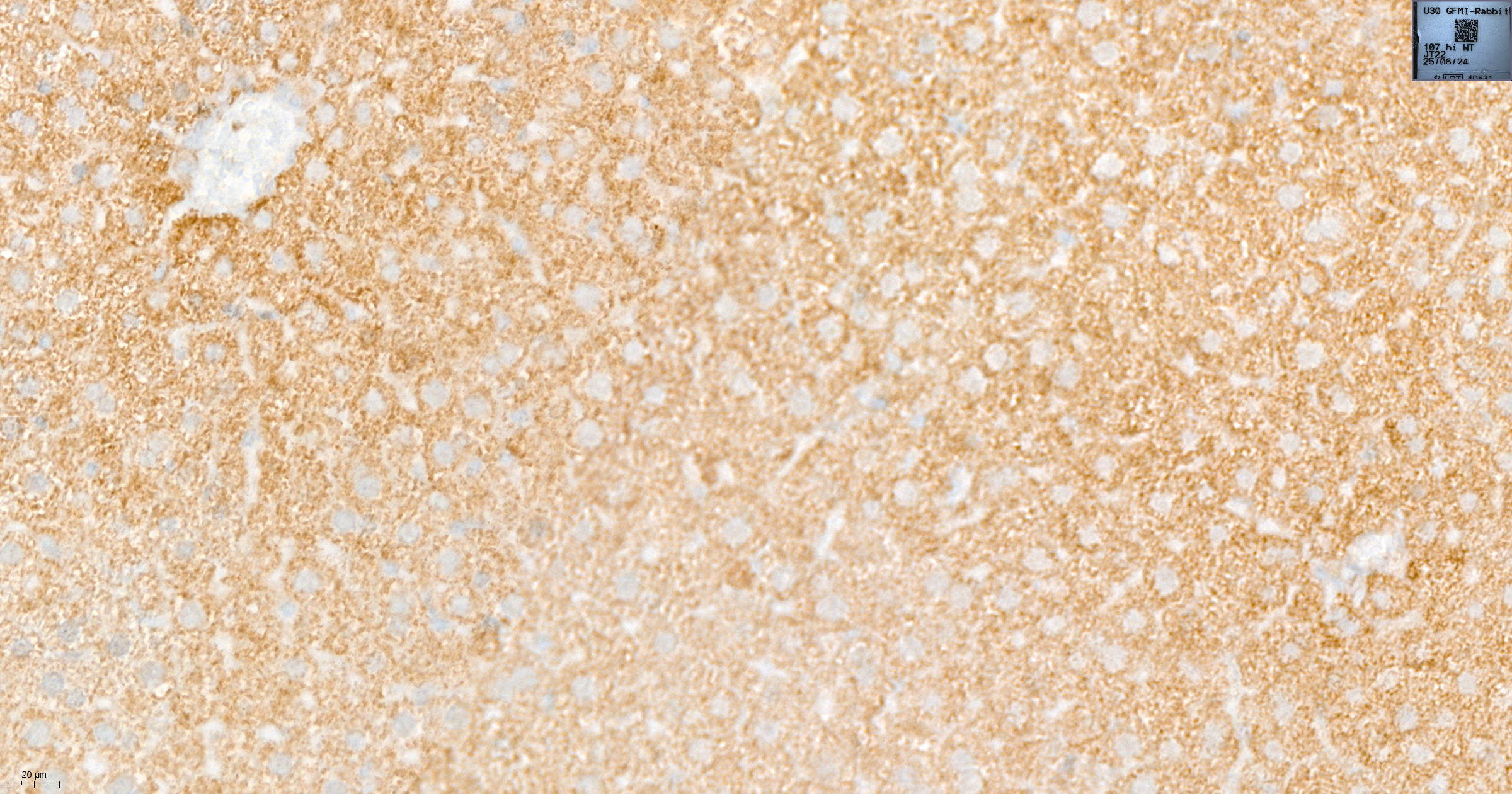

Supplement: Supplementary file 2 — Source data Fig. 1 [file 44321_2026_426_MOESM2_ESM.zip › Figure 1 updated/1E/Male WT - 107 hi WT_40.0x IHC EFG1 b.tif]

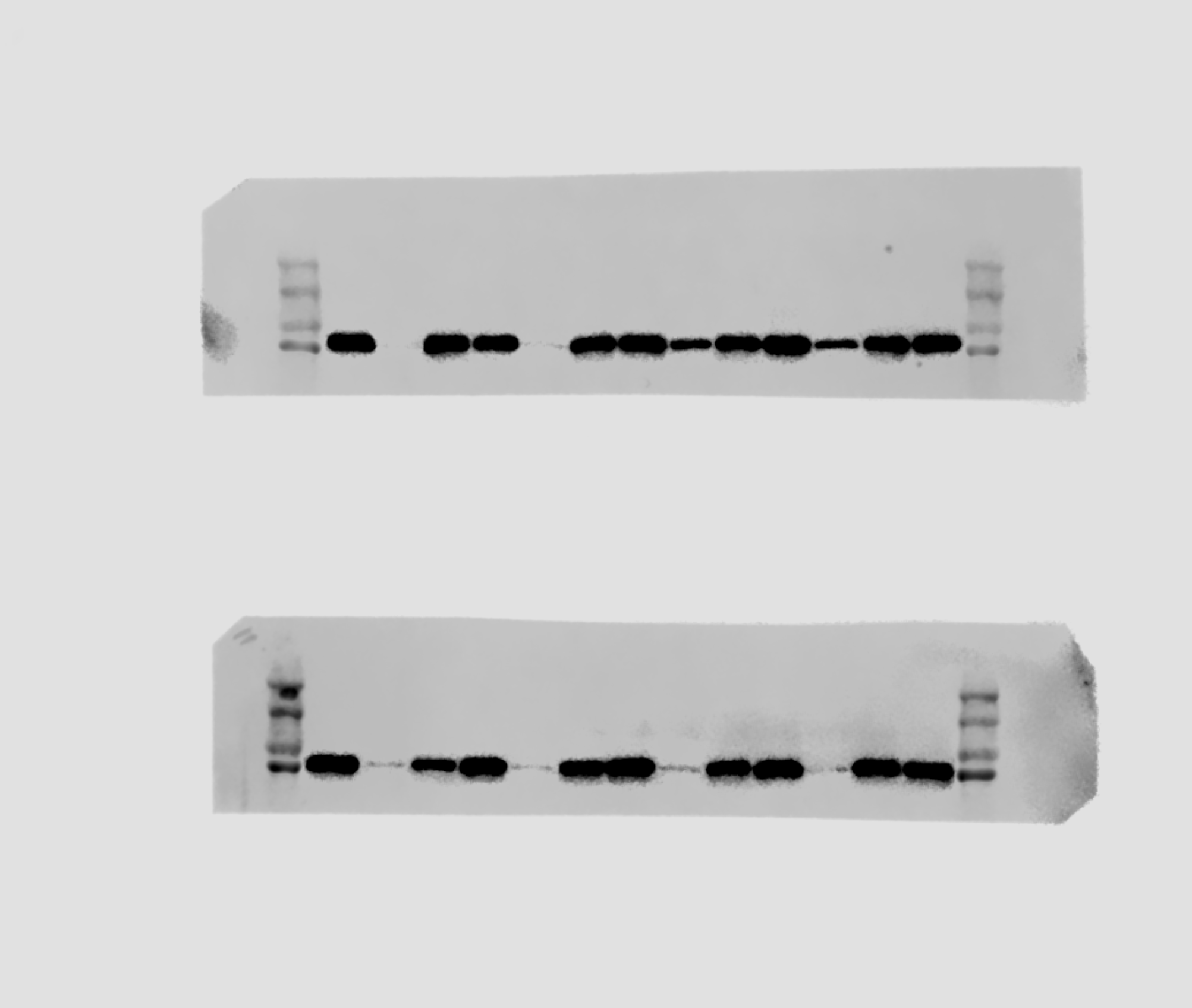

Supplement: Supplementary file 3 — Source data Fig. 2 [file 44321_2026_426_MOESM3_ESM.zip › Figure 2 updated/2C/F2C Females Brain EFG1 a b.tif]

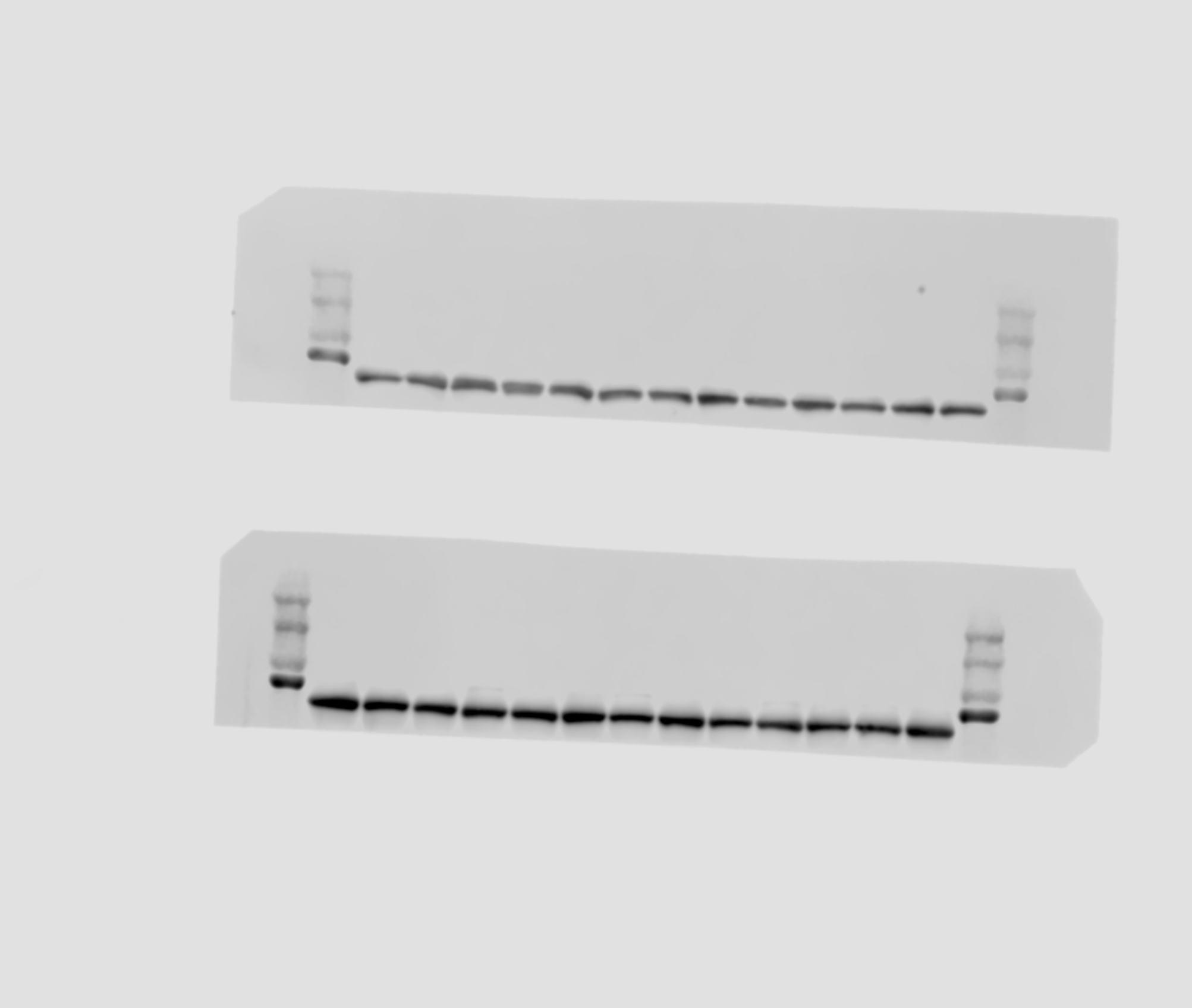

Supplement: Supplementary file 3 — Source data Fig. 2 [file 44321_2026_426_MOESM3_ESM.zip › Figure 2 updated/2C/F2C Females Brain SDHA a b.tif]

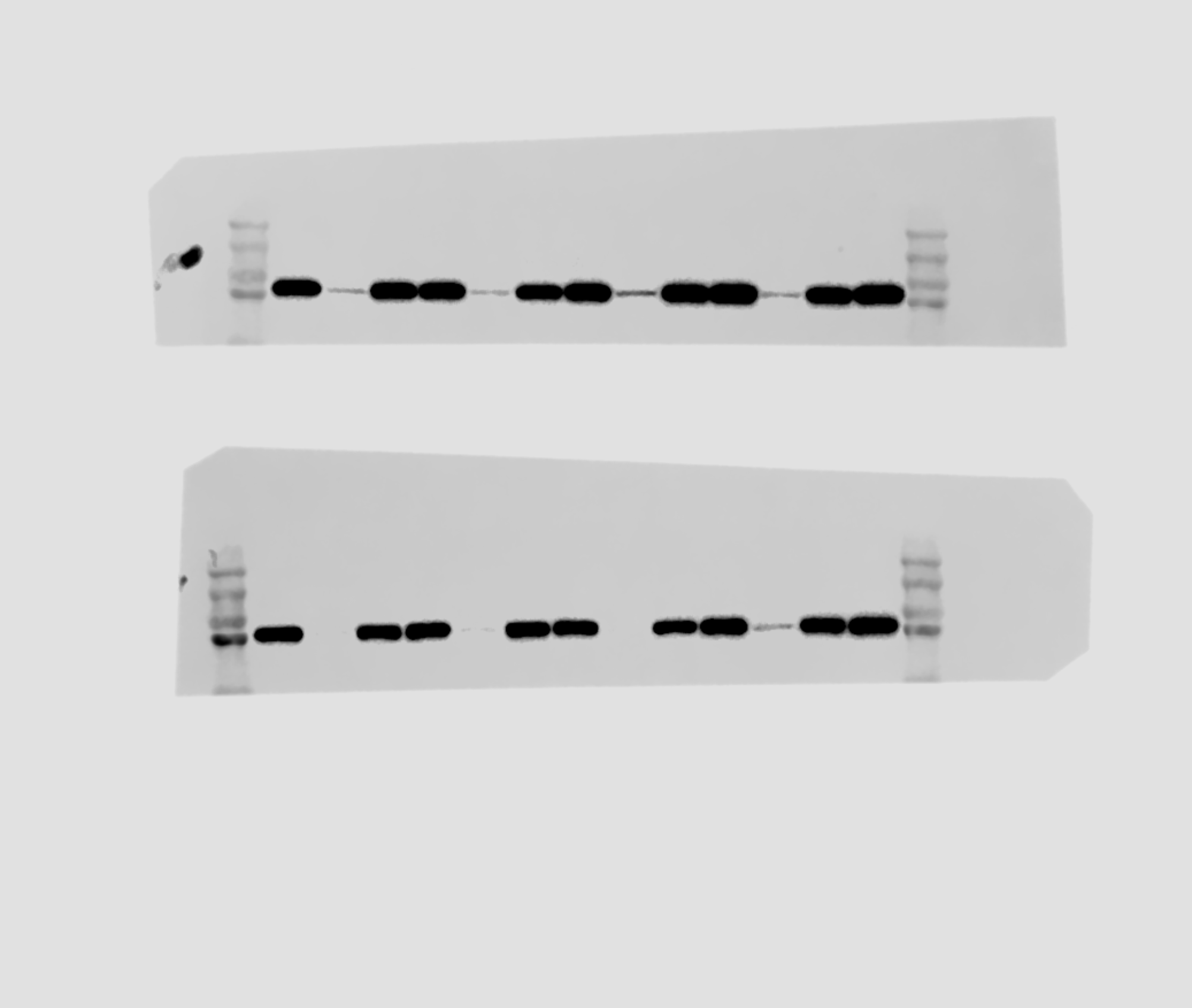

Supplement: Supplementary file 3 — Source data Fig. 2 [file 44321_2026_426_MOESM3_ESM.zip › Figure 2 updated/2C/F2C Males Brain EFG1 a b.tif]

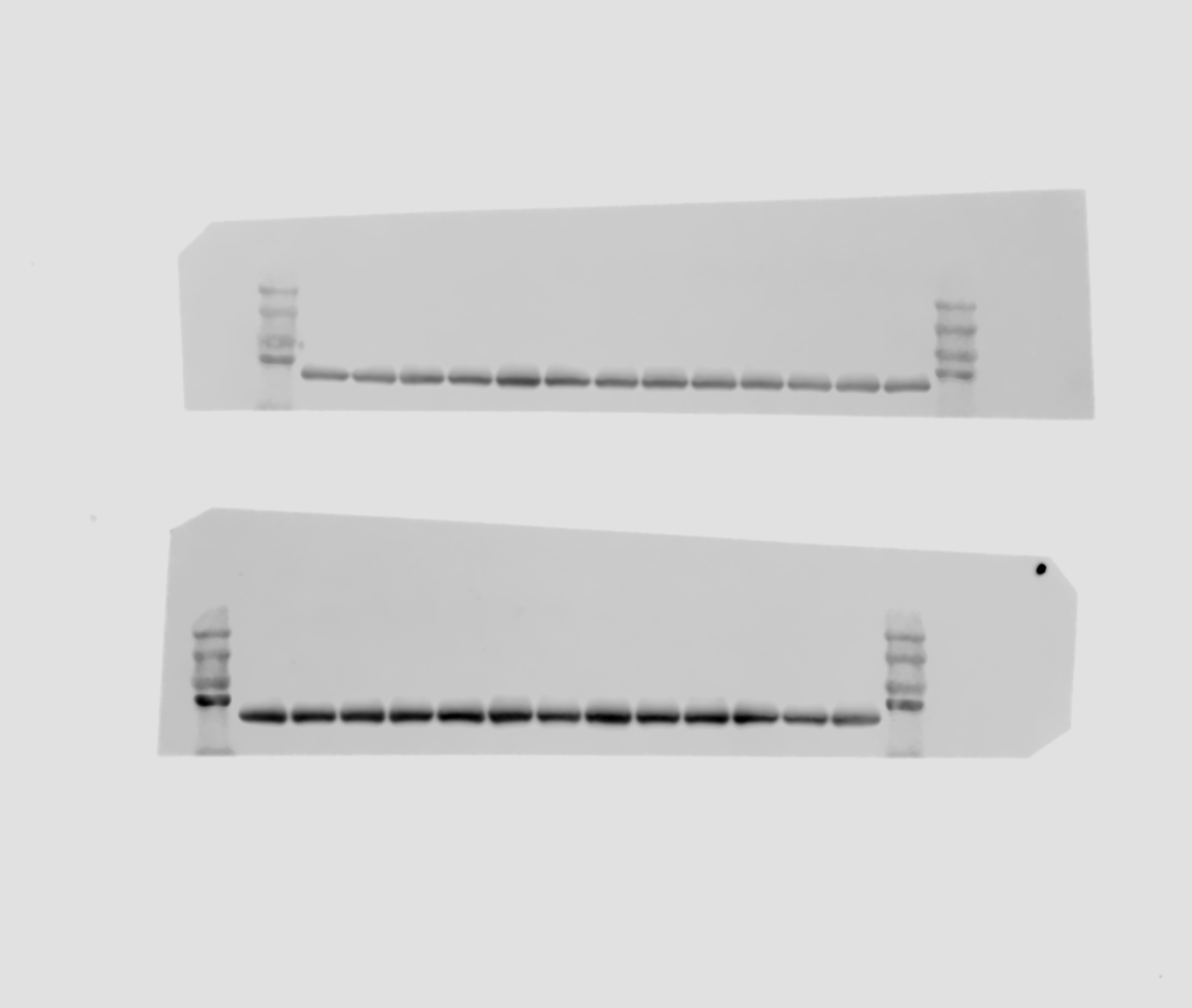

Supplement: Supplementary file 3 — Source data Fig. 2 [file 44321_2026_426_MOESM3_ESM.zip › Figure 2 updated/2C/F2C Males Brain SDHA a b.tif]

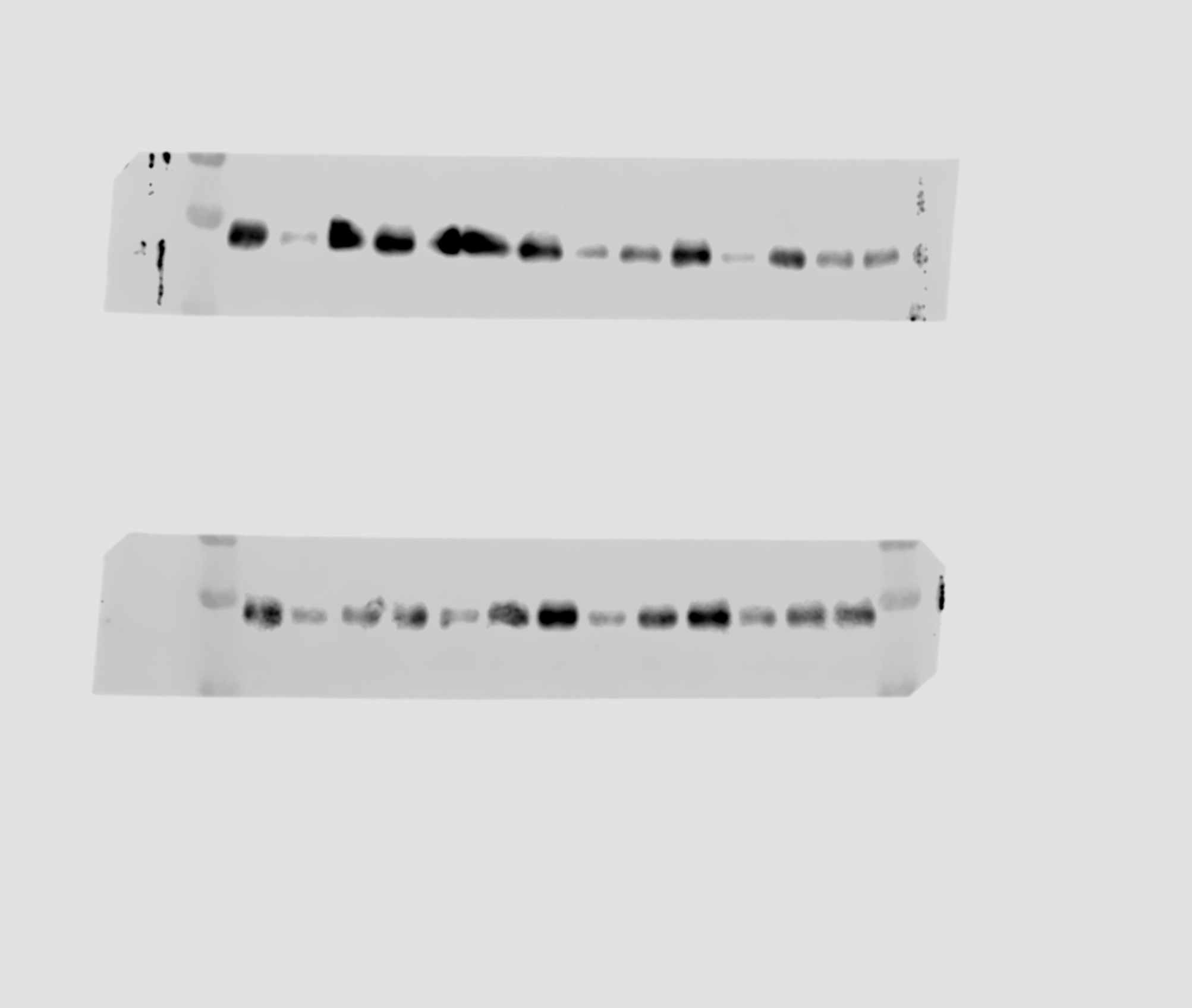

Supplement: Supplementary file 4 — Source data Fig. 3 [file 44321_2026_426_MOESM4_ESM.zip › Figure 3 updated/3A/F3A Females Liver COX1 a b.tif]

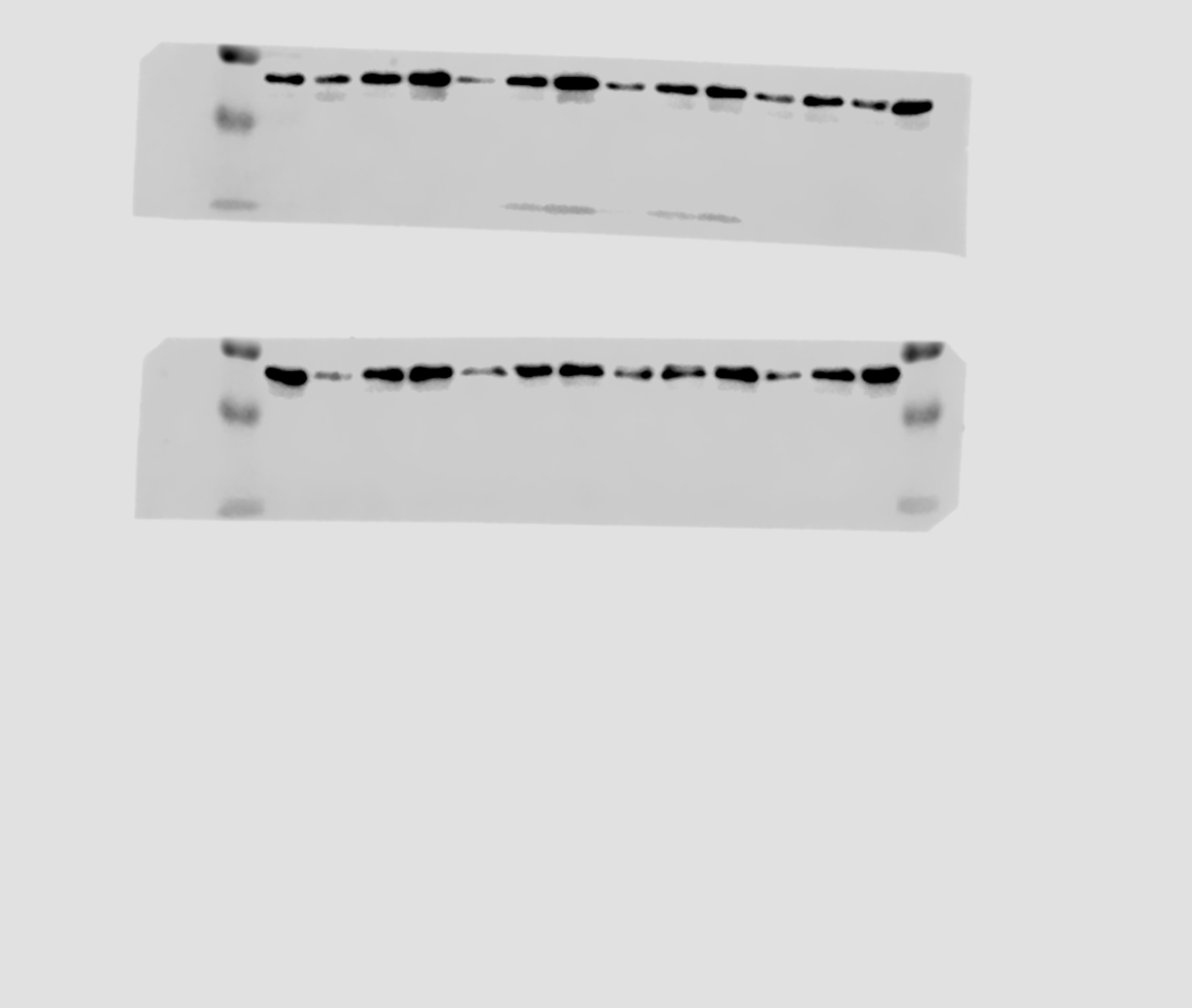

Supplement: Supplementary file 4 — Source data Fig. 3 [file 44321_2026_426_MOESM4_ESM.zip › Figure 3 updated/3A/F3A Females Liver COX2 c d.tif]

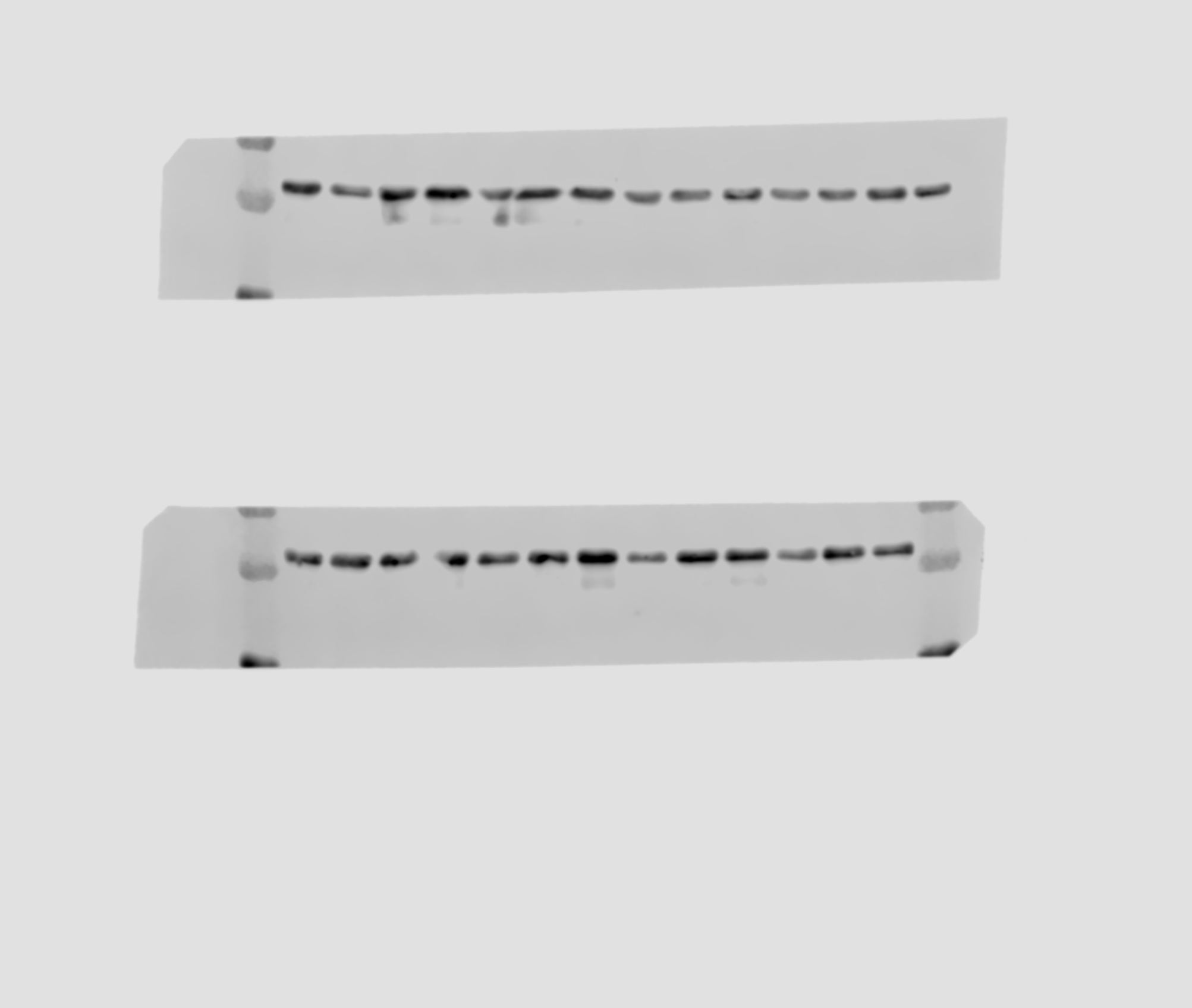

Supplement: Supplementary file 4 — Source data Fig. 3 [file 44321_2026_426_MOESM4_ESM.zip › Figure 3 updated/3A/F3A Females Liver NDUFA10 a b.tif]

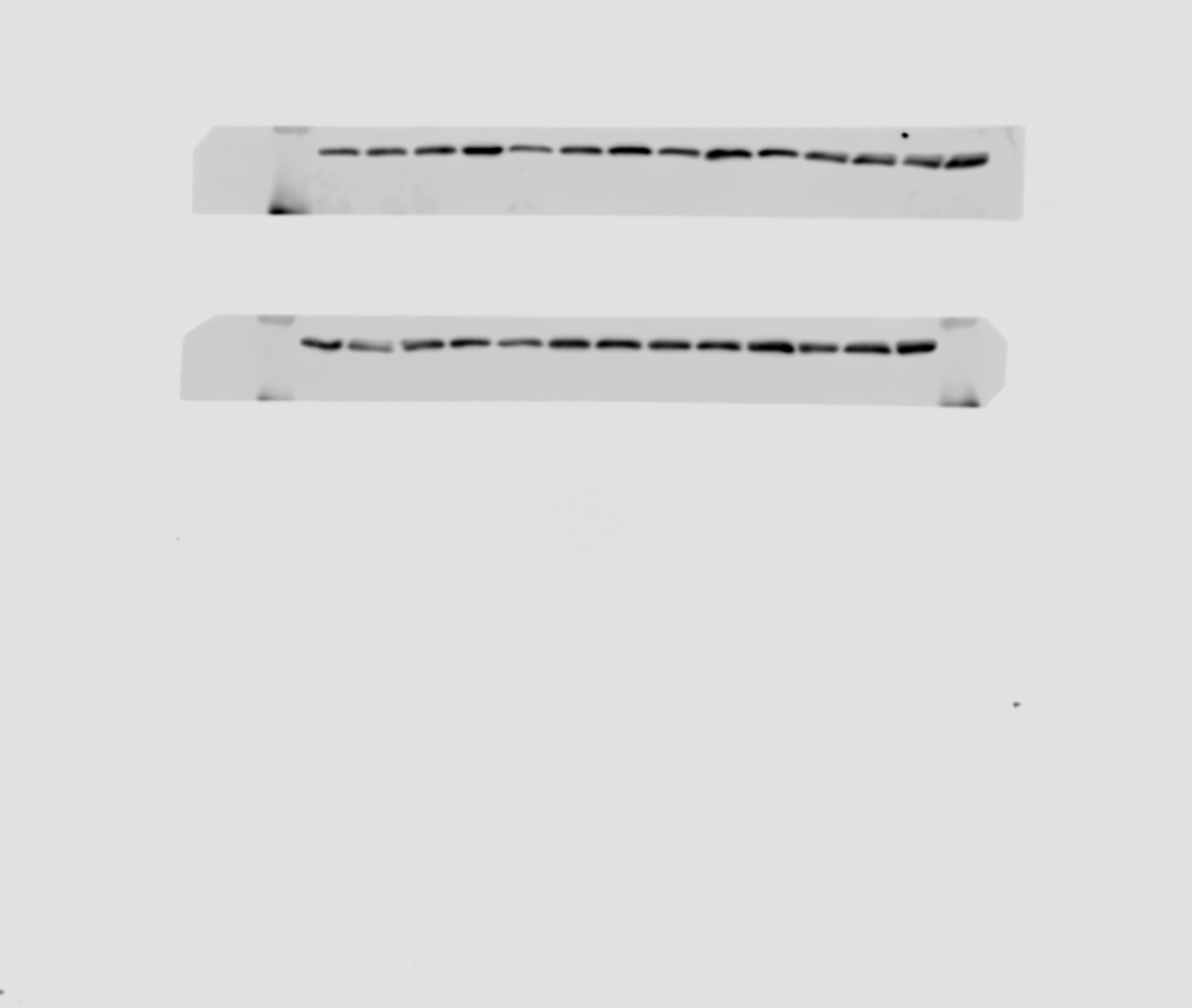

Supplement: Supplementary file 4 — Source data Fig. 3 [file 44321_2026_426_MOESM4_ESM.zip › Figure 3 updated/3A/F3A Females Liver NDUFA9 c d.tif]

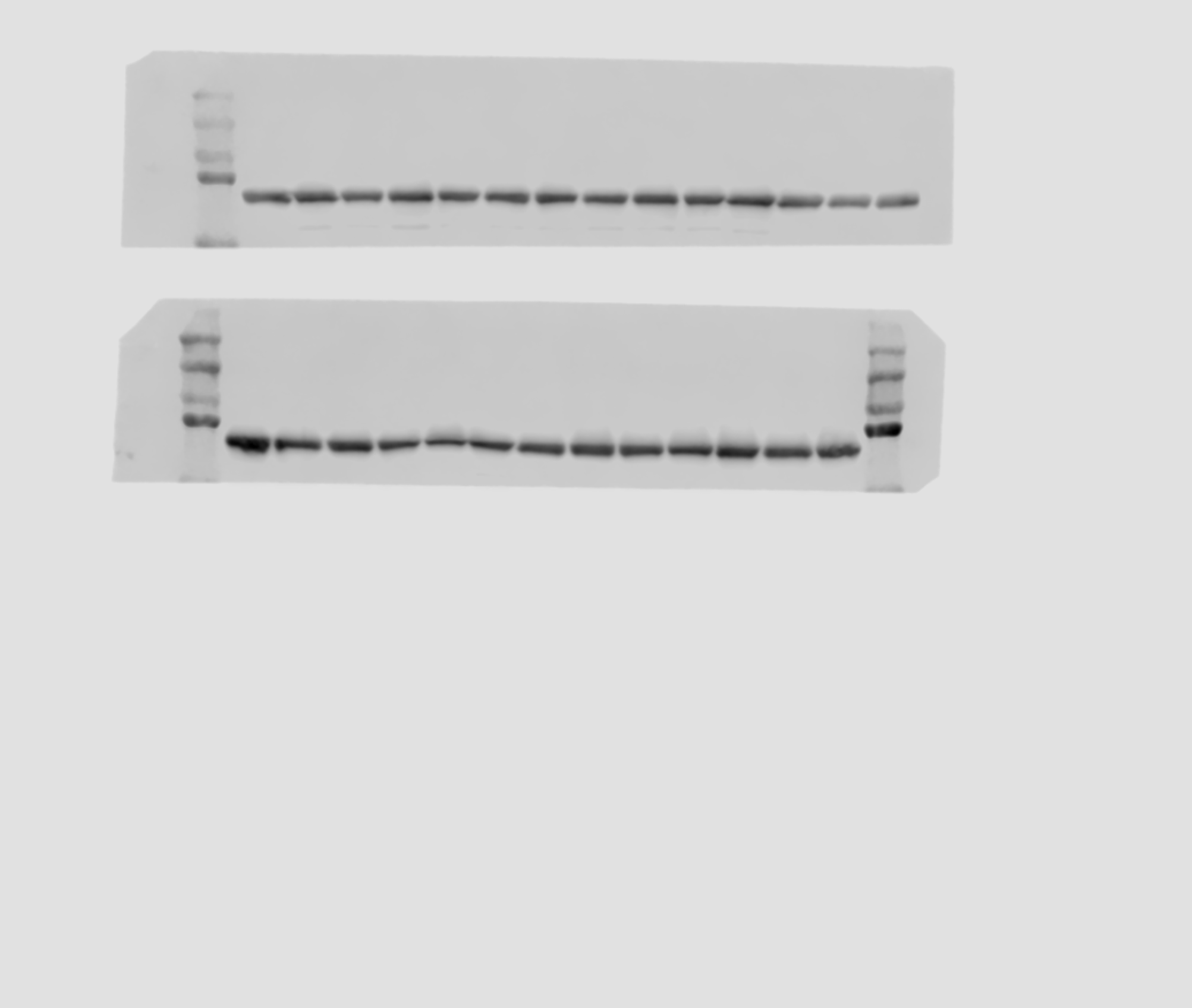

Supplement: Supplementary file 4 — Source data Fig. 3 [file 44321_2026_426_MOESM4_ESM.zip › Figure 3 updated/3A/F3A Females Liver SDHA c d.tif]

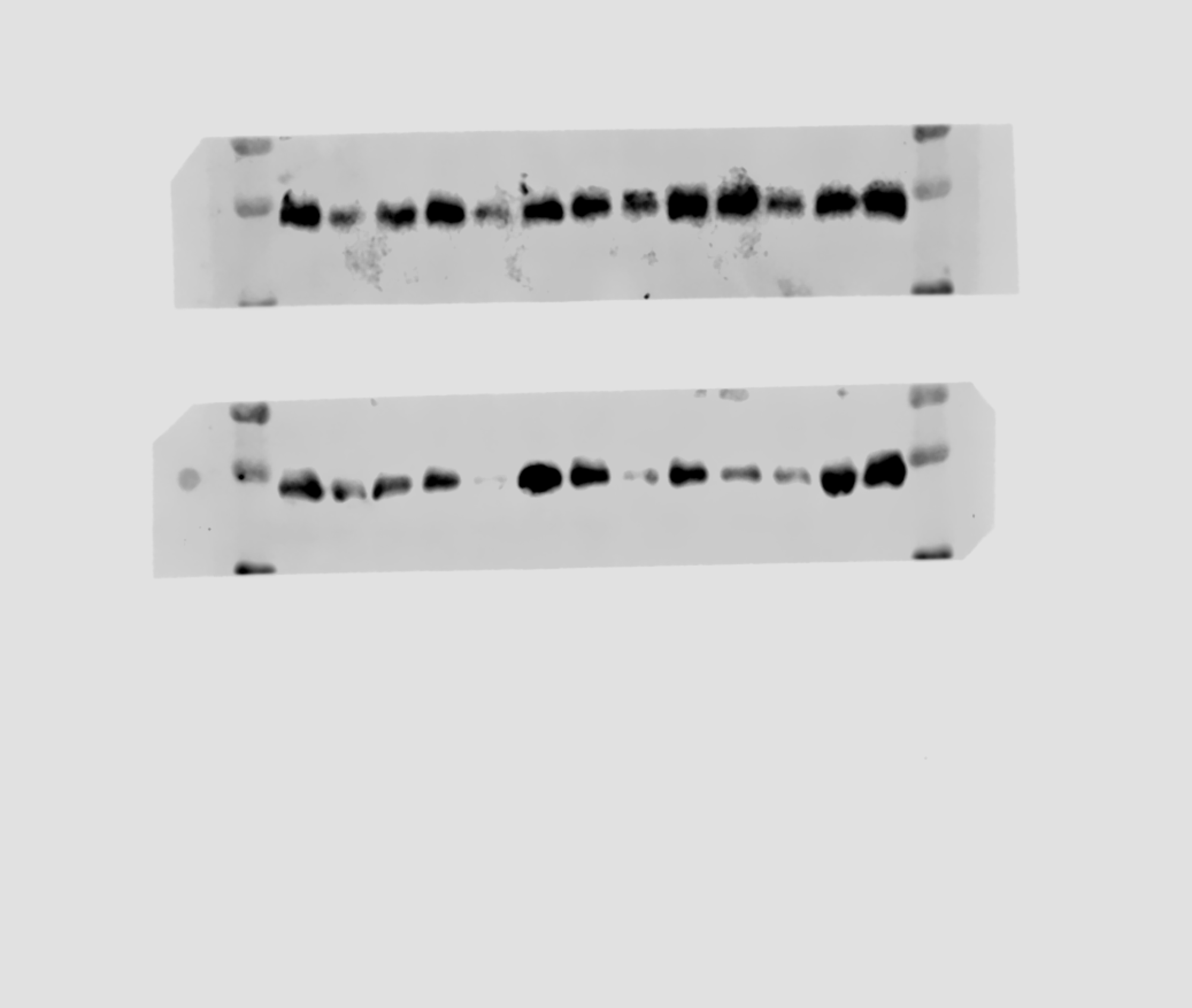

Supplement: Supplementary file 4 — Source data Fig. 3 [file 44321_2026_426_MOESM4_ESM.zip › Figure 3 updated/3A/F3A Males Liver COX1 a b.tif]

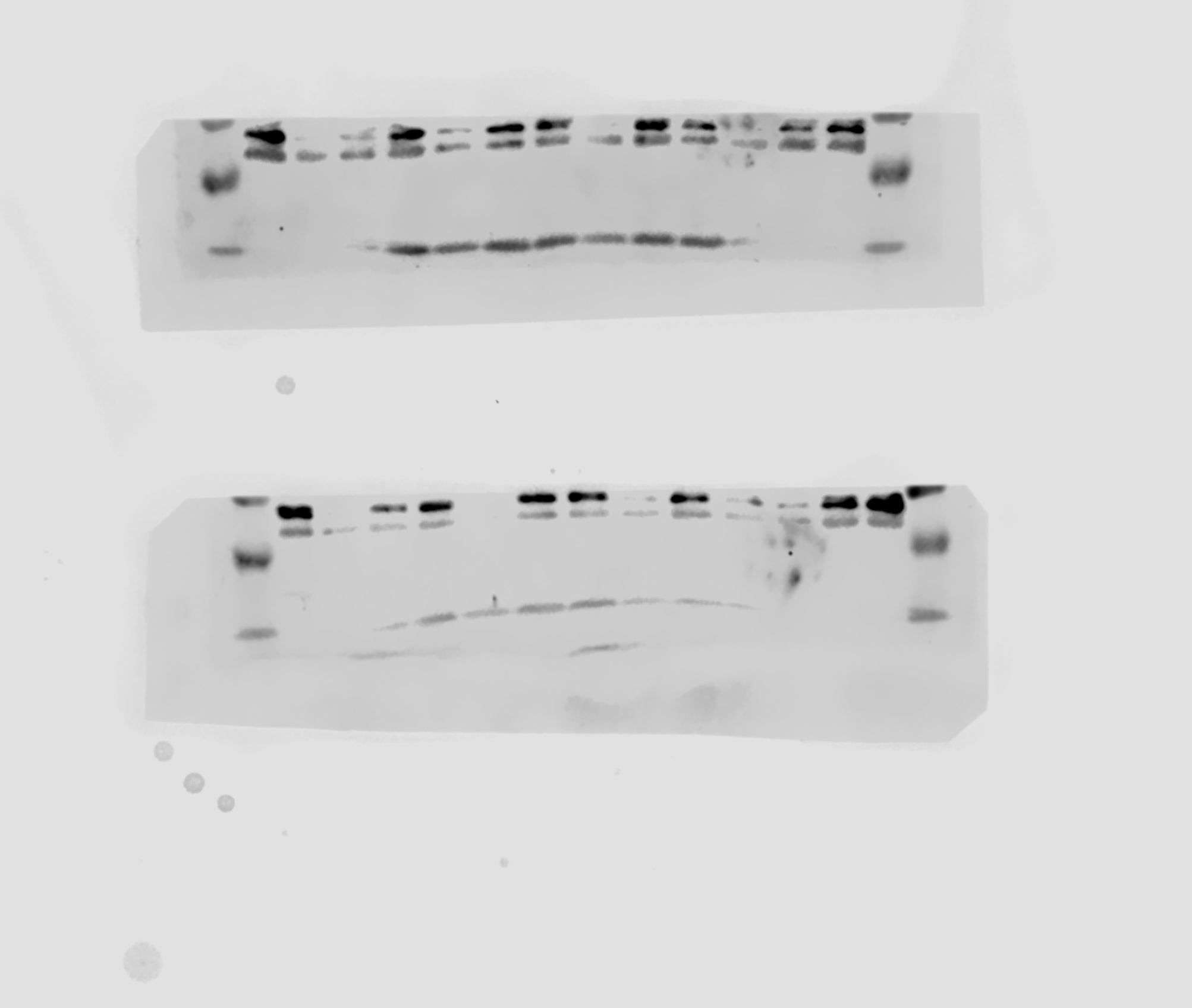

Supplement: Supplementary file 4 — Source data Fig. 3 [file 44321_2026_426_MOESM4_ESM.zip › Figure 3 updated/3A/F3A Males Liver COX2 a b.tif]

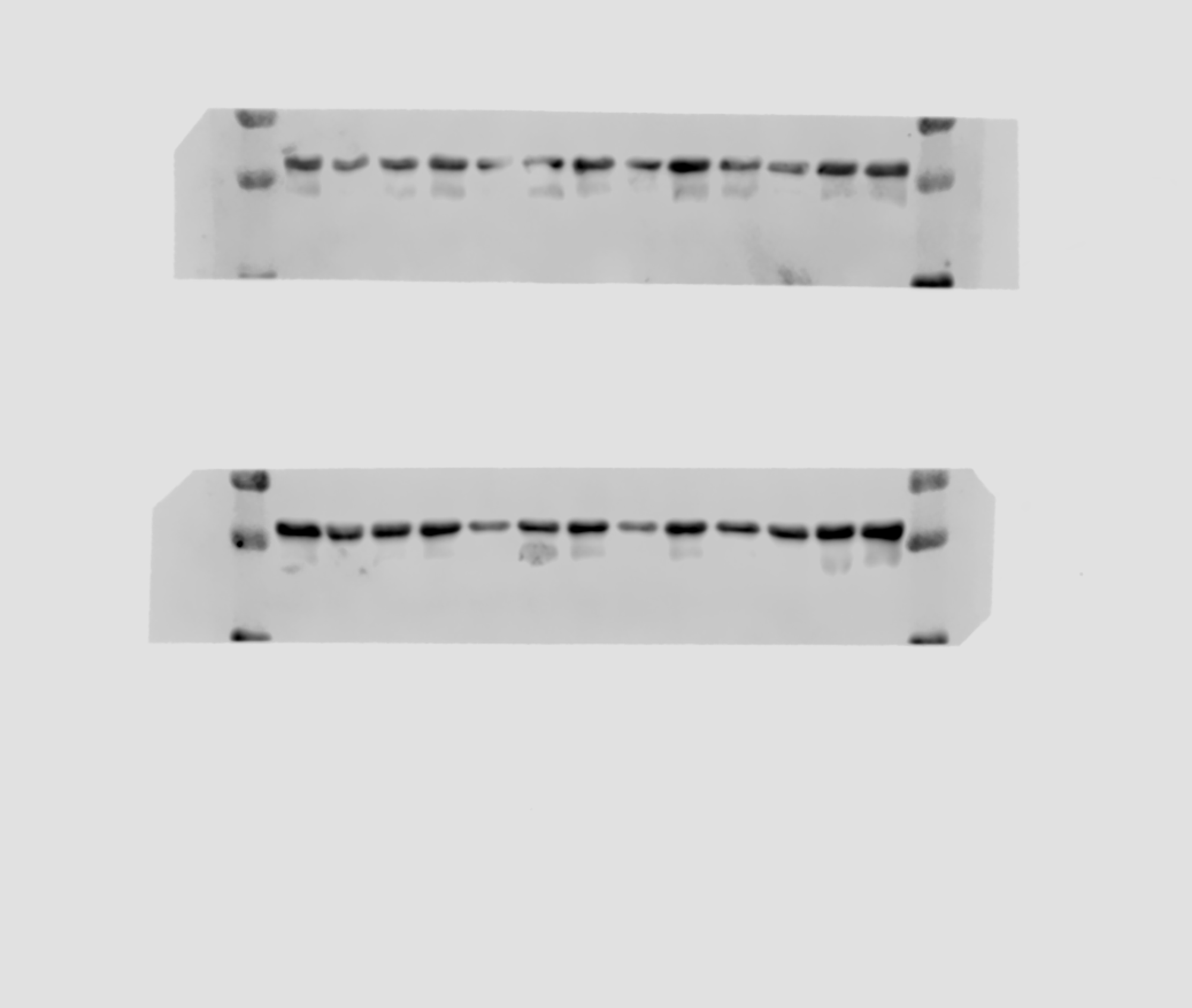

Supplement: Supplementary file 4 — Source data Fig. 3 [file 44321_2026_426_MOESM4_ESM.zip › Figure 3 updated/3A/F3A Males Liver NDUFA10 a b.tif]

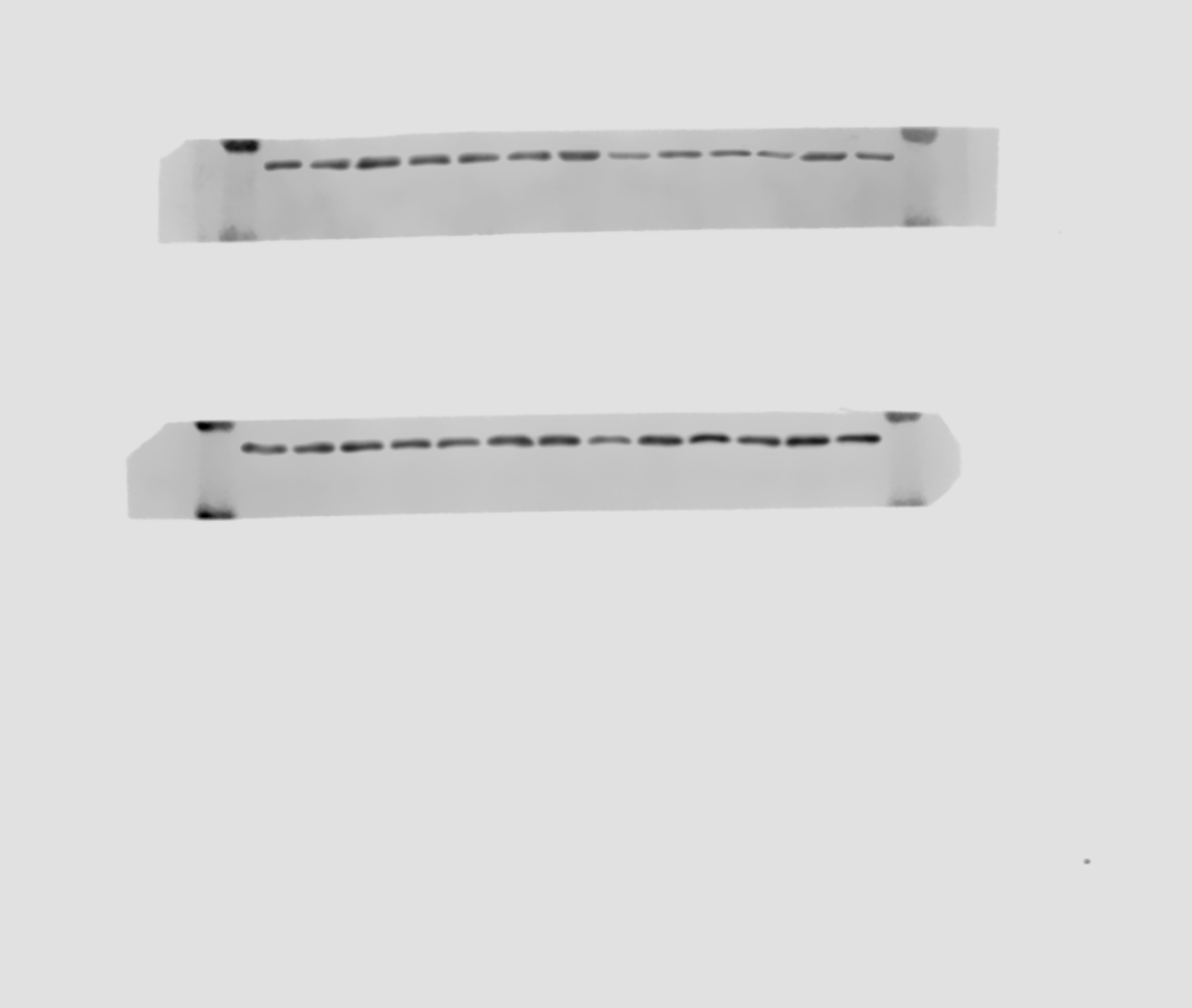

Supplement: Supplementary file 4 — Source data Fig. 3 [file 44321_2026_426_MOESM4_ESM.zip › Figure 3 updated/3A/F3A Males Liver NDUFA9 c d.tif]

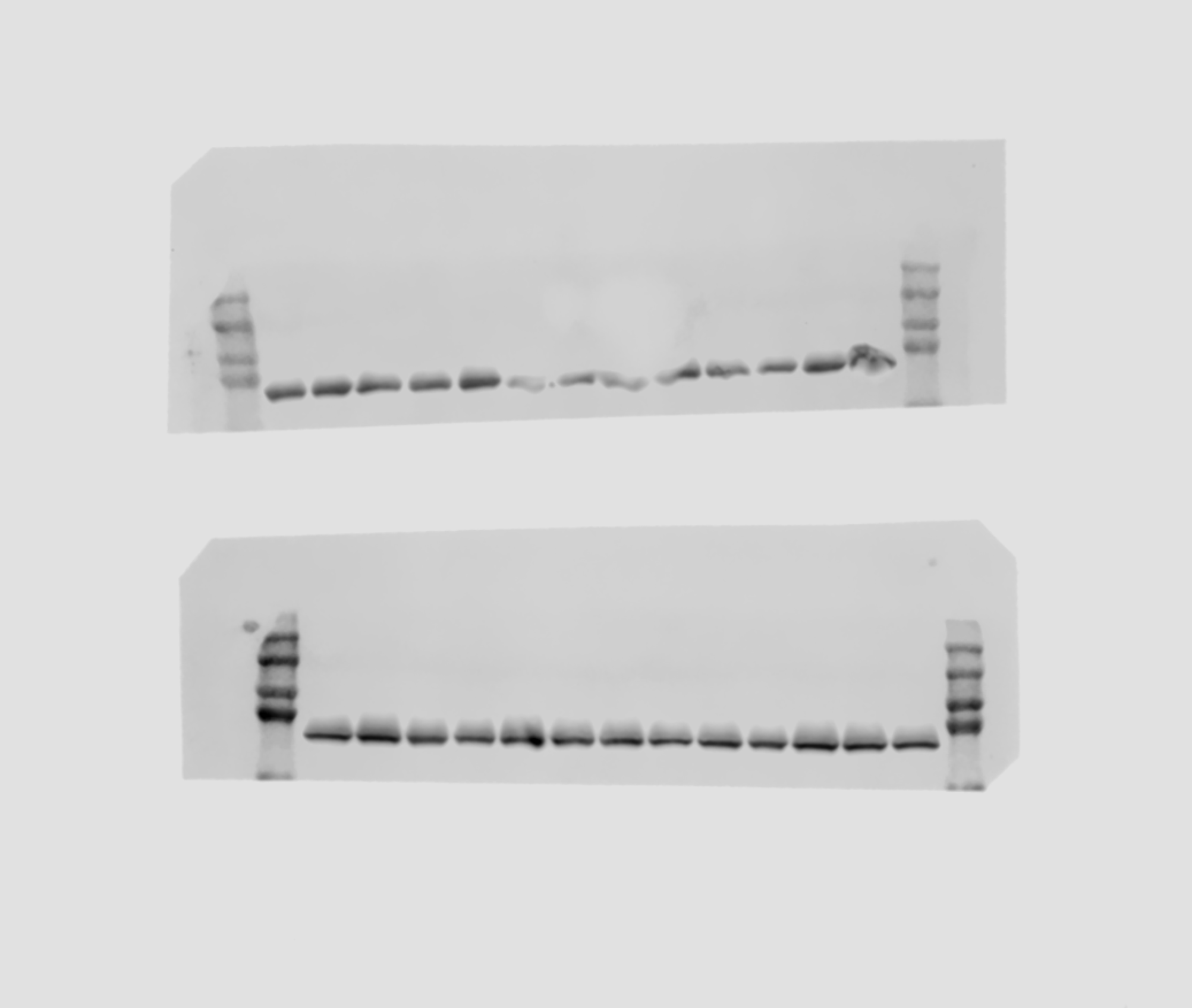

Supplement: Supplementary file 4 — Source data Fig. 3 [file 44321_2026_426_MOESM4_ESM.zip › Figure 3 updated/3A/F3A Males Liver SDHA c d.tif]

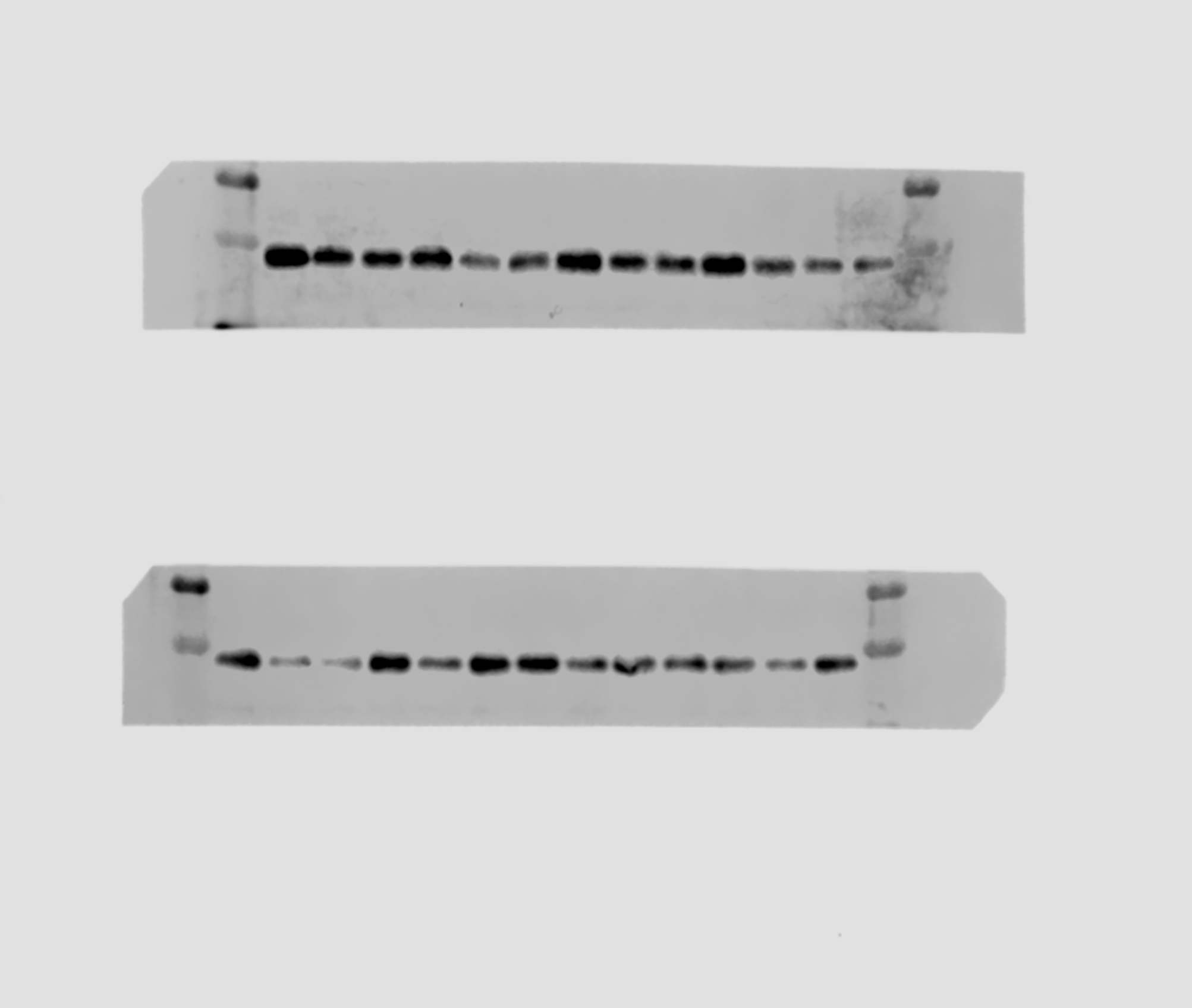

Supplement: Supplementary file 4 — Source data Fig. 3 [file 44321_2026_426_MOESM4_ESM.zip › Figure 3 updated/3B/F3B Females Brain COX1 a b.tif]

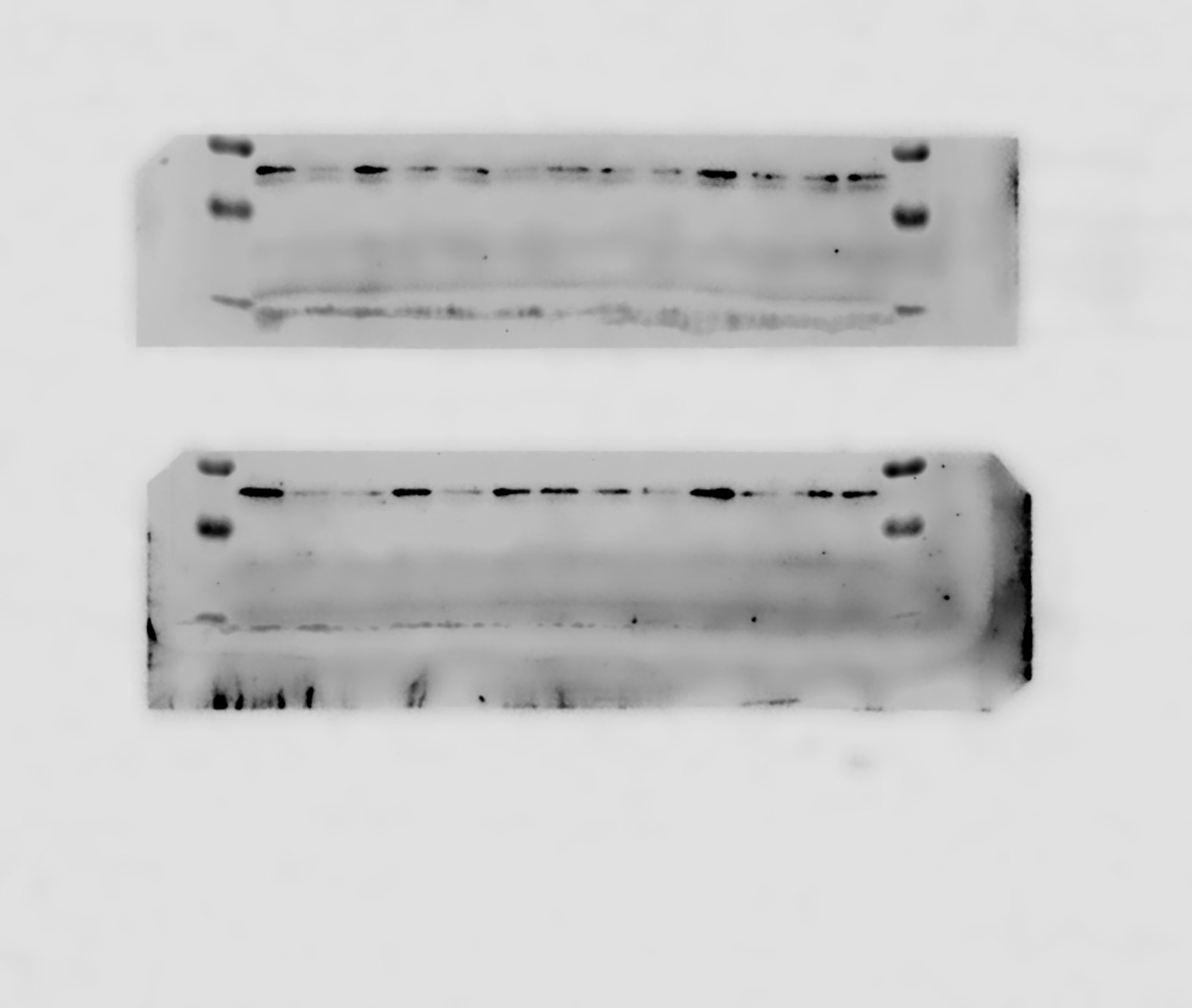

Supplement: Supplementary file 4 — Source data Fig. 3 [file 44321_2026_426_MOESM4_ESM.zip › Figure 3 updated/3B/F3B Females Brain COX2 a b.tif]

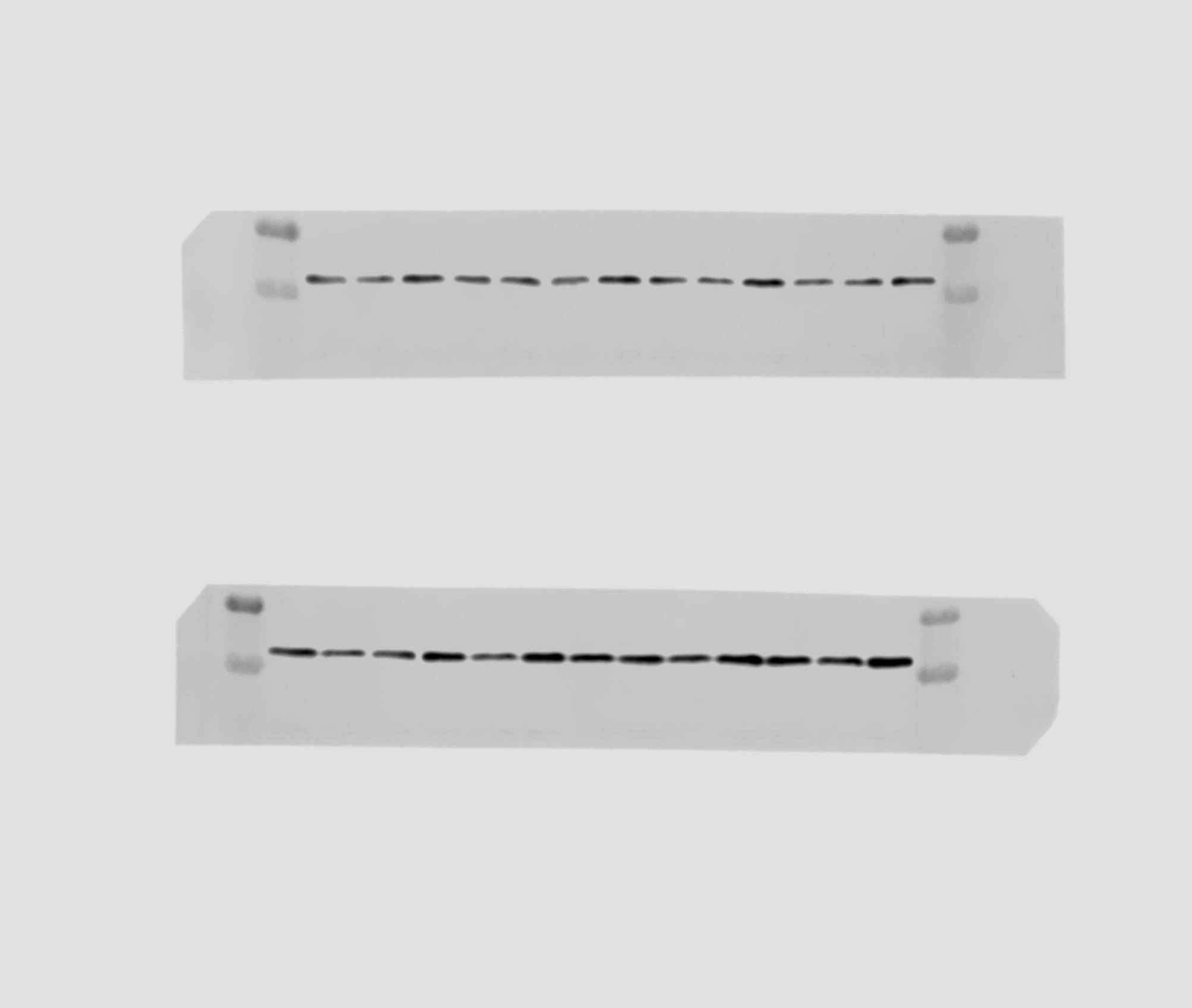

Supplement: Supplementary file 4 — Source data Fig. 3 [file 44321_2026_426_MOESM4_ESM.zip › Figure 3 updated/3B/F3B Females Brain NDUFA10 a b.tif]

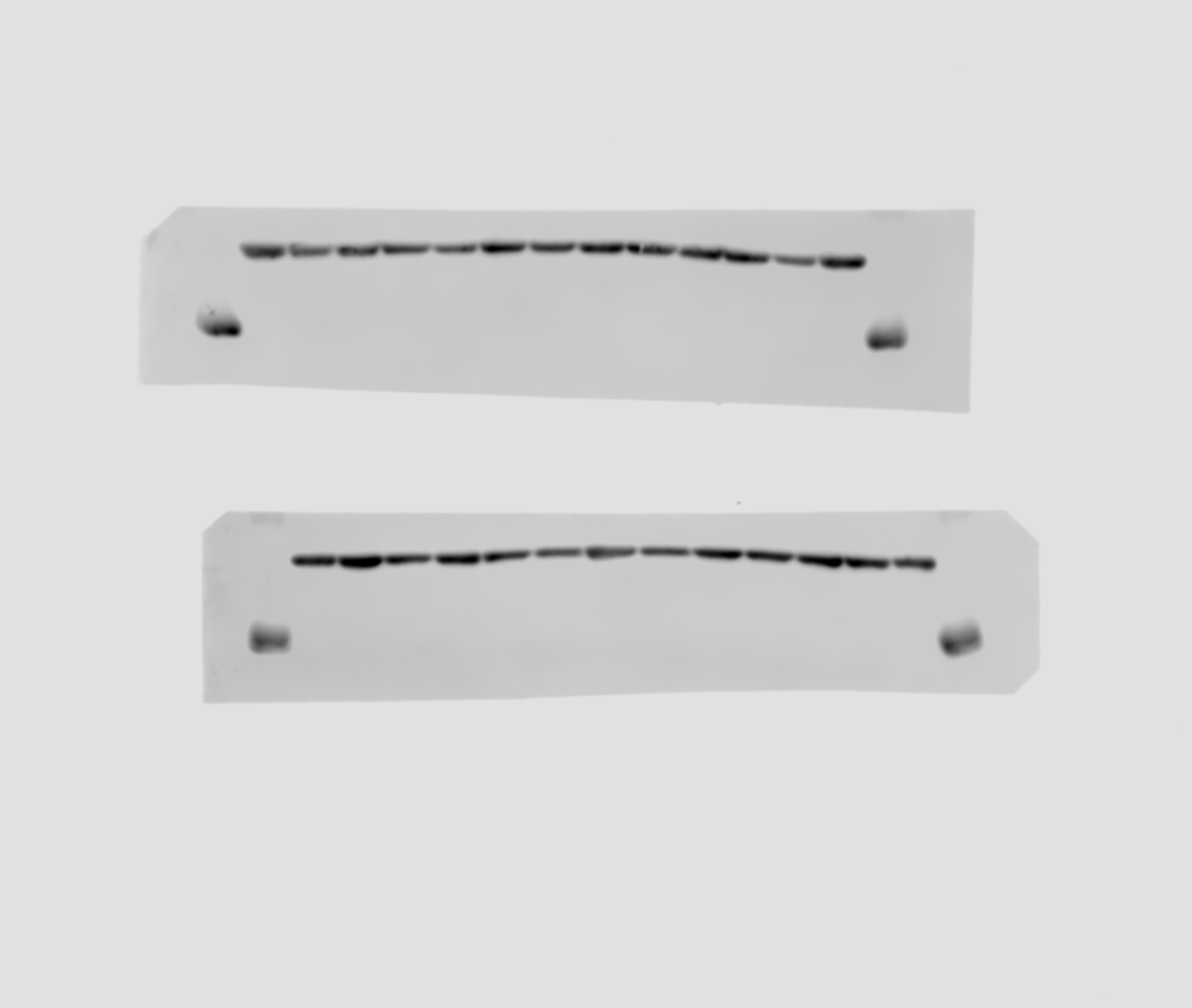

Supplement: Supplementary file 4 — Source data Fig. 3 [file 44321_2026_426_MOESM4_ESM.zip › Figure 3 updated/3B/F3B Females Brain NDUFA9 c d.tif]

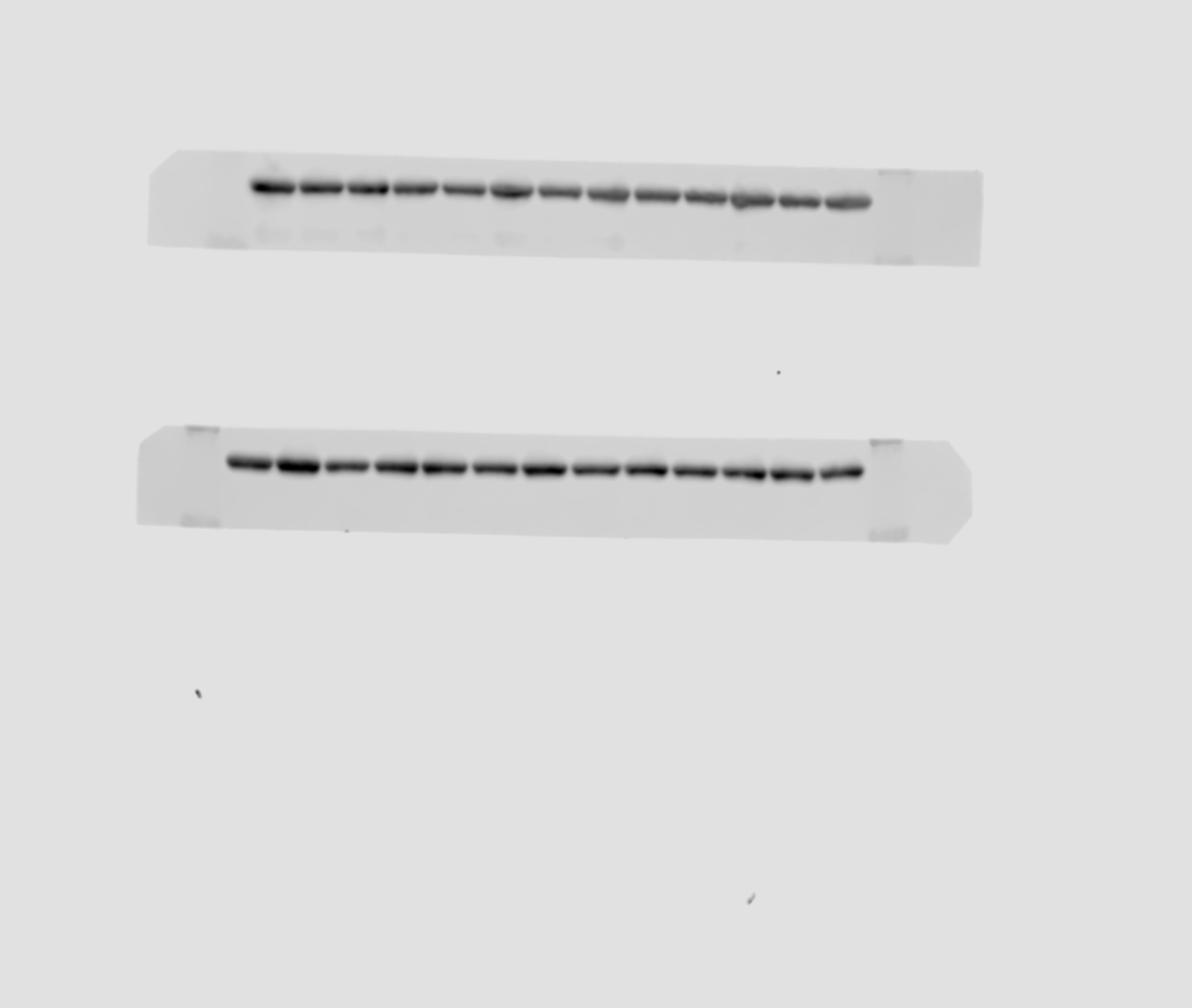

Supplement: Supplementary file 4 — Source data Fig. 3 [file 44321_2026_426_MOESM4_ESM.zip › Figure 3 updated/3B/F3B Females Brain SDHA c d.tif]

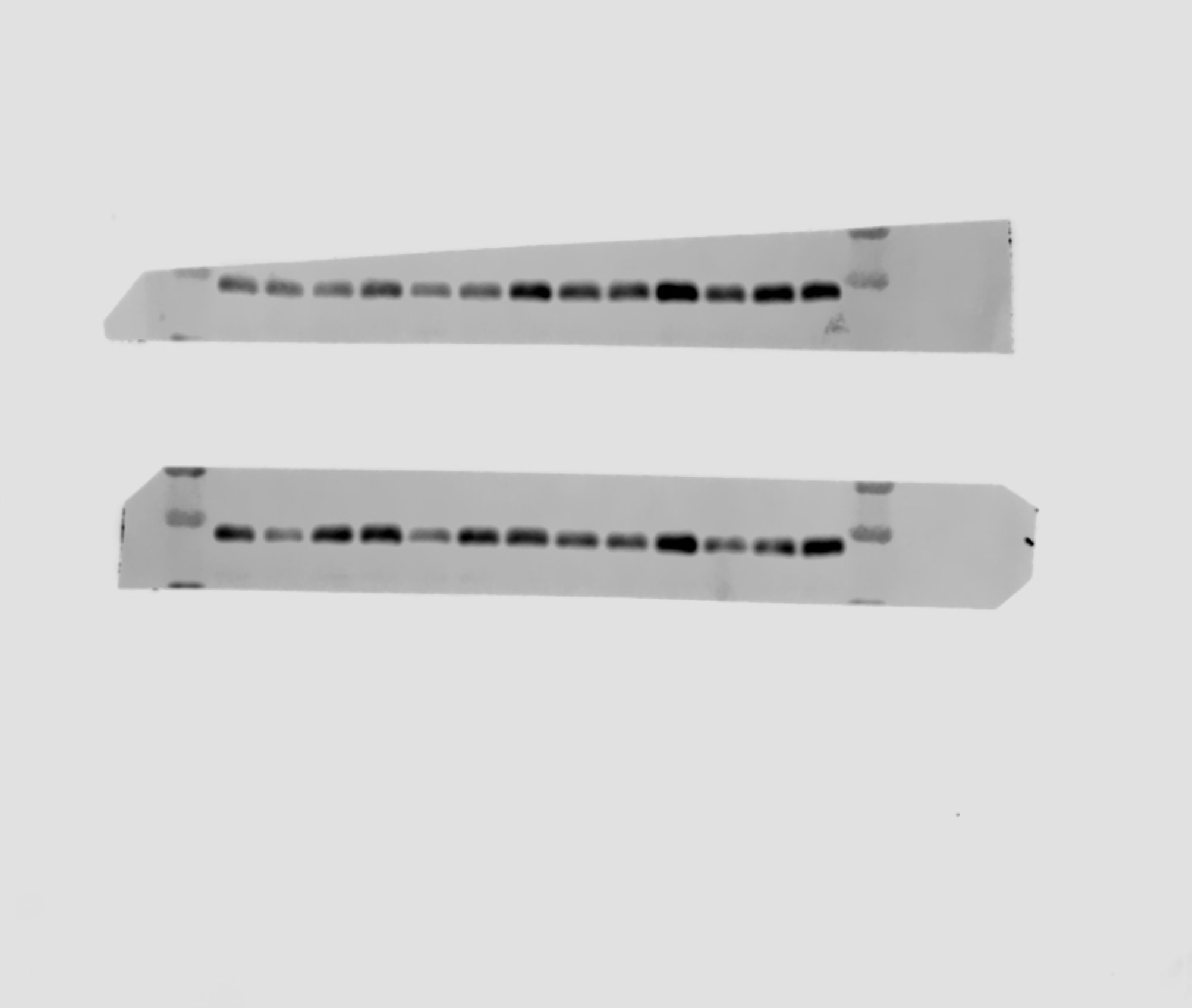

Supplement: Supplementary file 4 — Source data Fig. 3 [file 44321_2026_426_MOESM4_ESM.zip › Figure 3 updated/3B/F3B Males Brain COX1 a b.tif]

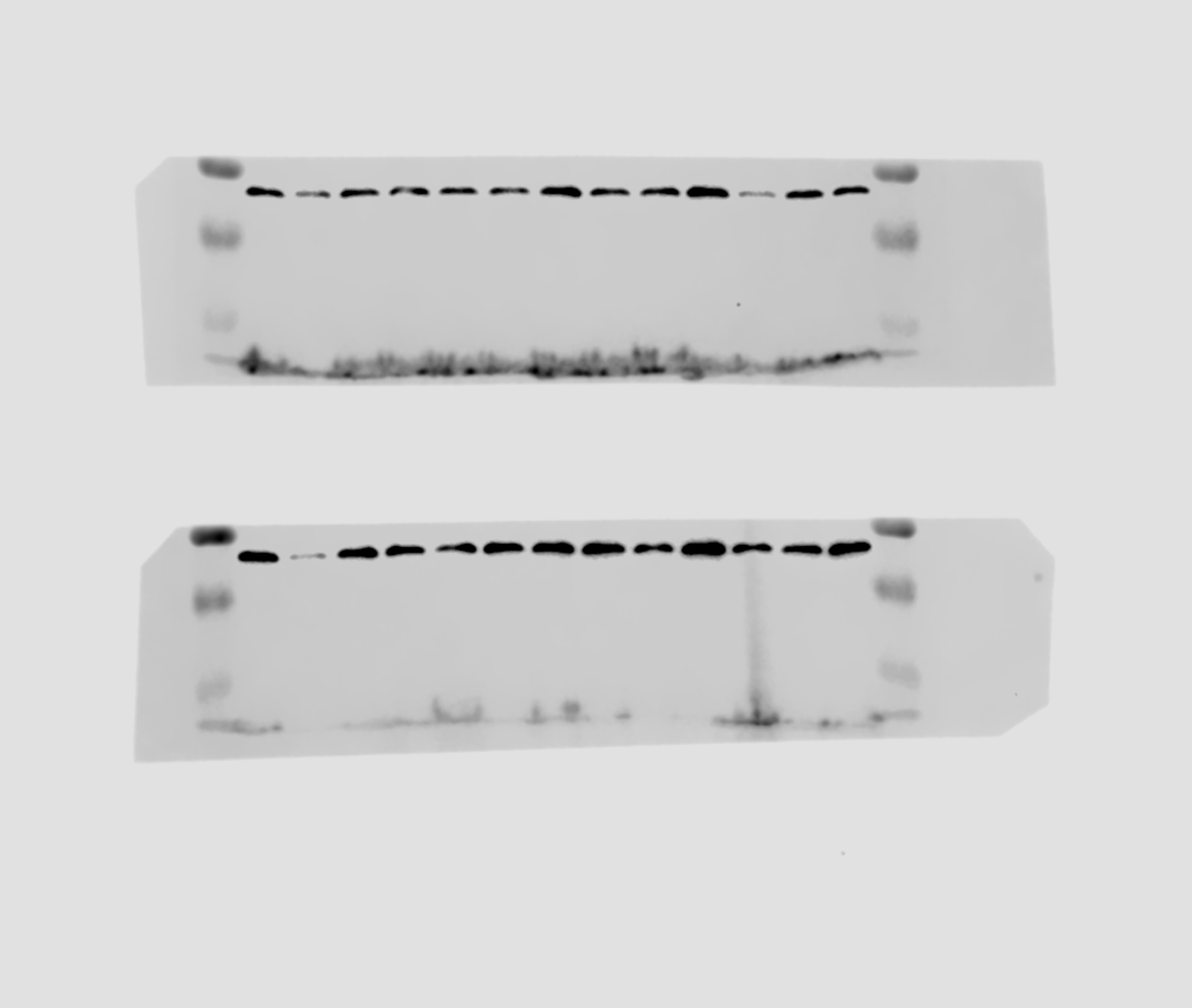

Supplement: Supplementary file 4 — Source data Fig. 3 [file 44321_2026_426_MOESM4_ESM.zip › Figure 3 updated/3B/F3B Males Brain COX2 a b.tif]

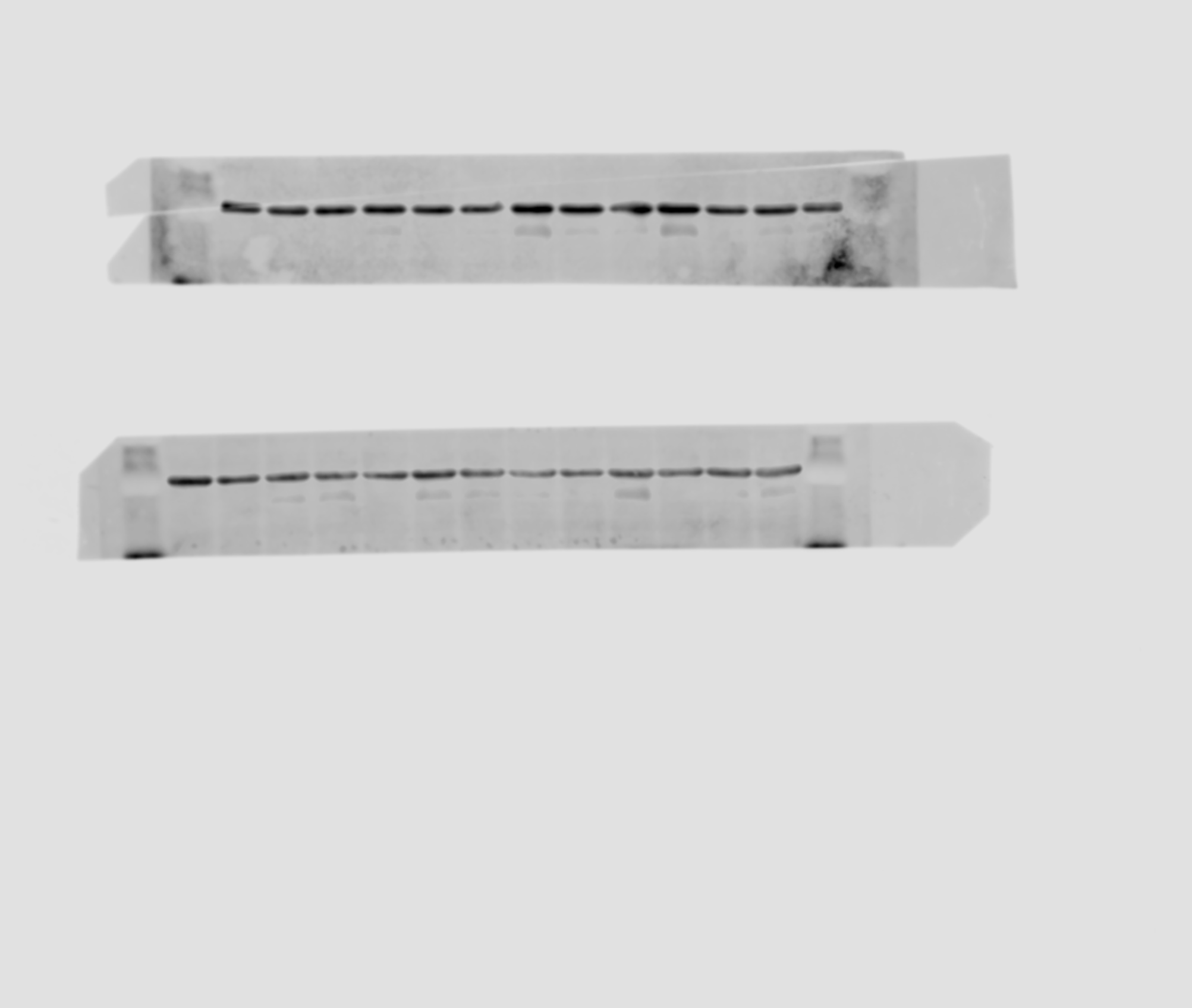

Supplement: Supplementary file 4 — Source data Fig. 3 [file 44321_2026_426_MOESM4_ESM.zip › Figure 3 updated/3B/F3B Males Brain NDUFA10 a b.tif]

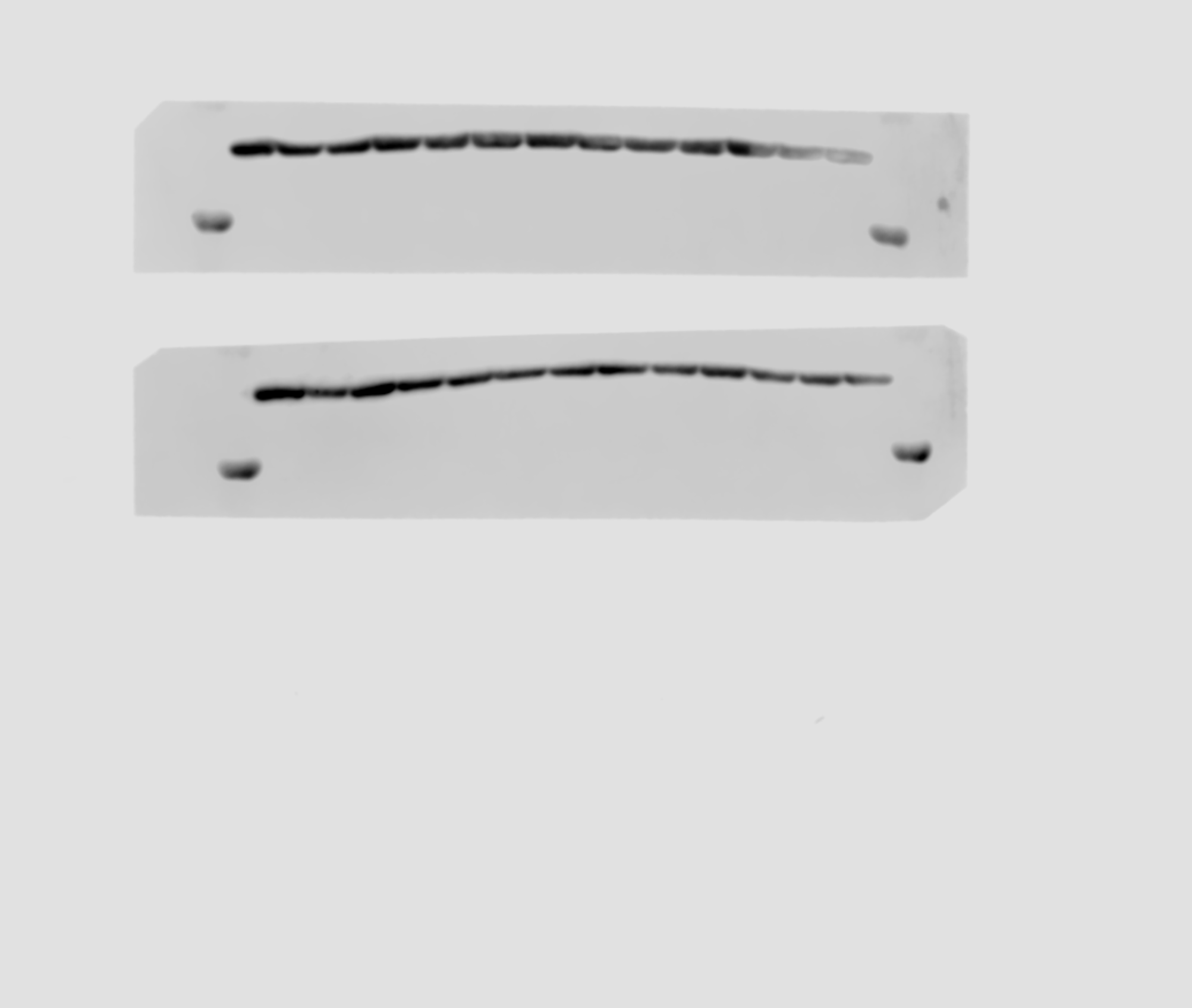

Supplement: Supplementary file 4 — Source data Fig. 3 [file 44321_2026_426_MOESM4_ESM.zip › Figure 3 updated/3B/F3B Males Brain NDUFA9 c d.tif]

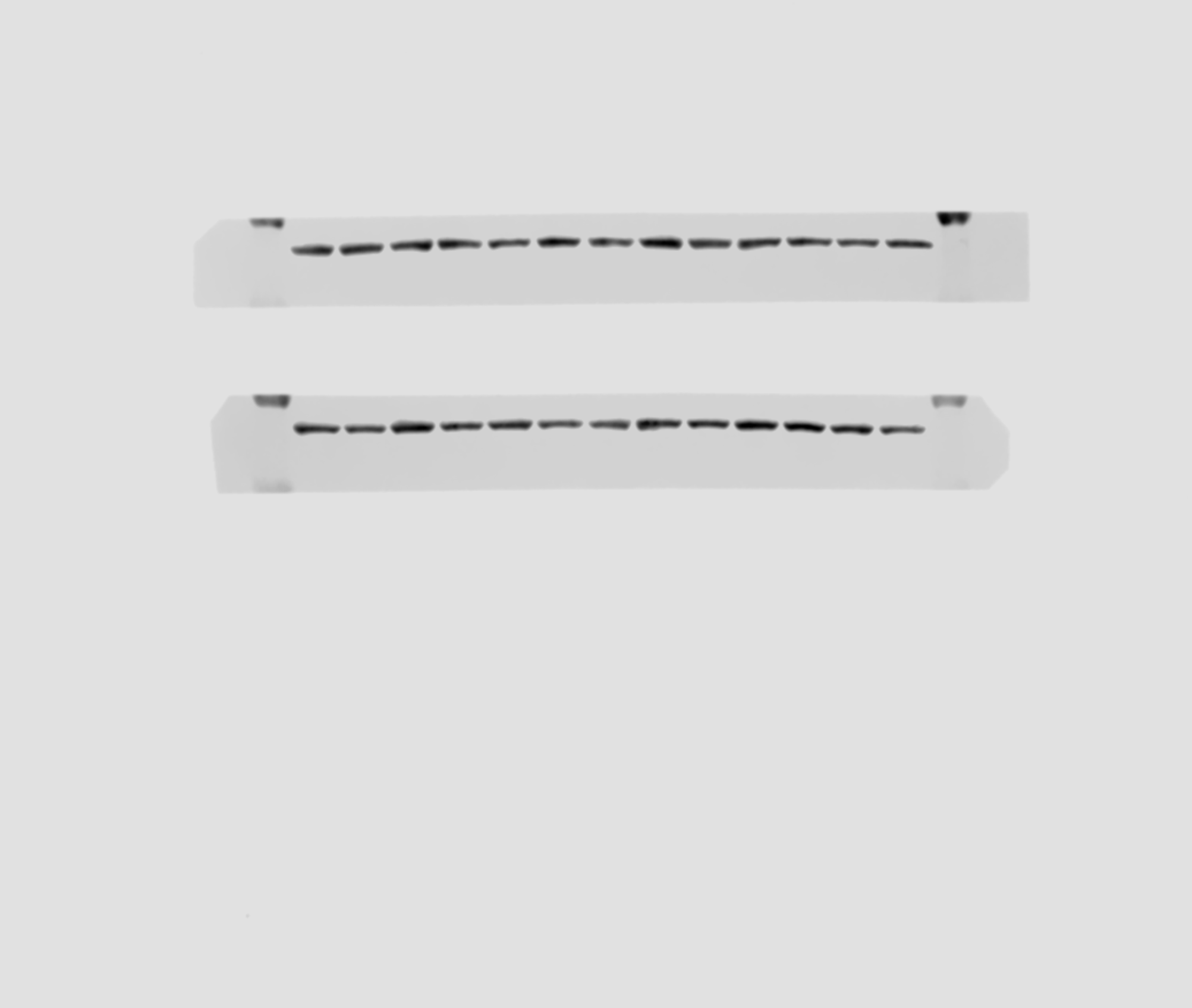

Supplement: Supplementary file 4 — Source data Fig. 3 [file 44321_2026_426_MOESM4_ESM.zip › Figure 3 updated/3B/F3B Males Brain SDHA c d.tif]

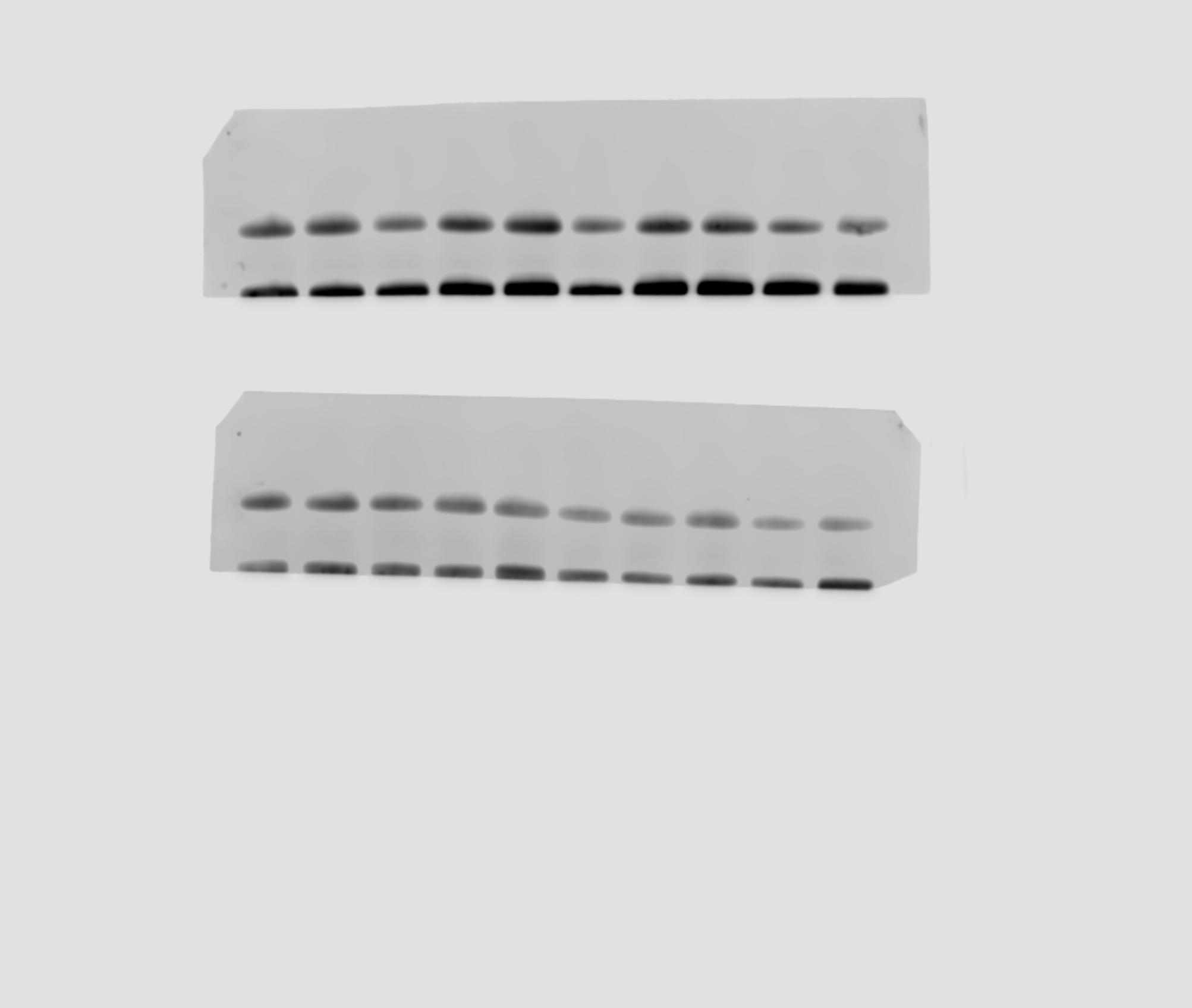

Supplement: Supplementary file 5 — Source data Fig. 4 [file 44321_2026_426_MOESM5_ESM.zip › Figure 4 updated/4A/F4A Females Liver CI a b.tif]

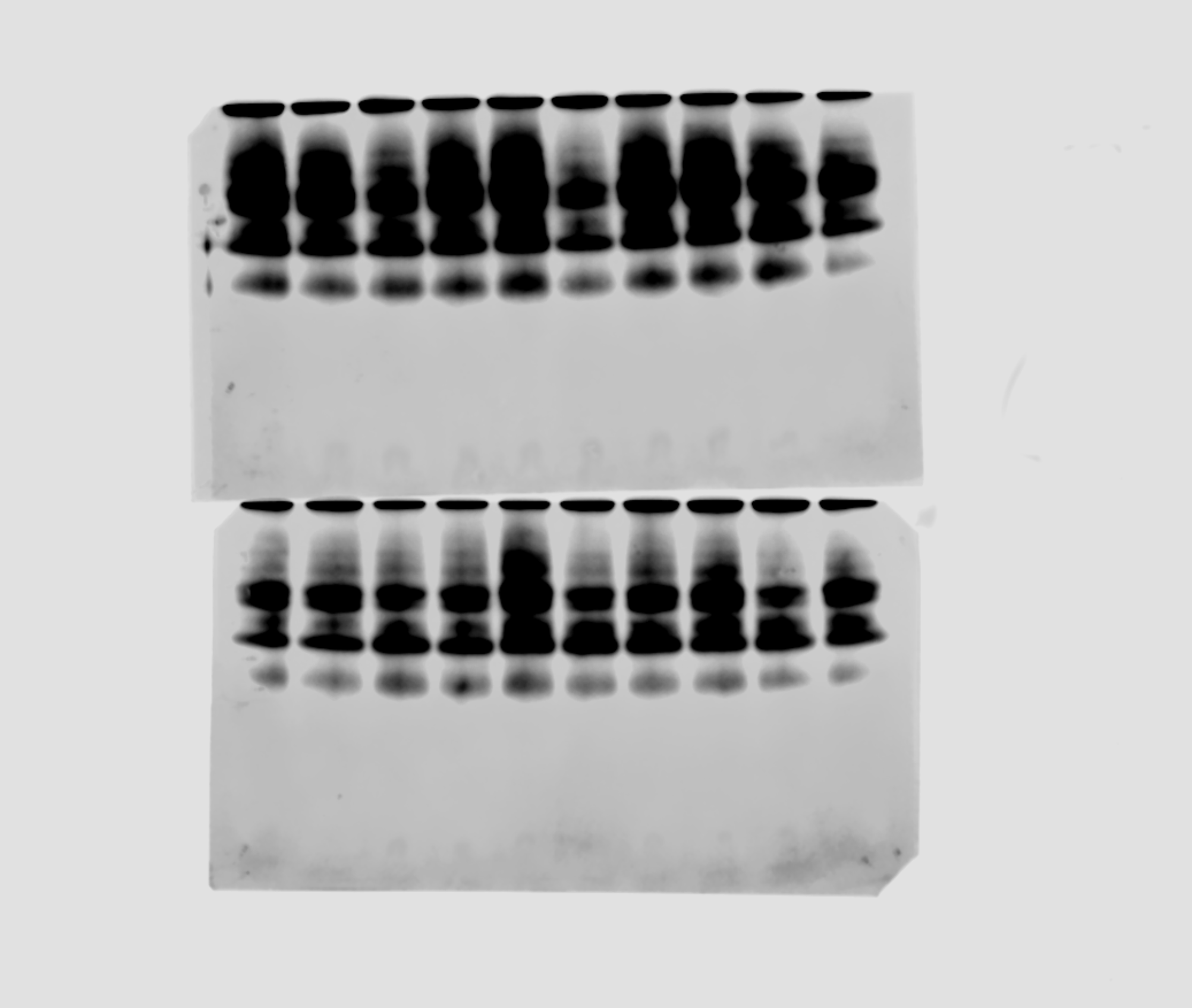

Supplement: Supplementary file 5 — Source data Fig. 4 [file 44321_2026_426_MOESM5_ESM.zip › Figure 4 updated/4A/F4A Females Liver CII a b.tif]

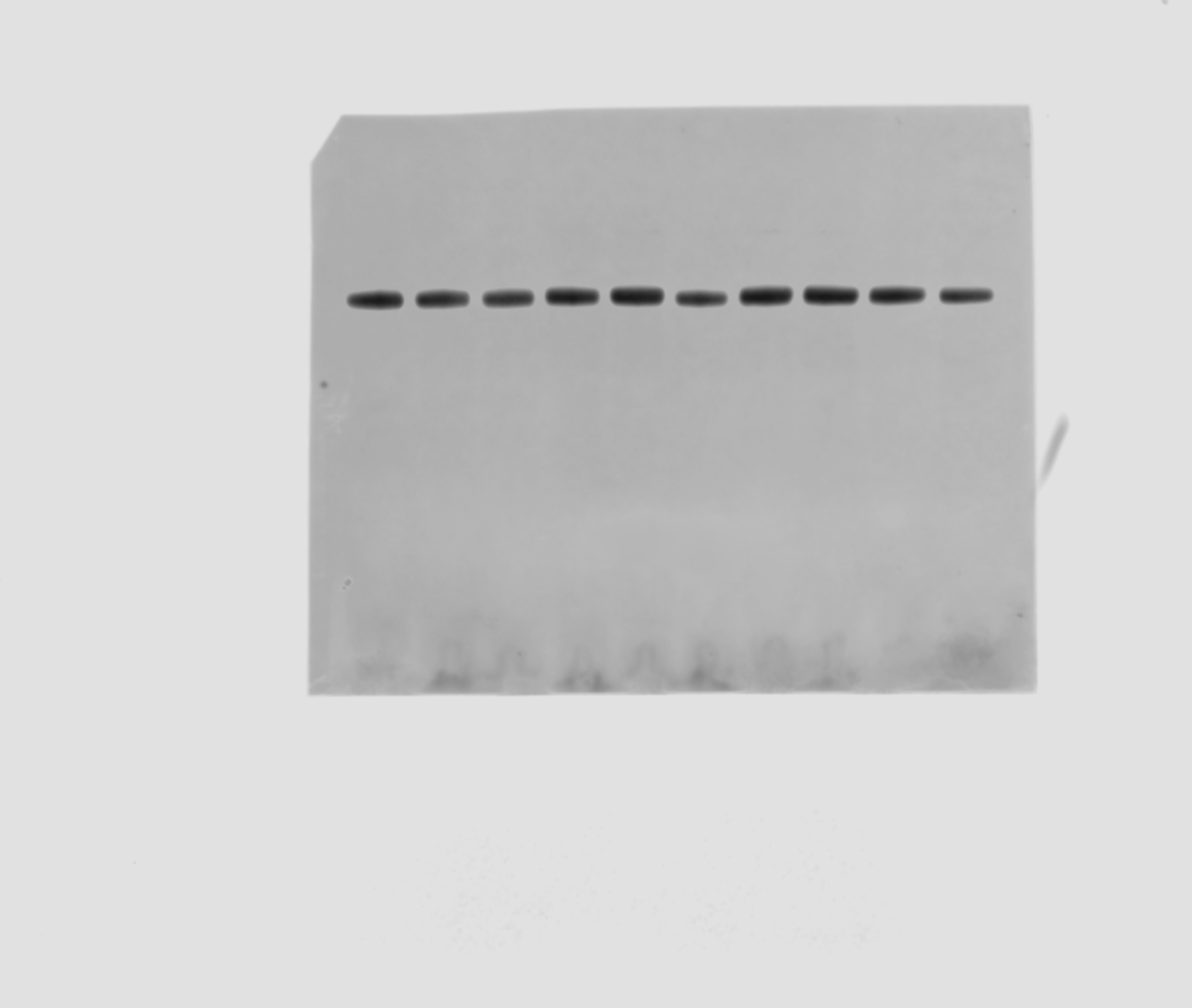

Supplement: Supplementary file 5 — Source data Fig. 4 [file 44321_2026_426_MOESM5_ESM.zip › Figure 4 updated/4A/F4A Females Liver CIII a.tif]

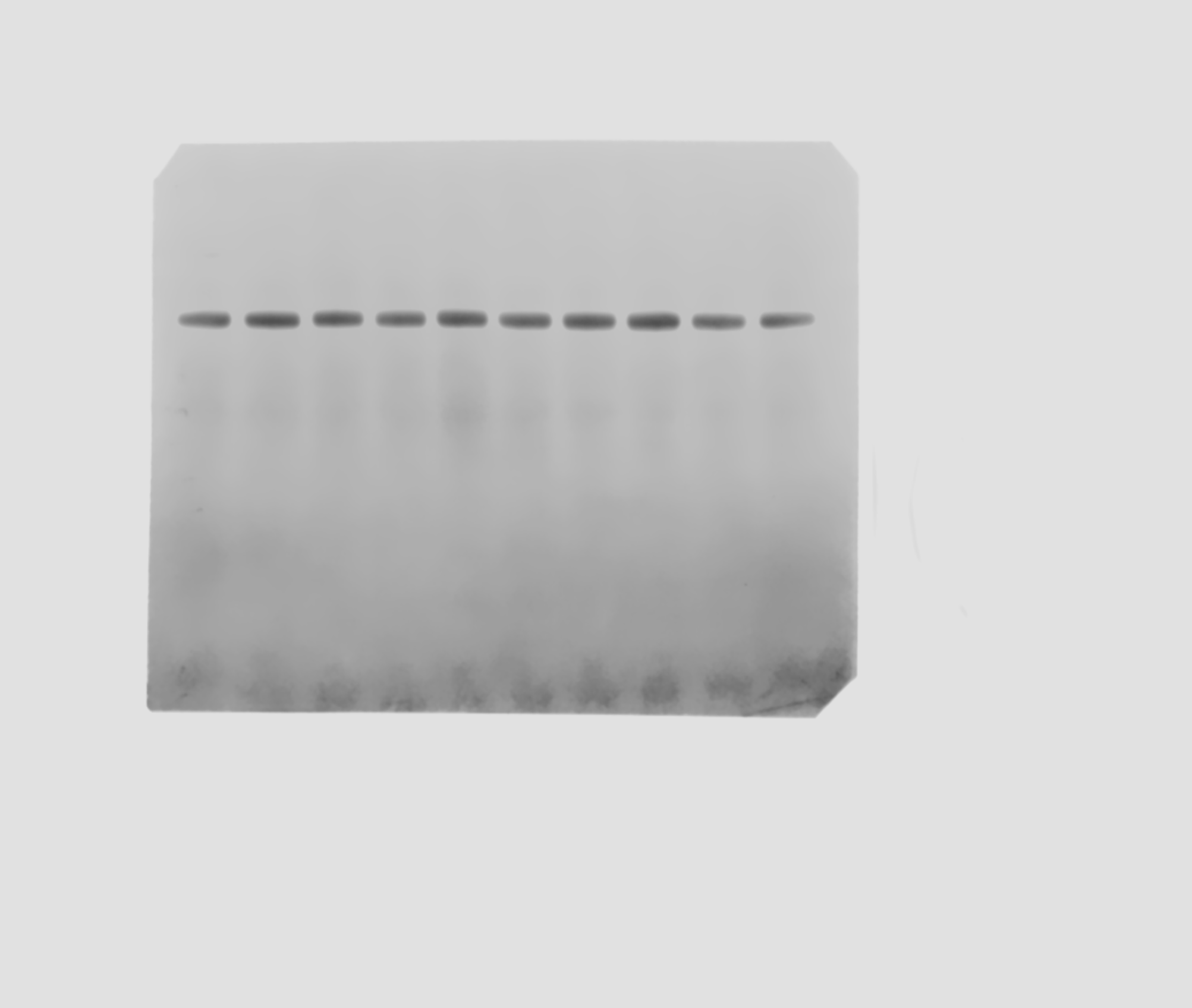

Supplement: Supplementary file 5 — Source data Fig. 4 [file 44321_2026_426_MOESM5_ESM.zip › Figure 4 updated/4A/F4A Females Liver CIII b.tif]

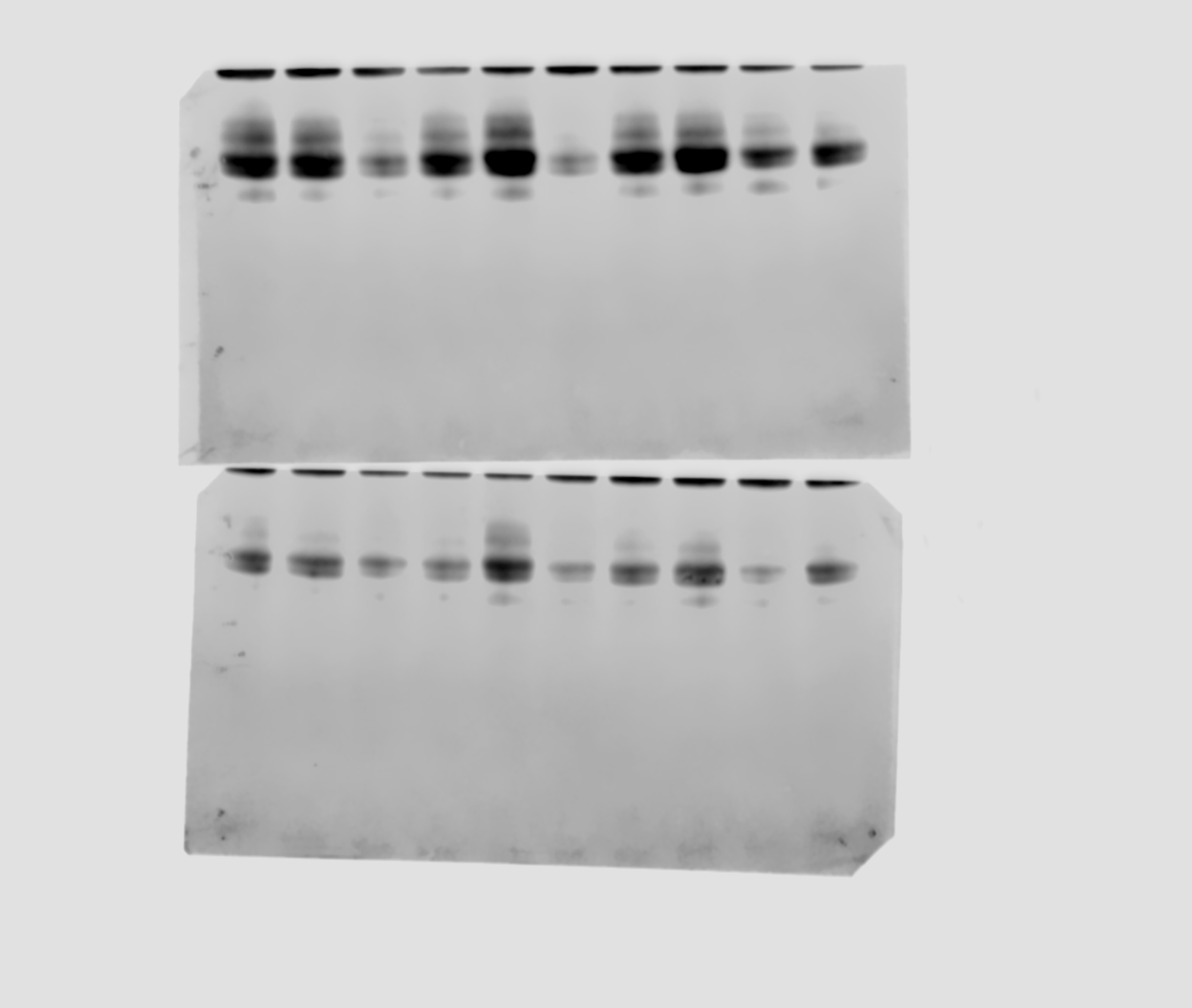

Supplement: Supplementary file 5 — Source data Fig. 4 [file 44321_2026_426_MOESM5_ESM.zip › Figure 4 updated/4A/F4A Females Liver CIV a b.tif]

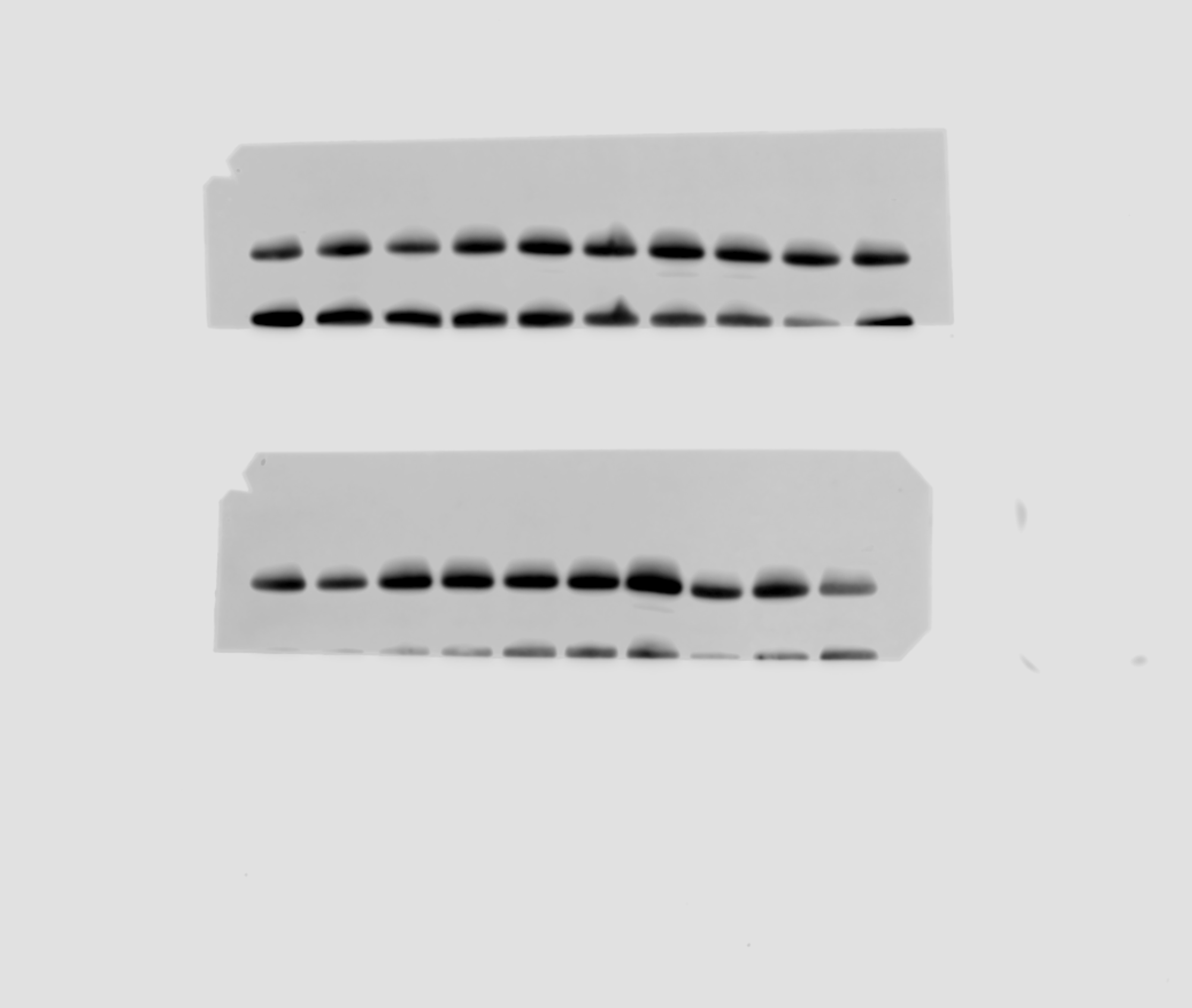

Supplement: Supplementary file 5 — Source data Fig. 4 [file 44321_2026_426_MOESM5_ESM.zip › Figure 4 updated/4A/F4A Males Liver CI a b.tif]

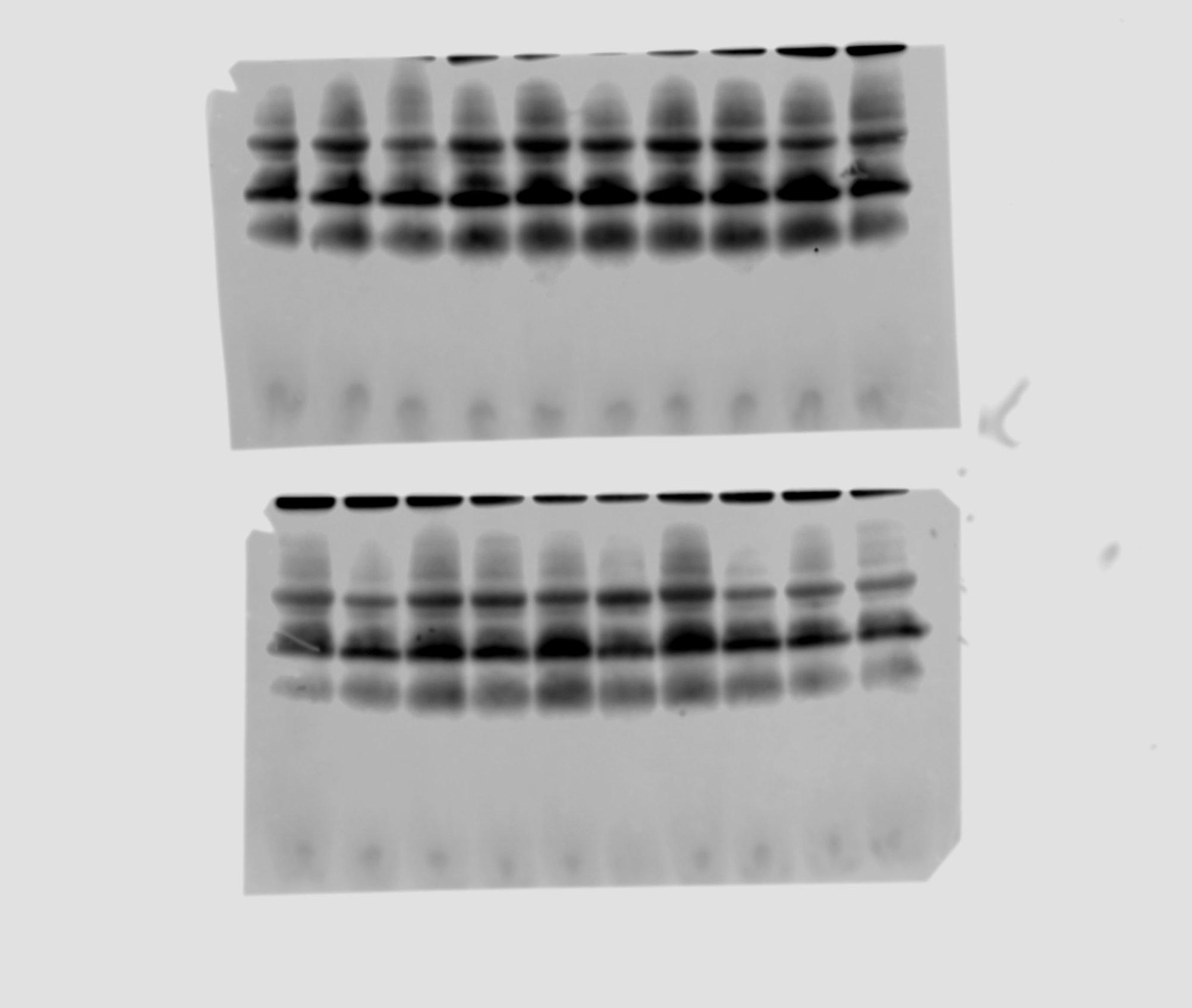

Supplement: Supplementary file 5 — Source data Fig. 4 [file 44321_2026_426_MOESM5_ESM.zip › Figure 4 updated/4A/F4A Males Liver CII a b.tif]

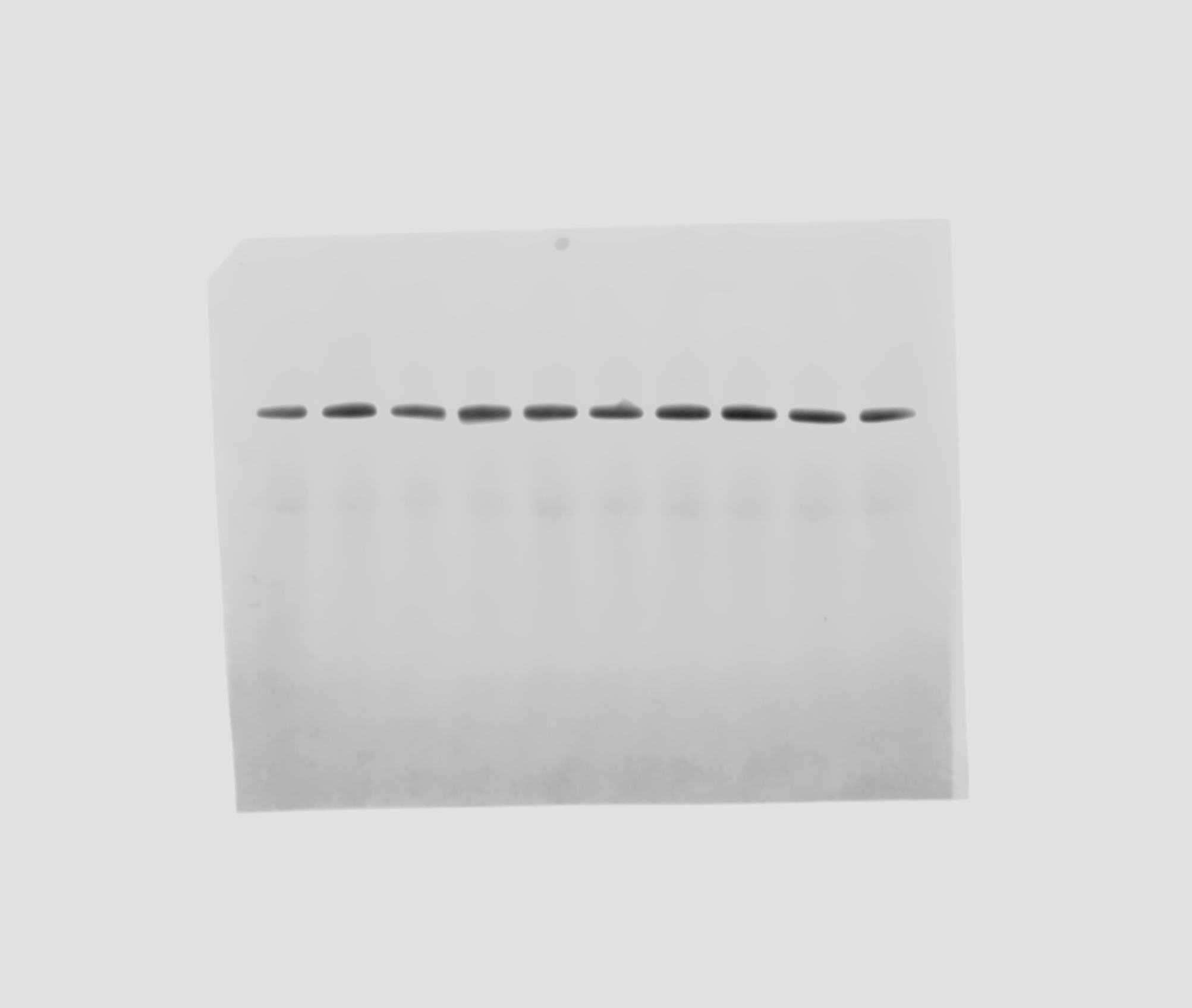

Supplement: Supplementary file 5 — Source data Fig. 4 [file 44321_2026_426_MOESM5_ESM.zip › Figure 4 updated/4A/F4A Males Liver CIII a.tif]

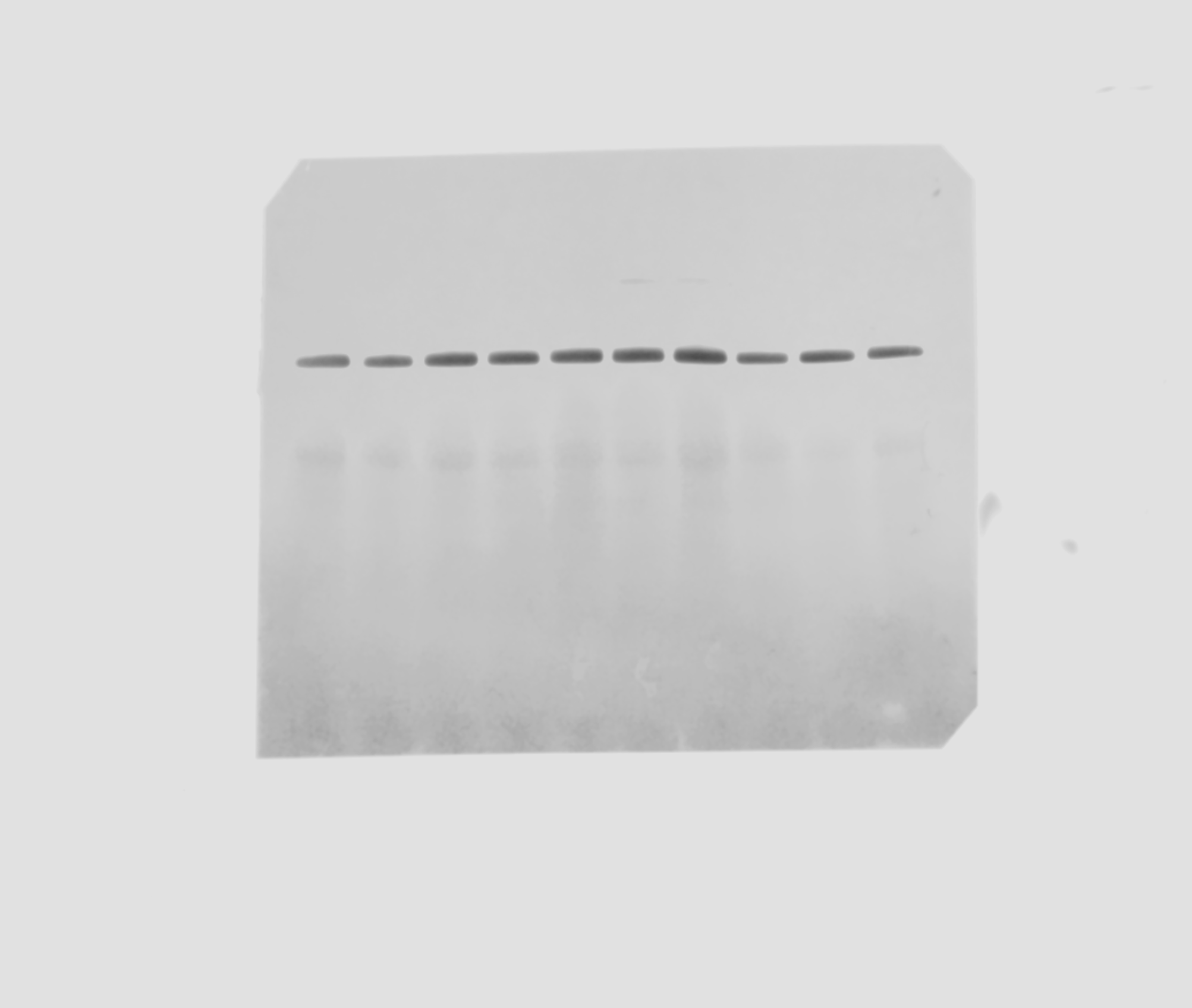

Supplement: Supplementary file 5 — Source data Fig. 4 [file 44321_2026_426_MOESM5_ESM.zip › Figure 4 updated/4A/F4A Males Liver CIII b.tif]

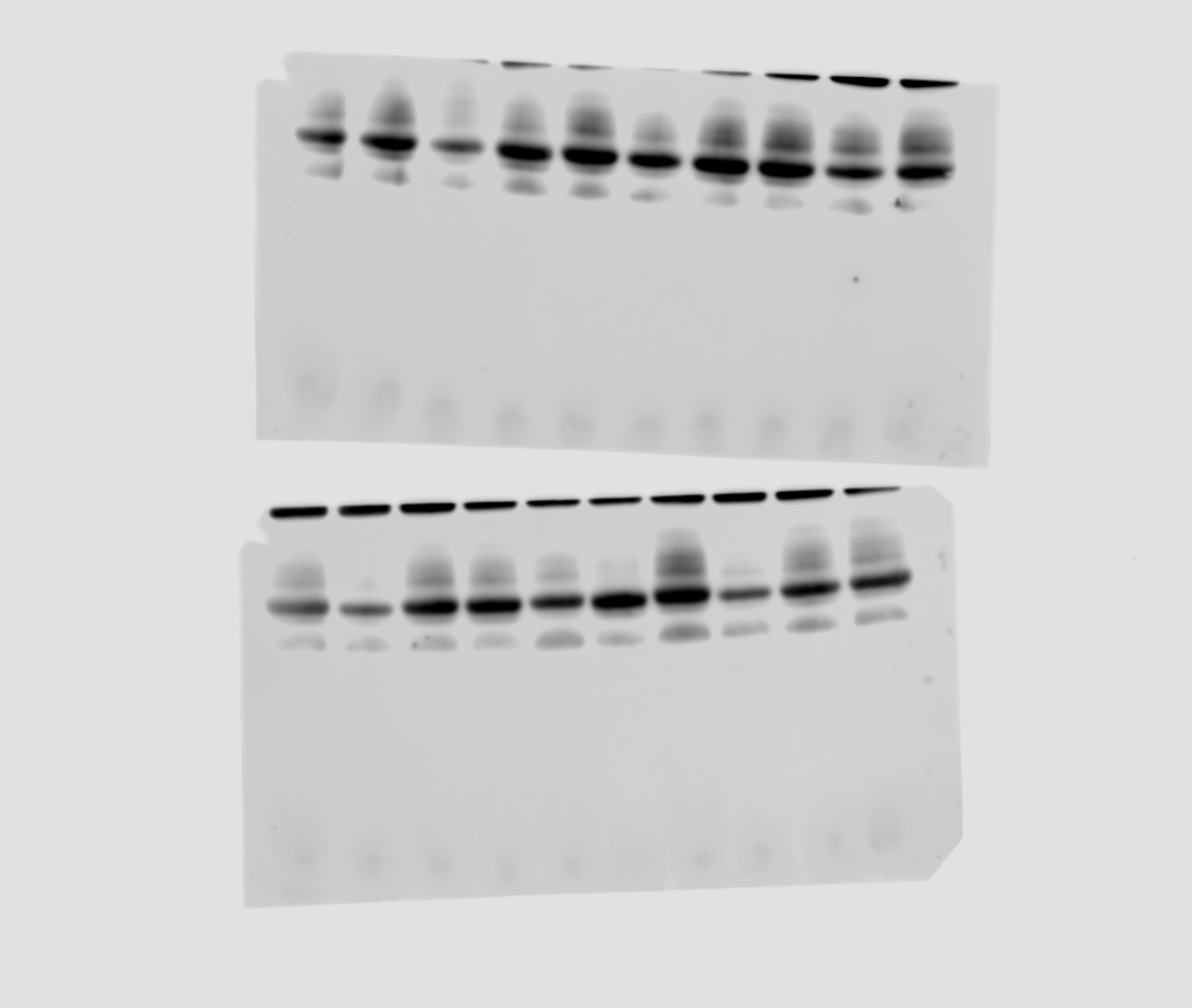

Supplement: Supplementary file 5 — Source data Fig. 4 [file 44321_2026_426_MOESM5_ESM.zip › Figure 4 updated/4A/F4A Males Liver CIV a b.tif]

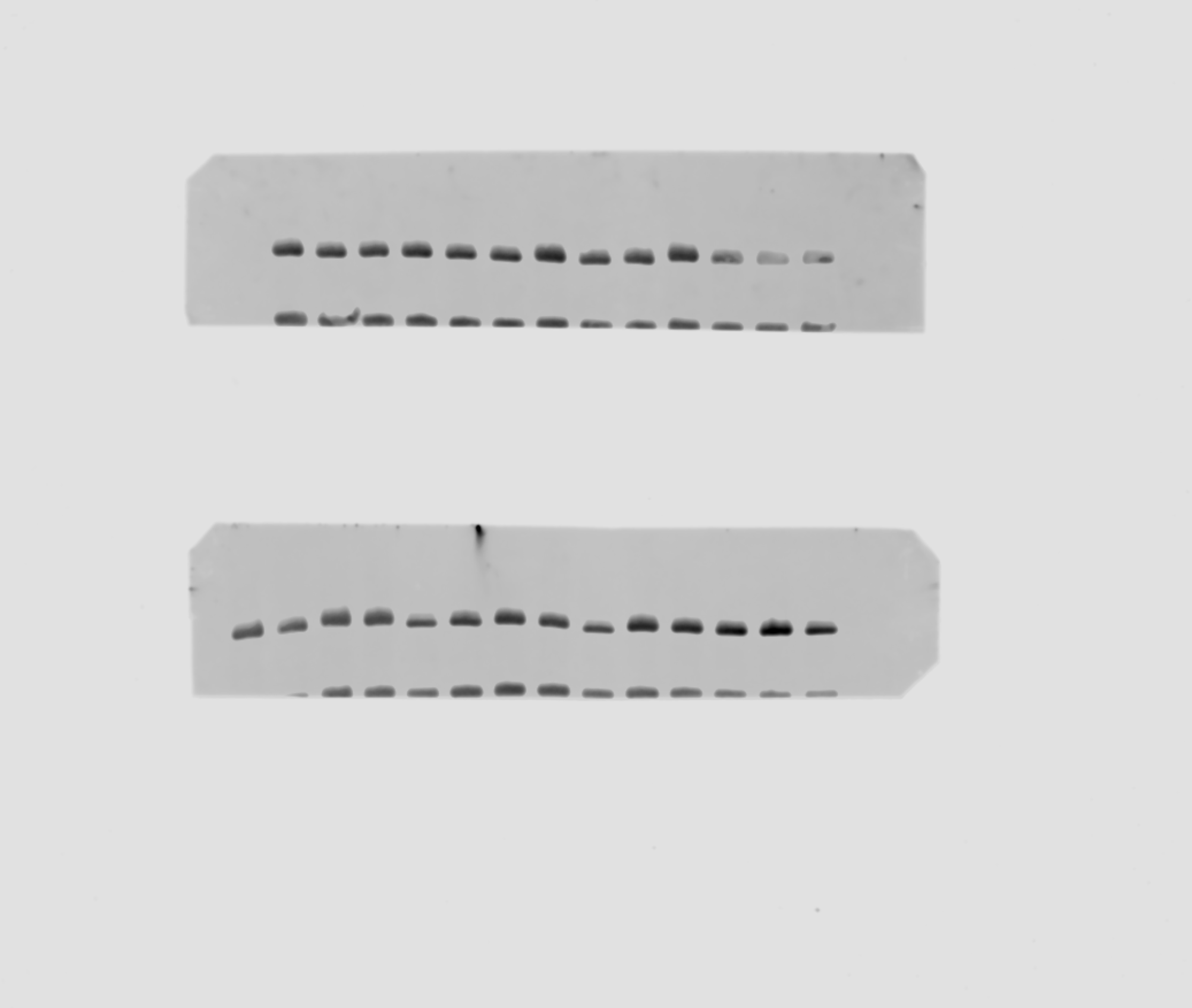

Supplement: Supplementary file 6 — Source data Fig. 5 [file 44321_2026_426_MOESM6_ESM.zip › Figure 5 updated/5A/F5A Females Brain CI a b.tif]

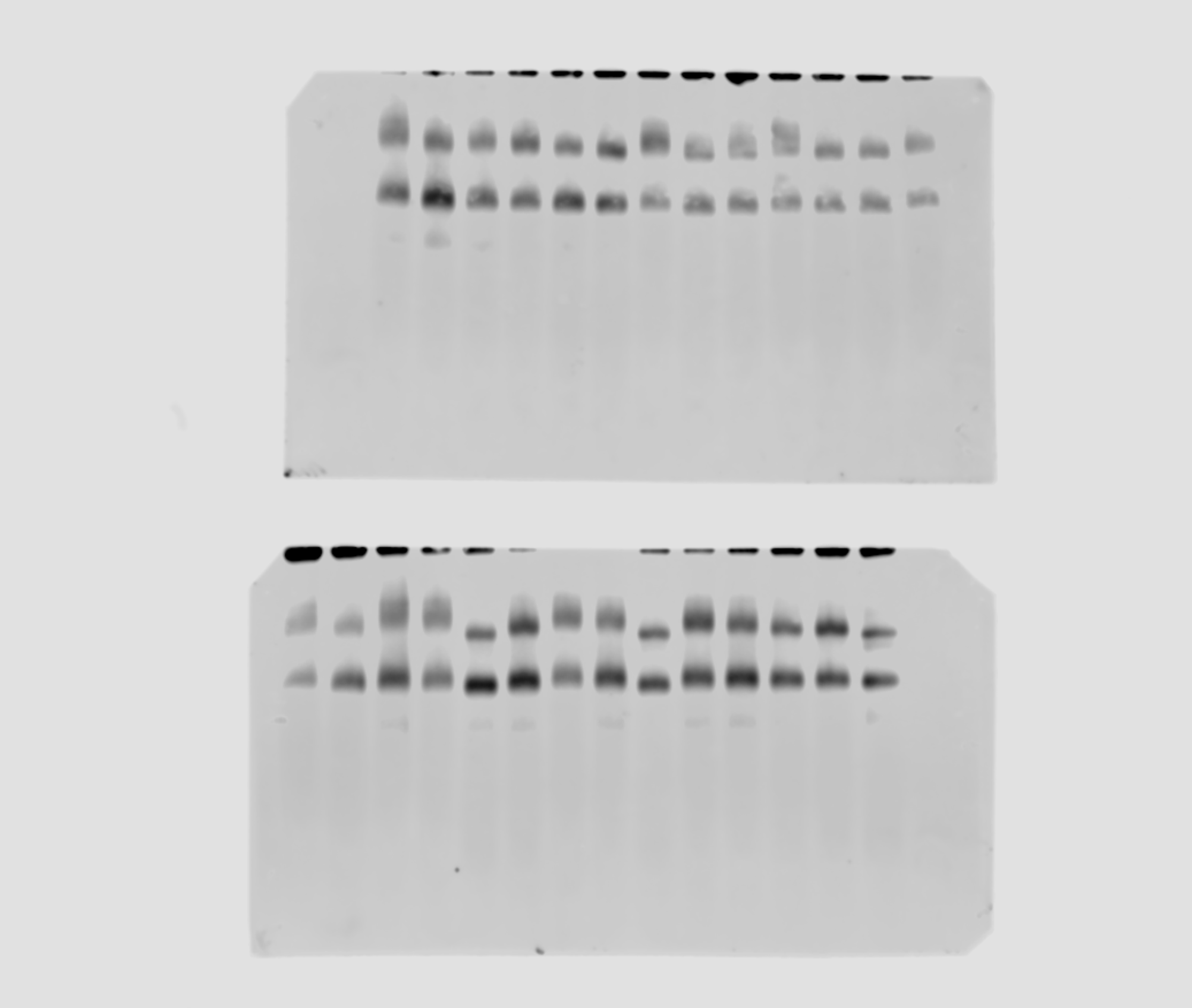

Supplement: Supplementary file 6 — Source data Fig. 5 [file 44321_2026_426_MOESM6_ESM.zip › Figure 5 updated/5A/F5A Females Brain CII a b.tif]

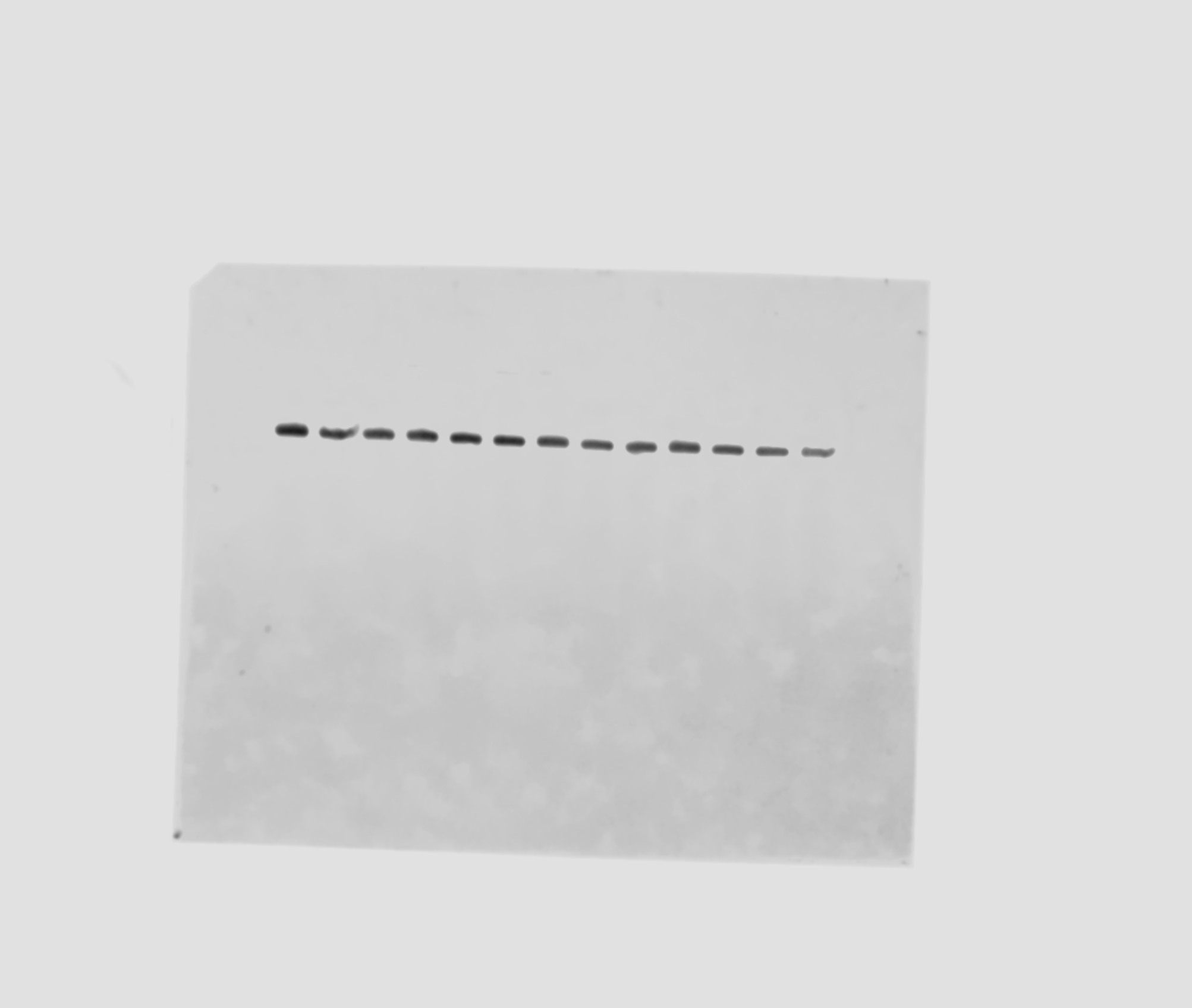

Supplement: Supplementary file 6 — Source data Fig. 5 [file 44321_2026_426_MOESM6_ESM.zip › Figure 5 updated/5A/F5A Females Brain CIII a.tif]

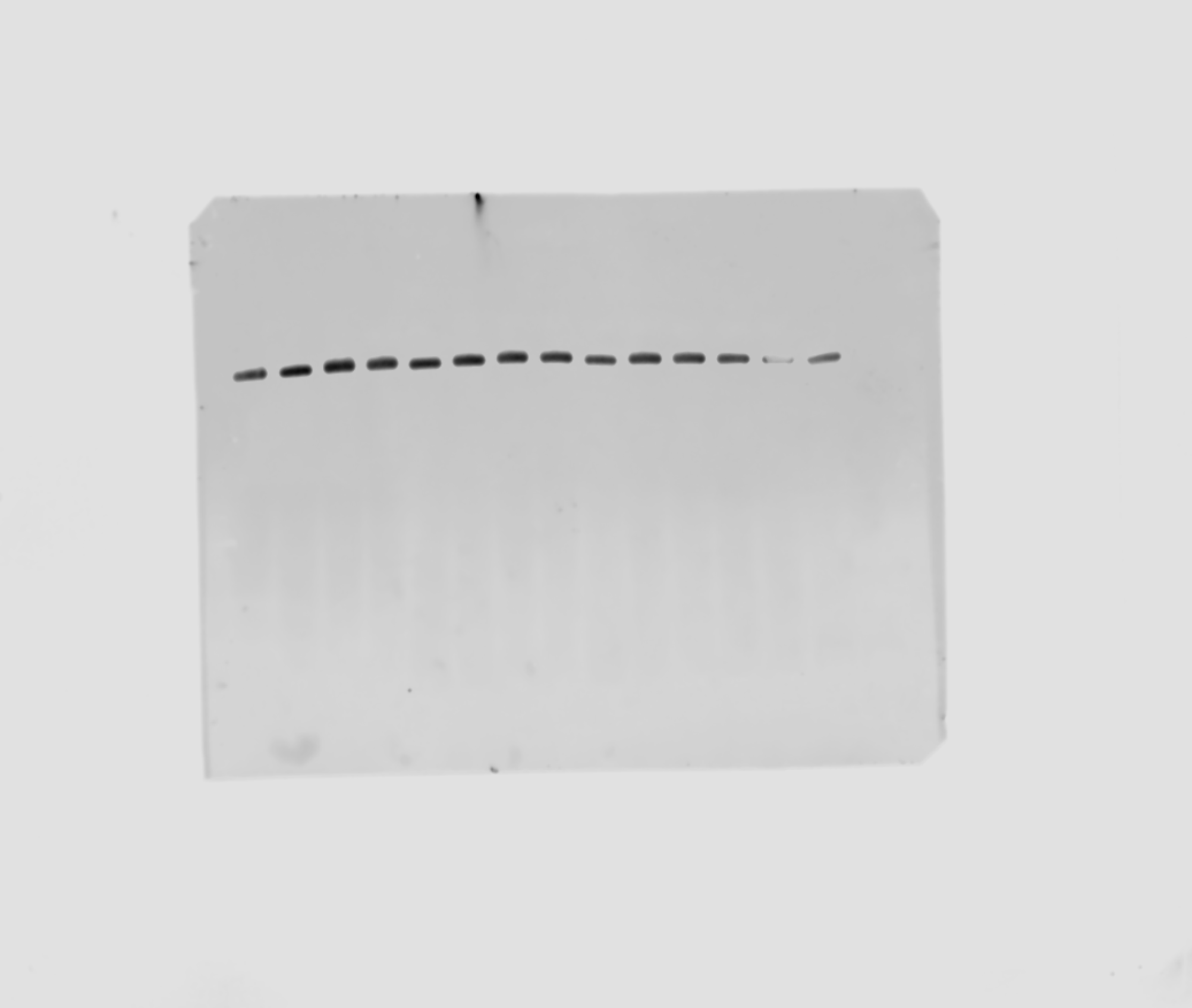

Supplement: Supplementary file 6 — Source data Fig. 5 [file 44321_2026_426_MOESM6_ESM.zip › Figure 5 updated/5A/F5A Females Brain CIII b.tif]

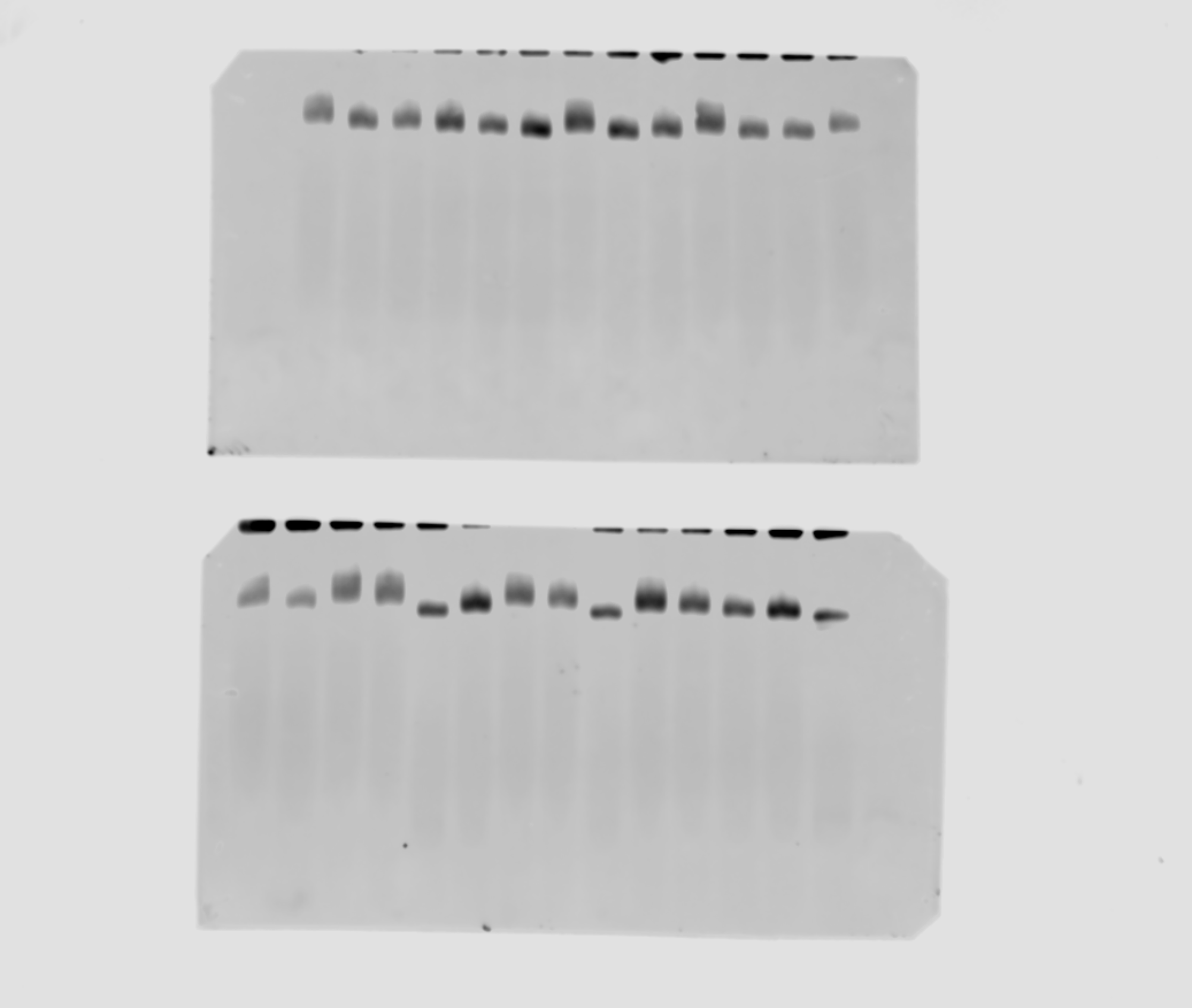

Supplement: Supplementary file 6 — Source data Fig. 5 [file 44321_2026_426_MOESM6_ESM.zip › Figure 5 updated/5A/F5A Females Brain CIV a b.tif]

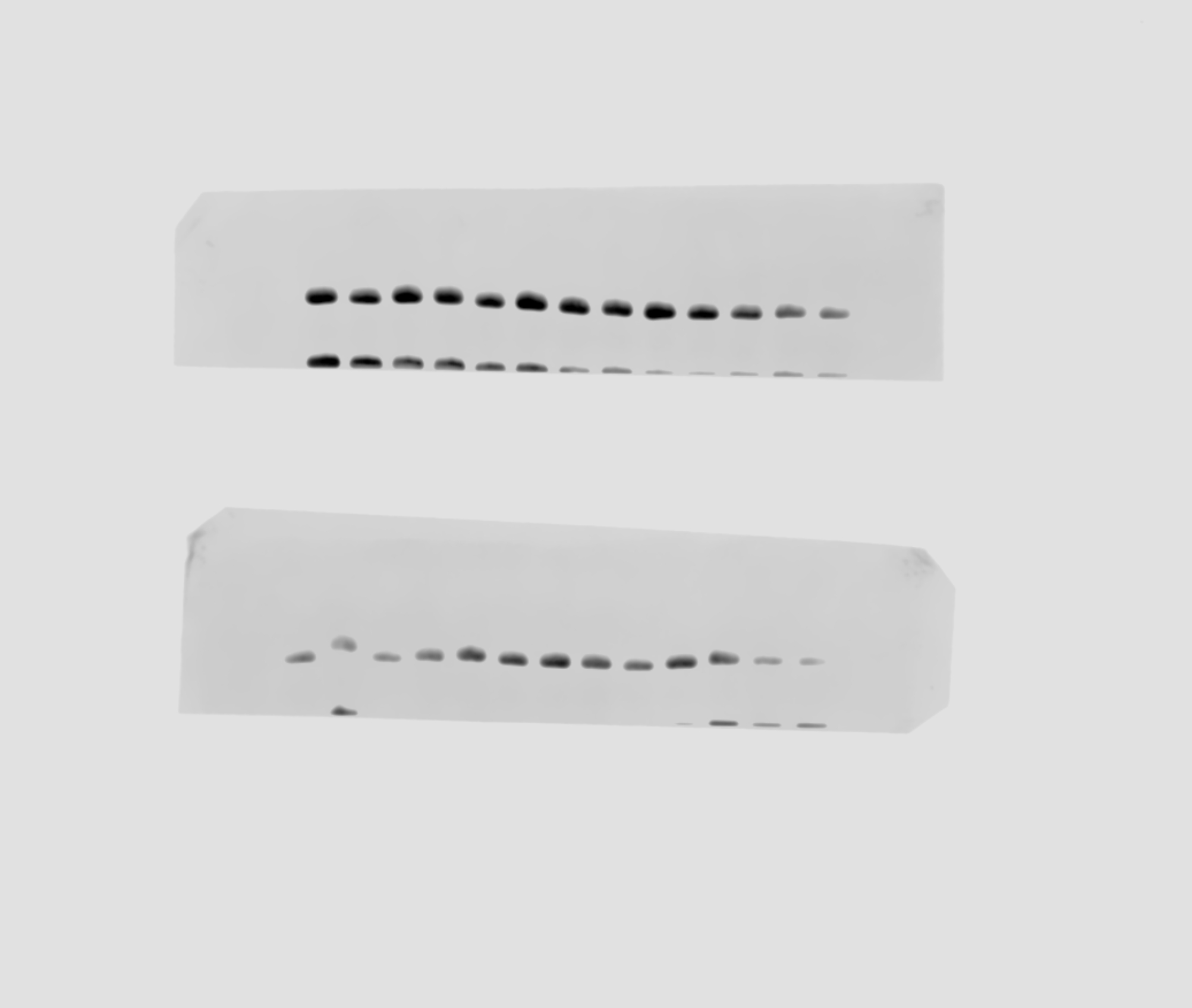

Supplement: Supplementary file 6 — Source data Fig. 5 [file 44321_2026_426_MOESM6_ESM.zip › Figure 5 updated/5A/F5A Males Brain CI a b.tif]

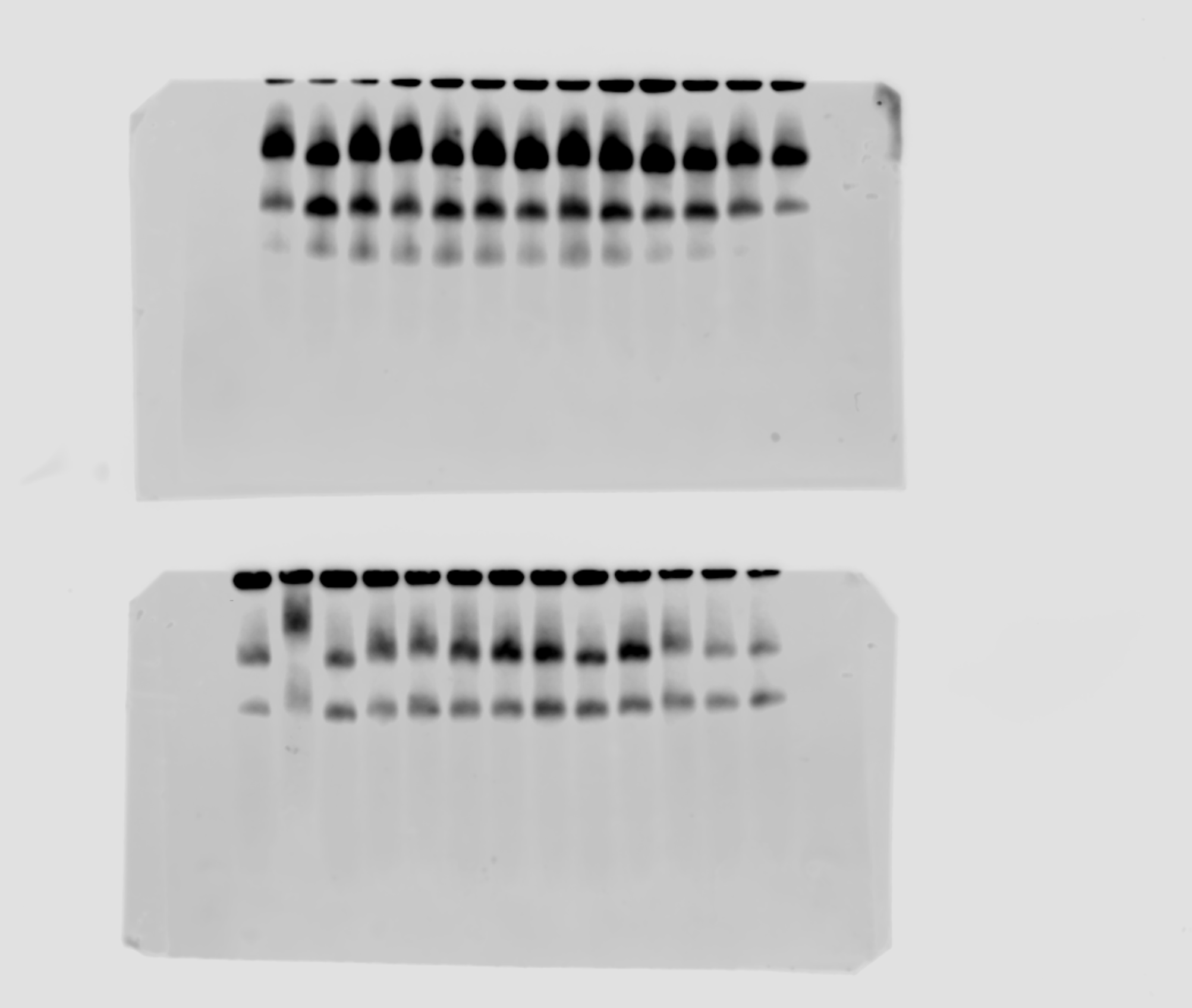

Supplement: Supplementary file 6 — Source data Fig. 5 [file 44321_2026_426_MOESM6_ESM.zip › Figure 5 updated/5A/F5A Males Brain CII a b.tif]

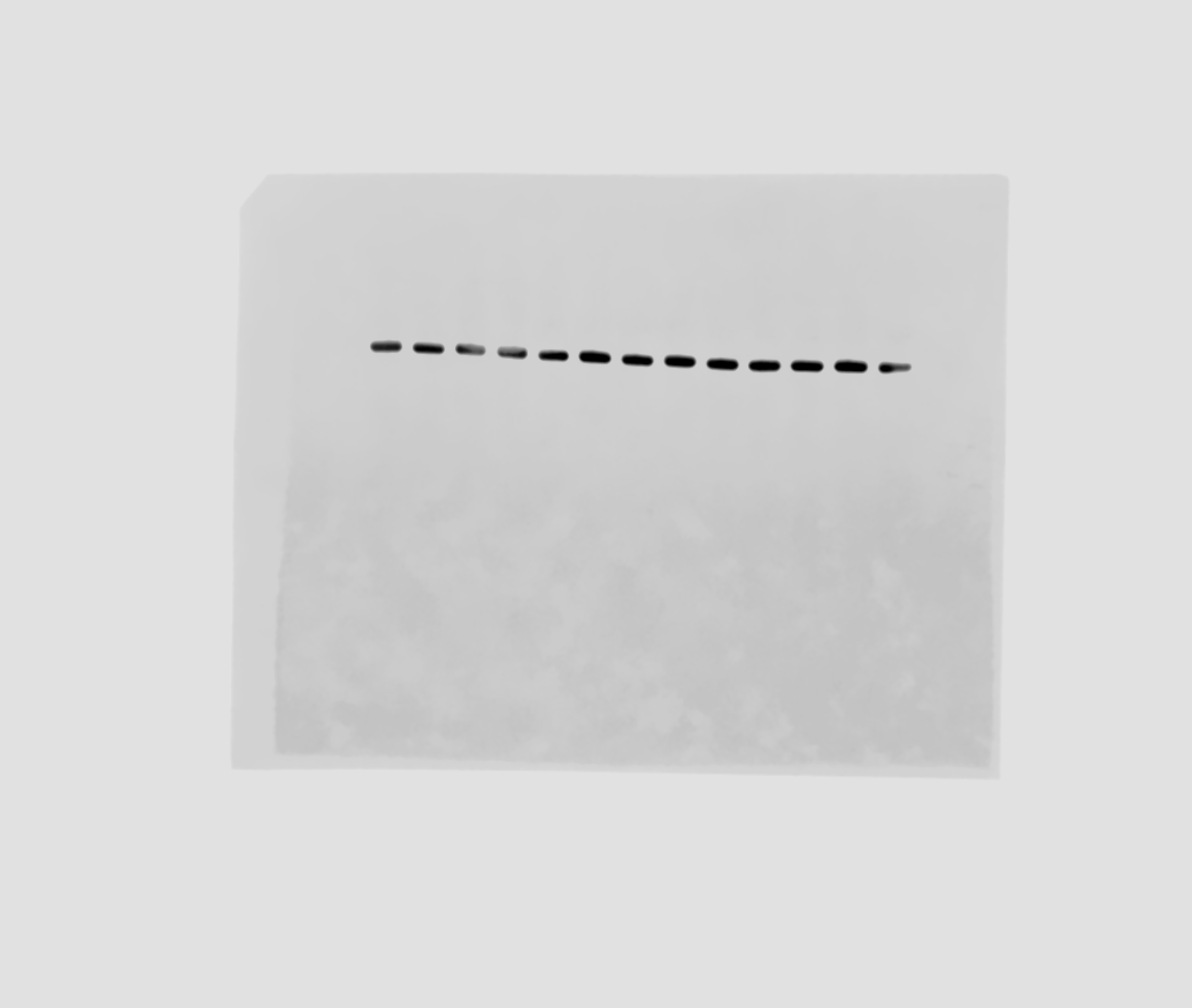

Supplement: Supplementary file 6 — Source data Fig. 5 [file 44321_2026_426_MOESM6_ESM.zip › Figure 5 updated/5A/F5A Males Brain CIII a.tif]

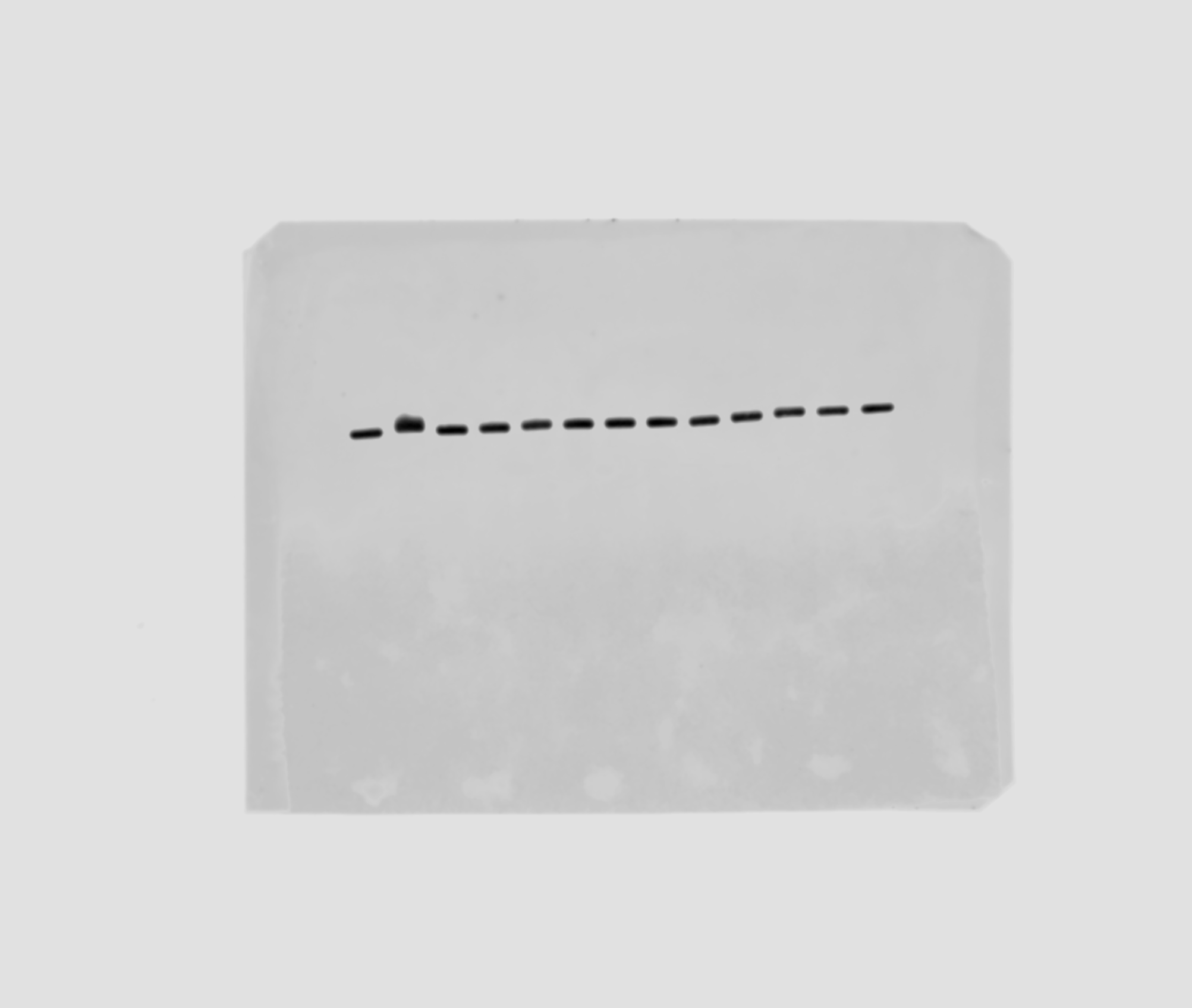

Supplement: Supplementary file 6 — Source data Fig. 5 [file 44321_2026_426_MOESM6_ESM.zip › Figure 5 updated/5A/F5A Males Brain CIII b.tif]

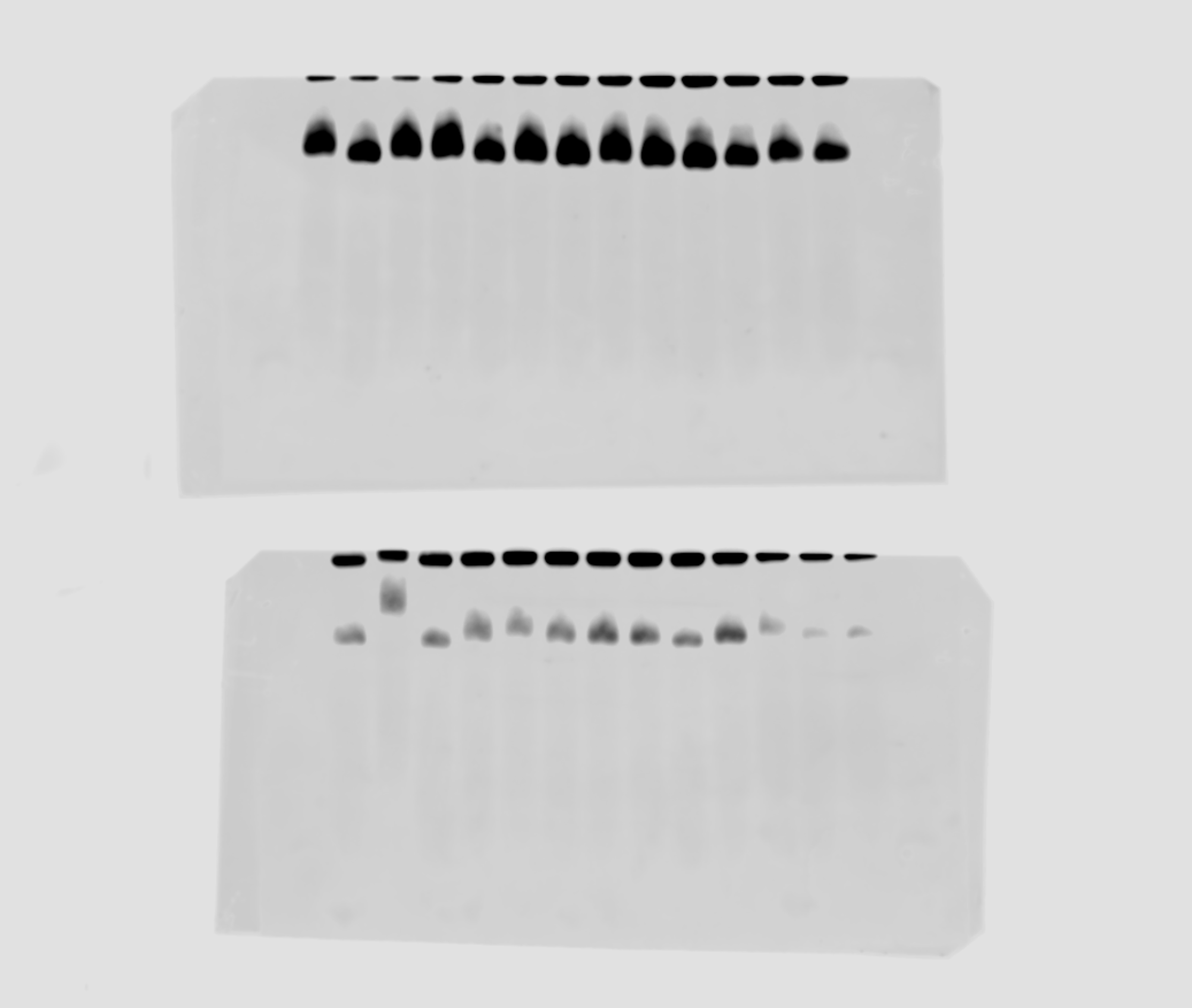

Supplement: Supplementary file 6 — Source data Fig. 5 [file 44321_2026_426_MOESM6_ESM.zip › Figure 5 updated/5A/F5A Males Brain CIV a b.tif]

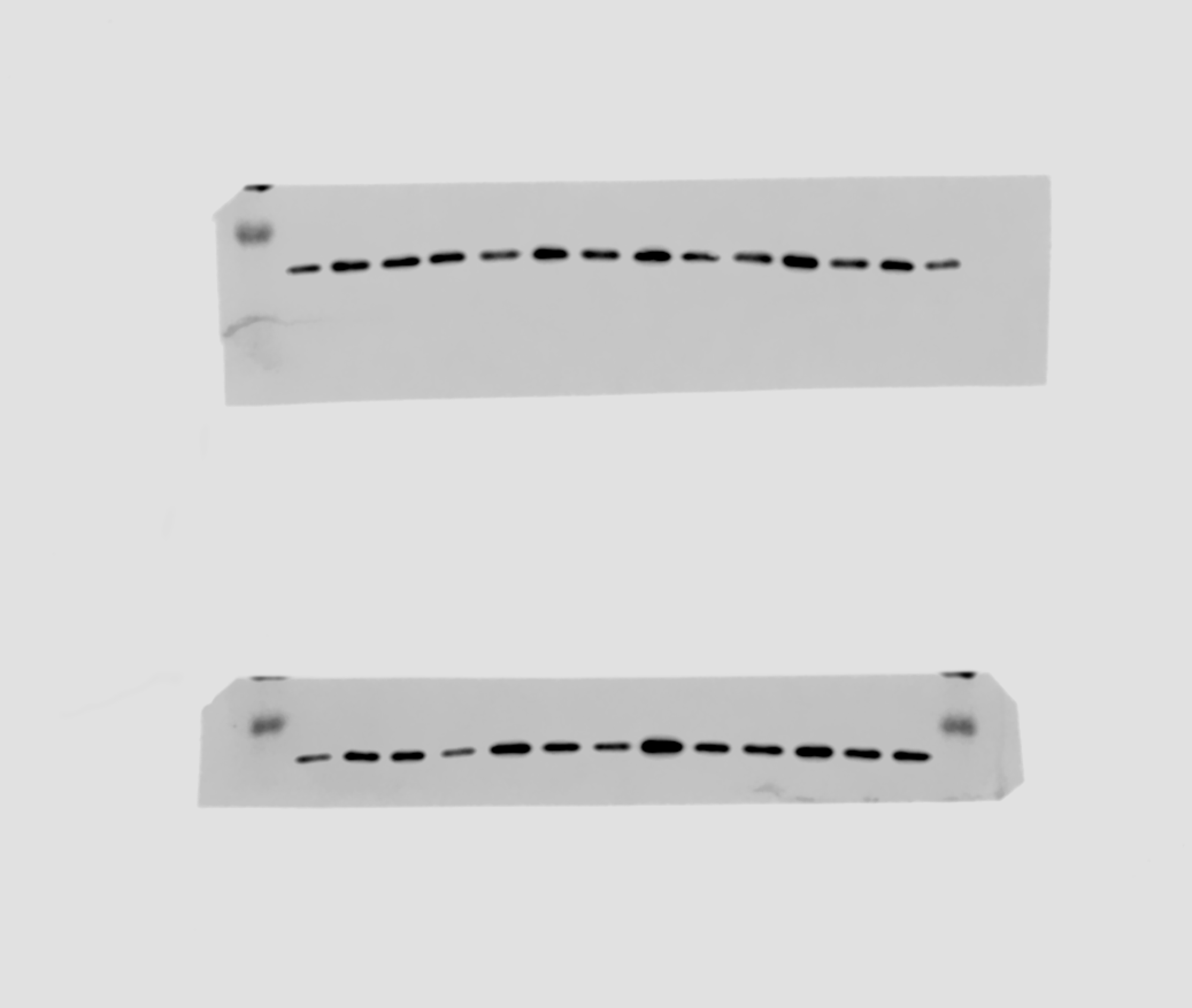

Supplement: Supplementary file 7 — Source data Fig. 6 [file 44321_2026_426_MOESM7_ESM.zip › Figure 6 updated/6A/F6A Females Liver MRPL13 a b.tif]

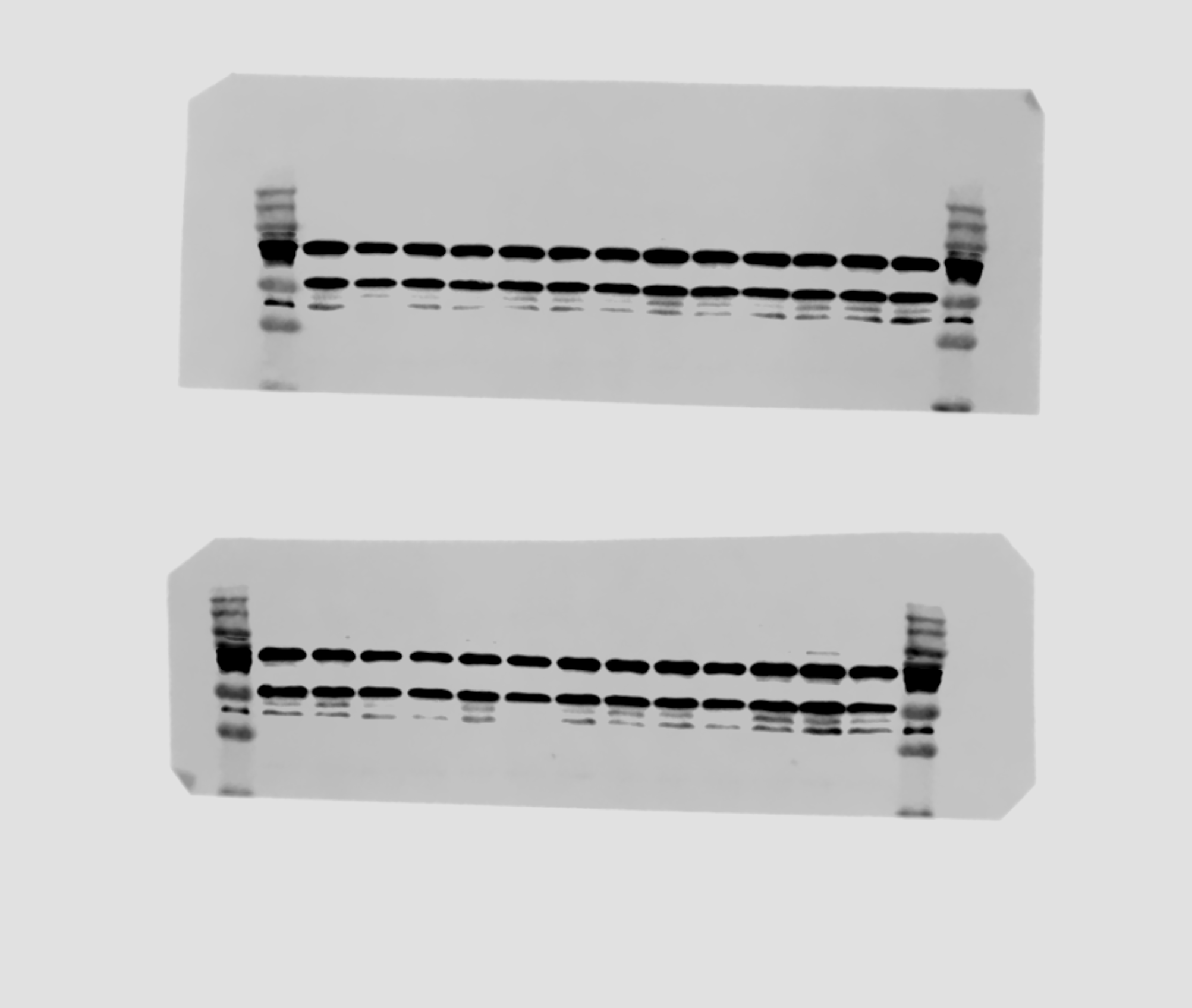

Supplement: Supplementary file 7 — Source data Fig. 6 [file 44321_2026_426_MOESM7_ESM.zip › Figure 6 updated/6A/F6A Females Liver MRPL37 e f.tif]

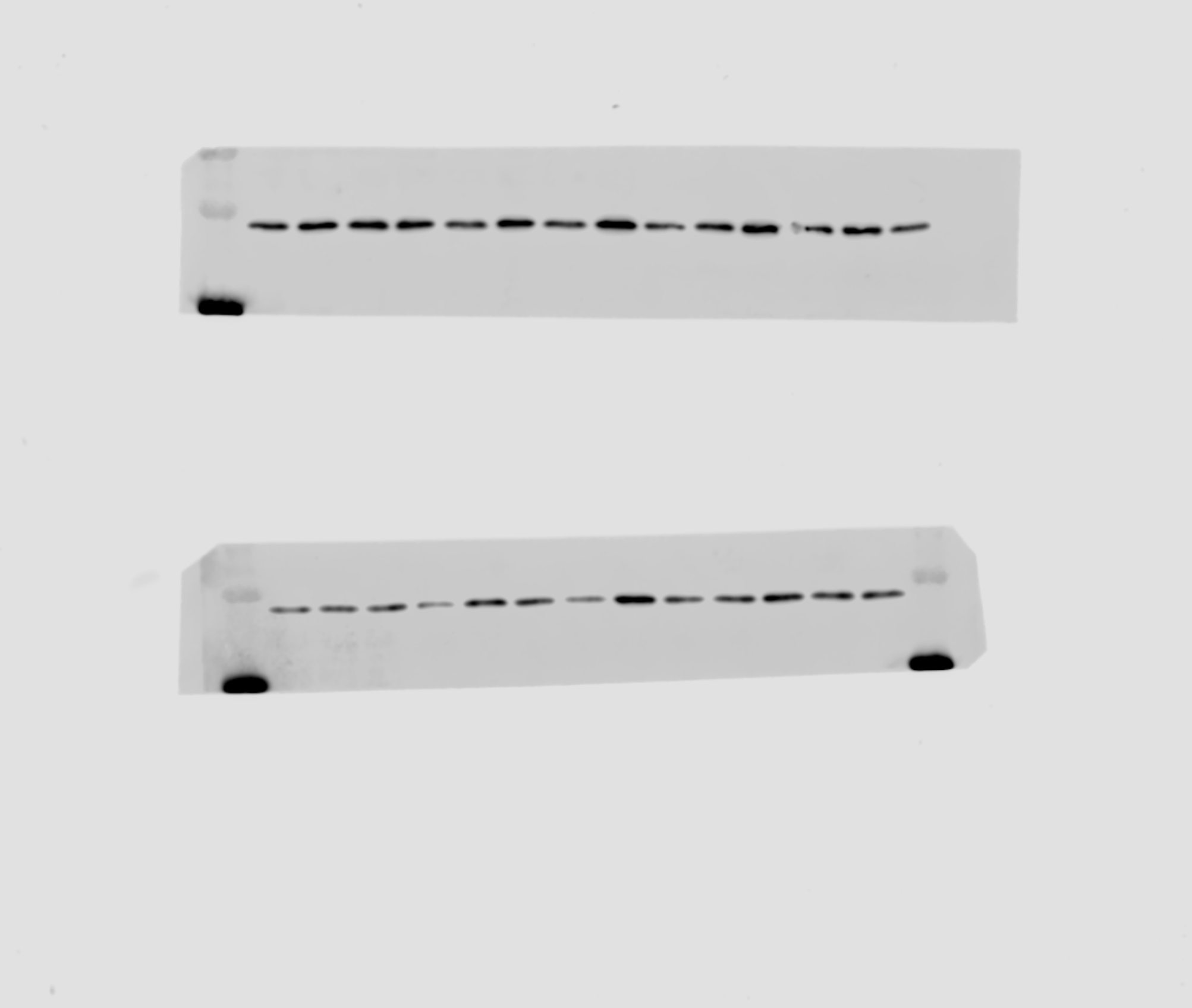

Supplement: Supplementary file 7 — Source data Fig. 6 [file 44321_2026_426_MOESM7_ESM.zip › Figure 6 updated/6A/F6A Females Liver MRPS35 a b.tif]

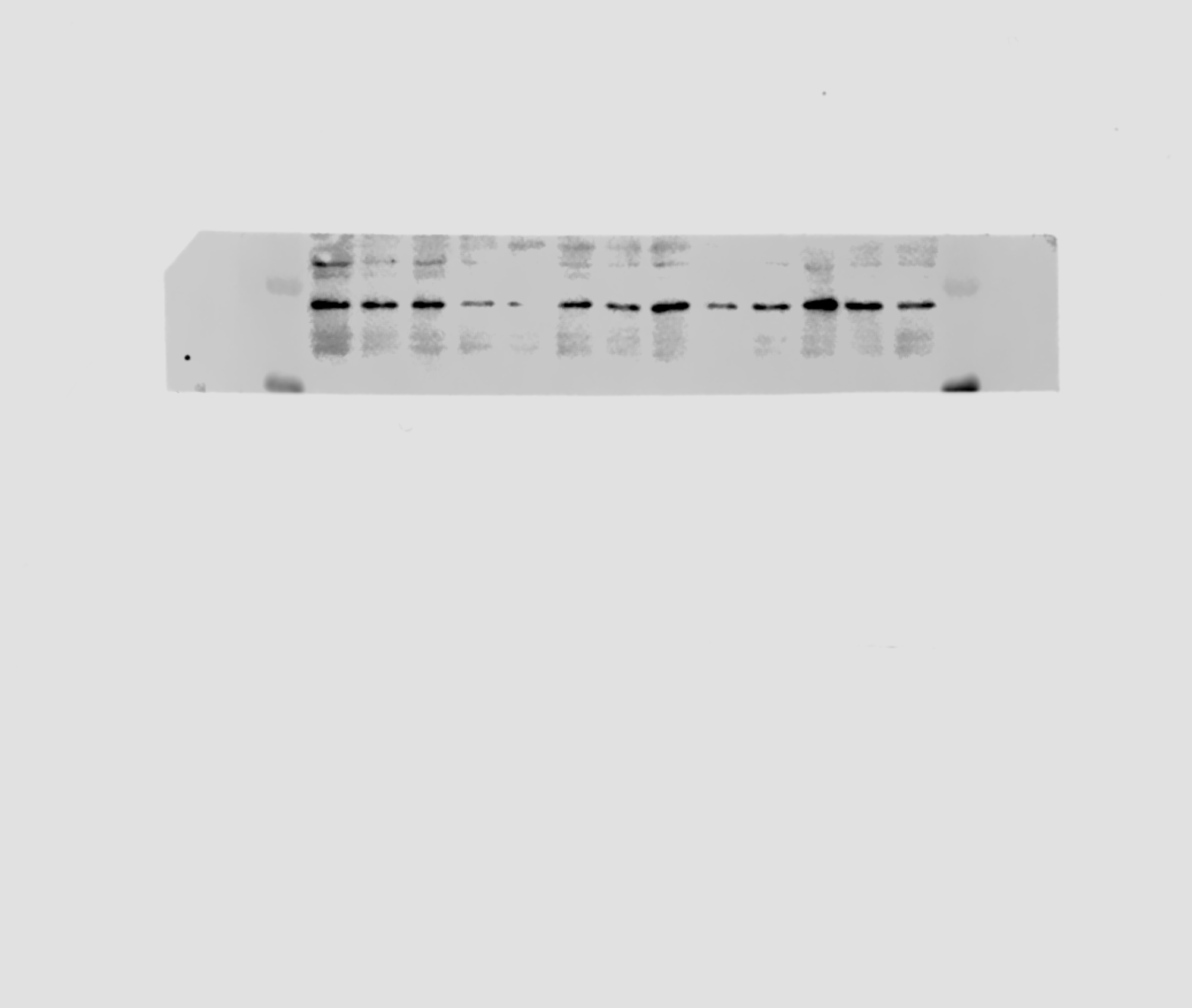

Supplement: Supplementary file 7 — Source data Fig. 6 [file 44321_2026_426_MOESM7_ESM.zip › Figure 6 updated/6A/F6A Females Liver MRPS9 c.tif]

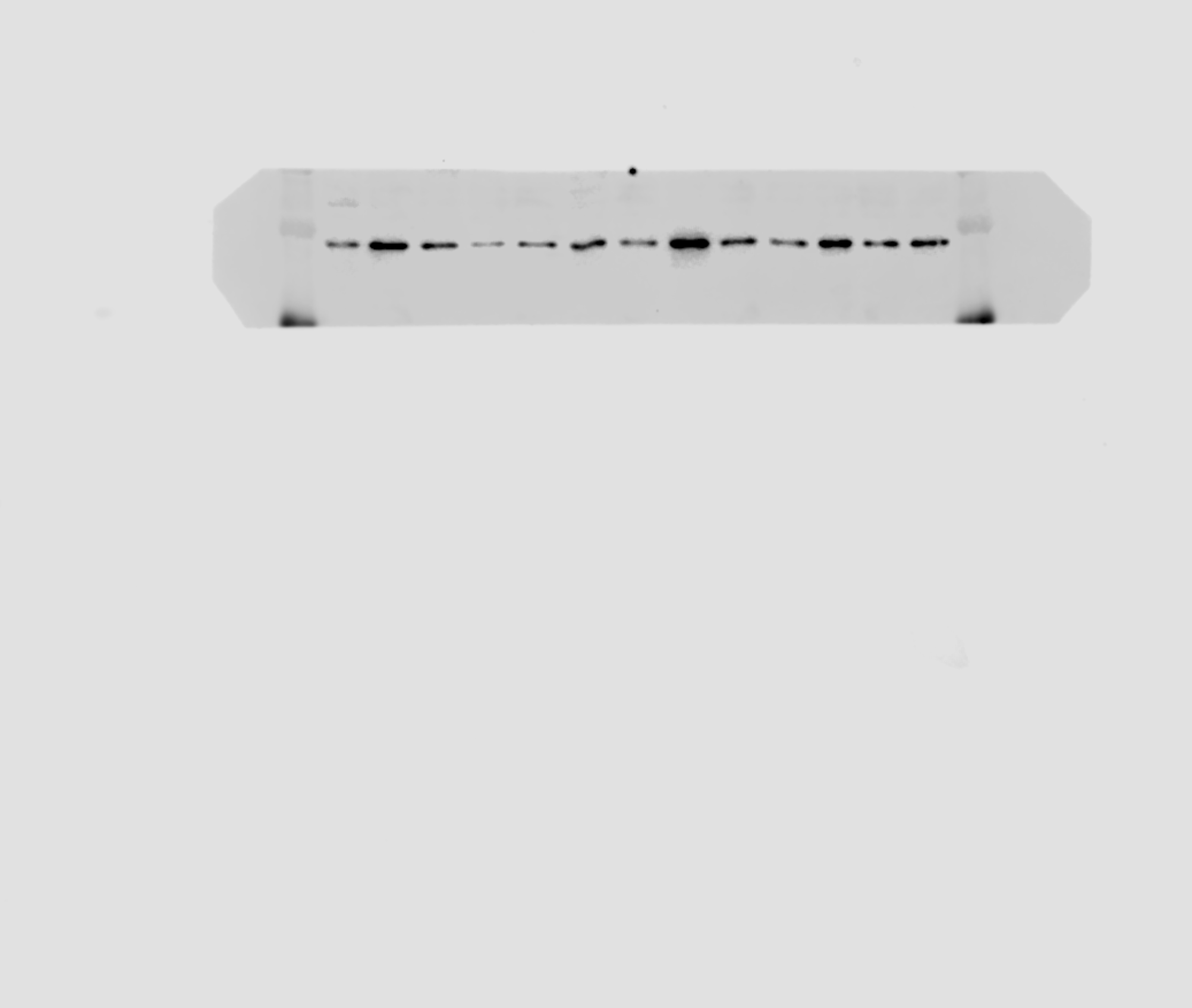

Supplement: Supplementary file 7 — Source data Fig. 6 [file 44321_2026_426_MOESM7_ESM.zip › Figure 6 updated/6A/F6A Females Liver MRPS9 d.tif]

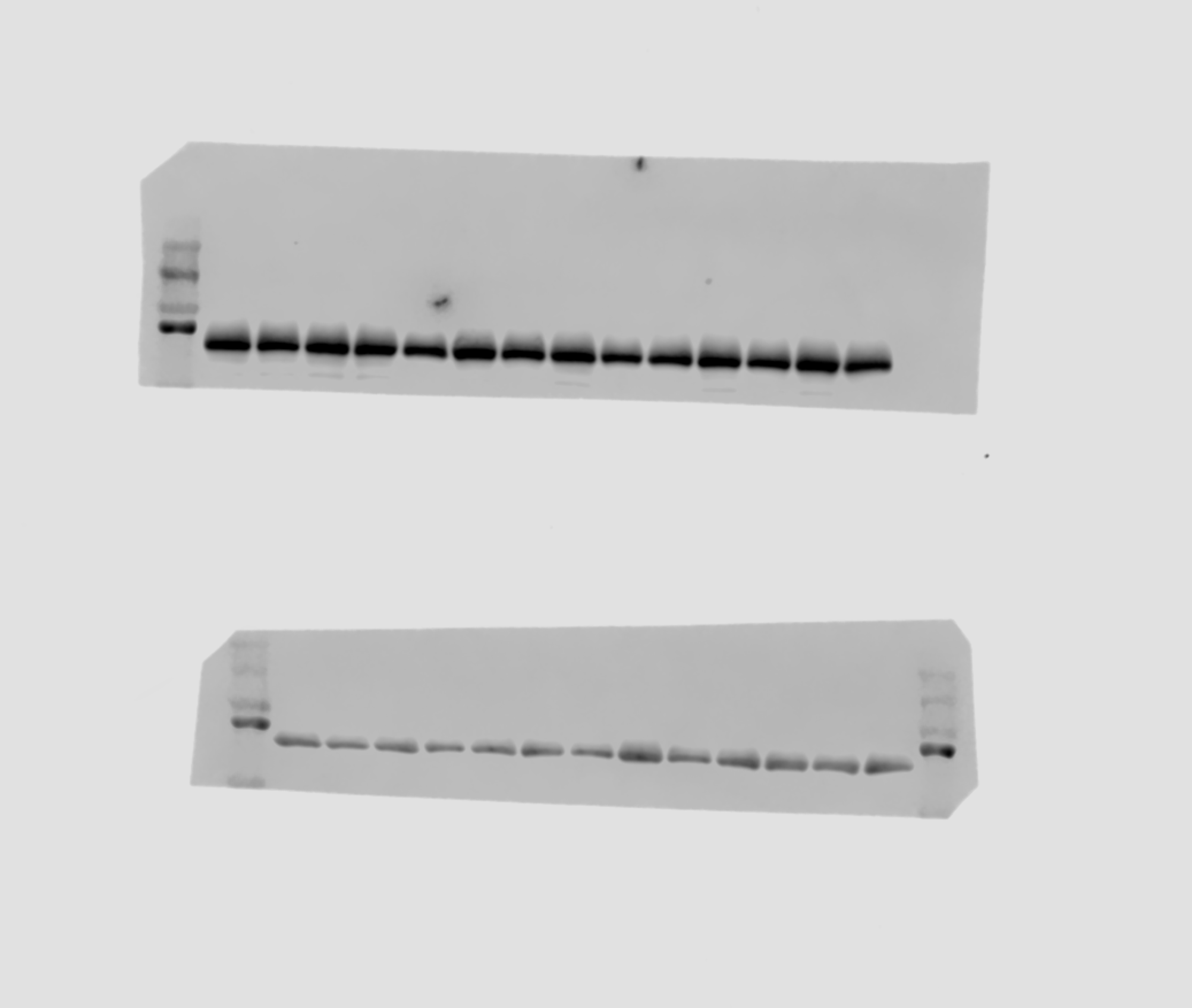

Supplement: Supplementary file 7 — Source data Fig. 6 [file 44321_2026_426_MOESM7_ESM.zip › Figure 6 updated/6A/F6A Females Liver SDHA a b.tif]

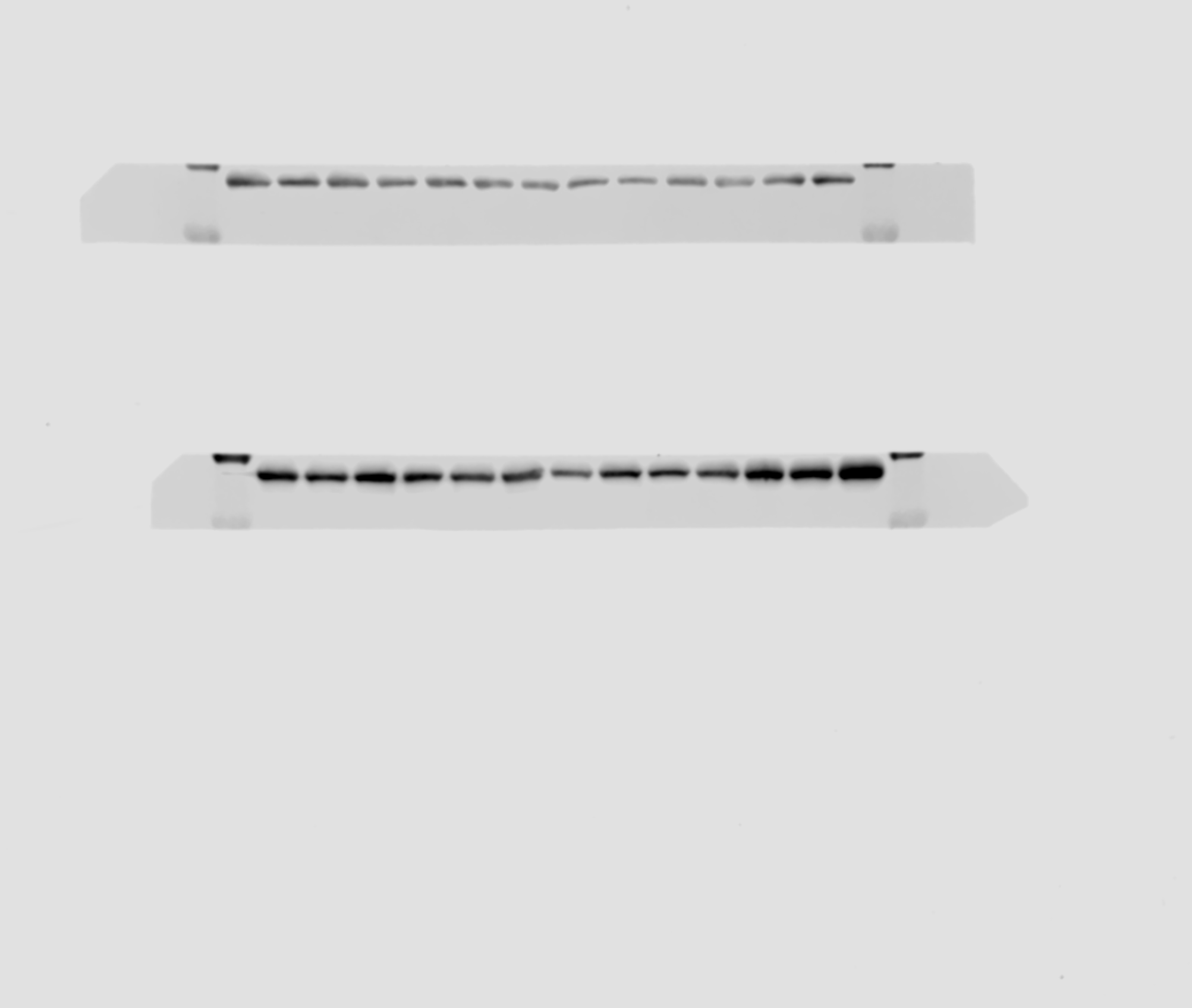

Supplement: Supplementary file 7 — Source data Fig. 6 [file 44321_2026_426_MOESM7_ESM.zip › Figure 6 updated/6A/F6A Females Liver SDHA c d.tif]

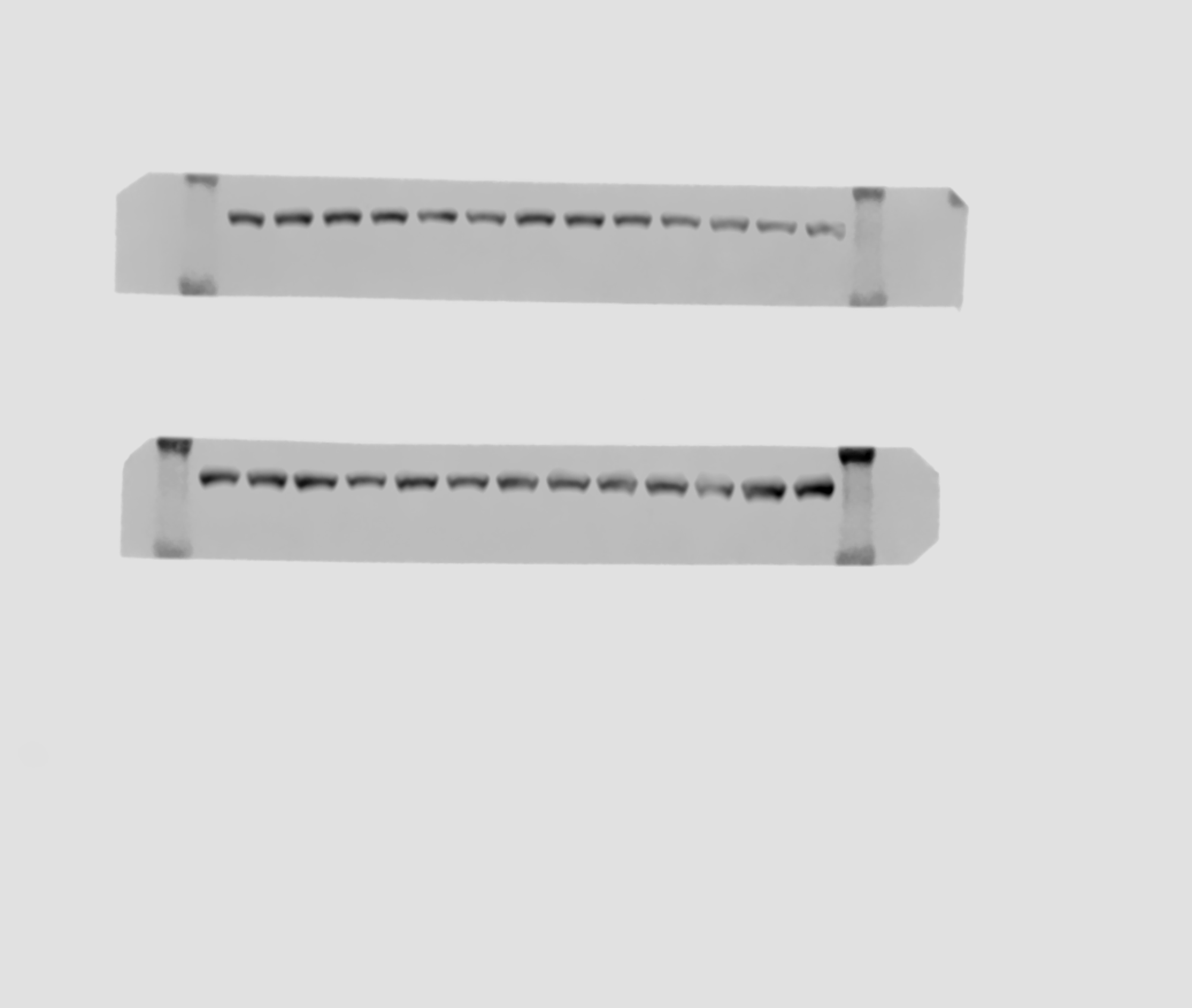

Supplement: Supplementary file 7 — Source data Fig. 6 [file 44321_2026_426_MOESM7_ESM.zip › Figure 6 updated/6A/F6A Females Liver SDHA e f.tif]

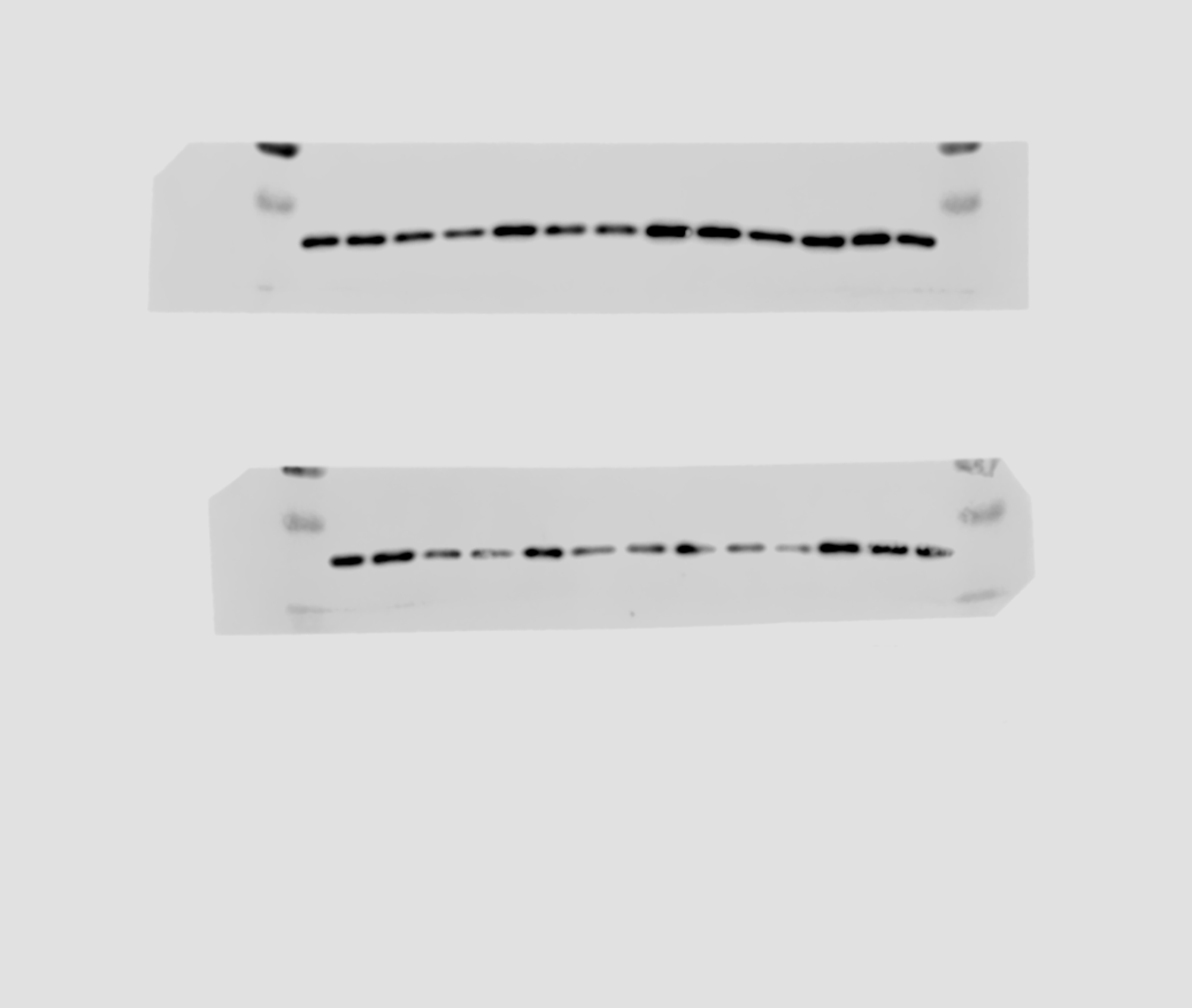

Supplement: Supplementary file 7 — Source data Fig. 6 [file 44321_2026_426_MOESM7_ESM.zip › Figure 6 updated/6A/F6A Males Liver MRPL13 a b.tif]

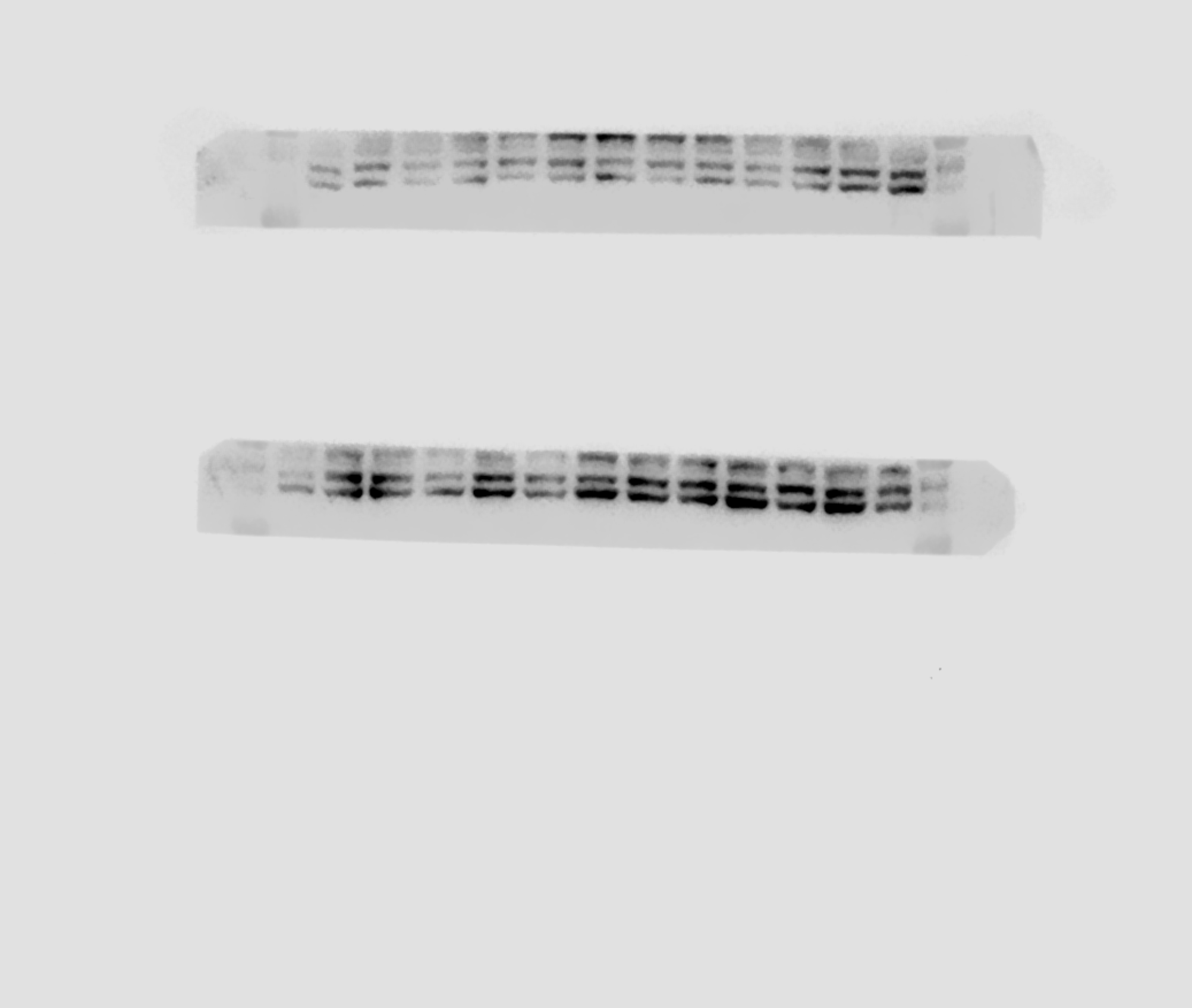

Supplement: Supplementary file 7 — Source data Fig. 6 [file 44321_2026_426_MOESM7_ESM.zip › Figure 6 updated/6A/F6A Males Liver MRPL37 e f.tif]

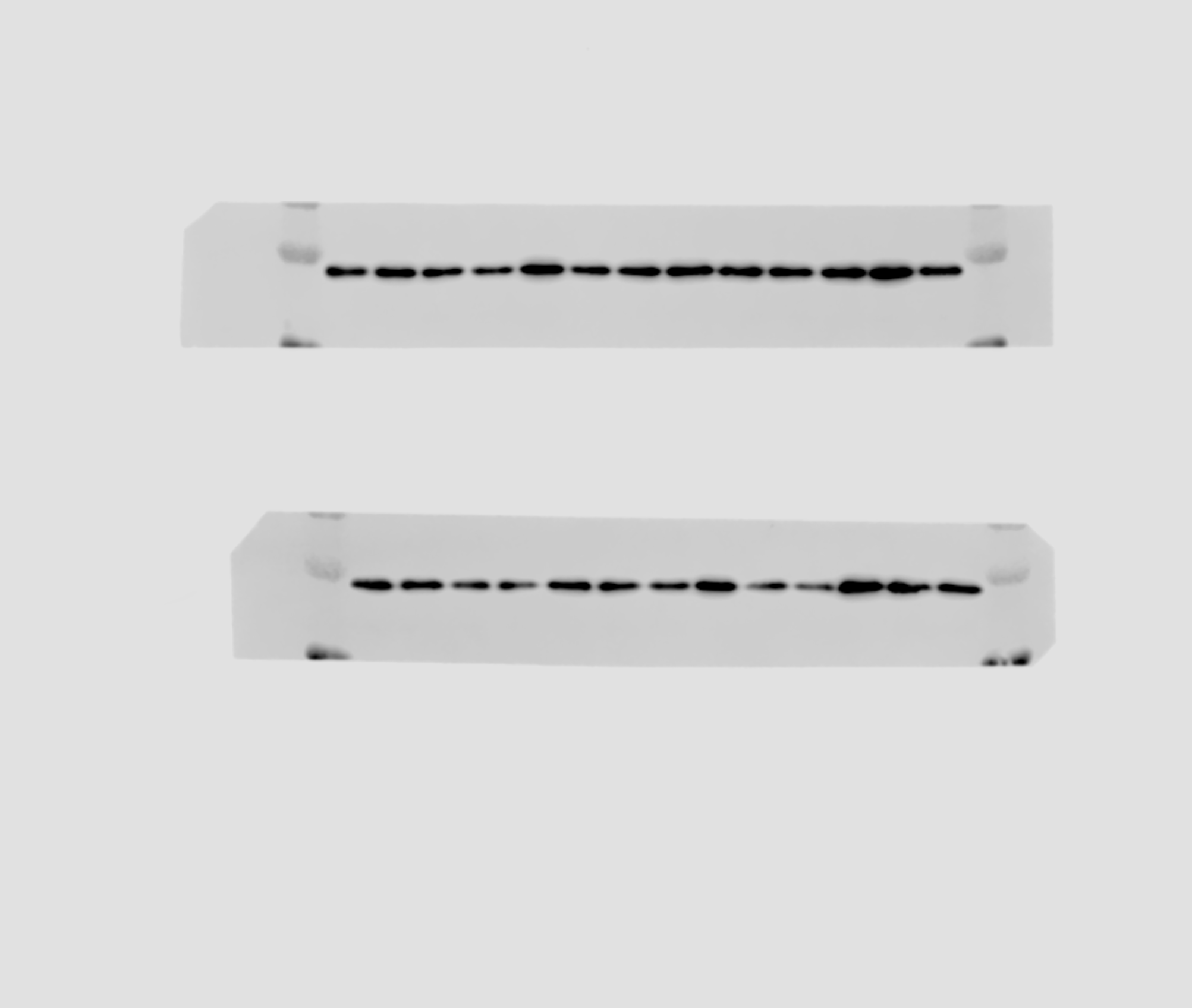

Supplement: Supplementary file 7 — Source data Fig. 6 [file 44321_2026_426_MOESM7_ESM.zip › Figure 6 updated/6A/F6A Males Liver MRPS35 a b.tif]

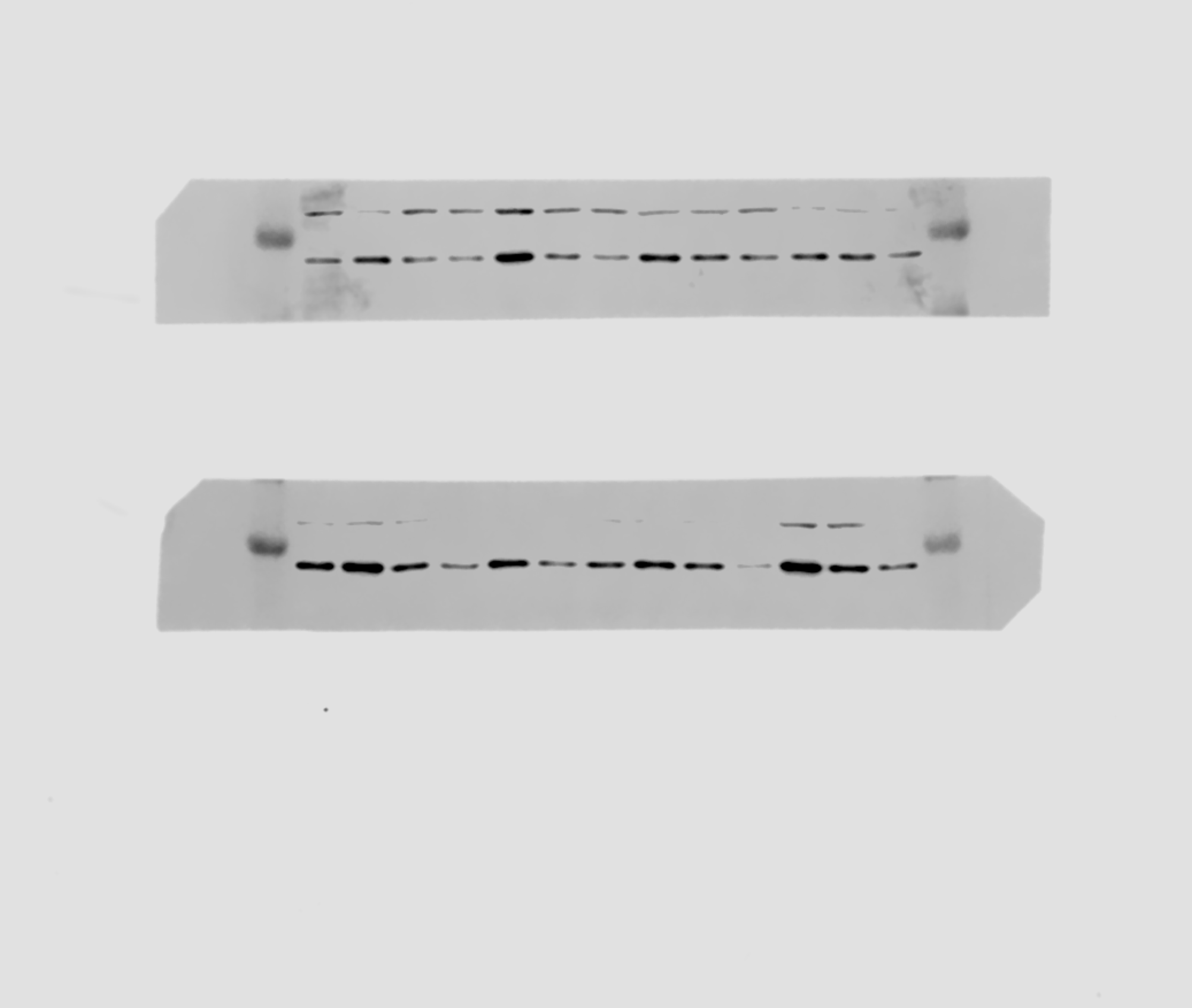

Supplement: Supplementary file 7 — Source data Fig. 6 [file 44321_2026_426_MOESM7_ESM.zip › Figure 6 updated/6A/F6A Males Liver MRPS9 c d.tif]

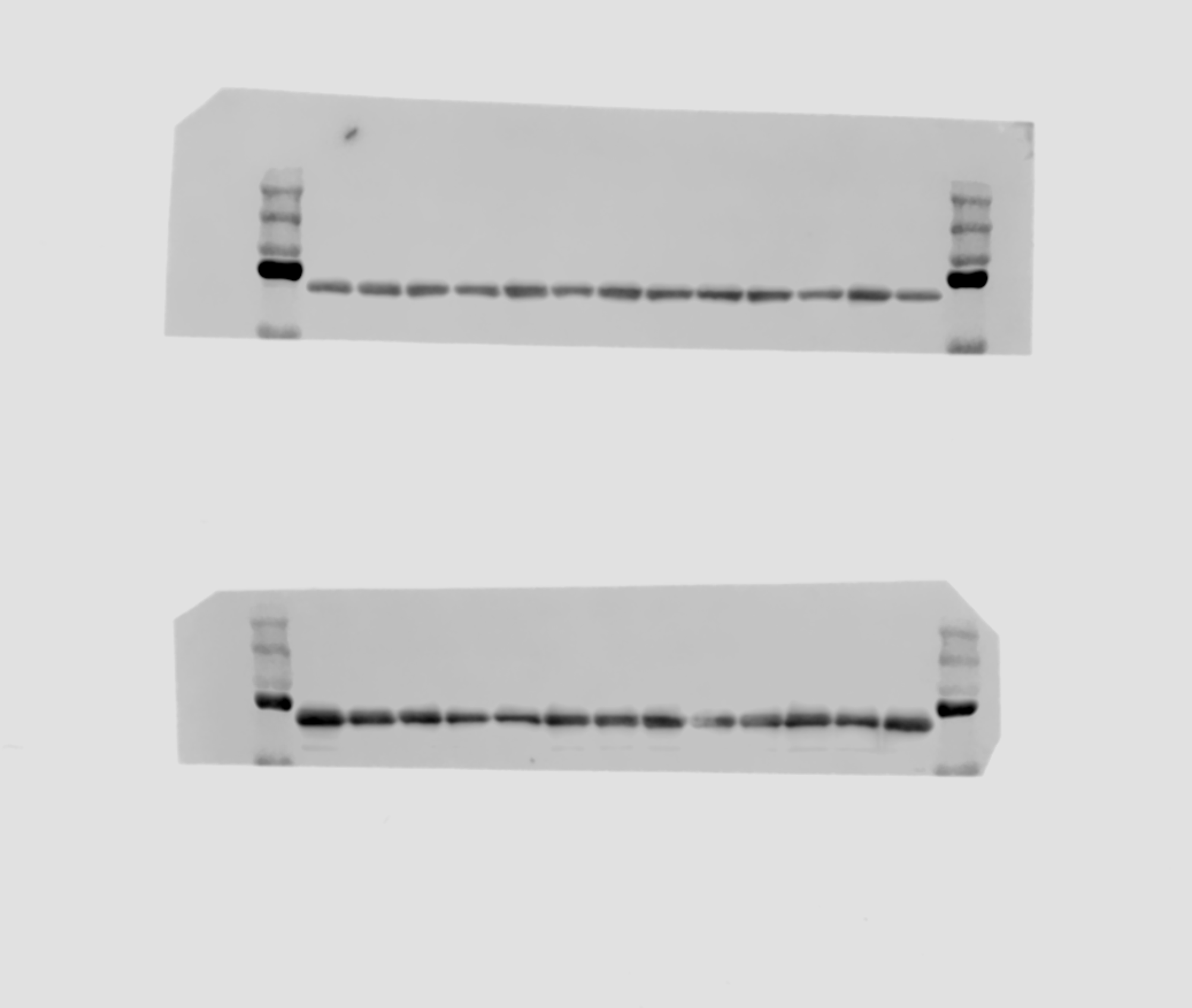

Supplement: Supplementary file 7 — Source data Fig. 6 [file 44321_2026_426_MOESM7_ESM.zip › Figure 6 updated/6A/F6A Males Liver SDHA a b.tif]

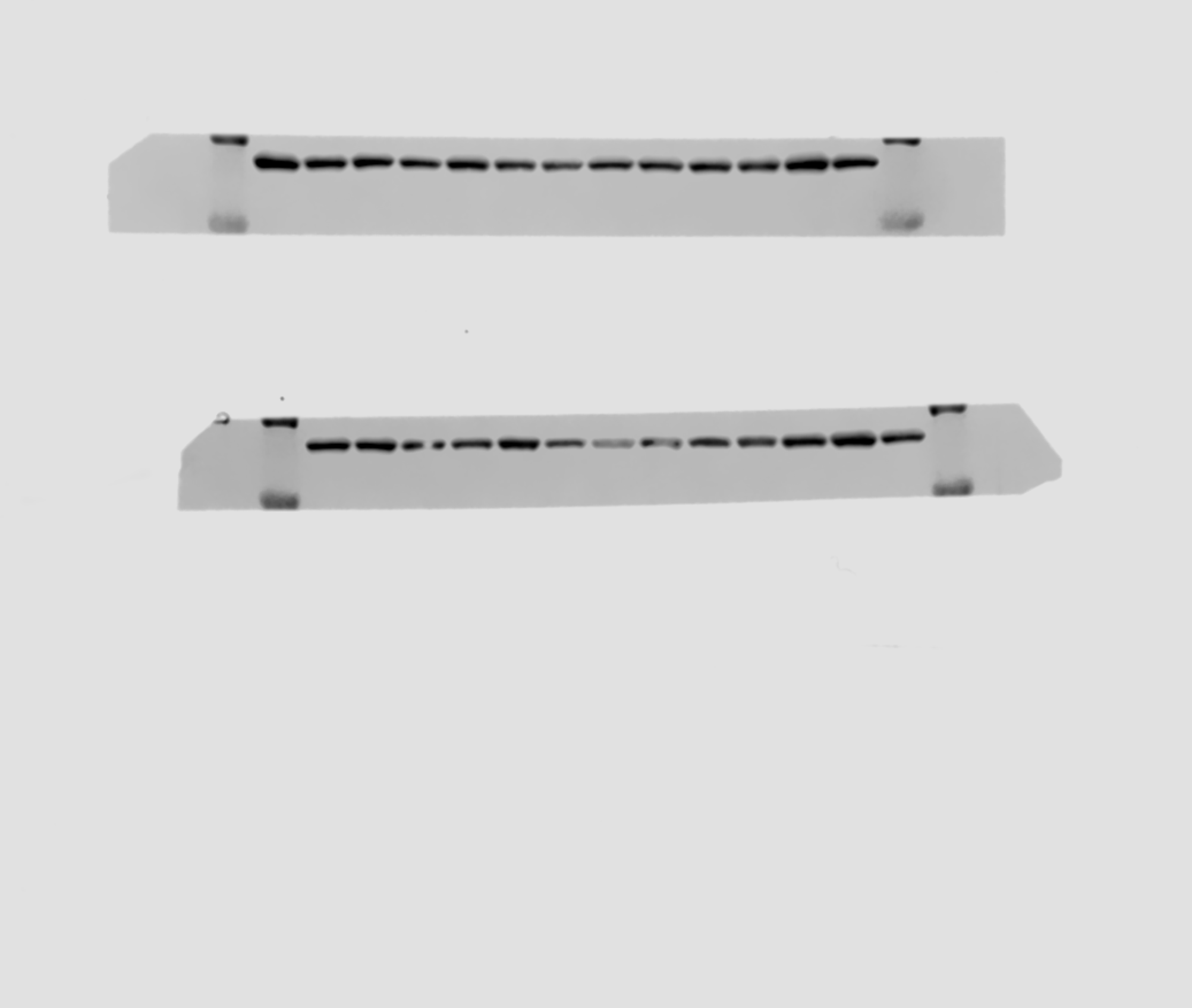

Supplement: Supplementary file 7 — Source data Fig. 6 [file 44321_2026_426_MOESM7_ESM.zip › Figure 6 updated/6A/F6A Males Liver SDHA c d.tif]

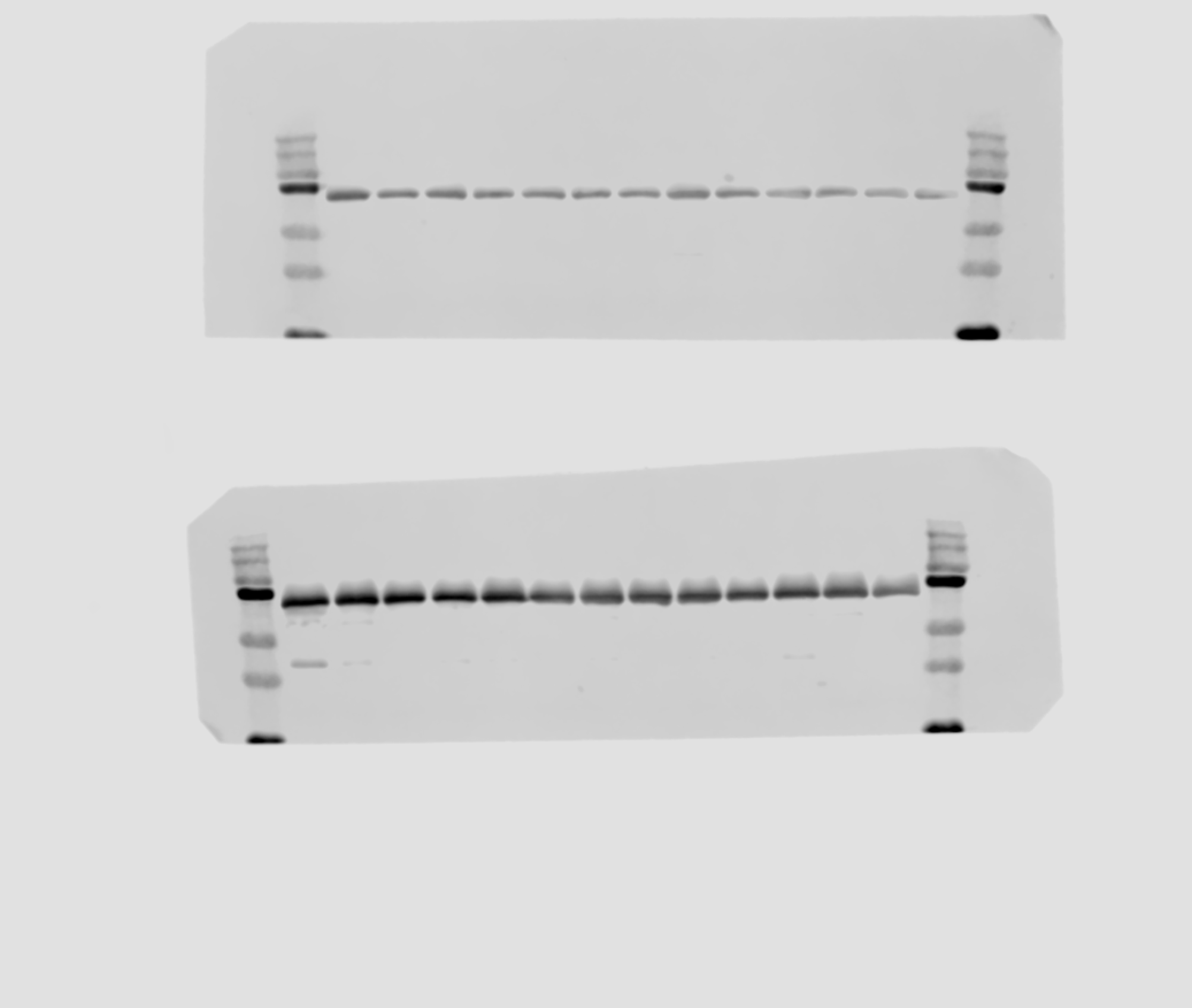

Supplement: Supplementary file 7 — Source data Fig. 6 [file 44321_2026_426_MOESM7_ESM.zip › Figure 6 updated/6A/F6A Males Liver SDHA e f.tif]

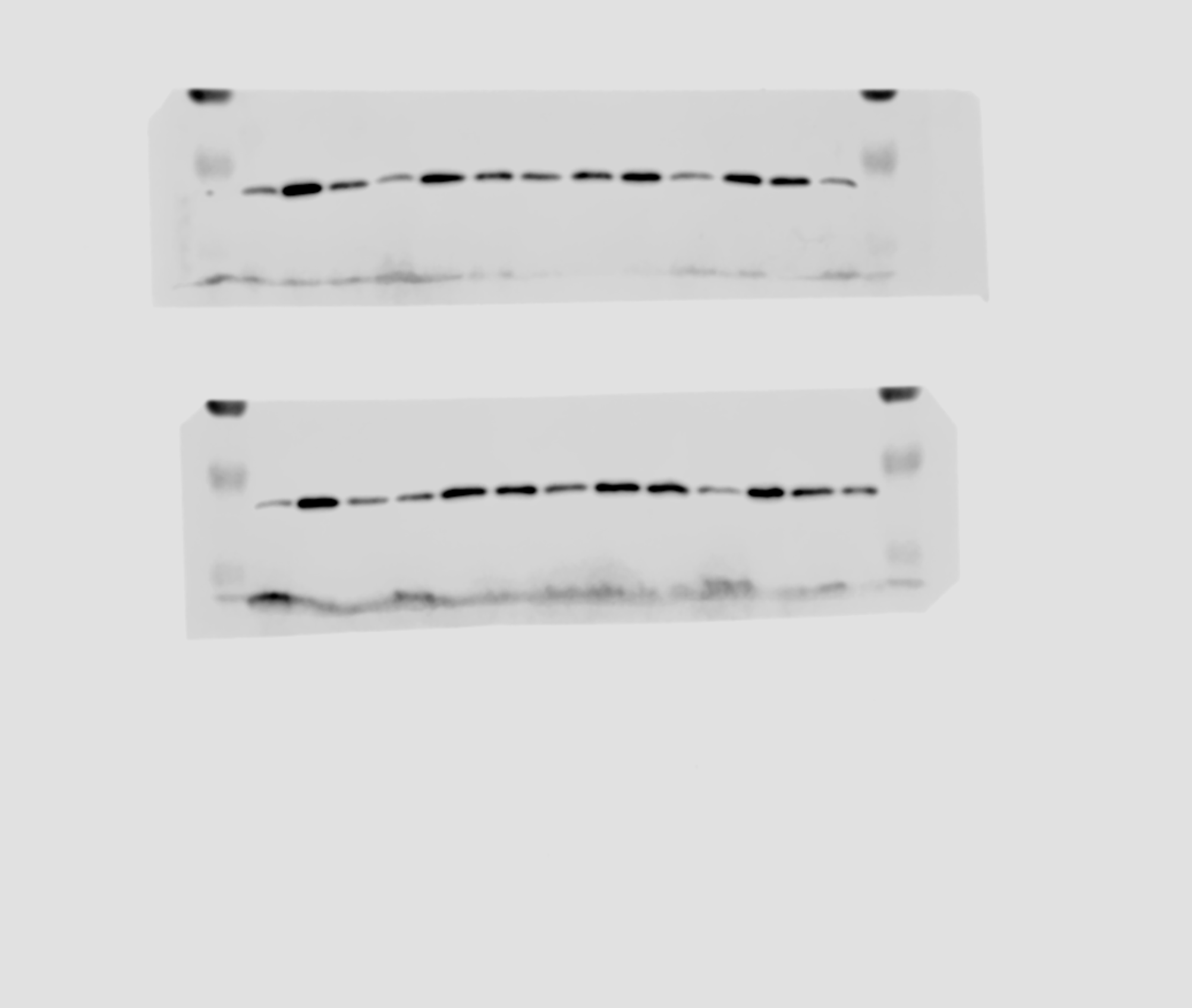

Supplement: Supplementary file 7 — Source data Fig. 6 [file 44321_2026_426_MOESM7_ESM.zip › Figure 6 updated/6B/F6B Females Brain MRPL13 e f.tif]

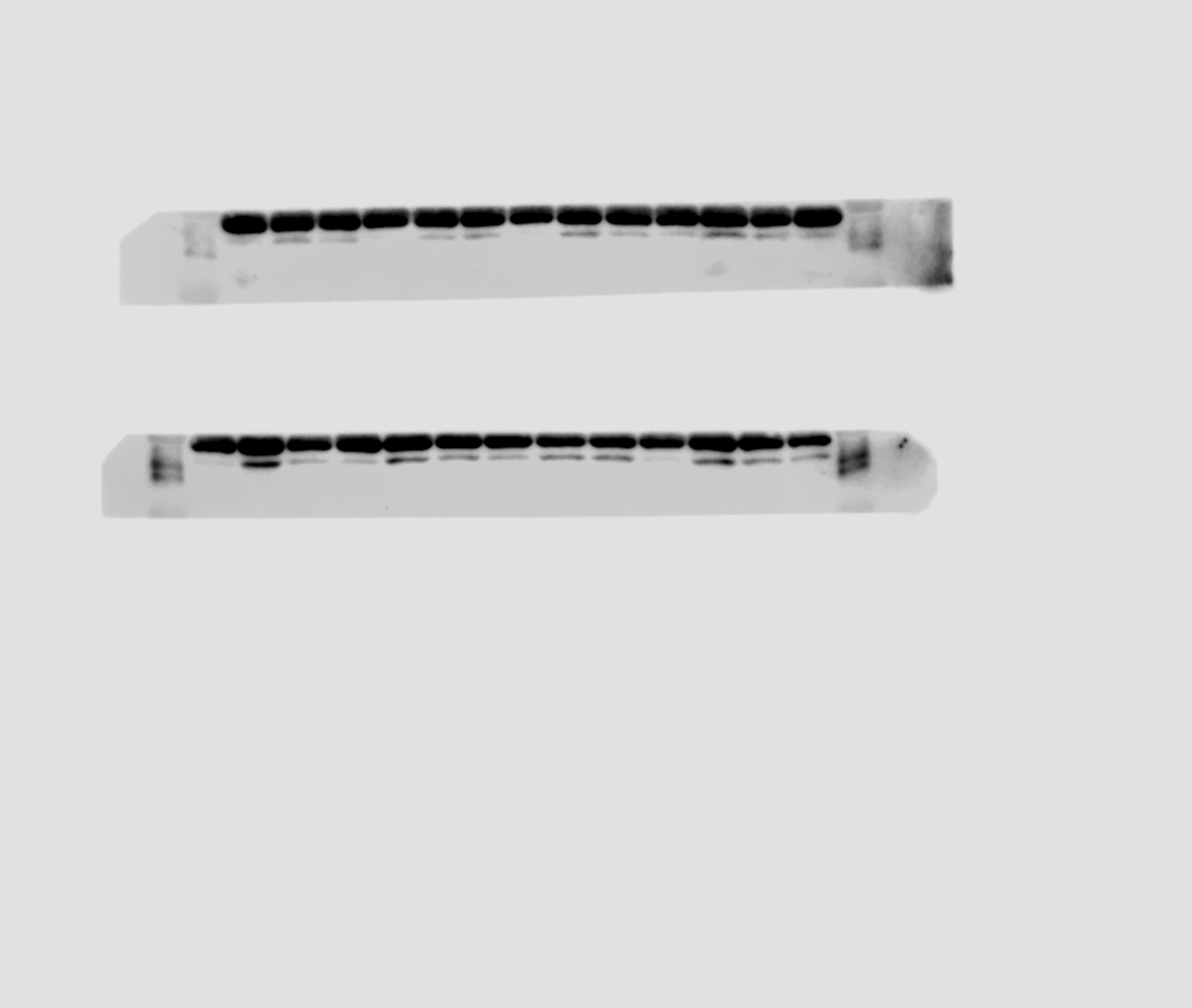

Supplement: Supplementary file 7 — Source data Fig. 6 [file 44321_2026_426_MOESM7_ESM.zip › Figure 6 updated/6B/F6B Females Brain MRPL37 c d.tif]

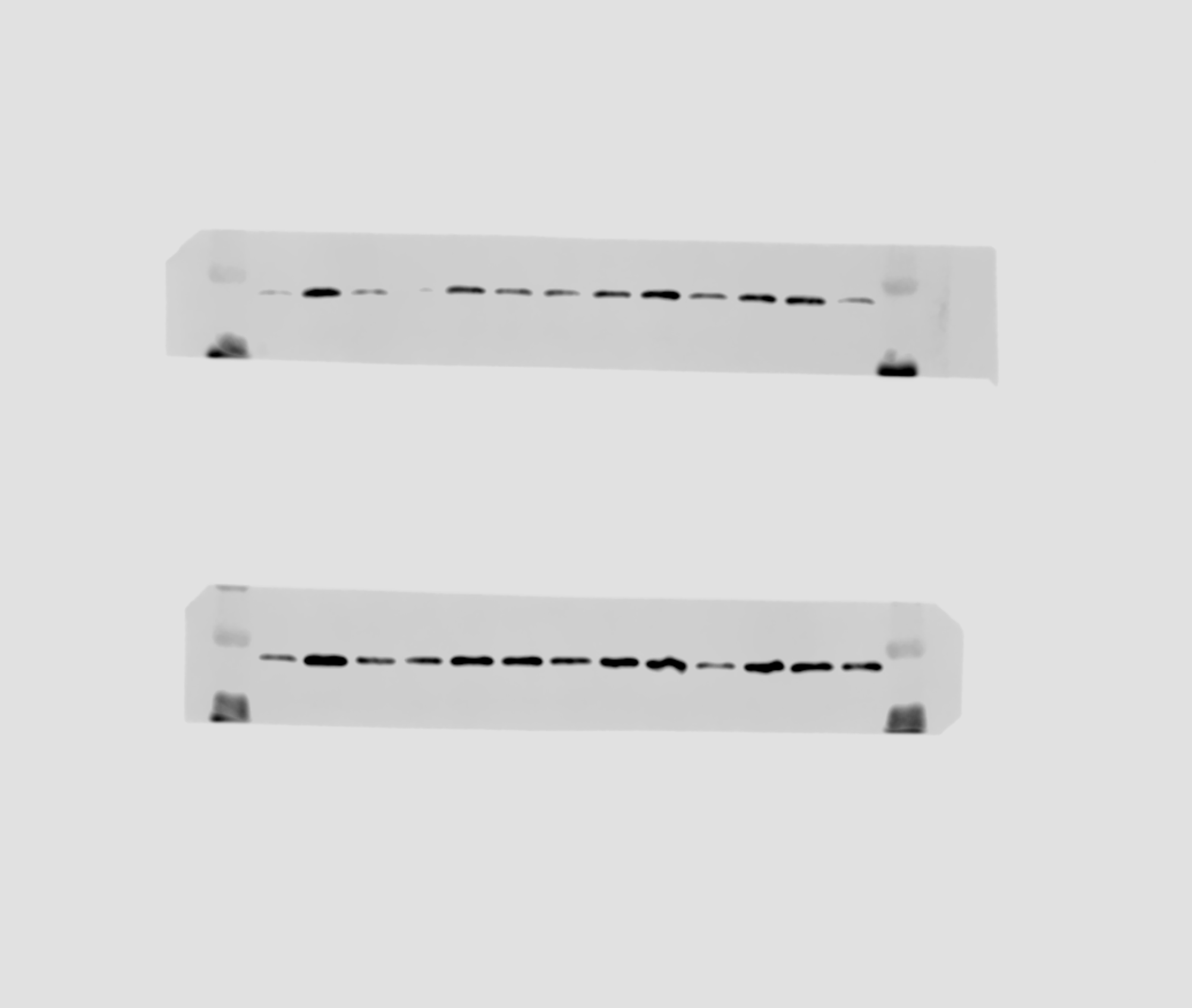

Supplement: Supplementary file 7 — Source data Fig. 6 [file 44321_2026_426_MOESM7_ESM.zip › Figure 6 updated/6B/F6B Females Brain MRPS35 e f.tif]

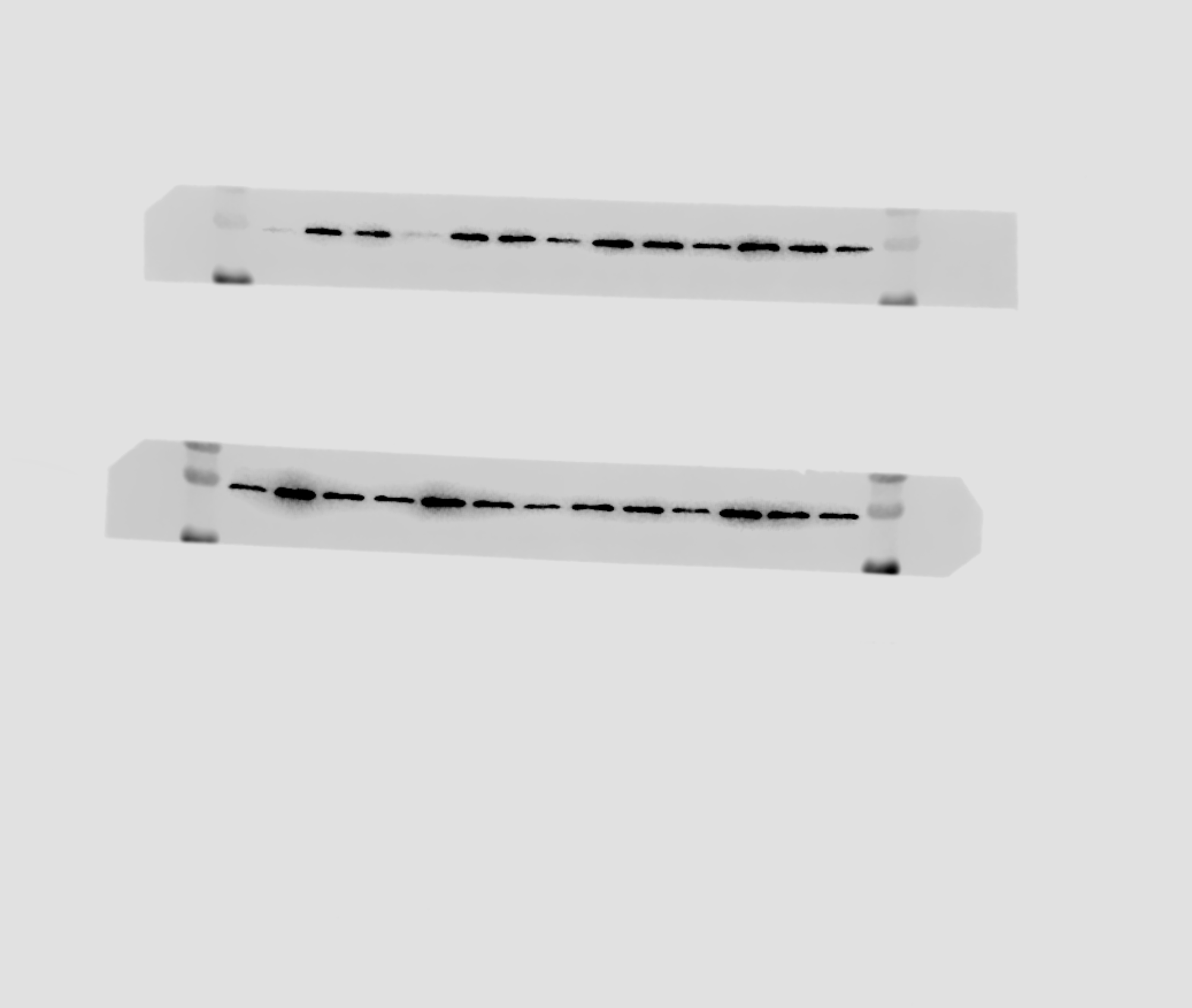

Supplement: Supplementary file 7 — Source data Fig. 6 [file 44321_2026_426_MOESM7_ESM.zip › Figure 6 updated/6B/F6B Females Brain MRPS9 a b.tif]

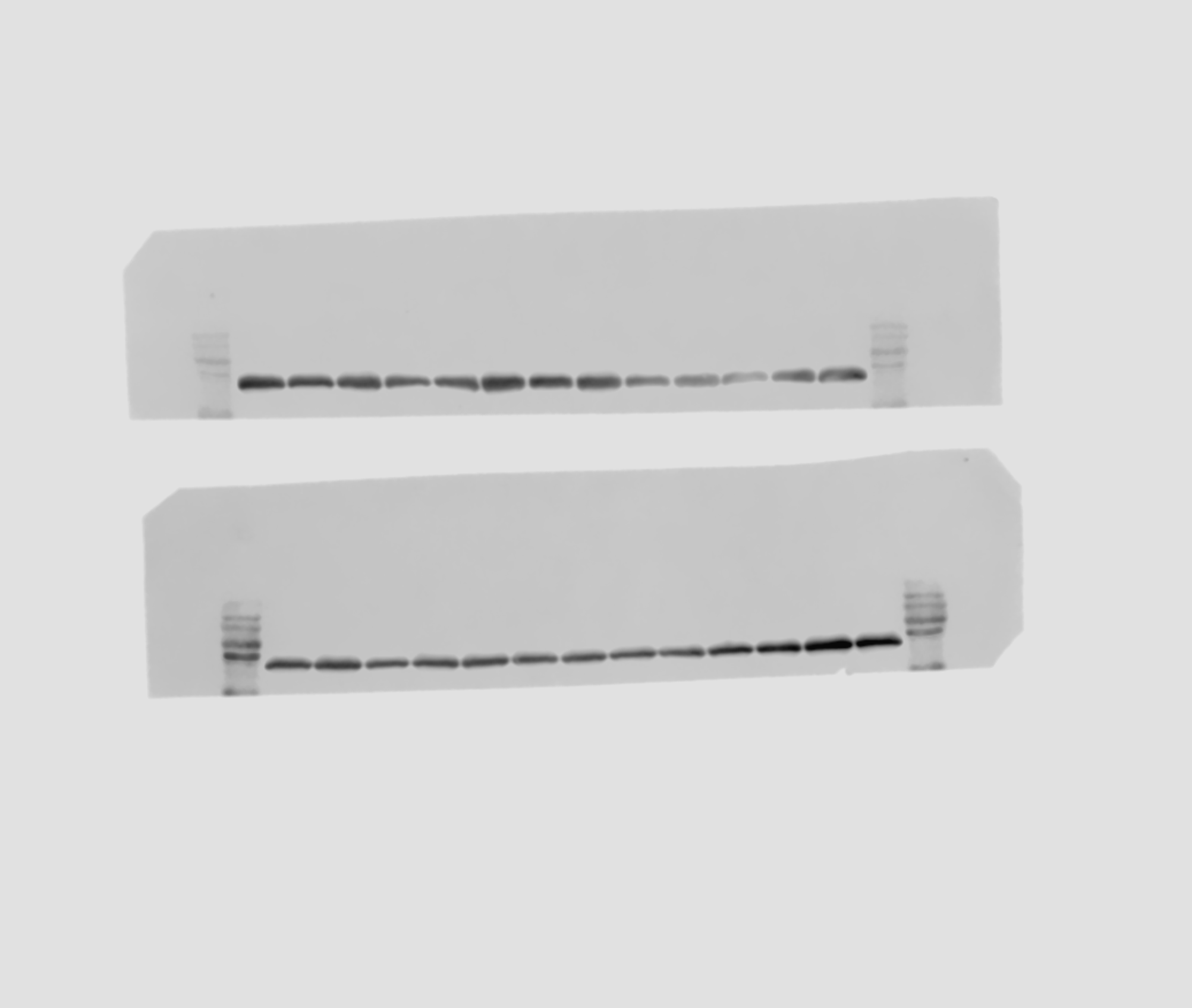

Supplement: Supplementary file 7 — Source data Fig. 6 [file 44321_2026_426_MOESM7_ESM.zip › Figure 6 updated/6B/F6B Females Brain SDHA a b.tif]

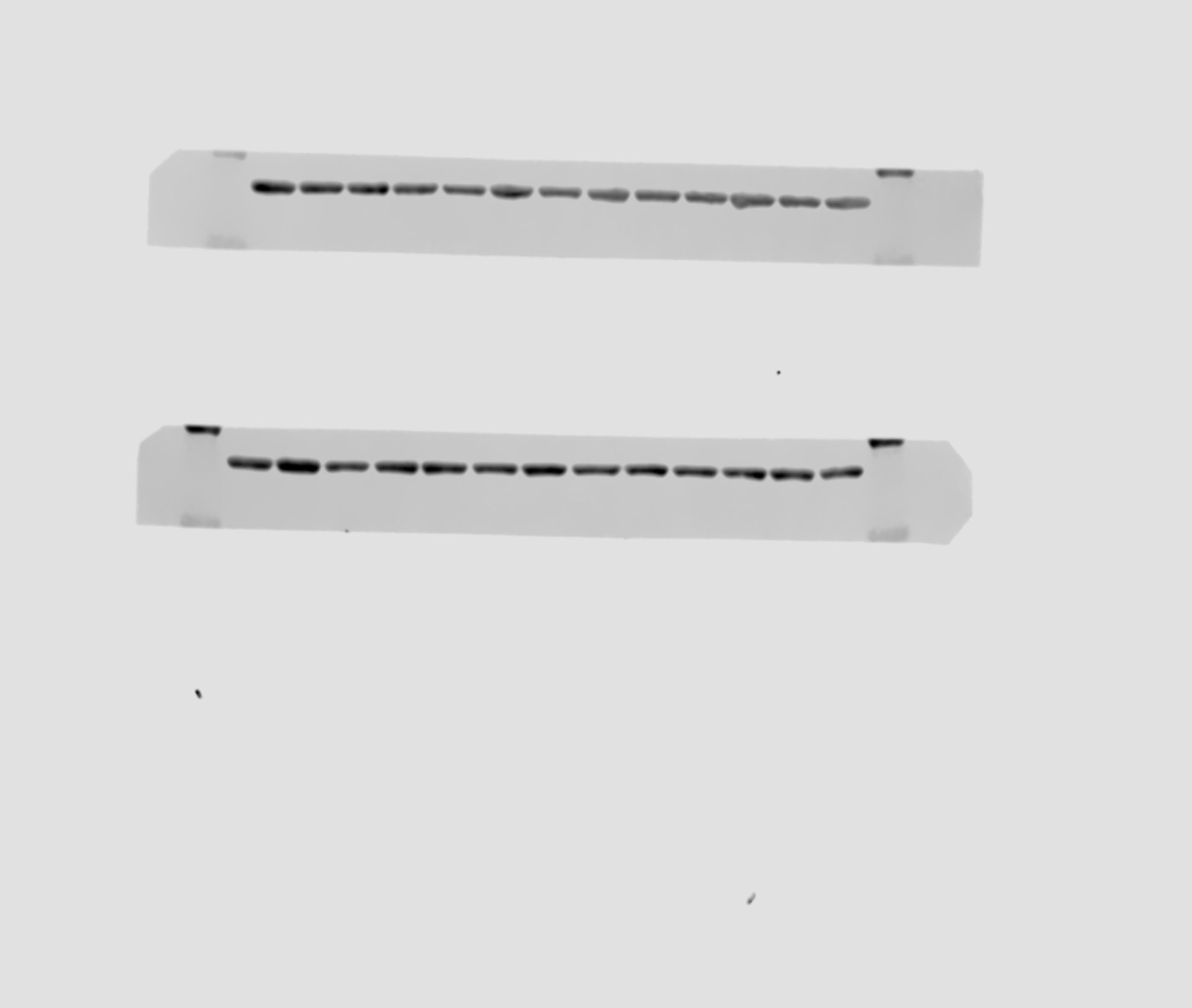

Supplement: Supplementary file 7 — Source data Fig. 6 [file 44321_2026_426_MOESM7_ESM.zip › Figure 6 updated/6B/F6B Females Brain SDHA c d.tif]

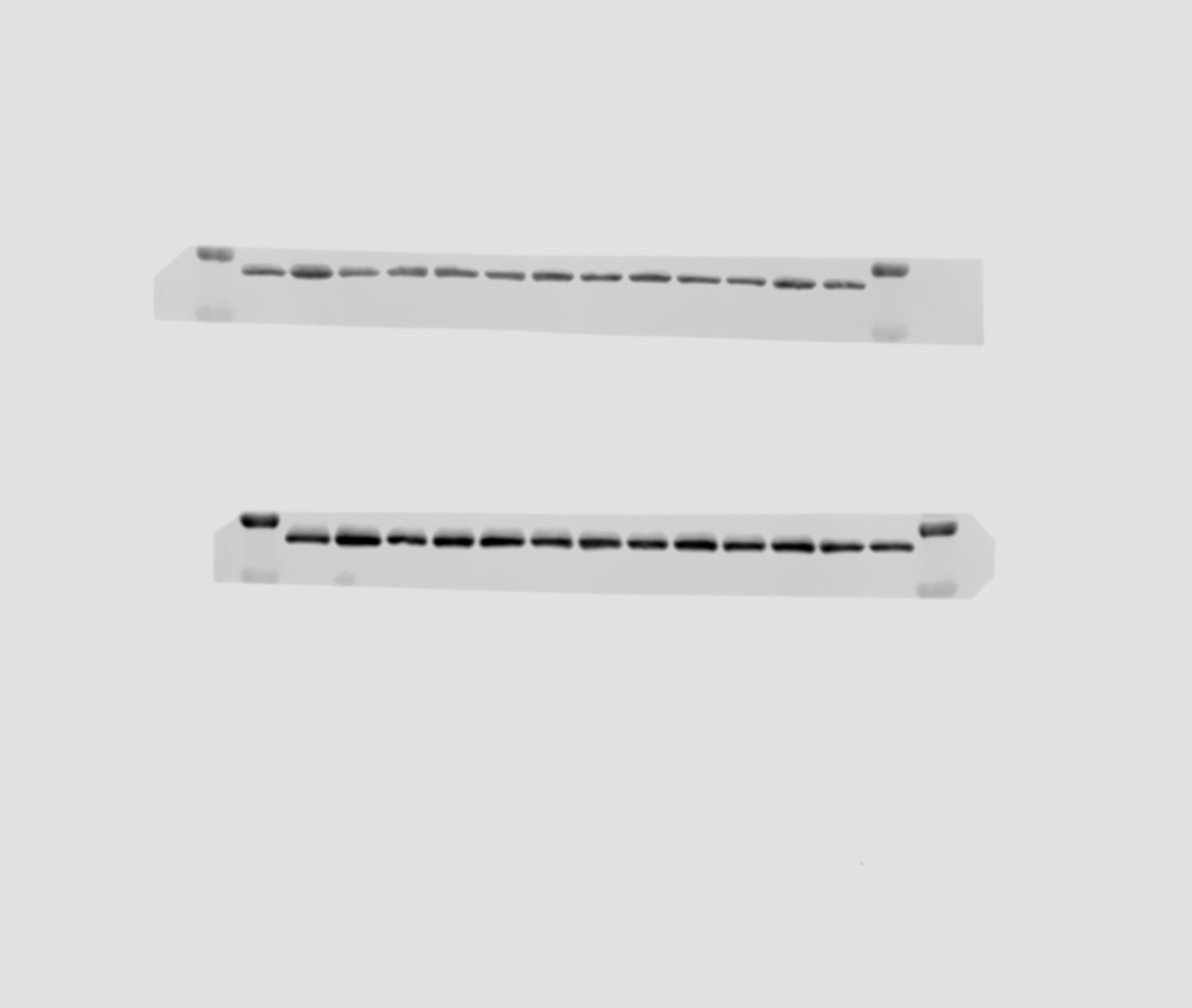

Supplement: Supplementary file 7 — Source data Fig. 6 [file 44321_2026_426_MOESM7_ESM.zip › Figure 6 updated/6B/F6B Females Brain SDHA e f.tif]

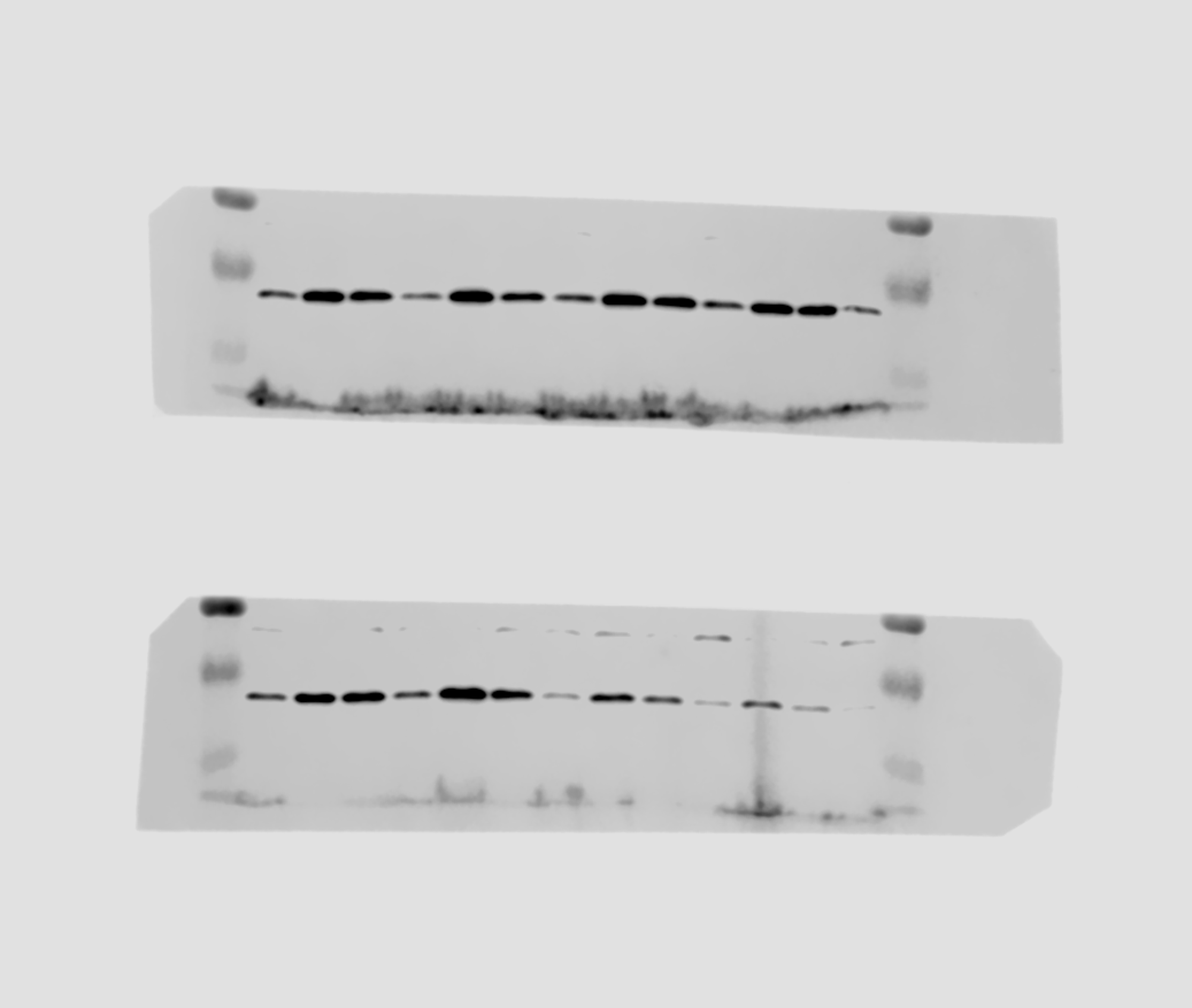

Supplement: Supplementary file 7 — Source data Fig. 6 [file 44321_2026_426_MOESM7_ESM.zip › Figure 6 updated/6B/F6B Males Brain MRPL13 g h.tif]

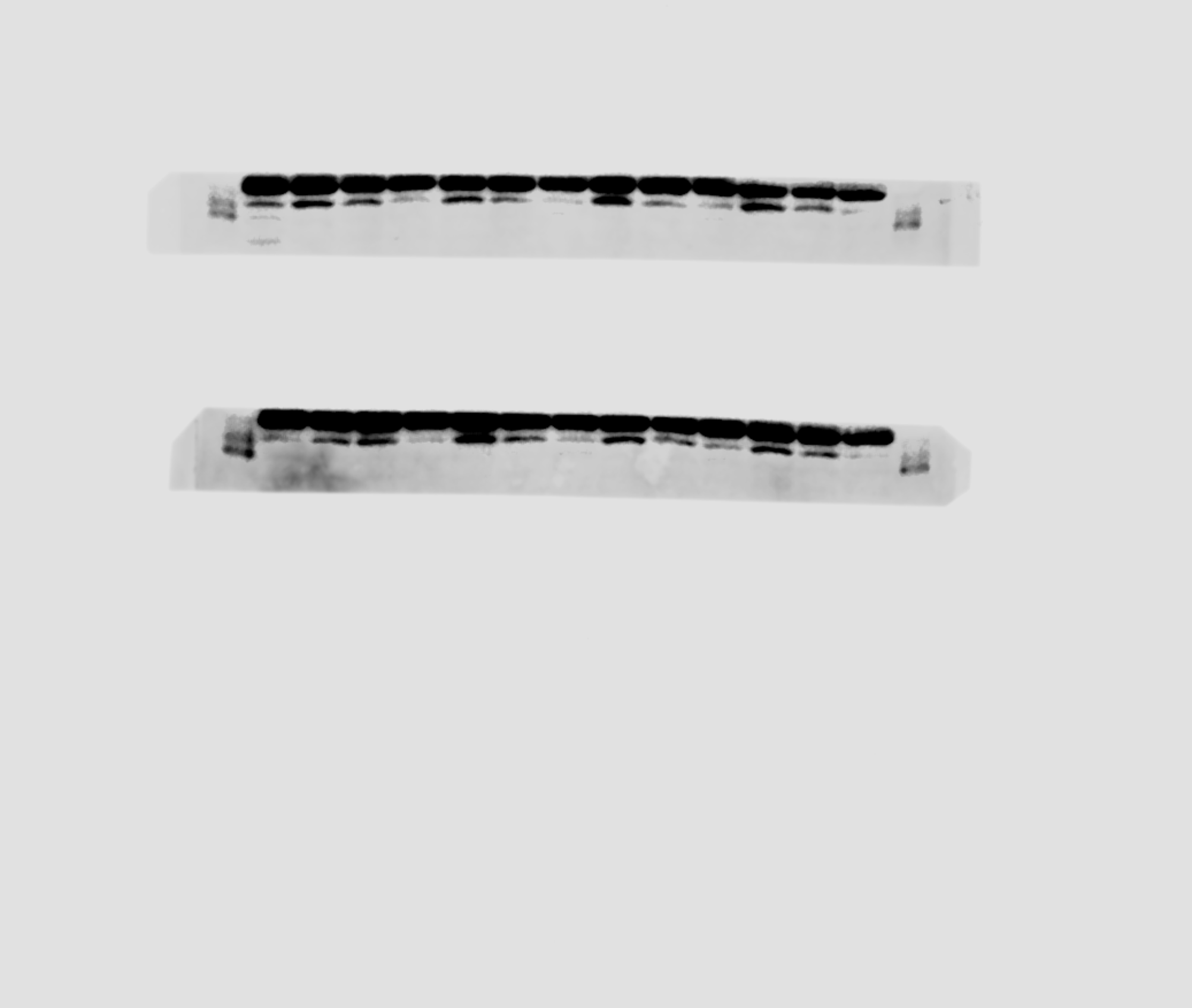

Supplement: Supplementary file 7 — Source data Fig. 6 [file 44321_2026_426_MOESM7_ESM.zip › Figure 6 updated/6B/F6B Males Brain MRPL37 e f.tif]

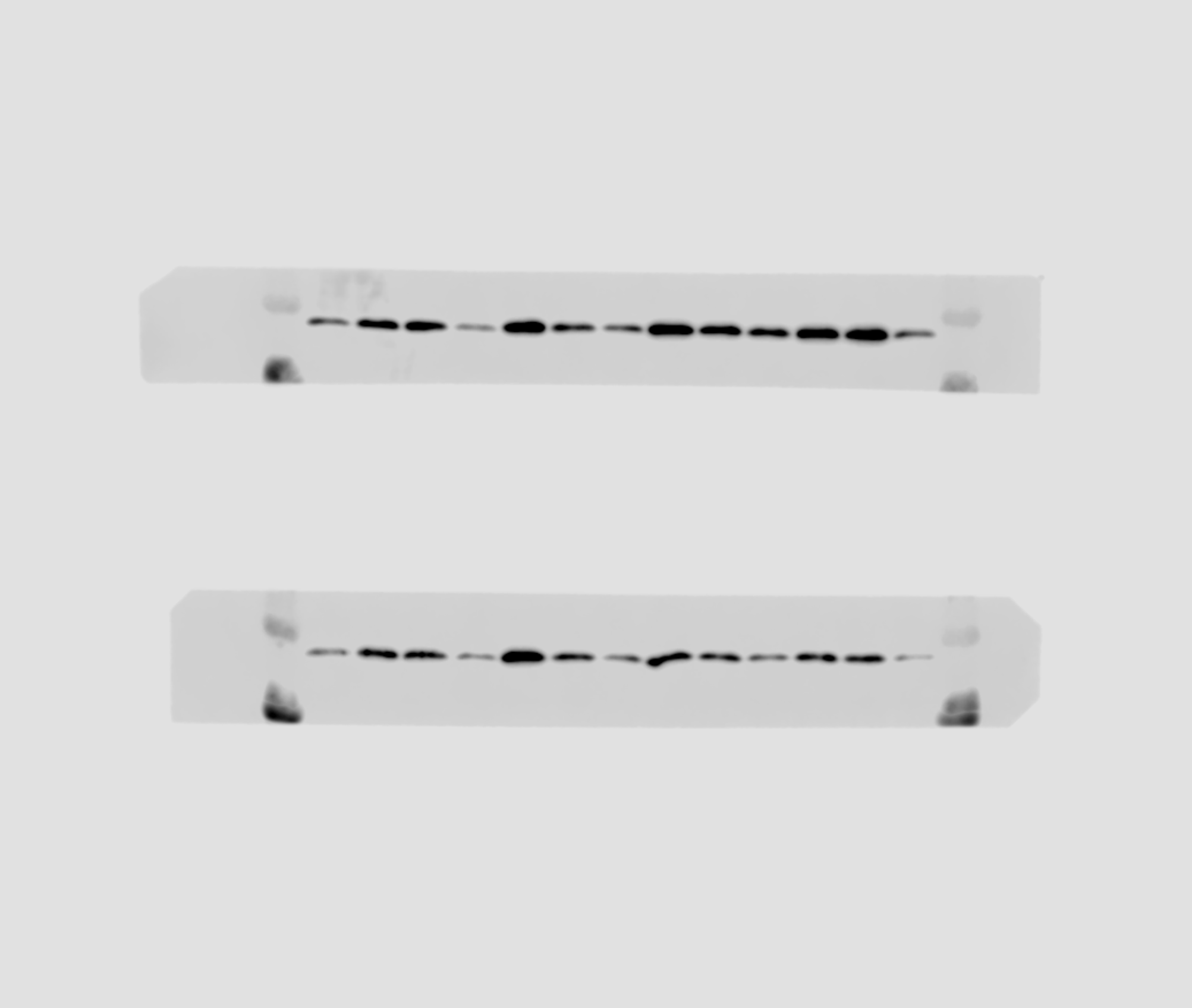

Supplement: Supplementary file 7 — Source data Fig. 6 [file 44321_2026_426_MOESM7_ESM.zip › Figure 6 updated/6B/F6B Males Brain MRPS35 c d.tif]

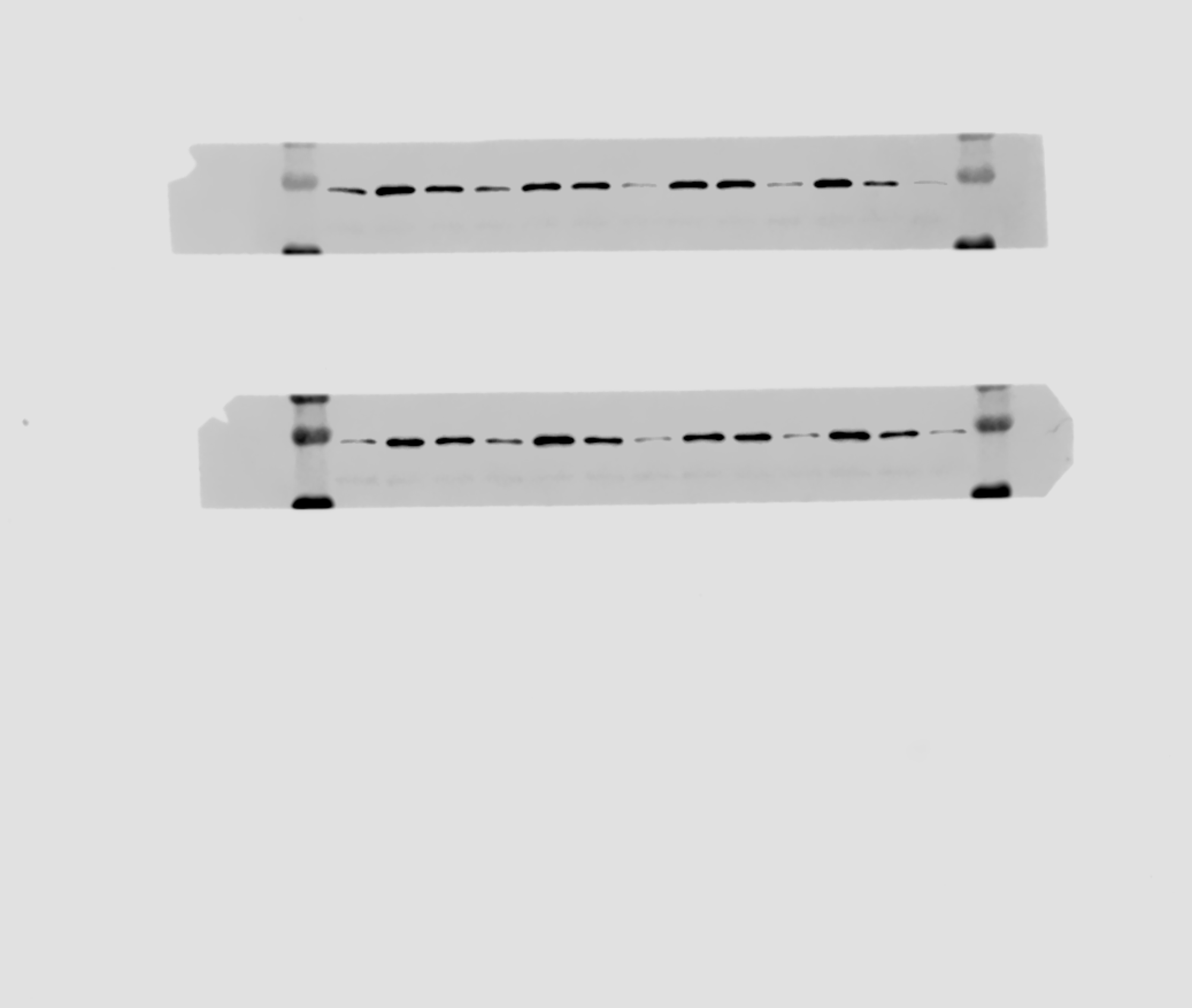

Supplement: Supplementary file 7 — Source data Fig. 6 [file 44321_2026_426_MOESM7_ESM.zip › Figure 6 updated/6B/F6B Males Brain MRPS9 a b.tif]

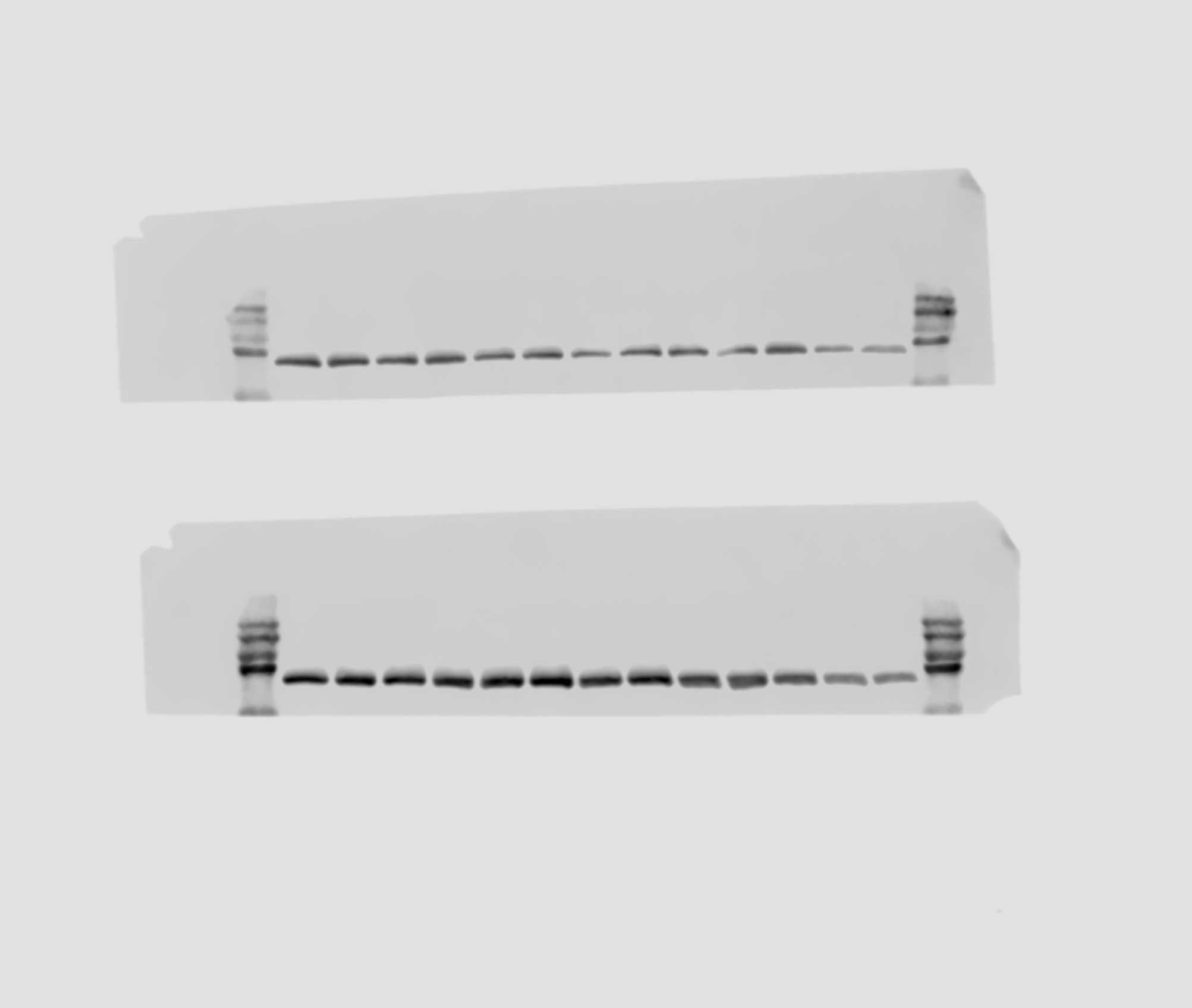

Supplement: Supplementary file 7 — Source data Fig. 6 [file 44321_2026_426_MOESM7_ESM.zip › Figure 6 updated/6B/F6B Males Brain SDHA a b.tif]

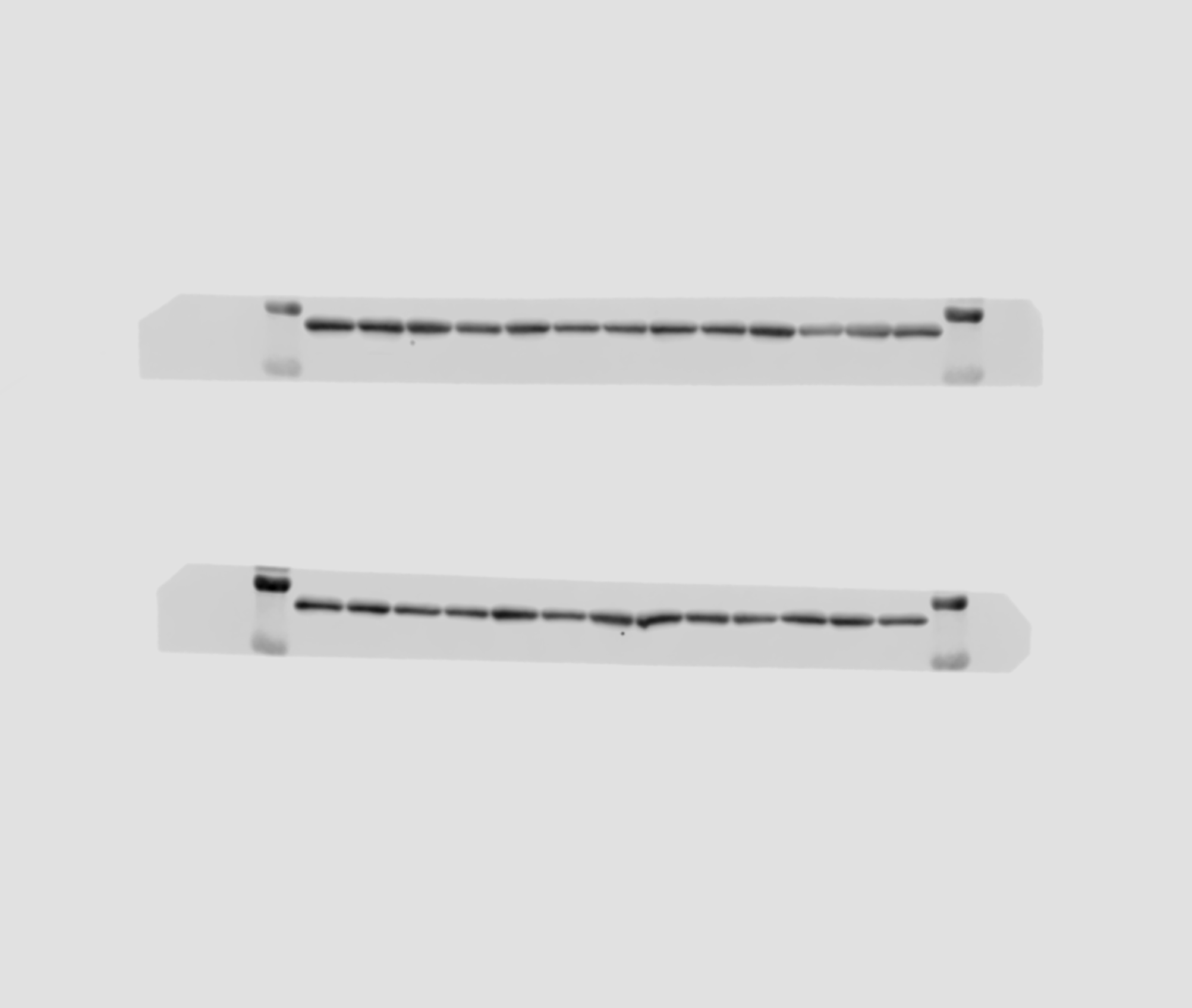

Supplement: Supplementary file 7 — Source data Fig. 6 [file 44321_2026_426_MOESM7_ESM.zip › Figure 6 updated/6B/F6B Males Brain SDHA c d.tif]

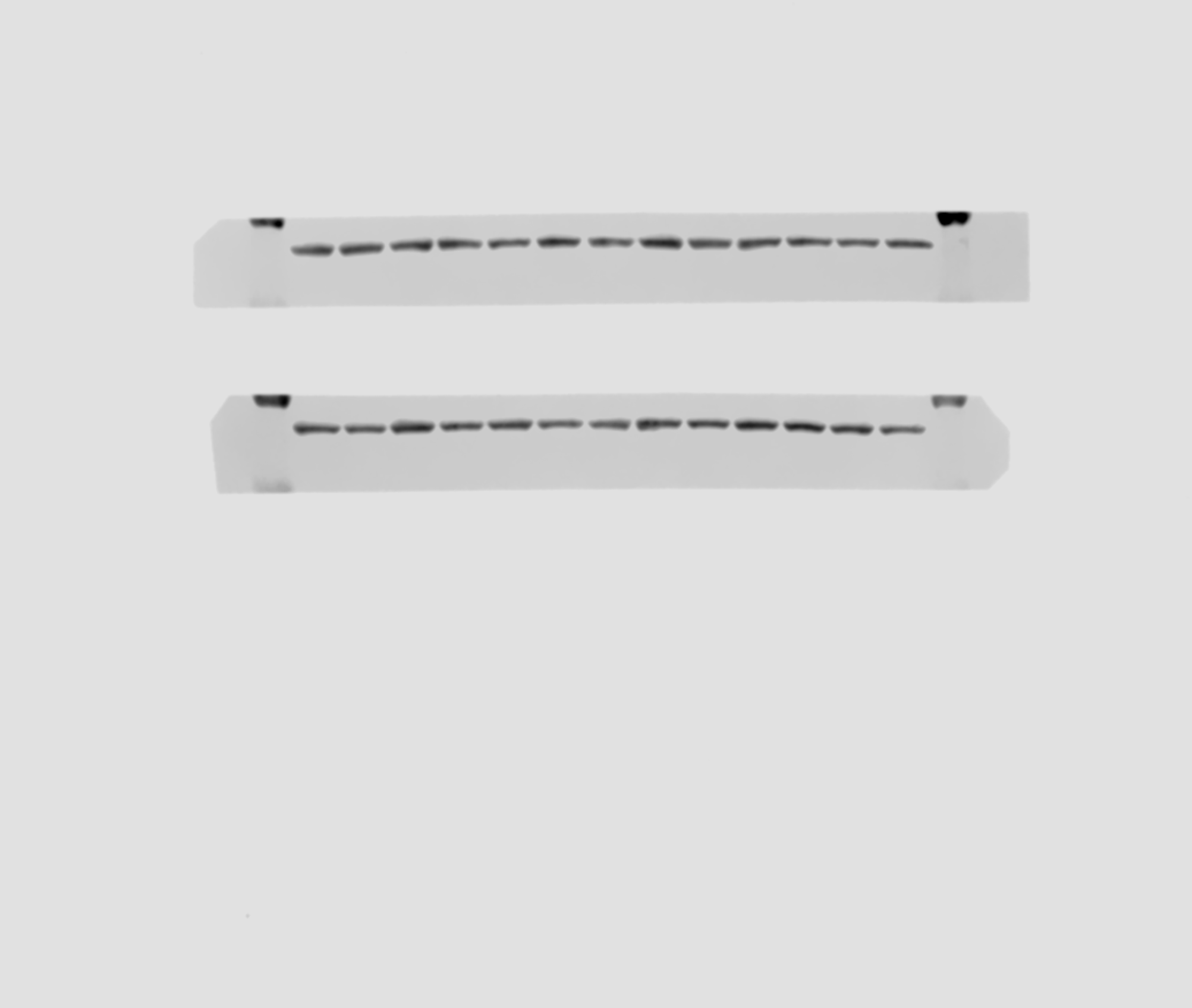

Supplement: Supplementary file 7 — Source data Fig. 6 [file 44321_2026_426_MOESM7_ESM.zip › Figure 6 updated/6B/F6B Males Brain SDHA e f.tif]

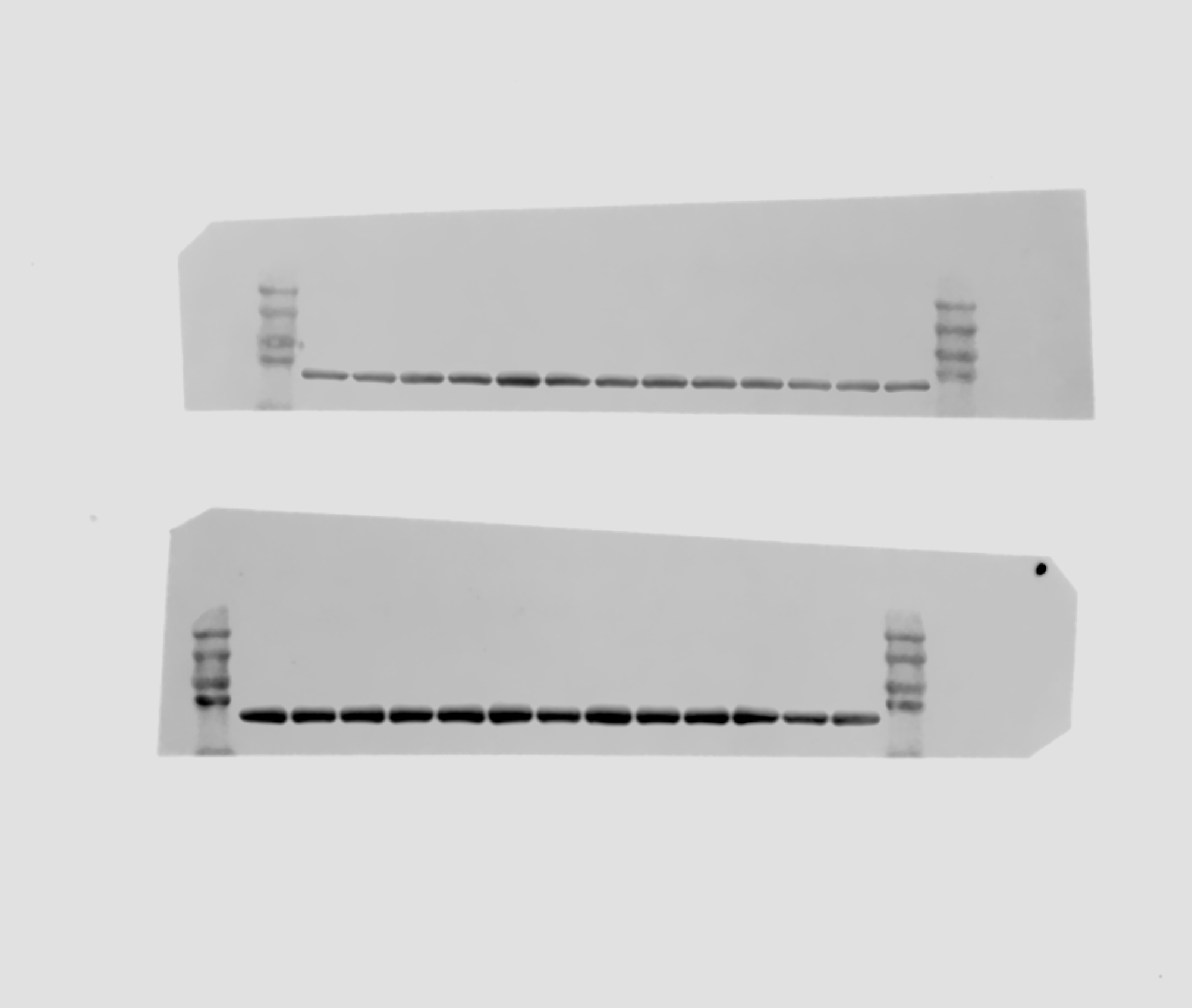

Supplement: Supplementary file 7 — Source data Fig. 6 [file 44321_2026_426_MOESM7_ESM.zip › Figure 6 updated/6B/F6B Males Brain SDHA g h.tif]

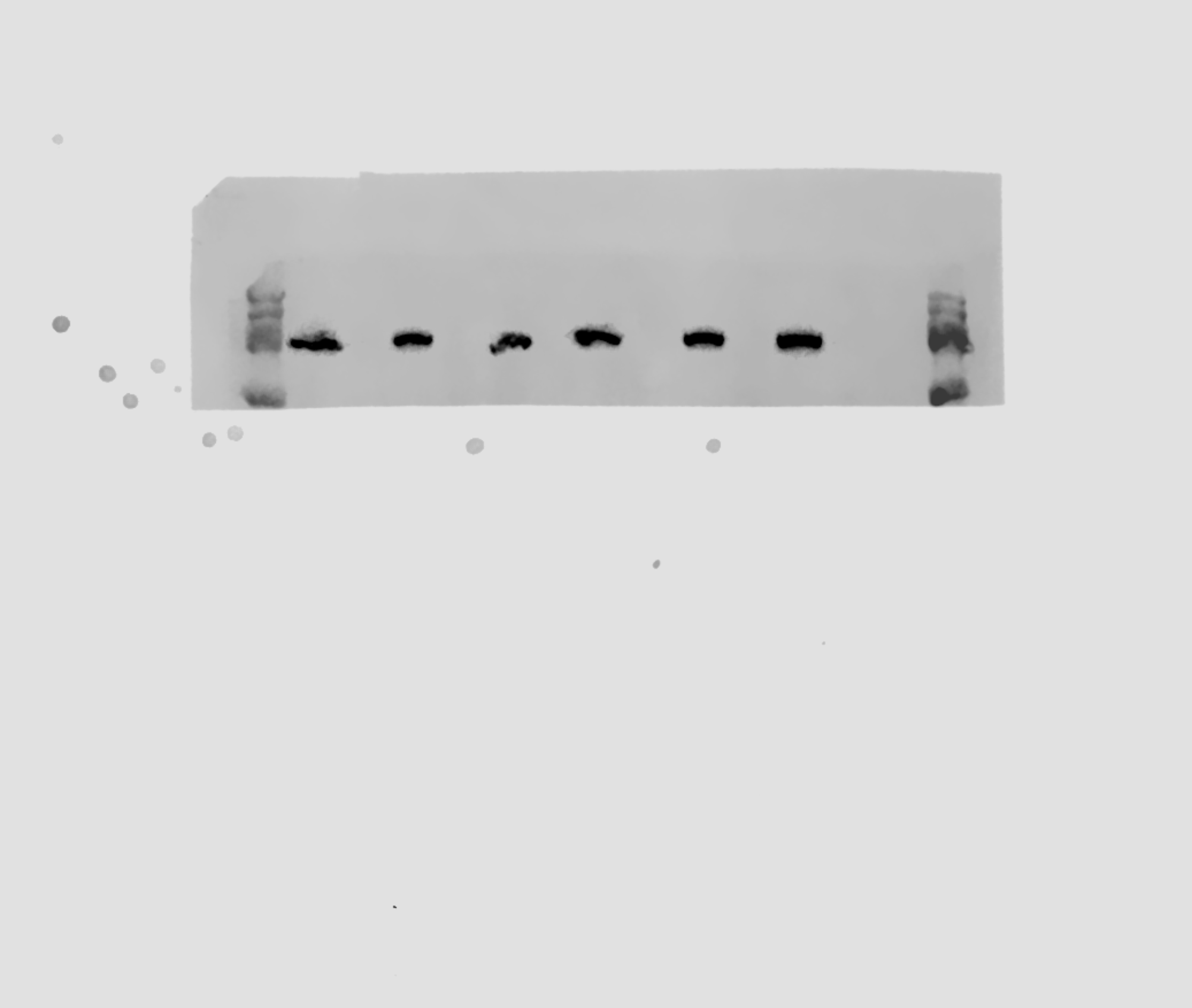

Supplement: Supplementary file 9 — Figure EV1 Source Data [file 44321_2026_426_MOESM9_ESM.zip › EV1 updated/EV1A/EV1A Brain EFG1.tif]

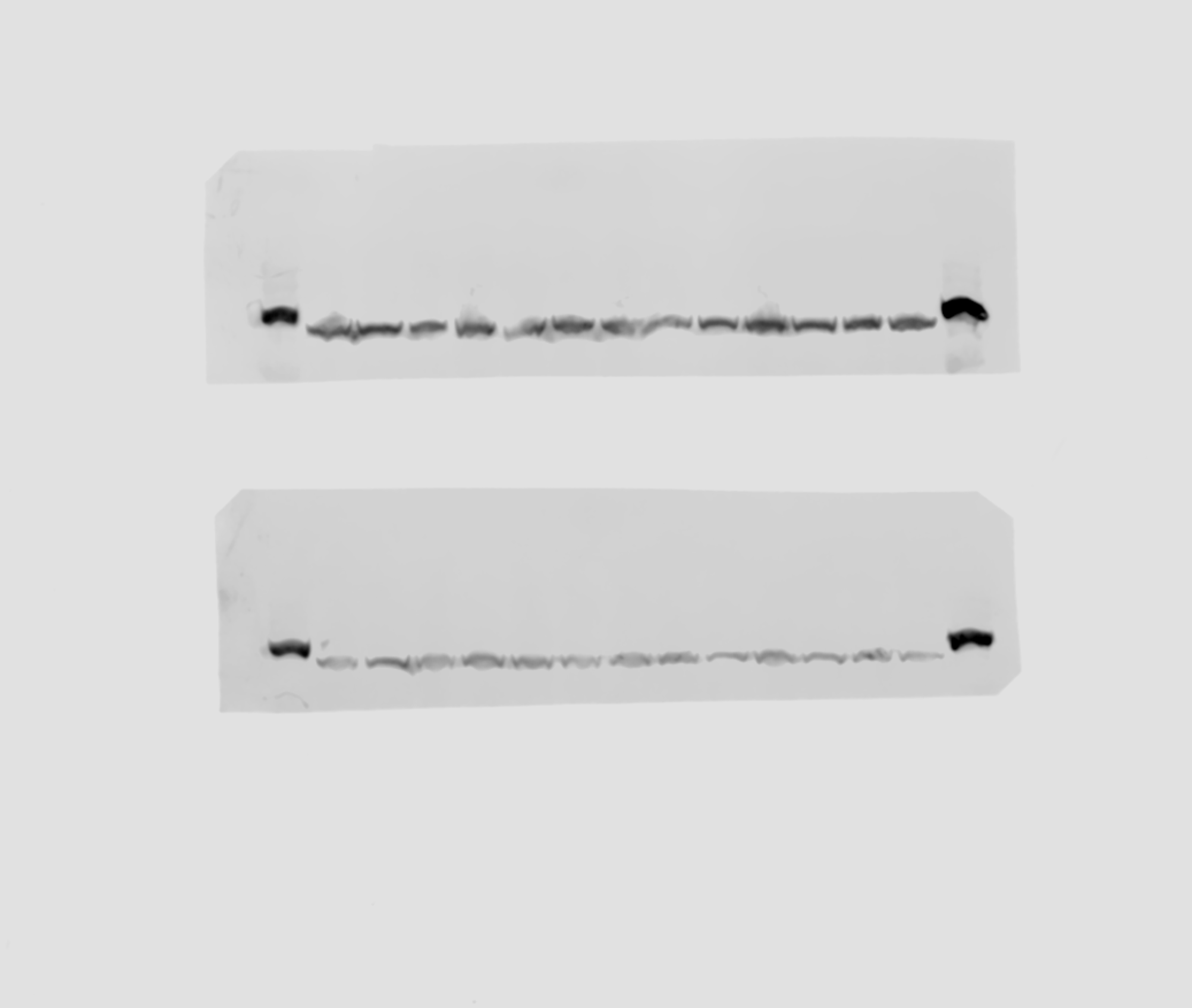

Supplement: Supplementary file 9 — Figure EV1 Source Data [file 44321_2026_426_MOESM9_ESM.zip › EV1 updated/EV1A/EV1A Brain SDHA a b.tif]

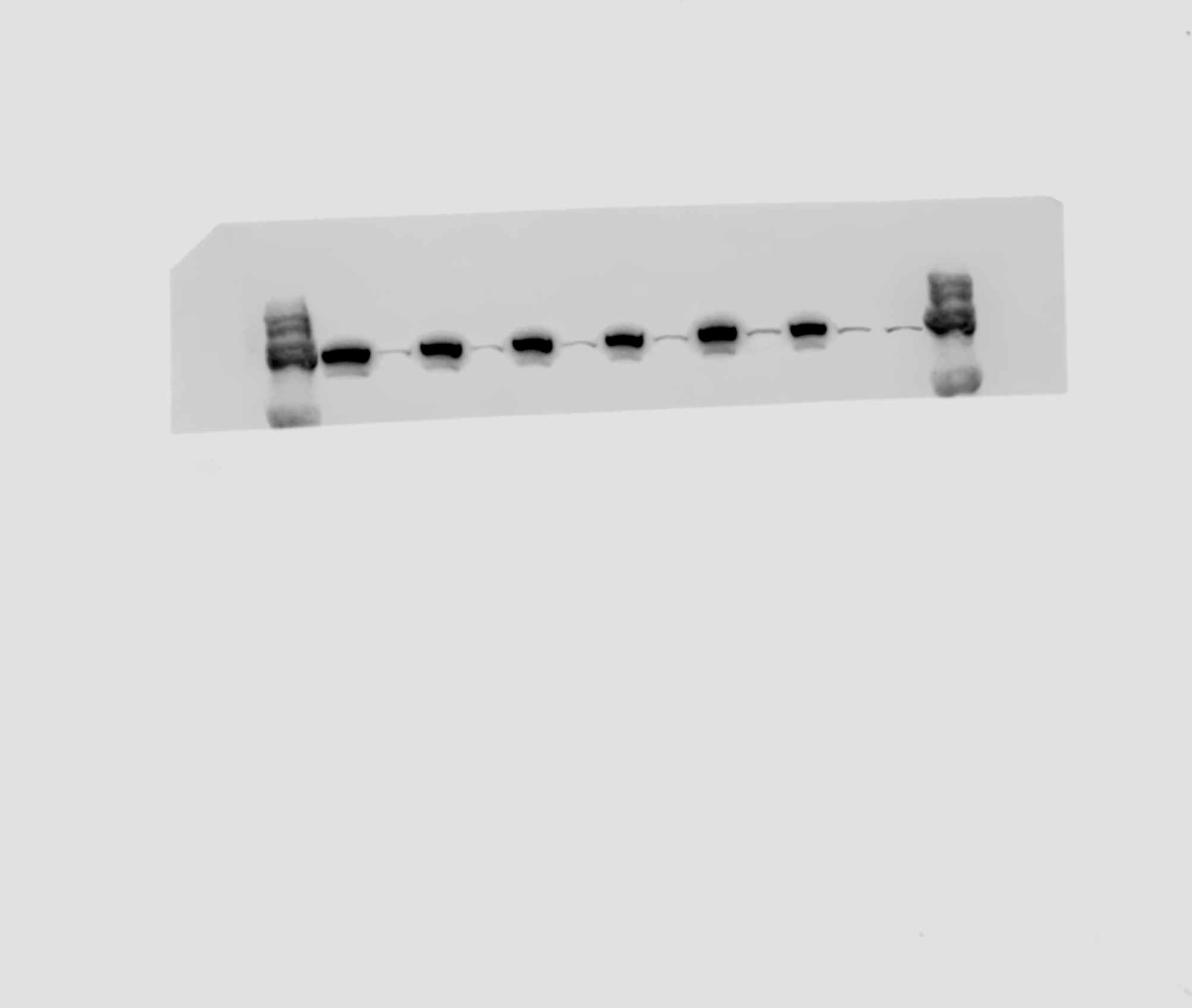

Supplement: Supplementary file 9 — Figure EV1 Source Data [file 44321_2026_426_MOESM9_ESM.zip › EV1 updated/EV1A/EV1A Liver EFG1.tif]

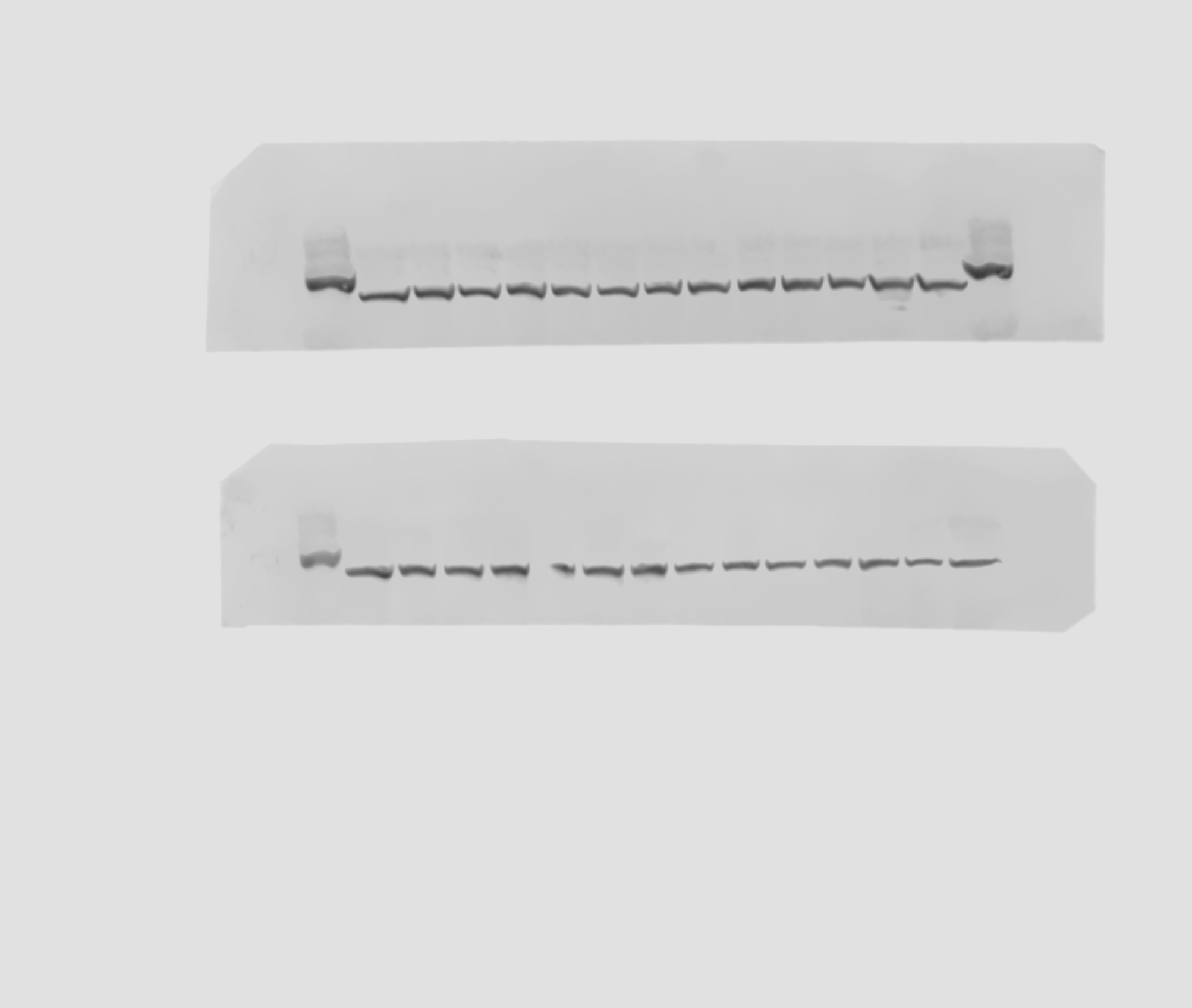

Supplement: Supplementary file 9 — Figure EV1 Source Data [file 44321_2026_426_MOESM9_ESM.zip › EV1 updated/EV1A/EV1A Liver SDHA a b.tif]

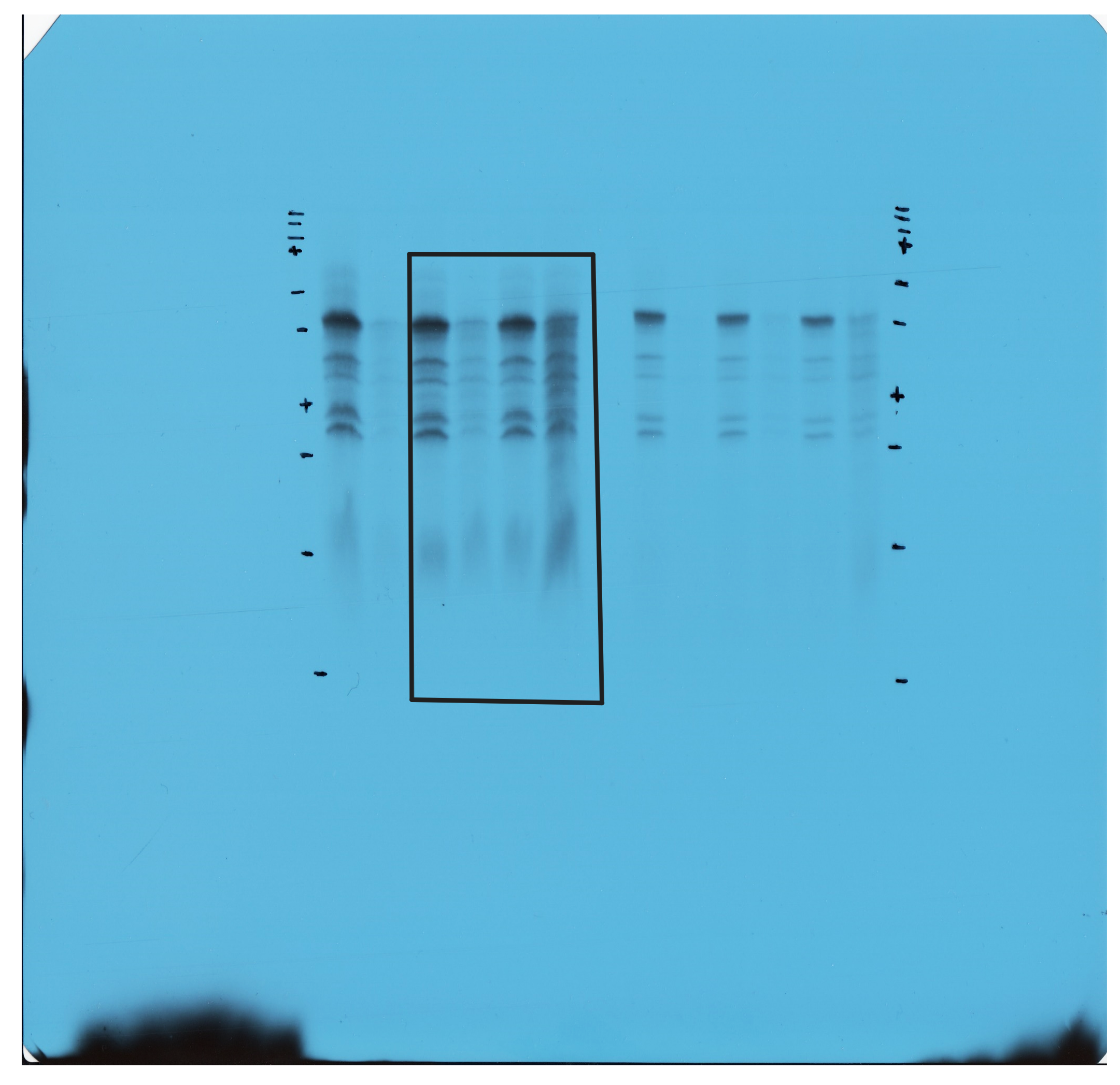

Supplement: Supplementary file 9 — Figure EV1 Source Data [file 44321_2026_426_MOESM9_ESM.zip › EV1 updated/EV1B/Autoradiography brain.png]

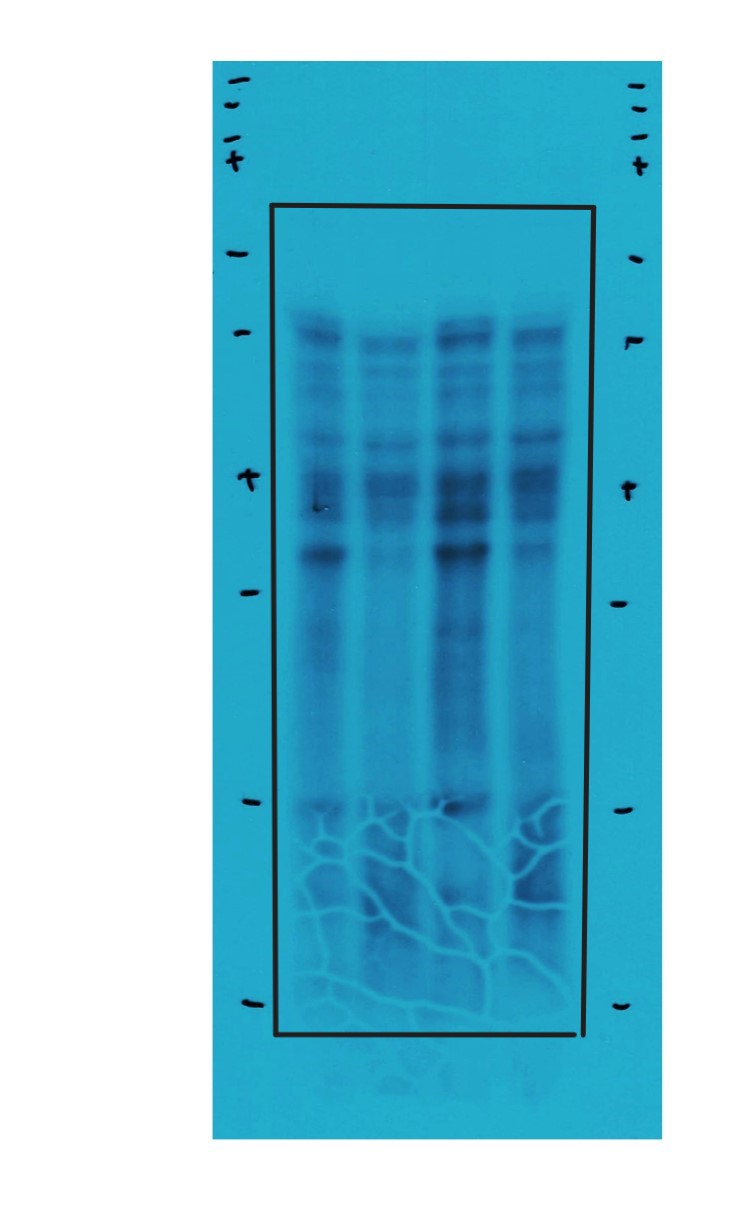

Supplement: Supplementary file 9 — Figure EV1 Source Data [file 44321_2026_426_MOESM9_ESM.zip › EV1 updated/EV1B/Autoradiography liver.jpg]

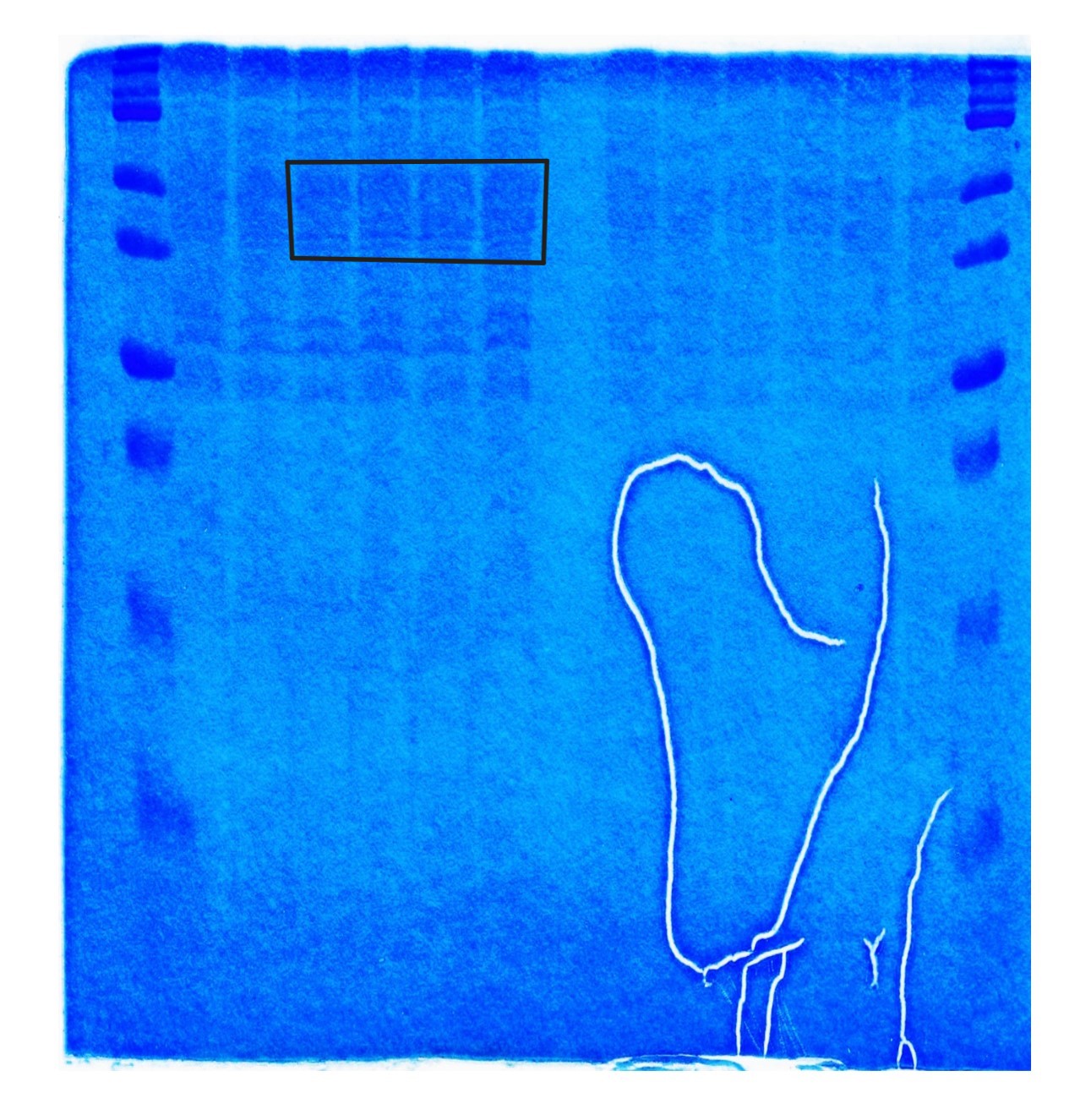

Supplement: Supplementary file 9 — Figure EV1 Source Data [file 44321_2026_426_MOESM9_ESM.zip › EV1 updated/EV1B/Coomassie brain.jpg]

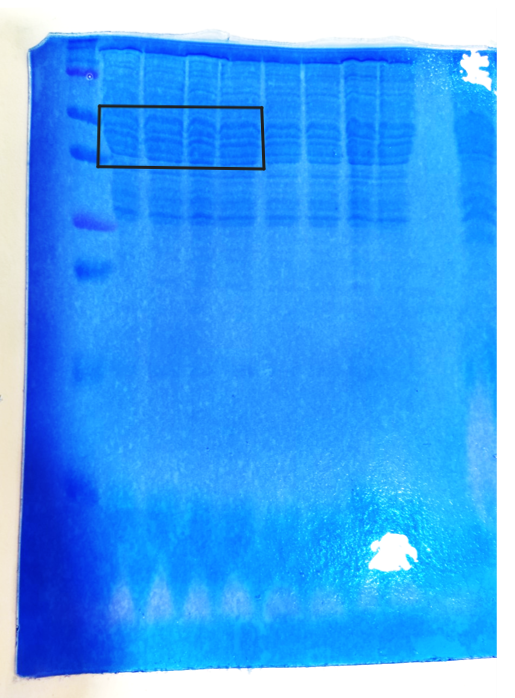

Supplement: Supplementary file 9 — Figure EV1 Source Data [file 44321_2026_426_MOESM9_ESM.zip › EV1 updated/EV1B/Coomassie liver.png]

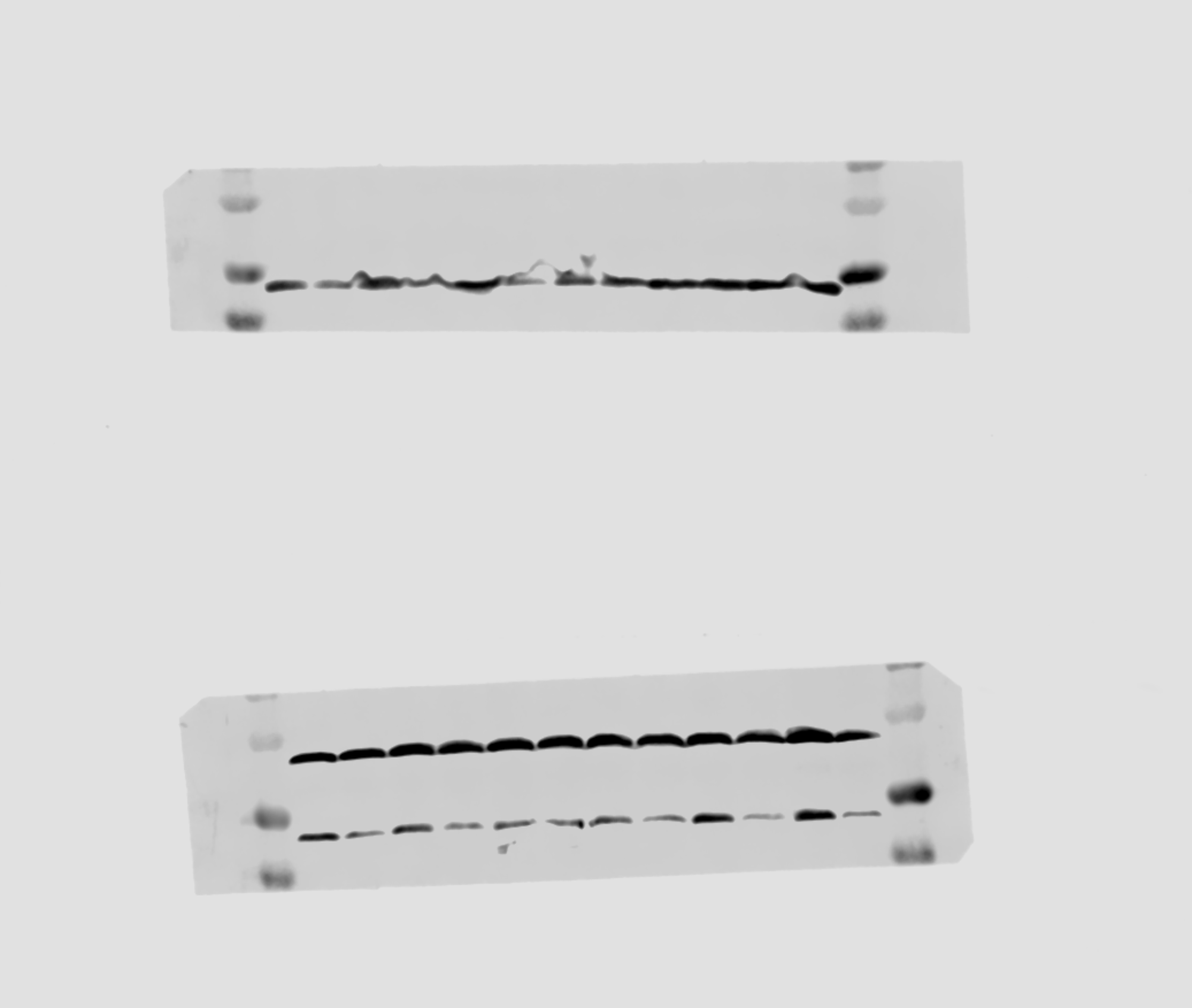

Supplement: Supplementary file 9 — Figure EV1 Source Data [file 44321_2026_426_MOESM9_ESM.zip › EV1 updated/EV1C/EV1C Brain COX2 a i b.tif]

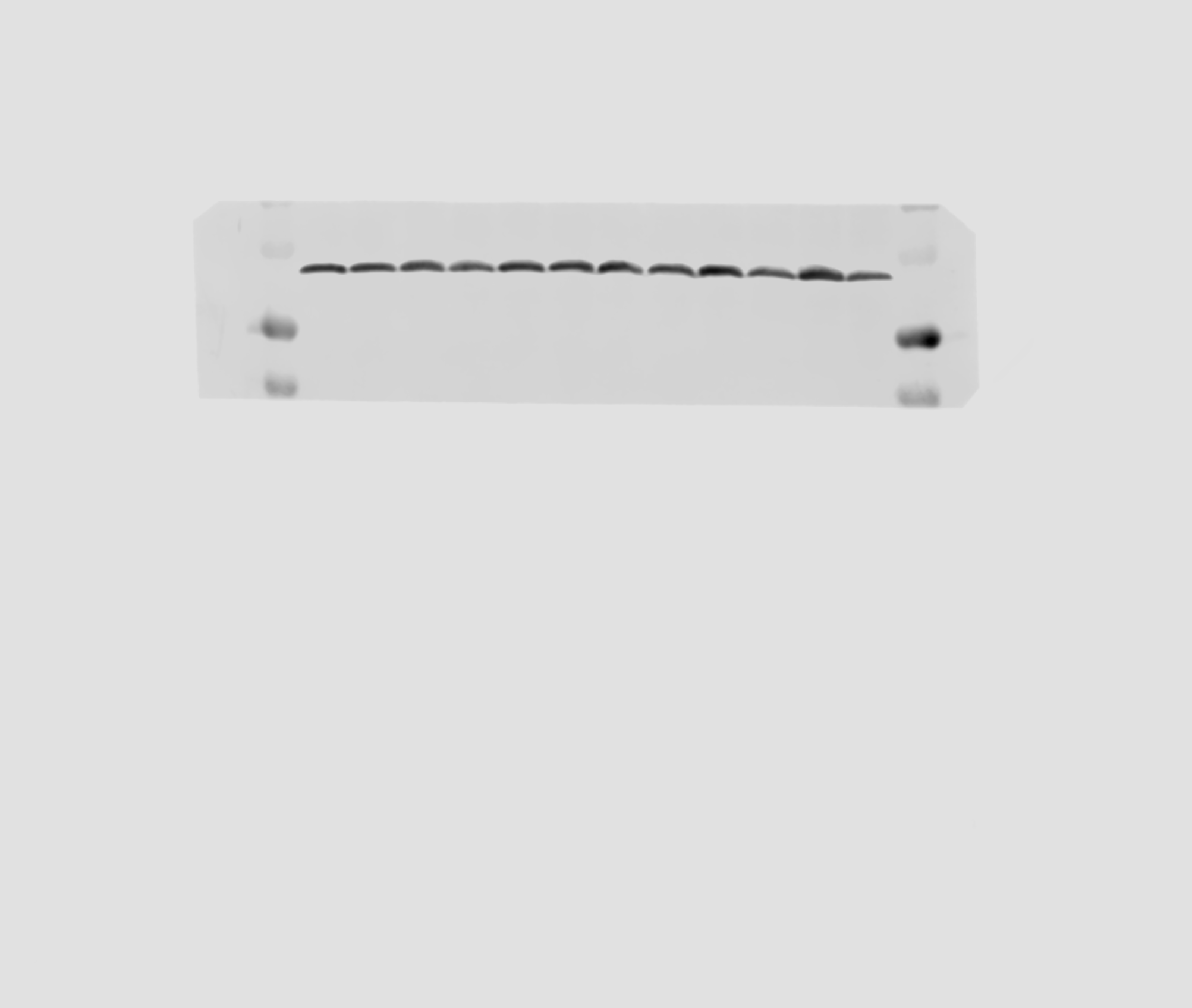

Supplement: Supplementary file 9 — Figure EV1 Source Data [file 44321_2026_426_MOESM9_ESM.zip › EV1 updated/EV1C/EV1C Brain NDUFA9.tif]
