# Supplementary material for: Systemic delivery of AAV-GFM1 corrects COXPD1 molecular alterations in Gfm1R671C/− mice
Source: EMBO Mol Med. 2026 Apr 17;18(6):2152–79. doi: 10.1038/s44321-026-00426-4 (PMC13269562; doi:10.1038/s44321-026-00426-4)
Supplement: Supplementary file 3 — Source data Fig. 2 [file 44321_2026_426_MOESM3_ESM.zip › Figure 2 updated/2C/Fig2C - WB EFG1 - Females and Males V2.pdf]

Brain mitochondria

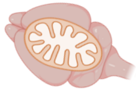

Females ♀

Western blot – SDS-PAGE

10 weeks old mice

ssAAV9P31-hSyn-GFM1

29/01/24

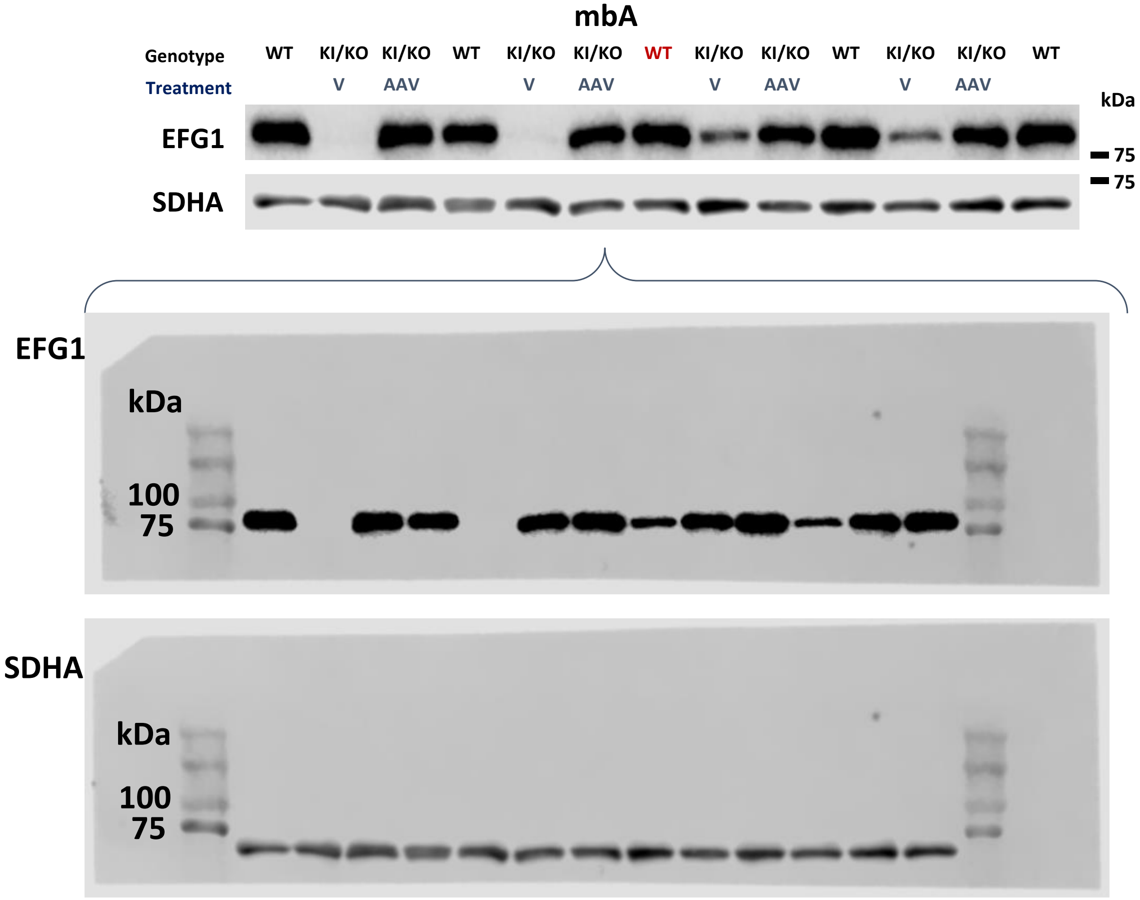

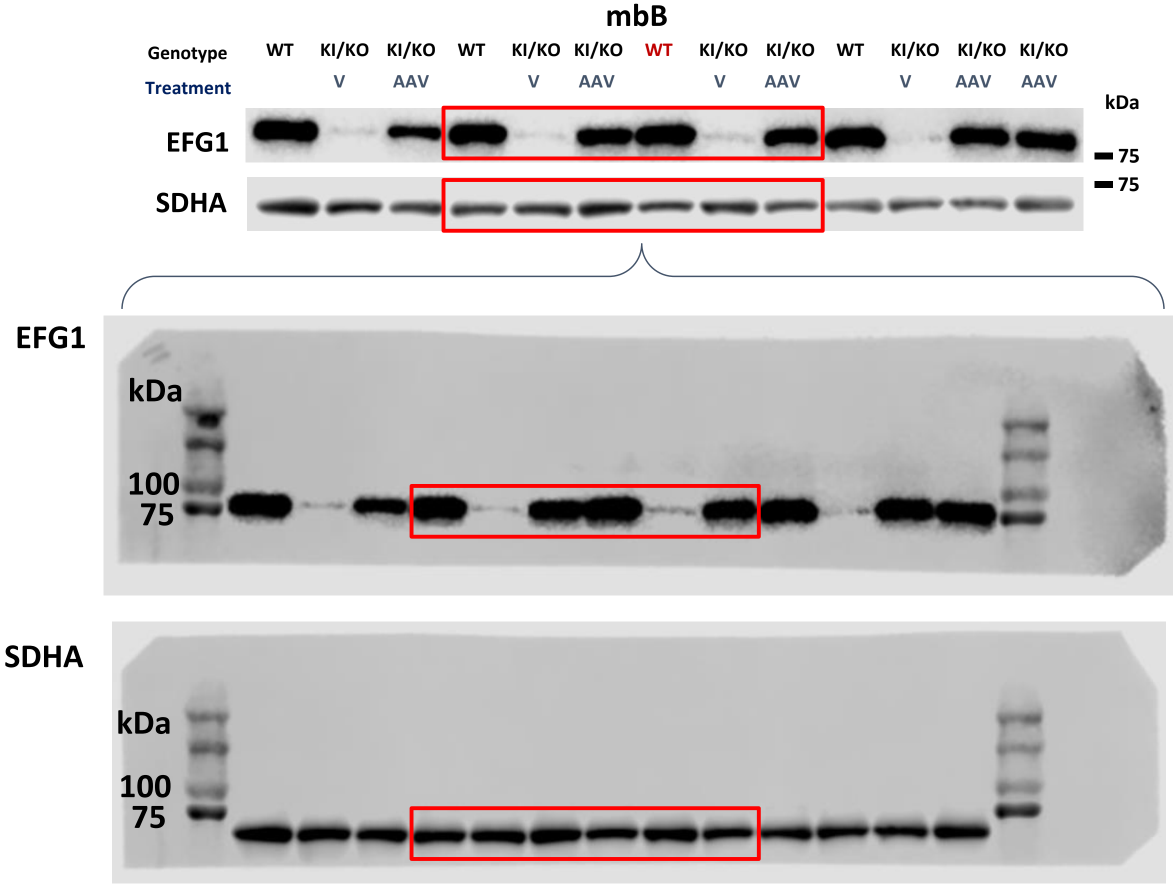

Selected area for publication

Brain mitochondria 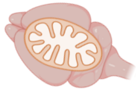 Males ♂

Western blot – SDS-PAGE

10 weeks old mice
ssAAV9P31-hSyn-GFM1

22/02/24

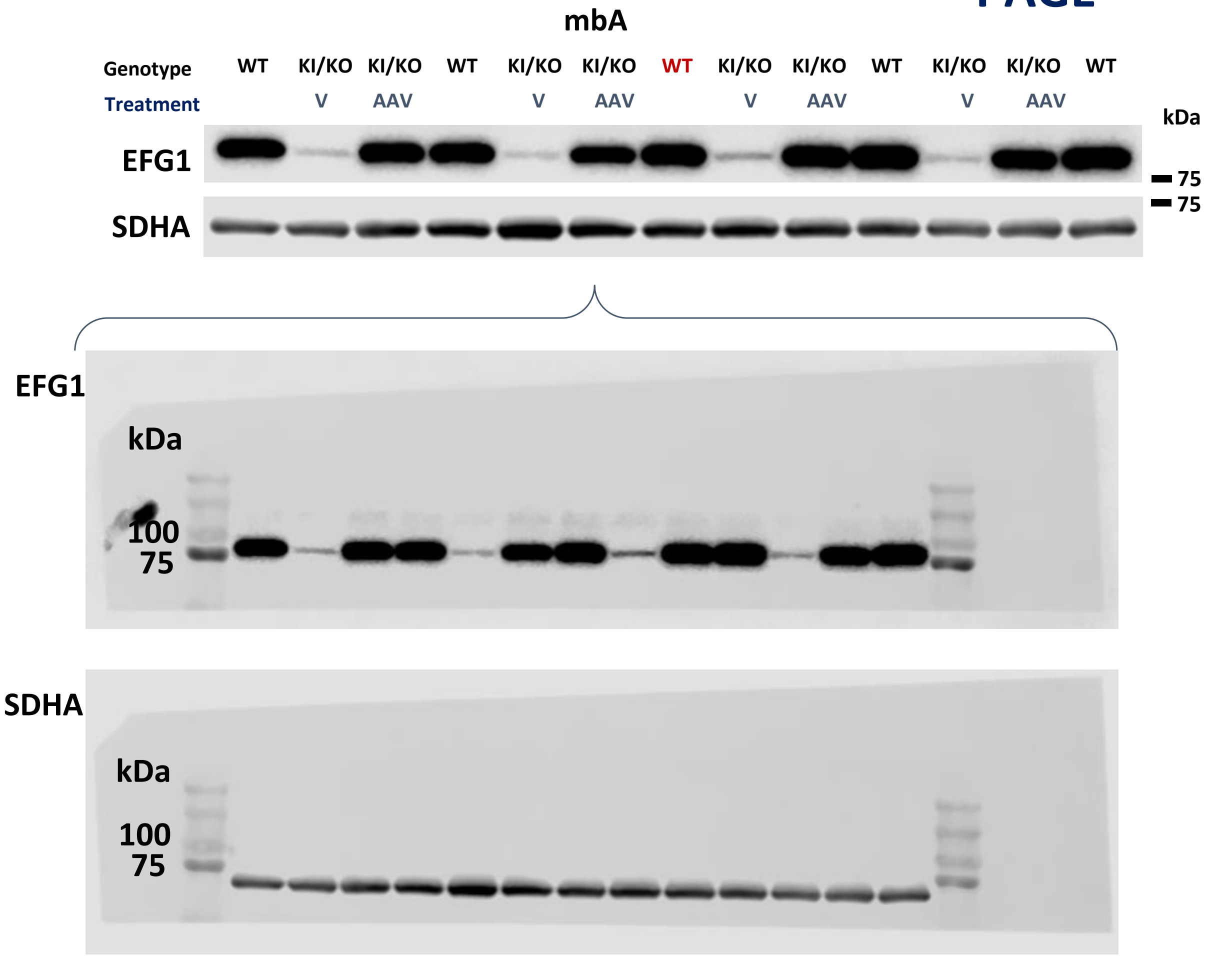

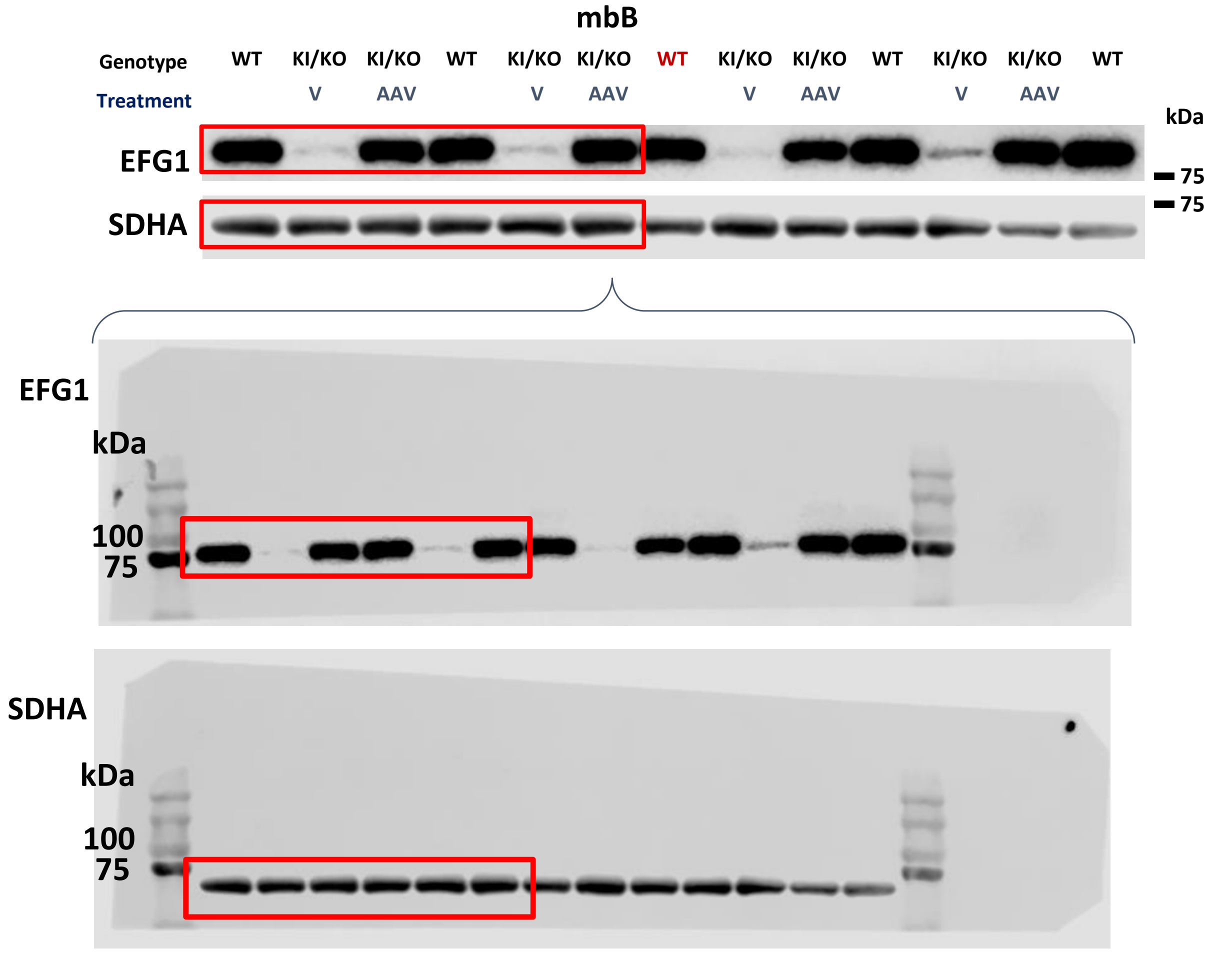

Selected area for publication
