# Supplementary material for: Systemic delivery of AAV-GFM1 corrects COXPD1 molecular alterations in Gfm1R671C/− mice
Source: EMBO Mol Med. 2026 Apr 17;18(6):2152–79. doi: 10.1038/s44321-026-00426-4 (PMC13269562; doi:10.1038/s44321-026-00426-4)
Supplement: Supplementary file 4 — Source data Fig. 3 [file 44321_2026_426_MOESM4_ESM.zip › Figure 3 updated/3A/Fig3A - WB mt Hi - CI and CIV Subunits.pdf]

Liver mitochondria
10 weeks old mice
ssAAV9-ApoE-hAAT-intron-GFM1

Females ♀

Western blot – SDS-PAGE

24/05/22

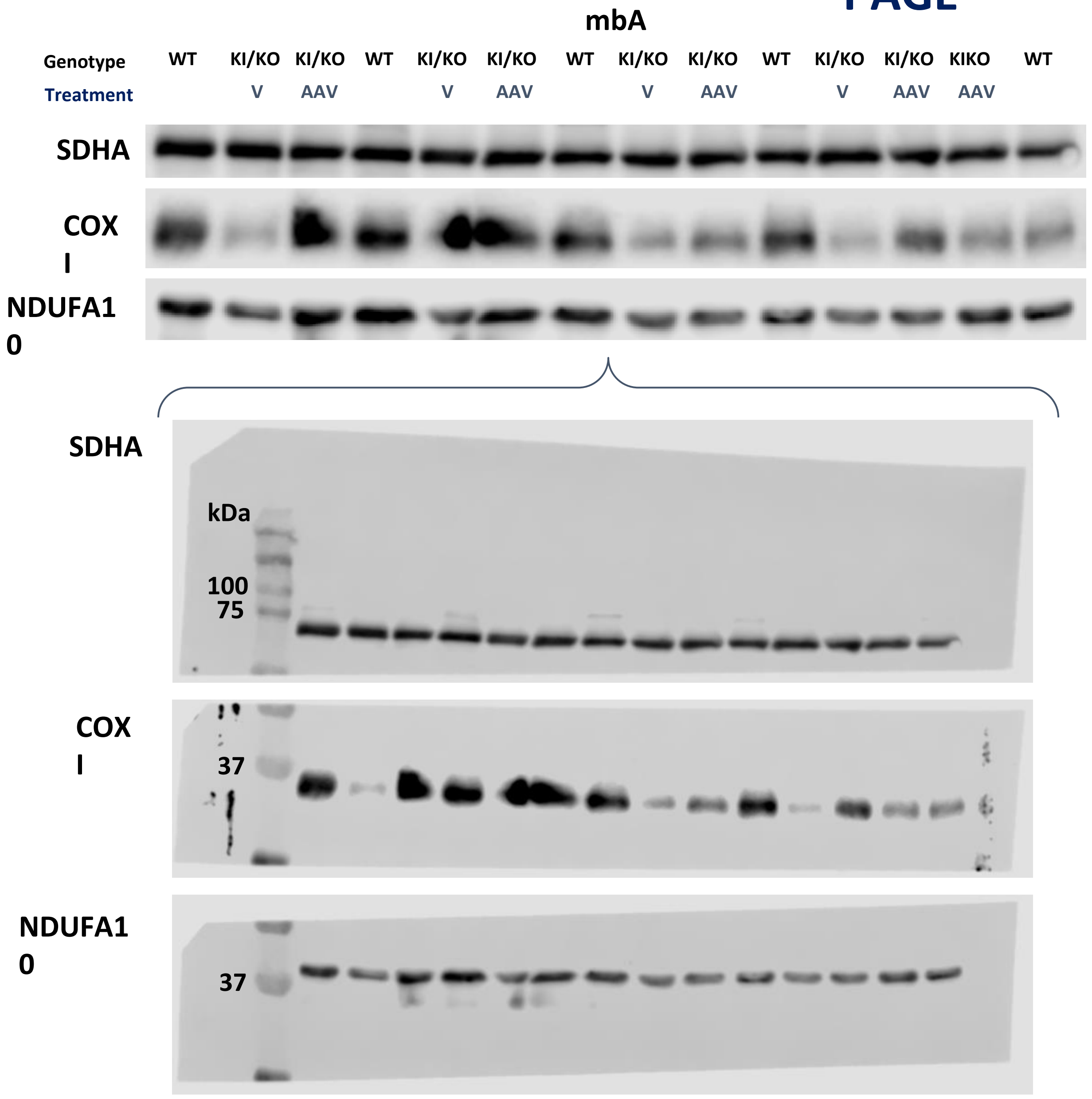

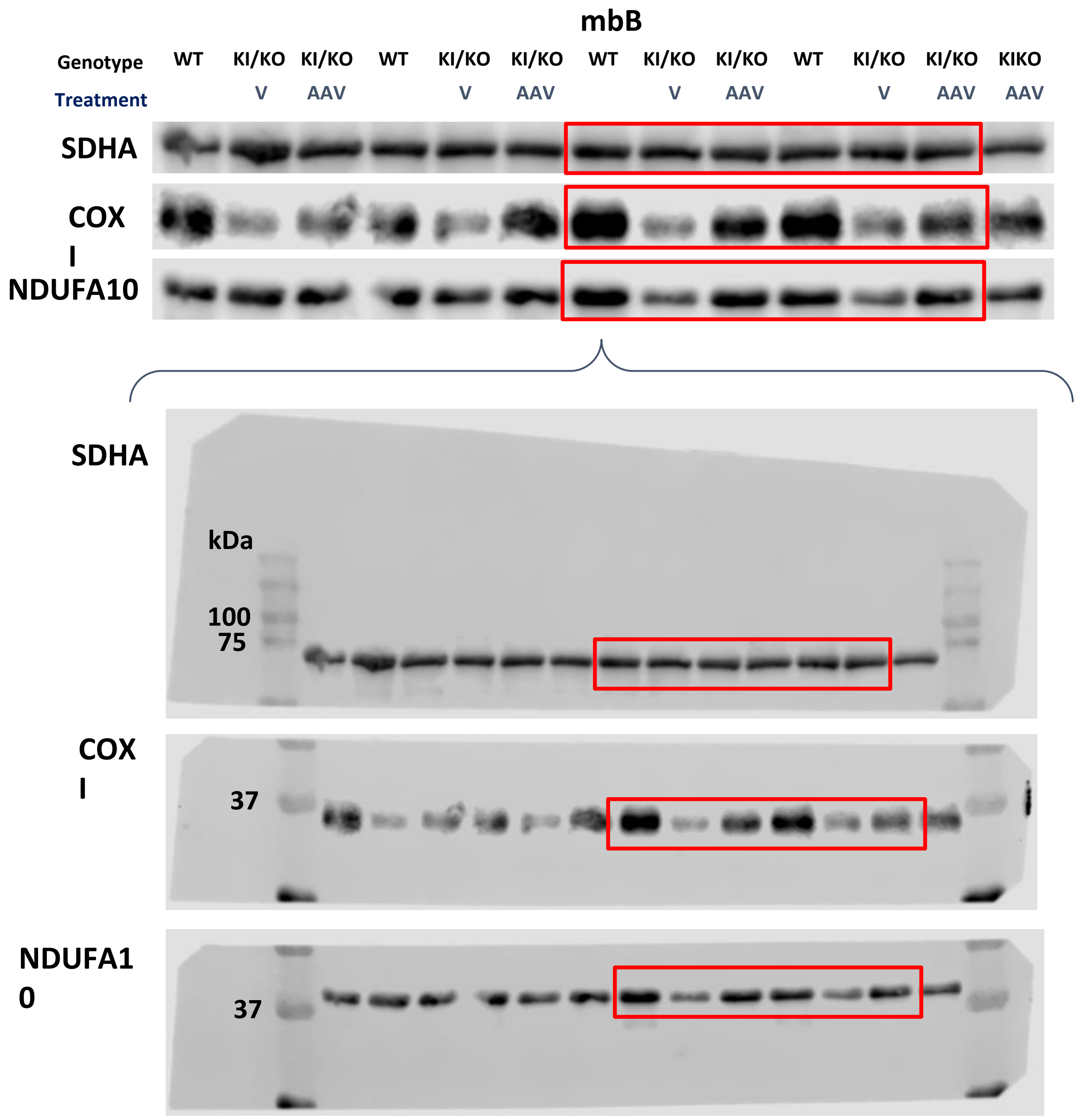

Selected area for publication

27/05/22

Liver mitochondria

Females ♀

Western blot – SDS-PAGE

10 weeks old mice
ssAAV9-ApoE-hAAT-intron-GFM1

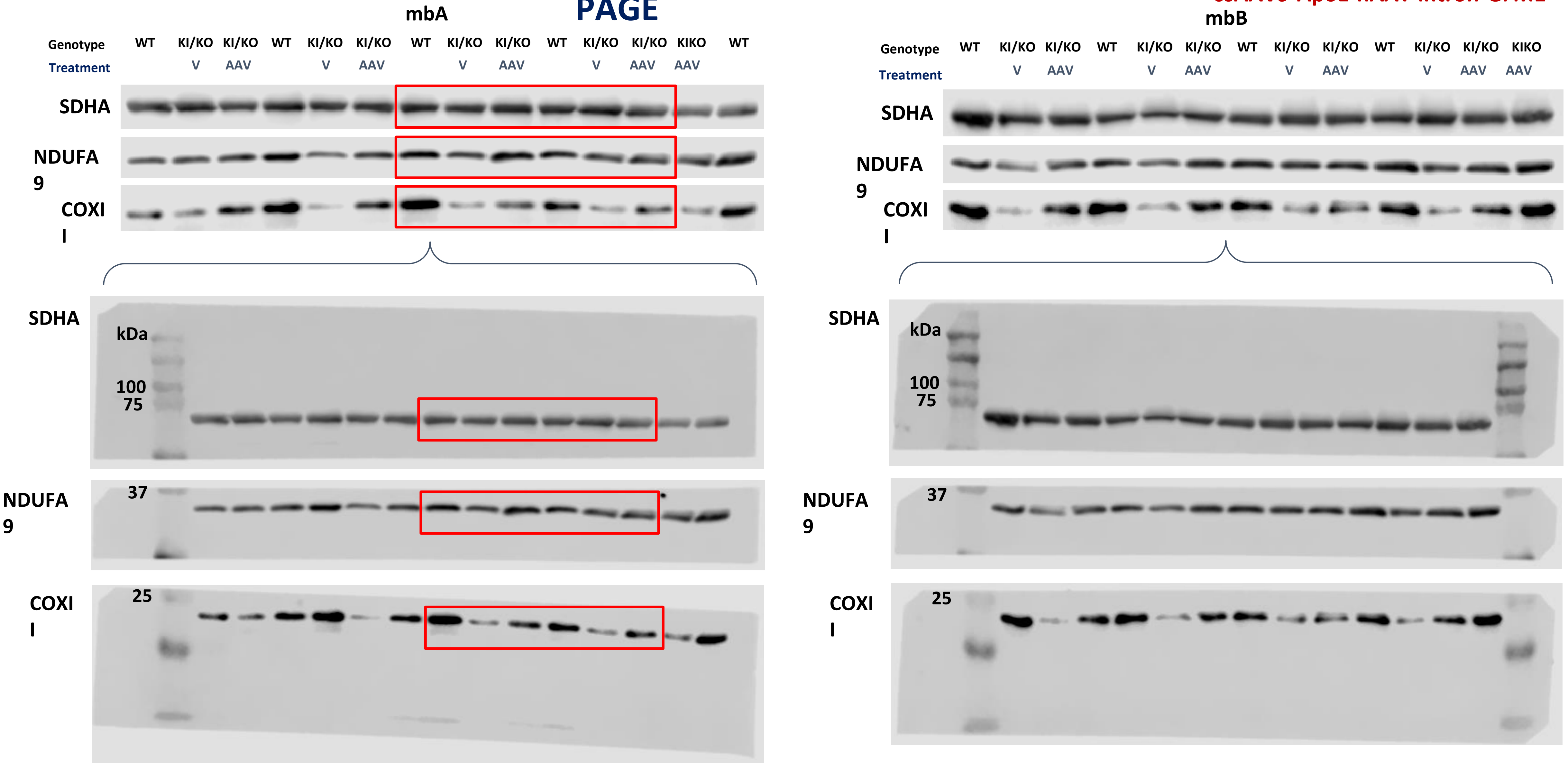

Selected area for publication

Liver mitochondria
10 weeks old mice
ssAAV9-ApoE-hAAT-intron-GFM1

Males ♂

Western blot – SDS-PAGE

15/06/22

Selected area for publication

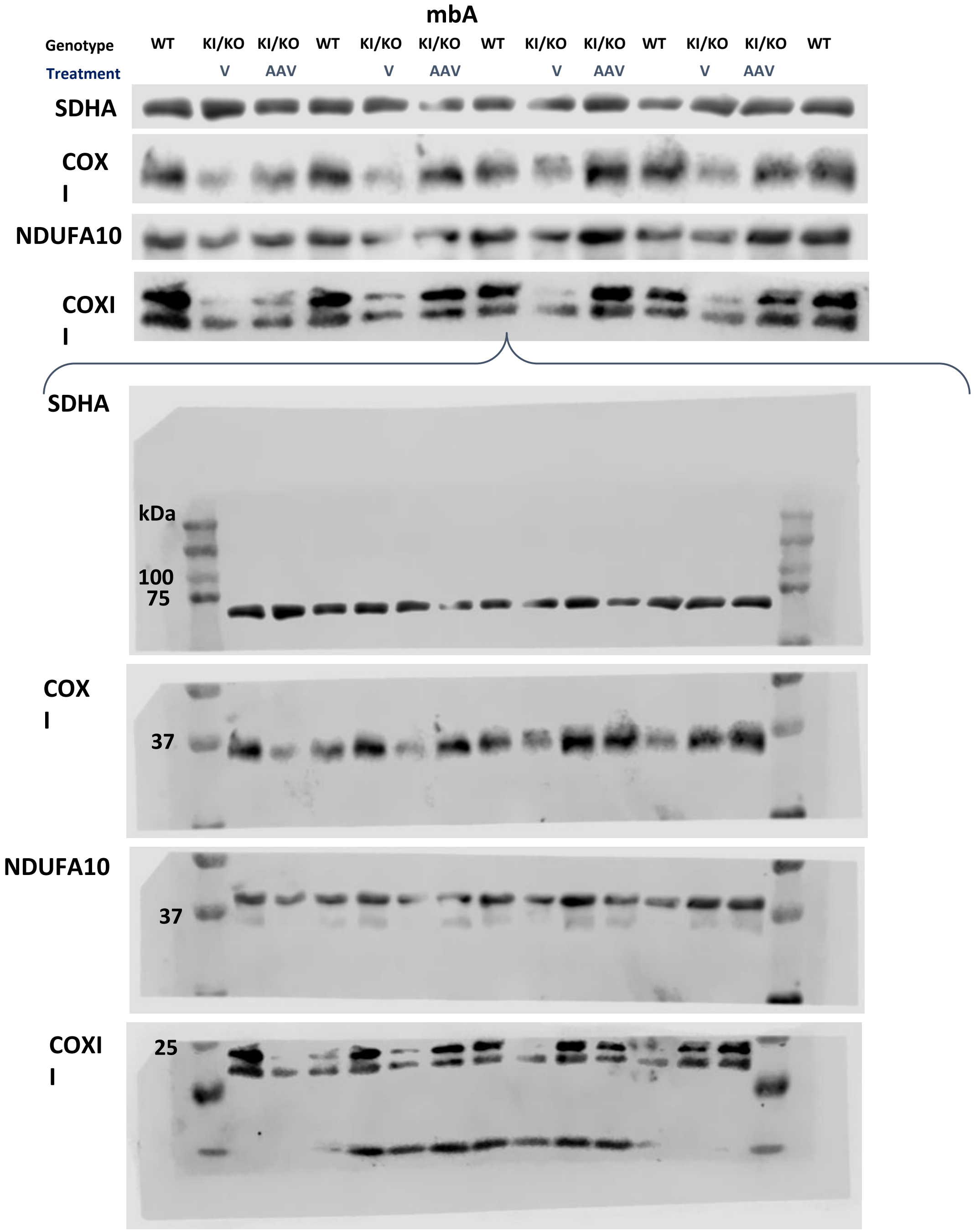

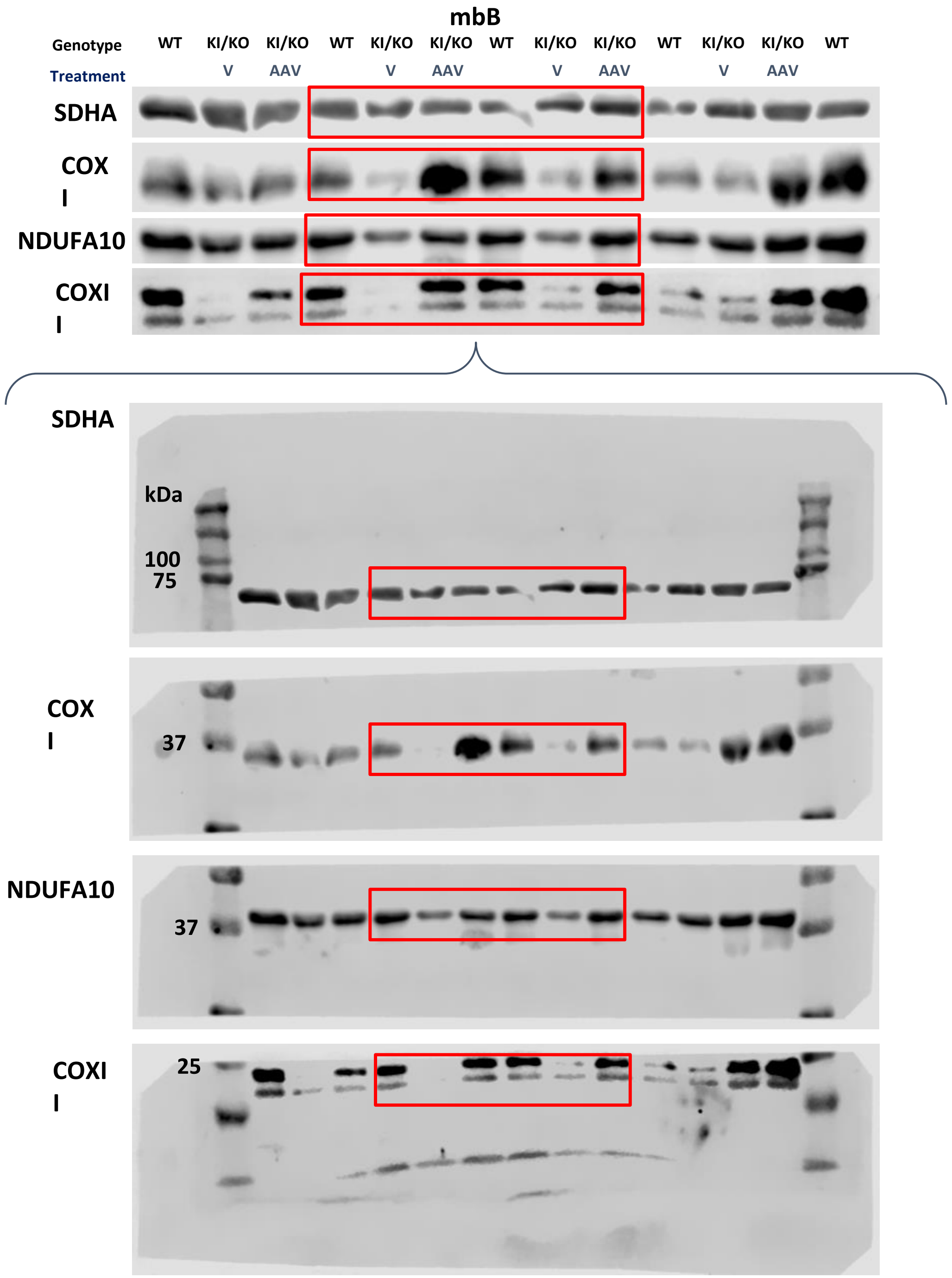

Liver mitochondria
10 weeks old mice
ssAAV9-ApoE-hAAT-intron-GFM1

Males ♂

Western blot – SDS-PAGE

20/06/22

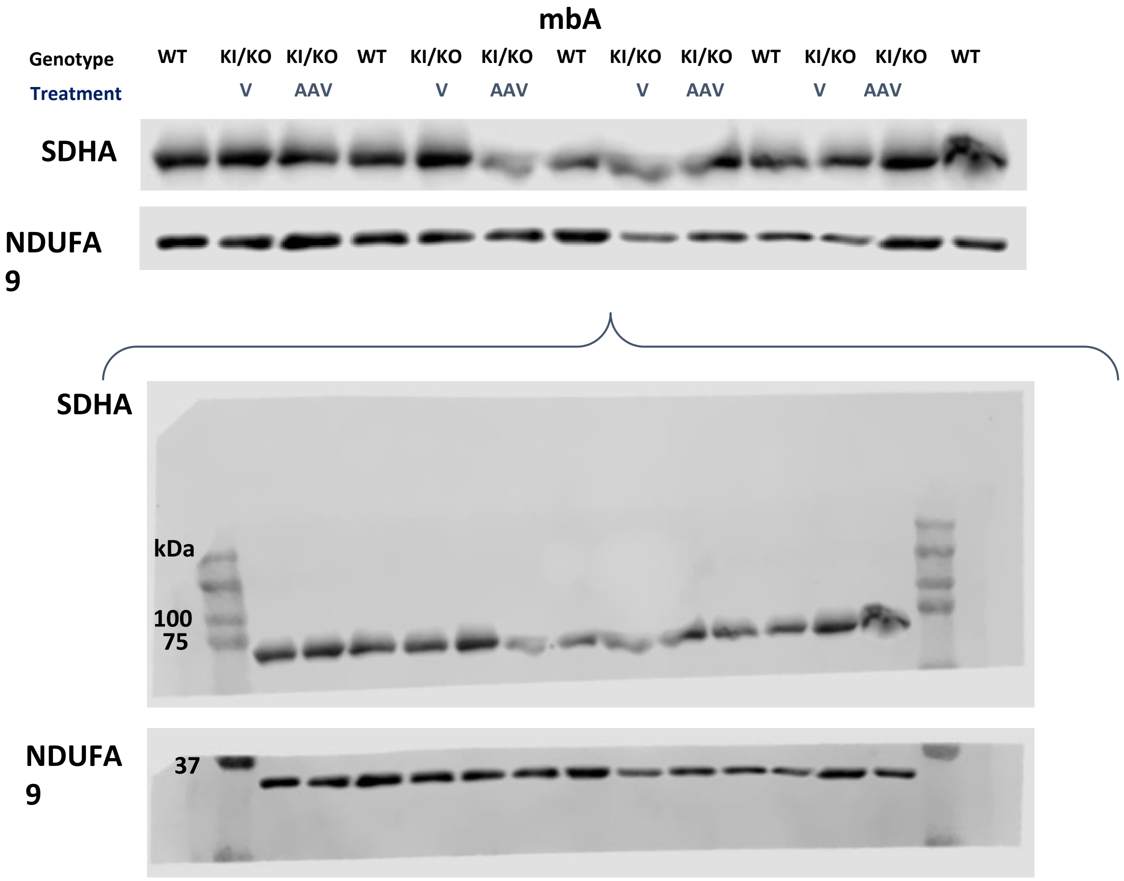

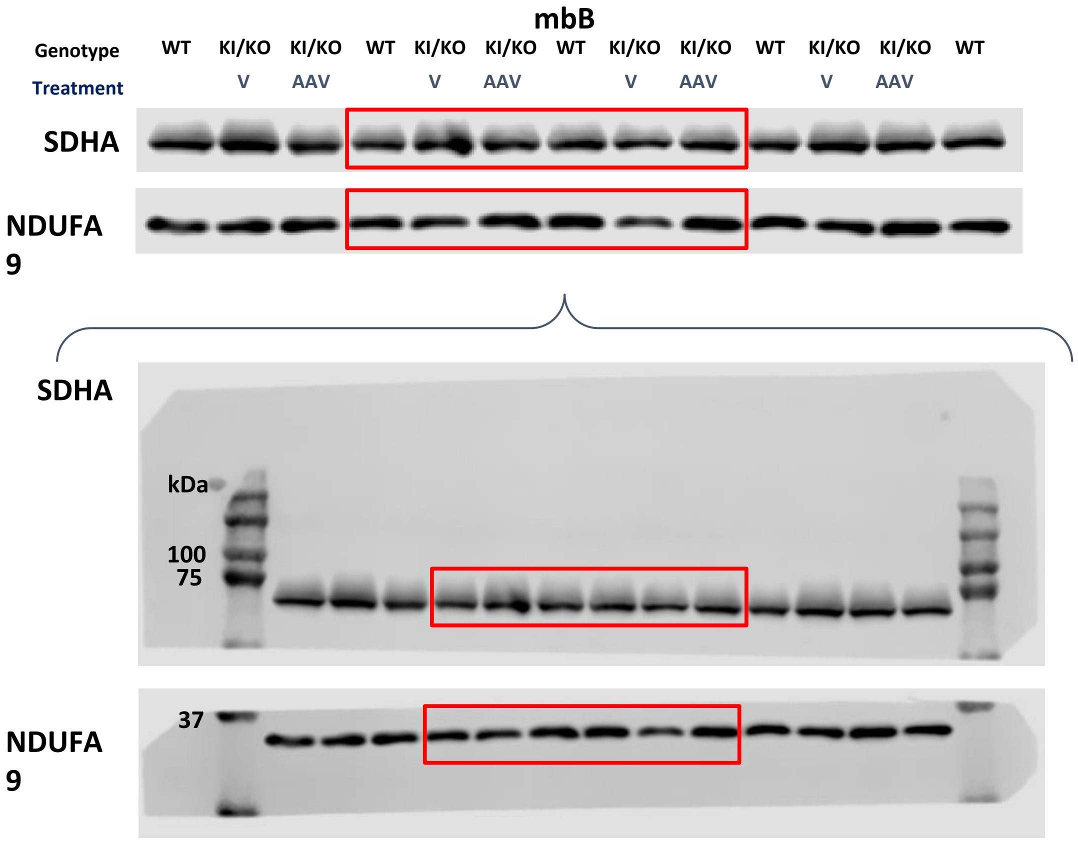

Selected area for publication
