# Supplementary material for: Systemic delivery of AAV-GFM1 corrects COXPD1 molecular alterations in Gfm1R671C/− mice
Source: EMBO Mol Med. 2026 Apr 17;18(6):2152–79. doi: 10.1038/s44321-026-00426-4 (PMC13269562; doi:10.1038/s44321-026-00426-4)
Supplement: Supplementary file 4 — Source data Fig. 3 [file 44321_2026_426_MOESM4_ESM.zip › Figure 3 updated/3B/Fig3B - WB mt ce Females and males V2.pdf]

Brain mitochondria

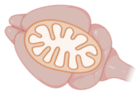

Females ♀

Western blot – SDS-PAGE

10 weeks old mice

ssAAV9P31-hSyn-GFM1

29/01/24

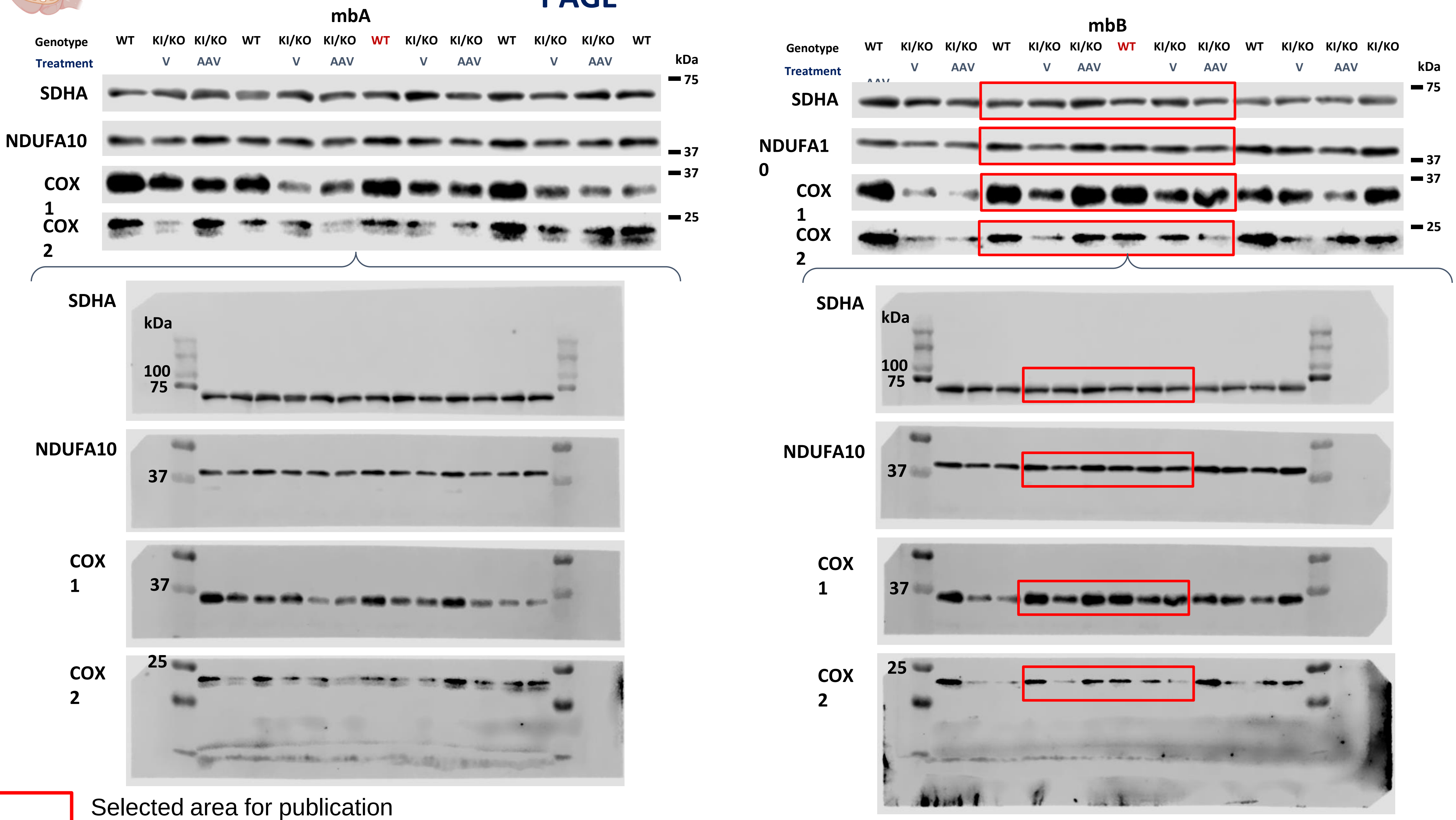

Selected area for publication

Brain mitochondria

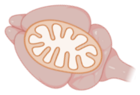

Females♀

Western blot – SDS-PAGE

10 weeks old mice
ssAAV9P31-hSyn-GFM1

18/04/24

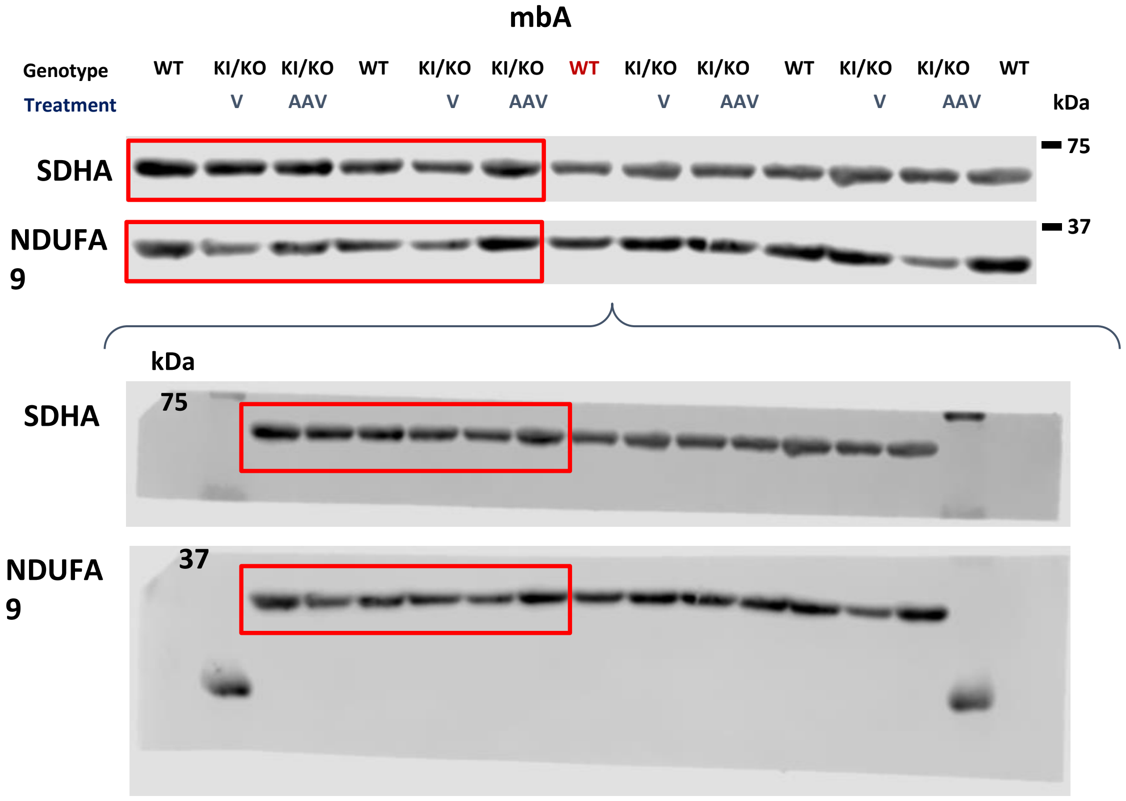

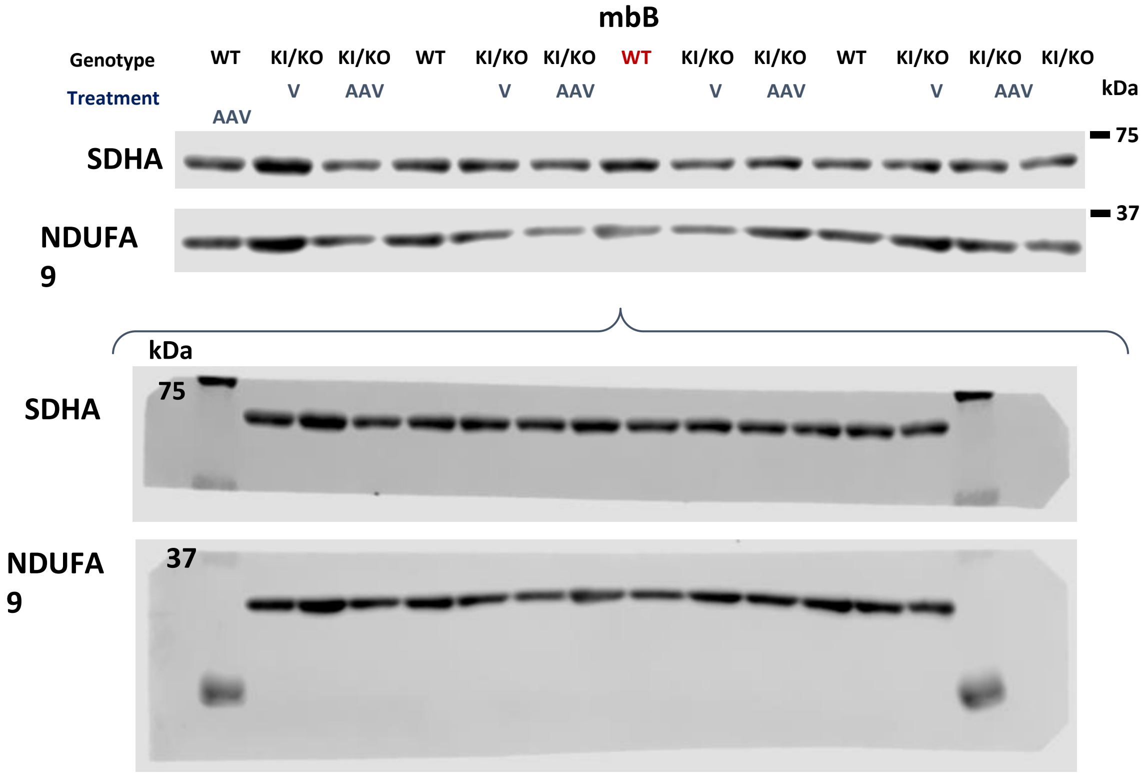

Selected area for publication

Brain mitochondria

Males ♂

Western blot – SDS-PAGE

10 weeks old mice
ssAAV9P31-hSyn-GFM1

22/02/24

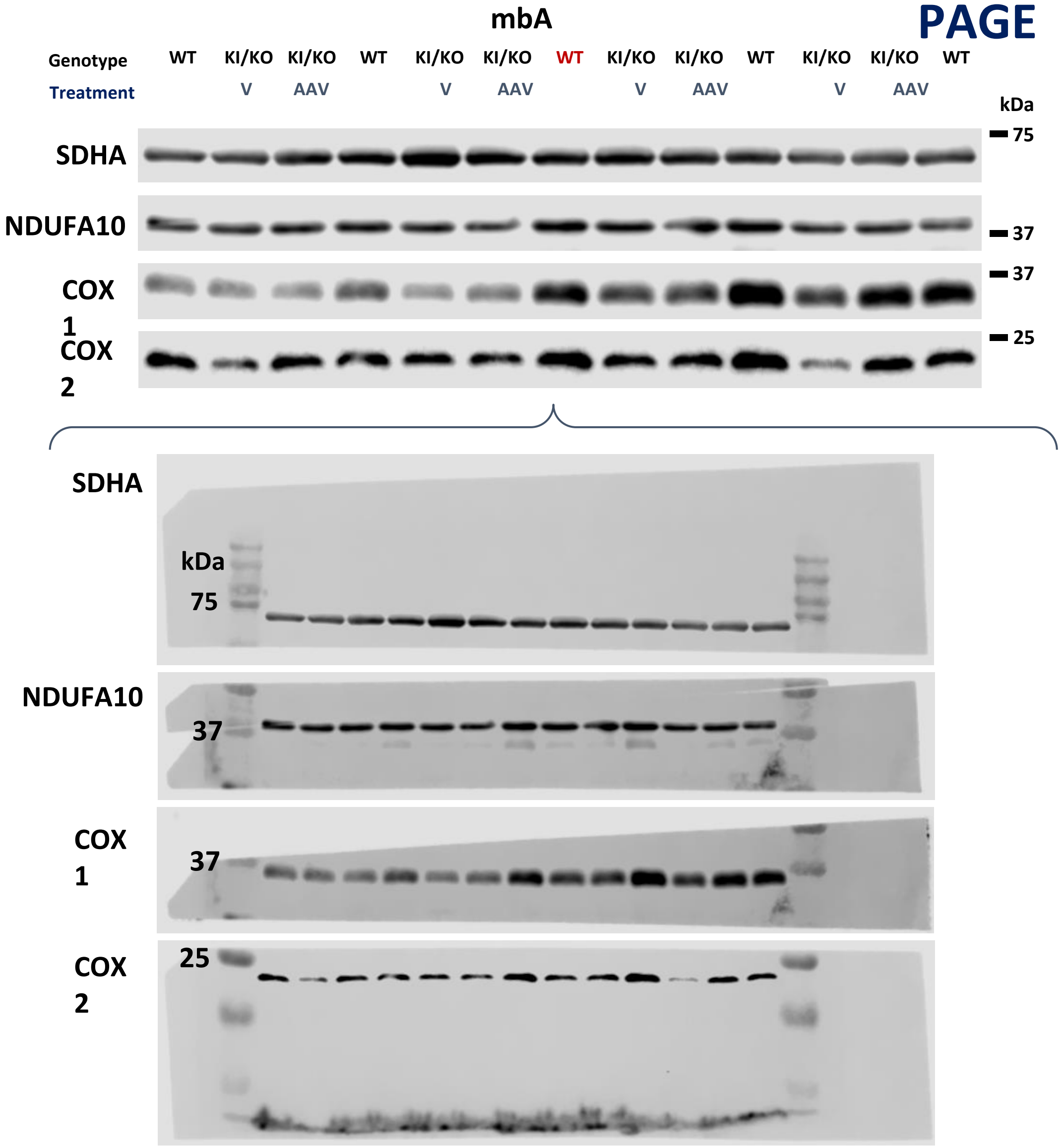

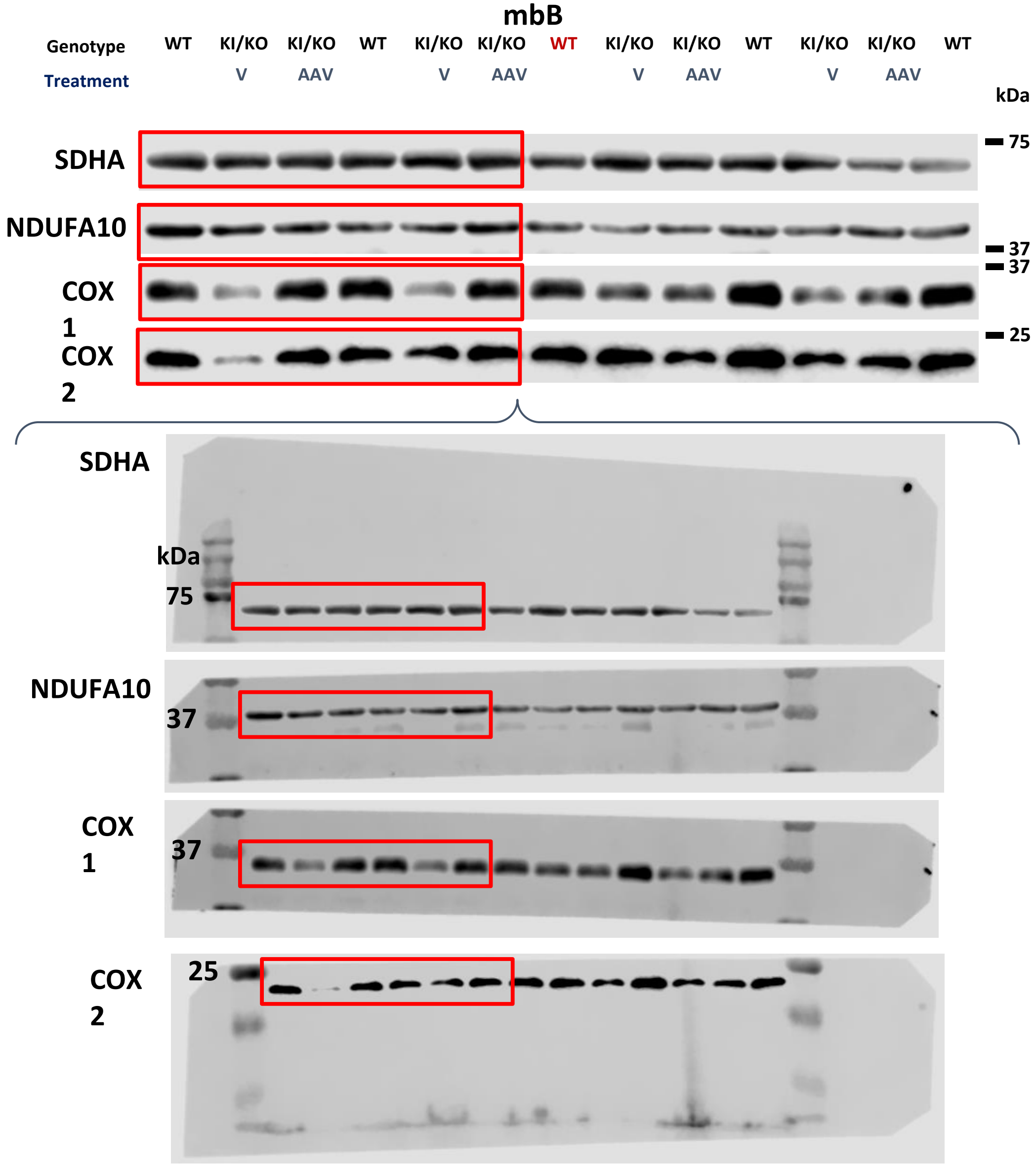

Selected area for publication

Brain mitochondria

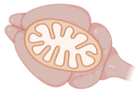

Males ♂

Western blot – SDS-PAGE

10 weeks old mice
ssAAV9P31-hSyn-GFM1

24/04/24

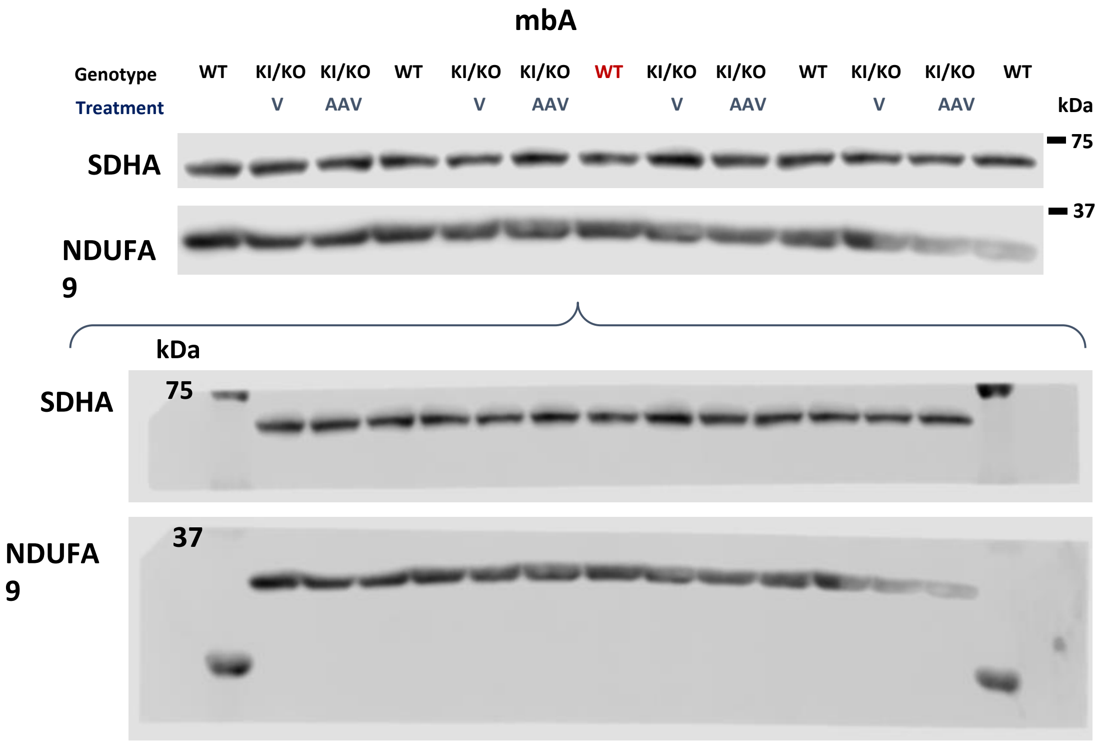

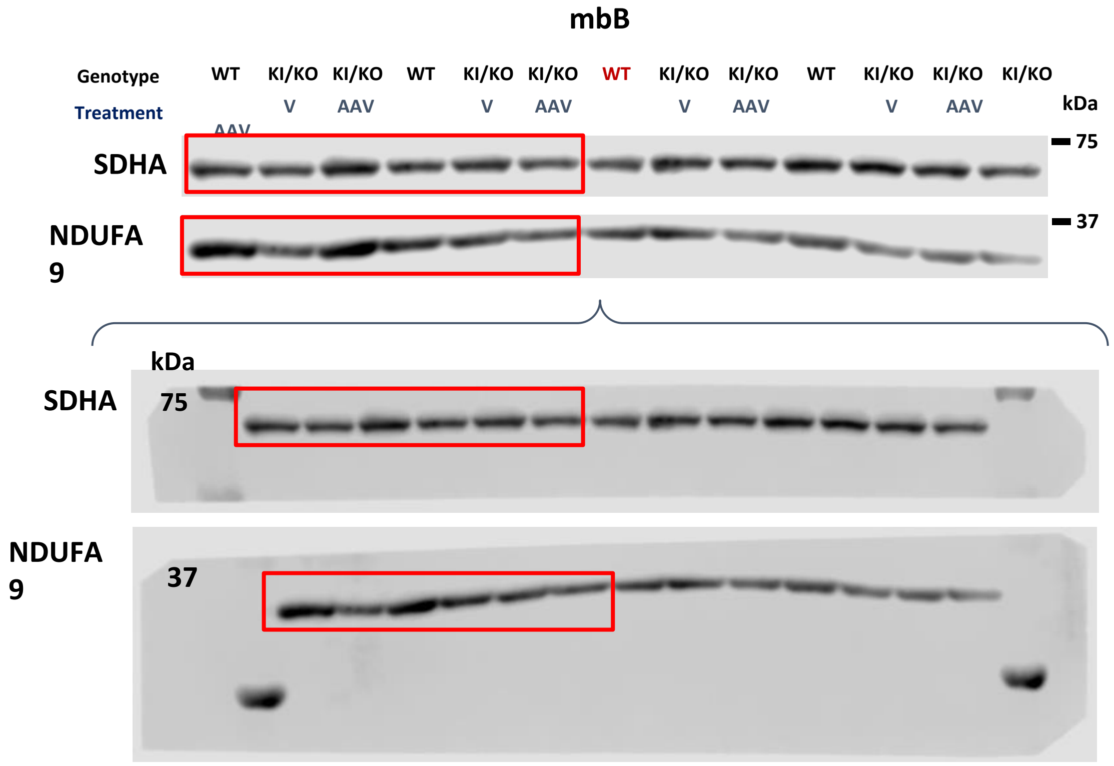

Selected area for publication
