# Supplementary material for: Systemic delivery of AAV-GFM1 corrects COXPD1 molecular alterations in Gfm1R671C/− mice
Source: EMBO Mol Med. 2026 Apr 17;18(6):2152–79. doi: 10.1038/s44321-026-00426-4 (PMC13269562; doi:10.1038/s44321-026-00426-4)

Females

BN-PAGE Assembled OXPHOS Complexes in liver mitochondria

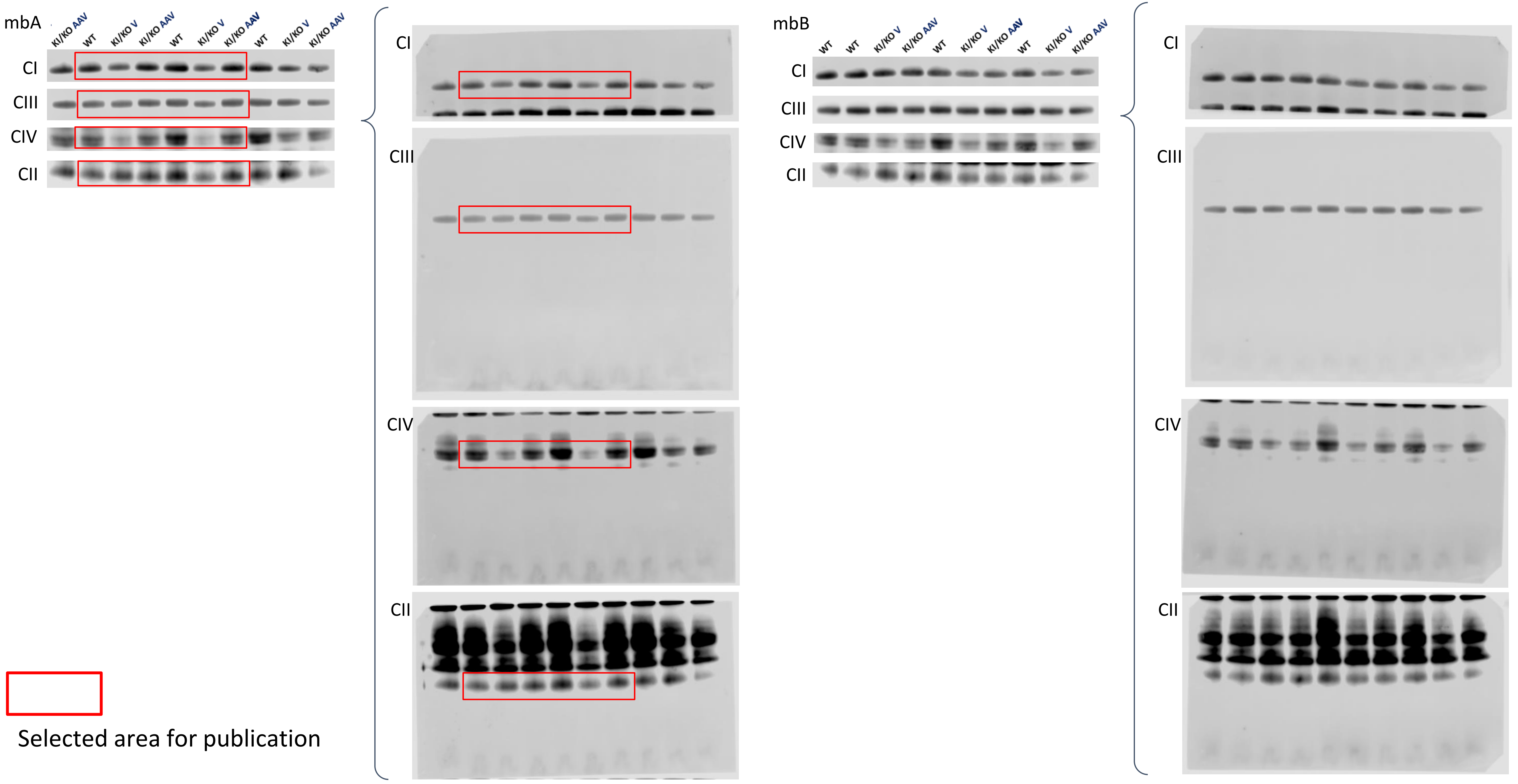

Males

BN-PAGE Assembled OXPHOS Complexes in liver mitochondria

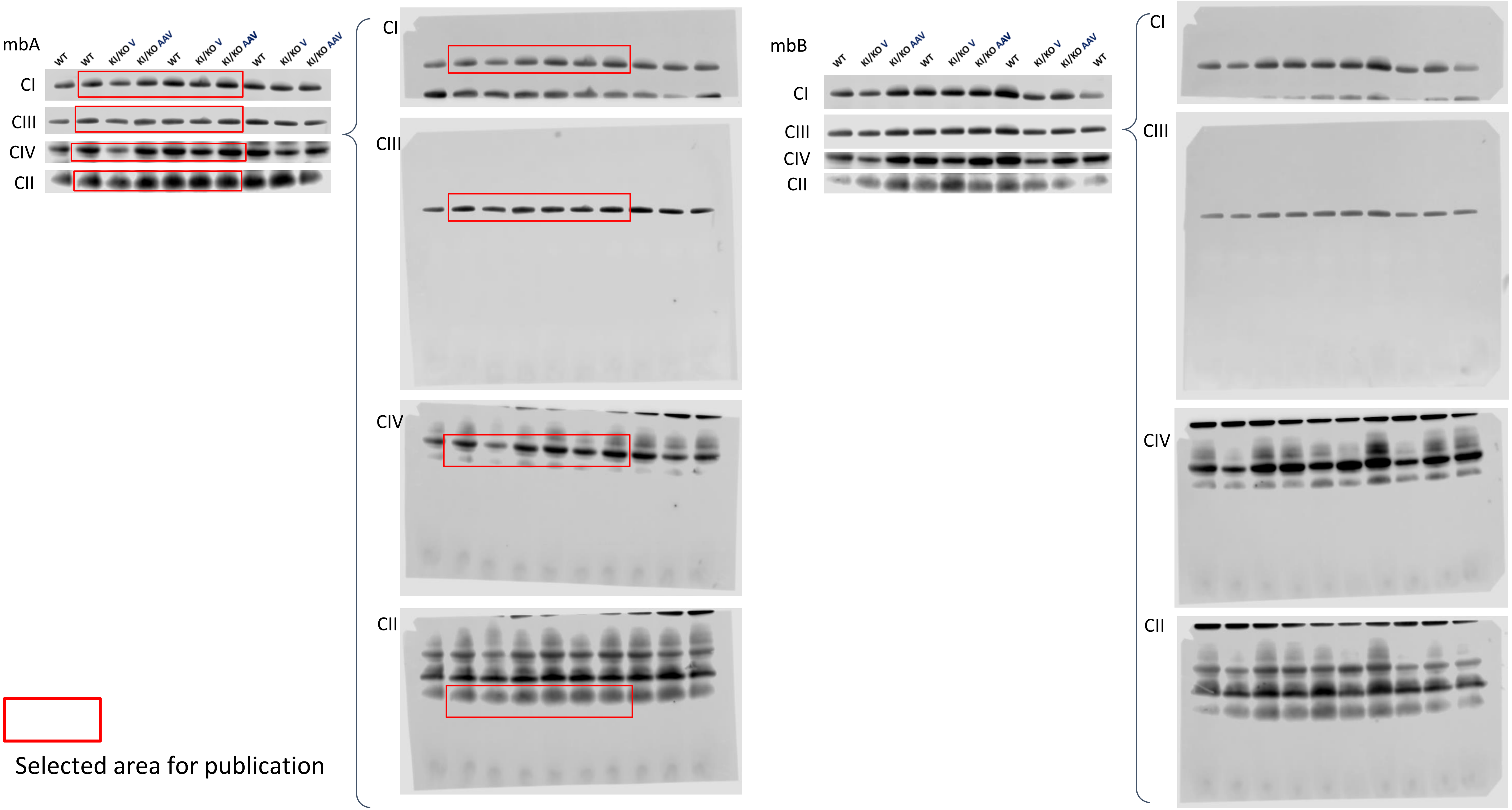

Supplement: Supplementary file 5 — Source data Fig. 4 [file 44321_2026_426_MOESM5_ESM.zip › Figure 4 updated/4A/Fig4A - BN-PAGE in liver mitochondria .pdf]
