# Supplementary material for: Systemic delivery of AAV-GFM1 corrects COXPD1 molecular alterations in Gfm1R671C/− mice
Source: EMBO Mol Med. 2026 Apr 17;18(6):2152–79. doi: 10.1038/s44321-026-00426-4 (PMC13269562; doi:10.1038/s44321-026-00426-4)
Supplement: Supplementary file 6 — Source data Fig. 5 [file 44321_2026_426_MOESM6_ESM.zip › Figure 5 updated/5A/Fig5A - BN-PAGE mt ce AAV9P31-hSyn-GFM1.pdf]

Western blot – BN-

Brain mitochondria

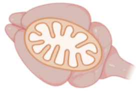

Females ♀

10 weeks old mice

ssAAV9P31-hSyn-GFM1

27/02/24

mbA

Genotype: WT | KI/KO | KI/KO | WT | KI/KO | KI/KO | WT | KI/KO | KI/KO | WT | KI/KO | KI/KO | WT

Treatment: | V | AAV | | V | AAV | | V | AAV | | V | AAV |

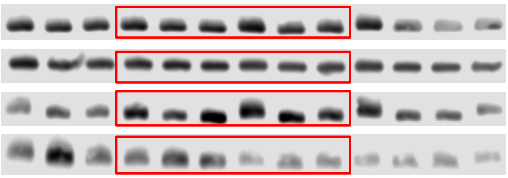

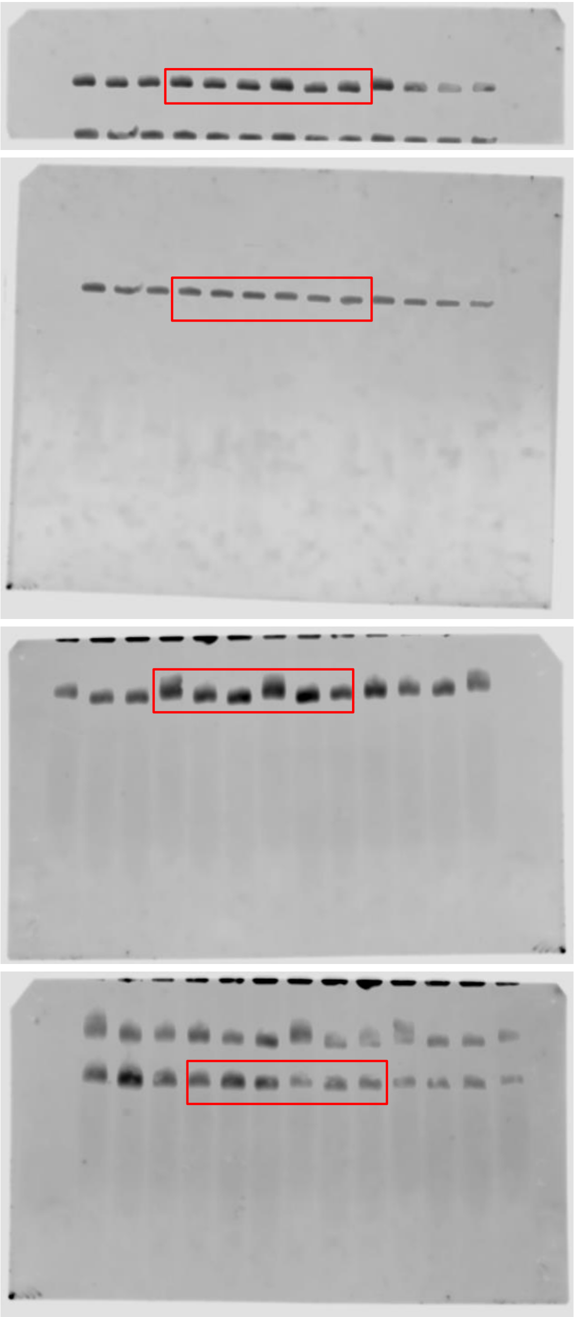

mbB

Genotype: WT | KI/KO | KI/KO | WT | KI/KO | KI/KO | WT | KI/KO | KI/KO | WT | KI/KO | KI/KO | WT | KI/KO

Treatment: AAV | V | AAV | | V | AAV | | V | AAV | | V | AAV | |

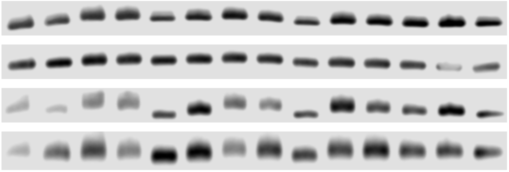

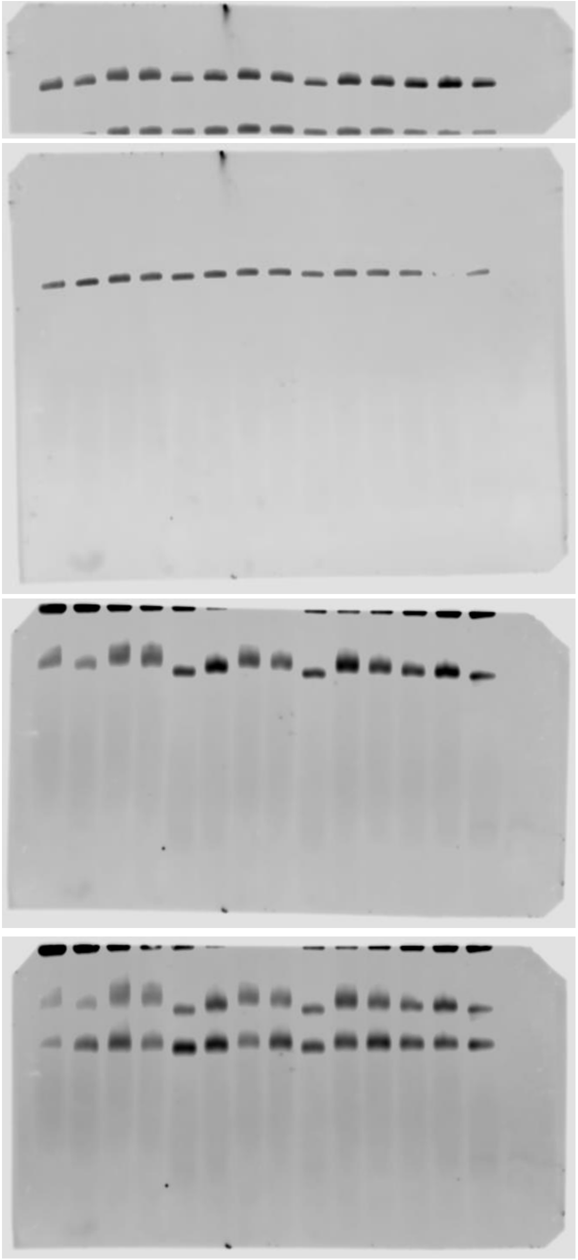

Selected area for publication

Brain mitochondria

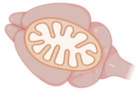

Males ♂

Western blot – BN-PAGE

10 weeks old mice
ssAAV9P31-hSyn-GFM1

29/02/24

mbA

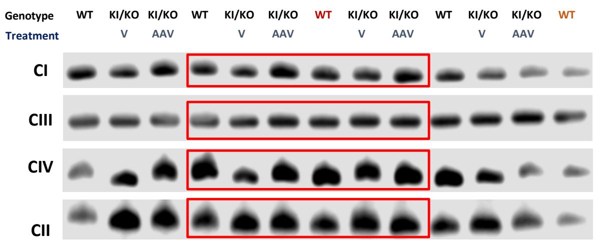

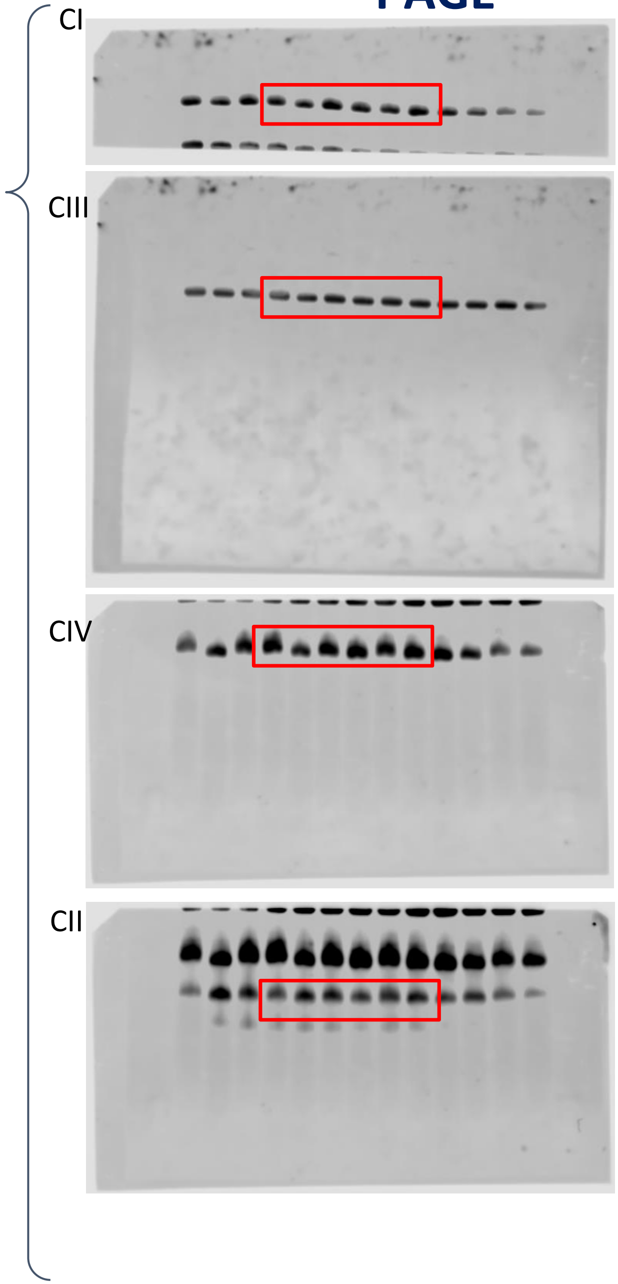

mbB

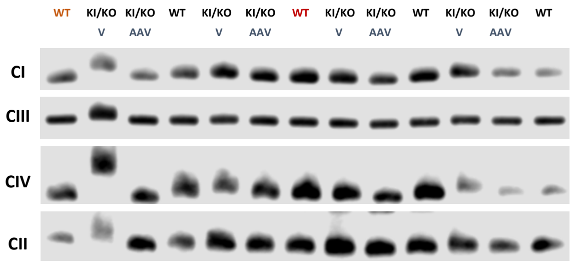

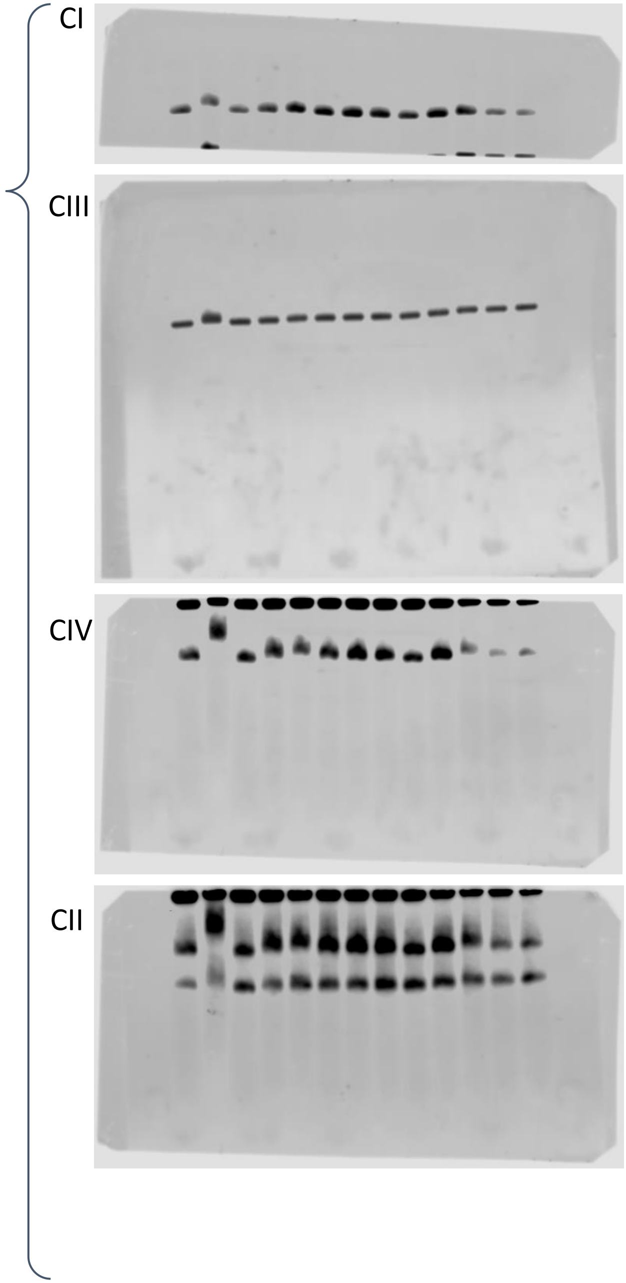

Selected area for publication
