# Supplementary material for: Systemic delivery of AAV-GFM1 corrects COXPD1 molecular alterations in Gfm1R671C/− mice
Source: EMBO Mol Med. 2026 Apr 17;18(6):2152–79. doi: 10.1038/s44321-026-00426-4 (PMC13269562; doi:10.1038/s44321-026-00426-4)
Supplement: Supplementary file 7 — Source data Fig. 6 [file 44321_2026_426_MOESM7_ESM.zip › Figure 6 updated/6A/Fig6A - WB MRPs mt Hi V2.pdf]

22/08/23

Liver mitochondria

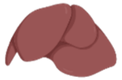

Females ♀

Western blot – SDS-PAGE

10 weeks old mice
ssAAV9-ApoE-hAAT-intron-GFM1

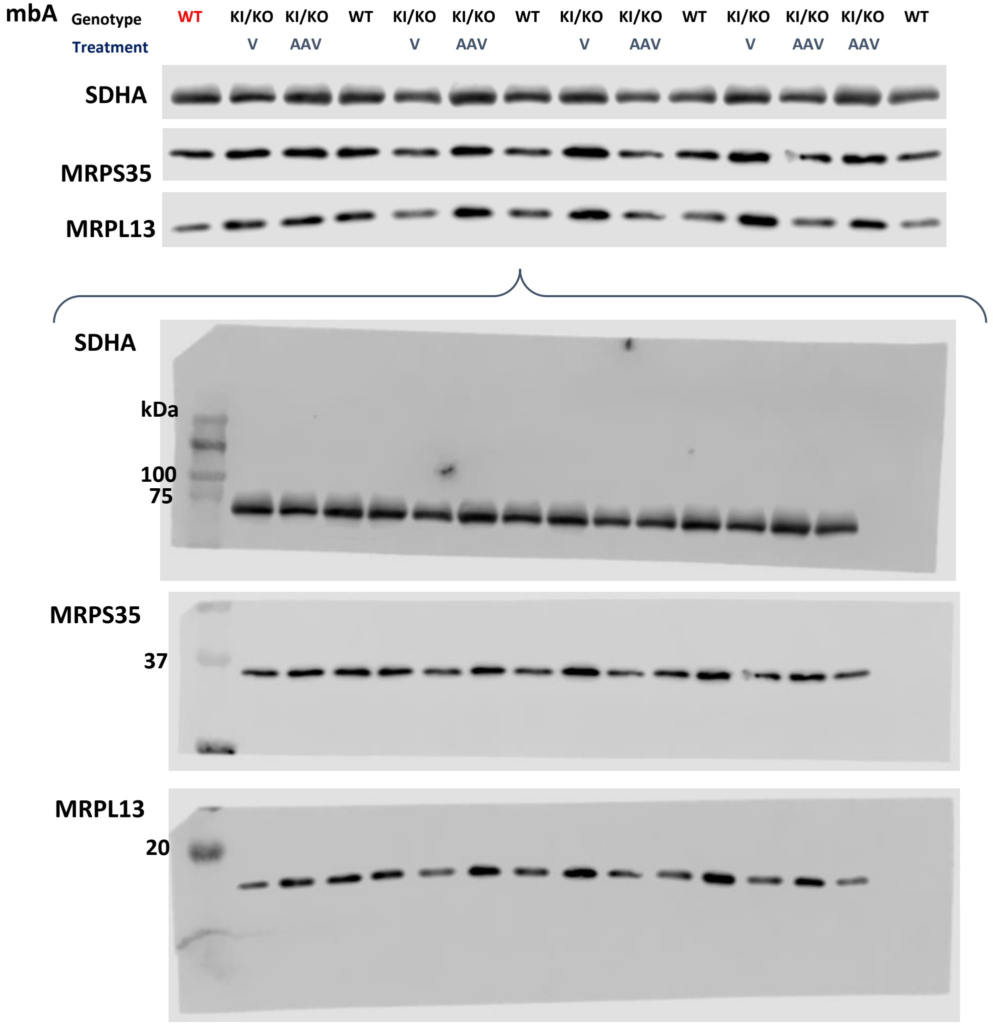

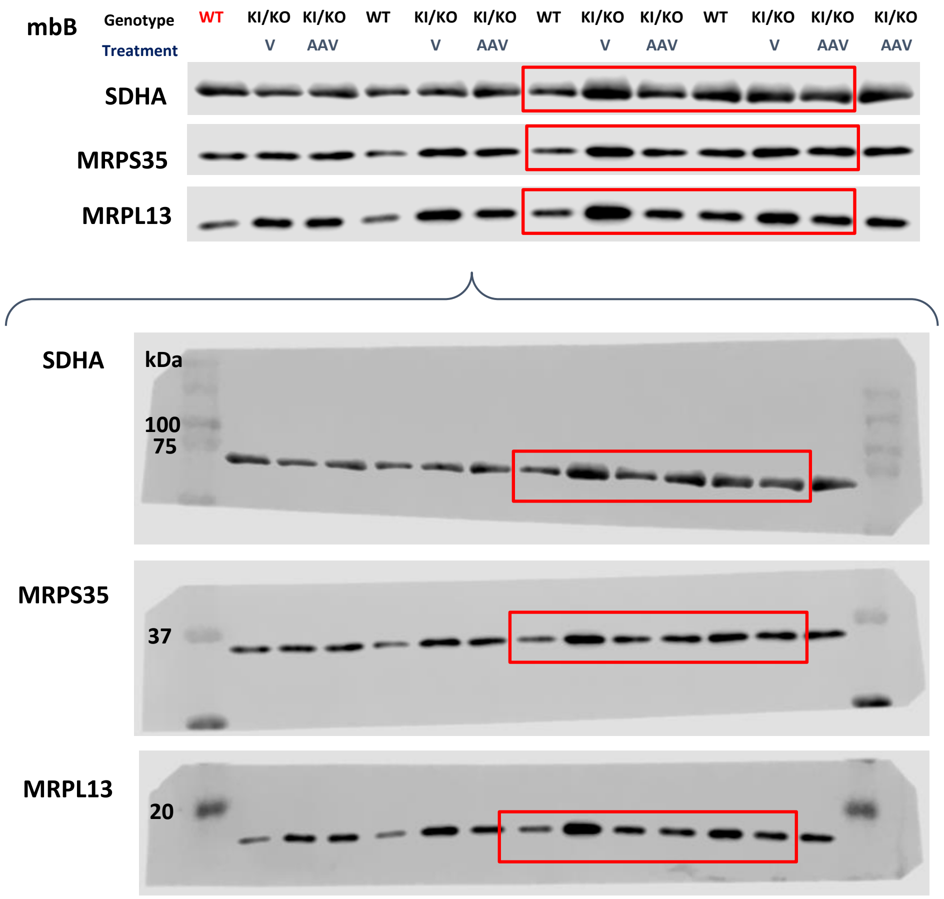

Selected area for publication

22/08/23

Liver mitochondria

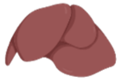

Females ♀

Western blot – SDS-PAGE

10 weeks old mice
ssAAV9-ApoE-hAAT-intron-GFM1

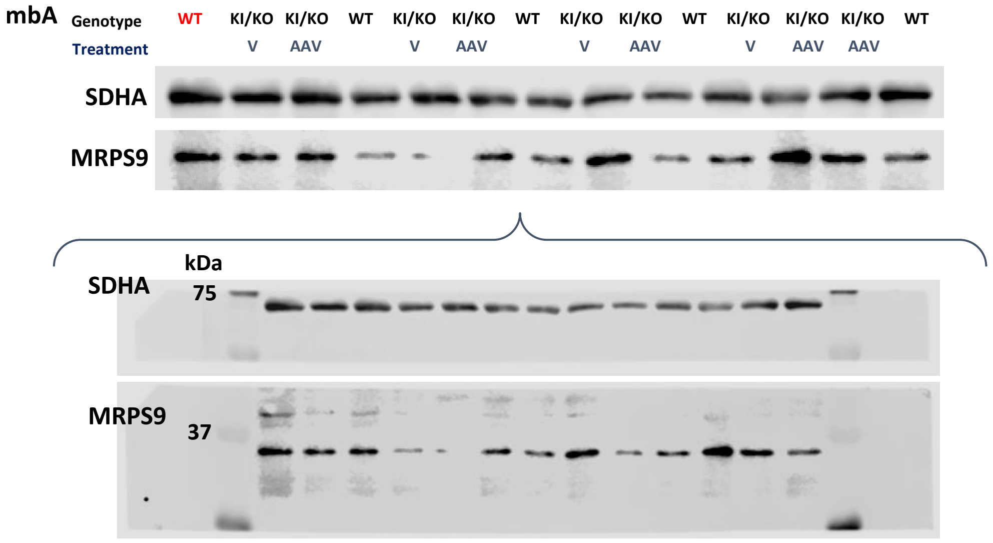

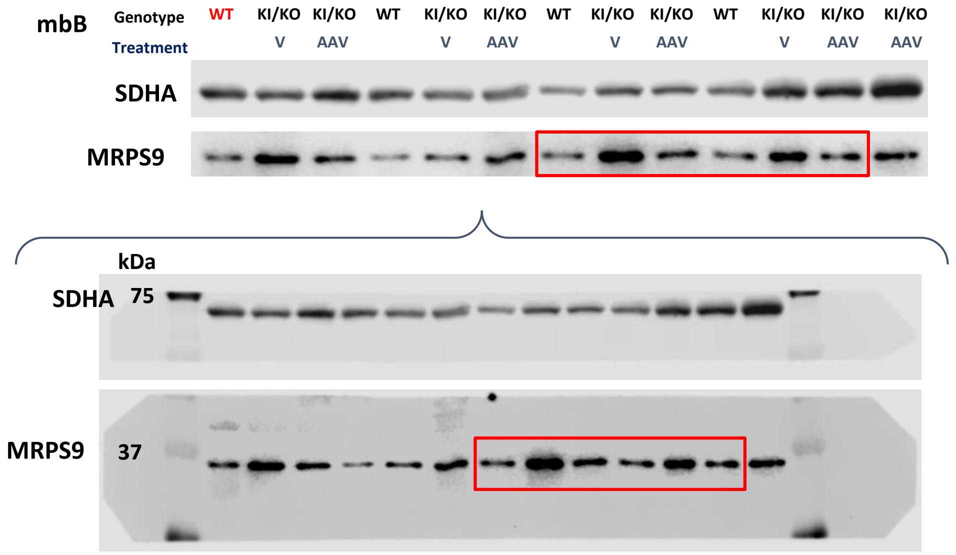

Selected area for publication

Liver mitochondria

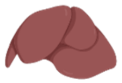

Western blot – SDS-PAGE Females ♀

10 weeks old mice
ssAAV9-ApoE-hAAT-intron-GFM1

30/04/24

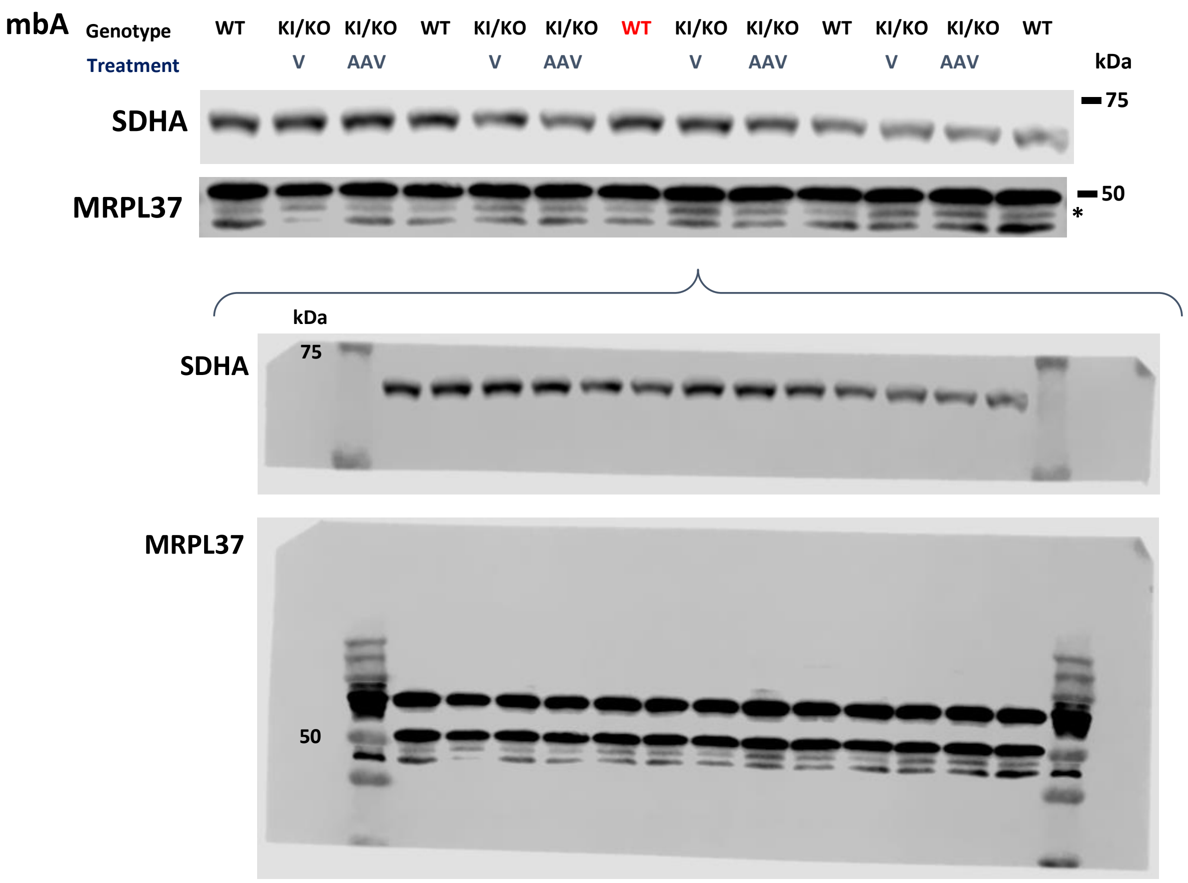

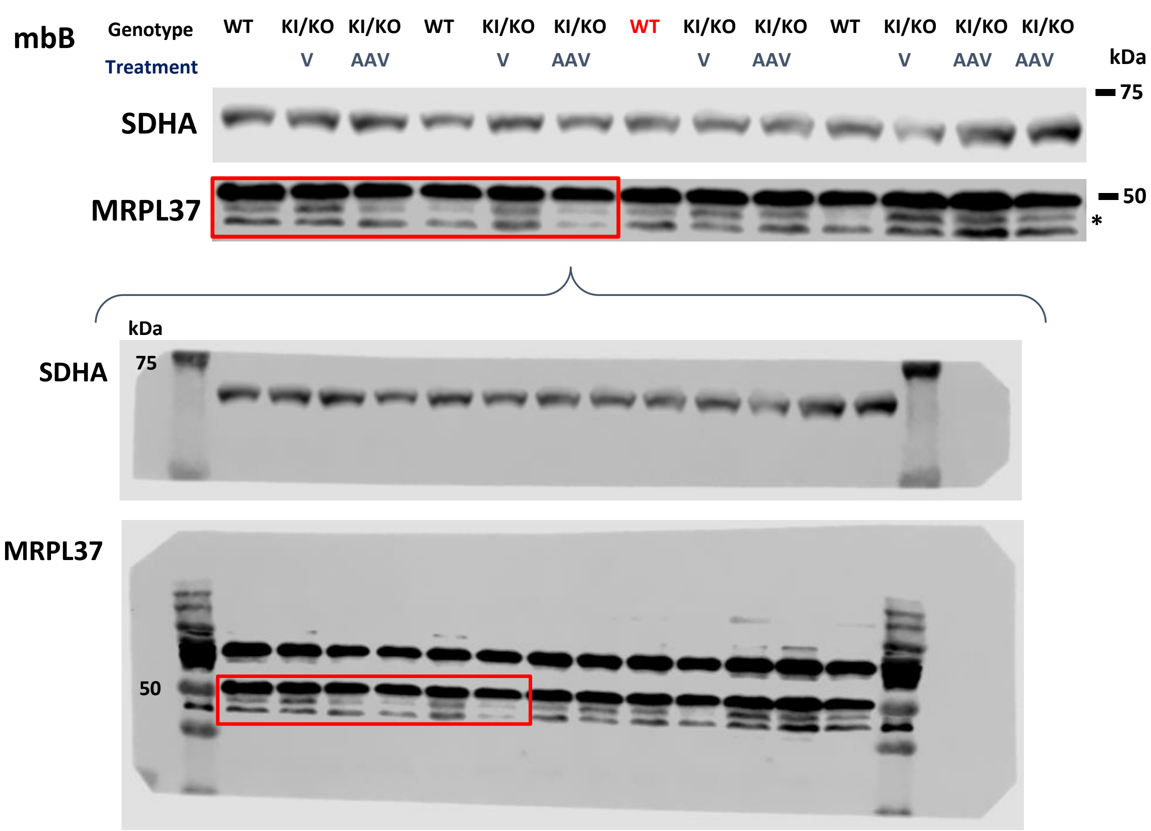

Selected area for publication

22/08/23

Liver mitochondria

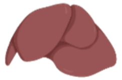

Males ♂

Western blot – SDS-PAGE

10 weeks old mice
ssAAV9-ApoE-hAAT-intron-GFM1

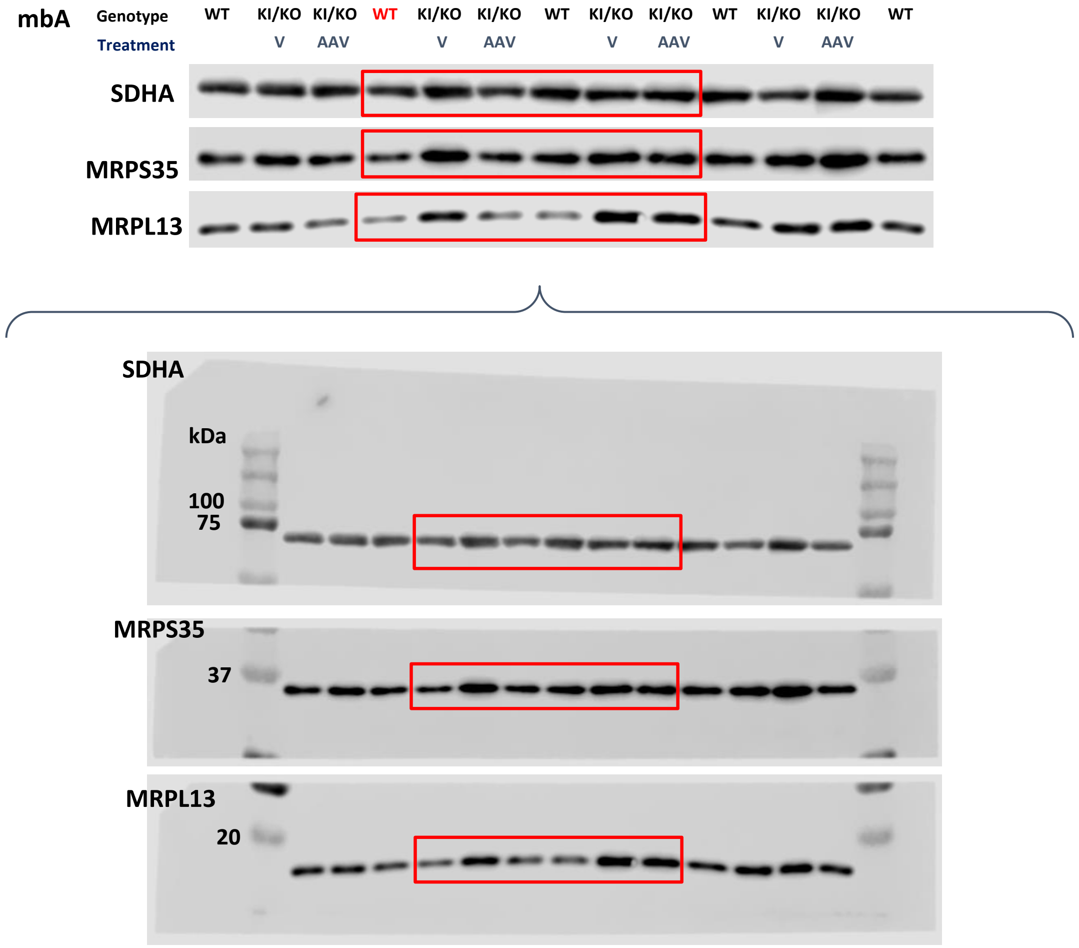

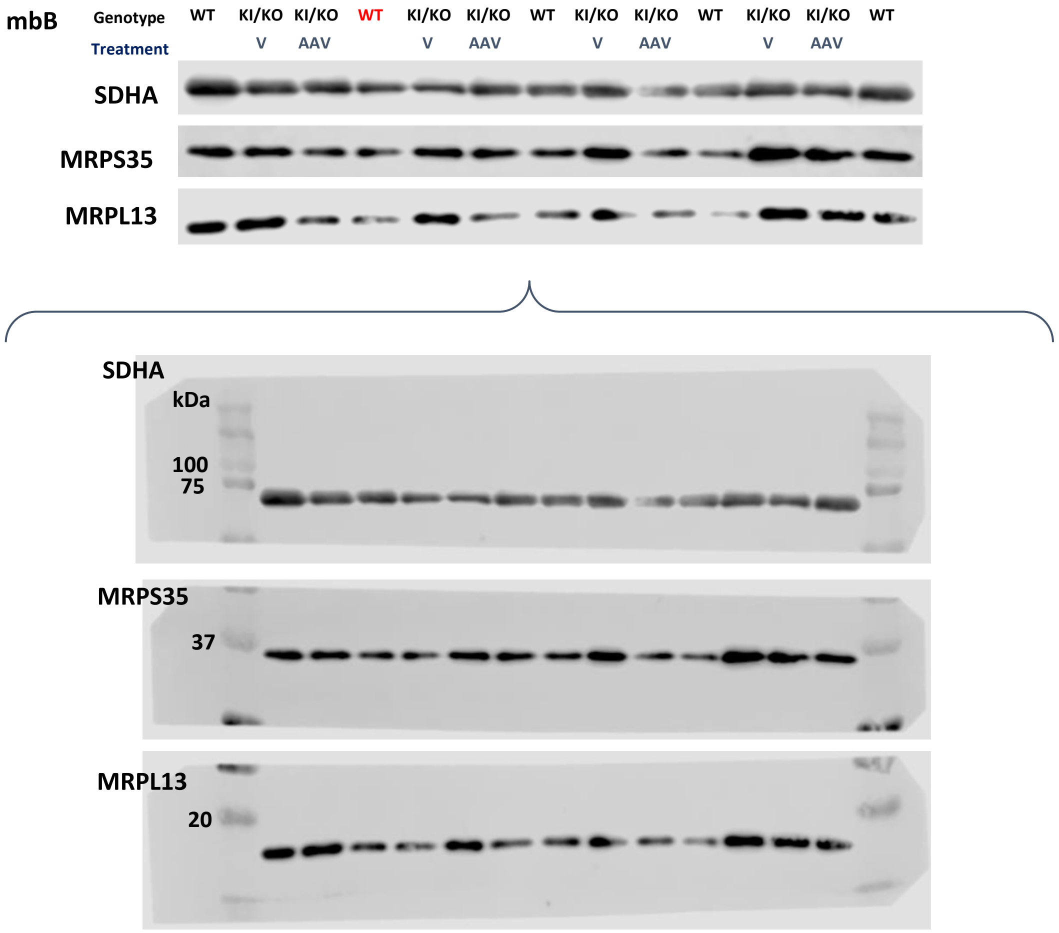

Selected area for publication

Liver mitochondria

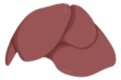

Males ♂

Western blot – SDS-PAGE

10 weeks old mice
ssAAV9-ApoE-hAAT-intron-GFM1

22/08/23

mbA

Genotype	WT	KI/KO	KI/KO	WT	KI/KO	KI/KO	WT	KI/KO	KI/KO	WT	KI/KO	KI/KO	WT
Treatment		V	AAV		V	AAV		V	AAV		V	AAV	

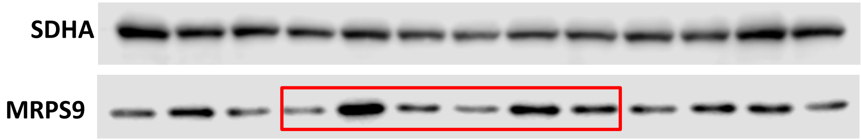

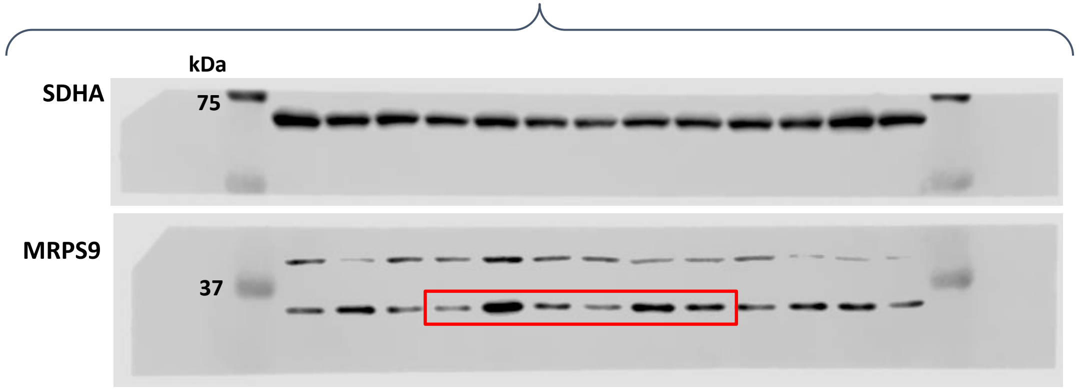

mbB

Genotype	WT	KI/KO	KI/KO	WT	KI/KO	KI/KO	WT	KI/KO	KI/KO	WT	KI/KO	KI/KO	WT
Treatment		V	AAV		V	AAV		V	AAV		V	AAV	

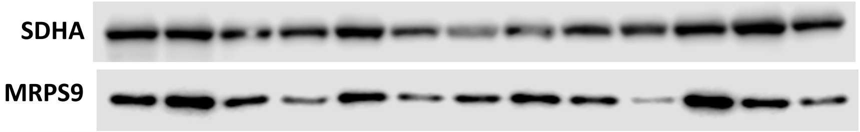

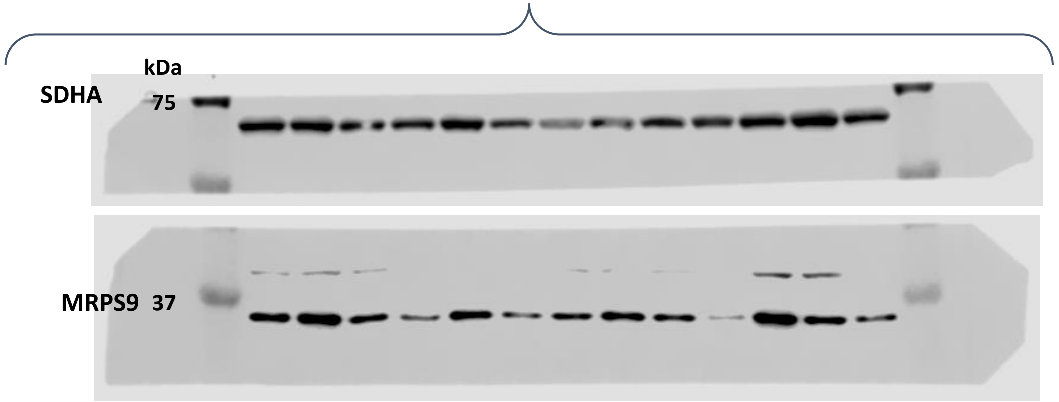

Selected area for publication

Liver mitochondria

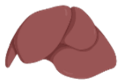

♂ Males

Western blot – SDS-PAGE Males

10 weeks old mice
ssAAV9-ApoE-hAAT-intron-GFM1

31/01/24

mbA

Genotype	WT	KI/KO	KI/KO	WT	KI/KO	KI/KO	WT	KI/KO	KI/KO	WT	KI/KO	KI/KO	WT
Treatment		V	AAV		V	AAV		V	AAV		V	AAV	

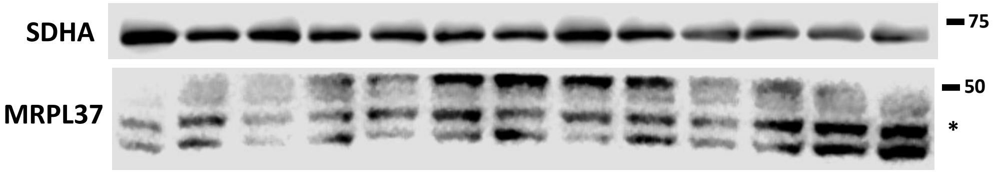

mbB

Genotype	WT	KI/KO	KI/KO	WT	KI/KO	KI/KO	WT	KI/KO	KI/KO	WT	KI/KO	KI/KO	WT
Treatment		V	AAV		V	AAV		V	AAV		V	AAV	

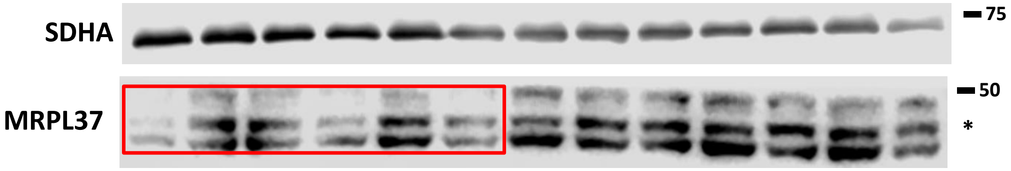

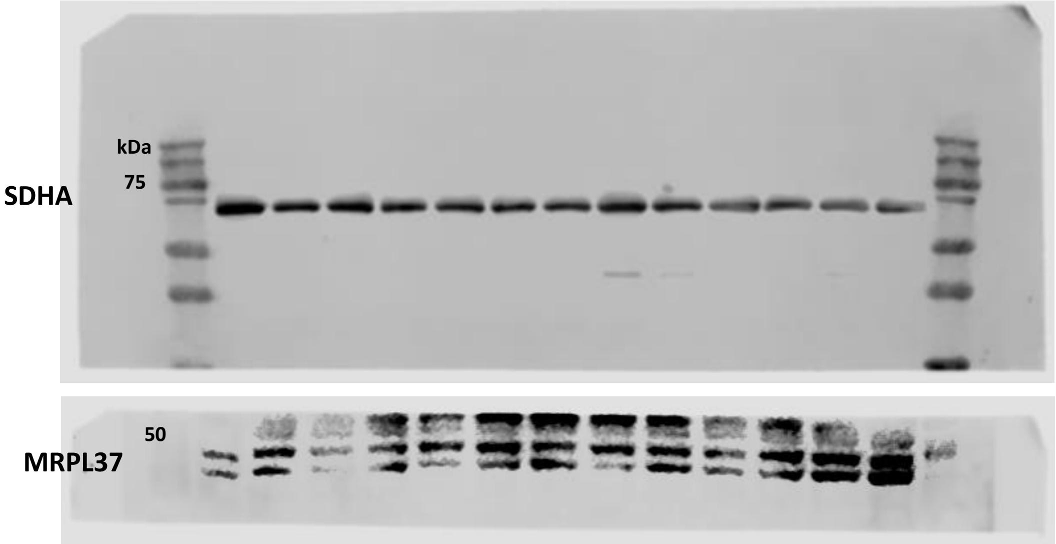

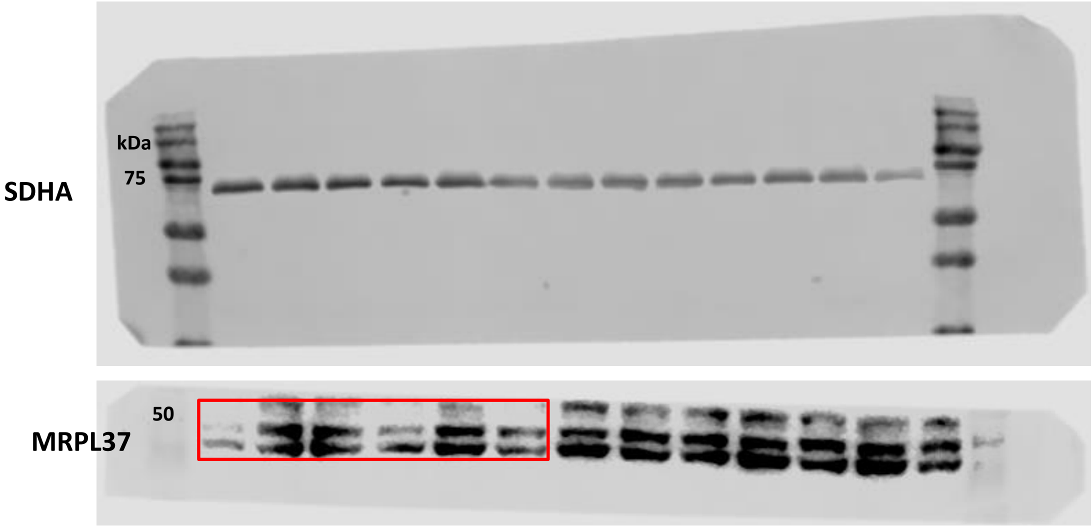

Selected area for publication
