# Supplementary material for: Systemic delivery of AAV-GFM1 corrects COXPD1 molecular alterations in Gfm1R671C/− mice
Source: EMBO Mol Med. 2026 Apr 17;18(6):2152–79. doi: 10.1038/s44321-026-00426-4 (PMC13269562; doi:10.1038/s44321-026-00426-4)
Supplement: Supplementary file 7 — Source data Fig. 6 [file 44321_2026_426_MOESM7_ESM.zip › Figure 6 updated/6B/Fig6B - MRPs mt ce Males and Females .pdf]

Brain mitochondria

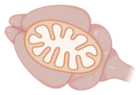

Females ♀

Western blot – SDS-PAGE

10 weeks old mice
ssAAV9P31-hSyn-GFM1

09/04/24

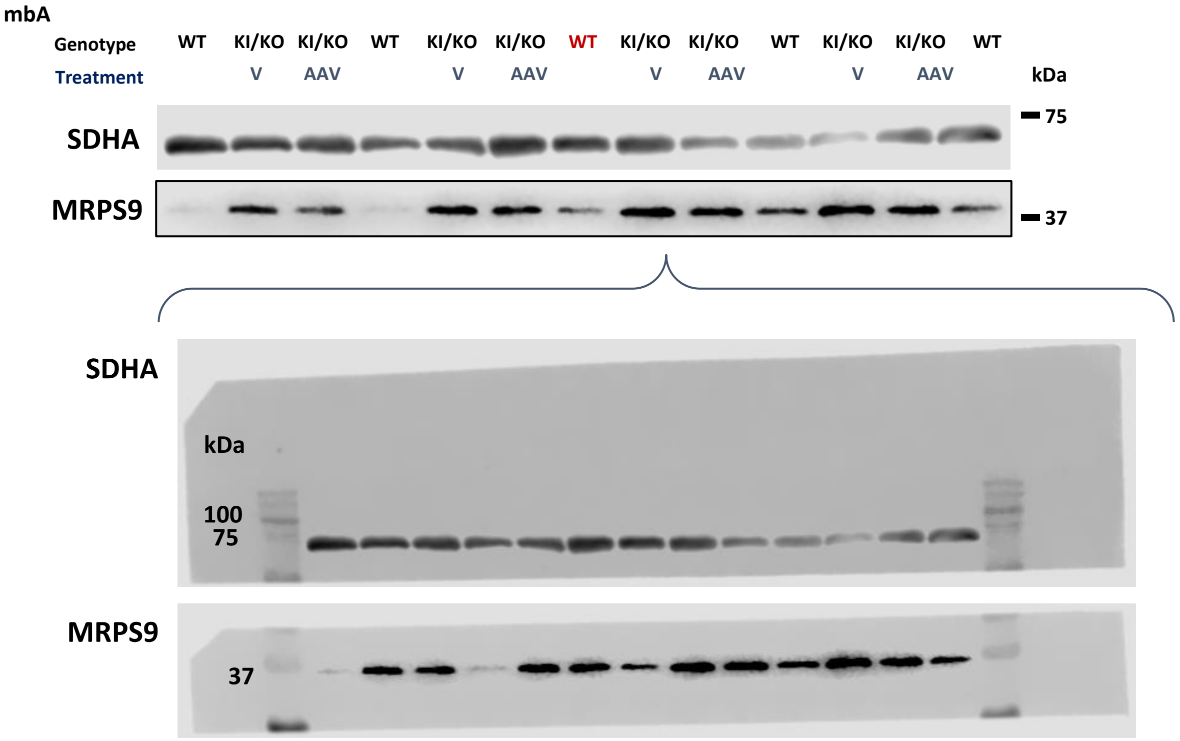

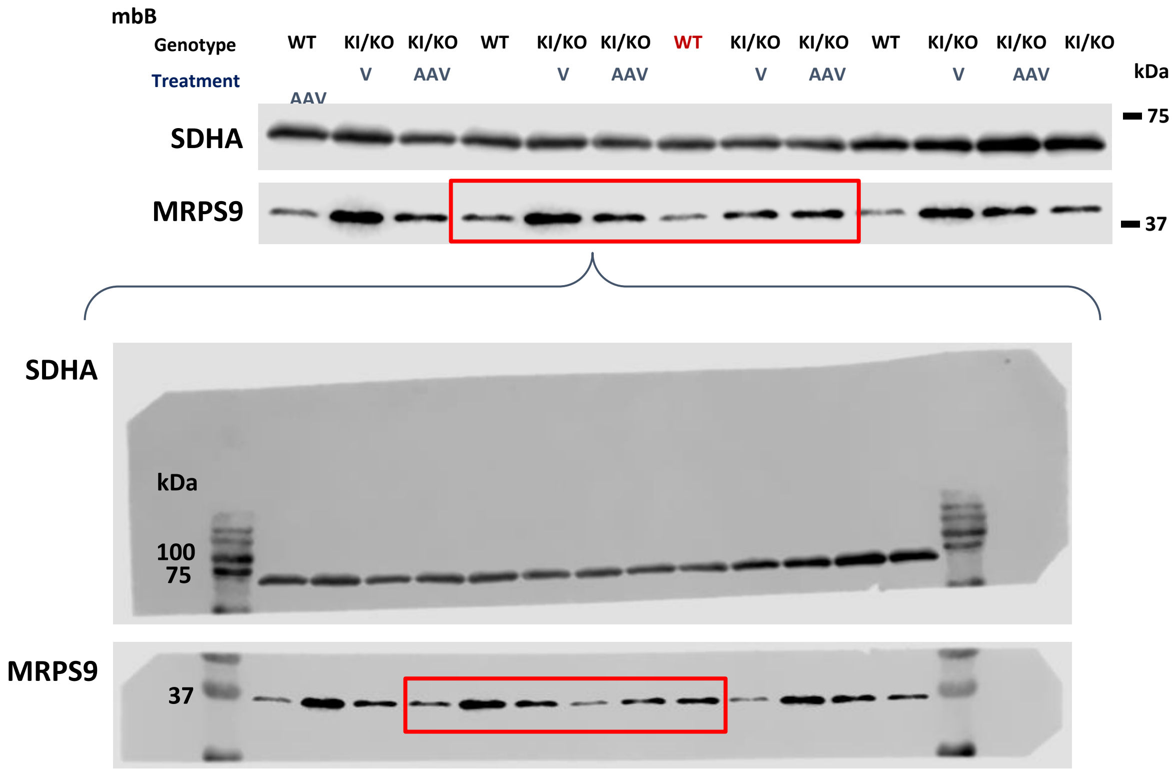

Selected area for publication

Brain mitochondria

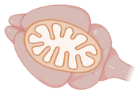

Females♀

Western blot – SDS-PAGE

10 weeks old mice
ssAAV9P31-hSyn-GFM1

18/04/24

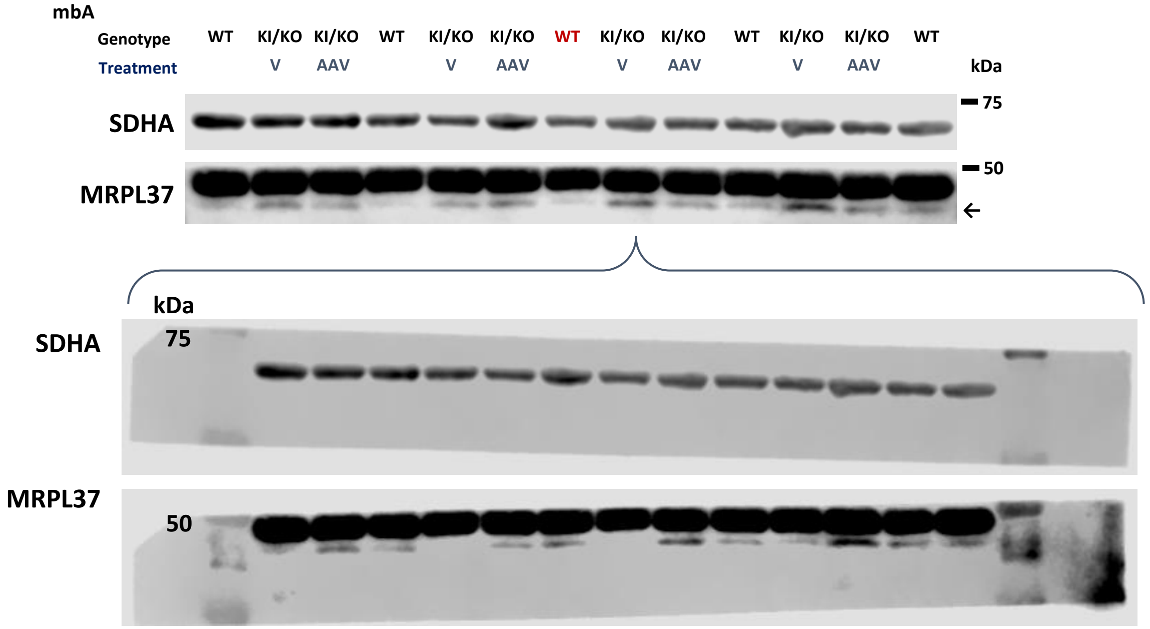

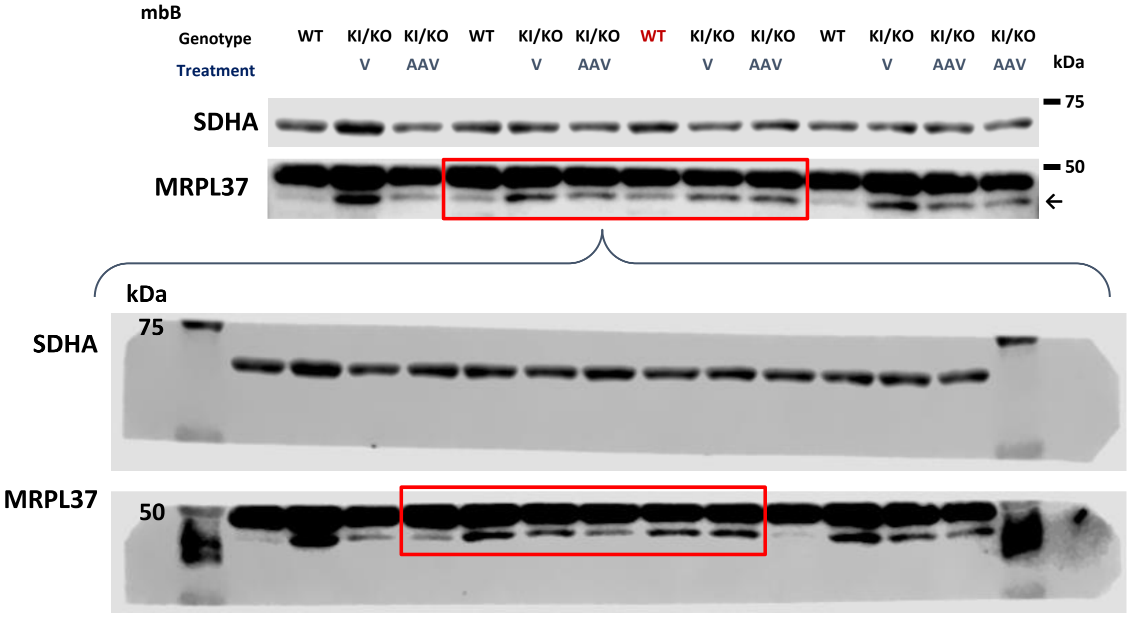

Selected area for publication

Brain mitochondria

Females ♀

Western blot – SDS-PAGE

10 weeks old mice
ssAAV9P31-hSyn-GFM1

27/03/24

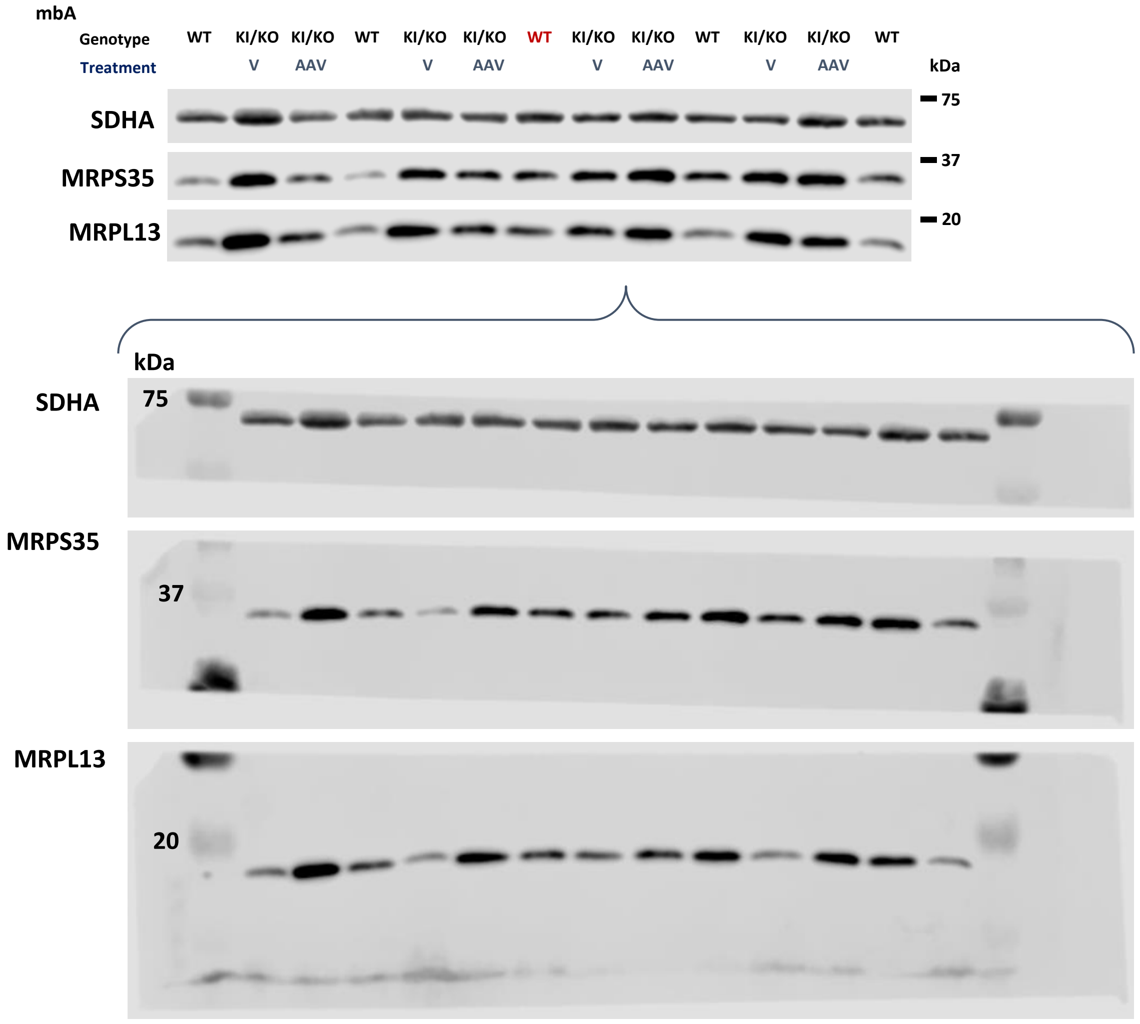

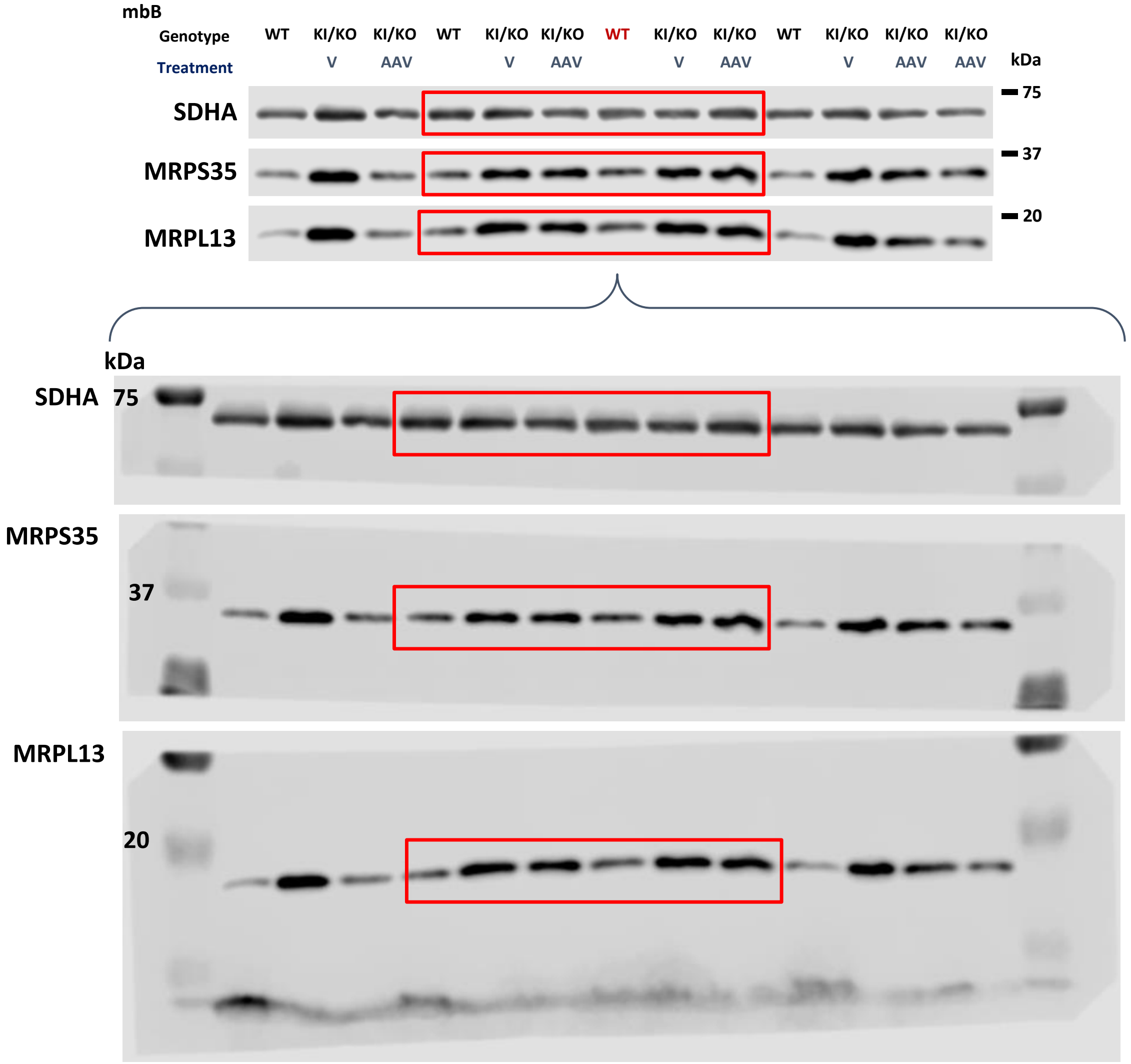

Selected area for publication

Brain mitochondria

Males ♂

Western blot – SDS-PAGE

10 weeks old mice
ssAAV9P31-hSyn-GFM1

10/04/24

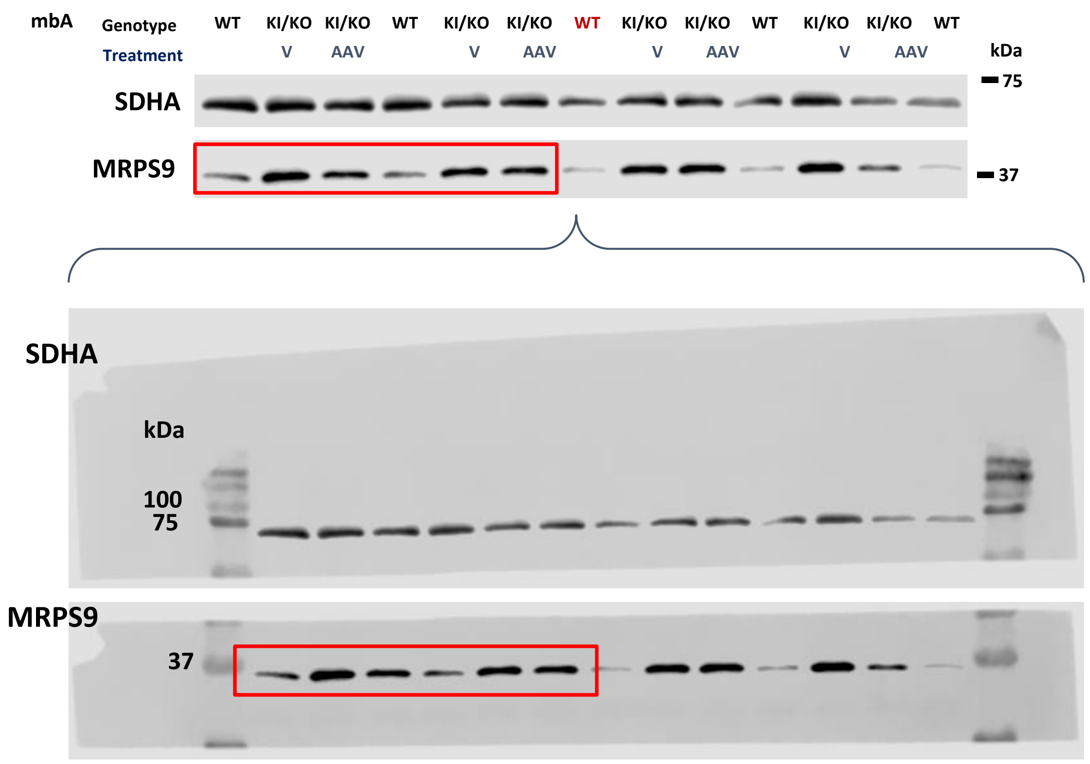

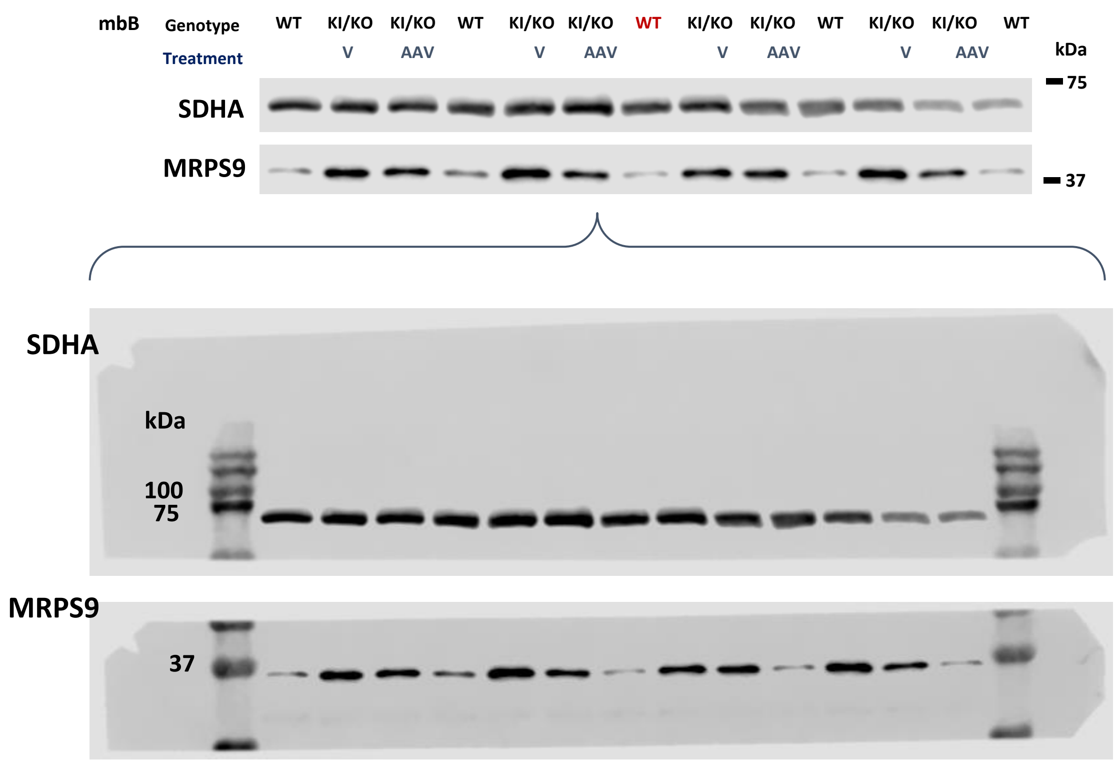

Selected area for publication

Brain mitochondria

Males ♂

Western blot – SDS-PAGE

10 weeks old mice
ssAAV9P31-hSyn-GFM1

02/04/24

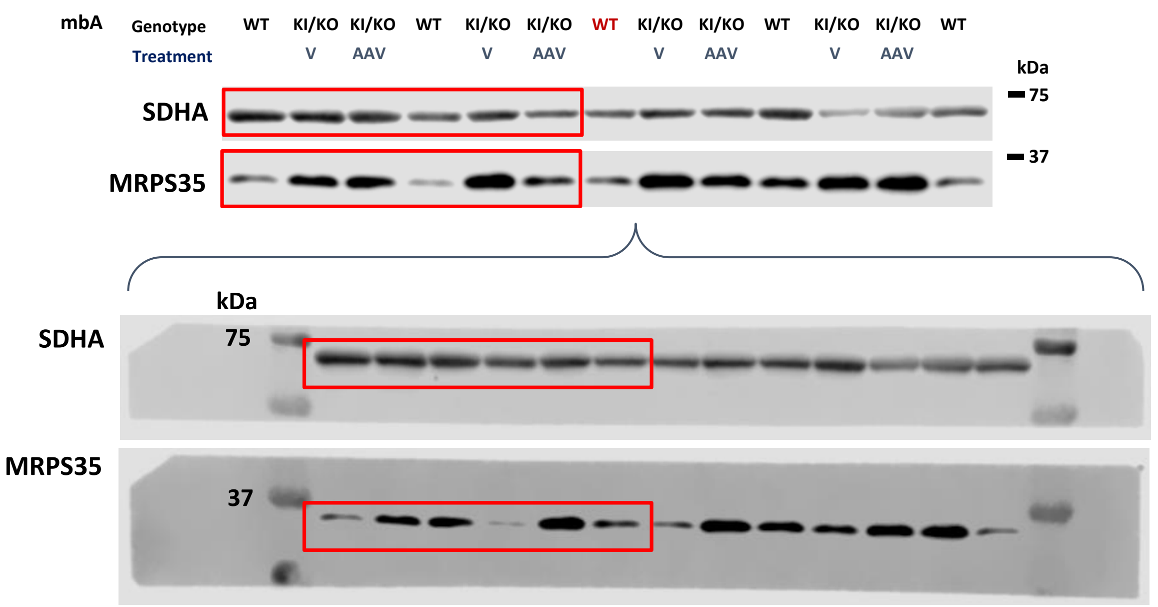

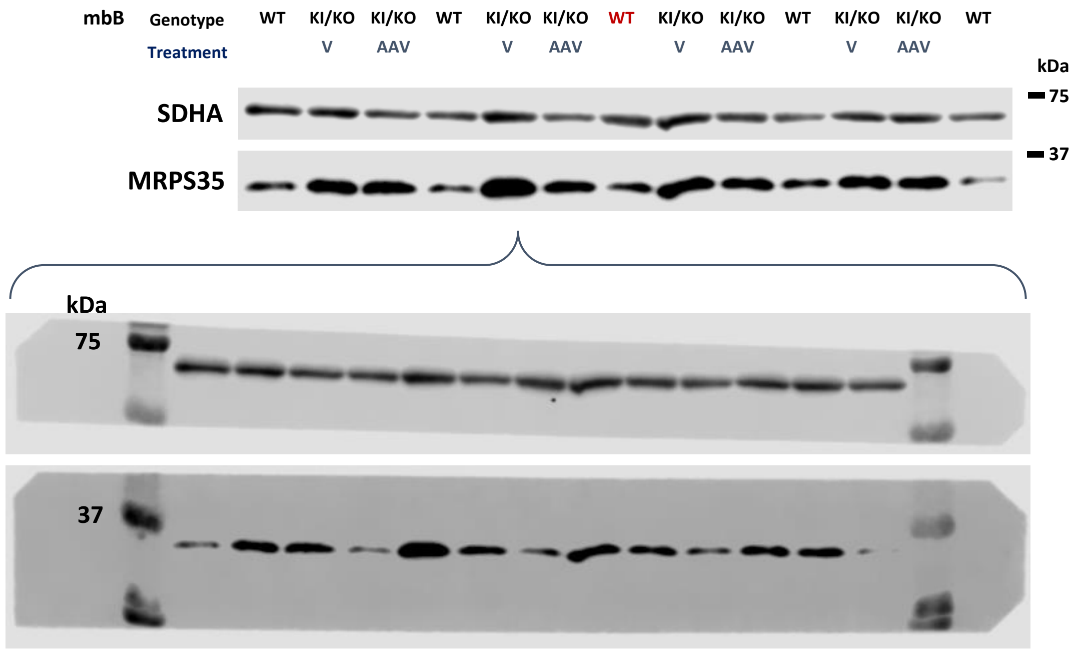

Selected area for publication

Brain mitochondria

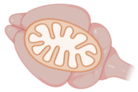

Males ♂

Western blot – SDS-PAGE

10 weeks old mice
ssAAV9P31-hSyn-GFM1

24/04/24

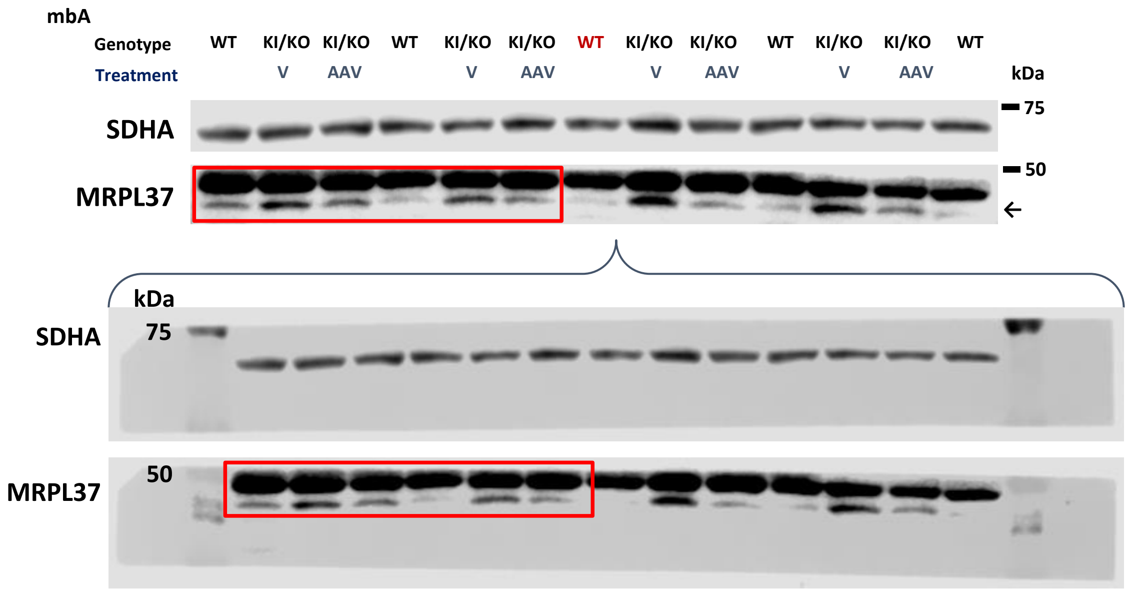

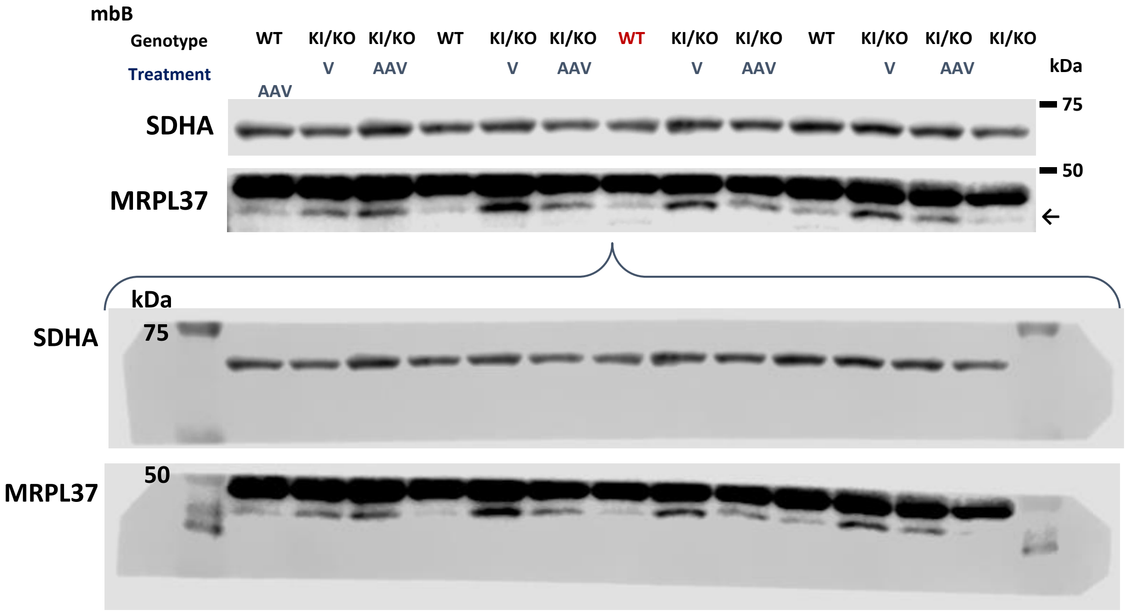

Selected area for publication

Brain mitochondria

Males ♂

Western blot – SDS-PAGE

10 weeks old mice
ssAAV9P31-hSyn-GFM1

22/02/24

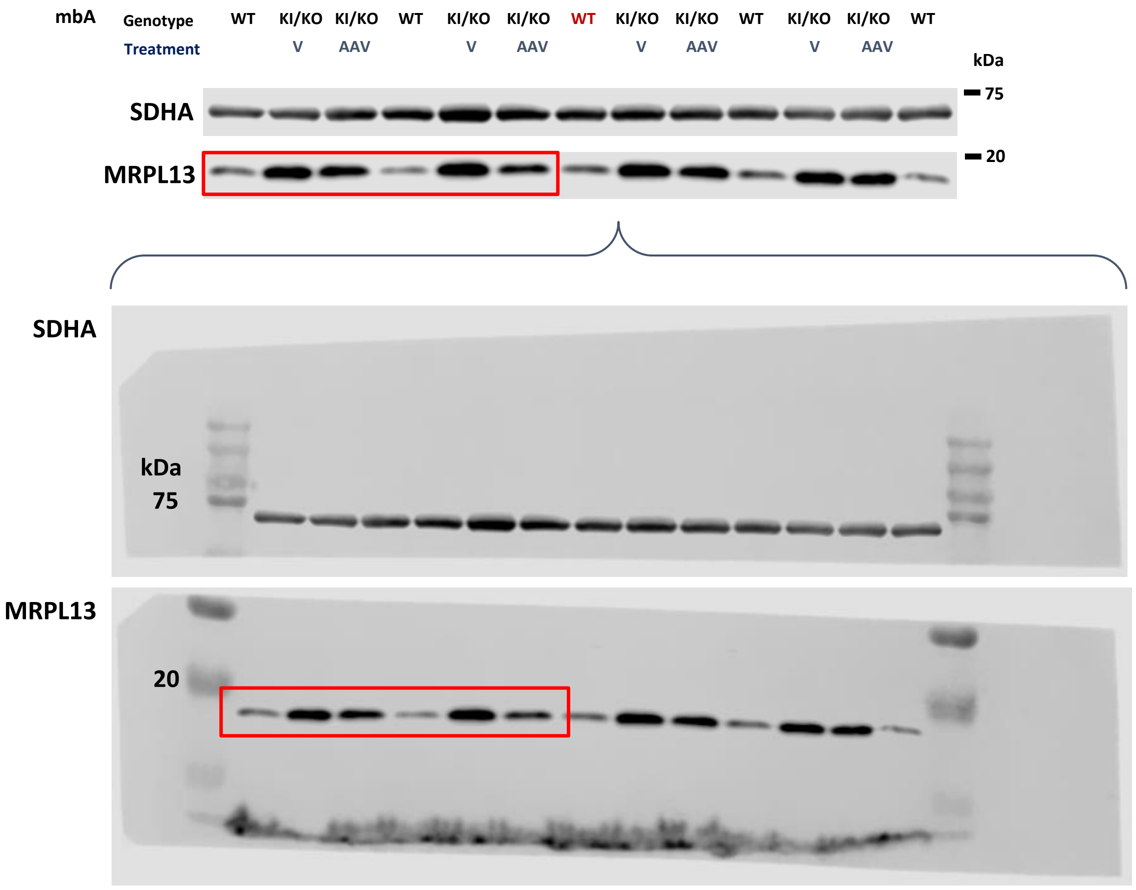

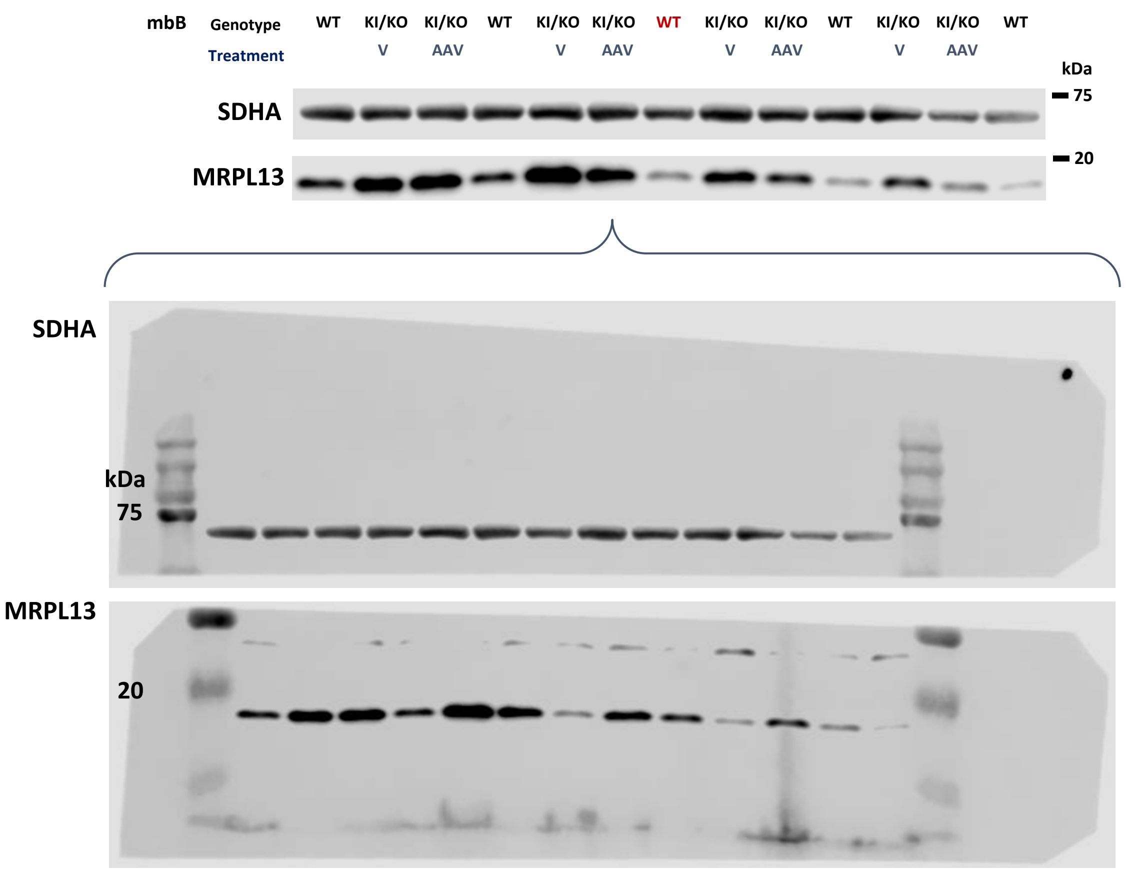

Selected area for publication
