# Supplementary material for: Systemic delivery of AAV-GFM1 corrects COXPD1 molecular alterations in Gfm1R671C/− mice
Source: EMBO Mol Med. 2026 Apr 17;18(6):2152–79. doi: 10.1038/s44321-026-00426-4 (PMC13269562; doi:10.1038/s44321-026-00426-4)

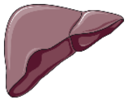

Mitochondria lysates from liver
30 weeks old mice

Blue Native - PAGE

Selected area for publication

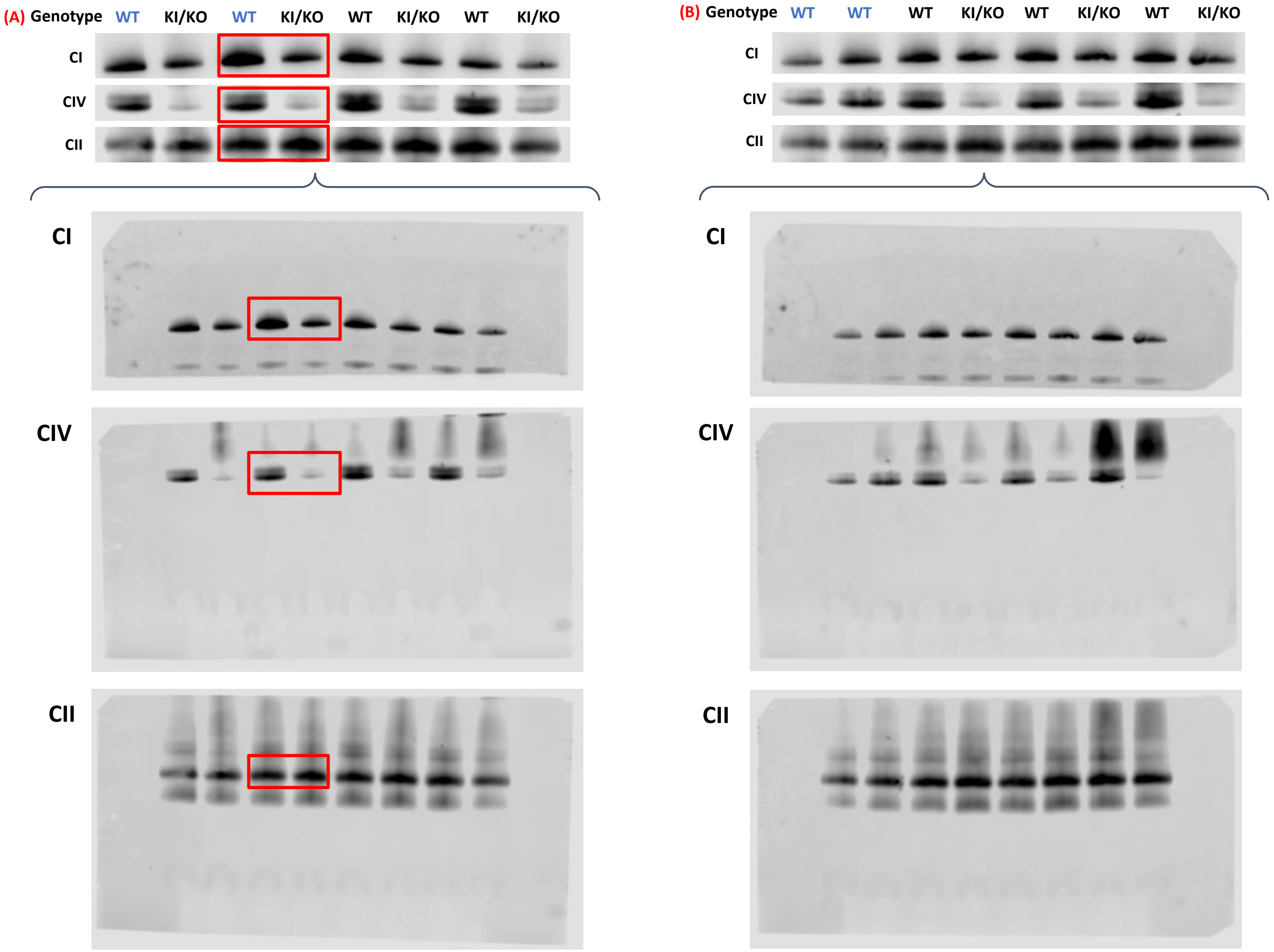

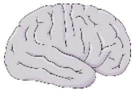

Mitochondria lysates from brain
30 weeks old mice

Blue Native - PAGE

Selected area for publication

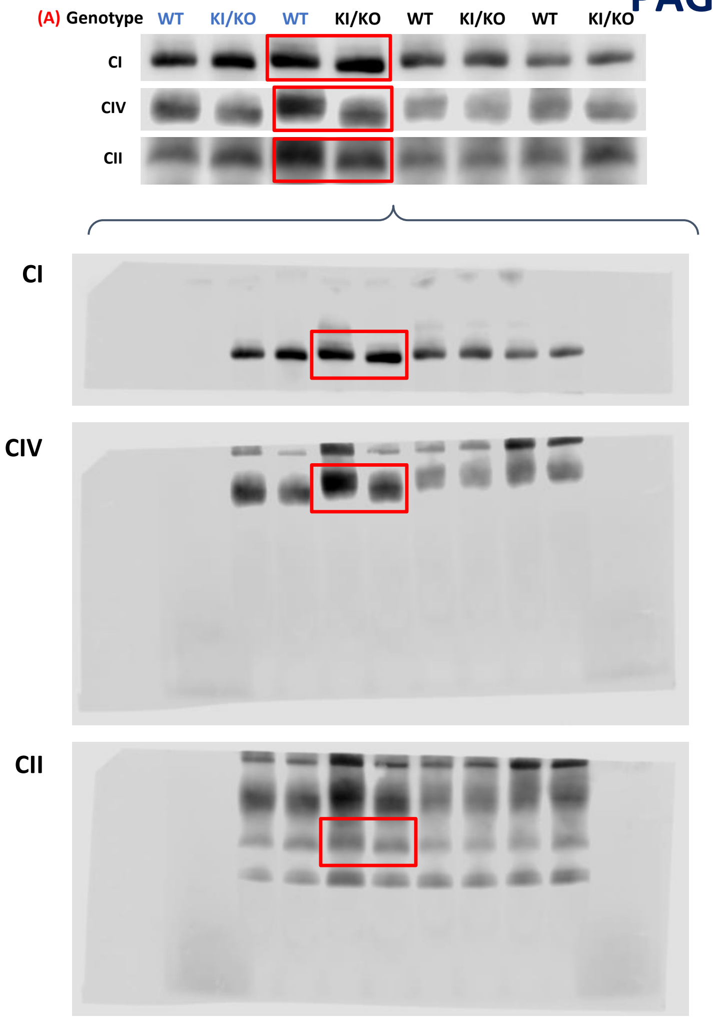

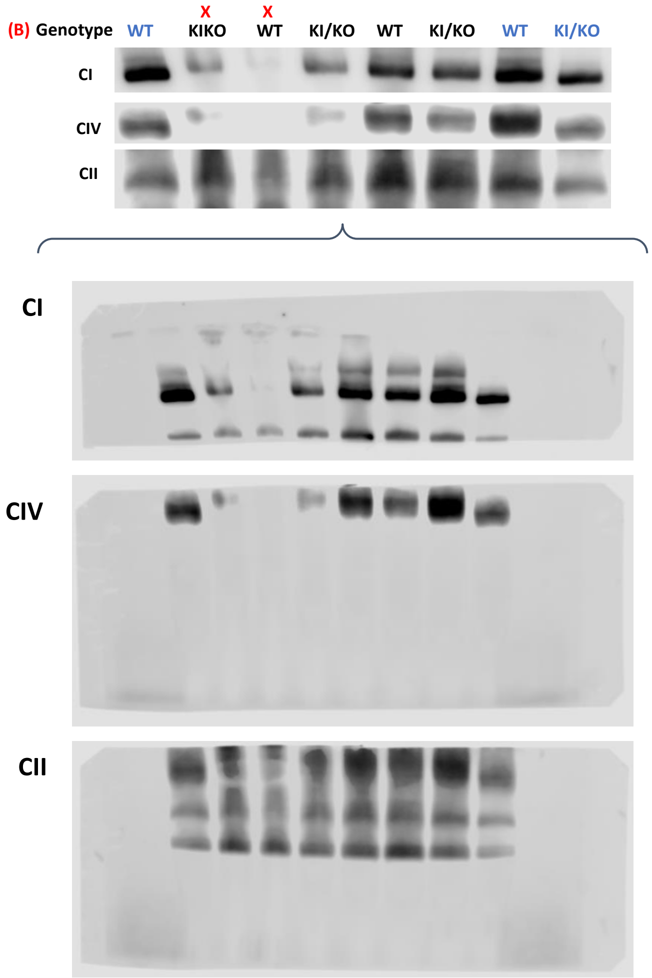

Supplement: Supplementary file 9 — Figure EV1 Source Data [file 44321_2026_426_MOESM9_ESM.zip › EV1 updated/EV1D/EV1D - BN-PAGE 30w liver and brain.pdf]
