# Supplementary figures and images for: Systemic delivery of AAV-GFM1 corrects COXPD1 molecular alterations in Gfm1R671C/− mice (part 2 of 2)
Source: EMBO Mol Med. 2026 Apr 17;18(6):2152–79. doi: 10.1038/s44321-026-00426-4 (PMC13269562; doi:10.1038/s44321-026-00426-4)

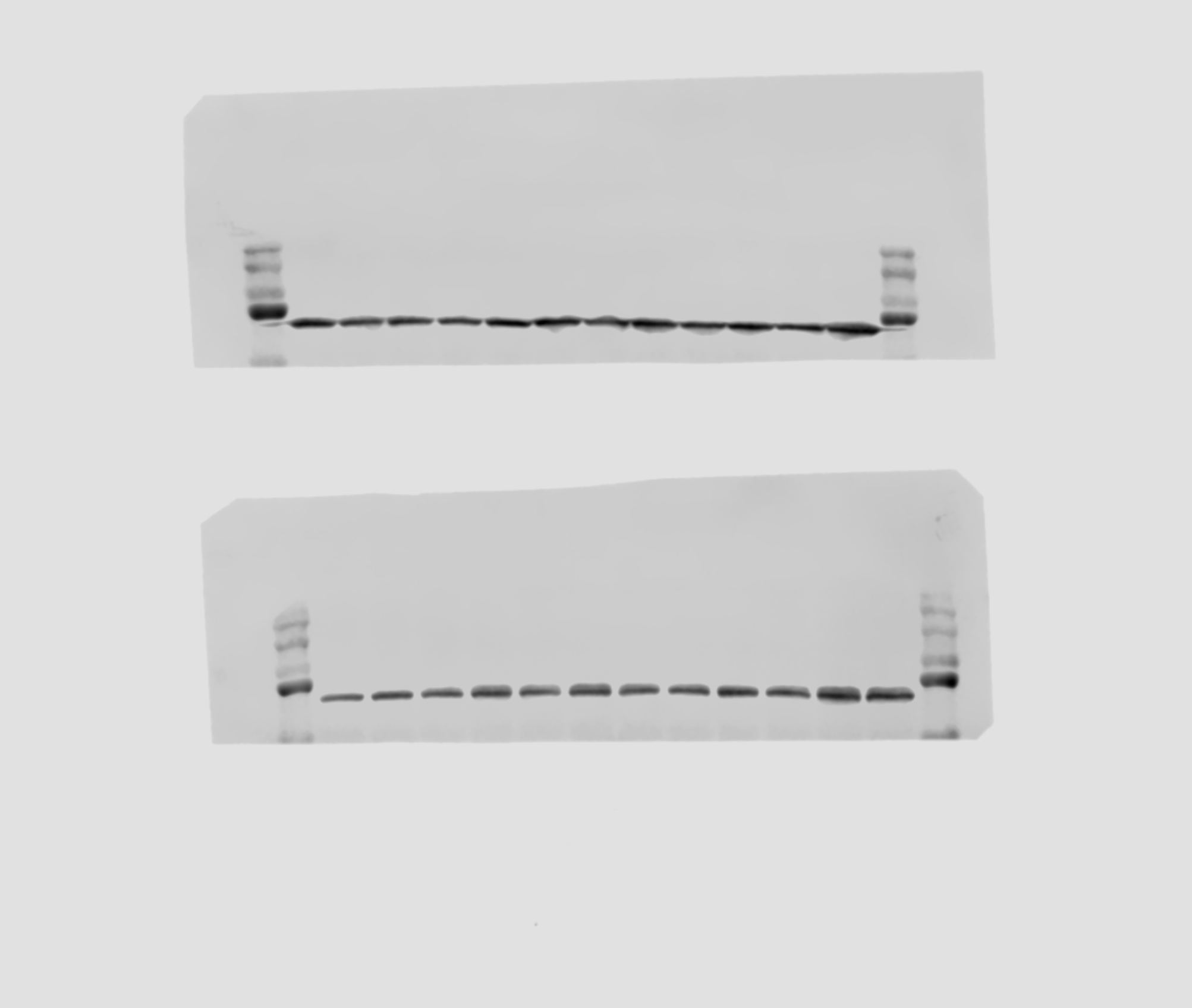

Supplement: Supplementary file 9 — Figure EV1 Source Data [file 44321_2026_426_MOESM9_ESM.zip › EV1 updated/EV1C/EV1C Brain SDHA a b.tif]

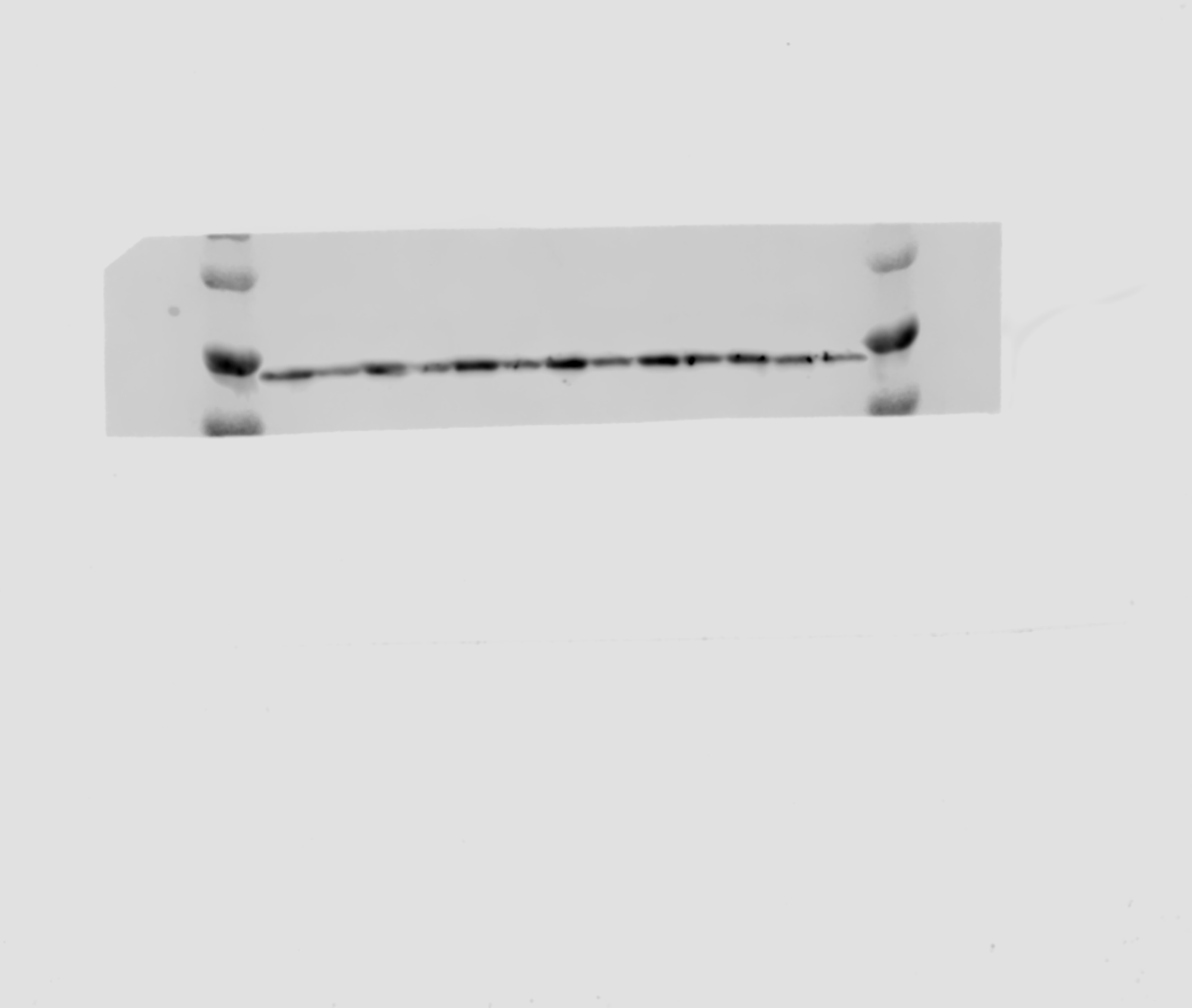

Supplement: Supplementary file 9 — Figure EV1 Source Data [file 44321_2026_426_MOESM9_ESM.zip › EV1 updated/EV1C/EV1C Liver COX2.tif]

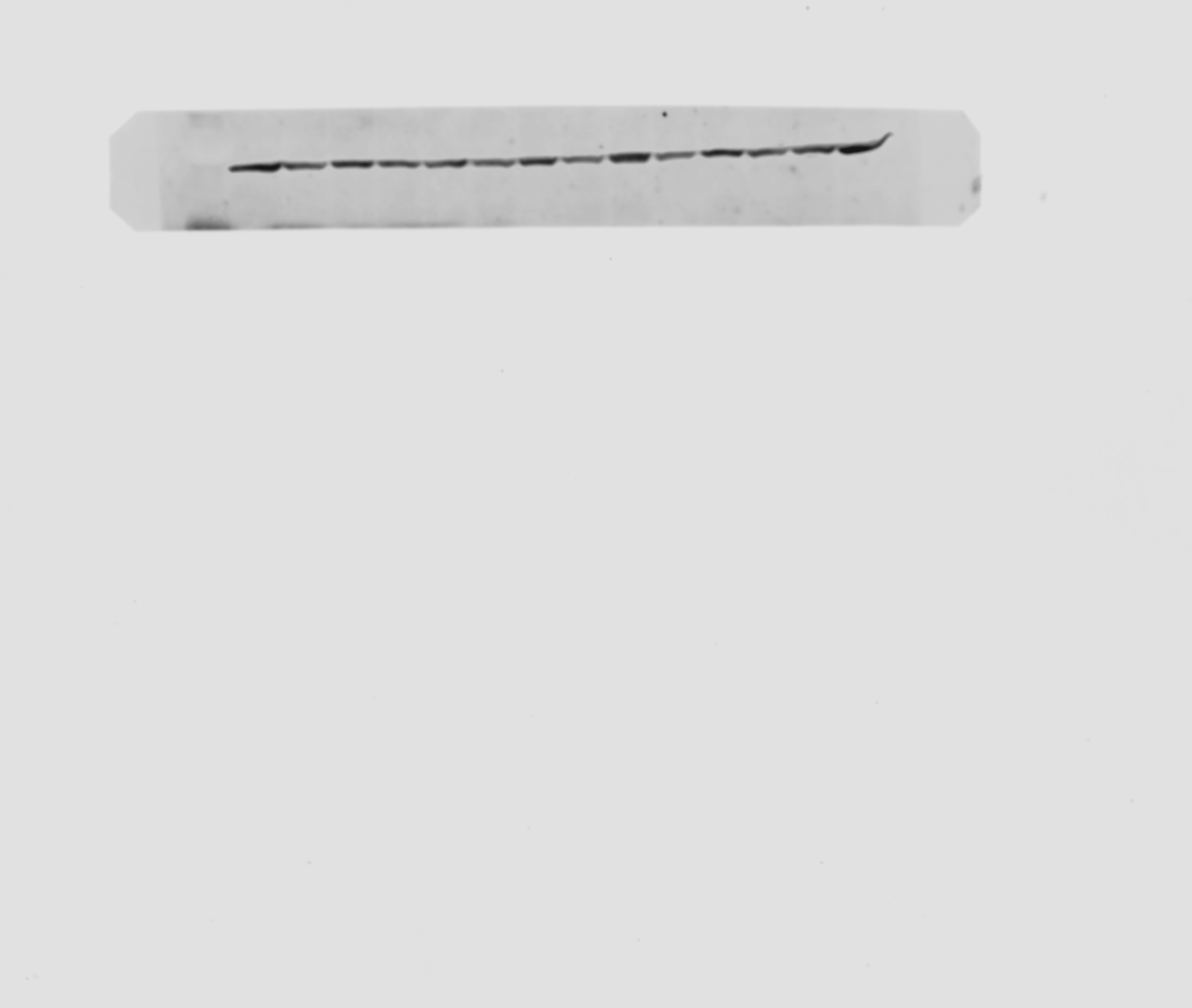

Supplement: Supplementary file 9 — Figure EV1 Source Data [file 44321_2026_426_MOESM9_ESM.zip › EV1 updated/EV1C/EV1C Liver NDUFA9.tif]

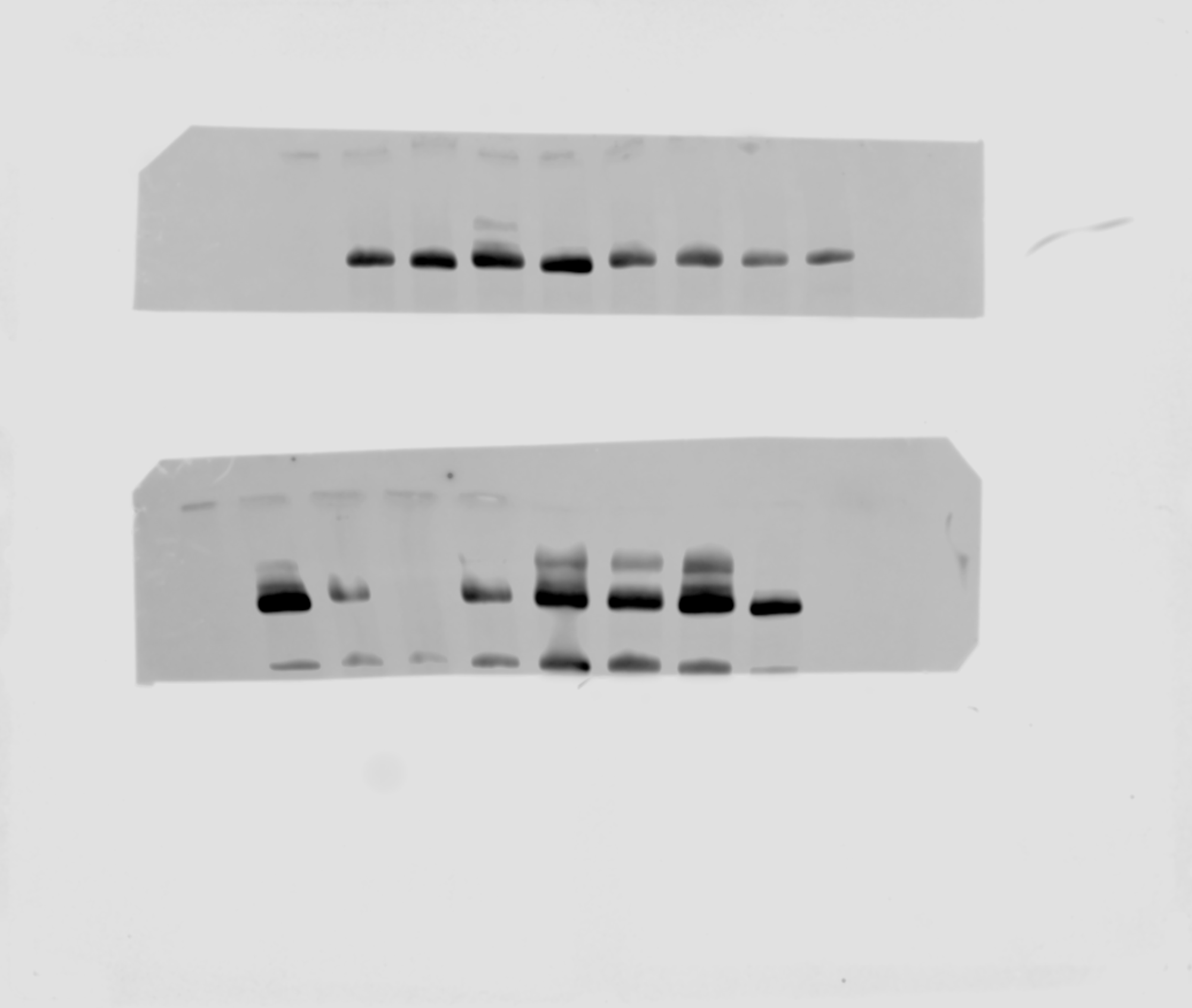

Supplement: Supplementary file 9 — Figure EV1 Source Data [file 44321_2026_426_MOESM9_ESM.zip › EV1 updated/EV1D/EV1D Brain CI a i b.tif]

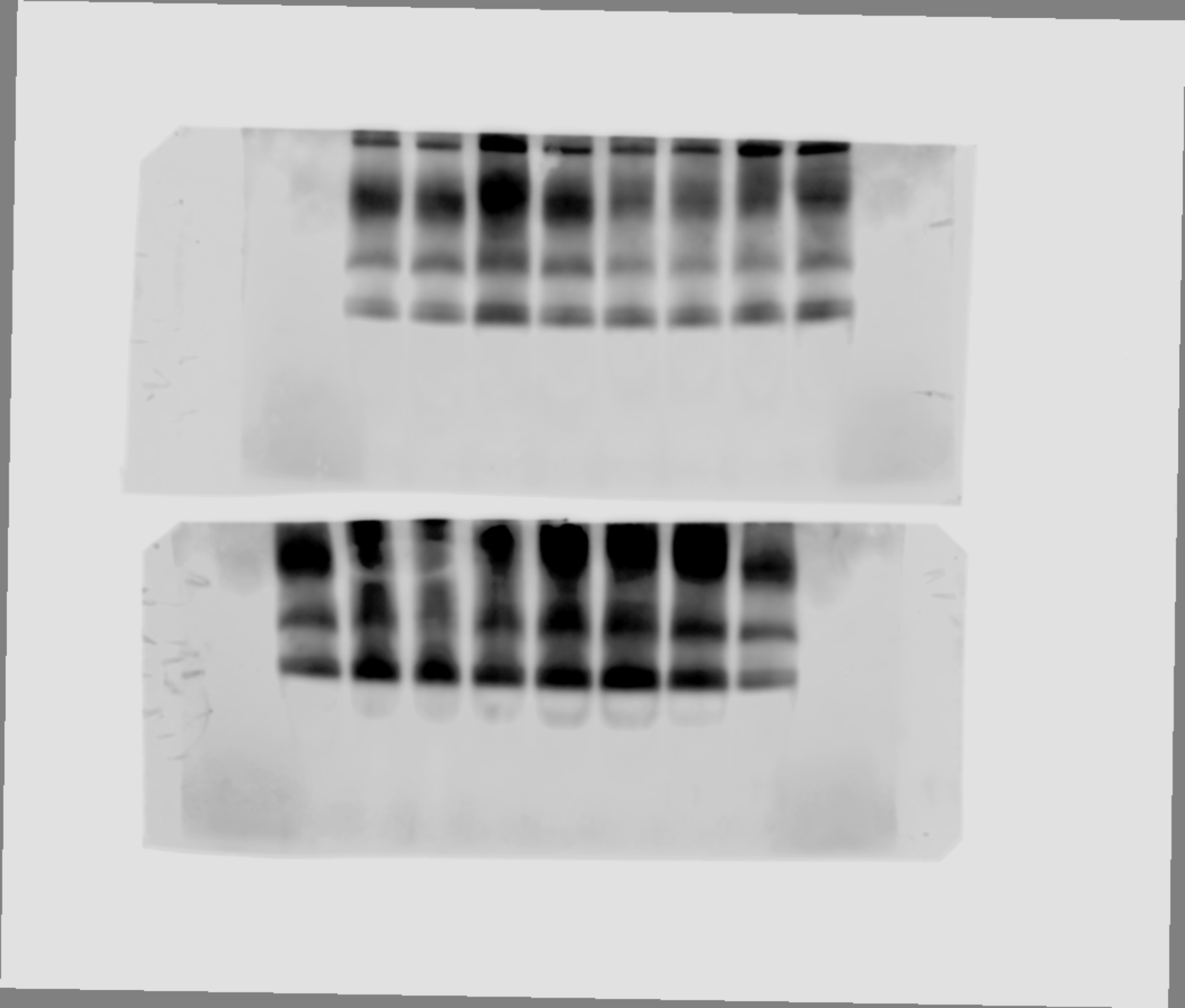

Supplement: Supplementary file 9 — Figure EV1 Source Data [file 44321_2026_426_MOESM9_ESM.zip › EV1 updated/EV1D/EV1D Brain CII a i b.tif]

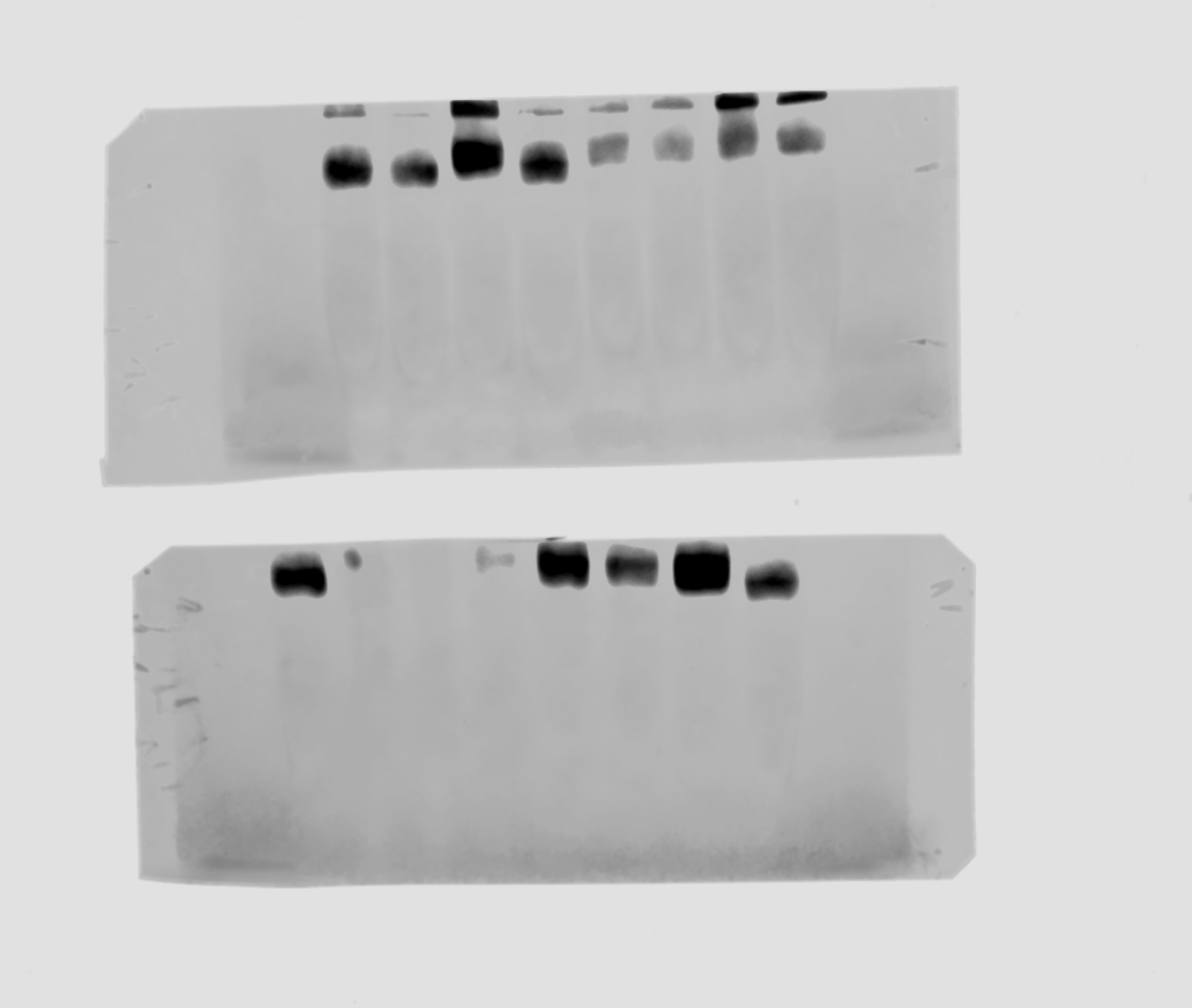

Supplement: Supplementary file 9 — Figure EV1 Source Data [file 44321_2026_426_MOESM9_ESM.zip › EV1 updated/EV1D/EV1D Brain CIV a i b.tif]

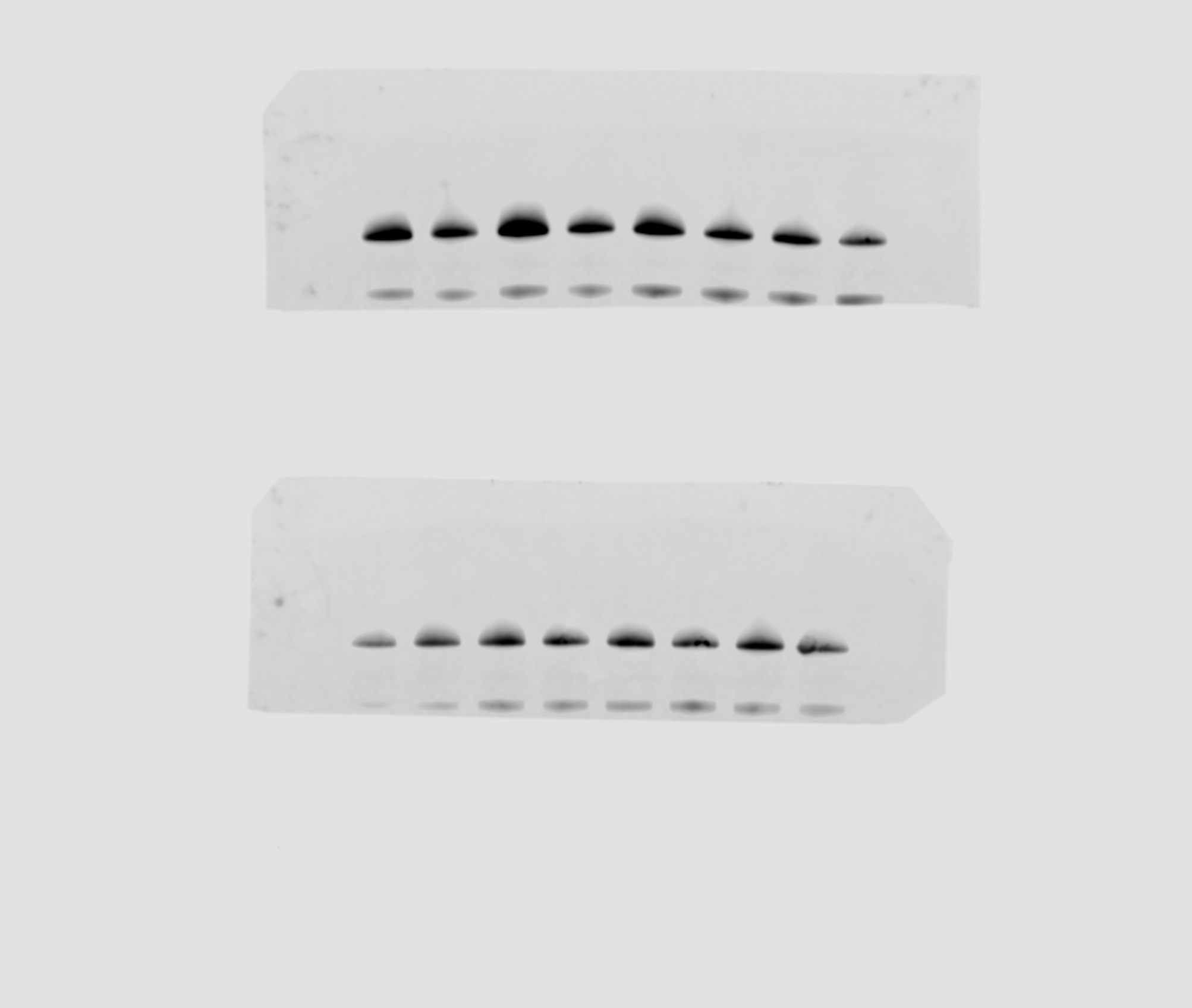

Supplement: Supplementary file 9 — Figure EV1 Source Data [file 44321_2026_426_MOESM9_ESM.zip › EV1 updated/EV1D/EV1D Liver CI a i b.tif]

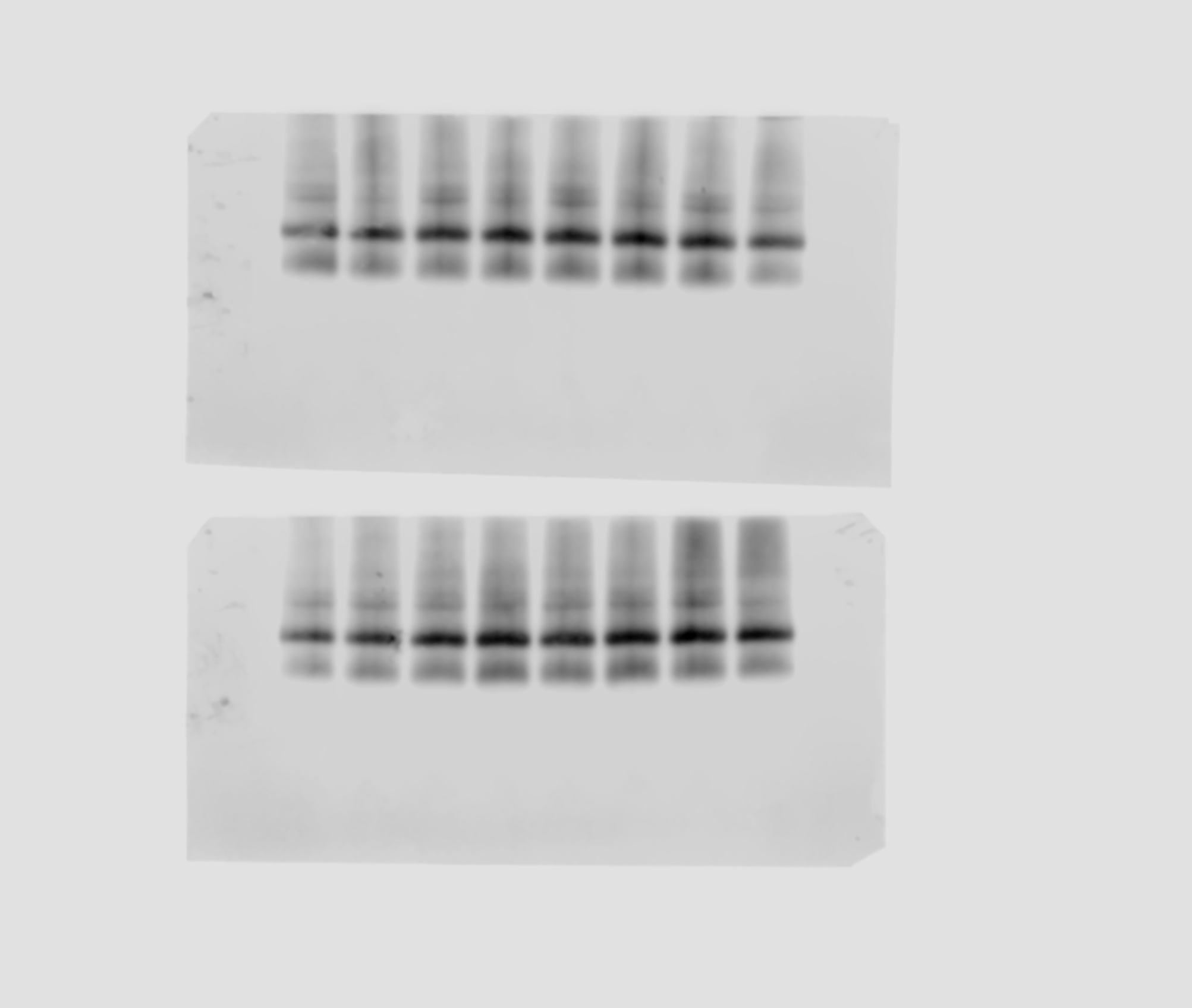

Supplement: Supplementary file 9 — Figure EV1 Source Data [file 44321_2026_426_MOESM9_ESM.zip › EV1 updated/EV1D/EV1D Liver CII a i b.tif]

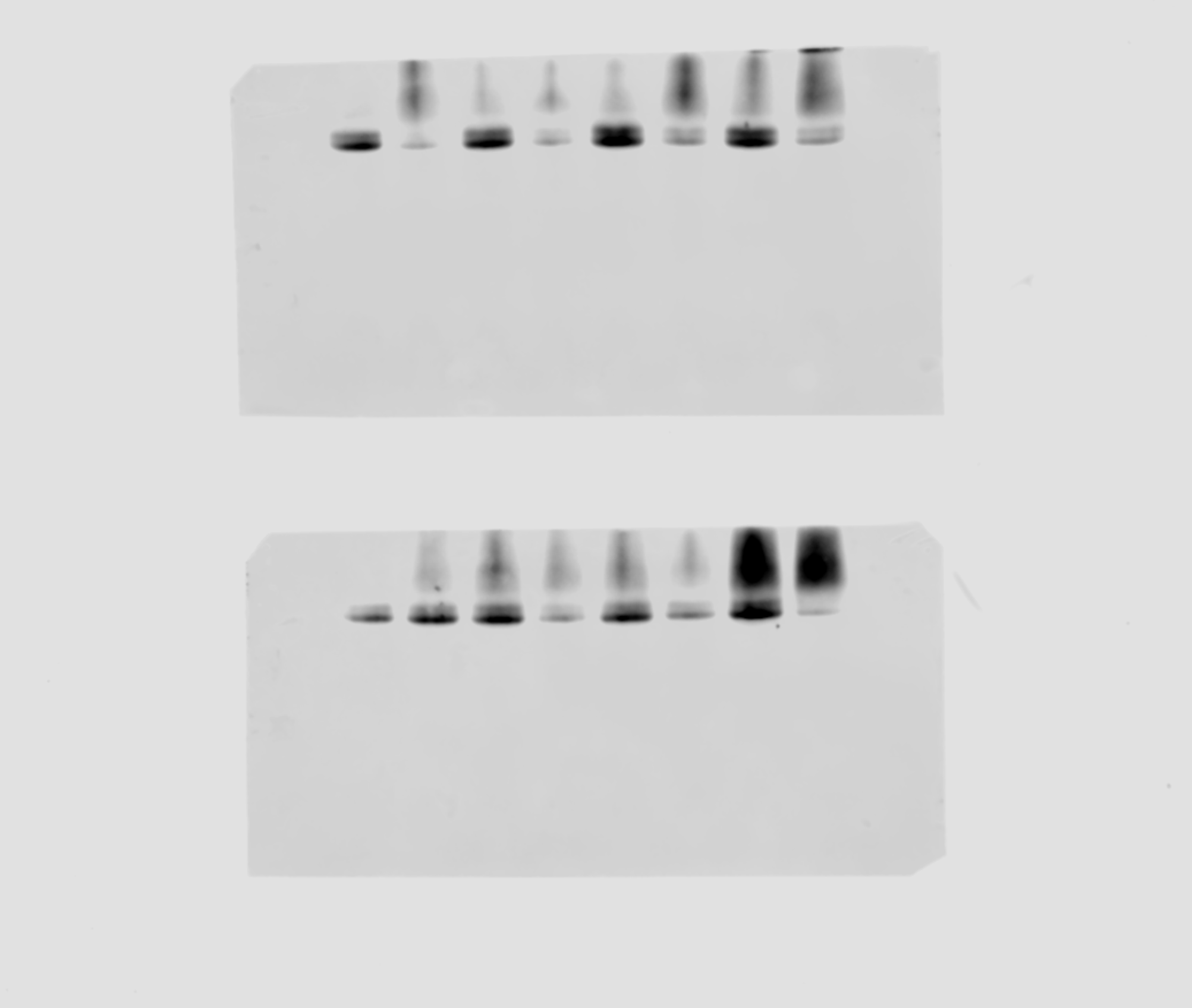

Supplement: Supplementary file 9 — Figure EV1 Source Data [file 44321_2026_426_MOESM9_ESM.zip › EV1 updated/EV1D/EV1D Liver CIV a i b.tif]

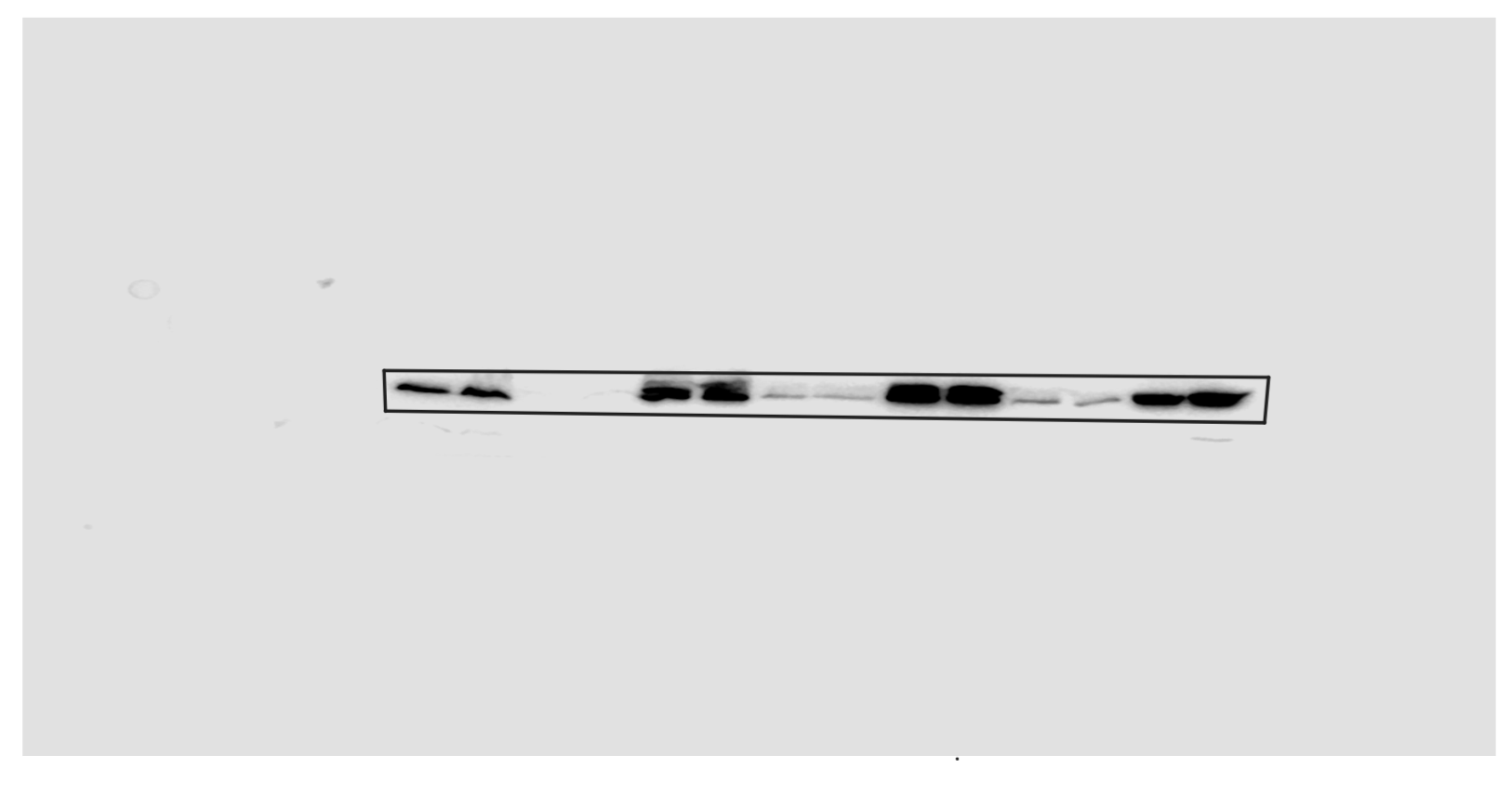

Supplement: Supplementary file 10 — Figure EV2 Source Data [file 44321_2026_426_MOESM10_ESM.zip › EV2 updated/EV2A/WB EFG1.png]

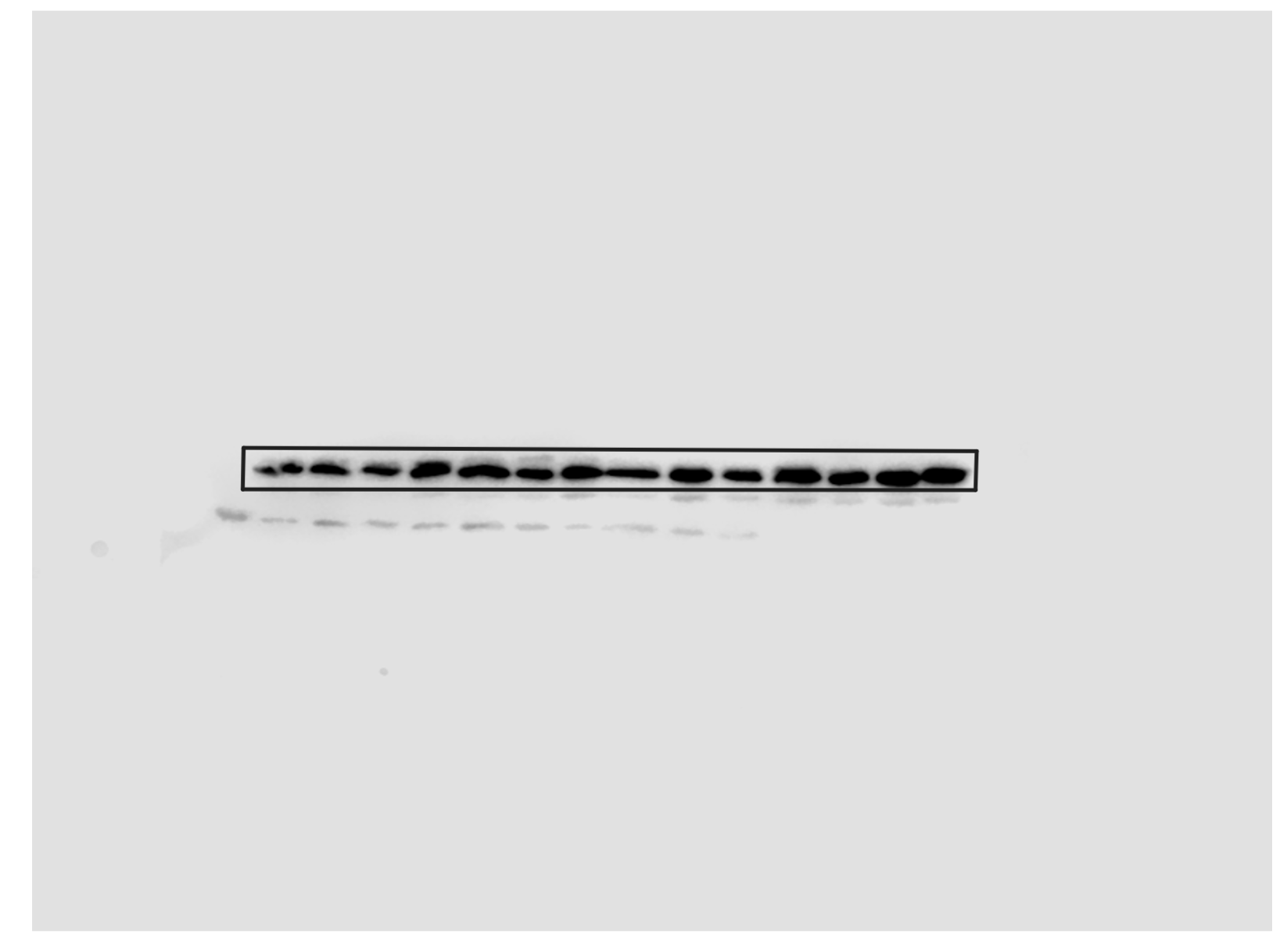

Supplement: Supplementary file 10 — Figure EV2 Source Data [file 44321_2026_426_MOESM10_ESM.zip › EV2 updated/EV2A/WB VDAC1.png]

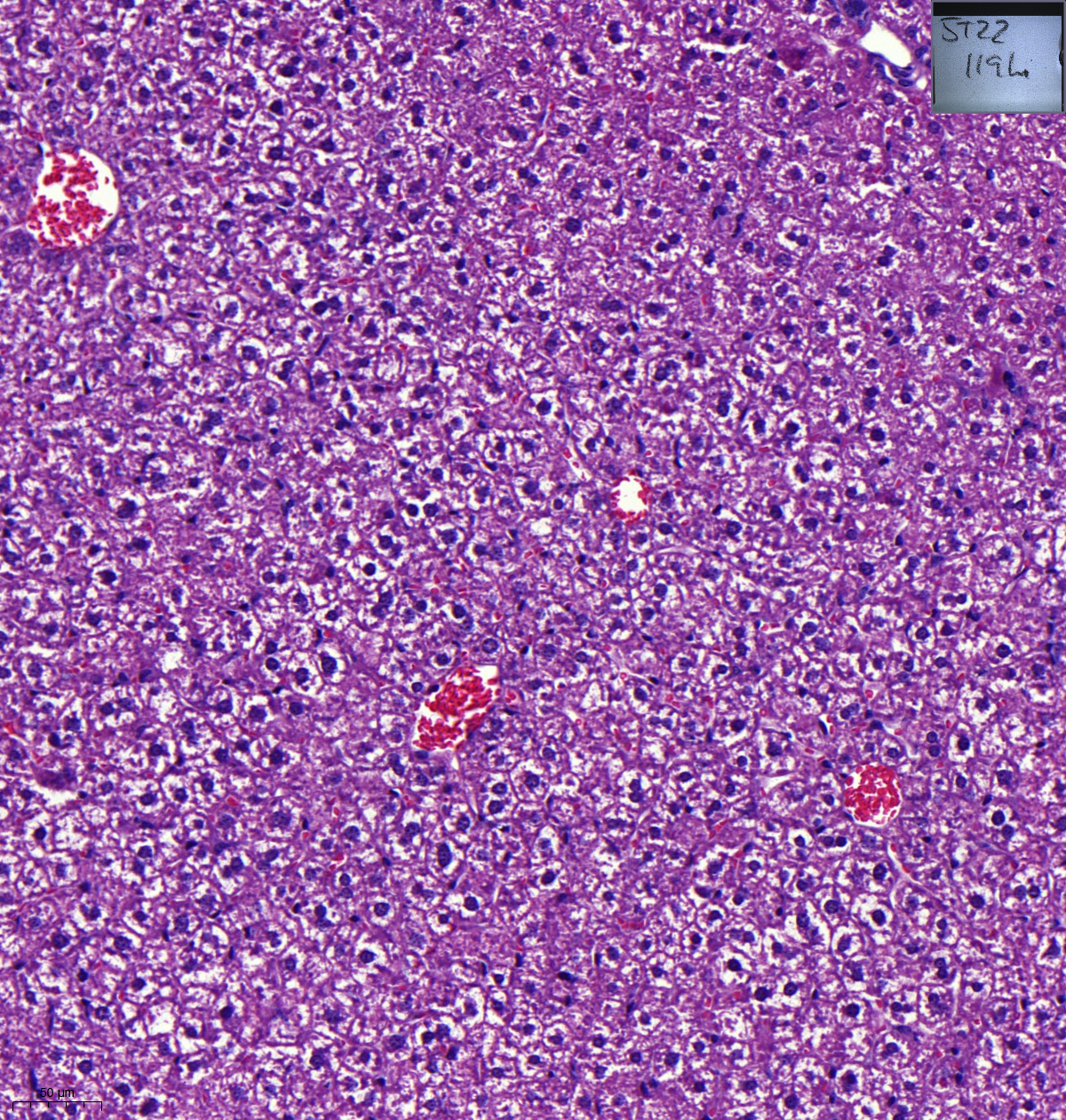

Supplement: Supplementary file 11 — Figure EV3 Source Data [file 44321_2026_426_MOESM11_ESM.zip › EV3 updated/Females/JT22-119hi Female KIKO AAV HE_20.0x.tif]

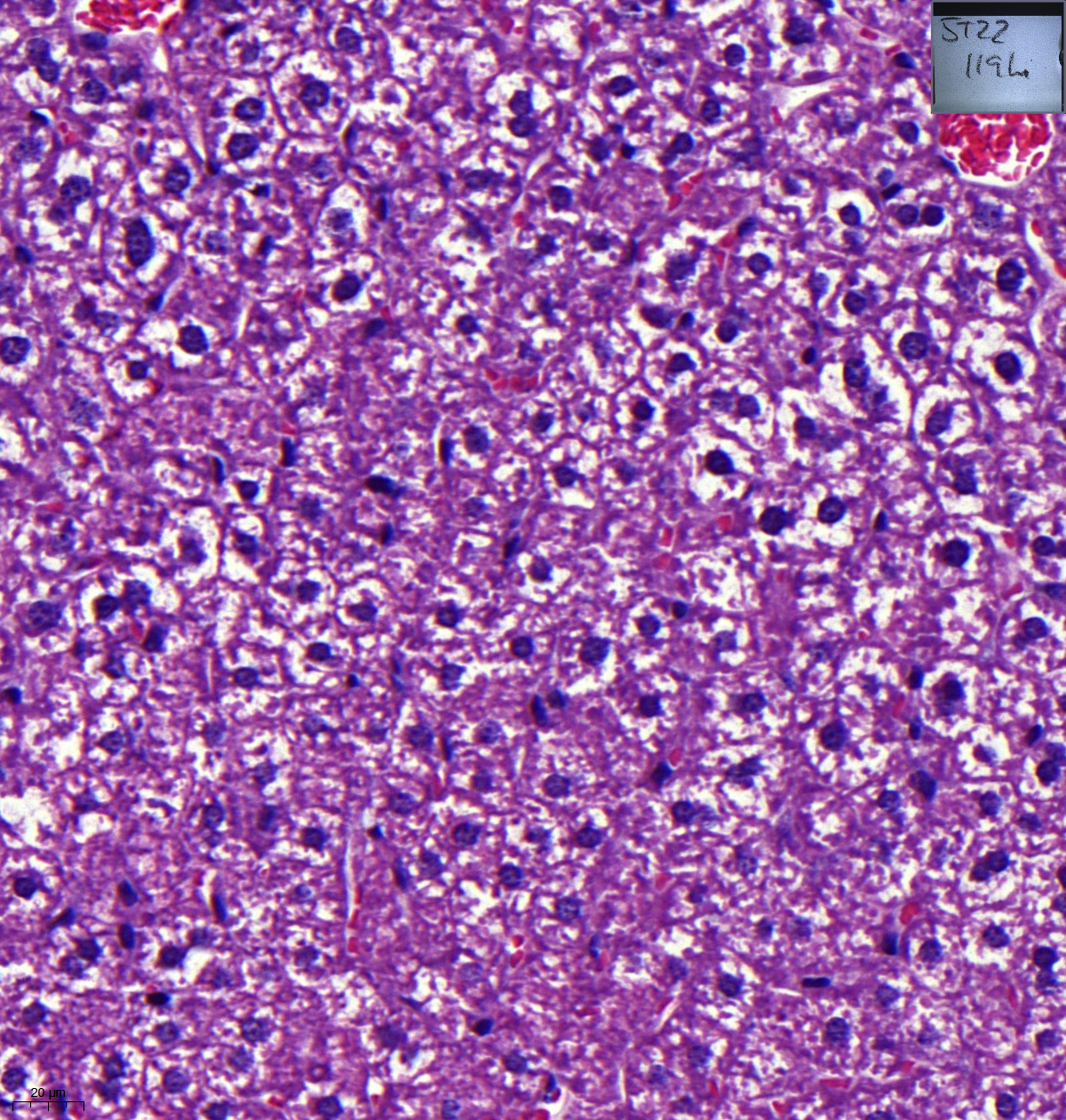

Supplement: Supplementary file 11 — Figure EV3 Source Data [file 44321_2026_426_MOESM11_ESM.zip › EV3 updated/Females/JT22-119hi Female KIKO AAV HE_40.0x.tif]

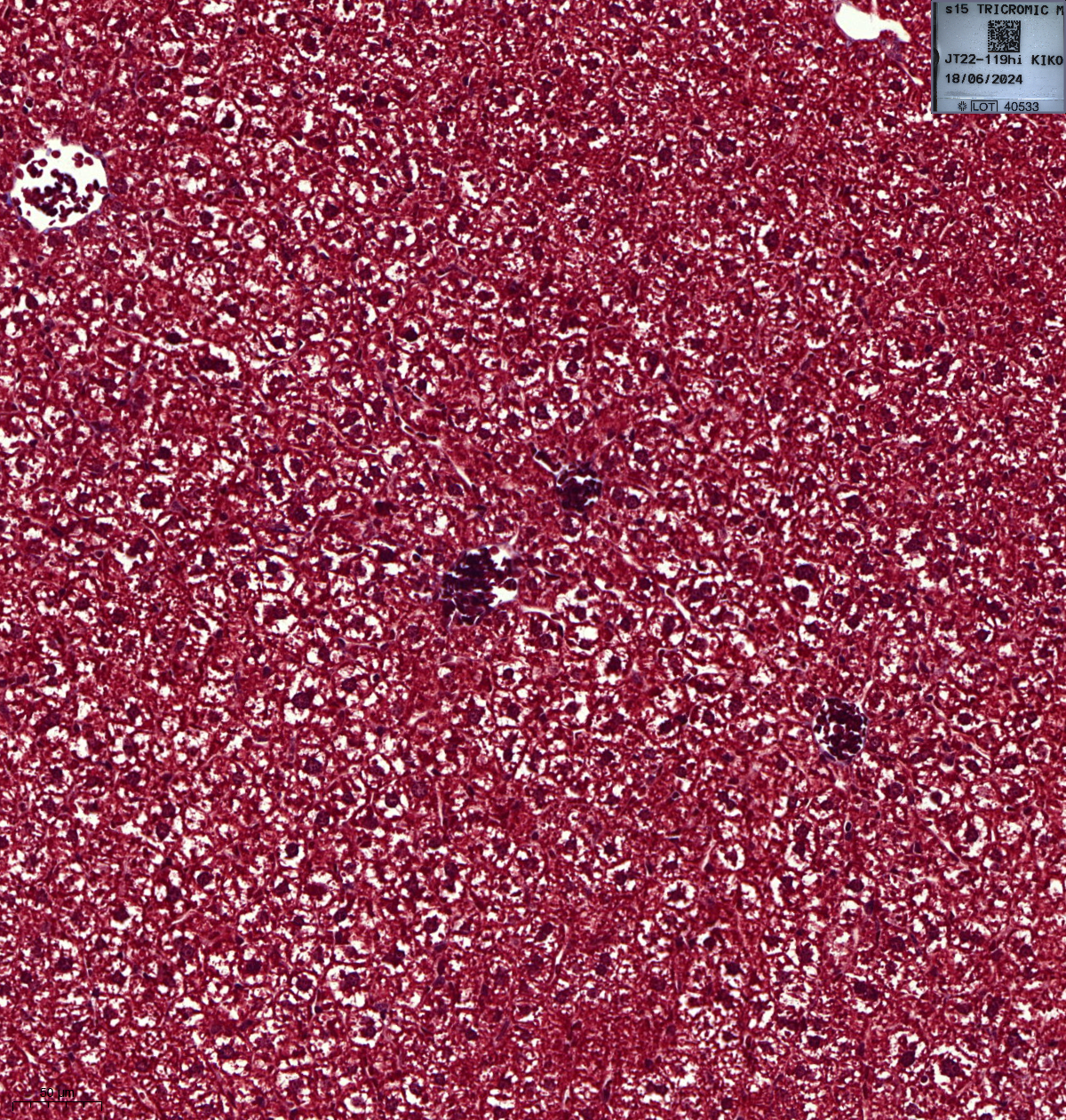

Supplement: Supplementary file 11 — Figure EV3 Source Data [file 44321_2026_426_MOESM11_ESM.zip › EV3 updated/Females/JT22-119hi Female KIKO AAV TRIC_20.0x.tif]

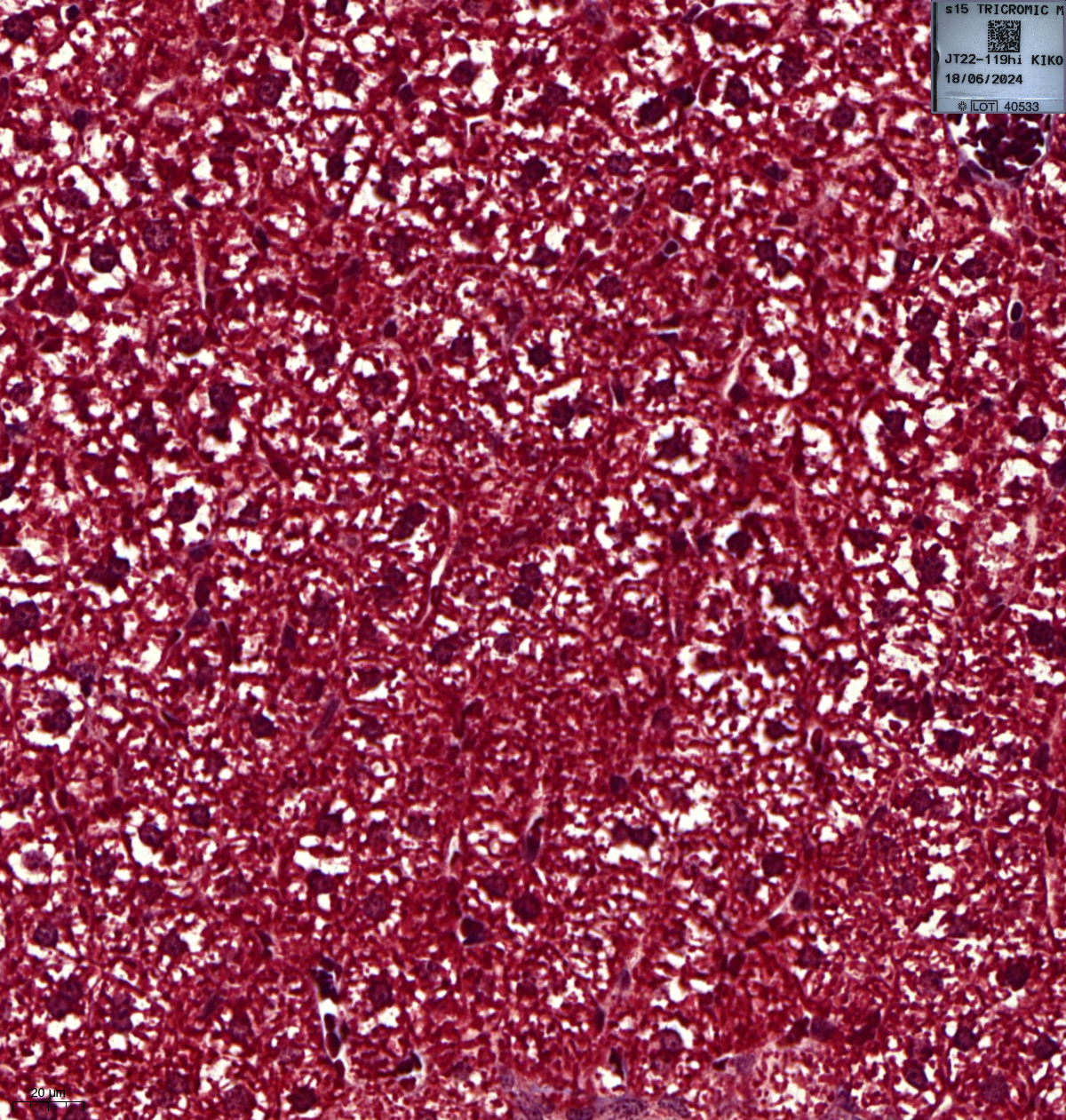

Supplement: Supplementary file 11 — Figure EV3 Source Data [file 44321_2026_426_MOESM11_ESM.zip › EV3 updated/Females/JT22-119hi Female KIKO AAV TRIC_40.0x.tif]

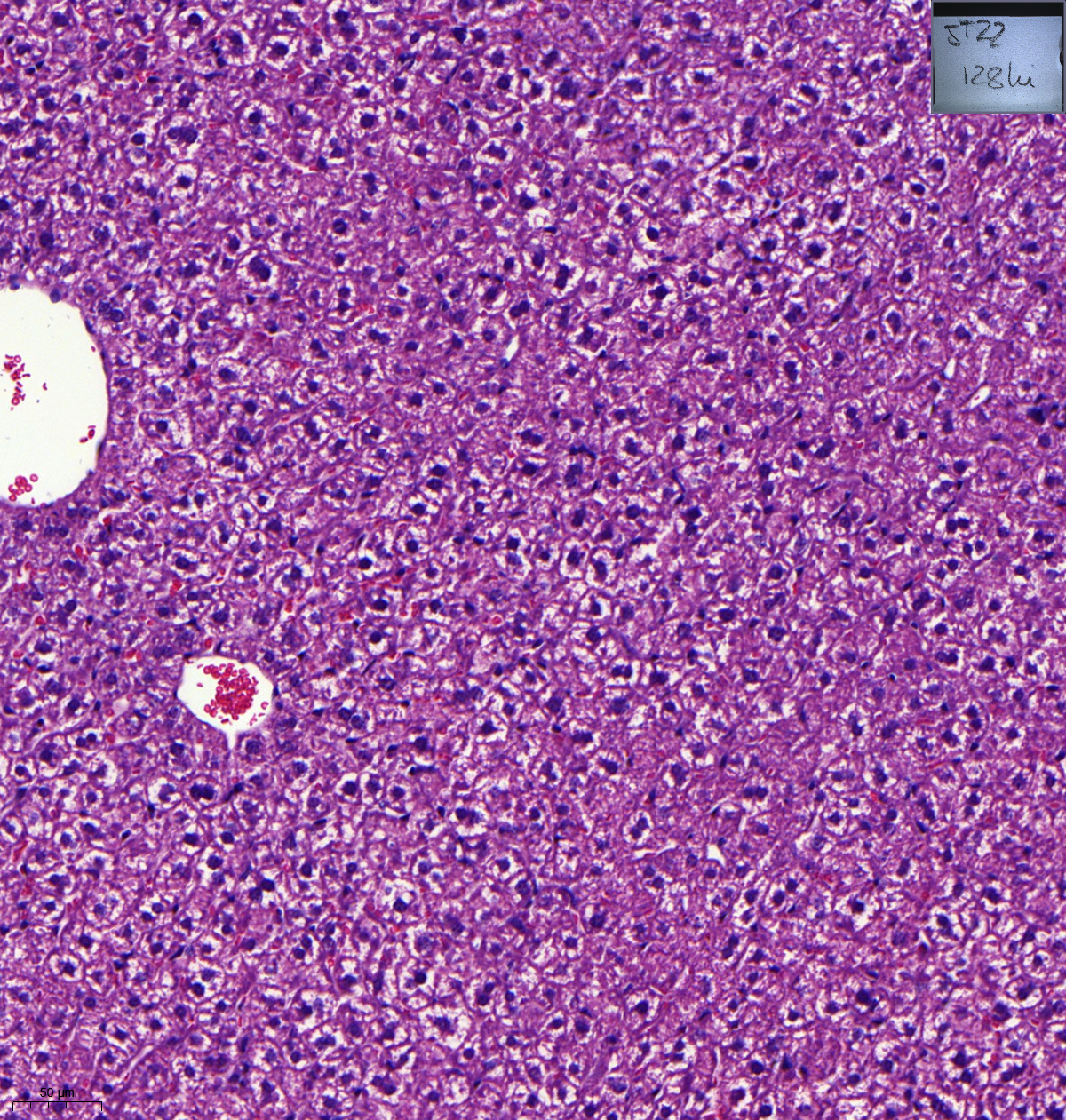

Supplement: Supplementary file 11 — Figure EV3 Source Data [file 44321_2026_426_MOESM11_ESM.zip › EV3 updated/Females/JT22-128hi Female KIKO HE_20.0x.tif]

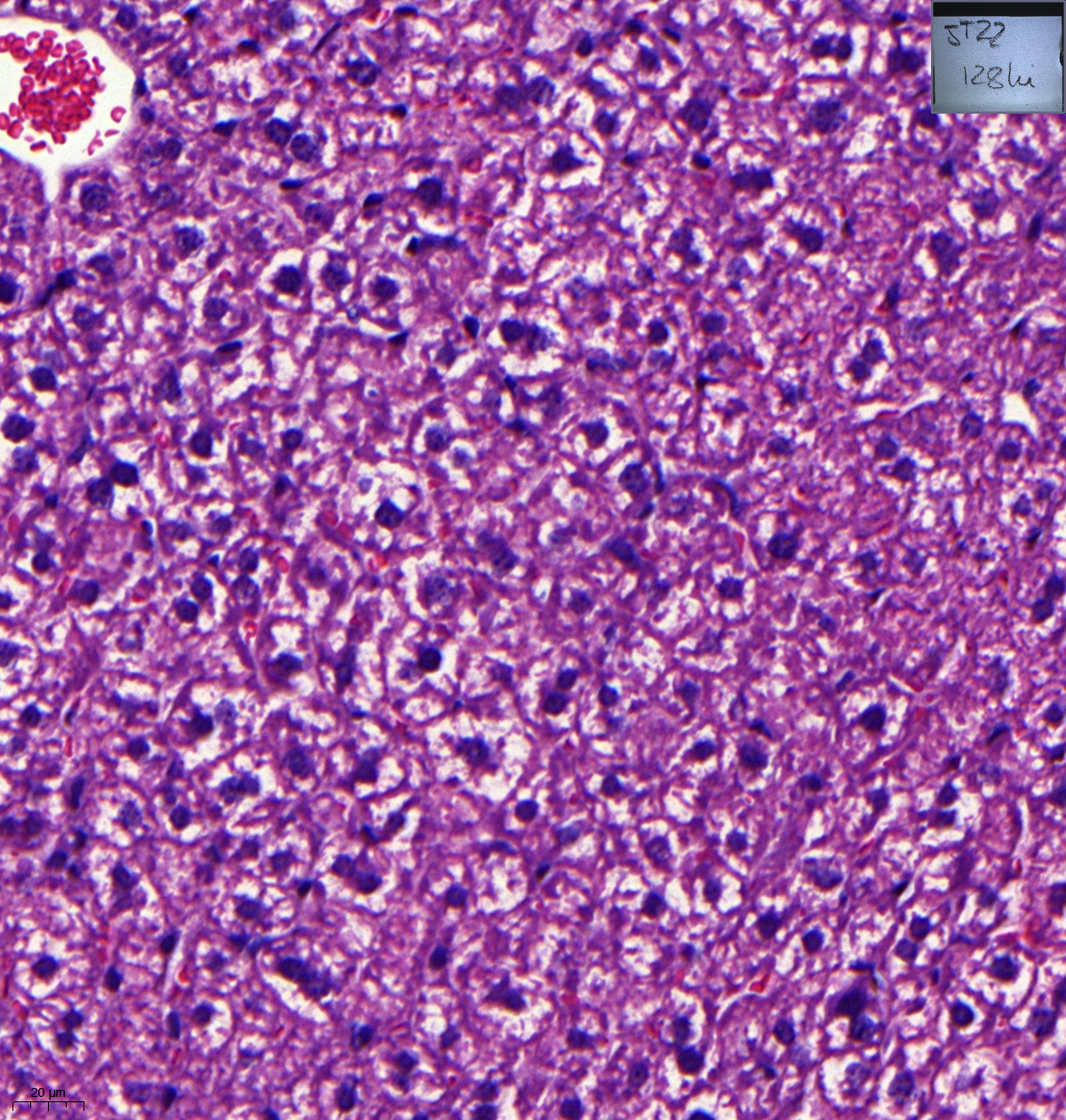

Supplement: Supplementary file 11 — Figure EV3 Source Data [file 44321_2026_426_MOESM11_ESM.zip › EV3 updated/Females/JT22-128hi Female KIKO HE_40.0x.tif]

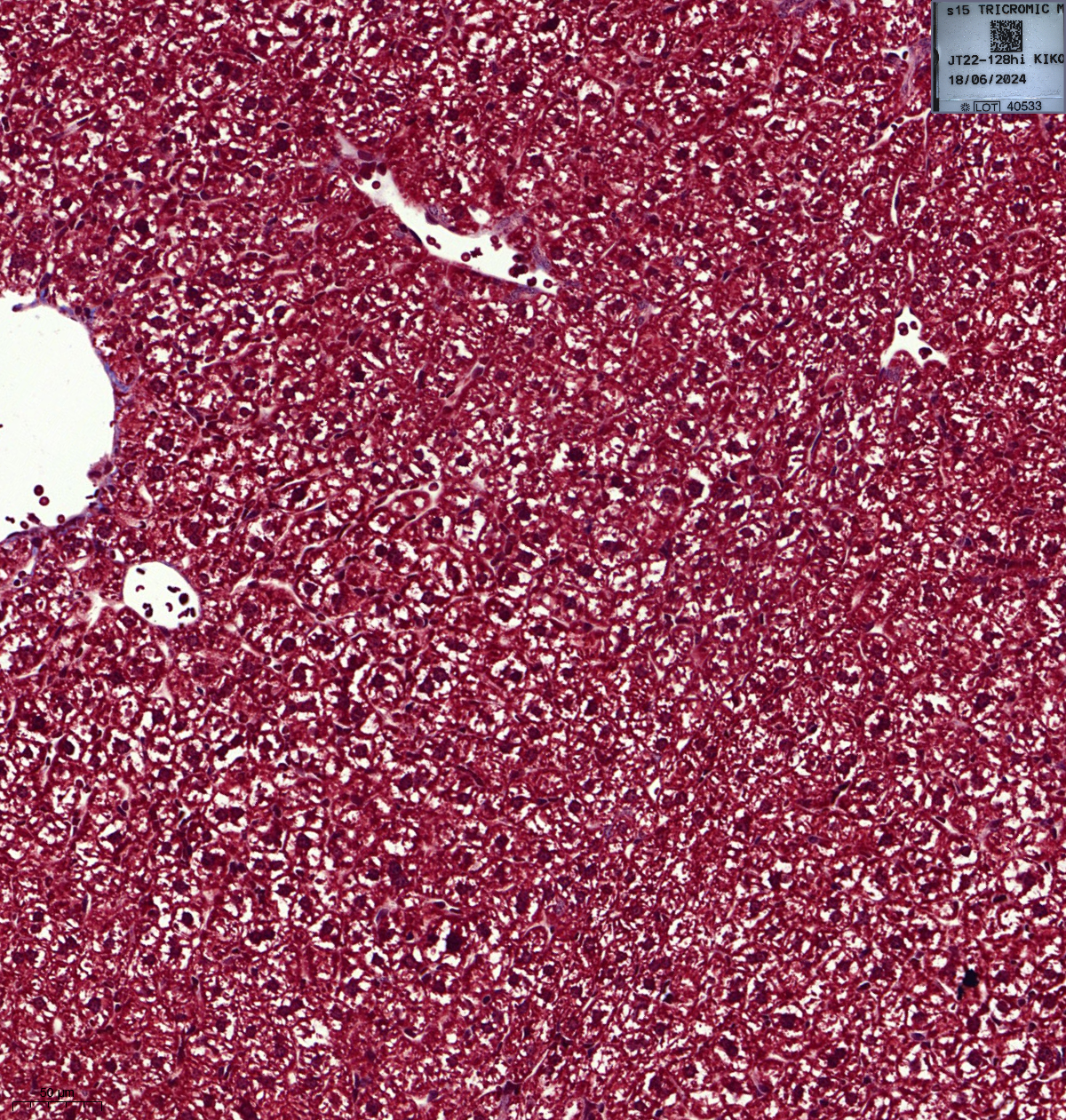

Supplement: Supplementary file 11 — Figure EV3 Source Data [file 44321_2026_426_MOESM11_ESM.zip › EV3 updated/Females/JT22-128hi Female KIKO TRIC_20.0x.tif]

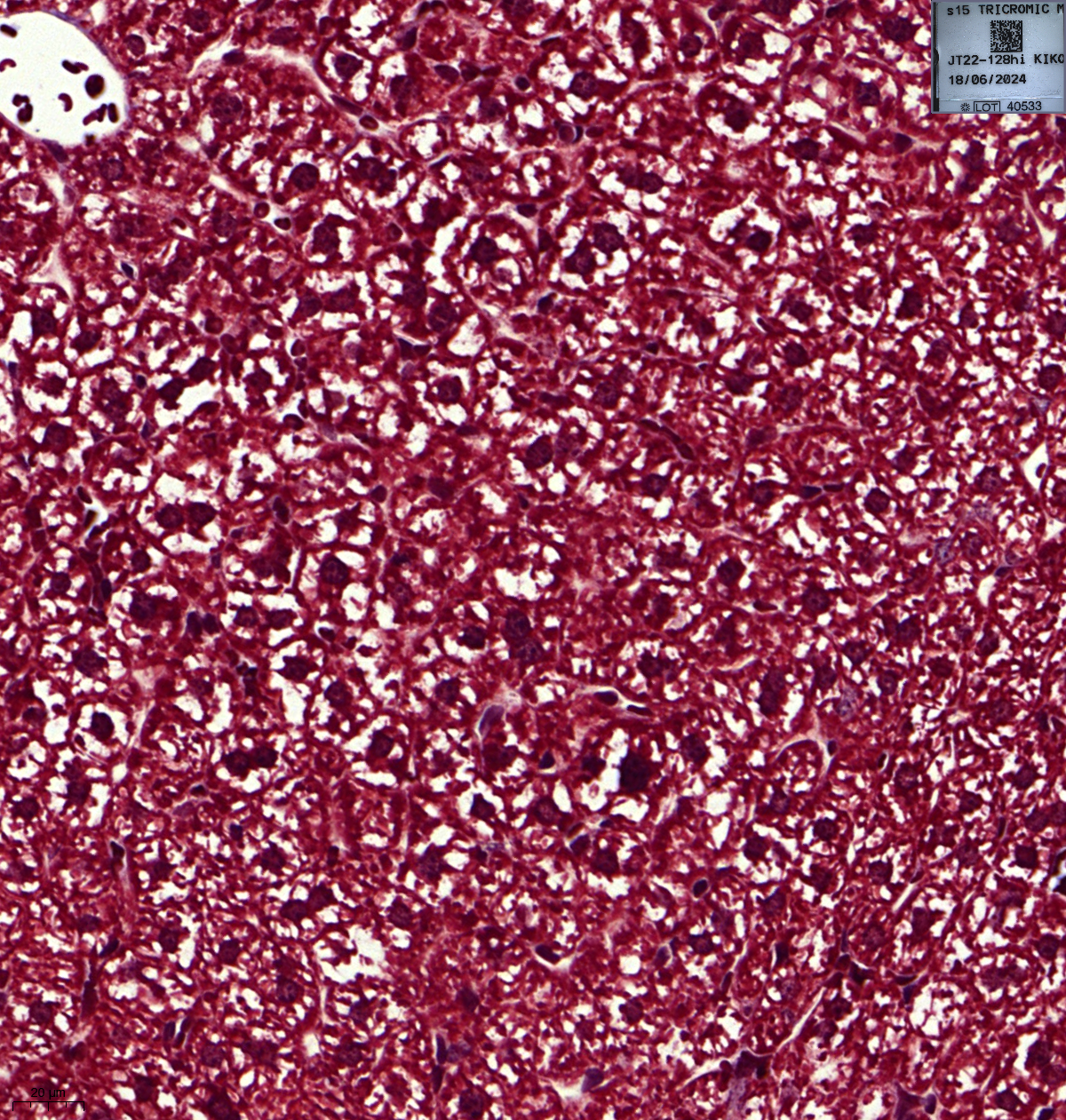

Supplement: Supplementary file 11 — Figure EV3 Source Data [file 44321_2026_426_MOESM11_ESM.zip › EV3 updated/Females/JT22-128hi Female KIKO TRIC_40.0x.tif]

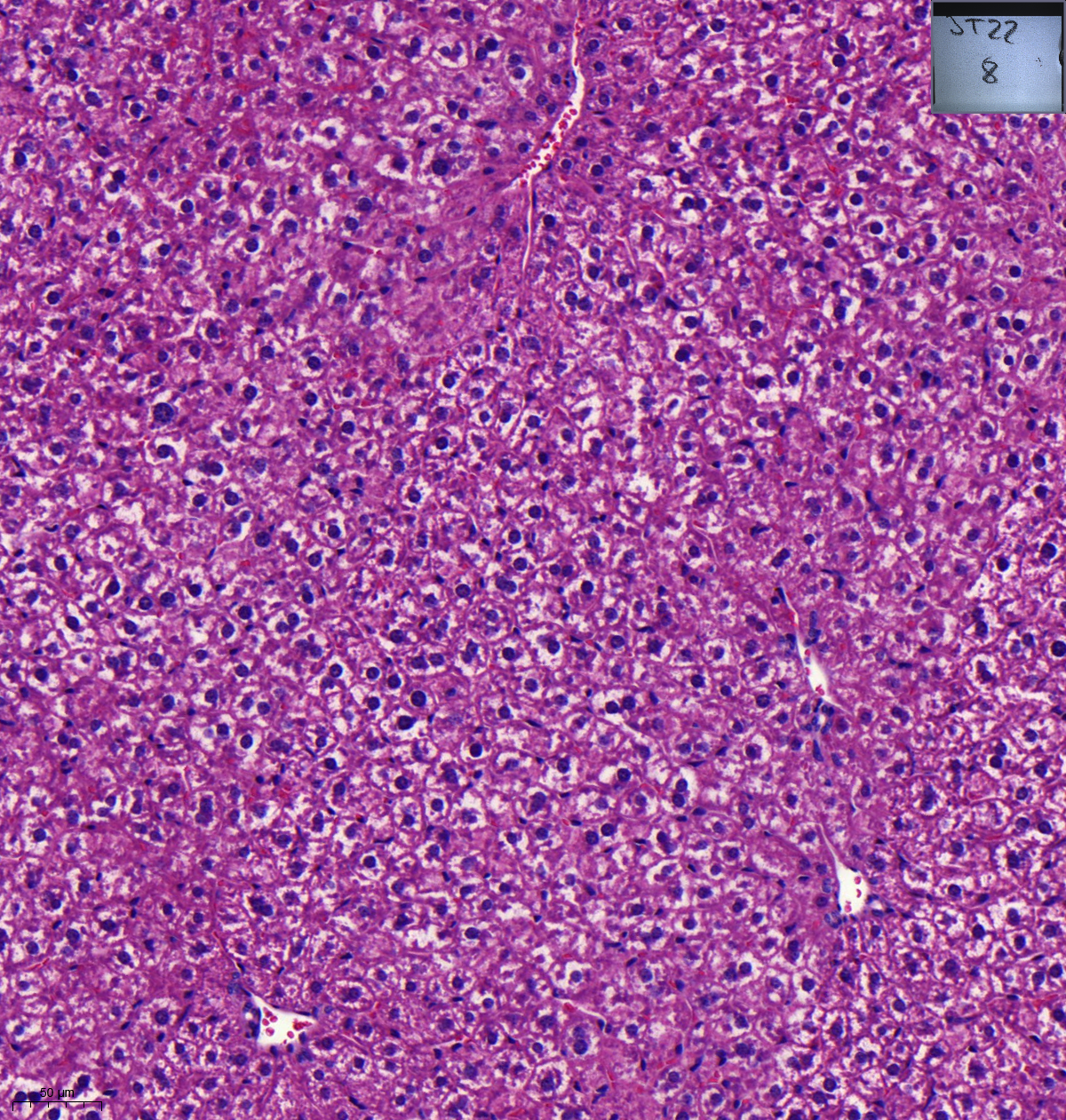

Supplement: Supplementary file 11 — Figure EV3 Source Data [file 44321_2026_426_MOESM11_ESM.zip › EV3 updated/Females/JT22-8hi Female WT HE_20.0x.tif]

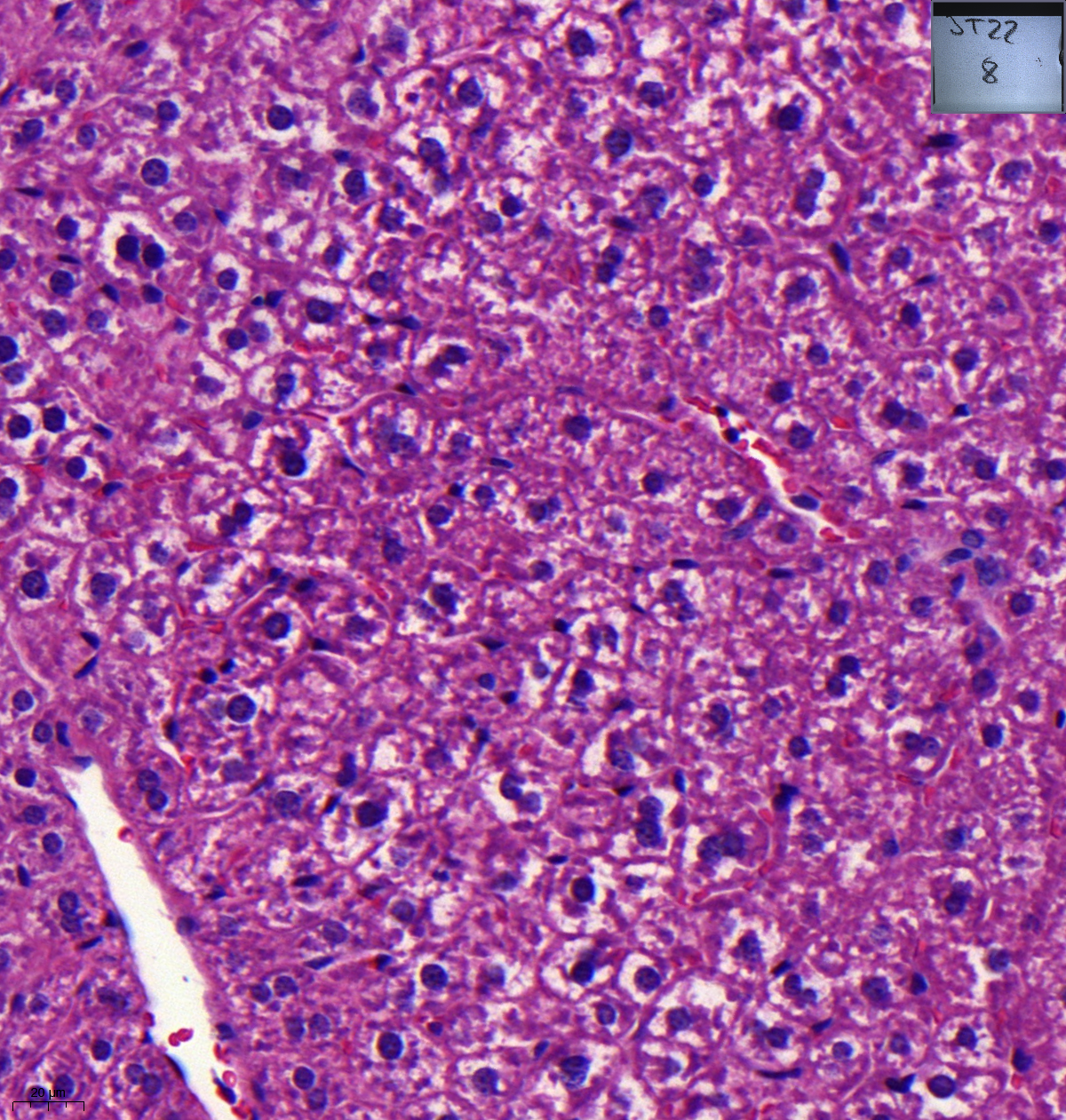

Supplement: Supplementary file 11 — Figure EV3 Source Data [file 44321_2026_426_MOESM11_ESM.zip › EV3 updated/Females/JT22-8hi Female WT HE_40.0x.tif]

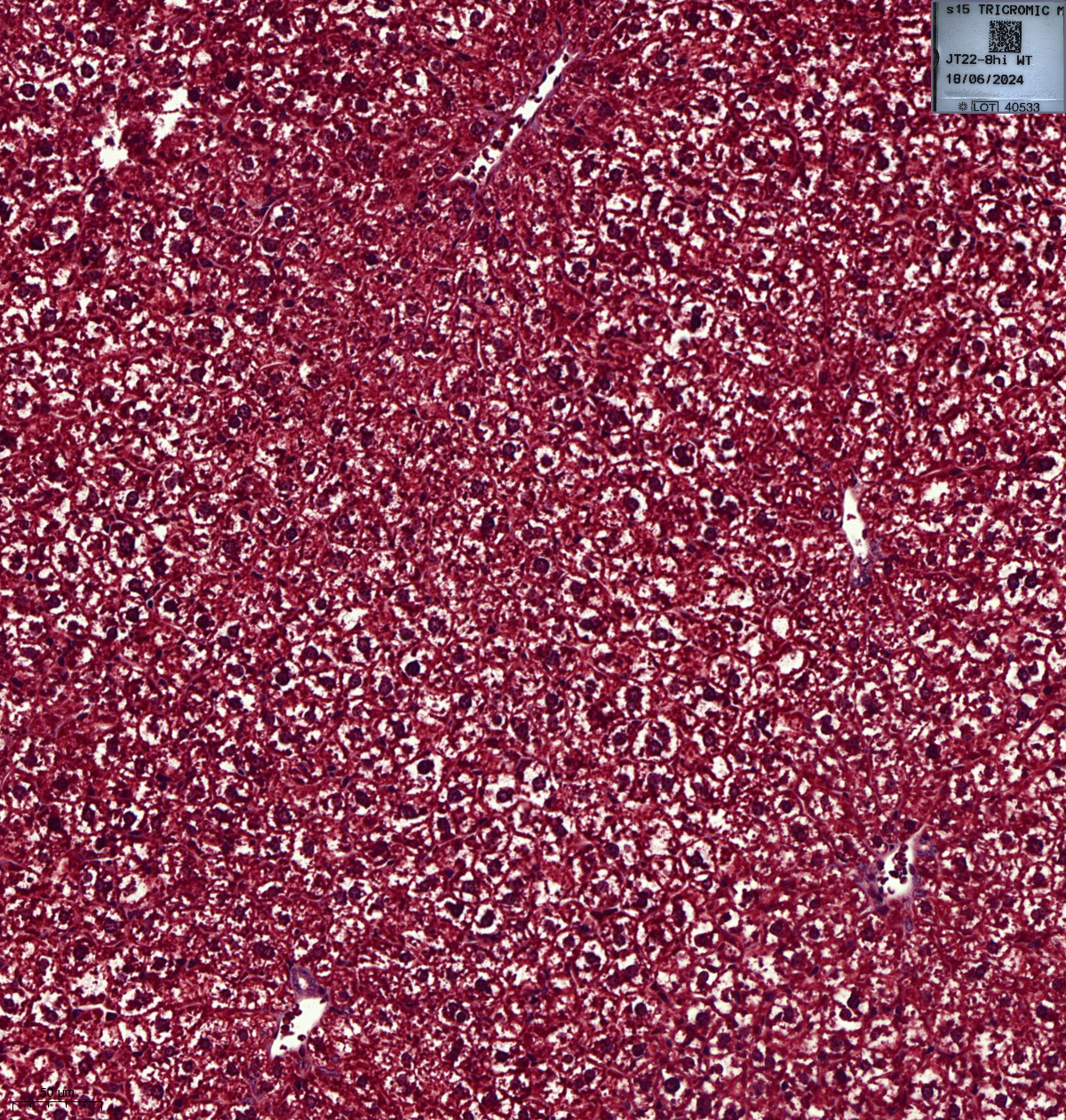

Supplement: Supplementary file 11 — Figure EV3 Source Data [file 44321_2026_426_MOESM11_ESM.zip › EV3 updated/Females/JT22-8hi Female WT TRIC_20.0x.tif]

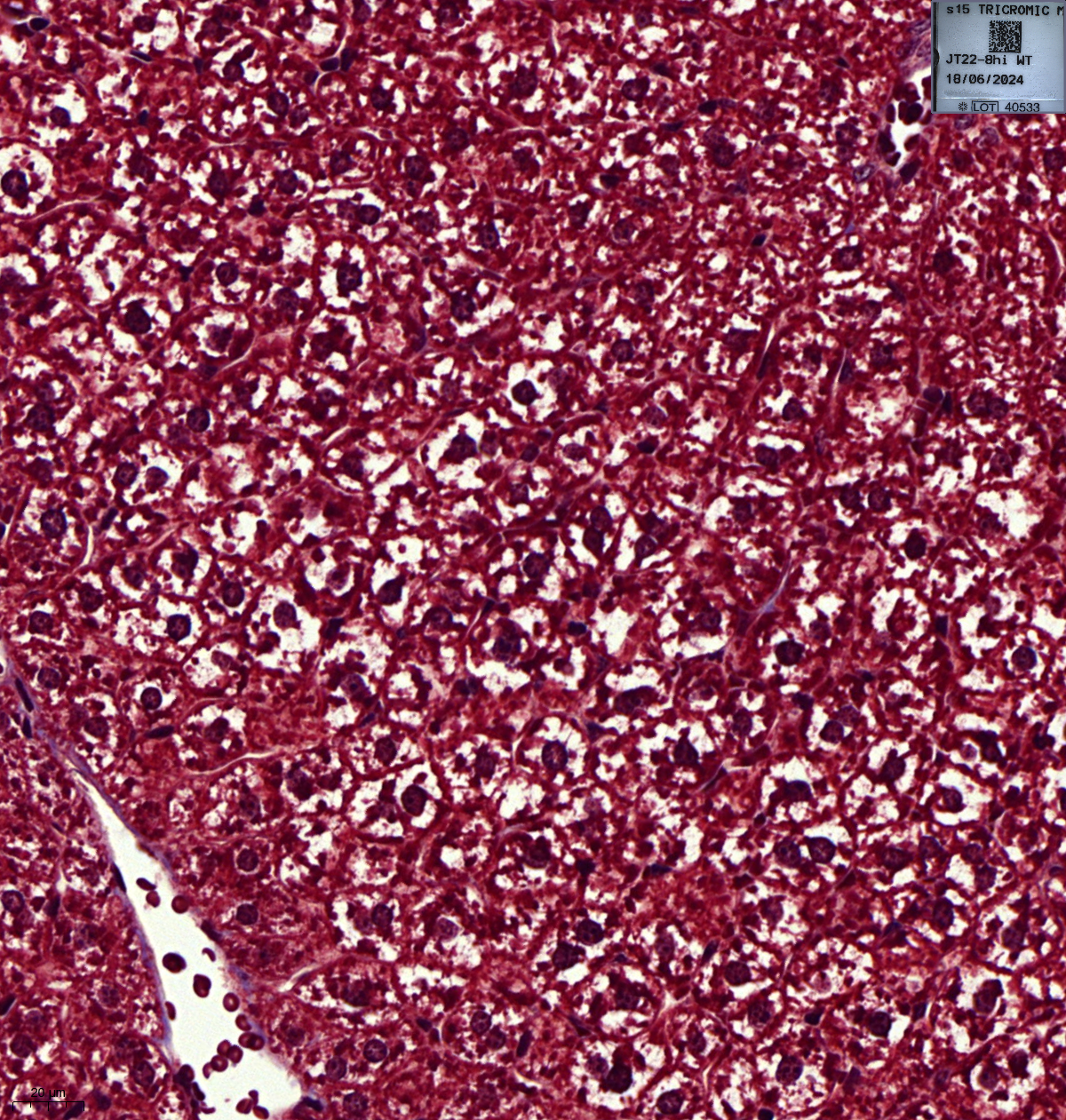

Supplement: Supplementary file 11 — Figure EV3 Source Data [file 44321_2026_426_MOESM11_ESM.zip › EV3 updated/Females/JT22-8hi Female WT TRIC_40.0x.tif]

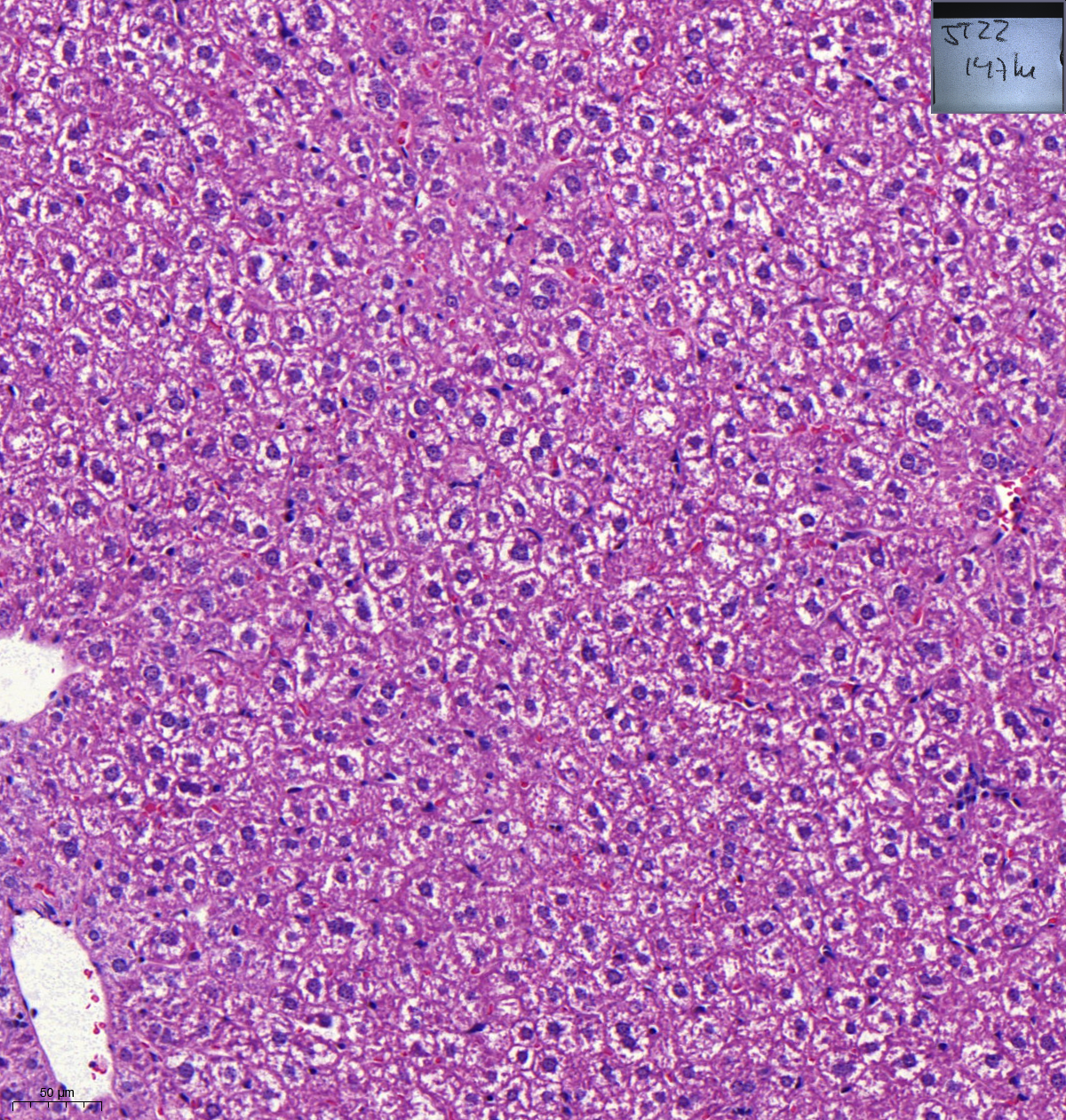

Supplement: Supplementary file 11 — Figure EV3 Source Data [file 44321_2026_426_MOESM11_ESM.zip › EV3 updated/Males/JT22-147hi Male KIKO HE_20.0x.tif]

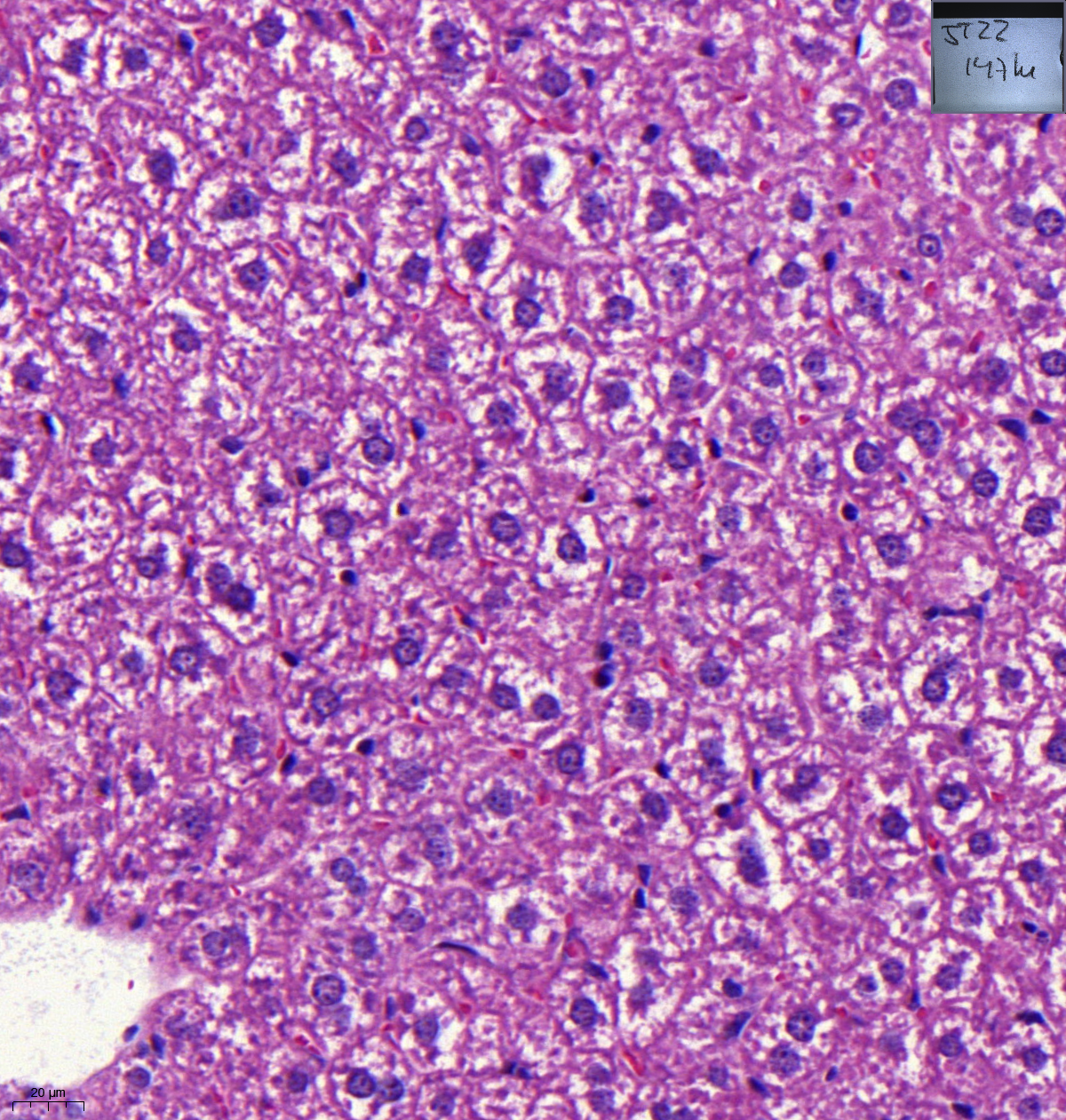

Supplement: Supplementary file 11 — Figure EV3 Source Data [file 44321_2026_426_MOESM11_ESM.zip › EV3 updated/Males/JT22-147hi Male KIKO HE_40.0x.tif]

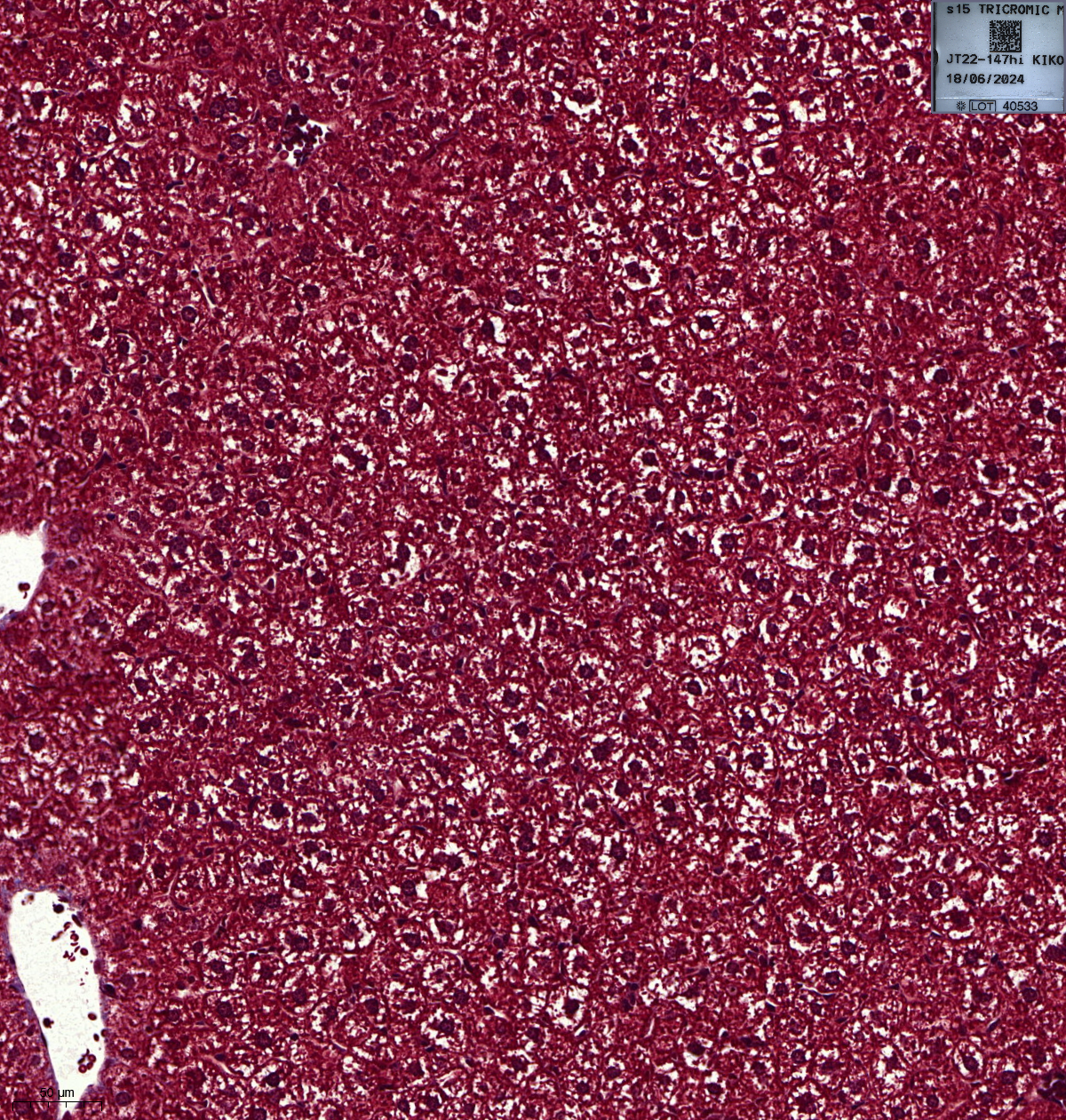

Supplement: Supplementary file 11 — Figure EV3 Source Data [file 44321_2026_426_MOESM11_ESM.zip › EV3 updated/Males/JT22-147hi Male KIKO TRIC_20.0x.tif]

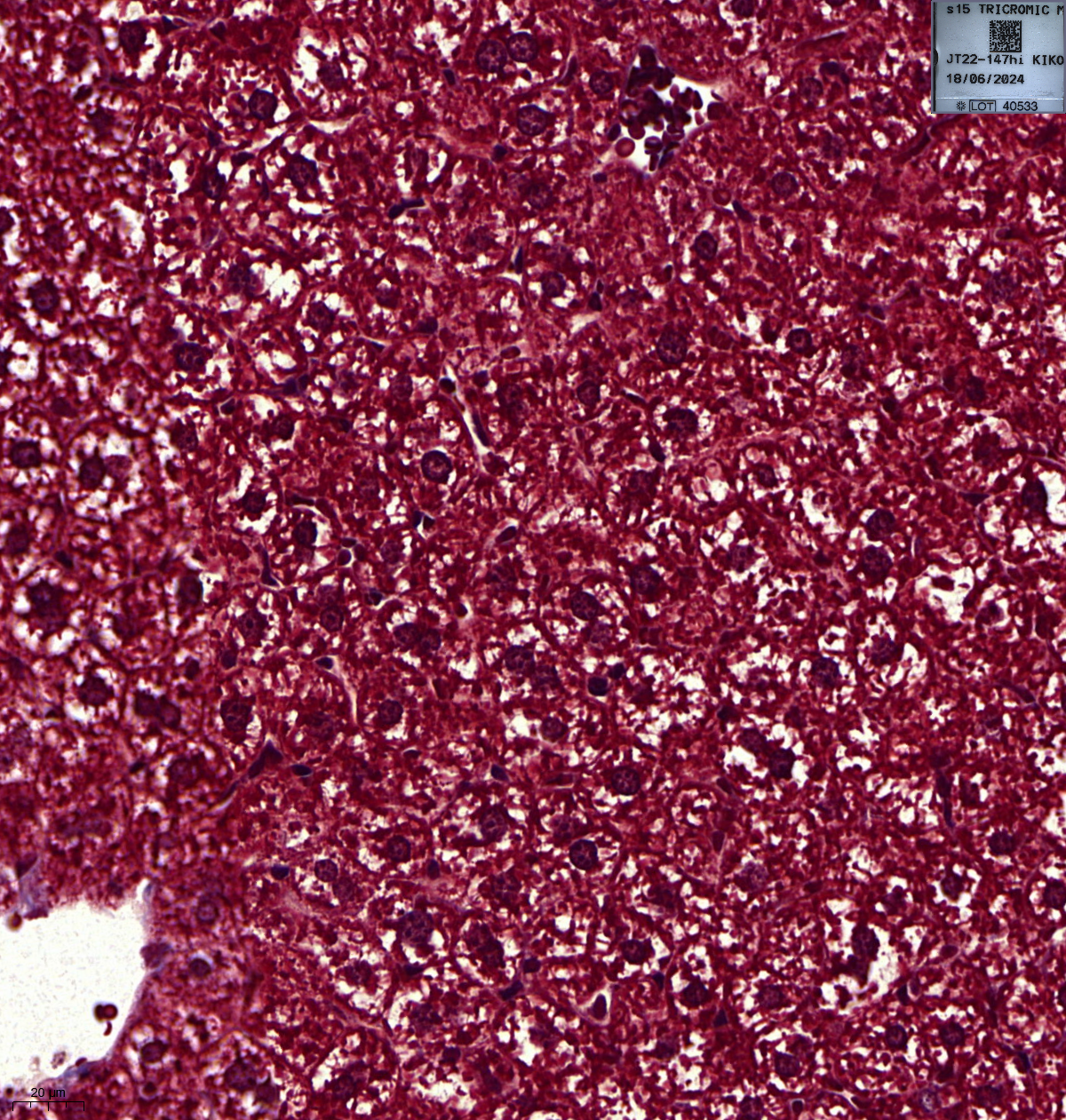

Supplement: Supplementary file 11 — Figure EV3 Source Data [file 44321_2026_426_MOESM11_ESM.zip › EV3 updated/Males/JT22-147hi Male KIKO TRIC_40.0x.tif]

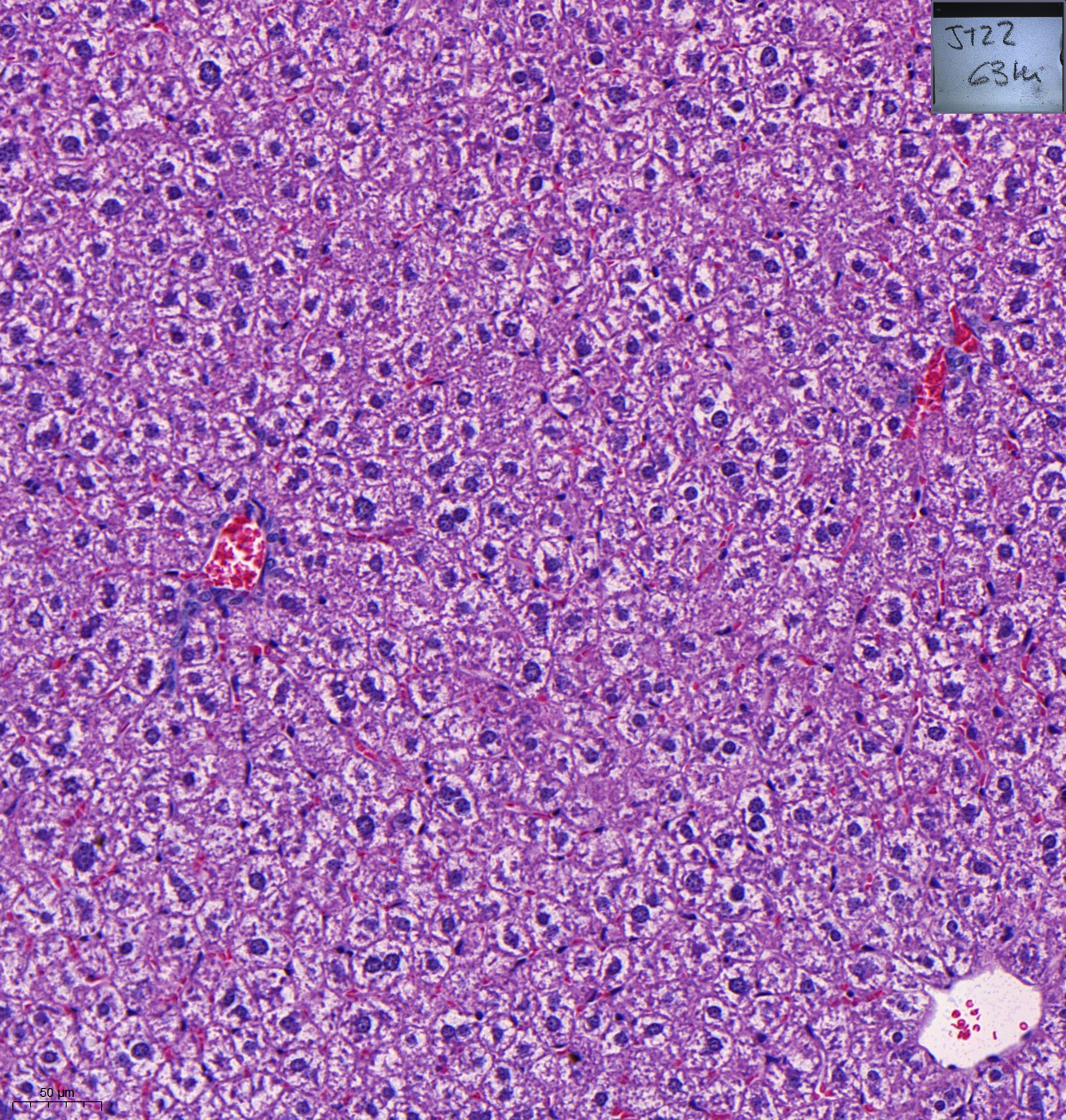

Supplement: Supplementary file 11 — Figure EV3 Source Data [file 44321_2026_426_MOESM11_ESM.zip › EV3 updated/Males/JT22-63hi Male WT HE_20.0x.tif]

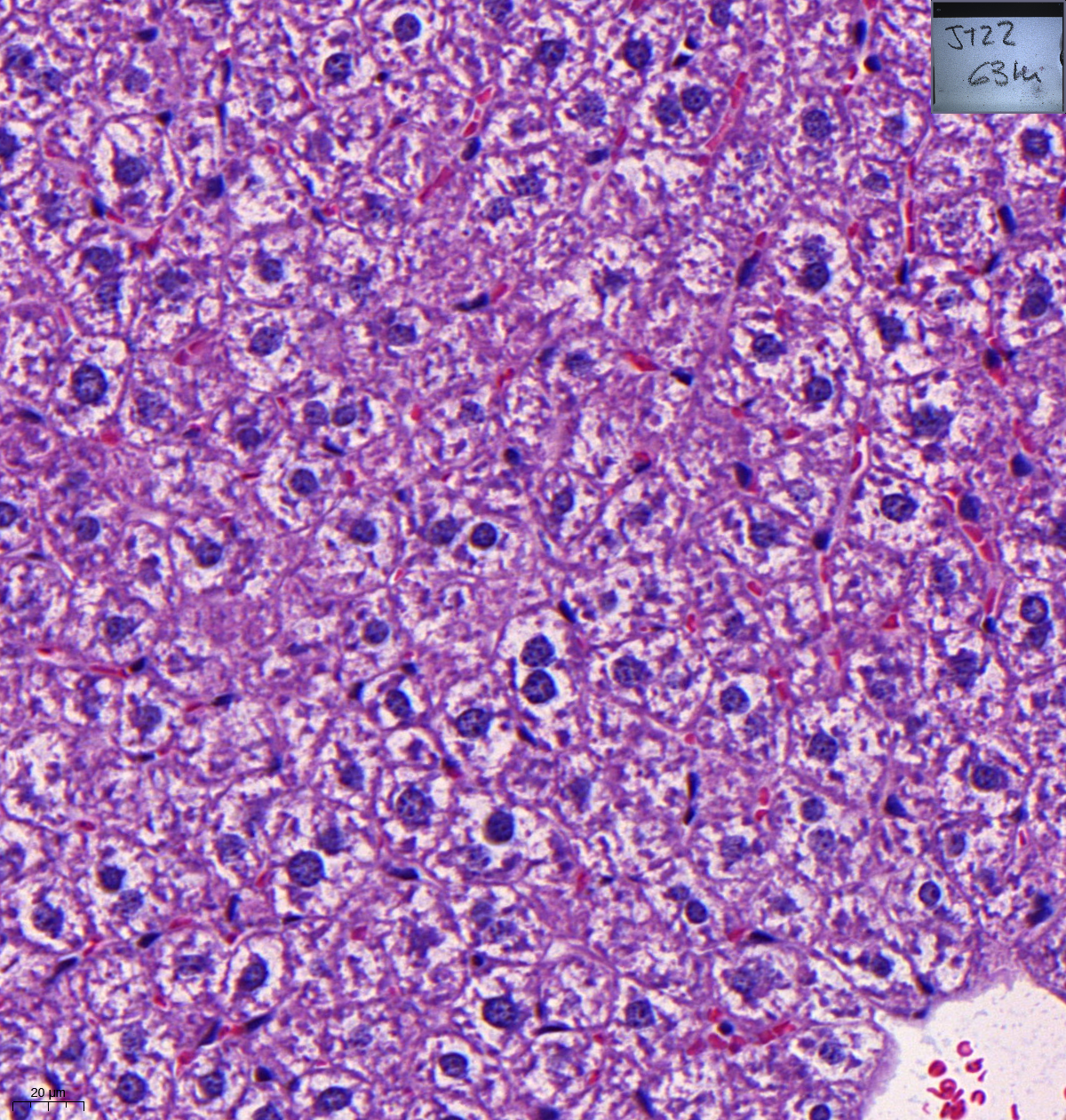

Supplement: Supplementary file 11 — Figure EV3 Source Data [file 44321_2026_426_MOESM11_ESM.zip › EV3 updated/Males/JT22-63hi Male WT HE_40.0x.tif]

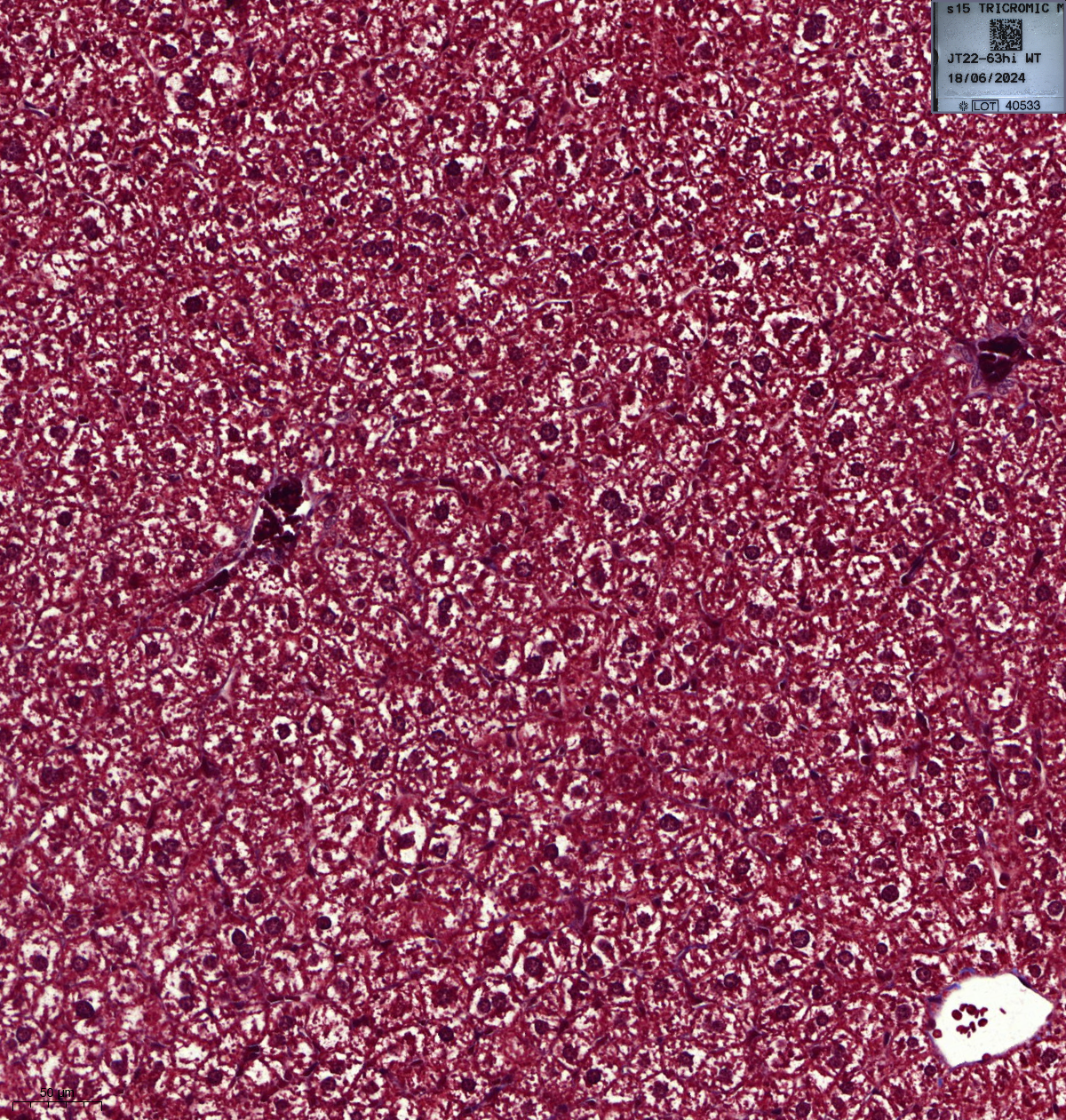

Supplement: Supplementary file 11 — Figure EV3 Source Data [file 44321_2026_426_MOESM11_ESM.zip › EV3 updated/Males/JT22-63hi Male WT TRIC_20.0x.tif]

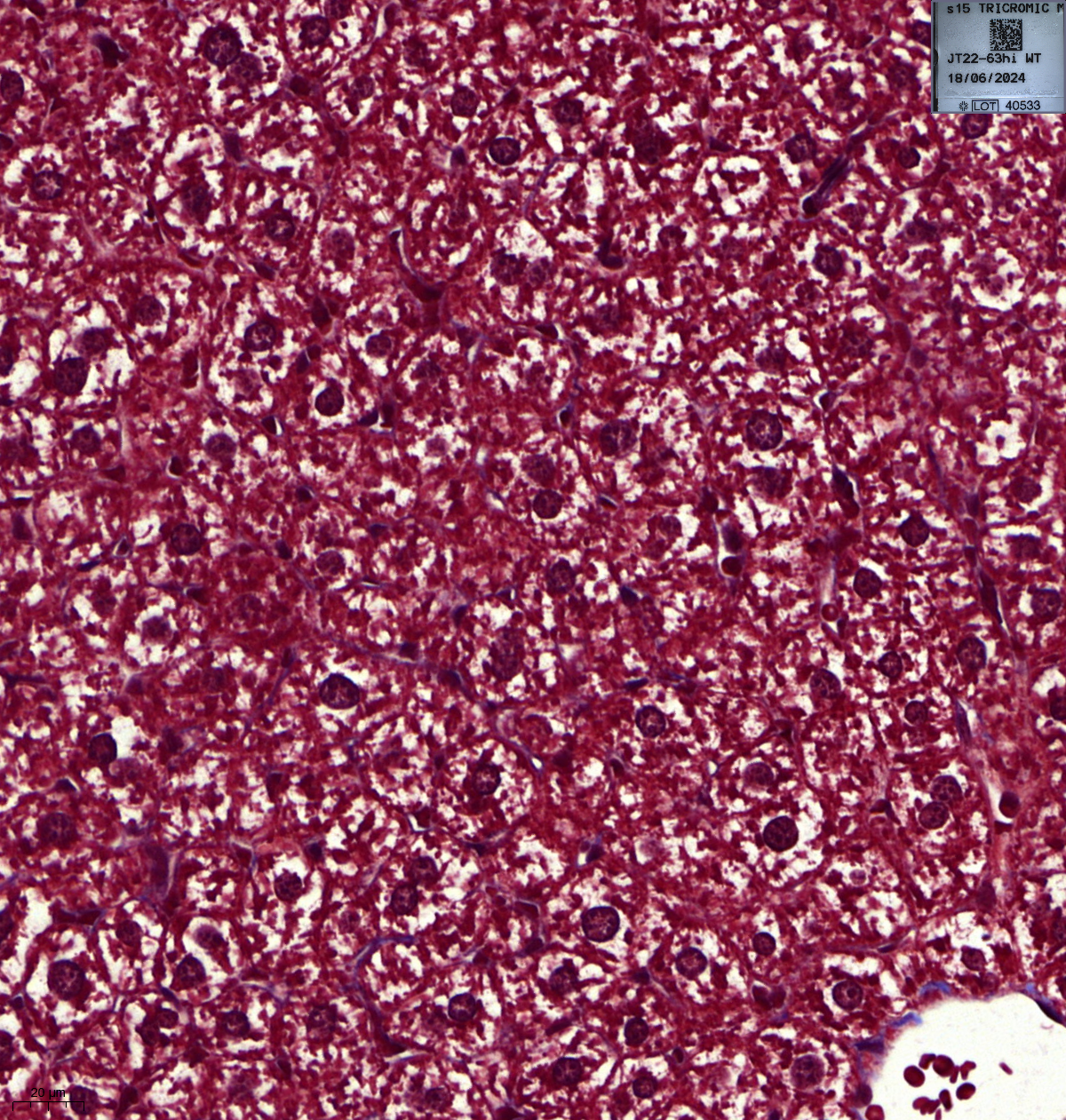

Supplement: Supplementary file 11 — Figure EV3 Source Data [file 44321_2026_426_MOESM11_ESM.zip › EV3 updated/Males/JT22-63hi Male WT TRIC_40.0x.tif]

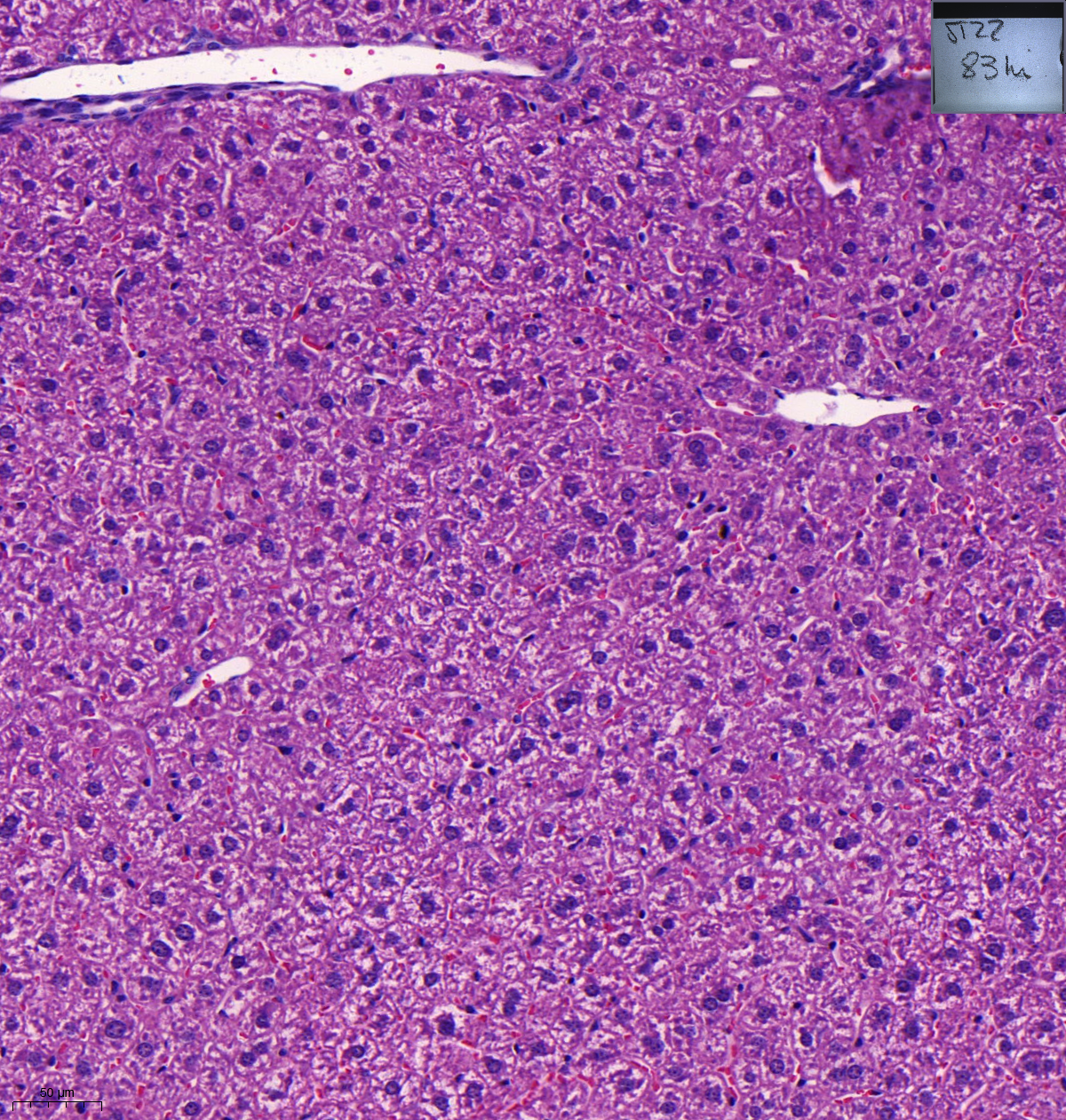

Supplement: Supplementary file 11 — Figure EV3 Source Data [file 44321_2026_426_MOESM11_ESM.zip › EV3 updated/Males/JT22-83hi Male KIKO AAV HE_20.0x.tif]

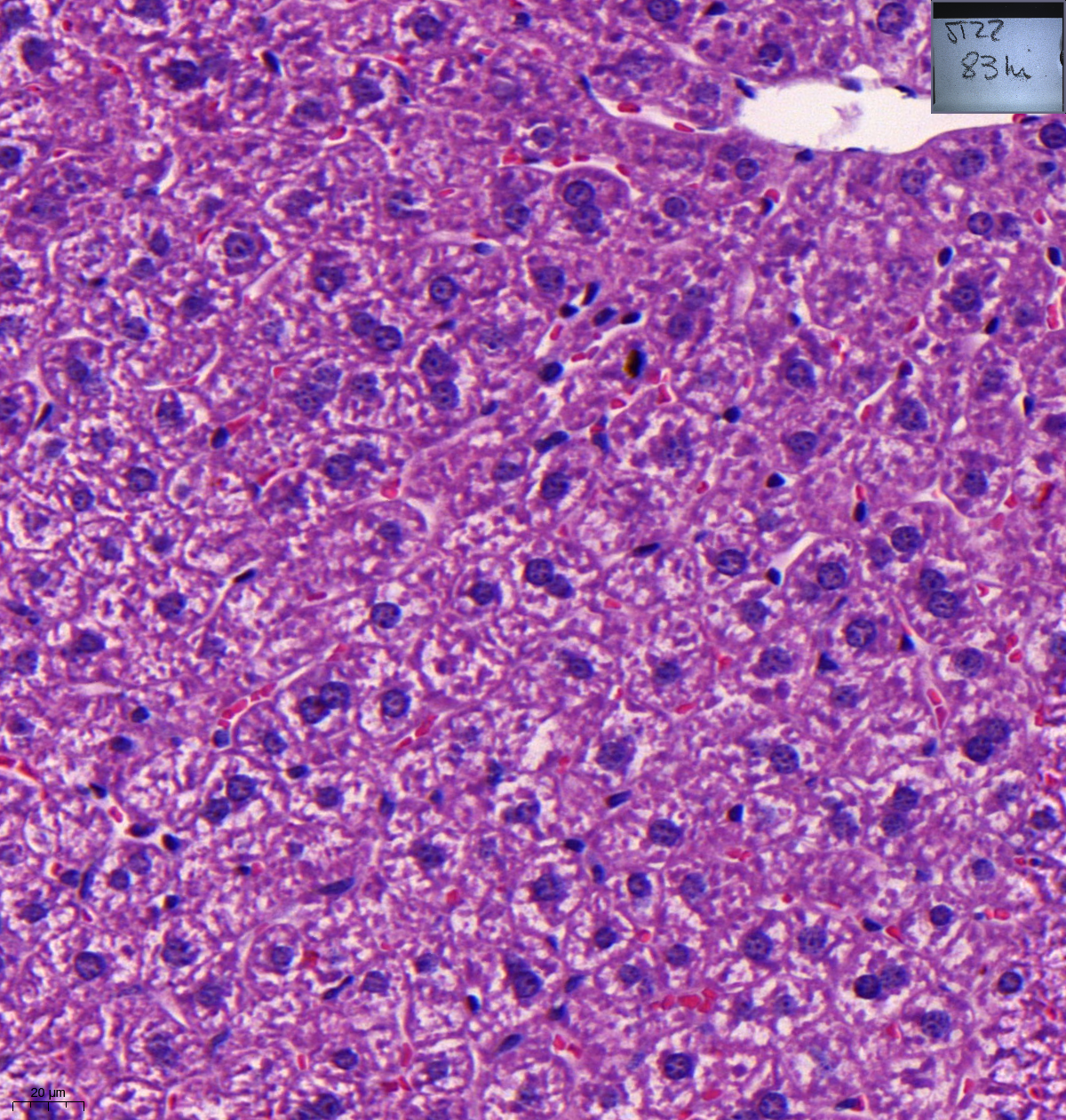

Supplement: Supplementary file 11 — Figure EV3 Source Data [file 44321_2026_426_MOESM11_ESM.zip › EV3 updated/Males/JT22-83hi Male KIKO AAV HE_40.0x.tif]

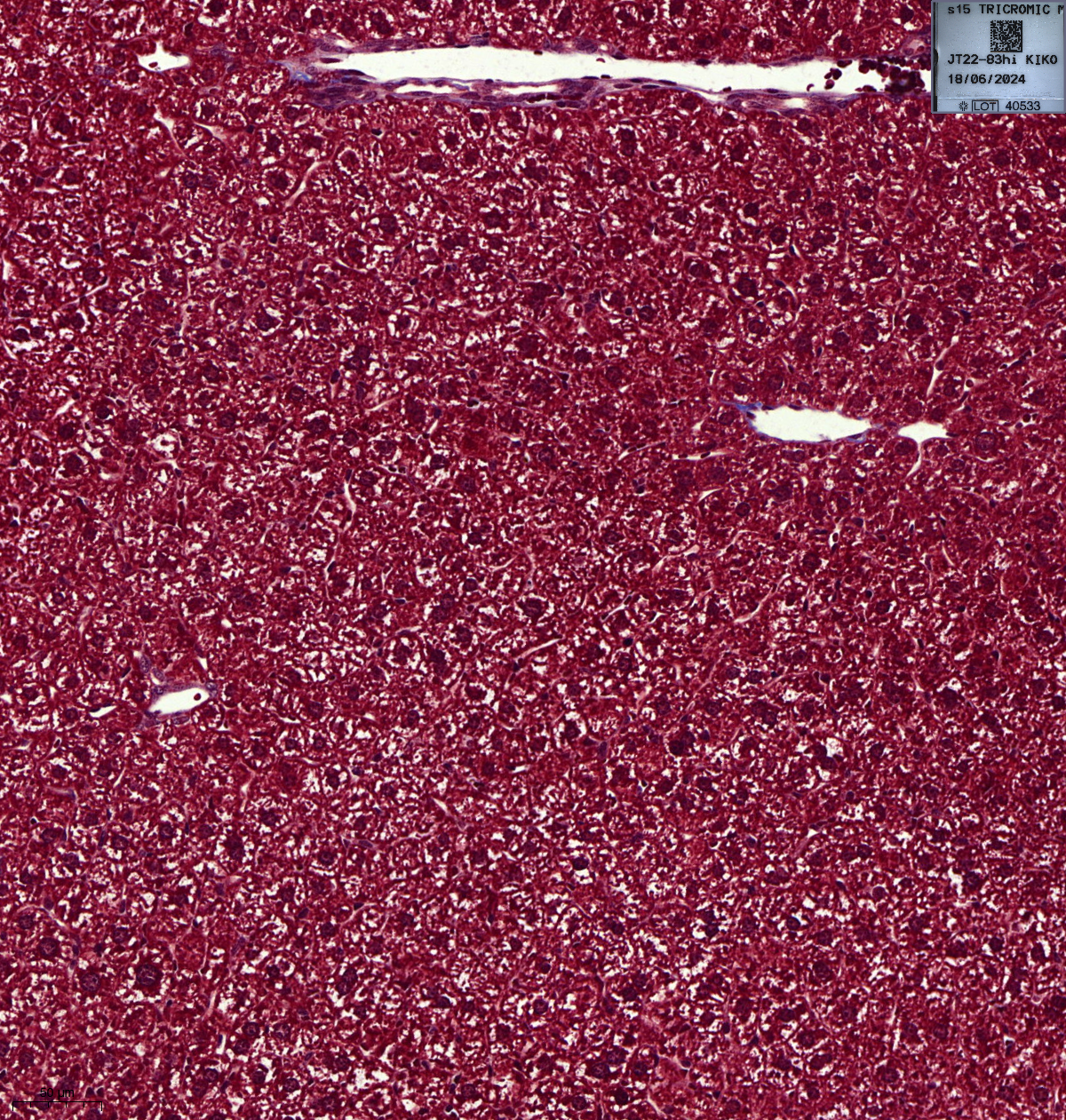

Supplement: Supplementary file 11 — Figure EV3 Source Data [file 44321_2026_426_MOESM11_ESM.zip › EV3 updated/Males/JT22-83hi Male KIKO AAV TRIC_20.0x.tif]

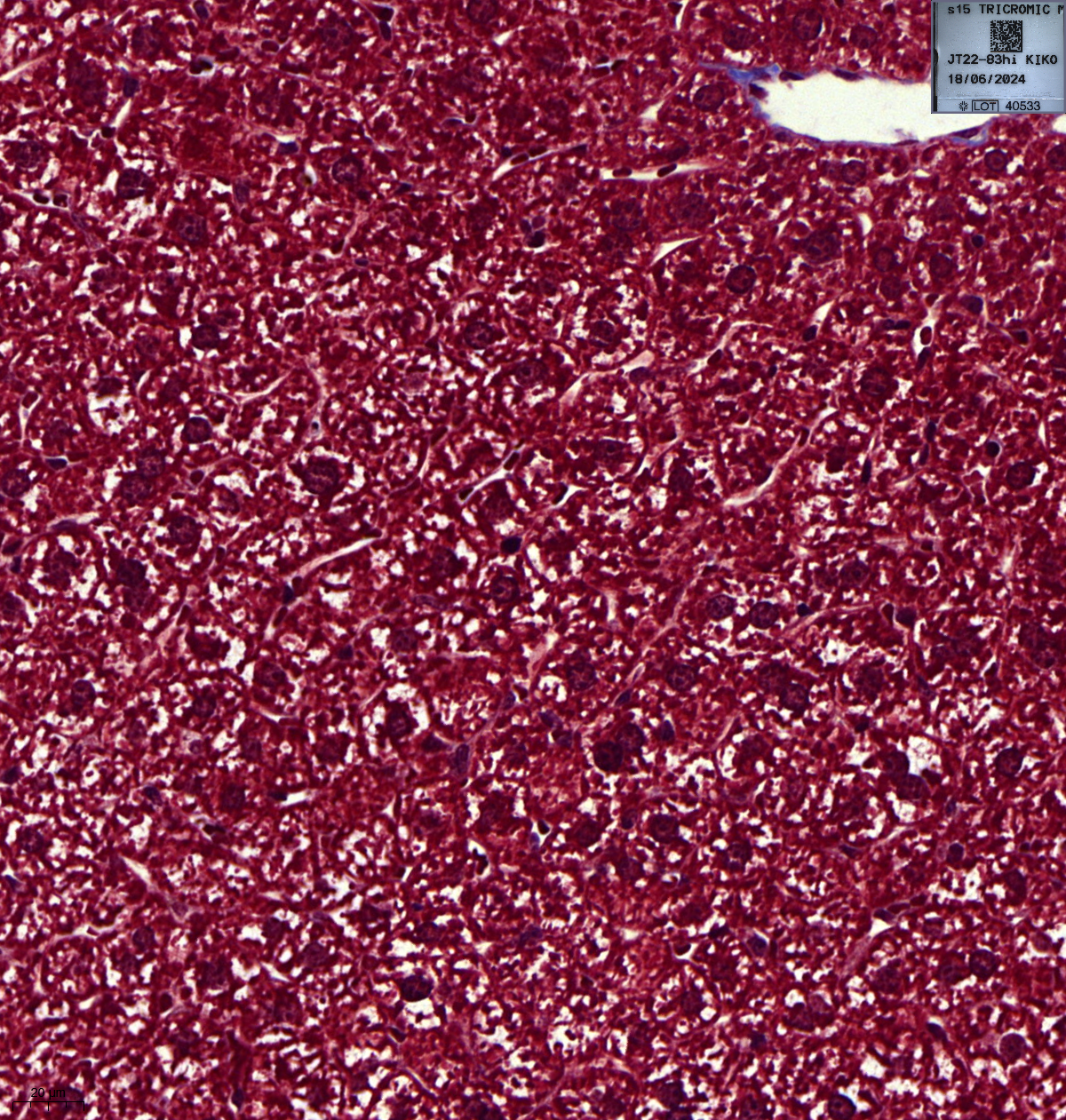

Supplement: Supplementary file 11 — Figure EV3 Source Data [file 44321_2026_426_MOESM11_ESM.zip › EV3 updated/Males/JT22-83hi Male KIKO AAV TRIC_40.0x.tif]
