# Supplementary material for: Systemic delivery of AAV-GFM1 corrects COXPD1 molecular alterations in Gfm1R671C/− mice
Source: EMBO Mol Med. 2026 Apr 17;18(6):2152–79. doi: 10.1038/s44321-026-00426-4 (PMC13269562; doi:10.1038/s44321-026-00426-4)
Supplement: Supplementary file 14 — Appendix [file 44321_2026_426_MOESM14_ESM.pdf]

APPENDIX

TABLE OF CONTENTS

Contains four Appendix Figures and one Appendix Table:

Appendix Figure S1. Lentiviral vectors used for the in vitro study......1
Appendix Figure S2. rAAV genomes, study groups and experimental design.......2
Appendix Figure S3. Body weight monitoring and tissue weights.......3
Appendix Figure S4. Mitochondrial content and mtDNA copy number in liver and brain from 8-week-old *Gfm1*$^{R671C/-}$ mice. 4
Appendix Table S1. Exact p-values and numbers of animals or replicates for each figure.......5

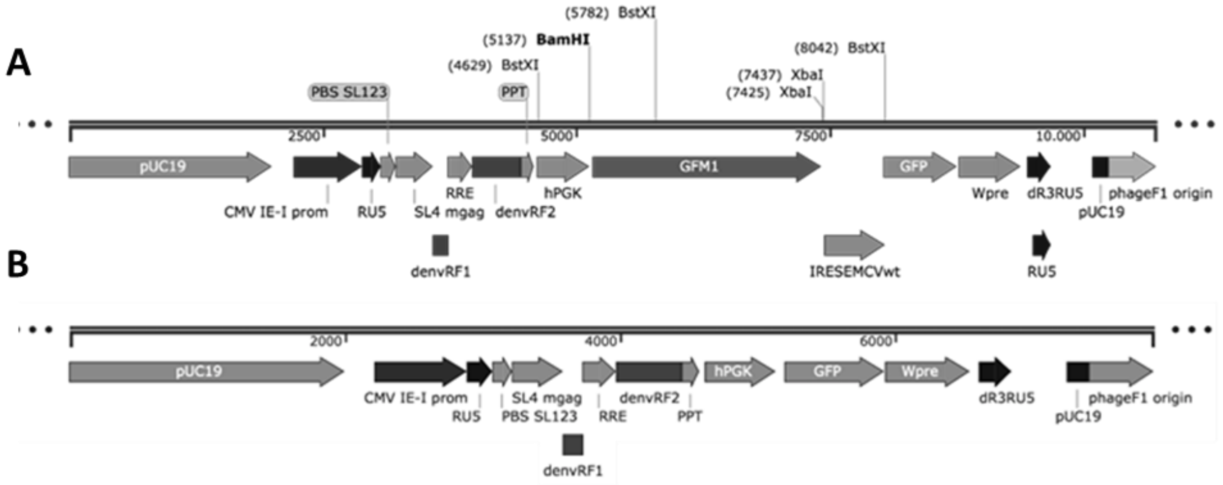

Appendix Figure S1. Lentiviral vectors used for the in vitro study

Schematic representation of the **(A)** LVs p305-GFM1LV and **B)** pSham LV. RRE: Rev response element; PPT: central polypurine tract; hPGK: human phosphoglycerate kinase promotor; IRES: internal ribosome entry site; GFP: green fluorescent protein; Wpre: woodchuck hepatitis virus posttranscriptional regulatory element.

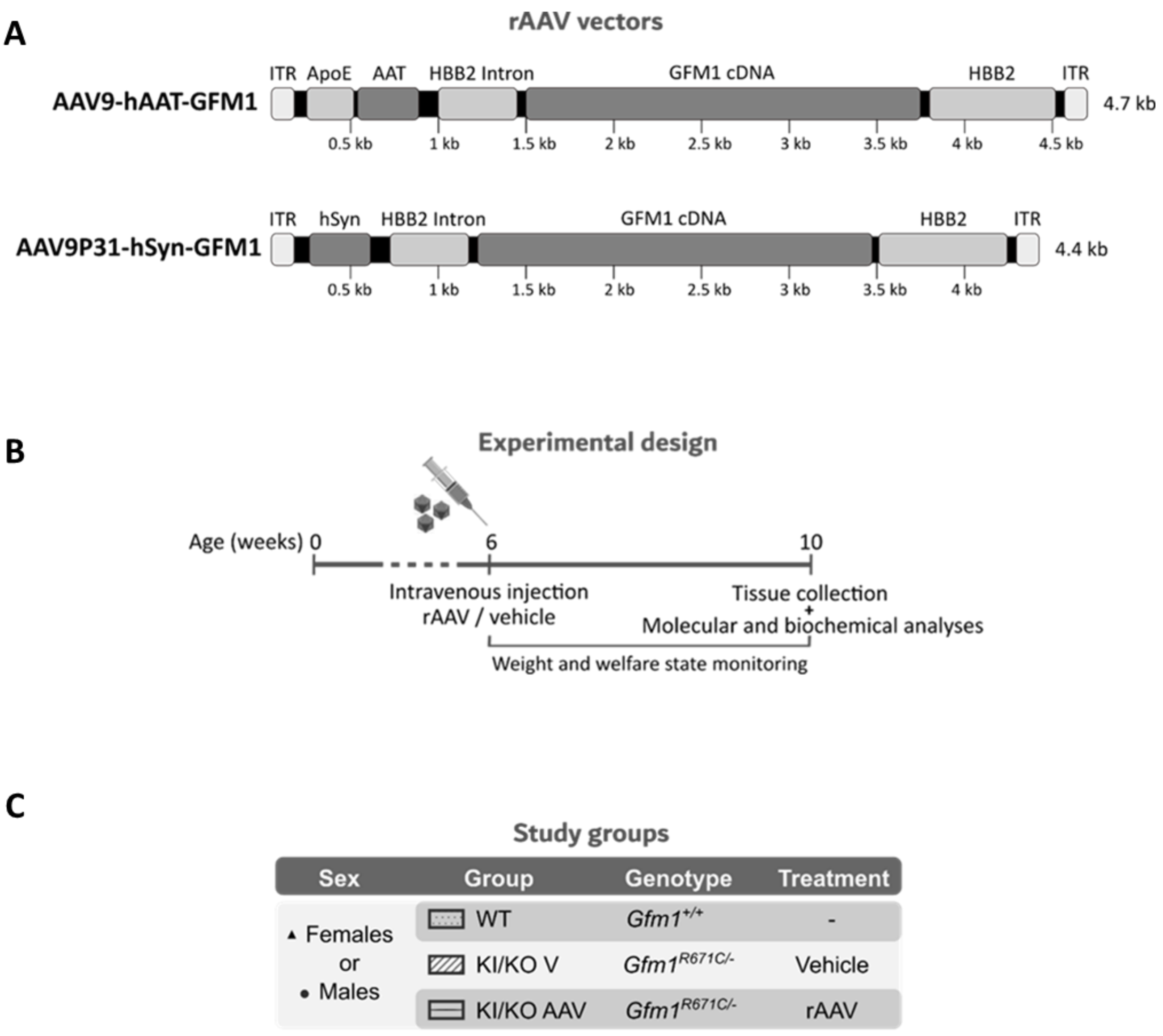

Appendix Figure S2. rAAV genomes, study groups and experimental design.

(A) Schematic representation of the recombinant AAV vectors (rAAV) studied. Both vectors are single stranded and flanked by AAV2 inverted terminal repeats. The liver-targeted vector (AAV9-hAAT-GFM1) was pseudotyped with the AAV9 capsid, while the CNS-targeted vector (AAV9P31-hSyn-GFM1) has the modified AAV9 capsid AAV9P31. ITR, inverted terminal repeats; ApoE, ApoE enhancer element; hAAT, α-1-antitrypsin promoter; HBB2 intron, bovine hemoglobin subunit b-2 intron; GFM1 cDNA, human *GFM1* coding sequence; HBB2, bovine hemoglobin subunit b-2 polyadenylation signal; hSyn, human synapsin 1 gene promoter. **(B)** Schematic representation of the experimental design for studying both vectors. Each rAAV vector was i.v. administered at a single $5x10^{12}$ vg/kg dose. A solution of saccharose 5% (w/v) in PBS + 0.001% pluronic acid F68 was used as vehicle. **(C)** Study groups.

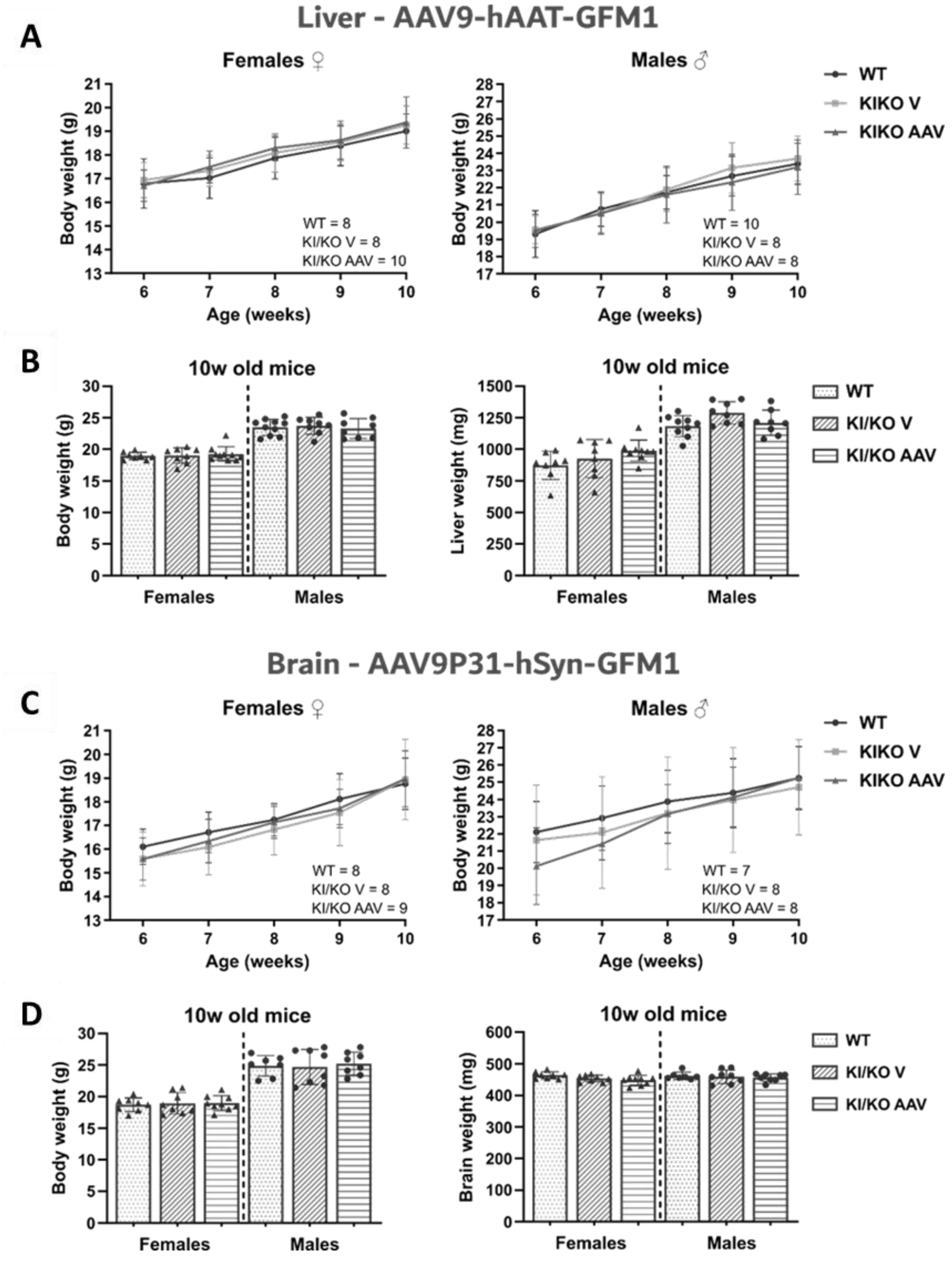

Appendix Figure S3. Body weight monitoring and tissue weights.
(**A**) Body weight monitoring of mice treated with the liver-targeted vector and (**B**) total body and liver weights 4 weeks after the vector administration; (**C**) body weight monitoring of mice treated with the CNS-targeted vector, and (**D**) total body and brain weights 4 weeks after the vector administration. Each point represents an animal measurement and bars represent the group mean (±SD). WT: wild type mice; KI/KO V: *Gfm1*$^{R671C/-}$ mice treated with vehicle; KI/KO AAV: *Gfm1*$^{R671C/-}$ mice treated with the therapeutic vector.

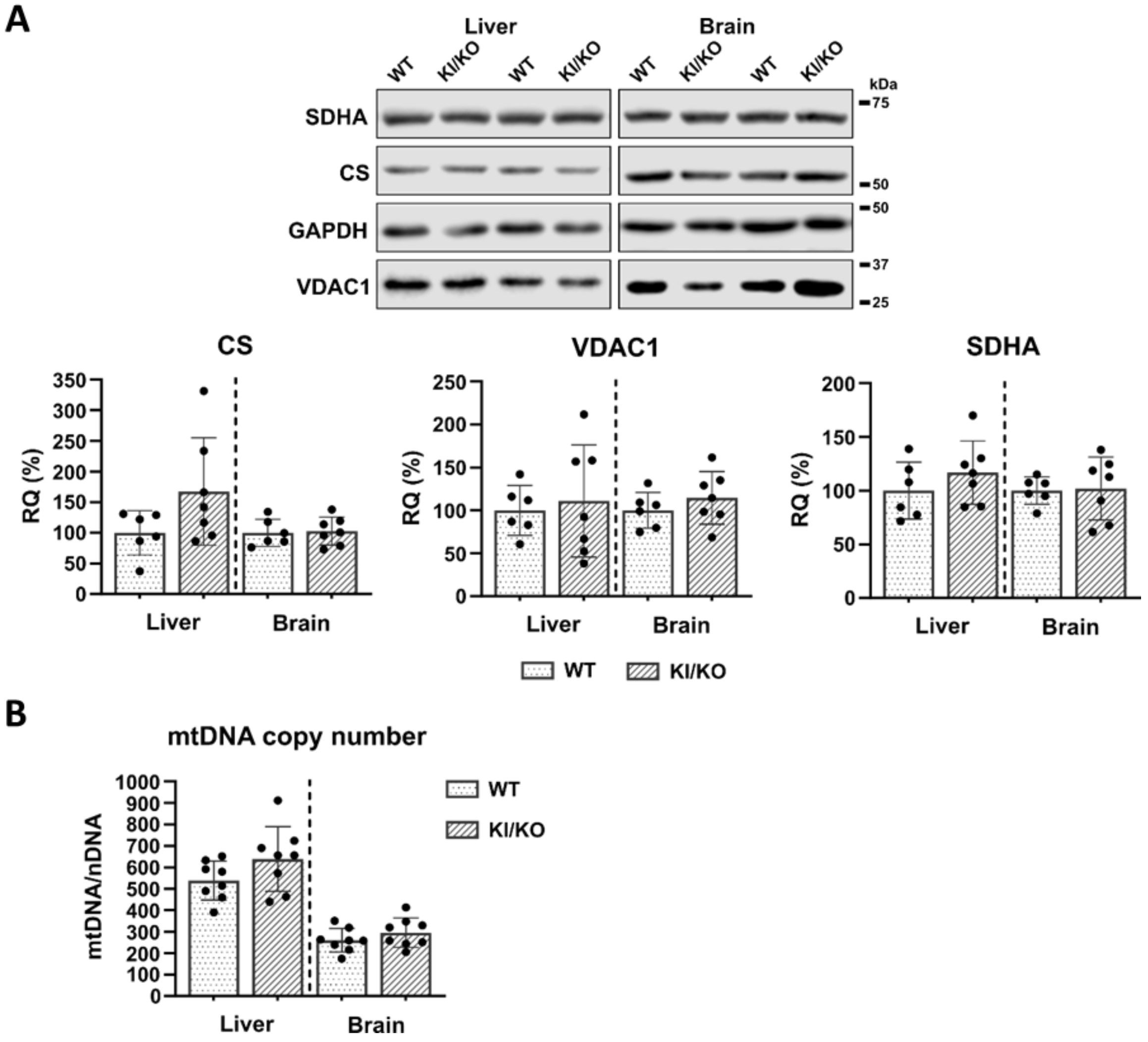

Appendix Figure S4. Mitochondrial content and mtDNA copy number in liver and brain from 8-week-old *Gfm1*$^{R671C/-}$ mice.

(A) SDS-PAGE western blots of SDHA (succinate dehydrogenase complex flavoprotein subunit A), CS (citrate synthase) and VDAC1 (voltage dependent anion channel 1), commonly used as markers of mitochondrial mass, using liver and brain total protein extracts. Densitometric quantifications of these proteins were corrected by GAPDH (as total protein control). Provided values are expressed as relative quantity (percentage of wild-type mean), and bars represent the mean relative quantity (±SD). **(B)** mtDNA copy number analysed by RT-qPCR using total DNA extracts from liver and brain. Specific *Taqman* probes were used to quantify the copy number of mitochondrial gene *mt-RNR2* (encoding 16S rRNA) and the nuclear gene *Ang1*, obtaining an equivalent ratio mtDNA/nDNA (±SD). RQ: relative quantification.

Appendix Table S1. Exact p-values and numbers of animals or replicates for each figure.

Figure 1. AAV9-hAAT-GFM1 transduction and hGFM1 expression in Gfm1R671C/- liver.

**Figure 1A - VCN**	
Females (n)	10
Males (n)	8
Mann Whitney test P value	<0.0001

**Figure 1B - GFM1 mRNA RQ**	
Females (n)	10
Males (n)	8
Mann Whitney test P value	<0.0001

**Figure 1C - EFG1 levels**			
**Females**		**Males**	
WT (n)	8	WT (n)	9
KI/KO V (n)	8	KI/KO V (n)	7
KI/KO AAV (n)	10	KI/KO AAV (n)	8
Kruskal-Wallis and Dunn's multiple comparisons test			
WT vs. KI/KO V - P value	<0.0001	WT vs. KI/KO V - P value	<0.0001
WT vs. KI/KO AAV - P value	0.0392	WT vs. KI/KO AAV - P value	0.0401
KI/KO V vs. KI/KO AAV - P value	0.0392	KI/KO V vs. KI/KO AAV - P value	0.1213
Mann Whitney test			
KI/KO V vs. KI/KO AAV - P value	<0.0001	KI/KO V vs. KI/KO AAV - P value	0.0003

**Figure 1D - Correlations**						
GFM1 mRNA vs. VCN	r Spearman=0.745	p=0.0004	Females (n)	10	Males (n)	8
EFG1 vs. VCN	r Spearman=0.829	p<0.0001	Females (n)	10	Males (n)	8
EFG1 vs. GFM1 mRNA	r Spearman=0.746	p=0.0004	Females (n)	10	Males (n)	8

Figure 2. AAV9P31-hSyn-GFM1 transduction and hGFM1 expression in Gfm1R671C/- brain.

**Figure 2A - VCN**					
Brain VCN		Liver VCN		rAAV transduction	
Females (n)	9	Females (n)	9	Brain	17
Males (n)	8	Males (n)	8	Liver	17
Mann Whitney test P value	0.9626	Mann Whitney test P value	0.0206	Mann Whitney test P value	<0.0001

**Figure 2B - GFM1 mRNA RQ in Brain**	
Females (n)	9
Males (n)	8
Mann Whitney test P value	0.3213

**Figure 2C - EFG1 levels**		
WT (n=16)	Females=8	Males=8
KI/KO V (n=16)	Females=8	Males=8
KI/KO AAV (n=17)	Females=9	Males=8
Kruskal-Wallis and Dunn's multiple comparisons test		
WT vs. KI/KO V - P value	<0.0001	
WT vs. KI/KO AAV - P value	0.0443	
KI/KO V vs. KI/KO AAV - P value	0.0006	
Mann Whitney test		
KI/KO V vs. KI/KO AAV - P value	<0.0001	

**Figure 2D - Correlations**						
GFM1 mRNA vs. VCN	r Spearman=0.706	p=0.0021	Females (n)	9	Males (n)	8
EFG1 vs. VCN	r Spearman=0.649	p=0.0059	Females (n)	9	Males (n)	8
EFG1 vs. GFM1 mRNA	r Spearman=0.564	p=0.0020	Females (n)	9	Males (n)	8

Figure 3. Levels of complex I and IV subunits in liver and brain mitochondria from rAAV-treated Gfm1R671C/- mice.

**Figure 3A - CI and CIV subunits in liver**			
**NDUFA9**			
**Females**		**Males**	
WT (n)	7	WT (n)	9
KI/KO V (n)	8	KI/KO V (n)	8
KI/KO AAV (n)	10	KI/KO AAV (n)	8
Kruskal-Wallis and Dunn's multiple comparisons test			
WT vs. KI/KO V - P value	0.0217	WT vs. KI/KO V - P value	0.1165
WT vs. KI/KO AAV - P value	>09999	WT vs. KI/KO AAV - P value	0.5918
KI/KO V vs. KI/KO AAV - P value	0.0359	KI/KO V vs. KI/KO AAV - P value	0.0033
Mann Whitney test			
KI/KO V vs. KI/KO AAV - P value	0.0085	KI/KO V vs. KI/KO AAV - P value	0,0019
**NDUFA10**			
**Females**		**Males**	
WT (n)	8	WT (n)	9
KI/KO V (n)	8	KI/KO V (n)	8
KI/KO AAV (n)	10	KI/KO AAV (n)	8
Kruskal-Wallis and Dunn's multiple comparisons test			
WT vs. KI/KO V - P value	0.0008	WT vs. KI/KO V - P value	0.0017
WT vs. KI/KO AAV - P value	>09999	WT vs. KI/KO AAV - P value	>09999
KI/KO V vs. KI/KO AAV - P value	0.0056	KI/KO V vs. KI/KO AAV - P value	0.0105
Mann Whitney test			
KI/KO V vs. KI/KO AAV - P value	0.0003	KI/KO V vs. KI/KO AAV - P value	0.0006
**COX1**			
**Females**		**Males**	
WT (n)	8	WT (n)	9
KI/KO V (n)	7	KI/KO V (n)	7
KI/KO AAV (n)	10	KI/KO AAV (n)	8
Kruskal-Wallis and Dunn's multiple comparisons test			
WT vs. KI/KO V - P value	0.0005	WT vs. KI/KO V - P value	0.0020
WT vs. KI/KO AAV - P value	>09999	WT vs. KI/KO AAV - P value	>09999
KI/KO V vs. KI/KO AAV - P value	0.0066	KI/KO V vs. KI/KO AAV - P value	0.0035
Mann Whitney test			
KI/KO V vs. KI/KO AAV - P value	0.0001	KI/KO V vs. KI/KO AAV - P value	0.0003
**COX2**			
**Females**		**Males**	
WT (n)	8	WT (n)	9
KI/KO V (n)	8	KI/KO V (n)	8
KI/KO AAV (n)	10	KI/KO AAV (n)	8
Kruskal-Wallis and Dunn's multiple comparisons test			
WT vs. KI/KO V - P value	<0.0001	WT vs. KI/KO V - P value	0.005
WT vs. KI/KO AAV - P value	0.2163	WT vs. KI/KO AAV - P value	>09999
KI/KO V vs. KI/KO AAV - P value	0.0161	KI/KO V vs. KI/KO AAV - P value	0.0060
Mann Whitney test			
KI/KO V vs. KI/KO AAV - P value	<0.0001	KI/KO V vs. KI/KO AAV - P value	0.0002

**Figure 3B - CI and CIV subunits in brain**		
**NDUFA9**		
WT (n=16)	Females=8	Males=8
KI/KO V (n=16)	Females=8	Males=8
KI/KO AAV (n=17)	Females=9	Males=8
Kruskal-Wallis and Dunn's multiple comparisons test		
WT vs. KI/KO V - P value	0.6481	
WT vs. KI/KO AAV - P value	>0.9999	
KI/KO V vs. KI/KO AAV - P value	>0.9999	
Mann Whitney test		
KI/KO V vs. KI/KO AAV - P value	0.7356	
**NDUFA10**		
WT (n=16)	Females=8	Males=8
KI/KO V (n=16)	Females=8	Males=8
KI/KO AAV (n=17)	Females=9	Males=8
Kruskal-Wallis and Dunn's multiple comparisons test		
WT vs. KI/KO V - P value	0.0090	
WT vs. KI/KO AAV - P value	0.5194	
KI/KO V vs. KI/KO AAV - P value	0.2956	
Mann Whitney test		
KI/KO V vs. KI/KO AAV - P value	0.0942	
**COX1**		
WT (n=16)	Females=8	Males=8
KI/KO V (n=16)	Females=8	Males=8
KI/KO AAV (n=17)	Females=9	Males=8
Kruskal-Wallis and Dunn's multiple comparisons test		
WT vs. KI/KO V - P value	0.0005	
WT vs. KI/KO AAV - P value	0.1167	
KI/KO V vs. KI/KO AAV - P value	0.2458	
Mann Whitney test		
KI/KO V vs. KI/KO AAV - P value	0.0531	
**COX2**		
WT (n=16)	Females=8	Males=8
KI/KO V (n=16)	Females=8	Males=8
KI/KO AAV (n=17)	Females=9	Males=8
Kruskal-Wallis and Dunn's multiple comparisons test		
WT vs. KI/KO V - P value	<0.0001	
WT vs. KI/KO AAV - P value	0.0260	
KI/KO V vs. KI/KO AAV - P value	0.1222	
Mann Whitney test		
KI/KO V vs. KI/KO AAV - P value	0.0187	

Figure 4. AAV9-hAAT-GFM1 effect on Gfm1R671C/- liver combined OXPHOS defect.

**Figure 4A - Complexes levels in liver**			
**CI**			
**Females**		**Males**	
WT (n)	6	WT (n)	7
KI/KO V (n)	6	KI/KO V (n)	6
KI/KO AAV (n)	7	KI/KO AAV (n)	6
Kruskal-Wallis and Dunn's multiple comparisons test			
WT vs. KI/KO V - P value	0.0168	WT vs. KI/KO V - P value	0.0147
WT vs. KI/KO AAV - P value	>09999	WT vs. KI/KO AAV - P value	>09999
KI/KO V vs. KI/KO AAV - P value	0.0636	KI/KO V vs. KI/KO AAV - P value	0.0549
Mann Whitney test			
KI/KO V vs. KI/KO AAV - P value	0.0140	KI/KO V vs. KI/KO AAV - P value	0.0152
**CII**			
**Females**		**Males**	
WT (n)	6	WT (n)	7
KI/KO V (n)	6	KI/KO V (n)	6
KI/KO AAV (n)	7	KI/KO AAV (n)	6
Kruskal-Wallis and Dunn's multiple comparisons test			
WT vs. KI/KO V - P value	>09999	WT vs. KI/KO V - P value	0.7908
WT vs. KI/KO AAV - P value	>09999	WT vs. KI/KO AAV - P value	>09999
KI/KO V vs. KI/KO AAV - P value	>09999	KI/KO V vs. KI/KO AAV - P value	>09999
Mann Whitney test			
KI/KO V vs. KI/KO AAV - P value	0.7308	KI/KO V vs. KI/KO AAV - P value	0.9372
**CIII**			
**Females**		**Males**	
WT (n)	6	WT (n)	7
KI/KO V (n)	6	KI/KO V (n)	6
KI/KO AAV (n)	6	KI/KO AAV (n)	6
Kruskal-Wallis and Dunn's multiple comparisons test			
WT vs. KI/KO V - P value	0.0799	WT vs. KI/KO V - P value	0.1035
WT vs. KI/KO AAV - P value	0.9127	WT vs. KI/KO AAV - P value	0.6798
KI/KO V vs. KI/KO AAV - P value	0.7026	KI/KO V vs. KI/KO AAV - P value	>09999
Mann Whitney test			
KI/KO V vs. KI/KO AAV - P value	0.1797	KI/KO V vs. KI/KO AAV - P value	0.3095
**CIV**			
**Females**		**Males**	
WT (n)	6	WT (n)	7
KI/KO V (n)	6	KI/KO V (n)	6
KI/KO AAV (n)	7	KI/KO AAV (n)	6
Kruskal-Wallis and Dunn's multiple comparisons test			
WT vs. KI/KO V - P value	0.0021	WT vs. KI/KO V - P value	0.0057
WT vs. KI/KO AAV - P value	>09999	WT vs. KI/KO AAV - P value	>09999
KI/KO V vs. KI/KO AAV - P value	0.0261	KI/KO V vs. KI/KO AAV - P value	0.0549
Mann Whitney test			
KI/KO V vs. KI/KO AAV - P value	0.0012	KI/KO V vs. KI/KO AAV - P value	0.0087

Figure 4B - Complexes activities in liver			
**CI Act**			
**Females**		**Males**	
WT (n)	8	WT (n)	8
KI/KO V (n)	8	KI/KO V (n)	8
KI/KO AAV (n)	10	KI/KO AAV (n)	8
Kruskal-Wallis and Dunn's multiple comparisons test			
WT vs. KI/KO V - P value	0.0511	WT vs. KI/KO V - P value	0.0011
WT vs. KI/KO AAV - P value	>09999	WT vs. KI/KO AAV - P value	0.8180
KI/KO V vs. KI/KO AAV - P value	0.0187	KI/KO V vs. KI/KO AAV - P value	0.0397
Mann Whitney test			
KI/KO V vs. KI/KO AAV - P value	0.0117	KI/KO V vs. KI/KO AAV - P value	0.0016
**CIV Act**			
**Females**		**Males**	
WT (n)	8	WT (n)	8
KI/KO V (n)	8	KI/KO V (n)	8
KI/KO AAV (n)	10	KI/KO AAV (n)	8
Kruskal-Wallis and Dunn's multiple comparisons test			
WT vs. KI/KO V - P value	0.0004	WT vs. KI/KO V - P value	0.0011
WT vs. KI/KO AAV - P value	0.0780	WT vs. KI/KO AAV - P value	0.5725
KI/KO V vs. KI/KO AAV - P value	0.2049	KI/KO V vs. KI/KO AAV - P value	0.0710
Mann Whitney test			
KI/KO V vs. KI/KO AAV - P value	0.0276	KI/KO V vs. KI/KO AAV - P value	0.0047
**CS Act**			
**Females**		**Males**	
WT (n)	8	WT (n)	8
KI/KO V (n)	8	KI/KO V (n)	8
KI/KO AAV (n)	10	KI/KO AAV (n)	8
Kruskal-Wallis and Dunn's multiple comparisons test			
WT vs. KI/KO V - P value	>09999	WT vs. KI/KO V - P value	0.8665
WT vs. KI/KO AAV - P value	>09999	WT vs. KI/KO AAV - P value	0.7300
KI/KO V vs. KI/KO AAV - P value	>09999	KI/KO V vs. KI/KO AAV - P value	0.0778
Mann Whitney test			
KI/KO V vs. KI/KO AAV - P value	0.5726	KI/KO V vs. KI/KO AAV - P value	0.0650

Figure 5. AAV9P31-hSyn-GFM1 effect on Gfm1R671C/- brain combined OXPHOS defect.

**Figure 5A - Complexes levels in brain**		
**CI**		
WT (n=16)	Females=8	Males=8
KI/KO V (n=15)	Females=8	Males=7
KI/KO AAV (n=17)	Females=9	Males=8
Kruskal-Wallis and Dunn's multiple comparisons test		
WT vs. KI/KO V - P value	0.0044	
WT vs. KI/KO AAV - P value	0.0306	
KI/KO V vs. KI/KO AAV - P value	>0.9999	
Mann Whitney test		
KI/KO V vs. KI/KO AAV - P value	0.3126	
**CII**		
WT (n=16)	Females=8	Males=8
KI/KO V (n=15)	Females=8	Males=7
KI/KO AAV (n=17)	Females=9	Males=8
Kruskal-Wallis and Dunn's multiple comparisons test		
WT vs. KI/KO V - P value	0.0199	
WT vs. KI/KO AAV - P value	0.3175	
KI/KO V vs. KI/KO AAV - P value	0.7323	
Mann Whitney test		
KI/KO V vs. KI/KO AAV - P value	0.2055	
**CIII**		
WT (n=16)	Females=8	Males=8
KI/KO V (n=15)	Females=8	Males=7
KI/KO AAV (n=17)	Females=9	Males=8
Kruskal-Wallis and Dunn's multiple comparisons test		
WT vs. KI/KO V - P value	0.0319	
WT vs. KI/KO AAV - P value	0.3661	
KI/KO V vs. KI/KO AAV - P value	0.8519	
Mann Whitney test		
KI/KO V vs. KI/KO AAV - P value	0.2455	
**CIV**		
WT (n=16)	Females=8	Males=8
KI/KO V (n=15)	Females=8	Males=7
KI/KO AAV (n=17)	Females=9	Males=8
Kruskal-Wallis and Dunn's multiple comparisons test		
WT vs. KI/KO V - P value	0.0005	
WT vs. KI/KO AAV - P value	0.0276	
KI/KO V vs. KI/KO AAV - P value	0.6115	
Mann Whitney test		
KI/KO V vs. KI/KO AAV - P value	0.0894	

**Figure 5B - Complexes activities in brain**		
**CI**		
WT (n=16)	Females=8	Males=8
KI/KO V (n=16)	Females=8	Males=8
KI/KO AAV (n=17)	Females=9	Males=8
Kruskal-Wallis and Dunn's multiple comparisons test		
WT vs. KI/KO V - P value	0.0059	
WT vs. KI/KO AAV - P value	>0.9999	
KI/KO V vs. KI/KO AAV - P value	0.0076	
Mann Whitney test		
KI/KO V vs. KI/KO AAV - P value	0.0008	
**CIV**		
WT (n=16)	Females=8	Males=8
KI/KO V (n=16)	Females=8	Males=8
KI/KO AAV (n=17)	Females=9	Males=8
Kruskal-Wallis and Dunn's multiple comparisons test		
WT vs. KI/KO V - P value	<0.0001	
WT vs. KI/KO AAV - P value	0.0499	
KI/KO V vs. KI/KO AAV - P value	0.0140	
Mann Whitney test		
KI/KO V vs. KI/KO AAV - P value	0.0003	
**CS**		
WT (n=16)	Females=8	Males=8
KI/KO V (n=16)	Females=8	Males=8
KI/KO AAV (n=17)	Females=9	Males=8
Kruskal-Wallis and Dunn's multiple comparisons test		
WT vs. KI/KO V - P value	0.0718	
WT vs. KI/KO AAV - P value	0.8369	
KI/KO V vs. KI/KO AAV - P value	0.6796	
Mann Whitney test		
KI/KO V vs. KI/KO AAV - P value	0.3134	

Figure 6. Mitoriboprotein levels in liver and brain mitochondria from rAAV treated Gfm1R671C/- mice.

**Figure 6A - MRPs in liver**			
**MRPS9**			
**Females**		**Males**	
WT (n)	8	WT (n)	9
KI/KO V (n)	7	KI/KO V (n)	8
KI/KO AAV (n)	9	KI/KO AAV (n)	8
Kruskal-Wallis and Dunn's multiple comparisons test			
WT vs. KI/KO V - P value	0.0308	WT vs. KI/KO V - P value	0.0012
WT vs. KI/KO AAV - P value	>09999	WT vs. KI/KO AAV - P value	>09999
KI/KO V vs. KI/KO AAV - P value	0.0725	KI/KO V vs. KI/KO AAV - P value	0.0280
Mann Whitney test			
KI/KO V vs. KI/KO AAV - P value	0.0085	KI/KO V vs. KI/KO AAV - P value	0.0030
**MRPS35**			
**Females**		**Males**	
WT (n)	8	WT (n)	9
KI/KO V (n)	8	KI/KO V (n)	8
KI/KO AAV (n)	10	KI/KO AAV (n)	8
Kruskal-Wallis and Dunn's multiple comparisons test			
WT vs. KI/KO V - P value	0.0023	WT vs. KI/KO V - P value	0.0057
WT vs. KI/KO AAV - P value	0.1689	WT vs. KI/KO AAV - P value	0.5222
KI/KO V vs. KI/KO AAV - P value	0.3030	KI/KO V vs. KI/KO AAV - P value	0.2683
Mann Whitney test			
KI/KO V vs. KI/KO AAV - P value	0.0343	KI/KO V vs. KI/KO AAV - P value	0.0830
**MRPL13**			
**Females**		**Males**	
WT (n)	8	WT (n)	9
KI/KO V (n)	8	KI/KO V (n)	8
KI/KO AAV (n)	10	KI/KO AAV (n)	8
Kruskal-Wallis and Dunn's multiple comparisons test			
WT vs. KI/KO V - P value	0.0023	WT vs. KI/KO V - P value	0.0090
WT vs. KI/KO AAV - P value	0.1370	WT vs. KI/KO AAV - P value	>09999
KI/KO V vs. KI/KO AAV - P value	0.3631	KI/KO V vs. KI/KO AAV - P value	0.0686
Mann Whitney test			
KI/KO V vs. KI/KO AAV - P value	0.0434	KI/KO V vs. KI/KO AAV - P value	0.0148
**MRPL37**			
**Females**		**Males**	
WT (n)	8	WT (n)	9
KI/KO V (n)	8	KI/KO V (n)	8
KI/KO AAV (n)	9	KI/KO AAV (n)	8
Kruskal-Wallis and Dunn's multiple comparisons test			
WT vs. KI/KO V - P value	0.2881	WT vs. KI/KO V - P value	0.0058
WT vs. KI/KO AAV - P value	0.8152	WT vs. KI/KO AAV - P value	0.3554
KI/KO V vs. KI/KO AAV - P value	>09999	KI/KO V vs. KI/KO AAV - P value	0.4051
Mann Whitney test			
KI/KO V vs. KI/KO AAV - P value	0.4807	KI/KO V vs. KI/KO AAV - P value	0.1304

Figure 6B - MRPs in brain		
**MRPS9**		
WT (n=16)	Females=8	Males=8
KI/KO V (n=16)	Females=8	Males=8
KI/KO AAV (n=17)	Females=9	Males=8
Kruskal-Wallis and Dunn's multiple comparisons test		
WT vs. KI/KO V - P value	<0.0001	
WT vs. KI/KO AAV - P value	0.0006	
KI/KO V vs. KI/KO AAV - P value	0.1599	
Mann Whitney test		
KI/KO V vs. KI/KO AAV - P value	0.0069	
**MRPS35**		
WT (n=16)	Females=8	Males=8
KI/KO V (n=16)	Females=8	Males=8
KI/KO AAV (n=17)	Females=9	Males=8
Kruskal-Wallis and Dunn's multiple comparisons test		
WT vs. KI/KO V - P value	<0.0001	
WT vs. KI/KO AAV - P value	<0.0001	
KI/KO V vs. KI/KO AAV - P value	0.6578	
Mann Whitney test		
KI/KO V vs. KI/KO AAV - P value	0.0942	
**MRPL13**		
WT (n=16)	Females=8	Males=8
KI/KO V (n=16)	Females=8	Males=8
KI/KO AAV (n=17)	Females=9	Males=8
Kruskal-Wallis and Dunn's multiple comparisons test		
WT vs. KI/KO V - P value	<0.0001	
WT vs. KI/KO AAV - P value	0.0006	
KI/KO V vs. KI/KO AAV - P value	0.1125	
Mann Whitney test		
KI/KO V vs. KI/KO AAV - P value	0.0028	
**MRPL37**		
WT (n=16)	Females=8	Males=8
KI/KO V (n=16)	Females=8	Males=8
KI/KO AAV (n=17)	Females=9	Males=8
Kruskal-Wallis and Dunn's multiple comparisons test		
WT vs. KI/KO V - P value	<0.0001	
WT vs. KI/KO AAV - P value	0.0024	
KI/KO V vs. KI/KO AAV - P value	0.0117	
Mann Whitney test		
KI/KO V vs. KI/KO AAV - P value	<0.0001	

Figure 7. Mitochondrial RNA levels in liver and brain from rAAV treated Gfm1R671C/- mice.

**Figure 7A - mtRNA levels in liver**			
**Nd4**			
**Females**		**Males**	
WT (n)	8	WT (n)	7
KI/KO V (n)	8	KI/KO V (n)	8
KI/KO AAV (n)	10	KI/KO AAV (n)	8
Kruskal-Wallis and Dunn's multiple comparisons test			
WT vs. KI/KO V - P value	0.0036	WT vs. KI/KO V - P value	0.0050
WT vs. KI/KO AAV - P value	0.0107	WT vs. KI/KO AAV - P value	0.0143
KI/KO V vs. KI/KO AAV - P value	>09999	KI/KO V vs. KI/KO AAV - P value	>09999
Mann Whitney test			
KI/KO V vs. KI/KO AAV - P value	0.6334	KI/KO V vs. KI/KO AAV - P value	0.8785
**Cox1**			
**Females**		**Males**	
WT (n)	8	WT (n)	7
KI/KO V (n)	8	KI/KO V (n)	8
KI/KO AAV (n)	10	KI/KO AAV (n)	8
Kruskal-Wallis and Dunn's multiple comparisons test			
WT vs. KI/KO V - P value	0.0023	WT vs. KI/KO V - P value	0.0301
WT vs. KI/KO AAV - P value	0.0114	WT vs. KI/KO AAV - P value	0.2377
KI/KO V vs. KI/KO AAV - P value	>09999	KI/KO V vs. KI/KO AAV - P value	>09999
Mann Whitney test			
KI/KO V vs. KI/KO AAV - P value	0.5726	KI/KO V vs. KI/KO AAV - P value	0.3823
**12S**			
**Females**		**Males**	
WT (n)	8	WT (n)	7
KI/KO V (n)	8	KI/KO V (n)	8
KI/KO AAV (n)	10	KI/KO AAV (n)	8
Kruskal-Wallis and Dunn's multiple comparisons test			
WT vs. KI/KO V - P value	<0.0001	WT vs. KI/KO V - P value	0.0022
WT vs. KI/KO AAV - P value	0.0513	WT vs. KI/KO AAV - P value	0.1004
KI/KO V vs. KI/KO AAV - P value	0.0401	KI/KO V vs. KI/KO AAV - P value	0.5910
Mann Whitney test			
KI/KO V vs. KI/KO AAV - P value	<0.0001	KI/KO V vs. KI/KO AAV - P value	0.1949
**16S**			
**Females**		**Males**	
WT (n)	8	WT (n)	7
KI/KO V (n)	8	KI/KO V (n)	8
KI/KO AAV (n)	10	KI/KO AAV (n)	7
Kruskal-Wallis and Dunn's multiple comparisons test			
WT vs. KI/KO V - P value	<0.0001	WT vs. KI/KO V - P value	0.0022
WT vs. KI/KO AAV - P value	0.0124	WT vs. KI/KO AAV - P value	0.1750
KI/KO V vs. KI/KO AAV - P value	0.3206	KI/KO V vs. KI/KO AAV - P value	0.4680
Mann Whitney test			
KI/KO V vs. KI/KO AAV - P value	0.0205	KI/KO V vs. KI/KO AAV - P value	0.1206

Figure 7B - mtRNA levels in brain		
**Nd4**		
WT (n=16)	Females=8	Males=8
KI/KO V (n=17)	Females=9	Males=8
KI/KO AAV (n=16)	Females=8	Males=8
Kruskal-Wallis and Dunn's multiple comparisons test		
WT vs. KI/KO V - P value	<0.0001	
WT vs. KI/KO AAV - P value	0.0002	
KI/KO V vs. KI/KO AAV - P value	0.2315	
Mann Whitney test		
KI/KO V vs. KI/KO AAV - P value	0.0079	
**Cox1**		
WT (n=16)	Females=8	Males=8
KI/KO V (n=17)	Females=9	Males=8
KI/KO AAV (n=16)	Females=8	Males=8
Kruskal-Wallis and Dunn's multiple comparisons test		
WT vs. KI/KO V - P value	<0.0001	
WT vs. KI/KO AAV - P value	0.0001	
KI/KO V vs. KI/KO AAV - P value	0.4104	
Mann Whitney test		
KI/KO V vs. KI/KO AAV - P value	0.0302	
**12S**		
WT (n=16)	Females=8	Males=8
KI/KO V (n=17)	Females=9	Males=8
KI/KO AAV (n=16)	Females=8	Males=8
Kruskal-Wallis and Dunn's multiple comparisons test		
WT vs. KI/KO V - P value	<0.0001	
WT vs. KI/KO AAV - P value	0.0004	
KI/KO V vs. KI/KO AAV - P value	0.1449	
Mann Whitney test		
KI/KO V vs. KI/KO AAV - P value	0.0028	
**16S**		
WT (n=16)	Females=8	Males=8
KI/KO V (n=17)	Females=9	Males=8
KI/KO AAV (n=16)	Females=8	Males=8
Kruskal-Wallis and Dunn's multiple comparisons test		
WT vs. KI/KO V - P value	<0.0001	
WT vs. KI/KO AAV - P value	0.0017	
KI/KO V vs. KI/KO AAV - P value	0.0164	
Mann Whitney test		
KI/KO V vs. KI/KO AAV - P value	<0.0001	

EV1. Liver and brain COXPD molecular phenotype in 30-week-old Gfm1R671C/- mice.

**EV1A - EFG1 Levels**			
**Liver**		**Brain**	
WT (n)	6	WT (n)	6
KI/KO (n)	7	KI/KO (n)	7
Mann Whitney test			
WT vs. KI/KO - P value	0.0012	WT vs. KI/KO - P value	0.0012

**EV1C - NDUFA9 and COX2 levels**			
**NDUFA9**			
**Liver**		**Brain**	
WT (n)	7	WT (n)	6
KI/KO (n)	7	KI/KO (n)	6
Mann Whitney test			
WT vs. KI/KO - P value	0.0070	WT vs. KI/KO - P value	0.0012
**COX2**			
**Liver**		**Brain**	
WT (n)	6	WT (n)	6
KI/KO (n)	7	KI/KO (n)	6
Mann Whitney test			
WT vs. KI/KO - P value	0.0012	WT vs. KI/KO - P value	0.0022

**EV1D - CI and CIV levels**			
**CI**			
**Liver**		**Brain**	
WT (n)	7	WT (n)	5
KI/KO (n)	7	KI/KO (n)	6
Mann Whitney test			
WT vs. KI/KO - P value	0.0262	WT vs. KI/KO - P value	0.2468
**CIV**			
**Liver**		**Brain**	
WT (n)	7	WT (n)	5
KI/KO (n)	7	KI/KO (n)	6
Mann Whitney test			
WT vs. KI/KO - P value	0.0006	WT vs. KI/KO - P value	0.0173

**EV1E - CI and CIV activities**			
**CI**			
**Liver**		**Brain**	
WT (n)	7	WT (n)	7
KI/KO (n)	8	KI/KO (n)	8
Mann Whitney test			
WT vs. KI/KO - P value	0.0002	WT vs. KI/KO - P value	0.0016
**CIV**			
**Liver**		**Brain**	
WT (n)	7	WT (n)	7
KI/KO (n)	7	KI/KO (n)	8
Mann Whitney test			
WT vs. KI/KO - P value	0.0003	WT vs. KI/KO - P value	0.0059

EV2. Lentiviral gene therapy rescues EFG1 depletion and corrects OXPHOS deficiency in patients' fibroblasts.

**EV2A - EFG1 levels**				
**Group**	**n**			
C1	3		Mann Whitney test	
C2	3		Groups compared	P - value
**Patient 1**			**Patient 1**	
NT	3		NT vs. G10	0.1000
pSHAM	3		NT vs. G50	0.1000
GFM1 G10	3		pSHAM vs. G10	0.1000
GFM1 G50	3		pSHAM vs. G50	0.1000
**Patient 2**			**Patient 2**	
NT	3		NT vs. G10	0.1000
pSHAM	3		NT vs. G50	0.1000
GFM1 G10	3		pSHAM vs. G10	0.1000
GFM1 G50	3		pSHAM vs. G50	0.1000
**Patient 3**			**Patient 3**	
NT	3		NT vs. G10	0.1000
pSHAM	3		NT vs. G50	0.1000
GFM1 G10	3		pSHAM vs. G10	0.1000
GFM1 G50	3		pSHAM vs. G50	0.1000

**EV2B**				
**CI Activity**				
**Group**	**n**			
C1	4		Mann Whitney test	
C2	4		Groups compared	P - value
**Patient 1**			**Patient 1**	
NT	3		NT vs. G10	0.1000
pSHAM	3		NT vs. G50	0.1000
GFM1 G10	3		pSHAM vs. G10	0.1000
GFM1 G50	3		pSHAM vs. G50	0.1000
**Patient 2**			**Patient 2**	
NT	3		NT vs. G10	0.1000
pSHAM	3		NT vs. G50	0.1000
GFM1 G10	3		pSHAM vs. G10	0.1000
GFM1 G50	3		pSHAM vs. G50	0.1000
**Patient 3**			**Patient 3**	
NT	3		NT vs. G10	0.1000
pSHAM	3		NT vs. G50	0.1000
GFM1 G10	3		pSHAM vs. G10	0.1000
GFM1 G50	3		pSHAM vs. G50	0.1000
**CIV Activity**				
**Group**	**n**			
C1	3		Mann Whitney test	
C2	3		Groups compared	P - value
**Patient 1**			**Patient 1**	
NT	3		NT vs. G10	0.1000
pSHAM	3		NT vs. G50	0.1000
GFM1 G10	3		pSHAM vs. G10	0.1000
GFM1 G50	3		pSHAM vs. G50	0.1000
**Patient 2**			**Patient 2**	
NT	3		NT vs. G10	0.1000
pSHAM	3		NT vs. G50	0.1000
GFM1 G10	3		pSHAM vs. G10	0.1000
GFM1 G50	3		pSHAM vs. G50	0.1000
**Patient 3**			**Patient 3**	
NT	3		NT vs. G10	0.1000
pSHAM	3		NT vs. G50	0.1000
GFM1 G10	3		pSHAM vs. G10	0.1000
GFM1 G50	3		pSHAM vs. G50	0.1000

EV4. VCN in non target tissues

AAV9-hAAT-GFM1	
**Tissue**	**n samples**
Liver	2
Spleen	4
Heart	4
Gastrocnemius	4

AAV9P31-hSyn-GFM1	
**Tissue**	**n samples**
Brain	2
Spleen	4
Heart	4
Gastrocnemius	4

EV5. Correlations between mitochondrial levels of EFG1 and complex CIV subunits.

**EV5A - AAV9-hAAT-GFM1**						
EFG1 vs. COX1	r Spearman=0.929	p<0.0001	Females (n)	10	Males (n)	8
EFG1 vs. COX2	r Spearman=0.463	p=0.053	Females (n)	10	Males (n)	8

**EV5B - AAV9P31-hSyn-GFM1**						
EFG1 vs. COX1	r Spearman=0.313	p=0.219	Females (n)	9	Males (n)	8
EFG1 vs. COX2	r Spearman=0.480	p=0.053	Females (n)	9	Males (n)	8
