# Supplementary material for: Systemic delivery of AAV-GFM1 corrects COXPD1 molecular alterations in Gfm1R671C/− mice
Source: EMBO Mol Med. 2026 Apr 17;18(6):2152–79. doi: 10.1038/s44321-026-00426-4 (PMC13269562; doi:10.1038/s44321-026-00426-4)
Supplement: Supplementary file 15 — Expanded View Figures [file 44321_2026_426_MOESM15_ESM.pdf]

Expanded View Figures

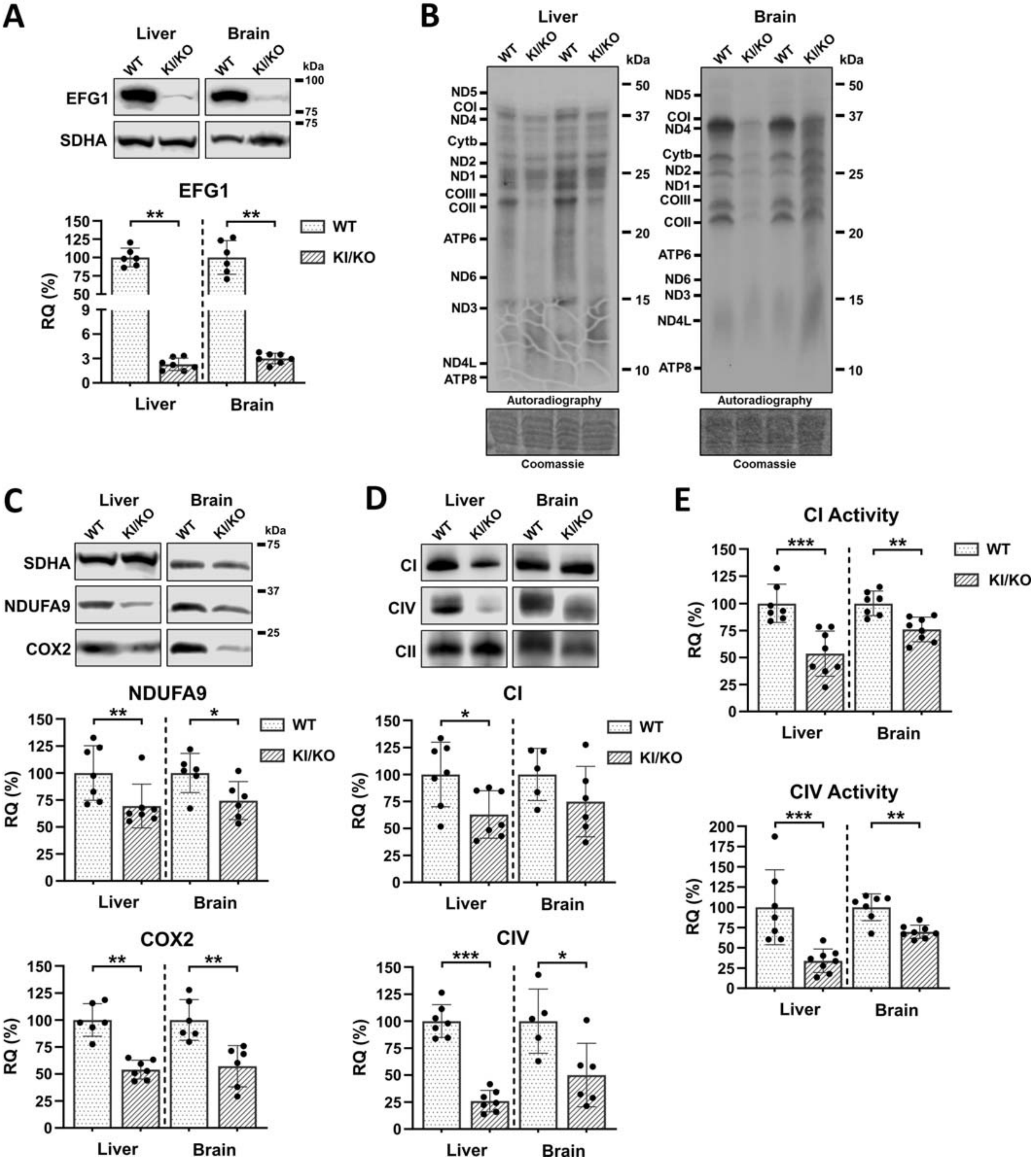

© The Author(s)

Figure EV1. Liver and brain COXPD molecular phenotype in 30-week-old *Gfm1*$^{R671C/-}$ mice.

(**A**) Mitochondrial EFG1 immunodetection in liver and brain by western blot SDS-PAGE. EFG1 protein levels were normalized to SDHA levels (used as mitochondrial loading control). (**B**) In organello mitochondrial translation performed using fresh liver and brain mitochondria. De novo synthesized proteins were radiolabelled with ^{35}S and detected through autoradiography. A Coomassie-stained electrophoresis gel was used as a protein loading control. (**C**) Western blot SDS-PAGE of NDUFA9 (CI subunit, nDNA encoded) and COX2 (CIV subunit, mtDNA encoded) in liver and brain mitochondria. Protein levels were normalized to SDHA levels. (**D**) Assembled complex I and IV levels analyzed by western blot BN-PAGE on liver and brain mitochondria. CII levels were used as mitochondrial protein loading control. (**E**) Spectrophotometric determination of complex I and IV enzyme activities, normalized to citrate synthase activity. Dots represent results for each mouse, and relative quantifications (RQ) are expressed as a percentage of the wild-type mean; bars represent the mean RQ (± SD). The exact *n* for each experiment is indicated in Appendix Table S1. Asterisks indicate statistical differences between the WT and KI/KO groups (*$P < 0.05$, **$P < 0.01$, ***$P < 0.001$, Mann–Whitney *U* test). RQ relative quantification. Source data are available online for this figure.

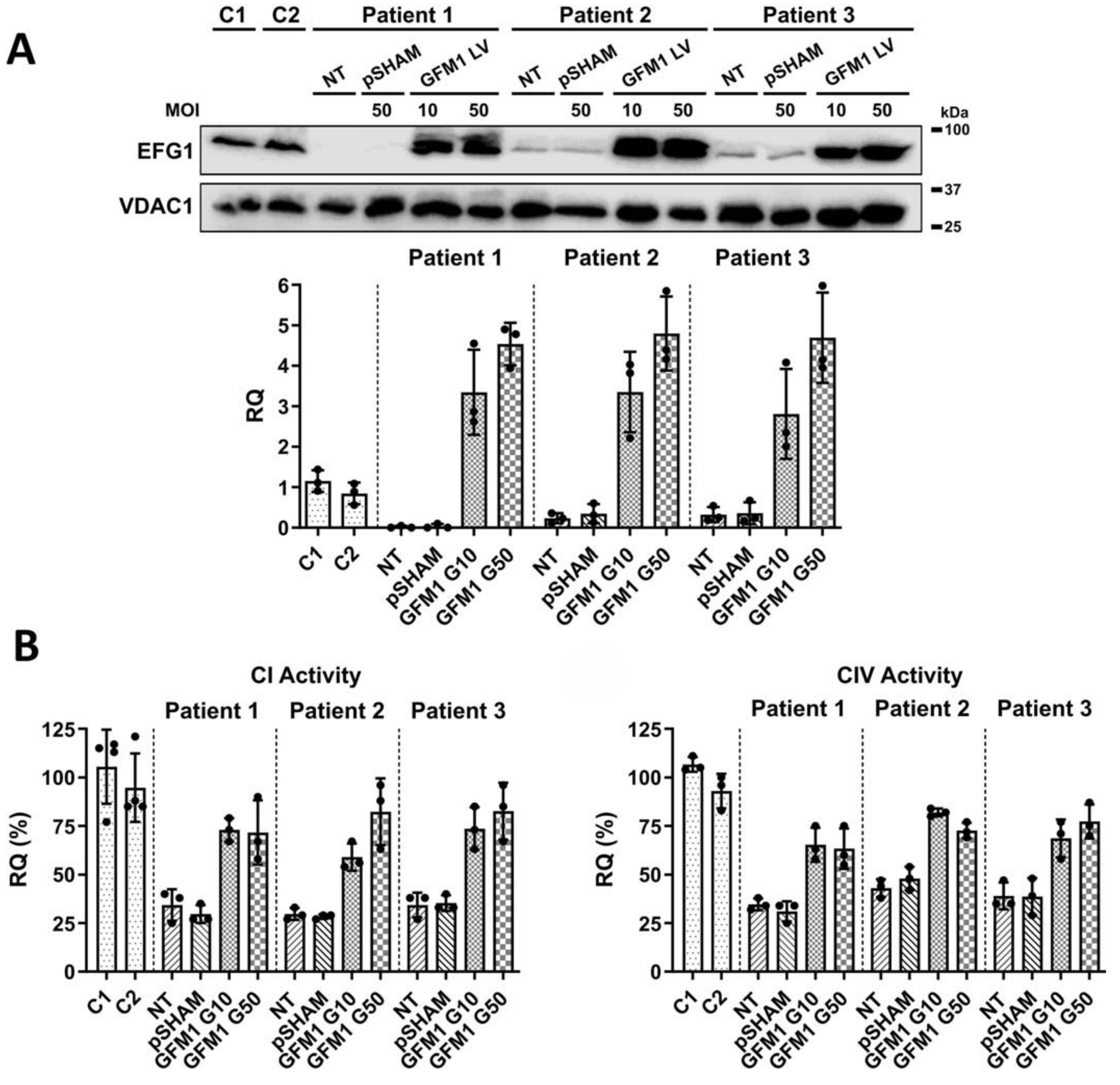

Figure EV2. Lentiviral gene therapy rescues EFG1 depletion and corrects OXPHOS deficiency in patients' fibroblasts.

(**A**) Western blot analysis of EFG1 protein levels after lentiviral transduction. EFG1 protein relative amount in the three patients' fibroblasts after transduction with the pSham LV at a MOI 50 or with the p305-GFM1LV at a MOI 10 (G10) or MOI 50 (G50), in comparison with their corresponding non-transduced cell lines (NT) or two healthy controls (C1 and C2). Results are expressed as the mean ± SD of three experiments carried with the same homogenate. All the values are normalized to VDAC1 and referred to the control protein levels mean (C1 and C2). (**B**) Enzyme activity determination of RC complexes CI and CIV. Relative activities in fibroblasts from C1 and C2 and in fibroblasts from P1, P2, and P3 non-treated (NT), transduced with pSham LV MOI 50 (pSham), or transduced with p305-GFM1LV at a MOI 10 (G10) or 50 (G50). Relative activity is defined as CI activity normalized to CS activity and referred to controls CI/CS activity mean. Bars represent the mean ± SD. RQ relative quantification. Three experimental replicates are depicted for each sample/condition, except for CI enzyme activity determination in C1 and C2, in which four experimental replicates are depicted. Source data are available online for this figure.

 © The Author(s)

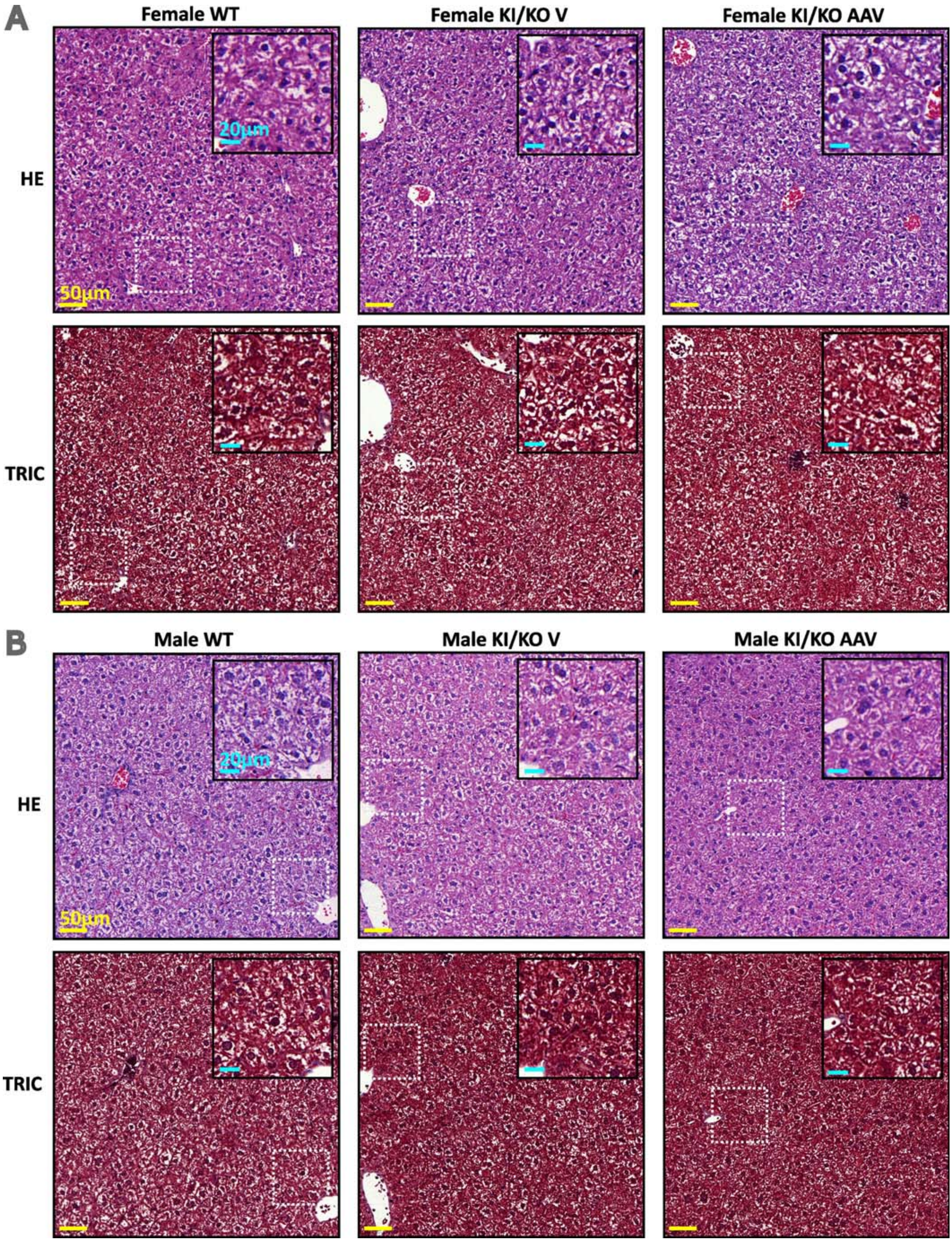
A
Female WT
Female KI/KO V
Female KI/KO AAV
HE
TRIC
20µm
50µm
B
Male WT
Male KI/KO V
Male KI/KO AAV
HE
TRIC
20µm
50µm

Figure EV3. Mouse liver histology of the AAV9-hAAT-GFM1 study.

Representative images from formalin-fixed and paraffin-embedded livers of 10-week-old female (**A**) and male (**B**) mice subjected to hematoxylin–eosin staining (HE) and Masson trichrome staining (TRIC). Zoom-out bar: 50 µm. Zoom-in bar: 20 µm. WT wild-type mice, KI/KO V *Gfm1*$^{R671C/-}$ mice treated with vehicle, KI/KO AAV *Gfm1*$^{R671C/-}$ mice treated with the therapeutic vector. Some selected areas (dotted white boxes) are magnified at the upper right corners of every panel. Source data are available online for this figure.

 © The Author(s)

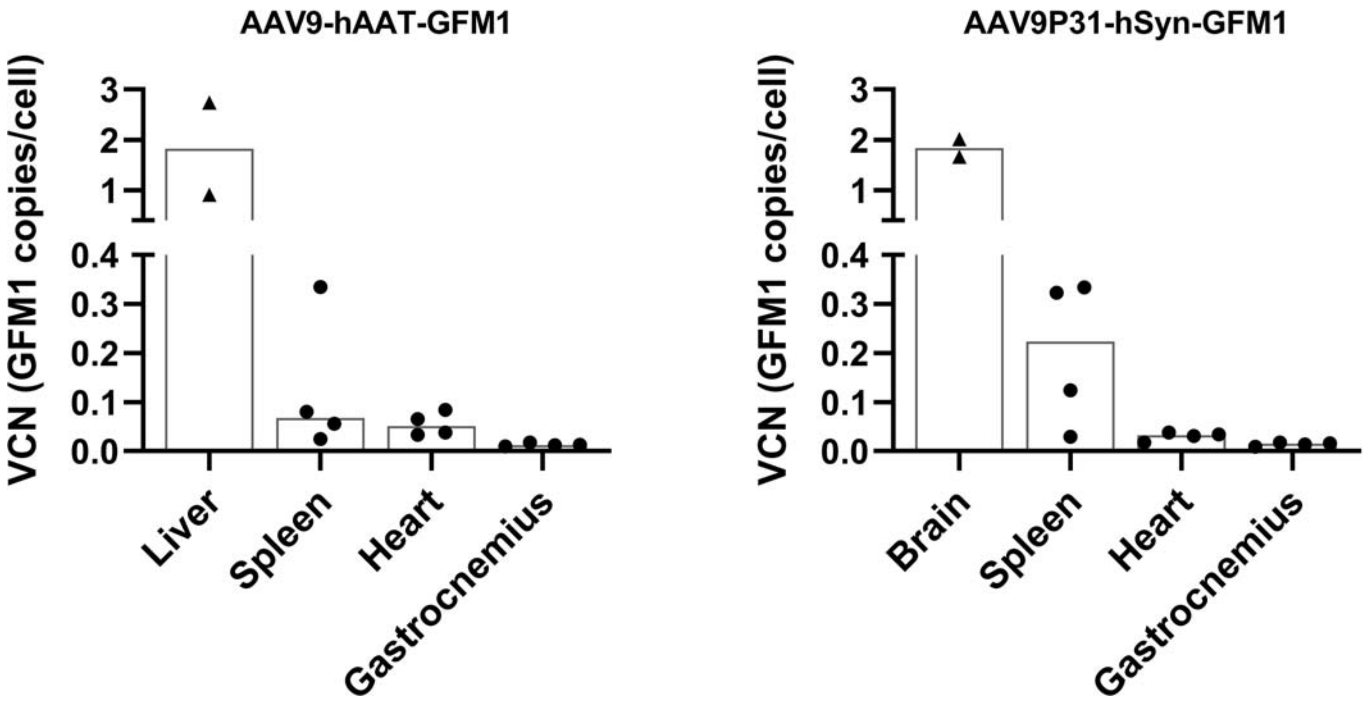

Figure EV4. Biodistribution of AAV9-hAAT-GFM1 and AAV9P31-hSyn-GFM1 in non-target tissues.

Transduction levels observed in *Gfm1*$^{R671C/-}$ mice treated with AAV9-hAAT-GFM1 (left) and AAV9P31-hSyn-GFM1 (right) in three non-target tissues ($n = 4$ animals for each treatment), as compared with the levels observed in the corresponding target tissues for each vector (liver $n = 2$, and brain $n = 2$, respectively) in two animals. Vector copy number (VCN) was analyzed by RT-qPCR using a specific human *GFM1* cDNA probe. Source data are available online for this figure.

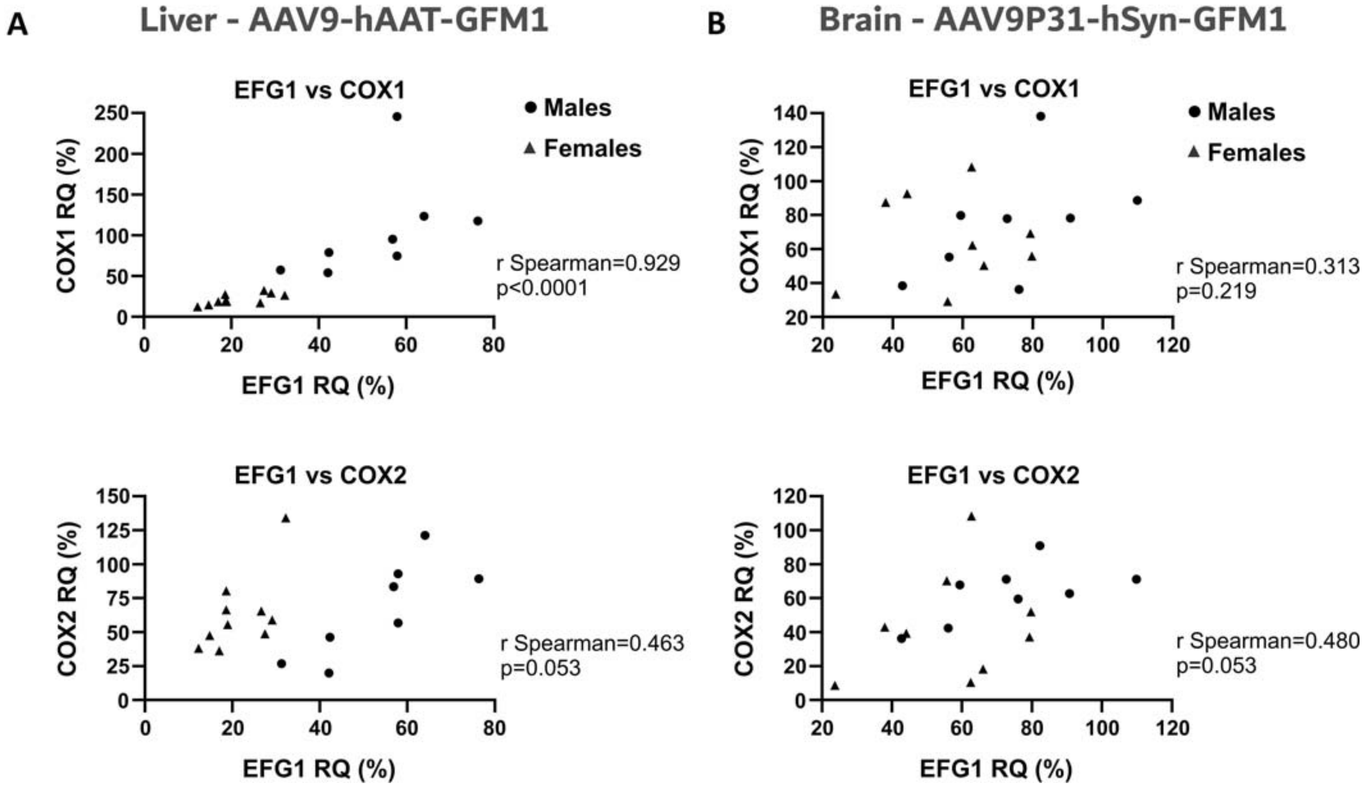

Figure EV5. Correlations between mitochondrial levels of EFG1 and complex CIV subunits.

Correlation comparing mitochondrial levels of EFG1 and mtDNA encoded CIV subunits (COX1 and COX2) in *Gfm1*$^{R671C/-}$ mice samples from (**A**) livers treated with AAV9-hAAT-GFM1 (females $n = 10$, males $n = 8$) and (**B**) brains treated with AAV9P31-hSyn-GFM1 (females $n = 9$ and males $n = 8$). Analysis was performed considering EFG1, COX1, and COX2 protein levels (RQ, % of WT mean values) in mitochondria from target tissues of KI/KO AAV females (triangles) and males (circles). The Spearman statistical test was applied to each comparison. RQ relative quantification. Source data are available online for this figure.

 © The Author(s)
