# Supplementary material for: Systemic delivery of AAV-GFM1 corrects COXPD1 molecular alterations in Gfm1R671C/− mice
Source: EMBO Mol Med. 2026 Apr 17;18(6):2152–79. doi: 10.1038/s44321-026-00426-4 (PMC13269562; doi:10.1038/s44321-026-00426-4)
Supplement: Supplementary file 1 — Peer Review File [file 44321_2026_426_MOESM1_ESM.pdf]

EMBO Molecular Medicine

Systemic delivery of AAV-GFM1 corrects COXPD1 molecular alterations in Gfm1R671C/- mice

Miguel Molina-Berenguer, Diego Herrero-Martínez, Antoni Vallbona-Garcia, Ferran Vila-Julià, Yolanda Cámara, Africa Vales, Gloria Gonzalez-Aseguinolaza, Javier Torres-Torronteras, and Ramon Marti

Corresponding author: Ramon Marti (ramon.marti@vhir.org)

Review Timeline:

Submission Date:	3rd Oct 25
Editorial Decision:	5th Nov 25
Revision Received:	18th Feb 26
Editorial Decision:	18th Mar 26
Revision Received:	30th Mar 26
Accepted:	1st Apr 26

Editor: Zeljko Durdevic

Transaction Report:

(Note: With the exception of the correction of typographical or spelling errors that could be a source of ambiguity, letters and reports are not edited. Depending on transfer agreements, referee reports obtained elsewhere may or may not be included in this compilation. Referee reports are anonymous unless the Referee chooses to sign their reports.)

5th Nov 2025

Dear Dr. Marti,

Thank you for the submission of your manuscript to EMBO Molecular Medicine. We have now received feedback from the three reviewers who agreed to evaluate your manuscript. All three referees recognize interest of the study but also raise important and partially overlapping concerns that should be addressed in a major revision. If you would like to discuss further the points raised by the referees, I am available to do so via email or video. Let me know if you are interested in this option.

Further consideration of a revision that addresses reviewers' concerns in full will entail a second round of review. EMBO Molecular Medicine encourages a single round of revision only and therefore, acceptance or rejection of the manuscript will depend on the completeness of your responses included in the next, final version of the manuscript. For this reason, and to save you from any frustrations in the end, I would strongly advise against returning an incomplete revision.

We would welcome the submission of a revised version within three months for further consideration. Please let us know if you require longer to complete the revision.

I look forward to receiving your revised manuscript.

Yours sincerely,

Zeljko Durdevic

Zeljko Durdevic
Senior Editor
EMBO Molecular Medicine

*****

When submitting your revised manuscript, please carefully review the instructions that follow below. We perform an initial quality control of all revised manuscripts before re-review; failure to include requested items will delay the evaluation of your revision.

We require:

1) A .docx formatted version of the manuscript text (including legends for main figures, EV figures and tables). Please make sure that the changes are highlighted to be clearly visible.

2) Individual production quality figure files as .eps, .tif, .jpg (one file per figure). For guidance, download the 'Figure Guide PDF': (https://www.embopress.org/page/journal/17574684/authorguide#figureformat).

3) A .docx formatted letter INCLUDING the reviewers' reports and your detailed point-by-point responses to their comments. As part of the EMBO Press transparent editorial process, the point-by-point response is part of the Review Process File (RPF), which will be published alongside your paper.

4) A complete author checklist, which you can download from our author guidelines (https://www.embopress.org/page/journal/17574684/authorguide#submissionofrevisions). Please insert information in the checklist that is also reflected in the manuscript. The completed author checklist will also be part of the RPF.

5) Please note that all corresponding authors are required to supply an ORCID ID for their name upon submission of a revised manuscript.

6) It is mandatory to include a 'Data Availability' section after the Materials and Methods. Before submitting your revision, primary datasets produced in this study need to be deposited in an appropriate public database, and the accession numbers and

database listed under 'Data Availability'. Please remember to provide a reviewer password if the datasets are not yet public (see https://www.embopress.org/page/journal/17574684/authorguide#dataavailability).

In case you have no data that requires deposition in a public database, please state so in this section. Note that the Data Availability Section is restricted to new primary data that are part of this study.

7) For data quantification: please specify the name of the statistical test used to generate error bars and P values, the number (n) of independent experiments (specify technical or biological replicates) underlying each data point and the test used to calculate p-values in each figure legend. The figure legends should contain a basic description of n, P and the test applied. Graphs must include a description of the bars and the error bars (s.d., s.e.m.). See also 'Figure Legend' guidelines: https://www.embopress.org/page/journal/17574684/authorguide#figureformat

8) At EMBO Press we ask authors to provide source data for the main manuscript figures. You will receive a separate email with instructions for providing source data with your revised manuscript, including how to upload and organize the files.

9) Our journal encourages inclusion of *data citations in the reference list* to directly cite datasets that were re-used and obtained from public databases. Data citations in the article text are distinct from normal bibliographical citations and should directly link to the database records from which the data can be accessed. In the main text, data citations are formatted as follows: "Data ref: Smith et al, 2001" or "Data ref: NCBI Sequence Read Archive PRJNA342805, 2017". In the Reference list, data citations must be labeled with "[DATASET]". A data reference must provide the database name, accession number/identifiers and a resolvable link to the landing page from which the data can be accessed at the end of the reference. Further instructions are available at .

10) We replaced Supplementary Information with Expanded View (EV) Figures and Tables that are collapsible/expandable online. A maximum of 5 EV Figures can be typeset. EV Figures should be cited as 'Figure EV1, Figure EV2" etc... in the text and their respective legends should be included in the main text after the legends of regular figures.

- For the figures that you do NOT wish to display as Expanded View figures, they should be bundled together with their legends in a single PDF file called *Appendix*, which should start with a short Table of Content. Appendix figures should be referred to in the main text as: "Appendix Figure S1, Appendix Figure S2" etc.

- Additional Tables/Datasets should be labeled and referred to as Table EV1, Dataset EV1, etc. Legends have to be provided in a separate tab in case of .xls files. Alternatively, the legend can be supplied as a separate text file (README) and zipped together with the Table/Dataset file.

See detailed instructions here:

.

11) The paper explained: EMBO Molecular Medicine articles are accompanied by a summary of the articles to emphasize the major findings in the paper and their medical implications for the non-specialist reader. Please provide a draft summary of your article highlighting

- the medical issue you are addressing,

- the results obtained and

- their clinical impact.

This may be edited to ensure that readers understand the significance and context of the research. Please refer to any of our published articles for an example.

12) Author contributions: You will be asked to provide CRediT (Contributor Role Taxonomy) terms in the submission system. These replace a narrative author contribution section in the manuscript.

13) A Conflict of Interest statement should be provided in the main text.

14) Every published paper now includes a 'Synopsis' to further enhance discoverability. Synopses are displayed on the journal webpage and are freely accessible to all readers. They include a short stand first (maximum of 300 characters, including space) as well as 2-5 one-sentences bullet points that summarizes the paper. Please write the bullet points to summarize the key NEW findings. They should be designed to be complementary to the abstract - i.e. not repeat the same text. We encourage inclusion of key acronyms and quantitative information (maximum of 30 words / bullet point). Please use the passive voice. Please attach these in a separate file or send them by email, we will incorporate them accordingly.

Please also suggest a striking image or visual abstract to illustrate your article as a PNG file 550 px wide x 300-600 px high.

15) Include a Reagents and Tools Table as part of the Methods section, which can be downloaded from our author guidelines (https://www.embopress.org/page/journal/17574684/authorguide#structuredmethods)

EMBO Molecular Medicine has a "scooping protection" policy, whereby similar findings that are published by others during review or revision are not a criterion for rejection. Should you decide to submit a revised version, I do ask that you get in touch after three months if you have not completed it, to update us on the status.

***** Reviewer's comments *****

Referee #1 (Comments on Novelty/Model System for Author):

The experiments are well performed, but the overallnovelty is limited. AAV vectors are widely used in clinical and preclinical gene therapy, and the paper does not add much to the field. As the mouse model does not reproduce the phenotype of the patients, it is hard to evaluate the clinical impact of this work.

Referee #1 (Remarks for Author):

In this study, Molina-Berenguer and colleagues investigate an AAV-based gene therapy approach aimed at correcting the molecular phenotype of the Gfm1R671C/- mouse model of combined oxidative phosphorylation deficiency type I (COXPD1). COXPD1 represents a severe mitochondrial disorder caused by mutations in GFM1, which encodes the mitochondrial translation elongation factor G1 (EFG1). These pathogenic variants result in a combined deficiency of oxidative phosphorylation complexes. In humans, the disease typically manifests as a neonatal encephalopathy, with or without hepatic involvement, and is frequently associated with early mortality.
The authors employed AAV9 and its derivative AAV9P31 to achieve targeted delivery to the liver and brain, respectively. To enhance tissue specificity and minimize off-target expression, they utilized organ-selective promoters, hAAT for the liver and SYN for the brain. In the liver, administration of AAV9-hAAT-GFM1 effectively restored the biochemical and molecular abnormalities associated with the Gfm1 mutation. In contrast, in the brain, recovery of OXPHOS subunit levels and enzymatic activities was only partial.
Overall, the study is well-conceived and provides convincing evidence that AAV-mediated gene replacement could represent a viable therapeutic strategy for COXPD1, thus offering a realistic prospect for clinical translation. However, the clinical relevance of the findings is unclear at the moment, as the mice show no phenotype. Several points warrant further clarification and discussion.
First, the rationale for the selected vector dose (5×10^{12} vg) should be explicitly justified. Although this dose falls within an acceptable experimental range, it remains relatively high. From a translational perspective, it would be important to assess whether lower doses might yield comparable efficacy while potentially reducing the risk of adverse effects. The authors should clarify whether dose-response analyses were performed or considered.
Second, while the use of tissue-specific promoters is well founded, it may also limit efficacy, particularly in the central nervous system. The Synapsin promoter ensures neuronal expression but excludes glial populations, which could restrict the overall therapeutic impact. It would be of interest to know whether the authors have considered employing a more broadly active promoter to evaluate whether expanded expression could improve the extent of phenotypic rescue in the brain.
A few additional points merit attention. The sequence of Supplementary Figures as cited in the text is confusing and should be revised to follow a logical, sequential order. The use of a multiplicity of infection (MOI) of 50 in the lentiviral experiments appears unusually high, and the authors should clarify the rationale for this choice. Furthermore, the claim that "transduction with p305-GFM1LV resulted in EFG1 expression in a dose-dependent manner" is not clearly supported by the data presented, as the dose dependency is not readily apparent. Finally, the correction of the biochemical defect in fibroblasts following lentiviral transduction appears incomplete, and the authors should provide a possible explanation for this partial rescue.

Referee #2 (Comments on Novelty/Model System for Author):

This study provides the first in vivo demonstration that AAV-mediated GFM1 delivery can restore mitochondrial translation and OXPHOS function in a model of COXPD1, a severe and previously untreatable mitochondrial disorder. While AAV-based gene therapy has been explored in other mitochondrial diseases, targeting GFM1 and achieving functional mitochondrial restoration represents a novel and significant advance.
The manuscript is clearly written, logically structured, and well-illustrated, with an introduction that provides a comprehensive

overview of the clinical context and biological rationale. The work carries a translational potential, as COXPD1 is a fatal condition in infants for which no effective treatment currently exists. Demonstrating a proof-of-concept gene therapy using AAV vectors directed to both liver and brain offers a therapeutic avenue for patients with GFM1 deficiency.
The study is technically sound, with well-controlled experiments conducted in both in vitro (patient fibroblasts) and in vivo (Gfm1R671C/- mouse) models. The experimental design is thorough, and the molecular and biochemical analyses are clearly described and appropriately executed. The inclusion of suitable controls and sex-balanced cohorts enhances the reliability of the findings.
The Gfm1R671C/- knock-in/knock-out mouse model accurately reproduces the molecular defects characteristic of COXPD1, including EFG1 depletion and OXPHOS deficiency in brain and liver, making it an appropriate and relevant system for preclinical evaluation of therapeutic interventions.

Referee #2 (Remarks for Author):

This study provides valuable mechanistic and translational insights and represents a significant advance in the development of gene therapy for COXPD1. However, the manuscript would benefit from minor additional analyses and clarifications to enhance its strength and translational relevance:
1. Clarify sample sizes and statistical analyses, ensuring transparency regarding the number of animals and replicates used for each experiment.
2. Discuss potential immune responses to AAV administration and the long-term stability of transgene expression.
3. If feasible, include functional outcome measures, such as behavioural performance or survival data, to demonstrate therapeutic benefit beyond molecular correction.
4. Acknowledge limitations, including the absence of long-term efficacy assessment, comprehensive toxicity evaluation, and direct evidence of phenotypic improvement (e.g., neuroprotection or extended lifespan).
5. Address the limited statistical power due to small sample sizes and emphasize how this may affect the strength of conclusions.
6. Provide a more detailed analysis of AAV biodistribution, particularly in non-target tissues, to better understand off-target effects and systemic safety.
7. Expand on clinical correlates, such as neurological or metabolic manifestations in the mouse model, to strengthen the translational potential of the findings.

Referee #3 (Comments on Novelty/Model System for Author):

The research study by Molina-Berenguer et al. report development of an AAV9-mediated gene replacement therapeutic approach to treat an autosomal recessive mitochondrial disease caused by mutations in the GFM1 gene called combined oxidative phosphorylation (OXPHOS) deficiency type I (COXPD1). The disease progression is very rapid as patients die in the first days or months of life signifying the urgent need to develop long-lasting and highly effective gene replacement options for this devastating disorder. Patients manifest liver failure and severe neonatal encephalopathy. The authors test two types of gene therapy strategies using an AAV9-vector to target the liver (using AAV-hAAT-GFM1) and an AAV9 variant capsid to target the brain (using AAV9P31-hSyn-GFM1) into Gfm1 R671C/- mice at 6 weeks of age. This approach resulted in partial restoration of EFG1 levels in both the liver and brain and correction of the hepatic COXPD molecular phenotype. However, a partial correction of the COXPD phenotype is observed in the brain. While my enthusiasm for this study remains high and the results of this study are encouraging and the data are of high scientific rigor, I do have a number of major and minor reservations that if addressed would provide for an excellent contribution to EMBO Molecular Medicine.
Major concerns:
1.) The title of the article implies that COXPD1 molecular alterations are not present in 6-week-old Gfm1 R671C/- mice when these mice are treated with AAV9. In a previous study by the same team, the authors examine pathology in 8-week-old mice and observe molecular phenotypes. It is not clear whether Gfm1 R671C/- mice already manifest COXPD1 molecular alterations when they are treated at 6 weeks. If so, then AAV9 treatment at 6 weeks would correct (and not necessarily prevent) the phenotypes in these mice and thereby would potentially provide greater clinical significance. If pathology exists at treatment in this mice, I would suggest changing the title from "prevents" to "corrects".

2.) Also, it is not clear why an earlier time point was not chosen to treat these mice since patients will die within days or months after life. Please include the rationale for a 6-week time point for intervention.

3.) Do Gfm1 R671C/- mice show neurobehavioral deficits, such as epilepsy or other observed behavioral deficits? Also, do the mice show neurodegeneration? If not, please add these details into the manuscript. If these mice do show these features and

behavioral deficits, does the AAV gene therapy treatment impact these phenotypes?

Minor concerns:
1.) In Figure 1b, a specific human GFM1 cDNA probe was utilized, although its human specificity was not tested with a un-injected or sham injected mouse as a control. Please incorporate this important control into this experiment.
2.) The rationale for using the 5x10^12 vg/kg dose of AAV is unclear for these studies. Please include justification why a higher dose was not attempted that would restore EFG1 levels similar to wild-type mouse levels. Similarly, please include justification in the text describing why only neuronal targeting in the brain was attempted in this mouse model using a synapsin promoter driving GFM1 since Gfm1 is expressed ubiquitously throughout the CNS.
3.) Lines 403-404: "Central Nervous System" should be in lower case.

Response to Reviewer's comments

Referee #1 (Comments on Novelty/Model System for Author):

The experiments are well performed, but the overall novelty is limited. AAV vectors are widely used in clinical and preclinical gene therapy, and the paper does not add much to the field. As the mouse model does not reproduce the phenotype of the patients, it is hard to evaluate the clinical impact of this work.
We acknowledge the limitations pointed out by the reviewer, but we do not think that what we add to the field is not relevant. The reviewer is right that AAV vectors are widely used in clinical and preclinical studies, but our work is focused on providing approaches to face the need of a treatment for a severe and ultra-rare disease with no therapy options so far, rather than developing or improving the vector tools. Reporting that this approach enables the correct import of a translation factor into mitochondria, and that the human protein is functional in the murine molecular context (which was not obvious, because the human protein had to correctly and functionally interact with the murine mitoribosome) constitute novel relevant information and suggest that this could be a molecularly robust approach.
It is also true that the animal model does not recapitulate the clinical phenotype of the patients, but this is so far the only available in vivo tool to investigate potential therapy approaches for this disorder. We must use whatever means we have in our hands to advance in looking for treatments of these orphan diseases, even with suboptimal models. We cannot claim that our work provides solid bases to jump into treating patients, but we think it provides results encouraging further investigation, refinement and improvement to get closer to a realistic gene therapy for this disease, as discussed in our manuscript.

Referee #1 (Remarks for Author):

In this study, Molina-Berenguer and colleagues investigate an AAV-based gene therapy approach aimed at correcting the molecular phenotype of the Gfm1R671C/- mouse model of combined oxidative phosphorylation deficiency type I (COXPD1). COXPD1 represents a severe mitochondrial disorder caused by mutations in GFM1, which encodes the mitochondrial translation elongation factor G1 (EFG1). These pathogenic variants result in a combined deficiency of oxidative phosphorylation complexes. In humans, the disease typically manifests as a neonatal encephalopathy, with or without hepatic involvement, and is frequently associated with early mortality.
The authors employed AAV9 and its derivative AAV9P31 to achieve targeted delivery to the liver and brain, respectively. To enhance tissue specificity and minimize off-target expression, they utilized organ-selective promoters, hAAT for the liver and SYN for the brain. In the liver, administration of AAV9-hAAT-GFM1 effectively restored the biochemical and molecular abnormalities associated with the Gfm1 mutation. In contrast, in the brain, recovery of OXPHOS subunit levels and enzymatic activities was only partial.
Overall, the study is well-conceived and provides convincing evidence that AAV-mediated gene replacement could represent a viable therapeutic strategy for COXPD1, thus offering a realistic prospect for clinical translation. However, the clinical relevance of the findings is unclear at the moment, as the mice show no phenotype. Several points warrant further clarification and discussion.

First, the rationale for the selected vector dose (5×10^{12} vg) should be explicitly justified. Although this dose falls within an acceptable experimental range, it remains relatively high. From a translational perspective, it would be important to assess whether lower doses might yield comparable efficacy while potentially reducing the risk of adverse effects. The authors should clarify whether dose-response analyses were performed or considered.

It is important to clarify that the dose was $5x10^{12}$ vg ***per kg*** (not per mouse); the total dose per mouse was around $1x10^{11}$ vg, with variations depending on the exact weight of the mice, treated at age 6 weeks.
We agree with the reviewer about the importance of dose response analyses, as high AAV doses can be associated to adverse effects. However, our current work was aimed to generate evidence that an AAV-mediated gene therapy ameliorates the molecular/biochemical phenotype observed in the COXPD1 animal model, thus identifying a potential therapy to be further investigated in additional studies. We choose a dose of $5x10^{12}$ vg/kg, based on our previous experience with AAV in mice (PMID: 24448160, 29284302, 30900470, 33232869), and on ranges of doses used in approved AAV treatments and clinical trials. After seeing that this was not able to fully correct the phenotype (especially for the brain-targeted vector), we decided to expand the molecular analyses rather than repeating experiments at lower doses (e.g., effect on other mito-ribosomal proteins and on mitochondrial mRNA) considering these could provide information about the molecular response triggered by the therapy in the animal model.
We think that reporting the results obtained with the $5x10^{12}$ vg/kg dose is informative enough considering the intended scope of this study. Intravenous doses of FDA-approved treatments with AAV vectors (Onasemnogene abeparvovec, Etranacogene dezaparvovec, Valoctocogene roxaparvovec, Delandistrogene moxeparvovec and Fidanacogene elaparvovec) range 0.5 – 133 $x10^{12}$ vg/kg (being in fact $1.1x10^{14}$ vg/kg for the AAV9 Onasemnogene abeparvovec to treat spinal muscular atrophy). Therefore, we think the dose falls within a clinically reasonable range. We have introduced the dose justification in the material and methods section (lines 218-225) and comments on dose-related aspects in the discussion of the revised version of the manuscript (lines 675-692).
In any case, we are currently conducting another study that will expand our knowledge about how this therapy approach can be improved and refined, introducing a new promoter (see answer to the second question below), as well as dose-response studies.

Second, while the use of tissue-specific promoters is well founded, it may also limit efficacy, particularly in the central nervous system. The Synapsin promoter ensures neuronal expression but excludes glial populations, which could restrict the overall therapeutic impact. It would be of interest to know whether the authors have considered employing a more broadly active promoter to evaluate whether expanded expression could improve the extent of phenotypic rescue in the brain.
Indeed, one of the limitations of our study is that it reports the effect of two different vectors: one of them targeted to the liver (not reaching the brain and therefore carrying a liver promoter), and the second one with a modified capsid that crosses the blood brain barrier and using the synapsin promoter that would allow us to study the effect in neurons, which constitute most cellular mass in the CNS. However, as the reviewer rightly points out, this promoter excludes glial cells, and broadening the therapeutic target to include them could significantly improve the efficacy of the treatment, which will have to be also addressed in further studies. We have explicitly justified why we used the synapsin promoter and commented this limitation in the revised version of the manuscript by reformulating one part of the discussion section (lines 734-742).
Having this in mind, we still think that our results clearly show that an AAV-mediated cell addition strategy can be used to correct the COXPD1 molecular phenotype in both tissues, but the vector and target strategy should be improved if this approach is to be translated to patients. The vector should be able to correct the phenotype at least in the two mainly affected systems (liver and CNS, including glial cells). For this reason, we are now conducting a new study in which we will use the AAV9P31 serotype (which we have shown to transduce liver and brain) with a newly designed promoter that drives expression in both tissues. This will constitute a new study that, in addition, will include dose-response studies as well as long term efficacy studies).

A few additional points merit attention.

The sequence of Supplementary Figures as cited in the text is confusing and should be revised to follow a logical, sequential order.

Following editorial instructions, the revised version of the manuscript has changed the way and order the Supplementary Information is shown. Now, the manuscript contains the figures, tables and schemes initially submitted as Supplementary Information in two different sets: the “Expanded View (EV) Figures and Tables” and an “Appendix”. We hope the new distribution and order in this revised form now follows a more logical and clear order.

The use of a multiplicity of infection (MOI) of 50 in the lentiviral experiments appears unusually high, and the authors should clarify the rationale for this choice.

We thank the observation of the reviewer, which has helped us detect a point that was probably no clear enough in the methods description. The MOI values we indicate are based on the titres obtained for our LV vectors. In the initial version of the manuscript, the titration method was just referred to our previous publication (Torres-Torronteras et al, 2011), without any details or explicit indication that the titration was carried out by treating HEK293T cells with serial dilutions of the production and further estimating the transduction rates by EGFP fluorescence flow cytometry. Because these are not absolute quantitative titres (such as those that could be derived from quantitative PCR methods or ELISA), the MOI is relative to the titre obtained with a proliferative cell line. In order to clarify this, we have inserted an explicit indication that the HEK293T cell line was used for the titration of the LV (lines 139-140).

We in fact used two different MOI (10 and 50) for these experiments, as described in the material and methods section and reported in the results (Figure EV2). We chose these two MOIs because the cells used in the experiment (cultured skin fibroblasts) are slow-cycling cells. Although LV can transduce cells in quiescence, the transduction is more efficient in dividing cells, and therefore we decided to use a moderate (10) and high (50) MOI in order to explore a possible dose-dependent response, together with favouring the transduction efficacy in this slow-dividing culture. In addition, based on our previous experience (Torres-Torronteras et al, 2011; PMID: 21451581), MOIs of 10 and 15 resulted in variable transduction efficiencies in proliferating cultured cells, and a MOI of 100 was used in murine hematopoietic progenitors, so that we decided to use MOIs within this range for our current experiments. Following the reviewer’s recommendation, we added the justification for the MOI used in the Material and Methods section of the revised version of the manuscript (lines 144-147).

Furthermore, the claim that "transduction with p305-GFM1LV resulted in EFG1 expression in a dose-dependent manner" is not clearly supported by the data presented, as the dose dependency is not readily apparent.

This claim was based on the observation of a trend to see higher EFG1 levels (as assessed by western-blot analysis) in cells treated with MOI 50 than those observed for cells treated with MOI 10 (Figure EV2a). Although statistical difference could not be reached, a consistent tendency is observed in all three patient-derived cell lines. However, we agree with the reviewer that the claim is not fully justified because there is some overlap in the results and also because the difference is, in average, barely an increase of 20 – 30%, far from a 5-fold increase that could be expected from MOI 10 to 50. Therefore, we have reformulated the claim in the revised version of the manuscript (lines 414-415).

Finally, the correction of the biochemical defect in fibroblasts following lentiviral transduction appears incomplete, and the authors should provide a possible explanation for this partial rescue.

The reviewer is right that the rescue of complex I and IV activities in LV-treated patients’ fibroblasts only reaches values around 75% of those observed in the controls. Considering that

EFG1 levels in LV-treated patient cells are 3 to 5-fold above those observed in normal controls, the most plausible explanation for this partial restoration of the downstream effect of EFG1 is that not all cells were effectively transduced by the LV, which would partially dilute the full restoration in transduced cells. In fact, flow cytometry studies conducted in the transduced fibroblasts revealed that the percentages of transduced (GFP positive) cells were 62%, 88% and 67%, which is consistent with the above explanation. We did not include this result in the manuscript because the characterization of the LV tool was not the main objective of the work, but we can insert this result if the reviewer and/or the editor think that it is worth to do it. Alternatively, we cannot rule out that the LV-mediated over-restoration of EFG1 levels in transduced cells could result in a molecular imbalance that somehow makes mitochondrial translation less efficient than that observed with normal EFG1 levels. We have included these possible explanations in the revised version of the manuscript (lines 629-637).

Referee #2 (Comments on Novelty/Model System for Author):

This study provides the first in vivo demonstration that AAV-mediated GFM1 delivery can restore mitochondrial translation and OXPHOS function in a model of COXPD1, a severe and previously untreatable mitochondrial disorder. While AAV-based gene therapy has been explored in other mitochondrial diseases, targeting GFM1 and achieving functional mitochondrial restoration represents a novel and significant advance.
The manuscript is clearly written, logically structured, and well-illustrated, with an introduction that provides a comprehensive overview of the clinical context and biological rationale. The work carries a translational potential, as COXPD1 is a fatal condition in infants for which no effective treatment currently exists. Demonstrating a proof-of-concept gene therapy using AAV vectors directed to both liver and brain offers a therapeutic avenue for patients with GFM1 deficiency.
The study is technically sound, with well-controlled experiments conducted in both in vitro (patient fibroblasts) and in vivo (Gfm1R671C/- mouse) models. The experimental design is thorough, and the molecular and biochemical analyses are clearly described and appropriately executed. The inclusion of suitable controls and sex-balanced cohorts enhances the reliability of the findings.
The Gfm1R671C/- knock-in/knock-out mouse model accurately reproduces the molecular defects characteristic of COXPD1, including EFG1 depletion and OXPHOS deficiency in brain and liver, making it an appropriate and relevant system for preclinical evaluation of therapeutic interventions.

We appreciate the positive opinion of the referee about our work.

Referee #2 (Remarks for Author):

This study provides valuable mechanistic and translational insights and represents a significant advance in the development of gene therapy for COXPD1. However, the manuscript would benefit from minor additional analyses and clarifications to enhance its strength and translational relevance:

1. Clarify sample sizes and statistical analyses, ensuring transparency regarding the number of animals and replicates used for each experiment.
We have introduced in the material and methods a brief statement about the number of animals treated and replicates (lines 228-236), and some additional clarifications in the Statistical analysis paragraph (lines 381-382). Each figure legend indicates the specific statistical test used for each analysis.

2. Discuss potential immune responses to AAV administration and the long-term stability of transgene expression.
We have introduced new paragraphs discussing these and other points in the revised version of the manuscript (lines 839-856).

3. If feasible, include functional outcome measures, such as behavioural performance or survival data, to demonstrate therapeutic benefit beyond molecular correction.
Unfortunately, none of the endpoints so far investigated for this animal model have revealed any phenotypical traits resembling the clinical presentation of the disease, beyond the molecular COXPD1 phenotype. As reported in our previous article (Molina-Berenguer et al, 2022; PMID: 34919756) there is no reduction in the lifespan (monitored until week 80 of life) and weight curves are not affected (except a very mild reduced body weight after postnatal day 7, which reached statistical significance in females only, and resumed to indistinguishable weight from the weaning). Neither differences were observed in several neuromuscular tests performed at 7 weeks of age (weight-lift, hanging-wire and treadmill) or at 25 weeks (rotarod) (published data at the PhD thesis, Molina-Berenguer http://hdl.handle.net/10803/690571). Therefore, we cannot show any effects beyond the molecular correction.
We believe that the observed effects on the biochemical endpoints constitute a proof of concept that this therapy has potential and deserves being further investigated. In this regard, we are currently conducting another study that will expand our knowledge about how this therapy approach can be improved and refined, introducing a new promoter, including dose-response studies, and exploring more functional traits not studied before (mainly behavioural tests). In case these additional tests reveal phenotypic traits not yet detected, we will be able to see whether the therapy has some effect on them.

4. Acknowledge limitations, including the absence of long-term efficacy assessment, comprehensive toxicity evaluation, and direct evidence of phenotypic improvement (e.g., neuroprotection or extended lifespan).
We have included a new comment in the discussion acknowledging these limitations (lines 839-856) of the revised version of the manuscript). Furthermore, a long-term efficacy study is ongoing with a single vector expressing the transgene both in hepatocytes and CNS, which will complement and expand the current report.

5. Address the limited statistical power due to small sample sizes and emphasize how this may affect the strength of conclusions.
As the reviewer points out, the limited number of experiments (often derived from the need to minimize numbers of animals to be used) affects the statistical power and sometimes limits the possibility to detect effects. Since parametric tests can only be used for samples derived from normal distributions among other requirements, and the reduced number of experiments does not allow us to reliably assume normality, we chose to use non-parametric tests, which have less requirements but have more limited statistical power (i.e., there is risk to miss some effects that could have been detected with more data). On the other hand, the use of non-parametric tests provides more robustness to the differences that are actually found to be statistically significant. We have added a comment on this general issue in the paragraph describing the statistical methods (lines 381-382) and a specific comment in one part of the discussion where this issue could be particularly relevant (lines 771-777).

6. Provide a more detailed analysis of AAV biodistribution, particularly in non-target tissues, to better understand off-target effects and systemic safety.
Following the reviewer’s suggestion, we have analysed three different non-target tissues (skeletal muscle, heart and spleen) to expand the biodistribution data that, in the first

submission, included target tissues only (liver and brain). Those tissues had been collected in our experiments with the AAV9 and AAV9P31 vectors. The new analysis has revealed that the vector copy number in non-target tissues is low but not negligible (between 0.01 and 0.1 vg/cell in most cases), although the VCN in spleen reached higher values in some animals, especially for the AAV9P31 vector (up to 0.3 vg/cell). For AAV9, the only tissue that reached vector copy number above 1 vg/cell was liver (1 – 4 vg/cell for females and 5 – 13 vg/cell for males). These results are in reasonable agreement with those previously reported for biodistribution of AAV9, where liver is the most transduced tissue, with all the other tissues (including brain, heart and spleen) showing VCN ten-fold lower or below (Walkey et al, Mol Ther 2025, 33:1282-99; doi: 10.1016/j.ymthe.2025.01.041). For AAV9P31, as already mentioned in the first submission, the brain reached the highest VCN among the analysed tissues, followed by the liver. The biodistribution was lower in non-target tissues, which parallels what was previously reported (Bunuales et al, Gene Ther 2024,31:455-66. doi: 10.1038/s41434-024-00466-w). Taken together, these results suggest that non-target tissues should be studied when accurate safety studies will be conducted in the future for this therapy approach.
In the revised version of the manuscript, the new results have been included in the new Figure EV4, mentioned in the results section (lines 457-456 and 491-493) and in the discussion (lines 713-722).
As already mentioned, we are currently conducting another study that will expand our knowledge about how this therapy approach can be improved and refined, with a new promoter. This work will further expand the biodistribution data for the AAV9P31 vector as well.

7. Expand on clinical correlates, such as neurological or metabolic manifestations in the mouse model, to strengthen the translational potential of the findings.
As mentioned in our response to the point 3 above, we failed to find any phenotypic traits recapitulating the clinical presentation of COXPD1. As mentioned, we are conducting a new work with a battery of new functional studies, including behavioural tests, to further explore this area. Having this limitation in mind, we think the new paragraph inserted at the end of the discussion (lines 839-856, indicated at the point 4 above) clearly points out that there is a translational potential of the therapy but also postulates the need to conduct further and deeper exploration of the "clinical" phenotype of the model.

Referee #3 (Comments on Novelty/Model System for Author):

The research study by Molina-Berenguer et al. report development of an AAV9-mediated gene replacement therapeutic approach to treat an autosomal recessive mitochondrial disease caused by mutations in the GFM1 gene called combined oxidative phosphorylation (OXPHOS) deficiency type I (COXPD1). The disease progression is very rapid as patients die in the first days or months of life signifying the urgent need to develop long-lasting and highly effective gene replacement options for this devastating disorder. Patients manifest liver failure and severe neonatal encephalopathy. The authors test two types of gene therapy strategies using an AAV9-vector to target the liver (using AAV-hAAT-GFM1) and an AAV9 variant capsid to target the brain (using AAV9P31-hSyn-GFM1) into Gfm1 R671C/- mice at 6 weeks of age. This approach resulted in partial restoration of EFG1 levels in both the liver and brain and correction of the hepatic COXPD molecular phenotype. However, a partial correction of the COXPD phenotype is observed in the brain. While my enthusiasm for this study remains high and the results of this study are encouraging and the data are of high scientific rigor, I do have a number of major and minor reservations that if addressed would provide for an excellent contribution to EMBO Molecular Medicine.

We appreciate the positive opinion of the referee about our work.

Major concerns:

1.) The title of the article implies that COXPD1 molecular alterations are not present in 6-week-old Gfm1 R671C/- mice when these mice are treated with AAV9. In a previous study by the same team, the authors examine pathology in 8-week-old mice and observe molecular phenotypes. It is not clear whether Gfm1 R671C/- mice already manifest COXPD1 molecular alterations when they are treated at 6 weeks. If so, then AAV9 treatment at 6 weeks would correct (and not necessarily prevent) the phenotypes in these mice and thereby would potentially provide greater clinical significance. If pathology exists at treatment in this mice, I would suggest changing the title from "prevents" to "corrects".
We thank the reviewer for raising this point, which is actually relevant because, as the reviewer points out, the correction of the molecular phenotype is of greater clinical significance than its prevention. In fact, although many of the groups studied in our previous report correspond to 8-week old mice (or 6 to 8 weeks old mice), other molecular alterations, such as EFG1 depletion, are already observed in 6-week old mice. Therefore, following the reviewer's suggestion, we changed the title from title from "prevents" to "corrects".

2.) Also, it is not clear why an earlier time point was not chosen to treat these mice since patients will die within days or months after life. Please include the rationale for a 6-week time point for intervention.
We thank the reviewer for raising this important point. In most cases, patients die during the infancy, and even those that survive longer present CNS affectations caused by impaired development during the infancy, which we can anticipate that will not be reverted by the treatment. Therefore, as the reviewer points out, treatment of patients will have to be applied as early as possible during the infancy to prevent a premature death and further irreversible CNS affectations.
However, we had to design of our studies considering the available animal model. Although the molecular alterations (EFG1 depletion and OXPHOS dysfunction) were detected in mice since 6 – 8 weeks of life, they did not affect the lifespan of mice, their neuromuscular function or other so far studied endpoints (reported in Molina-Berenguer et al, 2022; PMID: 34919756, and in Molina-Berenguer PhD thesis http://hdl.handle.net/10803/690571). Since the transduced gene remains episomal after AAV transduction, treatment of younger mice would have resulted in dilution of the GFM1 copy number per cell, at least in the liver, due to cell proliferation during development. This dilution would have posed some difficulties in the observation of the results in the animal model. This, combined with the fact that in mice there is not reduced life-span or other early "clinical" manifestations, beyond the molecular phenotype, made us choose the age of 6 weeks for the treatment and 10 weeks for sacrifice and endpoint analyses. With this design, the vector is not significantly diluted between the administration and the sacrifice point, and we maximize the chances to observe correction effects (as pointed by the reviewer in the above point), as the molecular phenotype is already present at 6 – 8 weeks of age.
In any case, the dilution problem will have to be faced and solved at the time of the clinical use of this approach. At that time, the main target effect will have to be also considered, because the dilution effect will be very relevant for the liver but not as relevant for the CNS, because in humans cell proliferation in the CNS is very limited after birth.
We have included a paragraph justifying the rationale for the treatment at six weeks in the revised version of the manuscript (lines 221-225).

3.) Do Gfm1 R671C/- mice show neurobehavioral deficits, such as epilepsy or other observed behavioral deficits? Also, do the mice show neurodegeneration? If not, please add these

details into the manuscript. If these mice do show these features and behavioral deficits, does the AAV gene therapy treatment impact these phenotypes?
We have not observed any epileptic episodes or other noticeable signs of CNS dysfunction in Gfm1 R671C/- mice at any age. Functional neuromuscular tests (weight-lift, hanging-wire, treadmill and rotarod) could not detect any differences in the mutant performance as compared with WT mice. Since we could not see any of these phenotypical traits in the mice at any age, we did not explore neurodegeneration by CNS histological analyses, imaging, biomarkers, nor conduct behavioural tests. We have included this information in the revised version of the manuscript (lines 839-856), so that the readers can be aware that the lack of phenotype did not allow us to investigate the potential effect of the therapy on CNS deficits beyond the molecular phenotype.
However, we are aware of the importance of this point when we approach COXPD1 and a potential treatment for this severe disease, and are now conducting another study which includes a more detailed exploration of the CNS phenotype (mainly behavioural tests), because we cannot rule out subtle deficits that could not be detected in our previous tests and observations.

Minor concerns:

1.) In Figure 1b, a specific human GFM1 cDNA probe was utilized, although its human specificity was not tested with a un-injected or sham injected mouse as a control. Please incorporate this important control into this experiment.
We agree with the reviewer that this is an important control that should be included. The human GFM1 cDNA probe used in this study (human TaqMan MGB expression assay Hs00227997_m1) has shown to be actually specific for the human cDNA in our hands. We observed null amplifications when tested retrotranscribed mRNA from untreated WT mice and vehicle-treated KI/KO mice. We had included this information in the narrative of the results section already in our first submission (now, lines 464-465 for Fig 1b, and lines 495-496 for Fig. 2b). Now, for clarity, we have included this statement in the corresponding figure legends as well (figures 1b and 2b in the revised version of the manuscript).

2.) The rationale for using the 5x10^12 vg/kg dose of AAV is unclear for these studies. Please include justification why a higher dose was not attempted that would restore EFG1 levels similar to wild-type mouse levels. Similarly, please include justification in the text describing why only neuronal targeting in the brain was attempted in this mouse model using a synapsin promoter driving GFM1 since Gfm1 is expressed ubiquitously throughout the CNS.
We chose this dose based on our previous experience treating mice with a different genetic defect with AAV8 and several hepatic promoters, including the AAT promoter used here. These studies showed that doses around 10^12 vg/kg resulted in efficacious transduction, expression of the transgene, and correction of the phenotype (PMID: 24448160, 29284302, 30900470, 33232869). In addition, the most frequent doses used in past and currently being conducted clinical trials using AAV vectors fall around 10^12 vg/kg, often lower (reviewed at PMID: 35027711). It is true that the only FDA-approved use of AAV9 (Onasemnogene abeparvovec, for spinal muscular atrophy) goes at a dose of 1.1x10^14 vg/kg. But clinical experience shows that high doses may lead to aminotransferase elevation derived from liver toxicity.
COXPD1 is a recessive disease, and our previous characterization of the animal model showed that heterozygous KI animals had reduced levels of EFG1 but preserved OXPHOS function (Molina-Berenguer et al, 2022). Accordingly, the partial restoration of EFG1 levels observed with this dose in our animal model (reaching values of 20% - 65% of the normal values), led to a nearly total recovery of OXPHOS function, when measured as CI and CIV activities in liver and brain (with the only exception of CIV activities in the liver of AAV9-treated females, in which partially restored 20% of EFG1 levels led to 58% of normal CIV activity). For this reason, we think the moderate dose we tested is informative enough, considering the obtained results

and the scope of this study. In order to explain this position, following the reviewer's suggestion, we have included a justification of the chosen dose in the Material and Methods section (lines 218-221) as well as a comment in the discussion justifying why we think a higher dose would not have added much more information as compared to what we already obtained (lines 675-692).
We used the synapsin promoter because it would allow us to study the effect in neurons, which constitute most cellular mass in the CNS. As the reviewer points out, it only targets neurons and not glial cells. With this promoter, the molecular restoration observed in total brain was relevant and nearly total when assessed as CI and CIV enzyme activity. However, we fully acknowledge that even if glial cells are less contributors to the total CNS mass, their physiological role is critical and so the restoration of the mitochondrial function of this subpopulation will be required to ensure clinical efficacy. We have explicitly justified why we used the synapsin promoter and commented this limitation in the revised version of the manuscript by reformulating one part of the discussion section (lines 734-742). However, we think the results sufficiently show the potential of this therapy approach for this disorder, and stablish bases to conduct more detailed studies to refine doses and endpoints, towards a future proposal for use in patients, based on robust preclinical studies conducted in this animal model (so far the only one available). In this regard, as commented to the reviewer 1, who also raised this issue, we are now conducting a new study in which we will use the AAV9P31 serotype (which we have shown transduces the liver and the brain) with a newly designed promoter that drives expression in both tissues, constituting a new study in which we will explore more in detail the expression in other cell types.

3.) Lines 403-404: "Central Nervous System" should be in lower case.

Done

18th Mar 2026

Dear Dr. Marti,

Thank you for the submission of your revised manuscript to EMBO Molecular Medicine. I am pleased to inform you that we will be able to accept your manuscript pending the following final amendments:

1) Please address referee #3 remaining concern.
2) Authors: There is a name discrepancy between Antoni Vallbona in our system and Antoni Vallbona-Garciain the manuscript. Please correct.
3) Figures:
- Please use capital letters for the panels.
- We note that SDHA western blot images in Fig. 1C and Fig. 2C were reused in Fig. 3A and 3B, respectively. Please cite in the figure legends every reused image.
- In Fig. EV3 please add dotted highlight boxes for the area of magnification.
- The resolution is too low for blot images. This reduction in resolution is commonly caused by converting original 16-bit TIFF files to RGB format for publication. While this is not inherently problematic, it can raise concerns about image integrity for critical readers. To avoid any misunderstanding and to meet EMBO Press standards, we kindly ask that you: Please supply the blot source data at the original captured resolution. Please upload the blot source data files as .tiff or pdf. Please do not use PowerPoint files or convert the files to Tiff from PowerPoint.
4) In the main manuscript file, please do the following:
- Please address all comments suggested by our data editors listed below:
o Figure legends:
1. Please note that the legends for figures S3 is not provided in the sequential manner. This needs to be rectified.
2. Please note that the exact p values are not provided in the legends of figures 1A-C; 2A, C.
3. Please note that information related to n is missing in the legends of figures 1A-C; 3A, B; 4A, B; 5A, B; 6A, B; 7A, B; EV1 A, C, D, E; S3 A-D; S4A, B.
- Figures should be called out in a sequential order. Currently, Fig 4A,B, is called out before Fig 3B, please correct. Also, add panel callouts for Fig EV1 and EV3.
- Please place Methods after Discussion.
- Indicate in legends exact n and exact p values, not a range, along with the statistical test used. To keep the figures "clear" some authors found providing an Appendix table Sx with all exact p-values preferable. You are welcome to do this if you want to.
- Rename "Conflict of interest statement" to "Disclosure and competing interests statement". We updated our journal's competing interests policy in January 2022 and request authors to consider both actual and perceived competing interests. Please review the policy https://www.embopress.org/competing-interests and update your competing interests if necessary.
- Author contributions: Please remove it from the manuscript and specify author contributions in our submission system. CRediT has replaced the traditional author contributions section because it offers a systematic machine-readable author contributions format that allows for more effective research assessment. You are encouraged to use the free text boxes beneath each contributing author's name to add specific details on the author's contribution. More information is available in our guide to authors:
https://www.embopress.org/page/journal/17574684/authorguide#authorshipguidelines
- In data availability statement please remove provided text and add the sentence "This study includes no data deposited in external repositories."
- Please correct the reference citation in the reference list. Where there are more than 10 authors on a paper, 10 will be listed, followed by "et al.". Please check "Author Guidelines" for more information.
https://www.embopress.org/page/journal/17574684/authorguide#referencesformat
5) Appendix: Please add page numbers to table of contents.
6) The Paper Explained: Please add it to the main manuscript file.
7) Synopsis:
- Please check your synopsis text and image before submission with your revised manuscript. Please be aware that in the proof stage minor corrections only are allowed (e.g., typos).
8) As part of the EMBO Publications transparent editorial process (see our Editorial at http://embomolmed.embopress.org/content/2/9/329), EMBO Molecular Medicine will publish online a Review Process File (RPF) to accompany accepted manuscripts. This file will be published in conjunction with your paper and will include the anonymous referee reports, your point-by-point response and all pertinent correspondence relating to the manuscript. Let us know if you want to remove or not any figures from it prior to publication. Please note that the Authors checklist will be published at the end of the RPF.
9) Please provide a point-by-point letter INCLUDING my comments as well as the reviewer's reports and your detailed responses (as Word file).

I look forward to reading a new revised version of your manuscript as soon as possible.

Yours sincerely,

Zeljko Durdevic

Zeljko Durdevic
Senior Editor
EMBO Molecular Medicine

*** Instructions to submit your revised manuscript ***

*** PLEASE NOTE *** As part of the EMBO Publications transparent editorial process initiative (see our Editorial at https://www.embopress.org/doi/pdf/10.1002/emmm.201000094), EMBO Molecular Medicine will publish online a Review Process File to accompany accepted manuscripts.

In the event of acceptance, this file will be published in conjunction with your paper and will include the anonymous referee reports, your point-by-point response and all pertinent correspondence relating to the manuscript. If you do NOT want this file to be published, please inform the editorial office at contact@embomolmed.org.

*****

When preparing your revised manuscript, please refer to our guidelines: https://link.springer.com/journal/44321/submission-guidelines#cms-Revised-submissions. We perform an initial quality control of all revised manuscripts before re-review; failure to include requested items will delay the evaluation of your revision.

We require:

1) A .docx formatted version of the manuscript text (including legends for main figures, EV figures and tables). Please make sure that the changes are highlighted to be clearly visible.

2) Individual production quality figure files as .eps, .tif, .jpg (one file per figure). For guidance, download the 'Figure Guide PDF': https://media.springernature.com/original/springer-cms/rest/v1/content/27825798/data/v1.

3) A .docx formatted letter INCLUDING the reviewers' reports and your detailed point-by-point responses to their comments. As part of the EMBO Press transparent editorial process, the point-by-point response is part of the Review Process File (RPF), which will be published alongside your paper.

4) A complete author checklist, which you can download from our author guidelines. Please insert information in the checklist that is also reflected in the manuscript. The completed author checklist will also be part of the RPF.

5) Please note that all corresponding authors are required to supply an ORCID ID for their name upon submission of a revised manuscript.

6) It is mandatory to include a 'Data Availability' section after the Materials and Methods. Before submitting your revision, primary datasets produced in this study need to be deposited in an appropriate public database, and the accession numbers and database listed under 'Data Availability'. Please remember to provide a reviewer password if the datasets are not yet public.

In case you have no data that requires deposition in a public database, please state so in this section. Note that the Data Availability Section is restricted to new primary data that are part of this study.

7) For data quantification: please specify the name of the statistical test used to generate error bars and P values, the number (n) of independent experiments (specify technical or biological replicates) underlying each data point and the test used to calculate p-values in each figure legend. The figure legends should contain a basic description of n, P and the test applied. Graphs must include a description of the bars and the error bars (s.d., s.e.m.).

8) At EMBO Press we ask authors to provide source data for the main manuscript figures. You will receive a separate email with instructions for providing source data with your revised manuscript, including how to upload and organize the files.

9) Our journal encourages inclusion of *data citations in the reference list* to directly cite datasets that were re-used and obtained from public databases. Data citations in the article text are distinct from normal bibliographical citations and should directly link to the database records from which the data can be accessed. In the main text, data citations are formatted as follows: "Data ref: Smith et al, 2001" or "Data ref: NCBI Sequence Read Archive PRJNA342805, 2017". In the Reference list, data citations must be labeled with "[DATASET]". A data reference must provide the database name, accession number/identifiers and a resolvable link to the landing page from which the data can be accessed at the end of the reference.

10) We replaced Supplementary Information with Expanded View (EV) Figures and Tables that are collapsible/expandable online. A maximum of 5 EV Figures can be typeset. EV Figures should be cited as 'Figure EV1, Figure EV2" etc... in the text and their respective legends should be included in the main text after the legends of regular figures.

- For the figures that you do NOT wish to display as Expanded View figures, they should be bundled together with their legends in a single PDF file called *Appendix*, which should start with a short Table of Content. Appendix figures should be referred to in the main text as: "Appendix Figure S1, Appendix Figure S2" etc.

- Additional Tables/Datasets should be labeled and referred to as Table EV1, Dataset EV1, etc. Legends have to be provided in a separate tab in case of .xls files. Alternatively, the legend can be supplied as a separate text file (README) and zipped together with the Table/Dataset file.

11) The paper explained: EMBO Molecular Medicine articles are accompanied by a summary of the articles to emphasize the major findings in the paper and their medical implications for the non-specialist reader. Please provide a draft summary of your article highlighting

- the medical issue you are addressing,

- the results obtained and

- their clinical impact.

This may be edited to ensure that readers understand the significance and context of the research. Please refer to any of our published articles for an example.

12) Author contributions: You will be asked to provide CRediT (Contributor Role Taxonomy) terms in the submission system. These replace a narrative author contribution section in the manuscript.

13) A Conflict of Interest statement should be provided in the main text.

14) Every published paper includes a 'Synopsis' to further enhance discoverability. Synopses are displayed on the journal webpage and are freely accessible to all readers. They include a short stand first (maximum of 300 characters, including space) as well as 2-5 one-sentences bullet points that summarizes the paper. Please write the bullet points to summarize the key NEW findings. They should be designed to be complementary to the abstract - i.e. not repeat the same text. We encourage inclusion of key acronyms and quantitative information (maximum of 30 words / bullet point). Please use the passive voice. Please attach these in a separate file or send them by email, we will incorporate them accordingly.

Please also suggest a striking image or visual abstract to illustrate your article as a PNG file 550 px wide x 300-600 px high.

15) Include a Reagents and Tools Table as part of the Methods section, which can be downloaded from our author guidelines.

EMBO Molecular Medicine has a "scooping protection" policy, whereby similar findings that are published by others during review or revision are not a criterion for rejection. Should you decide to submit a revised version, I do ask that you get in touch after three months if you have not completed it, to update us on the status.

*Additional important information regarding Figures

Each figure should be given in a separate file and should have the following resolution:
Graphs 800-1,200 DPI
Photos 400-800 DPI
Colour (only CMYK) 300-400 DPI"

Figures are not edited by the production team. All lettering should be the same size and style; figure panels should be indicated

by capital letters (A, B, C etc). Gridlines are not allowed except for log plots. Figures should be numbered in the order of their appearance in the text with Arabic numerals. Each Figure must have a separate legend and a caption is needed for each panel.

*Additional important information regarding figures and illustrations can be found at https://media.springernature.com/original/springer-cms/rest/v1/content/27825798/data/v1

***** Reviewer's comments *****

Referee #1 (Comments on Novelty/Model System for Author):

The experiments are well-performed and clearly presented. As already mentioned, the approach is not new, but I agree with the authors that this work may be of high medical impact. The mouse model used in the study is adequate.

Referee #1 (Remarks for Author):

The authors made an effort to address my concerns, and I have no further comments at this stage.

Referee #3 (Remarks for Author):

The authors have adequately addressed my previous concerns and have improved the overall clarity and included additional information to emphasize the importance of this work. However, I do have one additional minor concern for the authors. The dose of 5e12vg/kg is still considered low for treating the CNS via the IV route. For example, in the case of Canavan disease, the low dose cohort for a Phase 1/2 clinical trial is 1.32e14 vg/kg. I would consider including a sentence or two in the discussion describing future efforts should implement a dose escalating study to identify the maximum tolerated dose (MTD) that would lead to a full rescue of CNS related deficits in the mouse model.

***** Reviewer's comments *****

Referee #1 (Comments on Novelty/Model System for Author):
The experiments are well-performed and clearly presented. As already mentioned, the approach is not new, but I agree with the authors that this work may be of high medical impact. The mouse model used in the study is adequate.

Referee #1 (Remarks for Author):
The authors made an effort to address my concerns, and I have no further comments at this stage.

We thank the Referee 1 the time spent to review our manuscript.

Referee #3 (Remarks for Author):
The authors have adequately addressed my previous concerns and have improved the overall clarity and included additional information to emphasize the importance of this work. However, I do have one additional minor concern for the authors. The dose of 5e12vg/kg is still considered low for treating the CNS via the IV route. For example, in the case of Canavan disease, the low dose cohort for a Phase 1/2 clinical trial is 1.32e14 vg/kg. I would consider including a sentence or two in the discussion describing future efforts should implement a dose escalating study to identify the maximum tolerated dose (MTD) that would lead to a full rescue of CNS related deficits in the mouse model.

We agree with the reviewer that, unless the efficiency of the vector to cross the blood brain barrier (BBB) is substantially improved, the dose we tested in our study would be insufficient to reach the CNS in patients after intravenous injection. The need to improve the efficiency to cross the BBB in humans was already mentioned in the previous version of the manuscript (pages 16-17, lines 371 – 382 in the version submitted now), and following the suggestion of the reviewer, we have now added at the end of this paragraph a sentence indicating the need to conduct dose-escalation studies to find the maximum tolerated dose that will allow us to fully restore the CNS phenotype in the mice.

We also thank the Referee 3 the time spent to review our manuscript.

1st Apr 2026

Dear Dr. Marti,

We are pleased to inform you that your manuscript is accepted for publication and is now being sent to our publisher to be included in the next available issue of EMBO Molecular Medicine.

Your manuscript will be processed for publication by EMBO Press. It will be copy edited and you will receive page proofs prior to publication. Please note that you will be contacted by Springer Nature Author Services to complete licensing and payment information.

You may qualify for financial assistance for your publication charges - either via a Springer Nature fully open access agreement or an EMBO initiative. Check your eligibility: https://link.springer.com/journal/44321/how-to-publish-with-us

Should you be planning a Press Release on your article, please get in contact with embo_production@springernature.com as early as possible in order to coordinate publication and release dates.

If you have any questions, please do not hesitate to contact the Editorial Office. Thank you for your contribution to EMBO Molecular Medicine.

Yours sincerely,
Zeljko Durdevic

Zeljko Durdevic
Senior Editor
EMBO Molecular Medicine

>>> Please note that it is EMBO Molecular Medicine policy for the transcript of the editorial process (containing referee reports and your response letter) to be published as an online supplement to each paper. If you do NOT want this, you will need to inform the Editorial Office via email immediately. More information is available here: https://link.springer.com/partners/embo-press/editorial-policies#Peer%20review

EMBO Press Author Checklist

Corresponding Author Name: Ramon Martí	
Journal Submitted to: EMBO Molecular Medicine	
Manuscript Number: EMM-2025-22734	

USEFUL LINKS FOR COMPLETING THIS FORM
The EMBO Journal - Author Guidelines
EMBO Reports - Author Guidelines
Molecular Systems Biology - Author Guidelines
EMBO Molecular Medicine - Author Guidelines

Reporting Checklist for Life Science Articles (updated January
This checklist is adapted from Materials Design Analysis Reporting (MDAR) Checklist for Authors. MDAR establishes a minimum set of requirements in transparent reporting in the life sciences (see Statement of Task: 10.31222/osf.io/9sm4x). Please follow the journal's guidelines in preparing your
Please note that a copy of this checklist will be published alongside your article.

Abridged guidelines for figures
1. Data
The data shown in figures should satisfy the following conditions:
- the data were obtained and processed according to the field's best practice and are presented to reflect the results of the experiments in an accurate and unbiased manner.
- ideally, figure panels should include only measurements that are directly comparable to each other and obtained with the same assay.
- plots include clearly labeled error bars for independent experiments and sample sizes. Unless justified, error bars should not be shown for technical
- if n<5, the individual data points from each experiment should be plotted. Any statistical test employed should be justified.
- Source Data should be included to report the data underlying figures according to the guidelines set out in the authorship guidelines on Data

2. Captions
Each figure caption should contain the following information, for each panel where they are relevant:
- a specification of the experimental system investigated (eg cell line, species name).
- the assay(s) and method(s) used to carry out the reported observations and measurements.
- an explicit mention of the biological and chemical entity(ies) that are being measured.
- an explicit mention of the biological and chemical entity(ies) that are altered/varied/perturbed in a controlled manner.
- the exact sample size (n) for each experimental group/condition, given as a number, not a range;
- a description of the sample collection allowing the reader to understand whether the samples represent technical or biological replicates (including how many animals, litters, cultures, etc.).
- a statement of how many times the experiment shown was independently replicated in the laboratory.
- definitions of statistical methods and measures:
  - common tests, such as t-test (please specify whether paired vs. unpaired), simple χ2 tests, Wilcoxon and Mann-Whitney tests, can be unambiguously identified by name only, but more complex techniques should be described in the methods section;
  - are tests one-sided or two-sided?
  - are there adjustments for multiple comparisons?
  - exact statistical test results, e.g., P values = x but not P values < x;
  - definition of 'center values' as median or average;
  - definition of error bars as s.d. or s.e.m.

Please complete ALL of the questions below.
Select "Not Applicable" only when the requested information is not relevant for your study.

Materials

**Newly Created Materials**	**Information included in the manuscript?**	**In which section is the information available?** (Reagents and Tools Table, Materials and Methods, Figures, Data Availability Section)
New materials and reagents need to be available; do any restrictions apply?	Yes	Reagents and Tools table and Materials and Methods section. No restrictions apply

**Antibodies**	**Information included in the manuscript?**	**In which section is the information available?** (Reagents and Tools Table, Materials and Methods, Figures, Data Availability Section)
For **antibodies** provide the following information:<br>- Commercial antibodies: RRID (if possible) or supplier name, catalogue number and or/clone number<br>- Non-commercial: RRID or citation	Yes	Reagents and Tools table and Materials and Methods section.

**DNA and RNA sequences**	**Information included in the manuscript?**	**In which section is the information available?** (Reagents and Tools Table, Materials and Methods, Figures, Data Availability Section)
**Short novel DNA or RNA including primers, probes**: provide the sequences.	Yes	Reagents and Tools table and Materials and Methods section.

**Cell materials**	**Information included in the manuscript?**	**In which section is the information available?** (Reagents and Tools Table, Materials and Methods, Figures, Data Availability Section)
**Cell lines:** Provide species information, strain. Provide accession number in repository **OR** supplier name, catalog number, clone number, and/**OR** RRID.	Yes	Reagents and Tools table and Materials and Methods section.
**Primary cultures:** Provide species, strain, sex of origin, genetic modification status.	Yes	Reagents and Tools table and Materials and Methods section.
Report if the cell lines were recently **authenticated** (e.g., by STR profiling) and tested for mycoplasma contamination.	Yes	Materials and Methods section.

**Experimental animals**	**Information included in the manuscript?**	**In which section is the information available?** (Reagents and Tools Table, Materials and Methods, Figures, Data Availability Section)
**Laboratory animals or Model organisms:** Provide species, strain, sex, age, genetic modification status. Provide accession number in repository **OR** supplier name, catalog number, clone number, **OR** RRID.	Yes	Reagents and Tools table and Materials and Methods section.
**Animal observed in or captured from the field:** Provide species, sex, and age where possible.	Not Applicable	
Please detail **housing and husbandry conditions**.	Yes	Materials and Methods section.

**Plants and microbes**	**Information included in the manuscript?**	**In which section is the information available?** (Reagents and Tools Table, Materials and Methods, Figures, Data Availability Section)
**Plants:** provide species and strain, ecotype and cultivar where relevant, unique accession number if available, and source (including location for collected wild specimens).	Not Applicable	
**Microbes:** provide species and strain, unique accession number if available, and source.	Not Applicable	

**Human research participants**	**Information included in the manuscript?**	**In which section is the information available?** (Reagents and Tools Table, Materials and Methods, Figures, Data Availability Section)
If collected and within the bounds of privacy constraints report on age, sex and gender or ethnicity for all study participants.	Not Applicable	

**Core facilities**	**Information included in the manuscript?**	**In which section is the information available?** (Reagents and Tools Table, Materials and Methods, Figures, Data Availability Section)
If your work benefited from core facilities, was their service mentioned in the acknowledgments section?	Yes	Acknowledgements section

Design

Study protocol	Information included in the manuscript?	In which section is the information available? (Reagents and Tools Table, Materials and Methods, Figures, Data Availability Section)
If study protocol has been **pre-registered, provide DOI in the manuscript**. For clinical trials, provide the trial registration number **OR** cite DOI.	Not Applicable	
Report the **clinical trial registration number** (at ClinicalTrials.gov or equivalent), where applicable.	Not Applicable	

Laboratory protocol	Information included in the manuscript?	In which section is the information available? (Reagents and Tools Table, Materials and Methods, Figures, Data Availability Section)
Provide DOI OR other citation details if **external detailed step-by-step protocols** are available.	Not Applicable	

Experimental study design and statistics	Information included in the manuscript?	In which section is the information available? (Reagents and Tools Table, Materials and Methods, Figures, Data Availability Section)
Include a statement about **sample size** estimate even if no statistical methods were used.	Yes	Materials and Methods section.
Were any steps taken to minimize the effects of subjective bias when allocating animals/samples to treatment (e.g. **randomization procedure**)? If yes, have they been described?	Yes	Materials and Methods section.
Include a statement about **blinding** even if no blinding was done.	Yes	Materials and Methods section.
Describe **inclusion/exclusion criteria** if samples or animals were excluded from the analysis. Were the criteria pre-established? If sample or data points were omitted from analysis, report if this was due to attrition or intentional exclusion and provide justification.	Not Applicable	
For every figure, are **statistical tests** justified as appropriate? Do the data meet the assumptions of the tests (e.g., normal distribution)? Describe any methods used to assess it. Is there an estimate of variation within each group of data? Is the variance similar between the groups that are being statistically compared?	Yes	Materials and Methods section and Figure legends.

Sample definition and in-laboratory replication	Information included in the manuscript?	In which section is the information available? (Reagents and Tools Table, Materials and Methods, Figures, Data Availability Section)
In the figure legends: state number of times the experiment was **replicated** in laboratory.	Yes	Figure legends
In the figure legends: define whether data describe **technical or biological replicates**.	Yes	Figure legends

Ethics

Ethics	Information included in the manuscript?	In which section is the information available? (Reagents and Tools Table, Materials and Methods, Figures, Data Availability Section)
Studies involving **human participants**: State details of **authority granting ethics approval** (IRB or equivalent committee(s), provide reference number for approval.	Not Applicable	
Studies involving **human participants**: Include a statement confirming that **informed consent** was obtained from all subjects and that the experiments conformed to the principles set out in the WMA Declaration of Helsinki and the Department of Health and Human Services Belmont Report.	Not Applicable	
Studies involving **human participants: For** publication of **patient photos**, include a statement confirming that consent to publish was obtained.	Not Applicable	
Studies involving experimental **animals**: State details of **authority granting ethics approval** (IRB or equivalent committee(s), provide reference number for approval. Include a statement of compliance with ethical regulations.	Yes	Materials and Methods section
Studies involving **specimen and field samples:** State if relevant **permits** obtained, provide details of authority approving study; if none were required, explain why.	Not Applicable	

Dual Use Research of Concern (DURC)	Information included in the manuscript?	In which section is the information available? (Reagents and Tools Table, Materials and Methods, Figures, Data Availability Section)
Could your study fall under dual use research restrictions? Please check biosecurity documents and list of **select agents and toxins** (CDC): https://www.selectagents.gov/sat/list.htm	Not Applicable	
If you used a select agent, is the security level of the lab appropriate and reported in the manuscript?	Not Applicable	
If a study is subject to dual use research of concern regulations, is the name of the **authority granting approval and reference number** for the regulatory approval provided in the manuscript?	Not Applicable	

Reporting

The MDAR framework recommends adoption of discipline-specific guidelines, established and endorsed through community initiatives. Journals have their own policy about requiring specific guidelines and recommendations to complement MDAR.

Adherence to community standards	Information included in the manuscript?	In which section is the information available? (Reagents and Tools Table, Materials and Methods, Figures, Data Availability Section)
State if relevant guidelines or checklists (e.g., **ICMJE, MIBBI, ARRIVE, PRISMA**) have been followed or provided.	Not Applicable	
For **tumor marker prognostic studies,** we recommend that you follow the **REMARK** reporting guidelines (see link list at top right). See author guidelines, under 'Reporting Guidelines'. Please confirm you have followed these guidelines.	Not Applicable	
For **phase II and III randomized controlled trials**, please refer to the **CONSORT** flow diagram (see link list at top right) and submit the CONSORT checklist (see link list at top right) with your submission. See author guidelines, under 'Reporting Guidelines'. Please confirm you have submitted this list.	Not Applicable	

Data Availability

Data availability	Information included in the manuscript?	In which section is the information available? (Reagents and Tools Table, Materials and Methods, Figures, Data Availability Section)
Have **primary datasets** been deposited according to the journal's guidelines (see 'Data Deposition' section) and the respective accession numbers provided in the Data Availability Section?	Not Applicable	
Were **human clinical and genomic datasets** deposited in a public access-controlled repository in accordance to ethical obligations to the patients and to the applicable consent agreement?	Not Applicable	
Are **computational models** that are central and integral to a study available without restrictions in a machine-readable form? Were the relevant accession numbers or links provided?	Not Applicable	
If publicly available data were reused, provide the respective **data citations in the reference list**.	Not Applicable	
